# A robust prognostic signature for hormone-positive node-negative breast cancer

**DOI:** 10.1186/gm496

**Published:** 2013-10-11

**Authors:** Obi L Griffith, François Pepin, Oana M Enache, Laura M Heiser, Eric A Collisson, Paul T Spellman, Joe W Gray

**Affiliations:** 1 Department of Cancer and DNA Damage Responses, Life Sciences Division, Lawrence Berkeley National Laboratory, One Cyclotron Rd, Berkeley 94720, CA, USA; 2 Current affiliation: Department of Medicine, Division of Oncology, The Genome Institute, Washington University, Campus Box 8501, 4444 Forest Park Ave, St. Louis 63108, MO, USA; 3 Current affiliation: Sequenta Inc., 400 East Jamie Court, Suite 301, South San Francisco 94080, CA, USA; 4 Department of Biomedical Engineering, Center for Spatial Systems Biomedicine, Knight Cancer Institute, Oregon Health and Science University, 3303 SW Bond Ave, Portland, 97239, OR, USA; 5 Division of Hematology/Oncology, University of California San Francisco, 505 Parnassus Avenue, San Francisco 94143, CA, USA; 6 Current affiliation: Department of Molecular and Medical Genetics, Oregon Health and Science University, 3181 SW Sam Jackson Park Rd, Portland 97239, OR, USA

## Abstract

**Background:**

Systemic chemotherapy in the adjuvant setting can cure breast cancer in some patients that would otherwise recur with incurable, metastatic disease. However, since only a fraction of patients would have recurrence after surgery alone, the challenge is to stratify high-risk patients (who stand to benefit from systemic chemotherapy) from low-risk patients (who can safely be spared treatment related toxicities and costs).

**Methods:**

We focus here on risk stratification in node-negative, ER-positive, HER2-negative breast cancer. We use a large database of publicly available microarray datasets to build a random forests classifier and develop a robust multi-gene mRNA transcription-based predictor of relapse free survival at 10 years, which we call the Random Forests Relapse Score (RFRS). Performance was assessed by internal cross-validation, multiple independent data sets, and comparison to existing algorithms using receiver-operating characteristic and Kaplan-Meier survival analysis. Internal redundancy of features was determined using k-means clustering to define optimal signatures with smaller numbers of primary genes, each with multiple alternates.

**Results:**

Internal OOB cross-validation for the initial (full-gene-set) model on training data reported an ROC AUC of 0.704, which was comparable to or better than those reported previously or obtained by applying existing methods to our dataset. Three risk groups with probability cutoffs for low, intermediate, and high-risk were defined. Survival analysis determined a highly significant difference in relapse rate between these risk groups. Validation of the models against independent test datasets showed highly similar results. Smaller 17-gene and 8-gene optimized models were also developed with minimal reduction in performance. Furthermore, the signature was shown to be almost equally effective on both hormone-treated and untreated patients.

**Conclusions:**

RFRS allows flexibility in both the number and identity of genes utilized from thousands to as few as 17 or eight genes, each with multiple alternatives. The RFRS reports a probability score strongly correlated with risk of relapse. This score could therefore be used to assign systemic chemotherapy specifically to those high-risk patients most likely to benefit from further treatment.

## Background

Large randomized trials have shown that chemotherapy administered in the perioperative setting (for example, adjuvant chemotherapy) can cure patients otherwise destined to recur with systemic, incurable cancer [1]. Once this recurrence has happened, the same chemotherapy is never curative. Therefore, the adjuvant window is a privileged period of time, when the decision to administer additional therapy or not, as well as the type, duration, and intensity of such therapy takes center stage. Node-negative, ER-positive, HER2-negative breast cancer patients have a favorable prognosis when treated with adjuvant hormonal therapy only, but a fraction of such patients recur locally or systemically. Most patients are currently treated not only with hormonal therapy but also cytotoxic chemotherapy, even though it is probably unnecessary for most [1,2]. Our goal was to stratify these patients with respect to the likelihood of recurrence within 10 years after surgery. Earlier approaches to this problem were developed using relatively small datasets. Here we have explored the extent to which larger and more current meta-datasets can be used to improve predictor performance. Our approach was to develop a multi-gene transcription-level-based classifier of 10-year-relapse (disease recurrence within 10 years) using a large database of existing, publicly available microarray datasets. Existing solutions have been implemented on costly platforms that are slow to return results to the patient. However, new assay technologies exist which could make breast cancer prognostics much more accessible and timely. Some of these potential assay technologies can only measure transcription of a relatively small number of genes while still optimizing cost and efficiency. To this end, we developed a robust prognostic panel offering flexibility in both the number and identity of genes utilized from thousands to as few as eight genes, each with multiple alternatives to maximize the chance of successful migration to other assay technologies.

## Methods

### Literature search and curation

**Figure 1:**
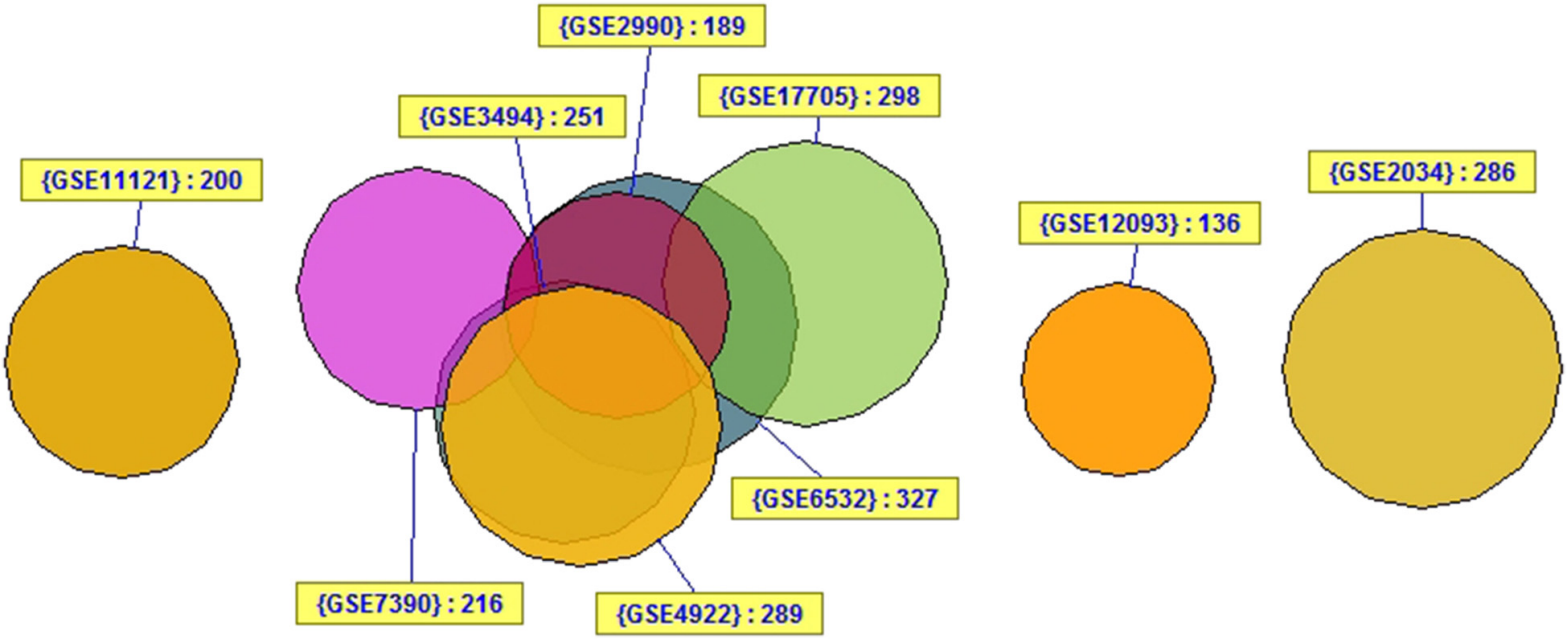
**Duplicate analysis showing approximate relationship between studies in analysis.** The VennMaster diagram shows the approximate overlap between GEO datasets used in the current study. Three studies show zero overlap while the other six show significant overlap.

**Figure 2:**
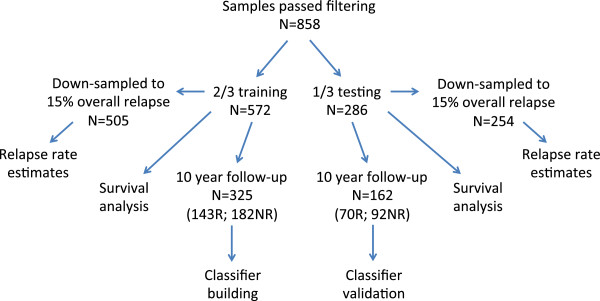
**Sample breakdown.** A total of 858 samples passed all filtering steps including 487 samples with 10-year follow-up data (213 relapse; 274 no relapse). The remaining 371 samples had insufficient follow-up for 10-year classification analysis but were retained for use in survival analysis. The 858 samples were broken into two-thirds training and one-third testing sets resulting in: a training set of 572 samples for use in survival analysis and 325 samples with 10-year follow-up (143 relapse; 182 no relapse) for classification analysis; and a testing set of 286 samples for use in survival analysis and 162 samples with 10-year follow-up (70 relapse; 92 no relapse) for classification analysis.

Studies were collected which provided gene expression data for ER+, LN-, HER2- patients with no systemic chemotherapy (hormonal therapy was allowed). Each study was required to have a sample size of at least 100, report LN status, and include time and events for either recurrence-free survival or distant metastasis-free survival. The latter were grouped together for survival analysis where all events represent either a local or distant relapse of disease. If ER or HER2 status were not reported, they were determined by array, but preference was given to studies with clinical determination first. A minimum of 10 years follow-up was required for training the classifier. However, patients with shorter follow-up were included in survival analyses. Patients with immediately postoperative events (time = 0) were excluded. Nine studies [3]–[11] meeting the above criteria were identified by searching PubMed and the Gene Expression Omnibus (GEO) database [12]. To allow combination of the largest number of samples, only the common Affymetrix U133A gene expression platform was used. A total of 2,175 breast cancer samples were identified. After filtering for only those samples which were ER+, node-negative, and had not received systemic chemotherapy, 1,403 samples remained. Duplicate analysis removed a further 405 samples due to the significant amount of redundancy between studies (Figure 1). Filtering for ER + and HER2- status using array determinations eliminated another 140 samples (51 ER-, 72 HER2+, 17 ER-/HER2+, Additional file 1: Figure S1). Some ER- samples were from the Schmidt (2008) dataset (31/201) which did not provide clinical ER status and thus for that study we relied solely on arrays for determination of ER status. However, there were also a small number (37/760) from the remaining studies, which represent discrepancies between array status and clinical determination. In such cases, both the clinical and array-based determinations were required to be positive for inclusion in further analysis. A total of 858 samples passed all filtering steps including 487 samples with 10-year follow-up data (213 relapse; 274 no relapse). The remaining 371 samples had insufficient follow-up for 10-year classification analysis but were retained for use in survival analysis. None of the 858 samples were treated with systemic chemotherapy but 302 (35.2%) were treated with adjuvant hormonal therapy of which 95.4% were listed as tamoxifen. The 858 samples were broken into two-thirds training and one-third testing sets resulting in: a training set of 572 samples for use in survival analysis and 325 samples with 10 year-follow-up (143 relapse; 182 no relapse) for classification analysis; and a testing set of 286 samples for use in survival analysis and 162 samples with 10-year follow-up (70 relapse; 92 no relapse) for classification analysis. The numbers of samples available for classification analysis and survival analysis differed because the former was performed only on those samples that could be binarized into 'relapse’ or 'no relapse’ with 10 years of follow-up whereas the latter made use of all patients (with censoring), even those with no relapse but <10 years follow-up. Table 1 outlines the datasets used in the analysis and Figure 2 illustrates the breakdown of samples for analysis.

**Table 1:** Studies included in analysis

**Study**	**GSE**	**Total samples**	**ER+/LN-/ untreated**^**a**^**/outcome**	**Duplicates removed**	**ER+/HER2-array**	**10-year relapse**	**10-year no relapse**
Desmedt, 2007 [3]	GSE7390	198	135	135	116	42	60
Ivshina, 2006 [4]	GSE4922	290	133	2	2	0	2
Loi, 2007 [5]	GSE6532	327	170	43	40	10	5
Miller, 2005 [6]	GSE3494	251	132	115	100	30	52
Schmidt, 2008 [7]	GSE11121	200	200^b^	200	155	25	46
Sotiriou, 2006 [8]	GSE2990	189	113	48	45	12	15
Symmans, 2010 [9]	GSE17705	298	175	110	102	12	41
Wang, 2005 [10]	GSE2034	286	209	209	173	67	29
Zhang, 2009 [11]	GSE12093	136	136	136	125	15	24
Nine studies		2,175	1403	998	858	213	274

^a^Untreated includes some patients with adjuvant endocrine therapy alone or radiation.

^b^ER status not available, filtered later based on array expression data.

### Preprocessing

All data processing and analyses were completed with open source R/Bioconductor packages. Raw data (Cel files) were downloaded from GEO. Duplicate samples were identified and removed if they had the same database identifier (for example, GSM accession), same sample/patient ID, or showed a high correlation (r >0.99) compared to any other sample in the dataset. Raw data were normalized and summarized using the affy [13] and gcrma [14] packages in R/Bioconductor. Probe sets were mapped to Entrez gene symbols using both standard and custom annotation files [15]. ER and HER2 expression status was determined using standard probe sets. For the Affymetrix U133A array we and others have found the probe set '205225_at’ to be most effective for determining ER status [16]. The rank sum of the best probe sets for ERBB2 (216835_s_at), GRB7 (210761_s_at), STARD3 (202991_at), and PGAP3 (55616_at) was used to determine HER2 amplification status. This was calculated by taking the sum of individual expression level ranks of each of the four probes. Cutoff values for ER and HER2 status were chosen by mixed model clustering (mclust [17,18] package). Mclust performs normal mixture modeling, fitted via an expectation maximization algorithm. A mixture model is a probabilistic model for representing the presence of subpopulations within an overall population. In this case we hypothesized that two populations exist in the dataset (ER + and ER- or HER2+ and HER2-) and would be represented by two distinct distributions of expression values. This is clearly visible in Additional file 1: Figure S1 where both ER and HER2 expression levels apparently manifest as two distinct distributions. In both cases, mclust successfully identified two distributions and we used the maximum value of the first distribution as an unbiased, objective cutoff to separate the two distributions. Unsupervised clustering was performed to assess the extent of batch effects. Once all prefiltering was complete, data were randomly split into training (two-thirds) and test (one-thirds) datasets while balancing for study of origin and number of relapses with 10-year follow-up. The test dataset was renormalized, put aside, left untouched, and only used for final validation, once each for the full-gene, 17-gene, and eight-gene classifiers. Training data was renormalized together as above. Probe sets were then filtered for a minimum of 20% samples with expression above background threshold (raw value >100) and coefficient of variation (COV) between 0.7 and 10. A total of 3,048 probe sets/genes passed this filtering and formed the basis for the 'full-gene-set’ model described below. The clinical annotations and preprocessed data for both training and test datasets (before filtering for percent expression and COV) are provided as Additional files 2, 3, 4, and 5.

### Classification (training)

Classification was performed on only training samples with either a relapse or no relapse after 10-year follow-up using the randomForest [19] package. Forests were created with at least 100,001 trees (odd number ensures fully deterministic model) and otherwise default settings. Performance was assessed by area under the curve (AUC) of a receiver-operating characteristic (ROC) curve, calculated with the 'ROCR’ package, from Random Forests internal out-of-bag (OOB) testing results. In Random Forests, this OOB testing takes the place of cross-validation to get an unbiased estimate of the test set error. Each tree is constructed using a different random bootstrap sample of about two-thirds of cases from the original data. Each case left out in the construction of the tree is run through that tree to get a classification. In this way, a test set classification is obtained for each case in about one-third of the trees. At the end of the run, the software takes j to be the class that got the most votes every time case n was OOB. The proportion of times that j is not equal to the true class of n averaged over all cases is the OOB error estimate. By default, RF performs a binary classification (for example, relapse *versus* no relapse). However it also reports a probability (proportion of 'votes’) for relapse, which we call the Random Forests Relapse Score (RFRS). Risk group thresholds were determined from the distribution of relapse probabilities using mixed model clustering to set cutoffs for low, intermediate, and high-risk groups.

### Determination of optimal 17-gene and eight-gene sets

**Figure 3:**
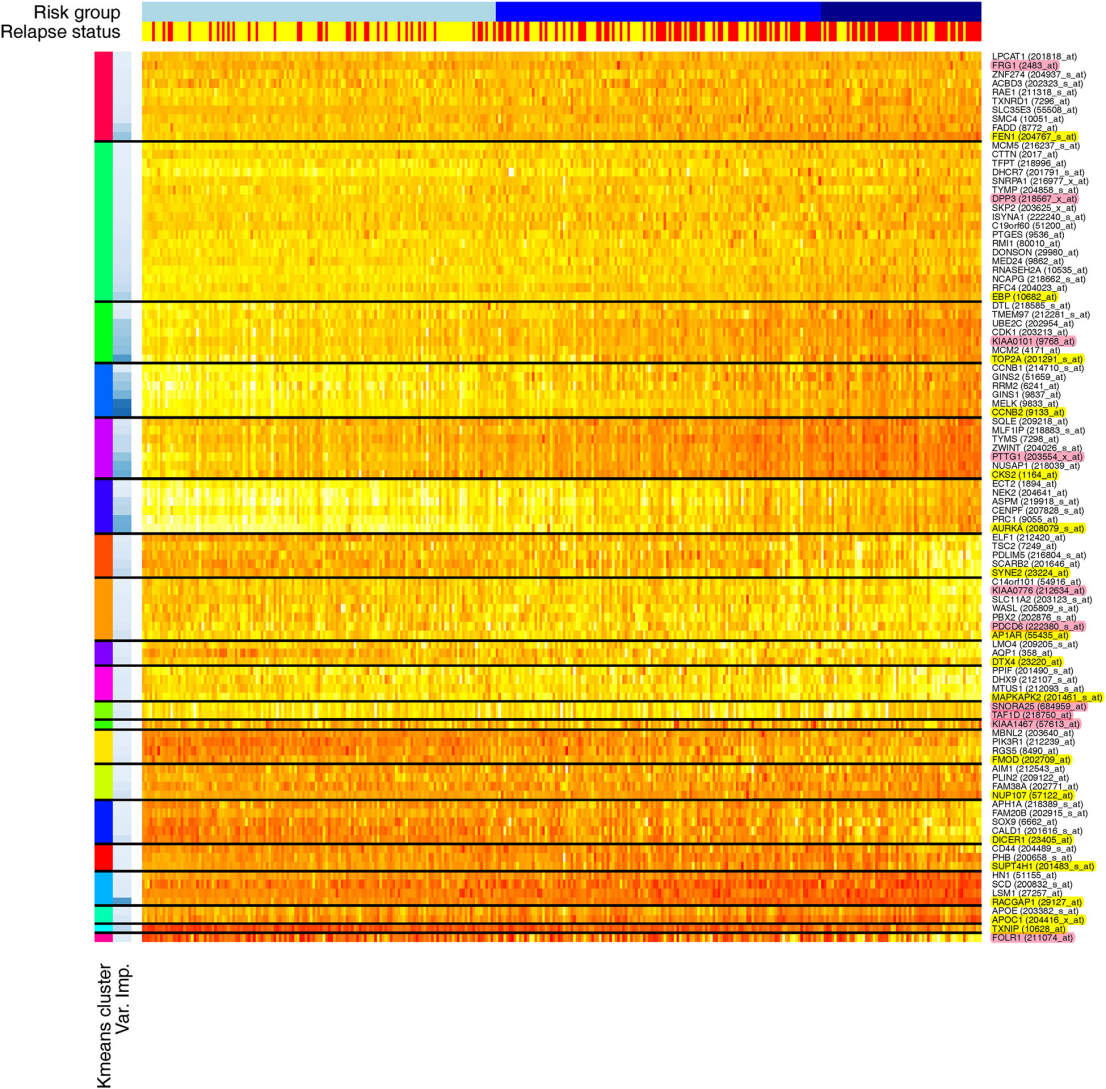
**Heatmap showing top 100 probe sets after k-means clustering (k = 20).** Training data (*n* = 325) were clustered by expression value using k-means clustering (k = 20) for the top 100 probe sets identified by random forest classification variable importance. The first color side bar on the left indicates cluster number and the second indicates relative variable importance within the cluster (darker blue = greater importance). The top side bars indicate risk group (low, intermediate, and high from left to right) and relapse status (red = relapse; yellow = no relapse). Genes (probe sets) are indicated on the right axis. Genes highlighted in yellow represent the primary genes in the model (best in each cluster). Genes not highlighted represent alternates to primary genes in each cluster. Genes highlighted in pink represent genes excluded from the model because of probe set sequence ambiguity or status as a hypothetical protein.

Initially an optimal set of 20 genes was selected by removing redundant probe sets and extracting the top 100 genes (by reported Gini variable importance), k-means clustering (k = 20) these genes and selecting the best gene from each cluster (again by variable importance) (Figure 3). Additional genes in each cluster will serve as robust alternates in case of failure to migrate primary genes to an assay platform. A gene might fail to migrate due to problems with prober/primer design or differences in the sensitivity of a specific assay for that gene. The top 100 genes/probe sets were also manually checked for sequence correctness by alignment to the reference genome (see Additional file 6). Seven genes/probe sets with ambiguous or erroneous alignments were excluded. Three genes/probe sets were also excluded because of their status as hypothetical proteins (KIAA0101, KIAA0776, KIAA1467). After these removals, three clusters were lost, leaving a set of 17 primary genes and 73 alternate genes. All but two primary genes have two or more alternates (TXNIP is without alternate, and APOC1 has a single alternate). Table 2 lists the final gene set, their top two alternate genes (where available) and their variable importance values (See Additional file 1: Table S1 for complete list). The above procedure was repeated to produce an optimal set of eight genes, this time starting from the top 90 non-redundant probe sets (excluding the 10 genes with problems identified above), k-means clustering (k = 8) these genes and selecting the best gene from each cluster (Additional file 1: Figure S2). All eight genes were also included in the 17-gene set and have at least two alternates (Table 3, Additional file 1: Table S2). Using the final optimized 17-gene and eight-gene sets as input, new RF models were built on training data. Actual probe sequences for the top 100 probe sets are provided in Additional file 7.

**Table 2:** The 17-gene RFRS signature

**Primary predictor**		**Alternate 1**		**Alternate 2**	
CCNB2	0.785	MELK	0.739	GINS1	0.476
TOP2A	0.590	MCM2	0.428	CDK1	0.379
RACGAP1	0.588	LSM1	0.139	SCD	0.125
CKS2	0.515	NUSAP1	0.491	ZWINT	0.272
AURKA	0.508	PRC1	0.499	CENPF	0.306
FEN1	0.403	FADD	0.313	SMC4	0.170
EBP	0.341	RFC4	0.264	NCAPG	0.234
TXNIP	0.292	N/A	N/A	N/A	N/A
SYNE2	0.270	SCARB2	0.225	PDLIM5	0.167
DICER1	0.209	CALD1	0.129	SOX9	0.125
AP1AR	0.201	PBX2	0.134	WASL	0.126
NUP107	0.197	FAM38A	0.165	PLIN2	0.110
APOC1	0.176	APOE	0.121	N/A	N/A
DTX4	0.164	AQP1	0.141	LMO4	0.120
FMOD	0.154	RGS5	0.120	PIK3R1	0.103
MAPKAPK2	0.151	MTUS1	0.136	DHX9	0.136
SUPT4H1	0.111	PHB	0.106	CD44	0.105

**Table 3:** The eight-gene RFRS signature

**Primary predictor**		**Alternate 1**		**Alternate 2**	
CCNB2	0.785	MELK	0.739	TOP2A	0.590
RACGAP1	0.588	TXNIP	0.292	APOC1	0.176
CKS2	0.515	NUSAP1	0.491	FEN1	0.403
AURKA	0.508	PRC1	0.499	CENPF	0.306
EBP	0.341	FADD	0.313	RFC4	0.264
SYNE2	0.270	SCARB2	0.225	PDLIM5	0.167
DICER1	0.209	FAM38A	0.165	FMOD	0.154
AP1AR	0.201	MAPKAPK2	0.151	MTUS1	0.136

### Validation (testing and survival analysis)

Survival analysis on all training data, now also including those patients with <10 years of follow-up, was performed with risk group as a factor, for the full-gene, 17-gene, and eight-gene models, using the 'survival’ package. Note, the risk scores and groups for samples used in training were assigned from internal OOB cross-validation. Only those patients not used in initial training (without 10-year follow-up) were assigned a risk score and group by *de novo* classification. Significance between risk groups was determined by Kaplan-Meier log rank test (with test for linear trend). To directly compare relapse rates at 10 years for each risk group to that reported in the OncotypeDX publication [20], the overall relapse rates in our patient cohort were randomly down-sampled to the same rate (15%) as in their cohort [20] and results averaged >1,000 iterations. To illustrate, the training dataset includes 572 samples with 143 relapse events (that is, 25.0% relapse rate). Samples with relapse events were randomly eliminated from the cohort until only 15% of remaining samples had relapse events (76/505 = 15%). This 'down-sampled’ dataset was then classified using the RFRS model to assign each sample to a risk group and the rates of relapse determined for each group. The entire down-sampling procedure was then repeated 1,000 times to obtain average estimated rates of relapse for each risk group given the overall rate of relapse of 15%. Setting the overall relapse rate to 15% is also useful because this more closely mirrors the general population rate of relapse. Without this down-sampling, expected relapse rates in each risk group would appear unrealistically high. See Figure 2 for explanation of the breakdown of samples into training and test sets used for classifier building and survival analysis.

Next, the full-gene, 17-gene, and eight-gene RF models along with risk group cutoffs were applied to the independent test data. The same performance metrics, survival analysis, and estimates of 10-year relapse rates were performed as above. The 17-gene model was also tested on the independent test data, stratified by treatment (untreated *vs.* hormone therapy treated), to evaluate whether performance of the signature was biased towards one patient subpopulation or the other. Finally, for direct performance comparison on the same datasets, the Oncotype DX algorithm [20] was implemented in R and applied to both training and test datasets.

The independent test data were not used in any way during the training phase. However, these samples represent a random subset of the same overall patient populations that were used in training. Therefore, they are not as fully independent as recommended by the Institute of Medicine 'committee on the review of omics-based tests for predicting patient outcomes in clinical trials’ [21]. Therefore, additional independent validations were performed against the NKI dataset [22] obtained from the Netherlands Cancer Institute [23] and the METABRIC dataset [24] accessed through Synapse [25]. The NKI data represent a set of 295 consecutive patients with primary stage I or II breast carcinomas. The dataset was filtered down to the 89 patients who were node-negative, ER-positive, HER2-negative, and not treated by systemic chemotherapy. Relapse times and events were defined by any of distant metastasis, regional recurrence or local recurrence. The METABRIC data represent a set of 1,992 primary breast tumors. The dataset was filtered down to the 315 patients who were node-negative, ER-positive, HER2-negative, and not treated by systemic chemotherapy. Relapse times and events were used as provided. Expression values from the NKI Agilent array data and METABRIC (Illumina HT 12v3) array were rescaled to the same distribution as that used in training using the 'preprocessCore’ package. Values for the eight-gene and 17-gene-set RFRS models were extracted for further analysis. If more than one Agilent or Illumina probe set could be mapped to an RFRS gene then the probe set with best performance was used. The full-gene-set model was not applied because not all Affymetrix-defined genes (probe sets) in the full-gene-set could be mapped to Agilent or Illumina-defined genes (probe sets). However, the 17-gene and eight-gene RFRS models were applied to NKI and METABRIC data to calculate predicted probabilities of relapse. Patients were divided into low-, intermediate-, and high-risk groups by ranking according to probability of relapse and then dividing so that the proportions in each risk group were identical to that observed in training. ROC AUC, survival *P* values, and estimated rates of relapse were then calculated as above. It should be noted that while the NKI clinical data described here (*n* = 89) had an average follow-up time of 9.55 years (excluding relapse events), 34 patients had a follow-up time of <10 years without an event (range, 1.78-9.83 years). Similarly, the METABRIC clinical data (*n* = 315) had an average follow-up time of 9.85 years (excluding relapse events) but 118 patients had a follow-up time of <10 years without an event (range, 0.05-9.80 years). These patients with <10 years of follow-up but no events would not have met our criteria for inclusion in the training dataset and likely include some events which have not occurred yet. If anything, this is likely to reduce the AUC estimate and underestimate *P* value significance in survival analysis.

### Selection of reference genes

While not necessary for Affymetrix, migration to other assay technologies (for example, RT-PCR approaches) may require highly and invariantly expressed genes to act as a reference for determining accurate gene expression level estimates. To this end, we developed two sets of reference genes. The first was chosen by the following criteria: (1) filtered if not expressed above background threshold (raw value >100) in 99% of samples; (2) filtered if not in top fifth percentile (overall) for mean expression; (3) filtered if not in top 10th percentile (remaining genes) for standard deviation; (4) ranked by coefficient of variation. The top 25 reference genes are listed in Additional file 1: Table S3 along with reference genes used by OncotypeDX. The second set of reference genes were chosen to represent three ranges of mean expression levels encompassed by genes in the 17-gene signature (low, 0-400; medium, 500-900; high, 1,200-1,600). For each mean expression range, genes were: (1) filtered if not expressed above background threshold (raw value >100) in 99% of samples; and (2) ranked by coefficient of variation. The top five genes from each range are listed in Additional file 1: Table S4. Reference genes underwent the same manual checks for sequence correctness by alignment to the reference genome as above (see Additional file 8). Five genes were marked for exclusion in the first set and three genes excluded from the second set. Actual probe sequences for all reference probe sets are provided in Additional file 9.

### Implementation of algorithm

The RFRS algorithm is implemented in the R programming language and can be applied to independent patient data. Input data are tab-delimited text files of normalized expression values with 17 or eight transcripts/genes as columns and patient(s) as rows. A sample patient data file (patient_data.txt) is presented in Additional file 10. A sample R program (RFRS_sample_code.R) for running the algorithm is presented in Additional file 10. The RFRS algorithm consists of a Random Forest of 100,001 decision trees. This is precomputed, provided as an R data object (see Additional file 11) and must be included in the working directory. Each node (branch) in each tree represents a binary decision based on transcript levels for transcripts described above. Based on these decisions, the patient is assigned to a terminal leaf of each decision tree, representing a vote for either 'relapse’ or 'no relapse’. The fraction of votes for 'relapse’ to votes for 'no relapse’ represents the RFRS - a measure of the probability of relapse. If RFRS is ≥0.606 the patient is assigned to the 'high-risk’ group, if ≥0.333 and <0.606 the patient is assigned to the ’intermediate-risk’ group, and if <0.333 the patient is assigned to the 'low-risk’ group. These cutoffs were chosen by mixed-model clustering during the training phase. The patient’s RFRS value is also used to determine a likelihood of relapse by comparison to a loess fit of RFRS *versus* likelihood of relapse for the training dataset. Precomputed R data objects for the loess fit (RelapseProbabilityFit.Rdata) and summary plot (RelapseProbabilityPlot.Rdata) are loaded from file (see Additional file 12). The patient’s estimated likelihood of relapse is determined, added to the summary plot, and output as a new report (see Additional file 13). A complete list of GEO sample identifiers, used for training and testing (Additional file 14), and additional sample R code (Additional file 15), are provided to help others reproduce the RFRS implementation.

## Results and discussion

Internal OOB cross-validation for the initial (full-gene-set) model on training data reported an ROC AUC of 0.704. This was comparable or better than those reported by Johannes et al. (2010) who tested a number of different classifiers on a smaller subset of the same data and found AUCs of 0.559 to 0.671 [26]. It also compares favorably to the AUC value of 0.688 when the OncotypeDX algorithm was applied to this same training dataset.

**Figure 4:**
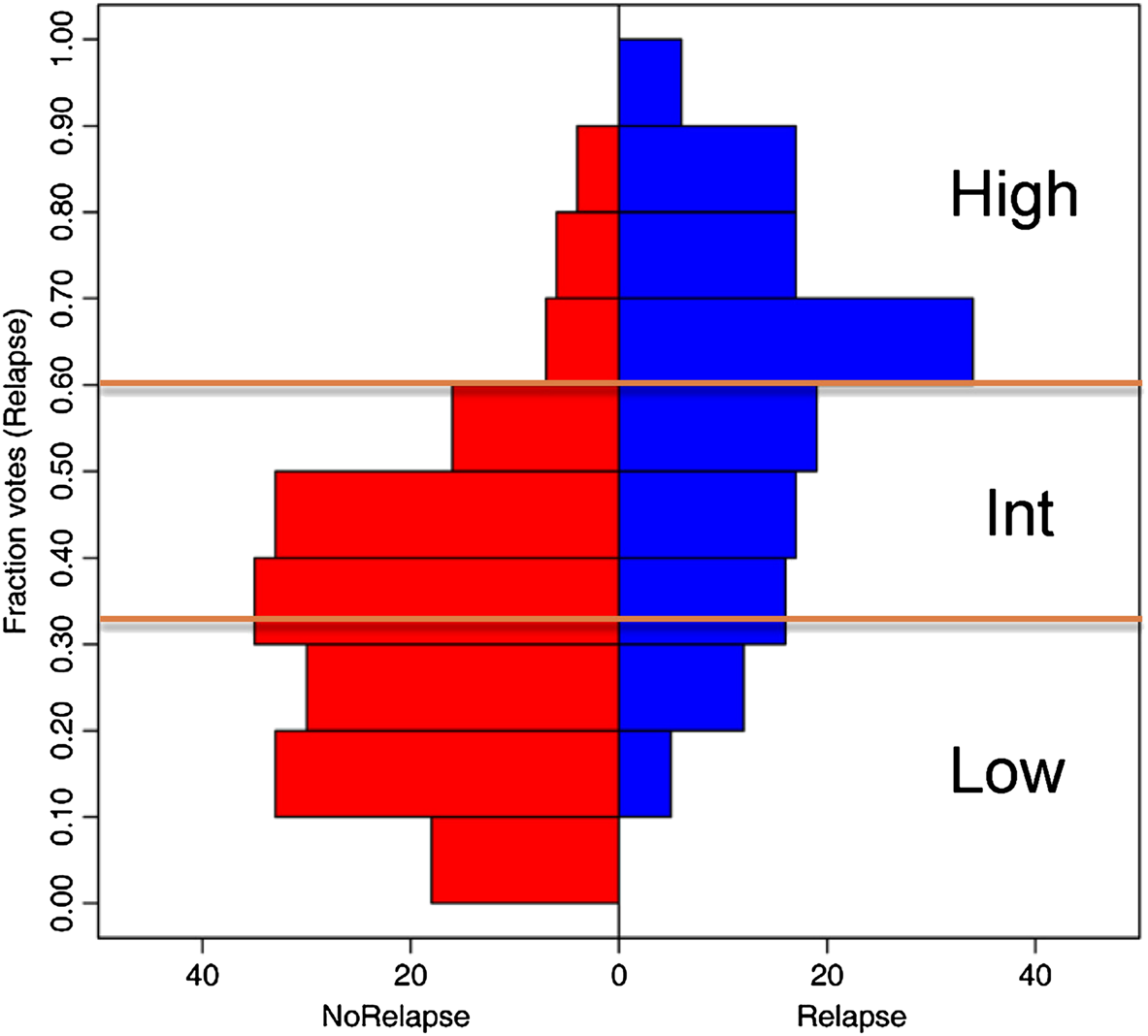
**Risk group threshold determination.** The distribution of RFRS scores was determined for patients in the training dataset (*n* = 325) comparing those with a known relapse (right side; in blue) *versus* those with no known relapse (left side; in red). As expected, patients without a known relapse tend to have a higher predicted likelihood of relapse (by RFRS) and *vice versa*. Mixed model clustering was used to identify thresholds (0.333 and 0.606) for defining low-, intermediate-, and high-risk groups as indicated.

**Figure 5:**
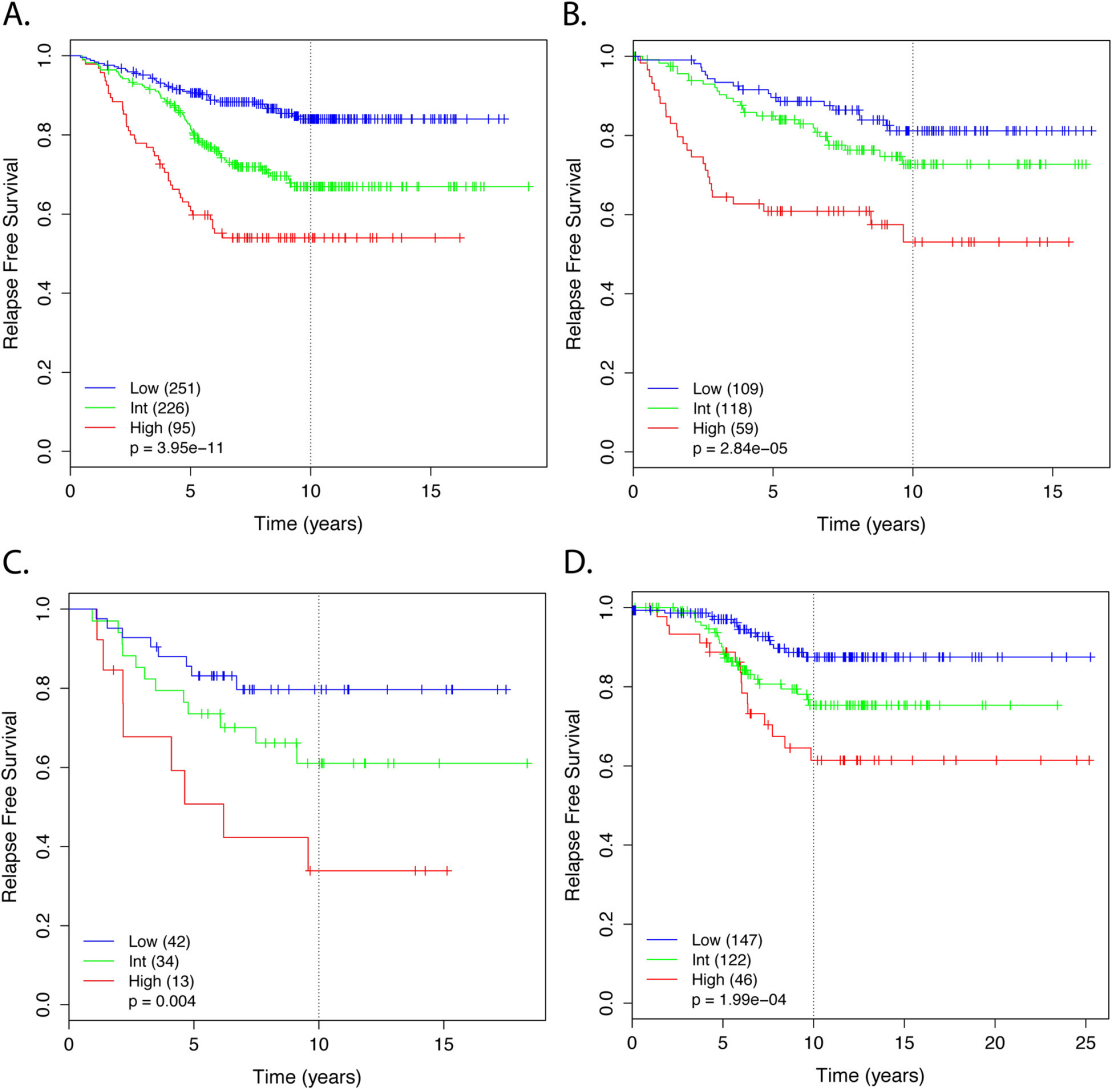
**Likelihood of relapse according to RFRS group.** Kaplan-Meier survival analysis shows a significant difference in relapse-free survival for low-, intermediate-, and high-risk groups as defined by: **(A)** the full-gene-set model on training data (*n* = 572, *P* = 3.95E-11); **(B)** the eight-gene-set model on independent test data (*n* = 286, *P* = 2.84E-05); **(C)** the eight-gene-set model on independent NKI data (*n* = 89, *P* = 0.004); **(D)** the 17-gene-set model on independent METABRIC data (*n* = 315, *P* <1.99E-04). Note, for panel **A**, the risk scores and corresponding groups for samples used in classifier training (*n* = 325) were assigned from internal OOB cross-validation. Only those patients not used in initial training (training data without 10-year follow-up; test data) were assigned a risk score and group by *de novo* classification. Significance between risk groups was determined by Kaplan-Meier log rank test (with test for linear trend).

Mixed model clustering analysis identified three risk groups with probabilities for low-risk <0.333; intermediate-risk 0.333-0.606; and high-risk ≥0.606 (Figure 4). Survival analysis determined a highly significant difference in relapse rate between risk groups (*P* = 3.95E-11; Figure 5A). After down-sampling to a 15% overall rate of relapse, approximately 46.7% (*n* = 235) of patients were placed in the low-risk group and were found to have a 10-year risk of relapse of only 8.0%. Similarly, 38.6% (*n* = 195) and 14.9% (*n* = 75) of patients were placed in the intermediate- and high-risk groups with rates of relapse of 17.6% and 30.3%, respectively. These results are very similar to those for OncotypeDX which were reported as 51% of patients in the low-risk category with a rate of distant recurrence at 10 years of 6.8% (95% CI, 4.0-9.6); 22% in intermediate-risk category with recurrence rate of 14.3% (95% CI, 8.3-20.3); and 27% in high-risk category with recurrence rate of 30.5% (95% CI, 23.6-37.4) [20].

Our goal was to identify a potential signature for migration to a more affordable and faster platform. We can expect a certain amount of attrition when migrating to such platforms. Therefore we developed a rational approach in which approximately 100 candidate signature genes were identified but organized into signatures of eight- and 17-gene sets with each gene having multiple alternates in case of platform migration failures. The signature sizes were chosen for practical reasons, with the assumption that high-throughput, low-cost platforms may have limitations to approximately 10 or 20 features and need space for one or more control/reference genes.

Validation of the models against the independent test dataset also showed very similar results to training estimates. The full-gene-set model had an AUC of 0.730 and the 17-gene and eight-gene optimized models had minimal reduction in performance with AUC of 0.715 and 0.690, respectively. Again, this compared favorably to the AUC value of 0.712 when the OncotypeDX algorithm was applied to the same test dataset. Survival analysis again found very significant differences between the risk groups for the full-gene (*P* = 6.54E-06), 17-gene (*P* = 9.57E-06), and eight-gene (*P* = 2.84E-05; Figure 5B) models. For the 17-gene model, approximately 38.2% (*n* = 97) of patients were placed in the low-risk group and were found to have a 10-year risk of relapse of only 7.8%. Similarly, 40.5% (*n* = 103) and 21.3% (*n* = 54) of patients were placed in the intermediate and high-risk groups with rates of relapse of 15.3% and 26.8%, respectively. Very similar results were observed for the full-gene and eight-gene models (Table 4). We have also compared performance to random gene sets of the same sizes (that is, eight- and 17-gene sets). Genes were chosen randomly from the total set of 3,048 genes which passed basic percent expression and COV filtering. On average, AUC values of 0.649 and 0.608 were estimated for random sets of 17 and eight genes compared to 0.715 and 0.690 for RFRS gene sets on the same independent test dataset. Only 2.2% of 17-gene and 3.6% of eight-gene random sets performed as well as those we chose. AURKA alone had an AUC of 0.623.

**Table 4:** Comparison of validation results in independent test data for full-gene-set, 17-gene, and eight-gene RFRS models

				**Relapse-free survival**				
**RFRS performance**		**Low risk**		**Intermediate risk**		**High risk**		
**Model**	**AUC**	**RR**	***n *** **(%)**	**RR**	***n *** **(%)**	**RR**	***n *** **(%)**	**KM ( *P *)**
**Full-gene-set**	0.730	6.9	78 (30.7)	15.8	133 (52.4)	26.8	43 (16.9)	6.54E-06
**17-gene**	0.715	7.8	97 (38.2)	15.3	103 (40.5)	26.8	54 (21.3)	9.57E-06
**Eight-gene**	0.690	9.7	101 (39.8)	13.9	105 (41.3)	28.3	48 (18.9)	2.84E-05

KM (*P*), p-value for Kaplan-Meier logrank test (with test for linear trend) from survival analysis; RR, relapse rate.

Validation against the additional, independent, NKI dataset also had very similar results. The 17-gene and eight-gene models had AUC values of 0.688 and 0.699, respectively, nearly identical to the results for the previous independent dataset. Differences between risk groups in survival analysis were also significant for both 17-gene (*P* = 0.023) and eight-gene (*P* = 0.004, Figure 5C) models. Similar results were also obtained from the validation against METABRIC data with 17-gene (AUC = 0.628, *P* <1.99E-04, Figure 5D) and eight-gene models (AUC = 0.642, *P* <0.04). It should be noted that any level of performance observed on independent test datasets is somewhat disadvantaged if they make use of different expression platforms (as NKI and METABRIC datasets do). Even with renormalizing or rescaling, the cutoffs and inter-variable relationships will likely be sensitive to changes in the inherent distribution and dynamic range that accompany another technology. When migrating to a new platform, an additional training phase should be performed. Our signatures and their alternates provide a convenient candidate list for development on such a new platform.

**Figure 6:**
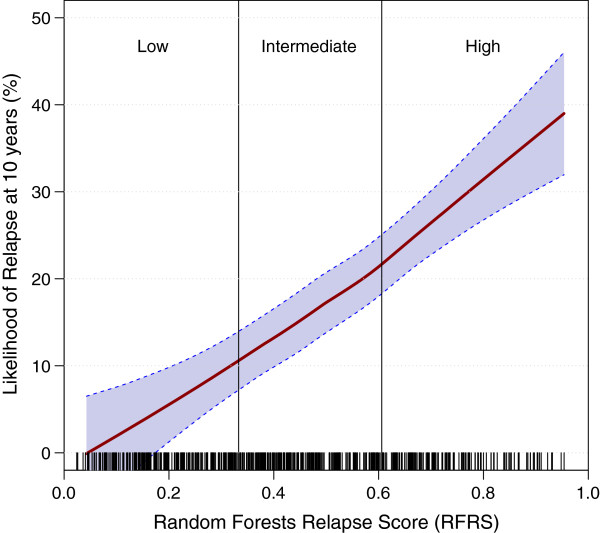
**Estimated likelihood of relapse at 10 years for any RFRS value.** The likelihood of relapse was calculated in the training dataset (*n* = 505) for 50 RFRS intervals (from 0 to 1). A smooth curve was fitted using a loess function and 95% confidence intervals plotted to represent the error in the fit. Short vertical marks just above the x axis, one for each patient, represent the distribution of RFRS values observed in the training data. Thresholds for risk groups are indicated. The plot shows a linear relationship between RFRS and likelihood of relapse at 10 years with the likelihood ranging from approximately 0 to 40%.

The trend between risk group and rate of relapse continued if groups were broken down further (using training data) into five equal groups instead of the three groups defined above (Additional file 1: Figure S4). This observation is consistent with the idea that the RFRS is a quantitative, linear measure directly related to probability of relapse. Figure 6 shows the likelihood of relapse at 10 years, calculated for 50 RFRS intervals (from 0 to 1), with a smooth curve fitted, using a loess function and 95% confidence intervals representing error in the fit. The distribution of RFRS values observed in the training data is represented by short vertical marks just above the x axis, one for each patient. From this training data we can estimate that 18.9% of patients from a population with an overall 15% relapse rate will be predicted to have a 5% or lower risk of relapse. Similarly, 37.0% will have a 10% or lower risk of relapse.

In order to maximize the total size of our training dataset we allowed samples to be included from both untreated patients and those who received adjuvant hormonal therapy such as tamoxifen. Since outcomes likely differ between these two groups, and they may represent fundamentally different subpopulations, it is possible that performance of our predictive signatures is biased towards one group or the other. To assess this issue we performed validation against the independent test dataset, stratified by treatment status, using the 17-gene model. Both groups were found to have comparable AUC values with the slightly better value of 0.740 for hormone-treated *versus* 0.709 for untreated. Survival curves were also highly similar and significant with *P* value of 0.004 and 3.76E-07 for treated and untreated respectively (Additional file 1: Figure S5A and S5B). The difference in *P* value appears more likely due to differences in the respective sample sizes than actual difference in survival curves. This is important because it predicts equal or better performance of the signature in hormonal-therapy treated patients.

**Figure 7:**
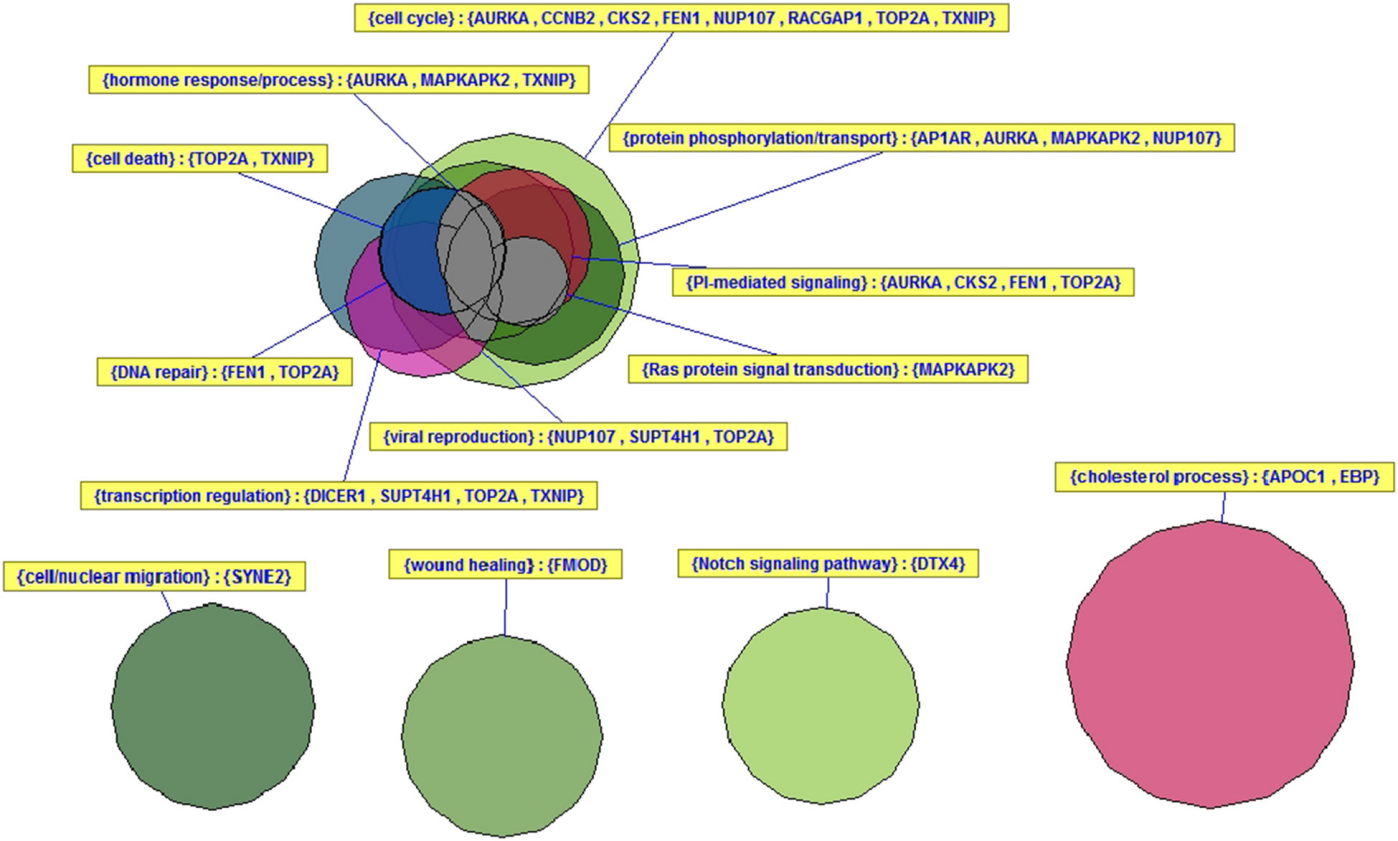
**Gene Ontology categorization of 17-gene model.** A Gene Ontology (GO) categorization was performed using DAVID to identify the associated GO biological processes for the 17-gene model. A VennMaster diagram represents the approximate overlap between GO terms. To simplify, redundant terms were grouped together. Genes in the 17-gene list are involved in a wide range of biological processes known to be involved in breast cancer biology including cell cycle, hormone response, cell death, DNA repair, transcription regulation, wound healing, and others. Since the eight-gene set is entirely contained in the 17-gene set it would be involved in many of the same processes.

Several prognostic signatures in breast cancer identify the same or very similar risk groups of patients [27,28]. While the genes utilized in the RFRS model have only minimal overlap with those identified in other breast cancer outcome signatures (Additional file 1: Figure S6), the prognostic ability of the RFRS model compares well with other assays. The 17-gene and eight-gene optimized sets have only a single gene (AURKA) in common with OncotypeDX [20], a single gene in common with Veridex [10] (FEN1, 17-gene set only), and none with Mammaprint [22]. The similar level of performance between our signature and others together with the lack of overlap in gene members suggests that there are many equivalent solutions to this problem. We provide an optimal subset for our training data but acknowledge that many other effective subsets are also possible and provide a method for identifying such combinations via the predictor alternates derived from k-mean clustering analysis. Gene Ontology categorization was performed using DAVID [29,30] and revealed that genes in the 17-gene list are involved in a wide range of biological processes known to be involved in breast cancer biology including cell cycle, hormone response, cell death, DNA repair, transcription regulation, wound healing, and others (Figure 7). Since the eight-gene set is entirely contained in the 17-gene set it would be involved in many of the same processes. GO categorization of the 17-gene set was provided to characterize this specific set of genes. However, when training our models, we required only that the chosen genes/probes be robustly and reproducibly predictive of relapse. We did not require the signatures to be a comprehensive list of such correlated genes/probes. In fact, we show that there are likely many essentially equivalent subsets of genes that would work equally well as predictors of outcome. For practical reasons we have attempted to choose a relatively small list of genes to facilitate migration to other technologies. With only 17 genes as an input list we would not expect statistically significant enrichment of genes for any category. Therefore, we caution against interpretation of these GO categorizations in that sense.

## Conclusions

While most breast cancers are diagnosed at a resectable and thus potentially curable stage, all carry some risk of relapse. As such, most patients are in theory candidates for adjuvant maneuvers to lessen the risk of relapse. We present a meta-analysis of gene expression data in 858 lymph-node-negative, ER-positive, HER2-negative, chemotherapy-naive breast tumors from published datasets. This dataset supports a method of predicting the likelihood of long-term survival without relapse for such breast cancers. The method involves assaying the expression level of one or more RNA transcripts or their expression products in a breast cancer sample, from a set of eight or 17 genes each with one or more alternate genes. We determine a RFRS and risk group by applying the RFRS algorithm to tumor derived gene expression. Furthermore, by comparing the RFRS value to outcomes in training data we can estimate a likelihood of long-term survival without breast cancer relapse in a patient. In theory, those breast cancer patients with tumors at high risk of relapse could be treated more aggressively whereas those at low risk of relapse could more safely avoid the risks and side effects of systemic chemotherapy. The benefits of this type of approach are expected to be significant and are currently being evaluated in the TailorRX (USA) and MINDACT (Europe) trials [31]. Our hope is that this method, together with a rapid assay platform could provide rapid (<1 h) and useful information to inform clinical decision-making. A test developed from this method could be applied to resected breast tumor tissue (either frozen or fresh), formalin-fixed, paraffin-embedded breast cancer tissue or tissue isolated from a core biopsy or fine needle aspirate. We also provide a list of reference genes for use in normalizing gene expression measurements if necessary.

We tested the hypothesis that a larger dataset with more current clinical data would allow development of a signature with better performance than existing methods. Our signature performed comparably but not significantly better, perhaps suggesting that we have reached the upper limits of accuracy for array-based transcriptional signatures for recurrence in ER+/LN- breast cancer. However, RFRS compares favorably to previously described, clinically available products that predict outcome in breast cancer (for example, OncotypeDX and Mammaprint) in several respects: (1) the signature was built from the largest training dataset available to date; (2) patients with HER2+ tumors were excluded, thus focusing only on patients without an existing clear treatment course; (3) the gene signature predicts relapse with equal success for both patients that went on to receive adjuvant hormonal therapy and those who did not; (4) the gene signature was designed for robustness with (in most cases) several alternate genes available for each primary gene; (5) probe set sequences have been validated by alignment and manual assessment. These features, particularly the latter two, make this signature an especially strong candidate for efficient migration to low-cost platforms for use in a clinical setting. Development of a panel for use in the clinic could take advantage of not only primary genes but also some number of alternate genes to increase the chance of a successful migration. Given the small but significant number of discrepancies observed between clinical and array-based determination of ER status we also recommend inclusion of standard biomarkers such as ER, PR, and HER2 on any design. We provide a list of consistently expressed genes, specific to breast tumor tissue, for use as control genes for those platforms that require them. Finally, we make available a large, carefully curated gene expression dataset for ER+, LN- breast cancer along with clinical annotations for use in the research community.

## Abbreviations

AUC: Area under curve; ER: Estrogen receptor; GEO: Gene Expression Omnibus; GO: Gene ontology; HER2: Human epidermal growth factor receptor 2; LN: Lymph node; OOB: Out of bag; RFRS: Random forest relapse score; ROC: Receiver-operating characteristic.

## Competing interests

Cepheid Inc. has licensed from OHSU technology of which OLG, FP, OME, PTS, and JWG are inventors. The technology is used in this research. This potential conflict of interest has been reviewed and managed by OHSU. The remaining authors declare that they have no competing interests.

## Authors’ contributions

OLG participated in the design of the study, assembled the data, developed analysis methods, performed the analysis, and drafted the manuscript. FP, OME, and LMH participated in the design of the study and helped with analysis. EAC participated in the design of the study and helped to draft the manuscript. PTS and JWG conceived of the study, and participated in its design and coordination and helped to draft the manuscript. All authors read and approved the final manuscript.

## Authors’ information

OLG and FP were postdoctoral fellows and LMH was a scientist at Lawrence Berkeley National Laboratory (LBNL), all under the supervision of PTS and JWG, during the production of this work. OLG is currently a genome fellow at the Genome Institute and research assistant professor at Washington University Medical School. OME is a student at University of California, Berkeley. LMH is currently a research assistant professor at Oregon Health & Science University (OHSU). EAC is a medical oncologist and an assistant professor at University of California, San Francisco School of Medicine. PTS was a staff scientist at LBNL and is currently an associate professor at OHSU. JWG was Life Sciences Division director and associate laboratory director for Biosciences at LBNL, program leader of Breast Oncology and Cancer Genetics at UCSF Helen Diller Family Comprehensive Cancer Center and a professor at UCSF. JWG is currently co-director of the Bay Area Breast Cancer Specialized Program of Research Excellence (SPORE), visiting faculty at LBNL, emeritus professor at UCSF, director of the OHSU Center for Spatial Systems Biomedicine (OCSSB), Gordon Moore endowed chair and the department of Biomedical Engineering chair at OHSU.

## Supplementary Material

Additional file 1 Contains all supplementary figures, tables, and a more detailed explanation of all additional files. Click here for file

Additional file 2 Contains clinical annotations for the 572 patients in the training dataset. Click here for file

Additional file 3 Contains clinical annotations for the 286 patients in the test dataset. Click here for file

Additional file 4 Contains GCRMA normalized mRNA expression values for the 572 patients in the training dataset. Click here for file

Additional file 5 Contains GCRMA normalized mRNA expression values for the 286 patients in the test dataset. Click here for file

Additional file 6 Describes alignment of the top 100 probe sets to the reference genome. Click here for file

Additional file 7 Contains actual probe sequences for the top 100 probe sets. Click here for file

Additional file 8 Describes alignment of all reference probe sets to the reference genome. Click here for file

Additional file 9 Contains actual probe sequences for all reference probe sets. Click here for file

Additional file 10 Provides sample patient data as an example of input needed for RFRS sample code and provides sample R code for running the RFRS algorithm. Click here for file

Additional file 11 Consists of the R data files for 17-gene and eight-gene models. Click here for file

Additional file 12 Consists of additional R data files which allow plotting of patients’ RFRS result and determination of predicted relapse probability for the report file. Click here for file

Additional file 13 Shows a sample patient report produced by the algorithm. Click here for file

Additional file 14 Consists of a complete list of GEO sample identifiers for training and test datasets combined. Click here for file

Additional file 15 Consists of additional sample R code to help reproduce the analysis. Click here for file
