# Supplementary material for: A robust prognostic signature for hormone-positive node-negative breast cancer
Source: Genome Med. 2013 Oct 11;5(10):92. doi: 10.1186/gm496 (PMC3961800; doi:10.1186/gm496)
Supplement: Additional file 1 — Contains all supplementary figures, tables, and a more detailed explanation of all additional files. [file gm496-S1.docx]

**Supplementary Materials**

**Supplementary Figure 1. ER (ESR1) and HER2 (ERBB2) status for 998 samples**Expression status was determined using the "205225_at" probe set for ER and the rank sum of the 216835_s_at (ERBB2), 210761_s_at (GRB7), 202991_at (STARD3) and 55616_at (PGAP3) probe sets for HER2. Threshold values were chosen by mixed model clustering. A total of 68 samples were determined to be ER-negative and 89 samples were determined to be HER2-positive. In total, 140 samples were either HER2-positive or ER-negative (17 were both) and were filtered from further analysis.

**
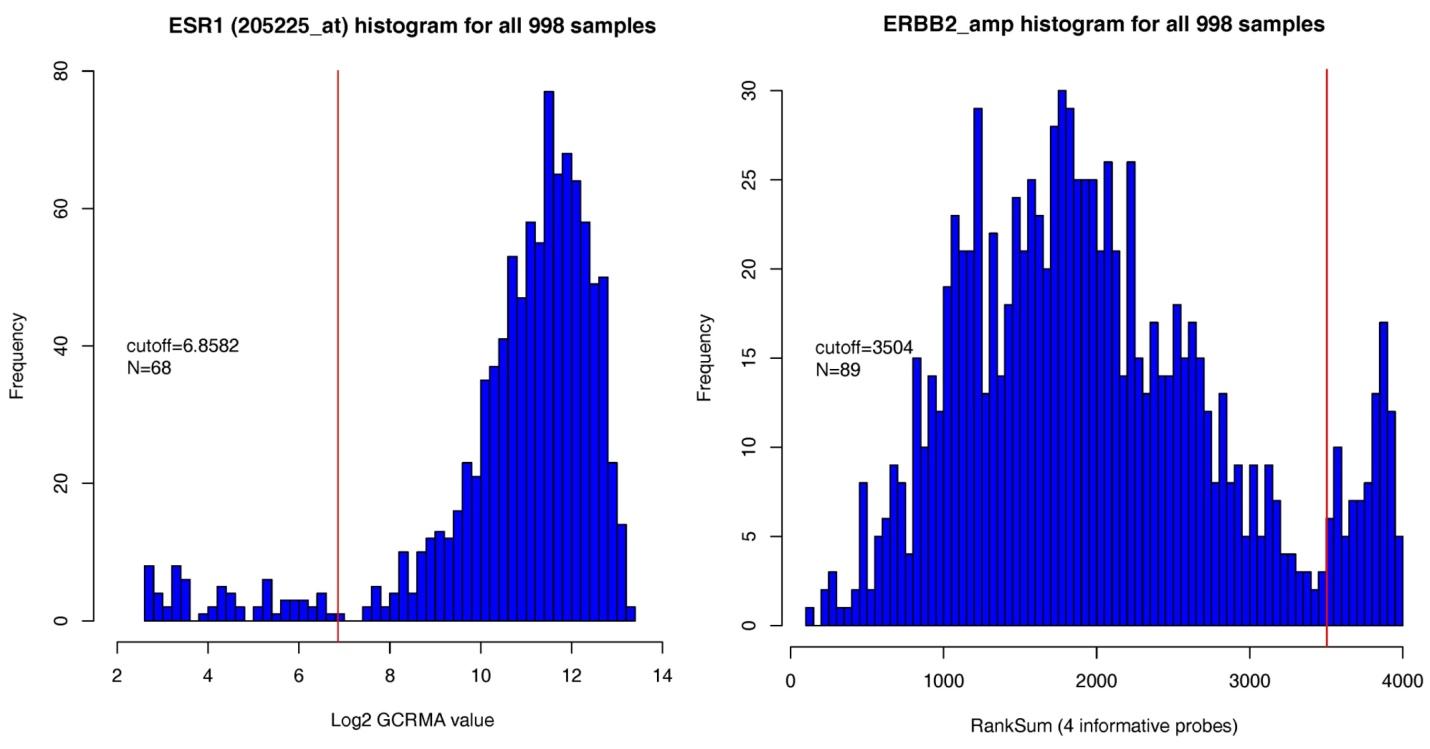
**

**Supplementary Figure 2. Heatmap showing top 90 probe sets after k-means clustering (k=8)**Training data (N = 325) were clustered by expression value using k-means clustering (k = 8) for the top 90 probe sets identified by random forest classification variable importance (10 genes were excluded because of probe set sequence ambiguity or status as a hypothetical protein). The first color side bar on the left indicates cluster number and the second indicates relative variable importance within the cluster (darker blue = greater importance). The top side bars indicate risk group (low, intermediate and high from left to right) and relapse status (red = relapse; yellow = no relapse). Genes (probe sets) are indicated on the right axis. Genes highlighted in yellow represent the primary genes in the model (best in each cluster). Genes not highlighted represent alternates to primary genes in each cluster. **
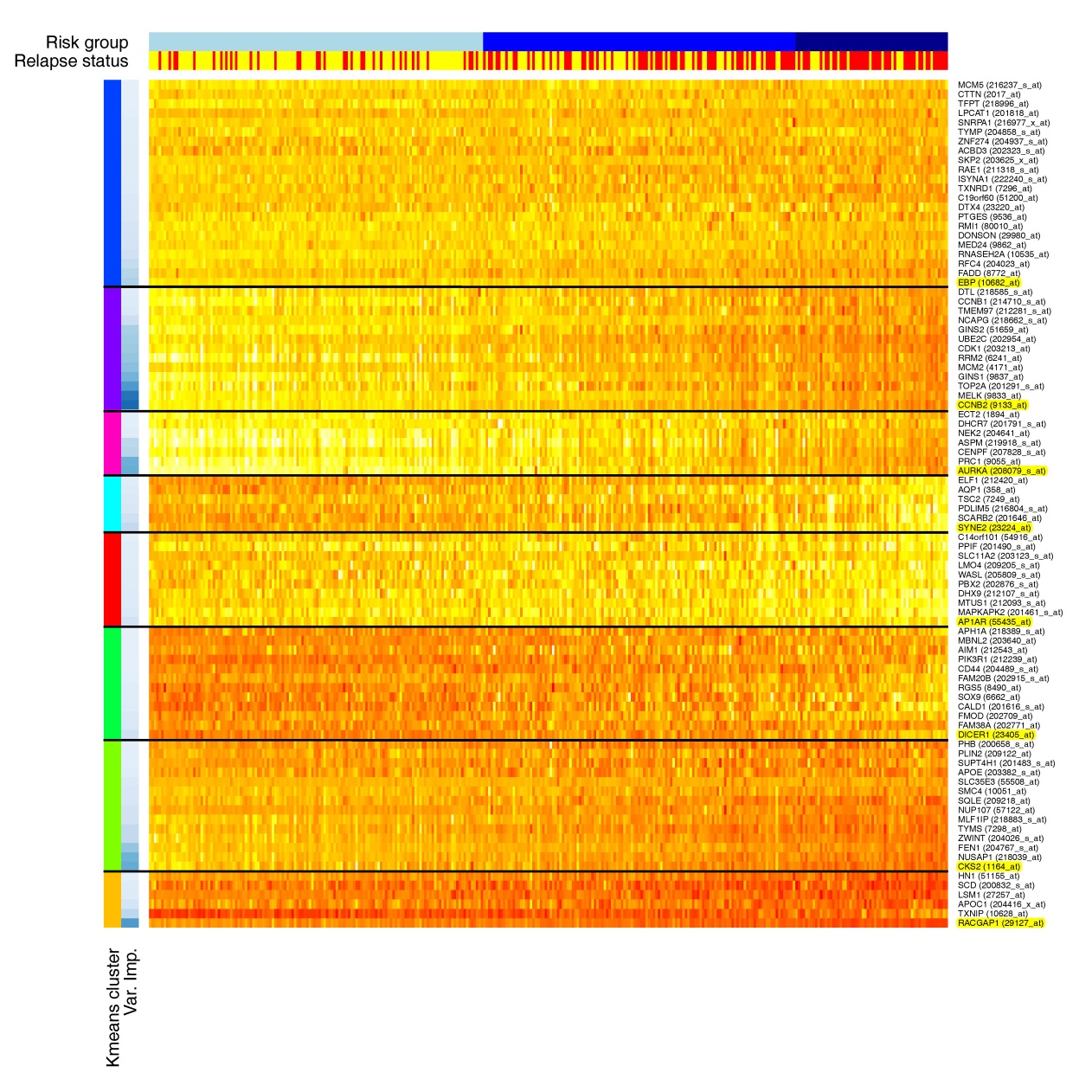
**

**Supplementary Figure 3. Sample patient report**Using the RFRS algorithm, a patient would be assigned an RFRS value. If RFRS is greater than or equal to 0.606 the patient is assigned to the “high-risk” group, if greater than or equal to 0.333 and less than 0.606 the patient is assigned to “intermediate-risk” group and if less than 0.333 the patient is assigned to “low-risk” group. The patient’s RFRS value is also used to determine a likelihood of relapse by comparison to a pre-calculated loess fit of RFRS versus likelihood of relapse for the training dataset. The patient’s estimated likelihood of relapse is determined, added to the summary plot, and output as a new report.

**
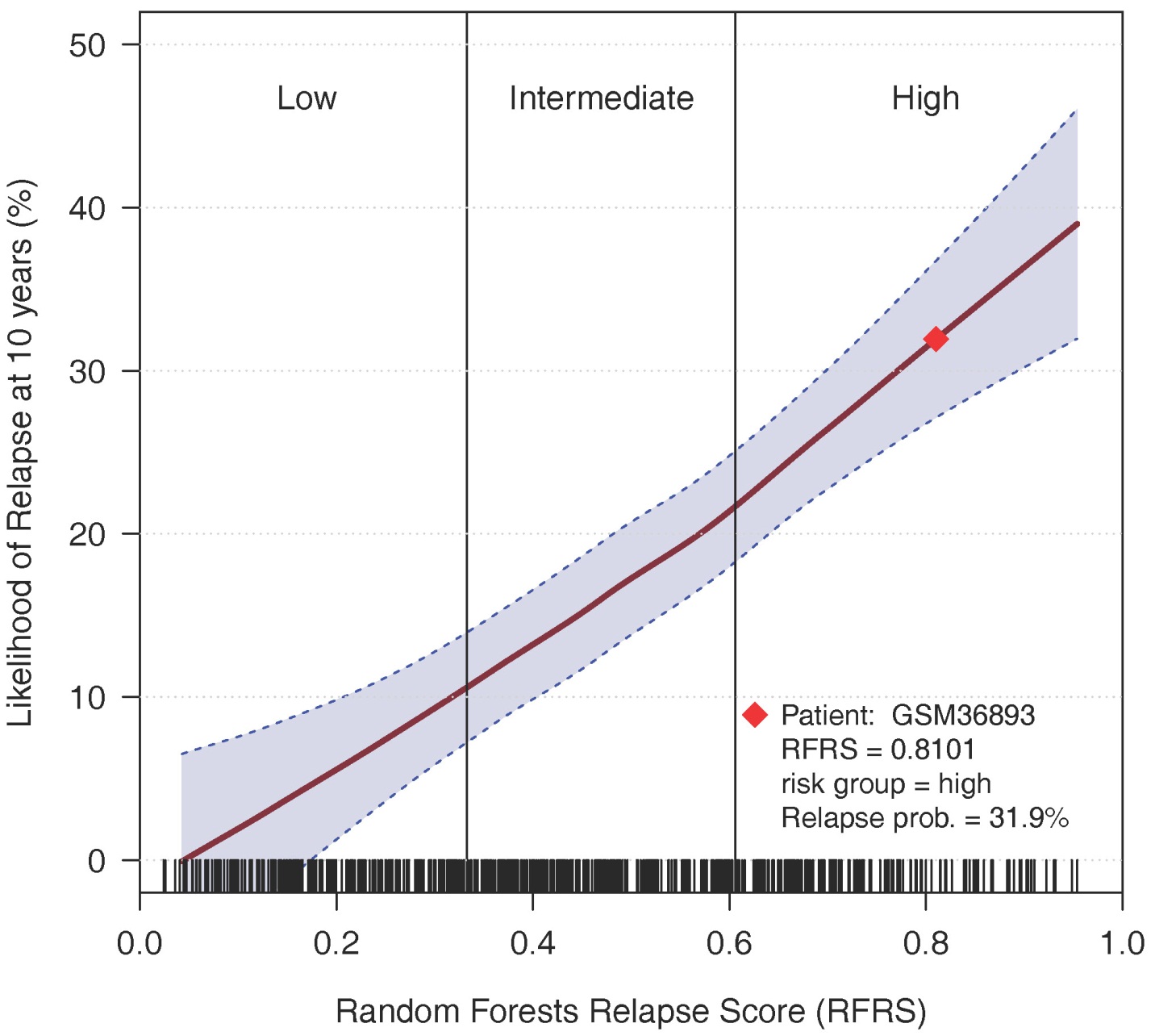
**

**Supplementary Figure 4. Likelihood of relapse according to RFRS group with breakdown into additional risk groups**Kaplan-Meier survival analysis shows a significant difference in relapse free survival for very-low, low, intermediate, high, and very-high-risk groups as defined by the full-gene-set model on training data (N = 572, P = 1.58E-11). Note, the risk scores and corresponding groups for samples used in classifier training (N=325) were assigned from internal OOB cross-validation. Only those patients not used in initial training (without 10-year follow-up) were assigned a risk score and group by de novo classification. Significance between risk groups was determined by Kaplan-Meier logrank test (with test for linear trend).


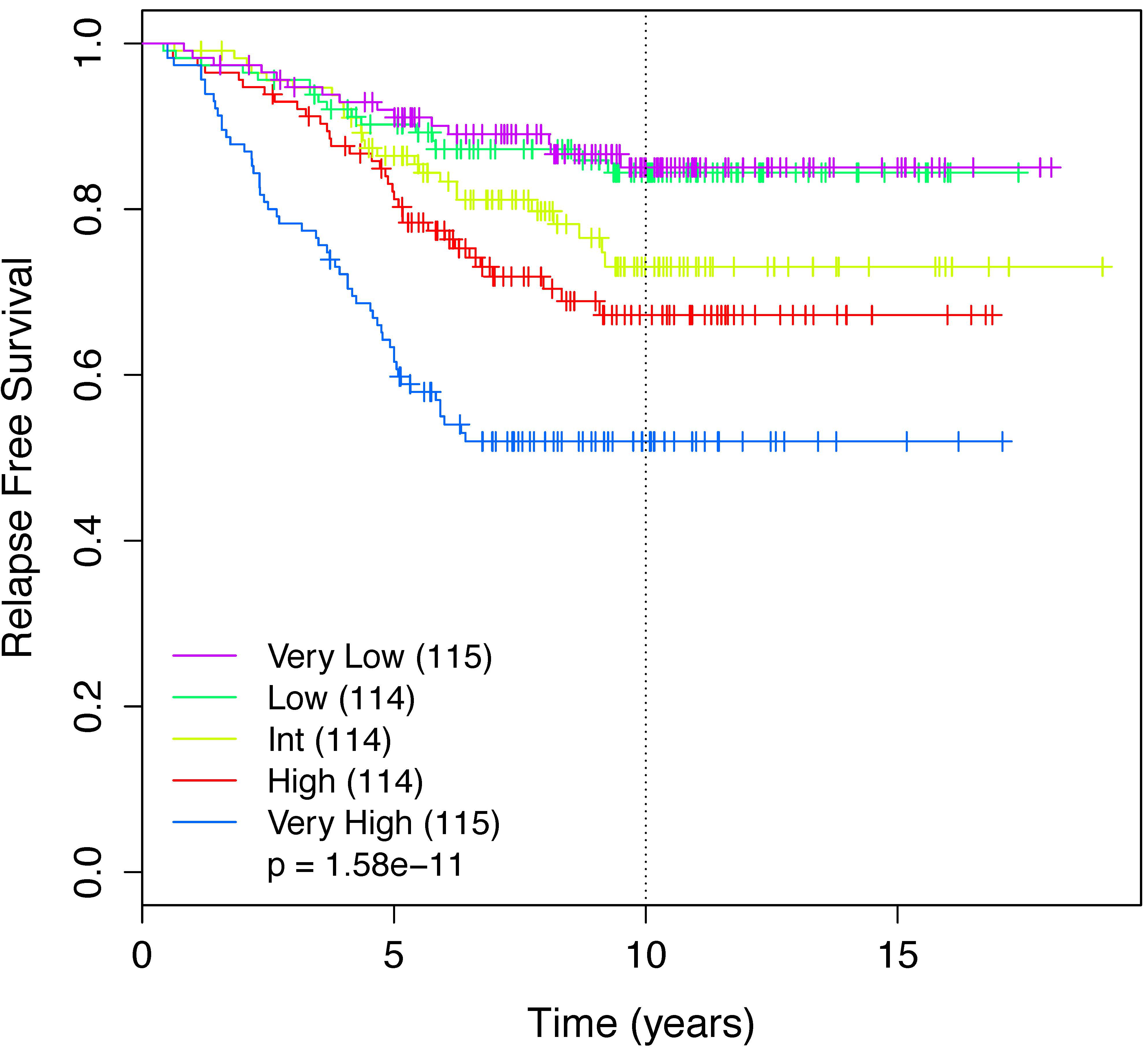


**Supplementary Figure 5. Likelihood of relapse according to RFRS group stratified by treatment status**Kaplan-Meier survival analysis shows a significant difference in relapse free survival for low, intermediate, and high risk groups as defined by the 17-gene-set model on test data stratified into (A) hormone-therapy-treated (N = 41, P = 0.004) and (B) untreated (N = 121, P = 3.76E-07). Significance between risk groups was determined by Kaplan-Meier logrank test (with test for linear trend).

**5A**

**
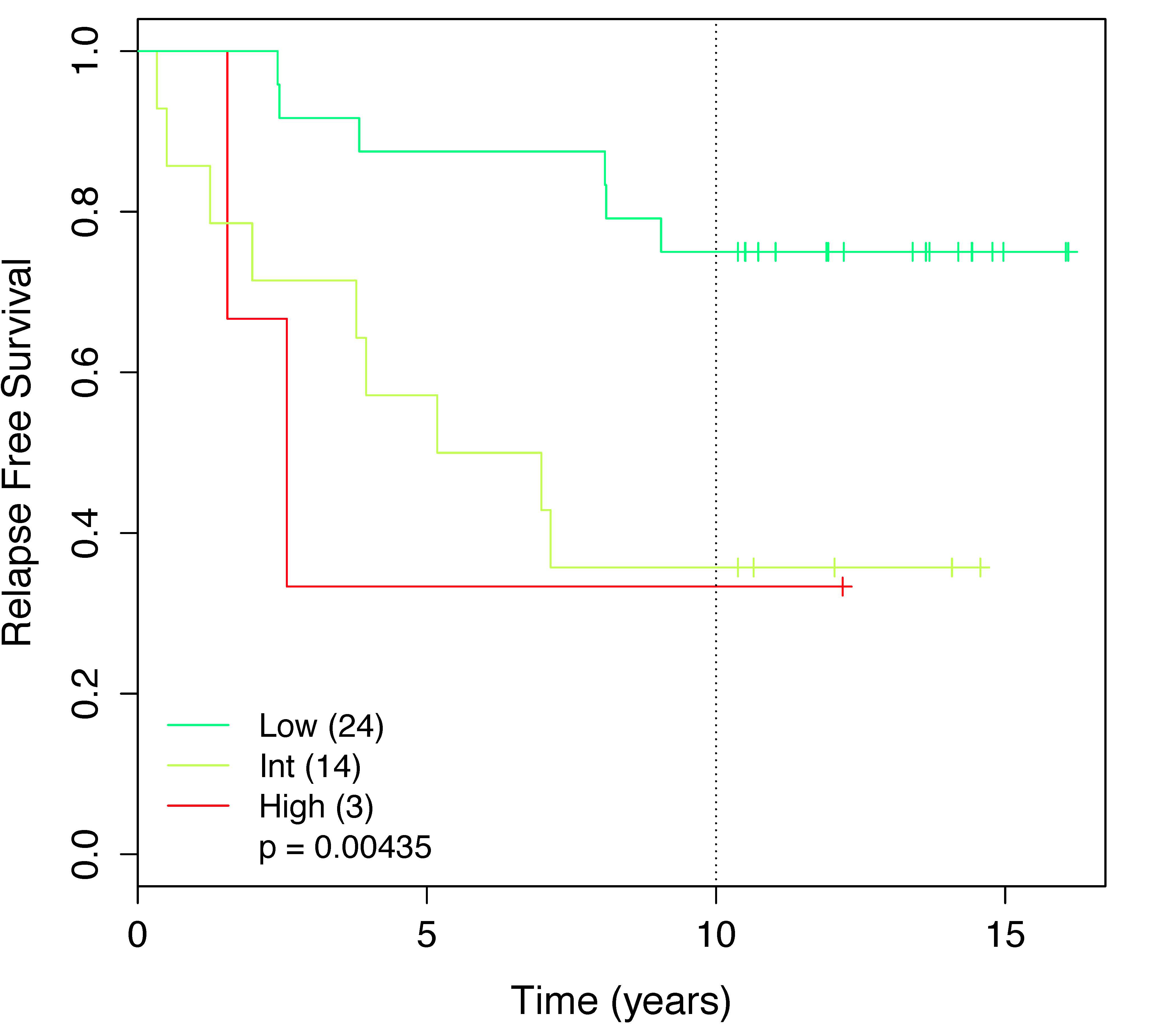
**

**5B**

**
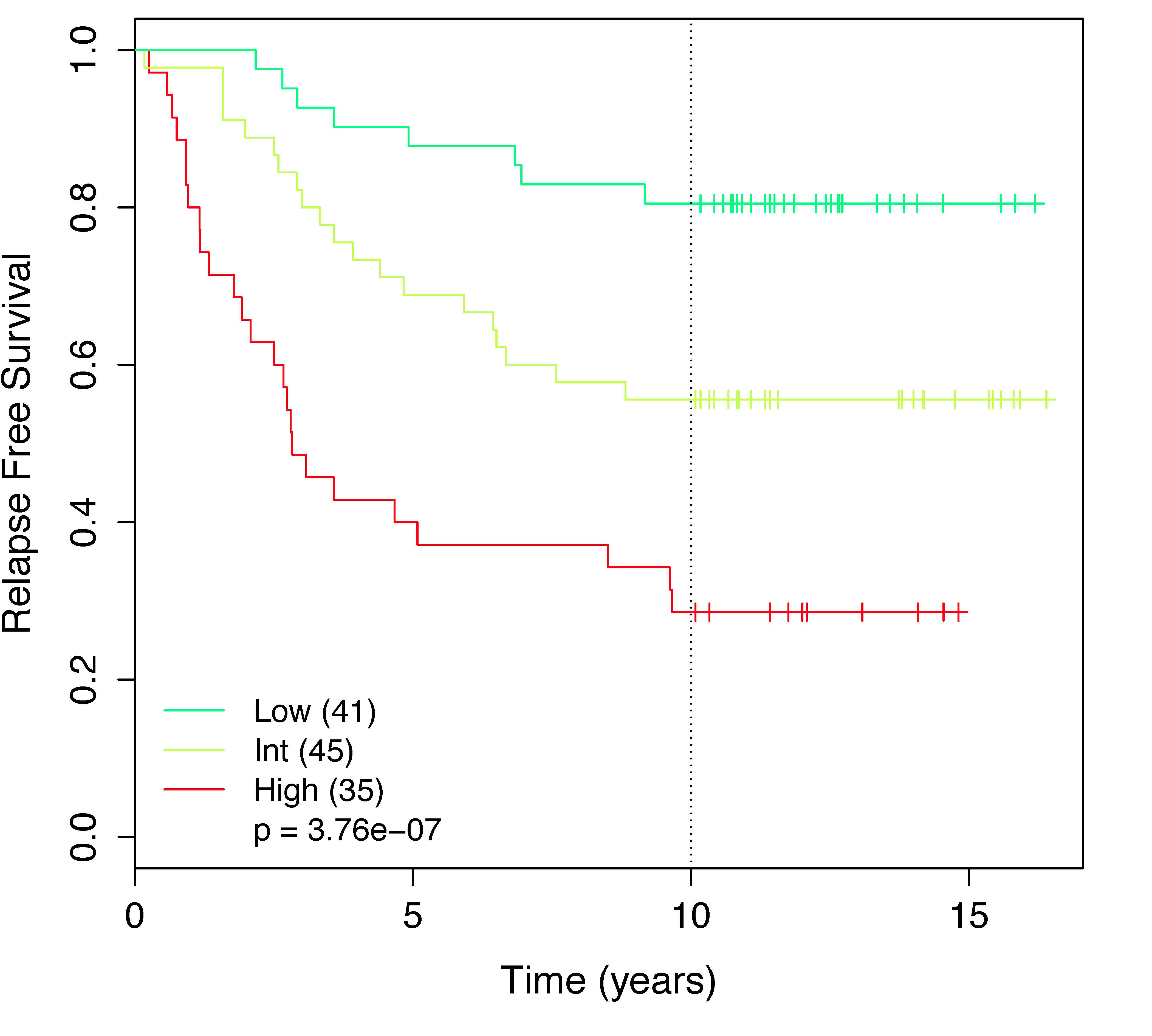
**

**Supplementary Figure 6. Venn diagram showing overlap between RFRS, OncotypeDX and Mammaprint**The top 100 genes identified for use in the RFRS method (all primary and alternate genes) showed minimal overlap with other existing prognostic marker sets. Specifically, two genes overlapped with OncotypeDX (AURKA, CCNB1), four with Veridex (CD44, FEN1, MLF1IP, SMC4), and six with Mammaprint (DTL, ECT2, MELK, NUSAP1, PRC1, RFC4). The 17-gene and 8-gene optimized sets have only a single gene (AURKA) in common with OncotypeDX, a single gene in common with Veridex (FEN1, 17-gene set only) and none with Mammaprint.


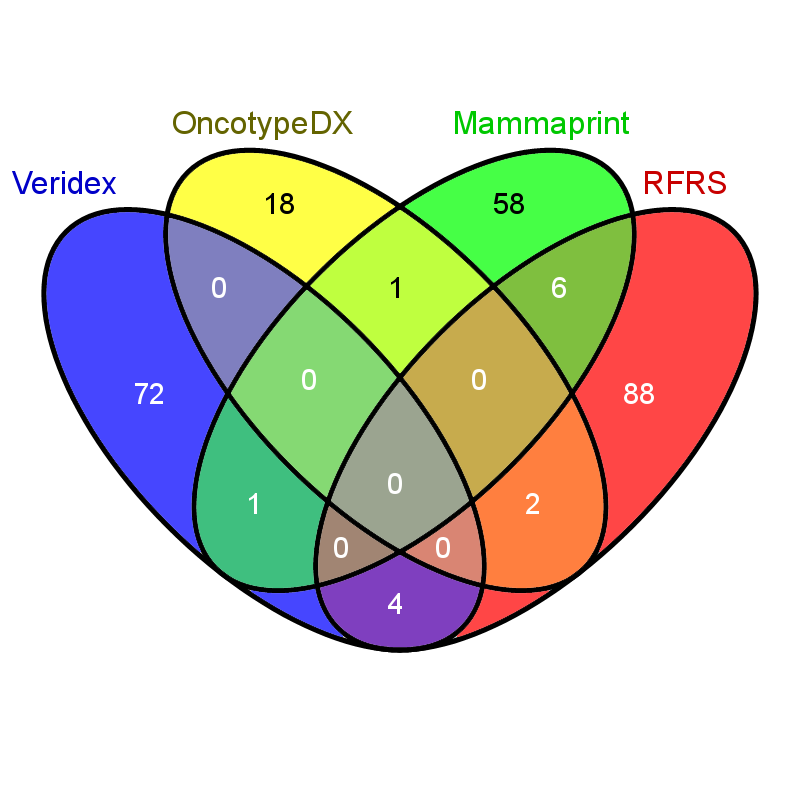


**Supplementary Table 1 - 100 probe sets including all primary, alternate, and excluded genes (k = 20 clusters)**

| **Gene (probe set)** | **EntrezID** | **CDF** | **VarImp** | **Predictor group** | **Predictor status** |
| --- | --- | --- | --- | --- | --- |
| CCNB2 (9133_at) | 9133 | custom | 0.785 | primary | predictor1 |
| MELK (9833_at) | 9833 | custom | 0.739 | alternate1 | predictor1 alternate1 |
| GINS1 (9837_at) | 9837 | custom | 0.476 | alternate2 | predictor1 alternate2 |
| RRM2 (6241_at) | 6241 | custom | 0.399 | alternate3 | predictor1 alternate3 |
| GINS2 (51659_at) | 51659 | custom | 0.354 | alternate4 | predictor1 alternate4 |
| CCNB1 (214710_s_at) | 891 | standard | 0.140 | alternate5 | predictor1 alternate5 |
| TOP2A (201291_s_at) | 7153 | standard | 0.590 | primary | predictor2 |
| MCM2 (4171_at) | 4171 | custom | 0.428 | alternate1 | predictor2 alternate1 |
| KIAA0101 (9768_at) | 9768 | custom | 0.409 | alternate2 | predictor2 alternate2 (excluded) |
| CDK1 (203213_at) | 983 | standard | 0.379 | alternate3 | predictor2 alternate3 |
| UBE2C (202954_at) | 11065 | standard | 0.365 | alternate4 | predictor2 alternate4 |
| TMEM97 (212281_s_at) | 27346 | standard | 0.147 | alternate5 | predictor2 alternate5 |
| DTL (218585_s_at) | 51514 | standard | 0.130 | alternate6 | predictor2 alternate6 |
| RACGAP1 (29127_at) | 29127 | custom | 0.588 | primary | predictor3 |
| LSM1 (27257_at) | 27257 | custom | 0.139 | alternate1 | predictor3 alternate1 |
| SCD (200832_s_at) | 6319 | standard | 0.125 | alternate2 | predictor3 alternate2 |
| HN1 (51155_at) | 51155 | custom | 0.104 | alternate3 | predictor3 alternate3 |
| CKS2 (1164_at) | 1164 | custom | 0.515 | primary | predictor4 |
| NUSAP1 (218039_at) | 51203 | standard | 0.491 | alternate1 | predictor4 alternate1 |
| PTTG1 (203554_x_at) | 9232 | standard | 0.408 | alternate2 | predictor4 alternate2 (excluded) |
| ZWINT (204026_s_at) | 11130 | standard | 0.272 | alternate3 | predictor4 alternate3 |
| TYMS (7298_at) | 7298 | custom | 0.269 | alternate4 | predictor4 alternate4 |
| MLF1IP (218883_s_at) | 79682 | standard | 0.204 | alternate5 | predictor4 alternate5 |
| SQLE (209218_at) | 6713 | standard | 0.174 | alternate6 | predictor4 alternate6 |
| AURKA (208079_s_at) | 6790 | standard | 0.508 | primary | predictor5 |
| PRC1 (9055_at) | 9055 | custom | 0.499 | alternate1 | predictor5 alternate1 |
| CENPF (207828_s_at) | 1063 | standard | 0.306 | alternate2 | predictor5 alternate2 |
| ASPM (219918_s_at) | 259266 | standard | 0.293 | alternate3 | predictor5 alternate3 |
| NEK2 (204641_at) | 4751 | standard | 0.134 | alternate4 | predictor5 alternate4 |
| ECT2 (1894_at) | 1894 | custom | 0.105 | alternate5 | predictor5 alternate5 |
| FEN1 (204767_s_at) | 2237 | standard | 0.403 | primary | predictor6 |
| FADD (8772_at) | 8772 | custom | 0.313 | alternate1 | predictor6 alternate1 |
| SMC4 (10051_at) | 10051 | custom | 0.170 | alternate2 | predictor6 alternate2 |
| SLC35E3 (55508_at) | 55508 | custom | 0.151 | alternate3 | predictor6 alternate3 |
| TXNRD1 (7296_at) | 7296 | custom | 0.136 | alternate4 | predictor6 alternate4 |
| RAE1 (211318_s_at) | 8480 | standard | 0.132 | alternate5 | predictor6 alternate5 |
| ACBD3 (202323_s_at) | 64746 | standard | 0.129 | alternate6 | predictor6 alternate6 |
| ZNF274 (204937_s_at) | 10782 | standard | 0.122 | alternate7 | predictor6 alternate7 |
| FRG1 (2483_at) | 2483 | custom | 0.108 | alternate8 | predictor6 alternate8 (excluded) |
| LPCAT1 (201818_at) | 79888 | standard | 0.106 | alternate9 | predictor6 alternate9 |
| EBP (10682_at) | 10682 | custom | 0.341 | primary | predictor7 |
| RFC4 (204023_at) | 5984 | standard | 0.264 | alternate1 | predictor7 alternate1 |
| NCAPG (218662_s_at) | 64151 | standard | 0.234 | alternate2 | predictor7 alternate2 |
| RNASEH2A (10535_at) | 10535 | custom | 0.205 | alternate3 | predictor7 alternate3 |
| MED24 (9862_at) | 9862 | custom | 0.191 | alternate4 | predictor7 alternate4 |
| DONSON (29980_at) | 29980 | custom | 0.186 | alternate5 | predictor7 alternate5 |
| RMI1 (80010_at) | 80010 | custom | 0.184 | alternate6 | predictor7 alternate6 |
| PTGES (9536_at) | 9536 | custom | 0.164 | alternate7 | predictor7 alternate7 |
| C19orf60 (51200_at) | 55049 | standard | 0.151 | alternate8 | predictor7 alternate8 |
| ISYNA1 (222240_s_at) | 51477 | standard | 0.135 | alternate9 | predictor7 alternate9 |
| SKP2 (203625_x_at) | 6502 | standard | 0.130 | alternate10 | predictor7 alternate10 |
| DPP3 (218567_x_at) | 10072 | standard | 0.126 | alternate11 | predictor7 alternate11 (excluded) |
| TYMP (204858_s_at) | 1890 | standard | 0.122 | alternate12 | predictor7 alternate12 |
| SNRPA1 (216977_x_at) | 6627 | standard | 0.116 | alternate13 | predictor7 alternate13 |
| DHCR7 (201791_s_at) | 1717 | standard | 0.113 | alternate14 | predictor7 alternate14 |
| TFPT (218996_at) | 29844 | standard | 0.105 | alternate15 | predictor7 alternate15 |
| CTTN (2017_at) | 2017 | custom | 0.102 | alternate16 | predictor7 alternate16 |
| MCM5 (216237_s_at) | 4174 | standard | 0.102 | alternate17 | predictor7 alternate17 |
| TXNIP (10628_at) | 10628 | custom | 0.292 | primary | predictor8 |
| SYNE2 (23224_at) | 23224 | custom | 0.270 | primary | predictor9 |
| SCARB2 (201646_at) | 950 | standard | 0.225 | alternate1 | predictor9 alternate1 |
| PDLIM5 (216804_s_at) | 10611 | standard | 0.167 | alternate2 | predictor9 alternate2 |
| TSC2 (7249_at) | 7249 | custom | 0.145 | alternate3 | predictor9 alternate3 |
| ELF1 (212420_at) | 1997 | standard | 0.119 | alternate4 | predictor9 alternate4 |
| DICER1 (23405_at) | 23405 | custom | 0.209 | primary | predictor10 |
| CALD1 (201616_s_at) | 800 | standard | 0.129 | alternate1 | predictor10 alternate1 |
| SOX9 (6662_at) | 6662 | custom | 0.125 | alternate2 | predictor10 alternate2 |
| FAM20B (202915_s_at) | 9917 | standard | 0.108 | alternate3 | predictor10 alternate3 |
| APH1A (218389_s_at) | 51107 | standard | 0.099 | alternate4 | predictor10 alternate4 |
| AP1AR (55435_at) | 55435 | custom | 0.201 | primary | predictor11 |
| PDCD6 (222380_s_at) | 10016 | standard | 0.154 | alternate1 | predictor11 alternate1 (excluded) |
| PBX2 (202876_s_at) | 5089 | standard | 0.134 | alternate2 | predictor11 alternate2 |
| WASL (205809_s_at) | 8976 | standard | 0.126 | alternate3 | predictor11 alternate3 |
| SLC11A2 (203123_s_at) | 4891 | standard | 0.119 | alternate4 | predictor11 alternate4 |
| KIAA0776 (212634_at) | 23376 | standard | 0.107 | alternate5 | predictor11 alternate5 (excluded) |
| C14orf101 (54916_at) | 54916 | custom | 0.101 | alternate6 | predictor11 alternate6 |
| NUP107 (57122_at) | 57122 | custom | 0.197 | primary | predictor12 |
| FAM38A (202771_at) | 9780 | standard | 0.165 | alternate1 | predictor12 alternate1 |
| PLIN2 (209122_at) | 123 | standard | 0.110 | alternate2 | predictor12 alternate2 |
| AIM1 (212543_at) | 202 | standard | 0.102 | alternate3 | predictor12 alternate3 |
| APOC1 (204416_x_at) | 341 | standard | 0.176 | primary | predictor13 |
| APOE (203382_s_at) | 348 | standard | 0.121 | alternate1 | predictor13 alternate1 |
| DTX4 (23220_at) | 23220 | custom | 0.164 | primary | predictor14 |
| AQP1 (358_at) | 358 | custom | 0.141 | alternate1 | predictor14 alternate1 |
| LMO4 (209205_s_at) | 8543 | standard | 0.120 | alternate2 | predictor14 alternate2 |
| TAF1D (218750_at) | 79101 | standard | 0.159 | primary | predictor15 (excluded) |
| SNORA25 (684959_at) | 684959 | custom | 0.127 | alternate1 | predictor15 alternate1 (excluded) |
| FMOD (202709_at) | 2331 | standard | 0.154 | primary | predictor16 |
| RGS5 (8490_at) | 8490 | custom | 0.120 | alternate1 | predictor16 alternate1 |
| PIK3R1 (212239_at) | 5295 | standard | 0.103 | alternate2 | predictor16 alternate2 |
| MBNL2 (203640_at) | 10150 | standard | 0.100 | alternate3 | predictor16 alternate3 |
| MAPKAPK2 (201461_s_at) | 9261 | standard | 0.151 | primary | predictor17 |
| MTUS1 (212093_s_at) | 57509 | standard | 0.136 | alternate1 | predictor17 alternate1 |
| DHX9 (212107_s_at) | 1660 | standard | 0.136 | alternate2 | predictor17 alternate2 |
| PPIF (201490_s_at) | 10105 | standard | 0.115 | alternate3 | predictor17 alternate3 |
| FOLR1 (211074_at) | 2348 | standard | 0.126 | primary | predictor18 (excluded) |
| KIAA1467 (57613_at) | 57613 | custom | 0.116 | primary | predictor19 (excluded) |
| SUPT4H1 (201483_s_at) | 6827 | standard | 0.111 | primary | predictor20 |
| PHB (200658_s_at) | 5245 | standard | 0.106 | alternate1 | predictor20 alternate1 |
| CD44 (204489_s_at) | 960 | standard | 0.105 | alternate2 | predictor20 alternate2 |

Notes: Excluded genes highlighted in red.

**Supplementary Table 2 - 90 probe sets (failed probe sets excluded) including all primary and alternate genes (k = 8 clusters)**

| **Gene (probe set)** | **CDF** | **VarImp** | **predictor group** | **predictor status** |
| --- | --- | --- | --- | --- |
| CCNB2 (9133_at) | custom | 0.785 | primary | predictor1 |
| MELK (9833_at) | custom | 0.739 | alternate1 | predictor1 alternate1 |
| TOP2A (201291_s_at) | standard | 0.590 | alternate2 | predictor1 alternate2 |
| GINS1 (9837_at) | custom | 0.476 | alternate3 | predictor1 alternate3 |
| MCM2 (4171_at) | custom | 0.428 | alternate4 | predictor1 alternate4 |
| RRM2 (6241_at) | custom | 0.399 | alternate5 | predictor1 alternate5 |
| CDK1 (203213_at) | standard | 0.379 | alternate6 | predictor1 alternate6 |
| UBE2C (202954_at) | standard | 0.365 | alternate7 | predictor1 alternate7 |
| GINS2 (51659_at) | custom | 0.354 | alternate8 | predictor1 alternate8 |
| NCAPG (218662_s_at) | standard | 0.234 | alternate9 | predictor1 alternate9 |
| TMEM97 (212281_s_at) | standard | 0.147 | alternate10 | predictor1 alternate10 |
| CCNB1 (214710_s_at) | standard | 0.140 | alternate11 | predictor1 alternate11 |
| DTL (218585_s_at) | standard | 0.130 | alternate12 | predictor1 alternate12 |
| RACGAP1 (29127_at) | custom | 0.588 | primary | predictor2 |
| TXNIP (10628_at) | custom | 0.292 | alternate1 | predictor2 alternate1 |
| APOC1 (204416_x_at) | standard | 0.176 | alternate2 | predictor2 alternate2 |
| LSM1 (27257_at) | custom | 0.139 | alternate3 | predictor2 alternate3 |
| SCD (200832_s_at) | standard | 0.125 | alternate4 | predictor2 alternate4 |
| HN1 (51155_at) | custom | 0.104 | alternate5 | predictor2 alternate5 |
| CKS2 (1164_at) | custom | 0.515 | primary | predictor3 |
| NUSAP1 (218039_at) | standard | 0.491 | alternate1 | predictor3 alternate1 |
| FEN1 (204767_s_at) | standard | 0.403 | alternate2 | predictor3 alternate2 |
| ZWINT (204026_s_at) | standard | 0.272 | alternate3 | predictor3 alternate3 |
| TYMS (7298_at) | custom | 0.269 | alternate4 | predictor3 alternate4 |
| MLF1IP (218883_s_at) | standard | 0.204 | alternate5 | predictor3 alternate5 |
| NUP107 (57122_at) | custom | 0.197 | alternate6 | predictor3 alternate6 |
| SQLE (209218_at) | standard | 0.174 | alternate7 | predictor3 alternate7 |
| SMC4 (10051_at) | custom | 0.170 | alternate8 | predictor3 alternate8 |
| SLC35E3 (55508_at) | custom | 0.151 | alternate9 | predictor3 alternate9 |
| APOE (203382_s_at) | standard | 0.121 | alternate10 | predictor3 alternate10 |
| SUPT4H1 (201483_s_at) | standard | 0.111 | alternate11 | predictor3 alternate11 |
| PLIN2 (209122_at) | standard | 0.110 | alternate12 | predictor3 alternate12 |
| PHB (200658_s_at) | standard | 0.106 | alternate13 | predictor3 alternate13 |
| AURKA (208079_s_at) | standard | 0.508 | primary | predictor4 |
| PRC1 (9055_at) | custom | 0.499 | alternate1 | predictor4 alternate1 |
| CENPF (207828_s_at) | standard | 0.306 | alternate2 | predictor4 alternate2 |
| ASPM (219918_s_at) | standard | 0.293 | alternate3 | predictor4 alternate3 |
| NEK2 (204641_at) | standard | 0.134 | alternate4 | predictor4 alternate4 |
| DHCR7 (201791_s_at) | standard | 0.113 | alternate5 | predictor4 alternate5 |
| ECT2 (1894_at) | custom | 0.105 | alternate6 | predictor4 alternate6 |
| EBP (10682_at) | custom | 0.341 | primary | predictor5 |
| FADD (8772_at) | custom | 0.313 | alternate1 | predictor5 alternate1 |
| RFC4 (204023_at) | standard | 0.264 | alternate2 | predictor5 alternate2 |
| RNASEH2A (10535_at) | custom | 0.205 | alternate3 | predictor5 alternate3 |
| MED24 (9862_at) | custom | 0.191 | alternate4 | predictor5 alternate4 |
| DONSON (29980_at) | custom | 0.186 | alternate5 | predictor5 alternate5 |
| RMI1 (80010_at) | custom | 0.184 | alternate6 | predictor5 alternate6 |
| PTGES (9536_at) | custom | 0.164 | alternate7 | predictor5 alternate7 |
| DTX4 (23220_at) | custom | 0.164 | alternate8 | predictor5 alternate8 |
| C19orf60 (51200_at) | standard | 0.151 | alternate9 | predictor5 alternate9 |
| TXNRD1 (7296_at) | custom | 0.136 | alternate10 | predictor5 alternate10 |
| ISYNA1 (222240_s_at) | standard | 0.135 | alternate11 | predictor5 alternate11 |
| RAE1 (211318_s_at) | standard | 0.132 | alternate12 | predictor5 alternate12 |
| SKP2 (203625_x_at) | standard | 0.130 | alternate13 | predictor5 alternate13 |
| ACBD3 (202323_s_at) | standard | 0.129 | alternate14 | predictor5 alternate14 |
| ZNF274 (204937_s_at) | standard | 0.122 | alternate15 | predictor5 alternate15 |
| TYMP (204858_s_at) | standard | 0.122 | alternate16 | predictor5 alternate16 |
| SNRPA1 (216977_x_at) | standard | 0.116 | alternate17 | predictor5 alternate17 |
| LPCAT1 (201818_at) | standard | 0.106 | alternate18 | predictor5 alternate18 |
| TFPT (218996_at) | standard | 0.105 | alternate19 | predictor5 alternate19 |
| CTTN (2017_at) | custom | 0.102 | alternate20 | predictor5 alternate20 |
| MCM5 (216237_s_at) | standard | 0.102 | alternate21 | predictor5 alternate21 |
| SYNE2 (23224_at) | custom | 0.270 | primary | predictor6 |
| SCARB2 (201646_at) | standard | 0.225 | alternate1 | predictor6 alternate1 |
| PDLIM5 (216804_s_at) | standard | 0.167 | alternate2 | predictor6 alternate2 |
| TSC2 (7249_at) | custom | 0.145 | alternate3 | predictor6 alternate3 |
| AQP1 (358_at) | custom | 0.141 | alternate4 | predictor6 alternate4 |
| ELF1 (212420_at) | standard | 0.119 | alternate5 | predictor6 alternate5 |
| DICER1 (23405_at) | custom | 0.209 | primary | predictor7 |
| FAM38A (202771_at) | standard | 0.165 | alternate1 | predictor7 alternate1 |
| FMOD (202709_at) | standard | 0.154 | alternate2 | predictor7 alternate2 |
| CALD1 (201616_s_at) | standard | 0.129 | alternate3 | predictor7 alternate3 |
| SOX9 (6662_at) | custom | 0.125 | alternate4 | predictor7 alternate4 |
| RGS5 (8490_at) | custom | 0.120 | alternate5 | predictor7 alternate5 |
| FAM20B (202915_s_at) | standard | 0.108 | alternate6 | predictor7 alternate6 |
| CD44 (204489_s_at) | standard | 0.105 | alternate7 | predictor7 alternate7 |
| PIK3R1 (212239_at) | standard | 0.103 | alternate8 | predictor7 alternate8 |
| AIM1 (212543_at) | standard | 0.102 | alternate9 | predictor7 alternate9 |
| MBNL2 (203640_at) | standard | 0.100 | alternate10 | predictor7 alternate10 |
| APH1A (218389_s_at) | standard | 0.099 | alternate11 | predictor7 alternate11 |
| AP1AR (55435_at) | custom | 0.201 | primary | predictor8 |
| MAPKAPK2 (201461_s_at) | standard | 0.151 | alternate1 | predictor8 alternate1 |
| MTUS1 (212093_s_at) | standard | 0.136 | alternate2 | predictor8 alternate2 |
| DHX9 (212107_s_at) | standard | 0.136 | alternate3 | predictor8 alternate3 |
| PBX2 (202876_s_at) | standard | 0.134 | alternate4 | predictor8 alternate4 |
| WASL (205809_s_at) | standard | 0.126 | alternate5 | predictor8 alternate5 |
| LMO4 (209205_s_at) | standard | 0.120 | alternate6 | predictor8 alternate6 |
| SLC11A2 (203123_s_at) | standard | 0.119 | alternate7 | predictor8 alternate7 |
| PPIF (201490_s_at) | standard | 0.115 | alternate8 | predictor8 alternate8 |
| C14orf101 (54916_at) | custom | 0.101 | alternate9 | predictor8 alternate9 |

**Supplementary Table 3. Top 25 reference genes (set 1) along with reference genes used by OncotypeDX.**

| **Probe set** | **Gene Symbol** | **Mean (exp)** | **S.D.** | **Fraction (exp)** | **COV** | **CDF** |
| --- | --- | --- | --- | --- | --- | --- |
| 211445_x_at | NACAP1 | 2266.5 | 210.9 | 1.00 | 0.093 | standard |
| 216515_x_at | PTMA | 2573.0 | 382.5 | 1.00 | 0.149 | standard |
| 217092_x_at | RPL7 | 1945.7 | 355.3 | 1.00 | 0.183 | standard |
| 103910_at | MYL12B | 1017.5 | 195.8 | 1.00 | 0.192 | custom |
| 208672_s_at | SFRS3 | 1713.0 | 380.0 | 1.00 | 0.222 | standard |
| 200960_x_at | CLTA | 1786.2 | 397.5 | 1.00 | 0.223 | standard |
| 200893_at | TRA2B | 1403.7 | 312.8 | 1.00 | 0.223 | standard |
| 217466_x_at | RPS2 | 1414.4 | 327.5 | 1.00 | 0.232 | standard |
| 23787_at | MTCH1 | 1120.0 | 269.8 | 1.00 | 0.241 | custom |
| 221767_x_at | HDLBP | 1174.4 | 284.9 | 1.00 | 0.243 | standard |
| 23191_at | CYFIP1 | 1345.1 | 329.4 | 1.00 | 0.245 | custom |
| 211069_s_at | SUMO1 | 1111.6 | 276.2 | 1.00 | 0.248 | standard |
| 201385_at | DHX15 | 1529.4 | 383.5 | 1.00 | 0.251 | standard |
| 200014_s_at | HNRNPC | 1517.7 | 385.3 | 1.00 | 0.254 | standard |
| 200667_at | UBE2D3 | 1090.1 | 279.3 | 1.00 | 0.256 | standard |
| 9802_at | DAZAP2 | 1181.2 | 303.6 | 1.00 | 0.257 | custom |
| 200058_s_at | SNRNP200 | 1104.4 | 285.9 | 1.00 | 0.259 | standard |
| 91746_at | YTHDC1 | 965.1 | 250.7 | 1.00 | 0.260 | custom |
| 1315_at | COPB1 | 1118.2 | 291.9 | 1.00 | 0.261 | custom |
| 4714_at | NDUFB8 | 1219.0 | 325.5 | 1.00 | 0.267 | custom |
| 40189_at | SET | 1347.9 | 360.7 | 1.00 | 0.268 | standard |
| 221743_at | CELF1 | 1094.0 | 294.2 | 1.00 | 0.269 | standard |
| 208775_at | XPO1 | 940.7 | 256.1 | 1.00 | 0.272 | standard |
| 211270_x_at | PTBP1 | 973.1 | 266.8 | 1.00 | 0.274 | standard |
| 211185_s_at | SF3B1 | 1077.9 | 297.9 | 1.00 | 0.276 | standard |
| 10109_at | ARPC2 | 1357.4 | 375.9 | 1.00 | 0.277 | custom |
| 51374_at | C2orf28 | 1021.3 | 283.0 | 1.00 | 0.277 | custom |
| 201336_at | VAMP3 | 959.2 | 267.4 | 1.00 | 0.279 | standard |
| 200028_s_at | STARD7 | 1087.9 | 303.4 | 1.00 | 0.279 | standard |
| 22872_at | SEC31A | 1040.4 | 290.5 | 1.00 | 0.279 | custom |
| **OncotypeDX Reference Genes** | | | | | |  |
| 213867_x_at | ACTB | 19566.3 | 4360.8 | 1.00 | 0.223 | standard |
| 200801_x_at | ACTB | 17901.0 | 3995.4 | 1.00 | 0.223 | standard |
| 2597_at | GAPDH | 11873.9 | 3810.3 | 1.00 | 0.321 | standard |
| 212581_x_at | GAPDH | 11930.9 | 4172.5 | 1.00 | 0.350 | standard |
| 217398_x_at | GAPDH | 6595.6 | 2460.2 | 1.00 | 0.373 | standard |
| 213453_x_at | GAPDH | 6695.2 | 2726.8 | 1.00 | 0.407 | standard |
| 60_at | ACTB | 3786.2 | 1622.3 | 1.00 | 0.428 | standard |
| 7037_at | TFRC | 781.8 | 466.6 | 1.00 | 0.597 | standard |
| 208691_at | TFRC | 1035.1 | 630.8 | 1.00 | 0.609 | standard |
| 207332_s_at | TFRC | 506.9 | 341.6 | 0.97 | 0.674 | standard |

Notes: Failed genes highlighted in red. RPLPO and GUS are also listed as reference genes for OncotypeDX, but were not found in U133A data.

**Supplementary Table 4. Top 15 reference genes (set 2)**

| **Range** | **Probe set** | **Gene Symbol** | **Mean (exp)** | **S.D.** | **Fraction (exp)** | **COV** | **CDF** |
| --- | --- | --- | --- | --- | --- | --- | --- |
| Low | 9927_at | MFN2 | 207.0 | 33.1 | 1.00 | 0.160 | custom |
|  | 26100_at | WIPI2 | 216.5 | 40.3 | 1.00 | 0.186 | custom |
|  | 201507_at | PFDN1 | 260.8 | 51.2 | 1.00 | 0.196 | standard |
|  | 7337_at | UBE3A | 225.3 | 46.5 | 0.99 | 0.207 | custom |
|  | 2976_at | GTF3C2 | 226.3 | 47.6 | 1.00 | 0.210 | custom |
| Medium | 10657_at | KHDRBS1 | 776.4 | 166.3 | 1.00 | 0.214 | custom |
|  | 201330_at | RARS | 502.6 | 117.1 | 1.00 | 0.233 | standard |
|  | 201319_at | MYL12A | 574.4 | 135.2 | 1.00 | 0.235 | standard |
|  | 3184_at | HNRNPD | 678.8 | 160.0 | 1.00 | 0.236 | custom |
|  | 200020_at | TARDBP | 521.1 | 124.4 | 1.00 | 0.239 | standard |
|  | 10236_at | HNRNPR | 570.1 | 140.4 | 1.00 | 0.246 | custom |
| High | 200893_at | TRA2B | 1403.7 | 312.8 | 1.00 | 0.223 | standard |
|  | 217466_x_at | RPS2 | 1414.4 | 327.5 | 1.00 | 0.232 | standard |
|  | 221619_s_at | MTCH1* | 1401.9 | 342.6 | 1.00 | 0.244 | standard |
|  | 208923_at | CYFIP1* | 1339.2 | 333.6 | 1.00 | 0.249 | standard |
|  | 201385_at | DHX15 | 1529.4 | 383.5 | 1.00 | 0.251 | standard |
|  | 200014_s_at | HNRNPC | 1517.7 | 385.3 | 1.00 | 0.254 | standard |
|  | 4714_at | NDUFB8 | 1219.0 | 325.5 | 1.00 | 0.267 | custom |

Notes: Failed genes highlighted in red. *MTCH1 and CYFIP1 were identified as potential reference genes in set #1 with custom CDF probesets and in set #2 with standard CDF probe sets. It is recommended to use customCDF probe sets for these.

**Supplementary Table 5. Gene lists for RFRS, Mammaprint, OncotypeDX, and Veridex signatures**

| **Veridex** | **Oncotype** | **Mammaprint** | **RFRS100** | **RFRS17** | **RFRS8** |
| --- | --- | --- | --- | --- | --- |
| ABLIM1 | ACTB | AA404325 | CCNB2 | CCNB2 | CCNB2 |
| ACACB | GAPDH | AA834945 | MELK | TOP2A | RACGAP1 |
| ACOT11 | RPLP0 | AI224578 | GINS1 | RACGAP1 | CKS2 |
| ANKHD1 | GUSB | AI283268 | RRM2 | CKS2 | AURKA |
| ANKHD1-EIF4EBP3 | TFRC | ALDH4A1 | GINS2 | AURKA | EBP |
| AP2A2 | MKI67 | AP2B1 | CCNB1 | FEN1 | SYNE2 |
| ARHGDIB | AURKA | AW014921 | TOP2A | EBP | DICER1 |
| ATAD2 | EPR1 | AYTL2 | MCM2 | TXNIP | AP1AR |
| BCL2L14 | CCNB1 | BBC3 | KIAA0101 | SYNE2 |  |
| BICD1 | MYBL2 | C16orf61 | CDK1 | DICER1 |  |
| C11orf51 | ERBB2 | C20orf46 | UBE2C | AP1AR |  |
| C11orf9 | GRB7 | C9orf30 | TMEM97 | NUP107 |  |
| C3 | FAS | CCNE2 | DTL | APOC1 |  |
| C6orf165 | PGR | CDC42BPA | RACGAP1 | DTX4 |  |
| CAPN2 | BCL2 | CDCA7 | LSM1 | FMOD |  |
| CBX3 | SCUBE2 | CENPA | SCD | MAPKAPK2 |  |
| CCNE2 | MMP11 | COL4A2 | HN1 | SUPT4H1 |  |
| CD44 | CTSL2 | DCK | CKS2 |  |  |
| CEP57 | GSTM1 | DIAPH3 | NUSAP1 |  |  |
| CLN8 | CD68 | DTL | PTTG1 |  |  |
| CNKSR1 | BAG1 | EBF4 | ZWINT |  |  |
| COL2A1 |  | ECT2 | TYMS |  |  |
| DUSP4 |  | EGLN1 | MLF1IP |  |  |
| EEF1A2 |  | ESM1 | SQLE |  |  |
| EIF4EBP3 |  | EXT1 | AURKA |  |  |
| ETV2 |  | FBXO31 | PRC1 |  |  |
| FEN1 |  | FGF18 | CENPF |  |  |
| FKBP2 |  | FLT1 | ASPM |  |  |
| FUT3 |  | GMPS | NEK2 |  |  |
| FUT5 |  | GNAZ | ECT2 |  |  |
| FUT6 |  | GPR126 | FEN1 |  |  |
| GABRQ |  | GPR180 | FADD |  |  |
| GAS2 |  | GSOX2 | SMC4 |  |  |
| GFOD2 |  | GSTM3 | SLC35E3 |  |  |
| GOLM1 |  | HRASLS | TXNRD1 |  |  |
| GTSE1 |  | IGFBP5 | RAE1 |  |  |
| HIST1H4H |  | LGP2 | ACBD3 |  |  |
| IL18 |  | LOC286052 | ZNF274 |  |  |
| KIAA0913 |  | LOC643008 | FRG1 |  |  |
| KPNA2 |  | MCM6 | LPCAT1 |  |  |
| LST1 |  | MELK | EBP |  |  |
| MAP4 |  | MMP9 | RFC4 |  |  |
| MLF1IP |  | MS4A7 | NCAPG |  |  |
| MMP23A |  | MTDH | RNASEH2A |  |  |
| MMP23B |  | NDC80 | MED24 |  |  |
| MYH2 |  | NMU | DONSON |  |  |
| NCAPG2 |  | NUSAP1 | RMI1 |  |  |
| NEFL |  | ORC6L | PTGES |  |  |
| NEURL |  | OXCT1 | C19orf60 |  |  |
| OR12D2 |  | PALM2-AKAP2 | ISYNA1 |  |  |
| ORC3 |  | PECI | SKP2 |  |  |
| PARP4 |  | PITRM1 | DPP3 |  |  |
| PARP4P2 |  | PRC1 | TYMP |  |  |
| PHF11 |  | RAB6A | SNRPA1 |  |  |
| PLK1 |  | RFC4 | DHCR7 |  |  |
| POLQ |  | RTN4RL1 | TFPT |  |  |
| PPP1CC |  | RUNDC1 | CTTN |  |  |
| PSMC2 |  | SCUBE2 | MCM5 |  |  |
| RFX7 |  | SERF1A | TXNIP |  |  |
| RPL23AP4 |  | SLC2A3 | SYNE2 |  |  |
| RPL23AP7 |  | STK32B | SCARB2 |  |  |
| RRNAD1 |  | TGFB3 | PDLIM5 |  |  |
| SLC35A1 |  | TSPYL5 | TSC2 |  |  |
| SMC4 |  | UCHL5 | ELF1 |  |  |
| SUPT16H |  | WISP1 | DICER1 |  |  |
| TACC2 |  | ZNF533 | CALD1 |  |  |
| TESPA1 |  |  | SOX9 |  |  |
| TMEM8A |  |  | FAM20B |  |  |
| TNFSF10 |  |  | APH1A |  |  |
| TNFSF12 |  |  | AP1AR |  |  |
| TNFSF12-TNFSF13 |  |  | PDCD6 |  |  |
| TNFSF13 |  |  | PBX2 |  |  |
| UCKL1 |  |  | WASL |  |  |
| YIF1A |  |  | SLC11A2 |  |  |
| ZCCHC8 |  |  | KIAA0776 |  |  |
| ZFP36L2 |  |  | C14orf101 |  |  |
| ZNF362 |  |  | NUP107 |  |  |
|  |  |  | FAM38A |  |  |
|  |  |  | PLIN2 |  |  |
|  |  |  | AIM1 |  |  |
|  |  |  | APOC1 |  |  |
|  |  |  | APOE |  |  |
|  |  |  | DTX4 |  |  |
|  |  |  | AQP1 |  |  |
|  |  |  | LMO4 |  |  |
|  |  |  | TAF1D |  |  |
|  |  |  | SNORA25 |  |  |
|  |  |  | FMOD |  |  |
|  |  |  | RGS5 |  |  |
|  |  |  | PIK3R1 |  |  |
|  |  |  | MBNL2 |  |  |
|  |  |  | MAPKAPK2 |  |  |
|  |  |  | MTUS1 |  |  |
|  |  |  | DHX9 |  |  |
|  |  |  | PPIF |  |  |
|  |  |  | FOLR1 |  |  |
|  |  |  | KIAA1467 |  |  |
|  |  |  | SUPT4H1 |  |  |
|  |  |  | PHB |  |  |
|  |  |  | CD44 |  |  |

**Details for additional files**

**Additional file 1 (DOCX): Supplementary_materials.docx –** This document. Contains supplementary materials.

**Additional file 2 (TXT): filtered_combined_data_anno.final.train.2.txt** – Clinical annotations for 572 patients in the training data set. Includes study, GEO accessions, patient identifiers, ER status, N stage, age, tumor size, grade, treatment, relapse status, and array-based determinations of HER2 and ER status.

**Additional file 3 (TXT): filtered_combined_data_anno.final.test.2.txt** – Clinical annotations for 286 patients in the test data set. Includes study, GEO accessions, patient identifiers, ER status, N stage, age, tumor size, grade, treatment, relapse status, and array-based determinations of HER2 and ER status.

**Additional file 4 (ZIP): processed_final2_train_survival_combined_gcrma.1.txt.gz and processed_final2_train_survival_combined_gcrma.2.txt.gz** – GCRMA normalized mRNA expression values for 572 patients in the training dataset. Includes probe sets for both standard and custom CDF along with mapping to Entrez Gene identifier and gene symbol. For size reasons, this file has been divided into two parts and should be concatenated together before use.

**Additional file 5 (GZ): processed_final2_test_survival_combined_gcrma.txt.gz** – GCRMA normalized mRNA expression values for 286 patients in the test dataset. Includes probe sets for both standard and custom CDF along with mapping to Entrez Gene identifier and gene symbol.

**Additional file 6 (ZIP): Mapping of top 100 probe sets to genome for future probe design and validation**Appendix1A.pdf includes a summary of mapping validation results for the top 100 probe sets. Appendix1B.pdf includes representative images for BLAT alignment visualized in the UCSC genome browser for the top 100 probe sets.

**Additional file 7 (DOCX): Probe sequences for top 100 probe sets**Appendix2.docx includes all probe sequences for each probe set in the top 100 probe set list. For standard CDF probe sets, the exemplar and consensus sequences used by Affymetrix are also included (where available). All sequences are in fasta format.

**Additional file 8 (ZIP): Mapping of reference probe sets to genome for future probe design and validation**Appendix3A.pdf includes a summary of mapping validation results for the top 25 reference probe sets in set #1 and 15 probe sets in set #2. Appendix3B.pdf includes representative images for BLAT alignment visualized in the UCSC genome browser for the top 25 reference probe sets in set #1 and 15 probe sets in set #2.

**Additional file 9 (DOCX): Probe sequences for reference probe sets**Appendix4.docx includes all probe sequences for each probe set in the top 25 reference probe sets in set #1 and 15 probe sets in set #2. For standard CDF probe sets, the exemplar and consensus sequences used by Affymetrix are also included (where available). All sequences are in fasta format.

**Additional file 10 (ZIP): patient_data.txt and RFRS_sample_code.R** – Sample patient data and sample code for running RFRS algorithm.

**Additional file 11 (ZIP): RF_model_17gene_optimized.Rdata and RF_model_8gene_optimized.Rdata** – RFRS model file for 17-gene and 8-gene models. One of the two must be specified in RFRS_sample_code.R depending on which model the user wishes to run.

**Additional file 12 (ZIP): RelapseProbabilityPlot.Rdata and RelapseProbabilityFit.Rdata** – R data files which allow plotting of patient’s RFRS result and determination of predicted relapse probability for the report file (patient_results.pdf).

**Additional file 13 (PDF): patient_results.pdf** – Sample patient report (Also presented in Supplementary Figure 3).

**Additional file 14 (TXT): GSM_list.txt** – Complete list of GEO GSM identifiers for training and test dataset combined. To be used with R workflow sample scripts.

**Additional file 15 (ZIP): processAffyData.R and RFRS_workflow.R –** Sample R workflows that demonstrate how to download all required Affymetrix array data, summarize and normalize probe sets, map probe identifiers to gene symbols, filter non-expressed or invariant probe sets, build a random forests model, perform mixed-model clustering to identify cut-offs, calculate ROC AUC values, apply random forest models to new data, down-sample relapses to realistic population frequency, and perform k-means clustering to identify redundant/robust alternatives for an optimal probe set.
