# Supplementary material for: A robust prognostic signature for hormone-positive node-negative breast cancer
Source: Genome Med. 2013 Oct 11;5(10):92. doi: 10.1186/gm496 (PMC3961800; doi:10.1186/gm496)
Supplement: Additional file 6 — Describes alignment of the top 100 probe sets to the reference genome. [file gm496-S6.zip › Addfile 6 and 7 (5)/1217714361945551_add6.pdf]

Appendix 3A

Gene (probe set)	CDF	Predictor Status	Mapping comments
CCNB2 (9133_at)	custom	predictor1	All nine probes map perfectly to CCNB2 locus on chr 15. Two probes map with single mismatch to intergenic region on chr7 (gene desert).
MELK (9833_at)	custom	predictor1 alternate1	All probes map perfectly to 3' exon of MELK locus on chr9
GINS1 (9837_at)	custom	predictor1 alternate2	All 10 probes map perfectly to 5' UTR of GINS1 locus on chr20. Probe 4 maps with little or no mismatch to intergenic/intronic regions on 21 other chromosomes.
RRM2 (6241_at)	custom	predictor1 alternate3	All probes map perfectly to 3' UTR of RRM2 locus on chr2. 3 probes also map with little mismatch to intronic region on chrX. Probe 6 also maps to intergenic region on chr1.
GINS2 (51659_at)	custom	predictor1 alternate4	All probes map perfectly to 3' UTR and exon nearest 3' end of GINS2 locus on chr16. Probe 5 also maps to intergenic region on chr20. Probe 1 also maps to intergenic region on chrX.
CCNB1 (214710_s_at)	standard	predictor1 alternate5	All probes (as well as a very poor alternate mapping of consensus) map perfectly to 3' UTR and exons 1-3 (from 3' end) of CCNB1 locus on chr5.
TOP2A (201291_s_at)	standard	predictor2	Consensus and target sequences map with near perfect and unambiguous. 10/11 probes perfect match. One probe with single mismatch. No ambiguous probes.
MCM2 (4171_at)	custom	predictor2 alternate1	All probes map perfectly to 3'UTR of MCM2 locus on chr3.
KIAA0101 (9768_at)	custom	predictor2 alternate2 (excluded)	All 20 probes map perfectly to exon nearest to 3' end and 3' UTR of KIAA0101 locus on chr15. Probe 2 also maps to 3' UTR of KIAA1751 locus on chr1 with 21 bases matching perfectly, as well as to antisense cDNA clone AX747205 on chr1 with 20 bases matching perfectly.
CDK1 (203213_at)	standard	predictor2 alternate3	All probes map perfectly to 3' UTR of CDC2 (aka CDK1) locus on chr10. Probe 8 also maps to intergenic region on chr22.
UBE2C (202954_at)	standard	predictor2 alternate4	All probes map perfectly or with little mismatch to 3' UTR or exons 2-4 (from 3' end) of UBE2C locus on chr20. 2 probes also map to intronic region on chr4, 4 probes also map to intronic region on chr14, 1 probe also maps to intergenic region on chr18, 2 probes also map to intronic region on chr19, and 2 probes also map to intergenic region on chr15.

TMEM97 (212281_s_at)	standard	predictor2 alternate5	All probes map perfectly to 3' UTR of TMEM97 locus on chr17. Probe 1 also maps to intergenic/intergonic region on chrs 1, 8, and 6.
DTL (218585_s_at)	standard	predictor2 alternate6	All probes map perfectly to 3' UTR of DTL locus on chr1. Probe8 also maps to intronic region on chr8.
RACGAP1 (29127_at)	custom	predictor3	All five probes map perfectly to RACGAP1 locus on chr12. Two probes map with single mismatch/indel to intergenic regions on chr12 (upstream of FKSG42) and chr9 (gene desert).
LSM1 (27257_at)	custom	predictor3 alternate1	All probes map perfectly to 3' exon of LSM1 locus on chr8. One probe also partially mapped (pos 1 to 20) to intergenic region of chr21.
SCD (200832_s_at)	standard	predictor3 alternate2	All 11 probes map perfectly to 3' UTR of SCD locus on chr10. 6 probes also map to probably noncoding cDNA clone CR620692 on chr17.
HN1 (51155_at)	custom	predictor3 alternate3	All probes map perfectly across all exons of HN1 locus on chr17. 3 probes also map to intergenic region on chr2.
CKS2 (1164_at)	custom	predictor4	All three probes map perfectly to CKS2 locus on chr9. No ambiguous mappings.
NUSAP1 (218039_at)	standard	predictor4 alternate1	Exemplar, target, and probes map well to 3' UTR of NUSAP1 locus on chr15. Three probes also map with split alignments to intronic/intergenic regions
PTTG1 (203554_x_at)	standard	predictor4 alternate2 (excluded)	All but probe 8 map perfectly to 3 exons and UTR nearest to 3' end of PTTG1 locus on chr5. Probe 8 and 3 other probes map with some mismatch to single exon of coding gene PTTG3 on chr8. 5 probes map with little or no mismatch to single exon of coding gene PTTG2 on chr4.
ZWINT (204026_s_at)	standard	predictor4 alternate3	All probes map perfectly to 3' UTR of ZWINT locus on chr10. Probe 3 also maps to intergenic region on chr10. Probe 11 maps to an intronic region on two other chromosomes as well.
TYMS (7298_at)	custom	predictor4 alternate4	All probes map perfectly to 3' UTR and exon nearest 3' end of TYMS (aka TS) on chr18. For 4 probes this overlaps with 3' UTR of ENOSF1 on opposite strand.
MLF1IP (218883_s_at)	standard	predictor4 alternate5	All probes map perfectly or with little mismatch to 3' UTR of MLF1IP (aka KLIP1) locus on chr4. Probe 1 also maps to intronic region on chrX.
SQLE (209218_at)	standard	predictor4 alternate6	All probes map perfectly to 3' UTR or exons 1-2 (from 3' end) of SQLE

			locus on chr8.
AURKA (208079_s_at)	standard	predictor5	Exemplar and target sequences have reasonable alignment to correct locus. However all 11 probes have perfect alignments and only a single probe had alignments elsewhere with mismatch.
PRC1 (9055_at)	custom	predictor5 alternate1	All probes map perfectly to 3'UTR of PRC1 locus on chr15. One probe maps partially (pos 4 to 23) to intergenic region on chr21. One probe maps (split alignment) to intronic region of MYOF on chr10.
CENPF (207828_s_at)	standard	predictor5 alternate2	All 11 probes map perfectly to 3' UTR and exon nearest 3' of CENPF locus on chr1. Probe 11 also maps to intergenic region on chr12.
ASPM (219918_s_at)	standard	predictor5 alternate3	All probes but probe 1 map perfectly to the 4 exons nearest to the 3' end of ASPM locus on chr1. Probe 1 failed to align.
NEK2 (204641_at)	standard	predictor5 alternate4	All probes but probe 10 map perfectly to 3' UTR of NEK2 locus on chr1. Probe 10 failed to align. 4 probes also map perfectly or with some mismatch to intergenic region on chr22, 5 probes also map perfectly or with somoe mismatch to intergenic region on ch14, 3 probes also map perfectly to intronic region on chr2, and probe 6 also maps with some mismatch to intronic region on chr3.
ECT2 (1894_at)	custom	predictor5 alternate5	All probes map perfectly to 3' UTR of ECT2 locus (aka DKFZp434C0523) on chr3. Probe 9 also maps to intergenic region on chrs 2 and 6. Probe 4 maps to intergonic region on chr12.
FEN1 (204767_s_at)	standard	predictor6	Exemplar, target, and probes map well to expected FEN1 locus (3'UTR). However, exemplar, target, and 5/11 probes also map to intronic region of HFM1 with some mismatches
FADD (8772_at)	custom	predictor6 alternate1	All probes map perfectly to 3'UTR of FADD locus on chr11.
SMC4 (10051_at)	custom	predictor6 alternate2	All 21 probes map perfectly to 2 exons nearest 3' end and 3' UTR of SMC4 locus on chr3 (overlap intronic region of IFT80). Probe 16 also maps almost perfectly to intronic region on chr11.
SLC35E3 (55508_at)	custom	predictor6 alternate3	All probes map perfectly to 3' UTR of SLC35E3 locus on chr12.
TXNRD1 (7296_at)	custom	predictor6 alternate4	All probes map perfectly to 3' UTR of TXNRD1 (aka TR) on chr12. Probe 1 also maps to intronic region on chr5.
RAE1 (211318_s_at)	standard	predictor6 alternate5	All probes map perfectly to 3' UTR or exon 1 (or 2, depending on

			isoform and counting from 3' end) of RAE1 locus on chr20.
ACBD3 (202323_s_at)	standard	predictor6 alternate6	All probes map perfectly or with little mismatch to 5' UTR or exons 1-3 (from 5' end) of ACBD3 locus on chr1.
ZNF274 (204937_s_at)	standard	predictor6 alternate7	All probes map perfectly to 3' UTR or exon 1 (from 3' end) of ZNF274 locus on chr19. Many poor mappings of exemplar.
FRG1 (2483_at)	custom	predictor6 alternate8 (excluded)	All probes map perfectly or with little mismatch to exons 2, 5, and 6 (from 3' end) of FRG1 locus on chr4. Probes 1 and 3 also map to FRG1B locus on chr20. Probe 2 also maps to intronic region of unknown contig Un_gl000219 and to noncoding cDNA clone AK096159 (and intronic overlaps) on chr9. Probes 3 and 4 also map to noncoding cDNA clone BC063132 on unknown contig Un_gl000241. Probe 3 also maps to intronic region on unknown contig Un_gl000219.
LPCAT1 (201818_at)	standard	predictor6 alternate9	All probes map perfectly to 3' UTR of LPCAT1 locus on chr5. Probe 6 failed to align.
EBP (10682_at)	custom	predictor7	All probes map perfectly to expected EBP locus on chrX. One probe also mapped to intron of LOC729085 on chr3 (with a single mismatch).
RFC4 (204023_at)	standard	predictor7 alternate1	Exemplar, target, and probes map well to 5 (3'-most) exons of RFC4 locus on chr3.
NCAPG (218662_s_at)	standard	predictor7 alternate2	All 11 probes map perfectly to 3' UTR and exon nearest 3' end of NCAPG locus on ch4. For 9 probes this overlaps with 3' UTR of LCORL on opposite strand. 2 probes also map to intergenic/intronic regions on 2 other chrs.
RNASEH2A (10535_at)	custom	predictor7 alternate3	All probes map with little or no mismatch to exons 1, 3 and 4 (from 3' end) of RNASEH2A locus on chr19.
MED24 (9862_at)	custom	predictor7 alternate4	All probes map perfectly to 3' UTR and 3' most exon of MED24 (aka THRAP4) locus on chr17. Probe 5 also maps to intergenic region on chr5.
DONSON (29980_at)	custom	predictor7 alternate5	All probes map perfectly to 3' UTR or exons 1-2 (from 3' end) of DONSON locus and overlap same exons of cDNA clone C21orf60 on chr21. 3 probes also map to intronic region on chr3.
RMI1 (80010_at)	custom	predictor7 alternate6	All probes map perfectly to 3' UTR of RMI1 locus on chr9. Probe 7 also

			maps to intronic region on chr12. Probe 3 also maps to intergenic region on chr18.
PTGES (9536_at)	custom	predictor7 alternate7	All probes map perfectly to 3' UTR or exon 1 (from 3' end) of PTGES (aka PIG12) locus on chr9. Probe 5 also maps to intronic region on chr16.
C19orf60 (51200_at)	standard	predictor7 alternate8	All probes map perfectly or with little mismatch to 3' UTR or exons 1-3 (from 3' end) of C19orf60 locus on chr19.
ISYNA1 (222240_s_at)	standard	predictor7 alternate9	All probes map perfectly to exons 1-3 (from 3' end) of ISYNA1 locus on chr19. 2 probes also map with some mismatch to intergenic region on chr4.
SKP2 (203625_x_at)	standard	predictor7 alternate10	All probes map perfectly to exon 1 (from 3' end) of SKP2 locus on chr5. 6 probes also map to intergenic region on chr3. 2 probes also map to intergenic region on chrX.
DPP3 (218567_x_at)	standard	predictor7 alternate11 (excluded)	All probes map perfectly to 3' UTR and exon 1 (from 3' end) of DPP3 locus on chr11, and 4 probes also overlap with 5' UTR and exon 1 (from 5' end) of BBS1 locus. 3 probes also map to intergenic region on chr9, 4 probes also map to intergenic region on chr4, and probe 11 also maps to intergenic region on chr2.
TYMP (204858_s_at)	standard	predictor7 alternate12	All probes map perfectly to exons 2-4 (from 3' end) of TYMP locus on chr22.
SNRPA1 (216977_x_at)	standard	predictor7 alternate13	All probes map perfectly or with little mismatch to exons 2-5 and 9 (from 3' end) of SNRPA1 locus on chr15. 8 probes also map perfectly or with little mismatch to intergenic region on chr2, and 8 other probes also map perfectly or with little mismatch to intergenic region on chr 15.
DHCR7 (201791_s_at)	standard	predictor7 alternate14	All probes map perfectly to 3' UTR of DHCR7 locus on chr11. Probe 8 also maps to intergenic region on chr14.
TFPT (218996_at)	standard	predictor7 alternate15	All probes but probe 3 map perfectly or with little mismatch to exons 1-5 (from 3' end) of TFPT locus on chr19. Probe 3 failed to align.
CTTN (2017_at)	custom	predictor7 alternate16	All probes map perfectly to exons 2 and 4 (from 5' end), 3' UTR, or exon nearest 3' end of CCTN locus on chr11. Probe 7 also maps with some mismatch to intergenic region on chr11. Probe 8 also maps to intronic

			region on chr12.
MCM5 (216237_s_at)	standard	predictor7 alternate17	All probes map perfectl to 3' UTR or exon nearest 3' end of MCM5 locus on chr22.
TXNIP (10628_at)	custom	predictor8	All probes map perfectly to expected TXNIP locus on chr1. One probe also mapped to intron of BC068246 on chr3 (split alignment).
SYNE2 (23224_at)	custom	predictor9	All probes map perfectly to 3'UTR of SYNE2 locus on chr14. One probe also mapped to intron of TP53BP2 on chr1 (split alignment).
SCARB2 (201646_at)	standard	predictor9 alternate1	Consensus, target, and probes map well to 3' UTR ofSCARB2 on chr4. One probe also maps (with single mismatches) to multiple other locations (all intergenic/intronic).
PDLIM5 (216804_s_at)	standard	predictor9 alternate2	All probes but probe 1 map perfectly to 2 exons nearest 3' end of PDLIM5 and LIM locus (aka PDLIM5) on chr4. Probe 1 failed to align. Many alternate mappings of consensus.
TSC2 (7249_at)	custom	predictor9 alternate3	All probes map perfectly to exons 1, 3, and 4 (from 3' end) of TSC2 locus on chr16 and overlap same exons of 2 variant protein (aka a part of TSC2).
ELF1 (212420_at)	standard	predictor9 alternate4	All probes but probe 7 map perfectly to 5' UTR and exons 2-4 (from 5' end) of ELF1 locus on chr13. Probe 7 failed to align.
DICER1 (23405_at)	custom	predictor10	All probes map perfectly to 3'UTR of DICER1 locus on chr14. Nine probes also map to intergenic region on chr5 (one perfect but split, eight with one or more mismatches).
CALD1 (201616_s_at)	standard	predictor10 alternate1	Consensus, target, and probes map well to 2 (3'-most) exons of CALD1 locus on chr7. Only probe1 does not map.
SOX9 (6662_at)	custom	predictor10 alternate2	All probes map perfectly to 3' UTR of SOX9 locus on chr17. 4 probes map to intronic regions on other chrs as well.
FAM20B (202915_s_at)	standard	predictor10 alternate3	All probes map perfectly to 3' UTR of FAM20B locus on chr1. Probe 8 also maps to intergenic region on chr14. Many poor alternate consensus mappings.
APH1A (218389_s_at)	standard	predictor10 alternate4	All probes map perfectly to 3' UTR of APH1A (aka PSF) locus on chr1. Probe 9 also maps with some mismatch to intronic region on chr20.
AP1AR (55435_at)	custom	predictor11	All probes map perfectly to 3'UTR of AP1AR locus on chr4. One probe

			also partially mapped (pos 5 to 25) to intergenic region (gene desert) of chr9.
PDCD6 (222380_s_at)	standard	predictor11 alternate1 (excluded)	Consensus, target, and probes map approximately equally well to multiple regions, the closest to expected is PDCD6 intron.
PBX2 (202876_s_at)	standard	predictor11 alternate2	All probes map perfectly or almost perfectly to 3' UTR of PBX2 locus on chr6. All probes also map perfectly or almost perfectly to 3' UTR of PBX2 locus on chr6 haplotype contigs.
WASL (205809_s_at)	standard	predictor11 alternate3	All probes but probe 7 map perfectly or with little mismatch to exons 6-9 (from 3' end) of WASL locus on chr7. Probe 7 failed to align.
SLC11A2 (203123_s_at)	standard	predictor11 alternate4	All probes map perfectly to 3' UTR of SLC11A2 locus on chr12. Several other transcripts of this gene do not include the same 3' UTR (probes align to introns of those transcripts). Probe 4 also maps with some mismatch to intronic region on chr5. Probe 5 also maps with some mismatch to intergenic region on chr4.
KIAA0776 (212634_at)	standard	predictor11 alternate5 (excluded)	All probes map perfectly to 3' UTR of KIAA0776 locus on chr6. Probe 3 also maps to intergenic region on chr6. Probe 2 also maps to intergenic region on chr5.
C14orf101 (54916_at)	custom	predictor11 alternate6	All probes map perfectly to 3' UTR of C14orf101 locus on chr14.
NUP107 (57122_at)	custom	predictor12	All probes map perfectly to two 3' most exons of NUP107 locus on chr12
FAM38A (202771_at)	standard	predictor12 alternate1	Exemplar, target, and probes map well to 2 (3'-most) exons of FAM38A locus on chr16. One probe maps with mismatch to intergenic region on chr20.
PLIN2 (209122_at)	standard	predictor12 alternate2	All probes map perfectly to 3' UTR or exon nearest 3' end of PLIN2 locus on chr9. Probe 6 also maps perfectly to intronic region on chr1.
AIM1 (212543_at)	standard	predictor12 alternate3	All probes map perfectly to 3' UTR of AIM1 locus. Many poor alternate mappings of consensus.
APOC1 (204416_x_at)	standard	predictor13	Exemplar, target, and probes map well across all 4 exons of medium length isoform of APOC1 on chr19. Exemplar, target and 3 probes also align (with some mismatches) to downstream (APOC1P1 pseudogene) EF553526

APOE (203382_s_at)	standard	predictor13 alternate1	Exemplar, target, and probes map well to 3' exon of APOE locus on chr19.
DTX4 (23220_at)	custom	predictor14	All probes map perfectly to 3'UTR of DTX4 locus on chr11. One probe also partially mapped (pos 1 to 20) to intergenic regions of chr9 and chr11.
AQP1 (358_at)	custom	predictor14 alternate1	All 22 probes map perfectly to 3' UTR of AQP1 locus on chr7. Four probes have imperfect alignment to intron/intergenic regions. One probe aligns to KLK3 CDS with single mismatch.
LMO4 (209205_s_at)	standard	predictor14 alternate2	All probes map perfectly to 3' UTR of LMO4 locus on chr1.
TAF1D (218750_at)	standard	predictor15 (excluded)	Exemplar, target, and probes map well to TAF1D locus on chr11. Four probes also have reasonable matches elsewhere. Two probes align just downstream of 3' end of gene and overlap with another gene on the opposite strand. The other nine probes align in intron of TAF1D although some overlap with SNORA25.
SNORA25 (684959_at)	custom	predictor15 alternate1 (excluded)	All 6 probes map perfectly to SNORA25 locus on chr11. Two probes also map imperfectly to other intergenic/intronic regions. Note, SNORA25 is in the intron of TAF1D.
FMOD (202709_at)	standard	predictor16	Exemplar, target, and probes map very well to expected FMOD locus (3'UTR) on chr1. One probe has partial alignment (pos 4 to 25) to intron of COX7B2.
RGS5 (8490_at)	custom	predictor16 alternate1	All 33 probes map perfectly to 3' exon of RGS5 locus on chr1. Four probes also map with mismatches to other intron/intergenic regions.
PIK3R1 (212239_at)	standard	predictor16 alternate2	All probes map perfectly to 3' UTR of PIK3R1 locus on chr5. Probe 8 also maps almost perfectly to intronic region on chr11.
MBNL2 (203640_at)	standard	predictor16 alternate3	All probes map perfectly to 3' UTR of MBNL2 locus on chr13.
MAPKAPK2 (201461_s_at)	standard	predictor17	Exemplar, target, and probes map very well to expected MAPKAPK2 locus (3' end) on chr1. No probes mapping elsewhere.
MTUS1 (212093_s_at)	standard	predictor17 alternate1	All 11 probes map perfectly to 3' UTR of MTUS1 locus on chr8. One probe with mismatch and partial alignment to intergenic region on chr3.
DHX9 (212107_s_at)	standard	predictor17 alternate2	All but probe 9 map perfectly or with some mismatch to exons 14-16 from 3' end of DHX9 on chr1. Probe 9 and 3 other probes map to

			intergenic region on chr13. 2 probes also map to intergenic region on chr11.
PPIF (201490_s_at)	standard	predictor17 alternate3	All probes map perfeclty to 3' UTR and exons 1-5 (from 3' end) of PPIF locus on chr10. Probe 6 also maps to intronic region on chr6.
FOLR1 (211074_at)	standard	predictor18 (excluded)	Exemplar, target, and probes map well to multiple loci (21, Un_gl000220, 16, Un_gl000229, Y, 2), none of which correspond to expected locus for FOLR1 on chr11 or any obvious CDS
KIAA1467 (57613_at)	custom	predictor19 (excluded)	All probes map perfectly to 3'UTR of KIAA1467 locus on chr12.
SUPT4H1 (201483_s_at)	standard	predictor20	Exemplar, target, and probes map well to 4 exons of expected SUPT4H1 locus on chr17. However 4 probes also map reasonably well to intergenic region just upstream of CLEC4D on chr12.
PHB (200658_s_at)	standard	predictor20 alternate1	All but probe 9 map perfectly to 3 exons/UTR nearest to 3' end of PHB on chr17. Eight probes, target, and consensus map imperfectly to intergenic regions on various other chromosomes.
CD44 (204489_s_at)	standard	predictor20 alternate2	All probes map perfectly to 3' UTR of CD44 locus on chr11.
