# Supplementary material for: A robust prognostic signature for hormone-positive node-negative breast cancer
Source: Genome Med. 2013 Oct 11;5(10):92. doi: 10.1186/gm496 (PMC3961800; doi:10.1186/gm496)

Appendix 3B

CCNB2 (9133_at)

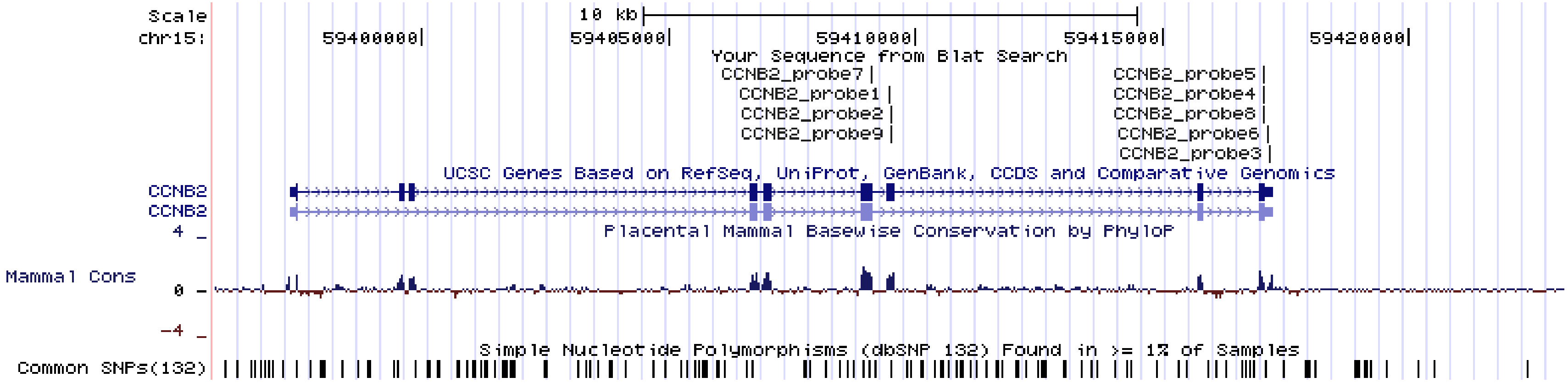

MELK (9833_at)

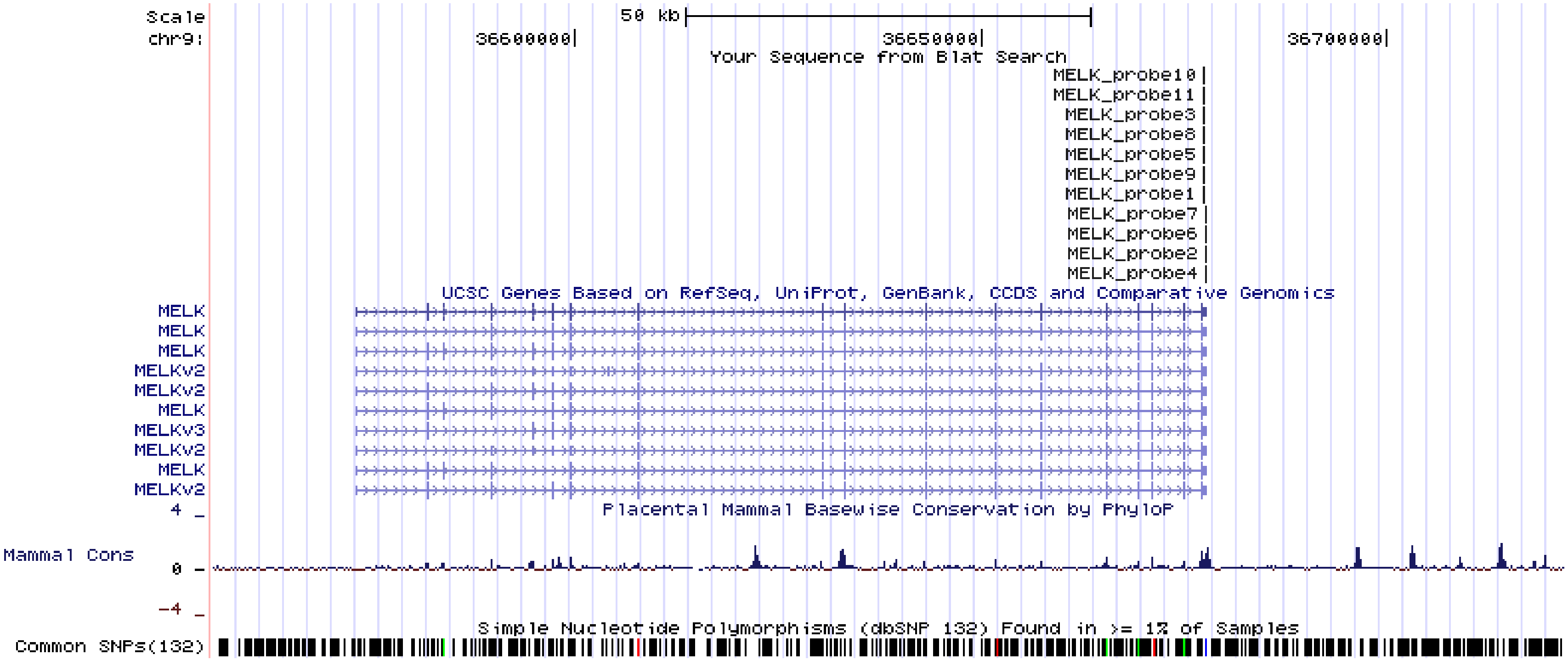
Scale
chr9:
50 kb
36600000
36650000
36700000
Your Sequence from Blat Search
MELK_probe10
MELK_probe11
MELK_probe3
MELK_probe8
MELK_probe5
MELK_probe9
MELK_probe1
MELK_probe7
MELK_probe6
MELK_probe2
MELK_probe4
UCSC Genes Based on RefSeq, UniProt, GenBank, CCDS and Comparative Genomics
MELK
MELK
MELK
MELKv2
MELKv2
MELK
MELKv3
MELKv2
MELK
MELKv2
Placental Mammal Basewise Conservation by PhyloP
4
Mammal Cons
0
-4
Simple Nucleotide Polymorphisms (dbSNP 132) Found in >= 1% of Samples
Common SNPs(132)

GINS1 (9837_at)

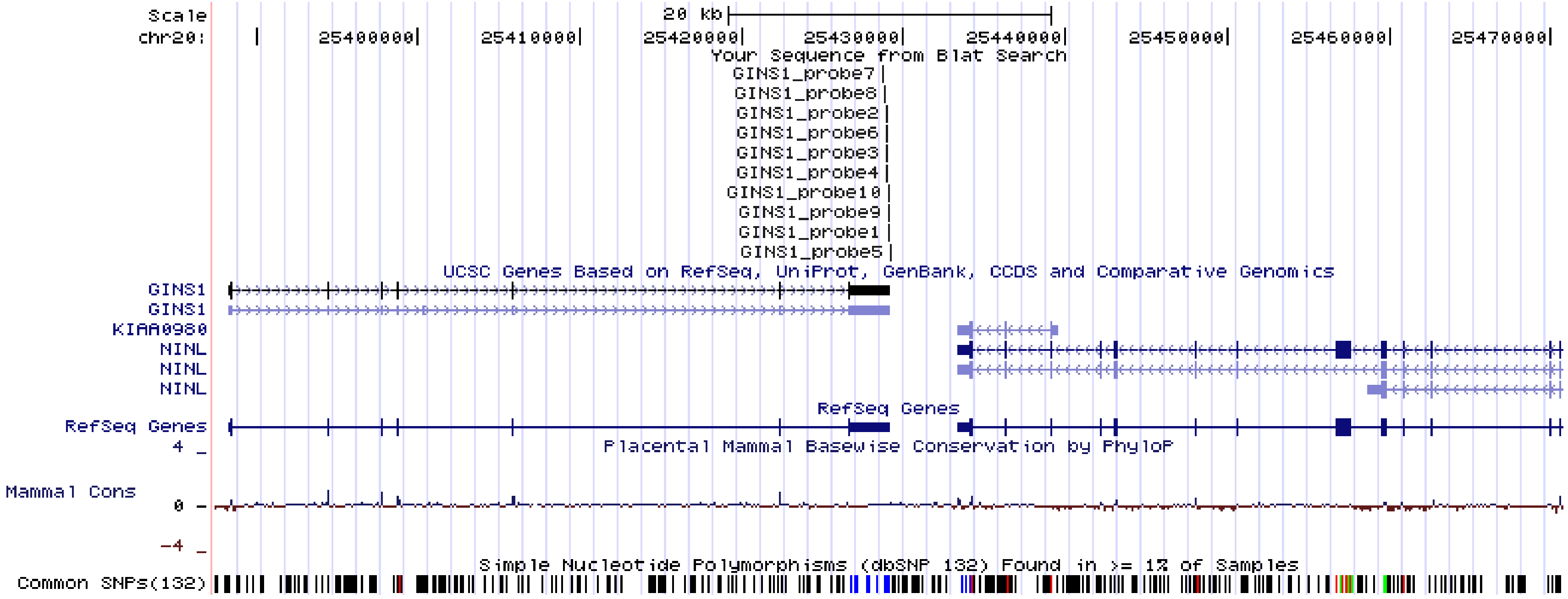

RRM2 (6241_at)

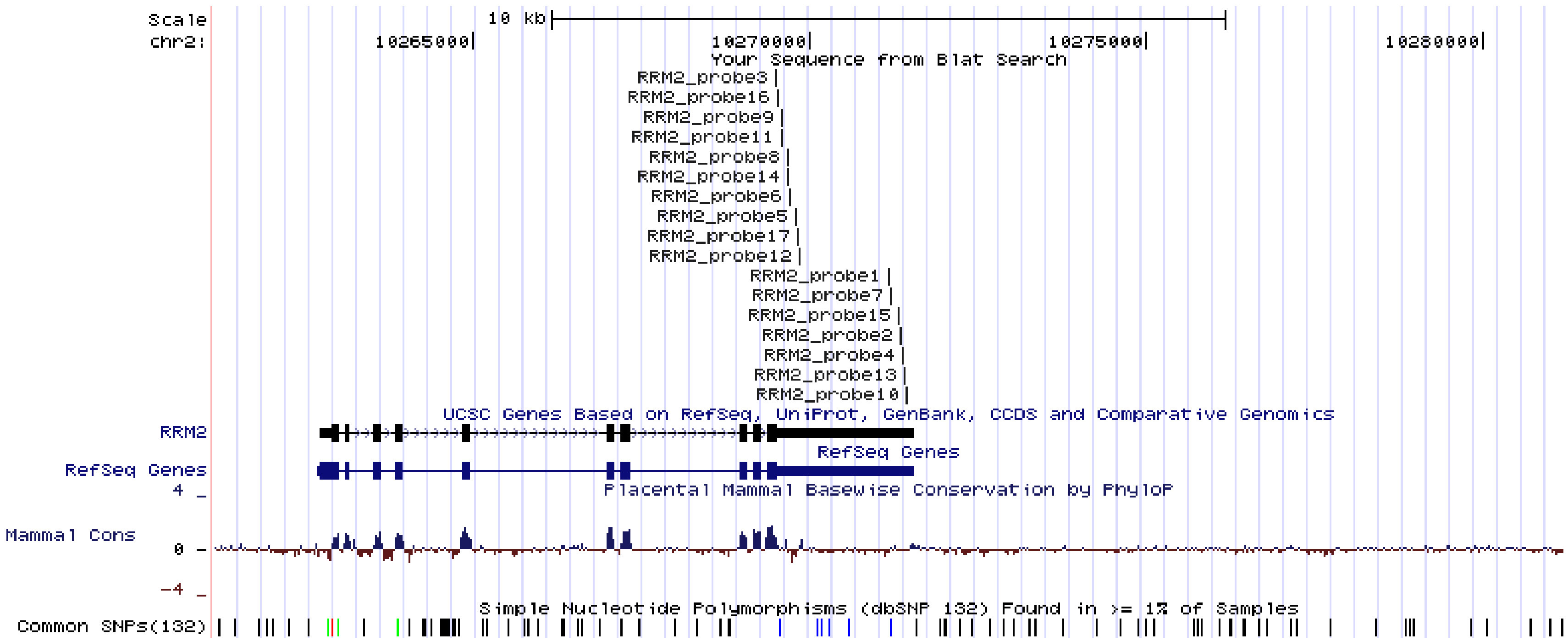
Scale
chr2:
10 kb
10265000
10270000
10275000
10280000
Your Sequence from Blat Search
RRM2_probe3
RRM2_probe16
RRM2_probe9
RRM2_probe11
RRM2_probe8
RRM2_probe14
RRM2_probe6
RRM2_probe5
RRM2_probe17
RRM2_probe12
RRM2_probe1
RRM2_probe7
RRM2_probe15
RRM2_probe2
RRM2_probe4
RRM2_probe13
RRM2_probe10
UCSC Genes Based on RefSeq, UniProt, GenBank, CCDS and Comparative Genomics
RRM2
RefSeq Genes
RefSeq Genes
4
Placental Mammal Basewise Conservation by PhyloP
Mammal Cons
0
-4
Simple Nucleotide Polymorphisms (dbSNP 132) Found in >= 1% of Samples
Common SNPs(132)

GINS2 (51659_at)

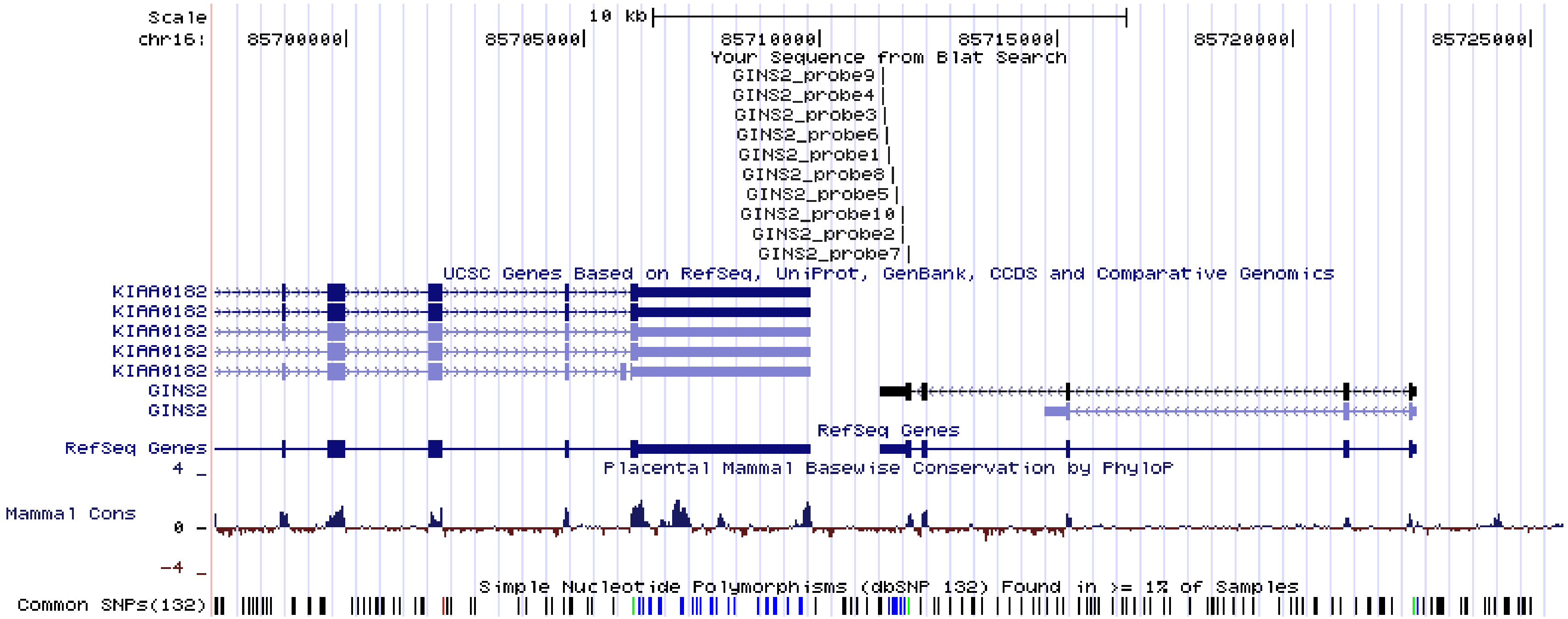

CCNB1 (214710_s_at)

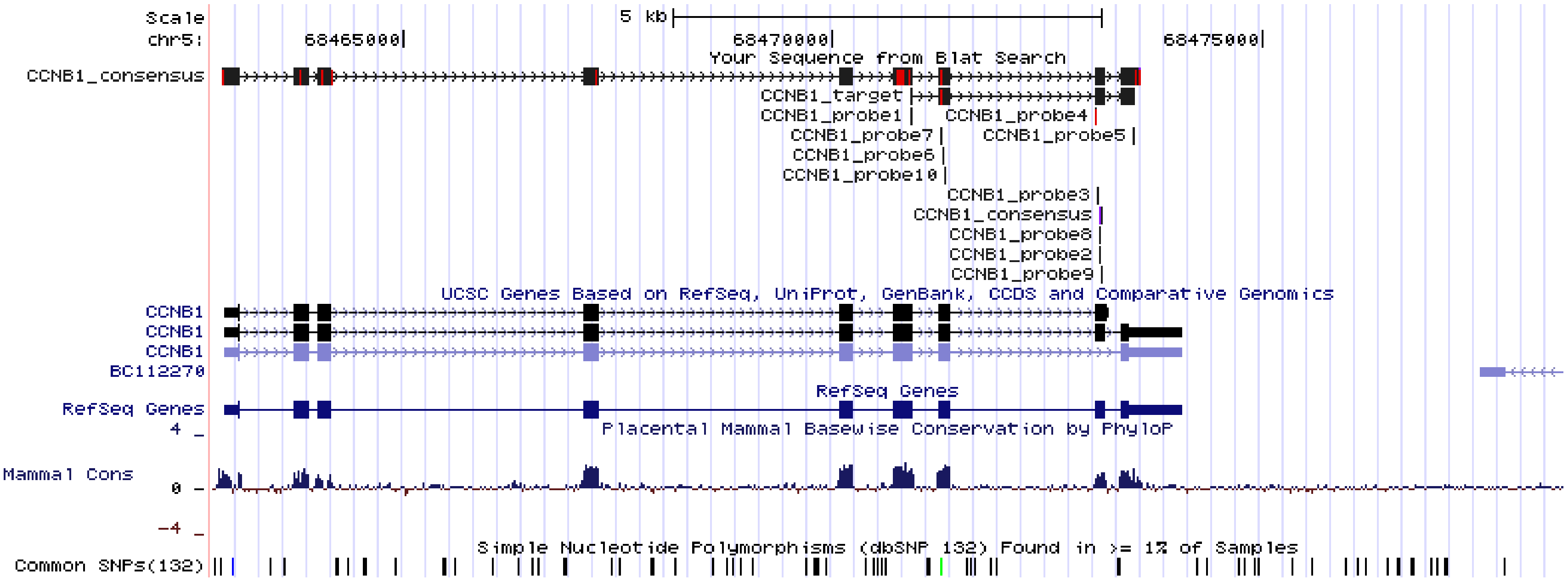

TOP2A (201291_s_at)

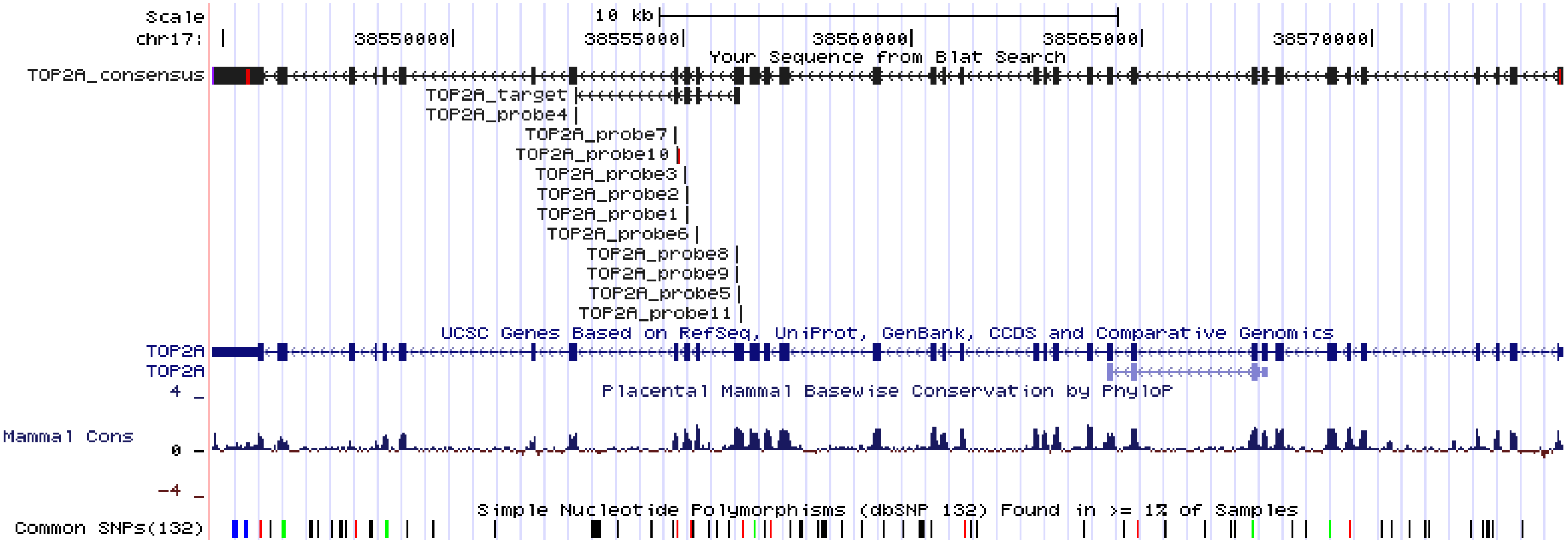

MCM2 (4171_at)

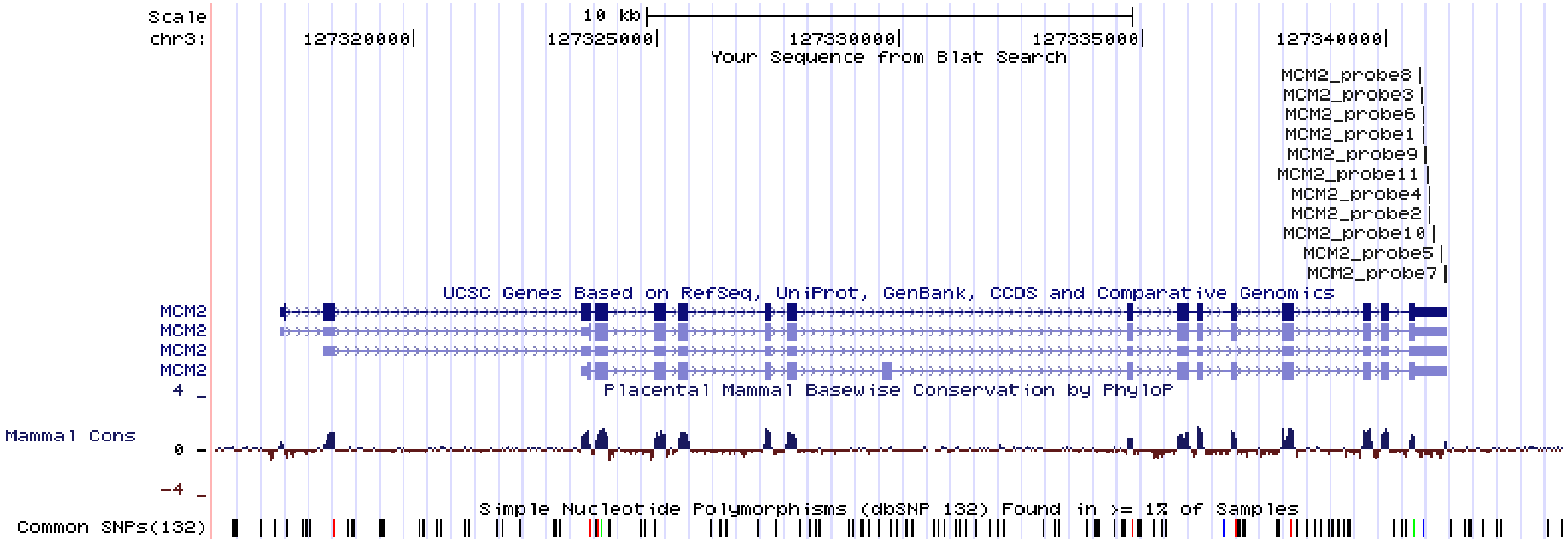
Scale
chr3:
10 kb
127320000
127325000
127330000
127335000
127340000
Your Sequence from Blat Search
MCM2_probe8
MCM2_probe3
MCM2_probe6
MCM2_probe1
MCM2_probe9
MCM2_probe11
MCM2_probe4
MCM2_probe2
MCM2_probe10
MCM2_probe5
MCM2_probe7
UCSC Genes Based on RefSeq, UniProt, GenBank, CCDS and Comparative Genomics
MCM2
MCM2
MCM2
MCM2
4
Placental Mammal Basewise Conservation by PhyloP
Mammal Cons
0
-4
Simple Nucleotide Polymorphisms (dbSNP 132) Found in >= 1% of Samples
Common SNPs(132)

KIAA0101 (9768_at)

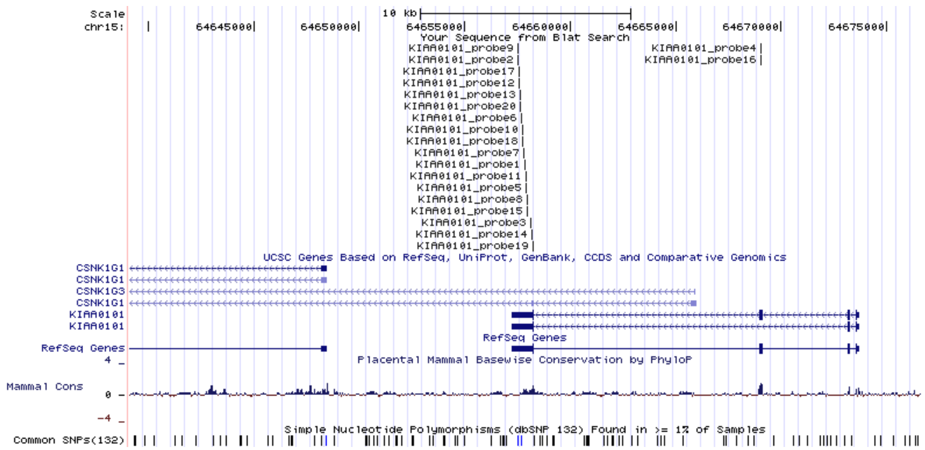

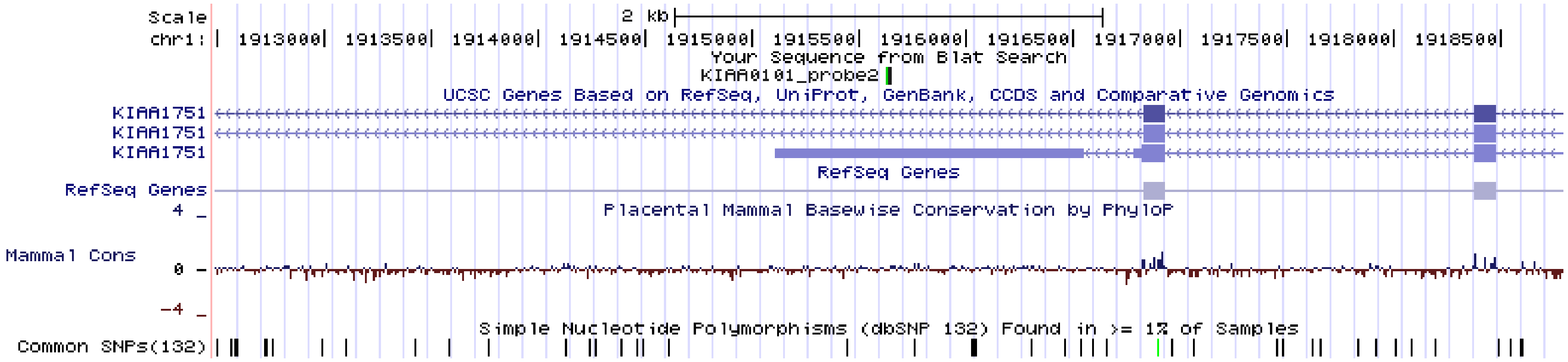

CDK1 (203213_at)

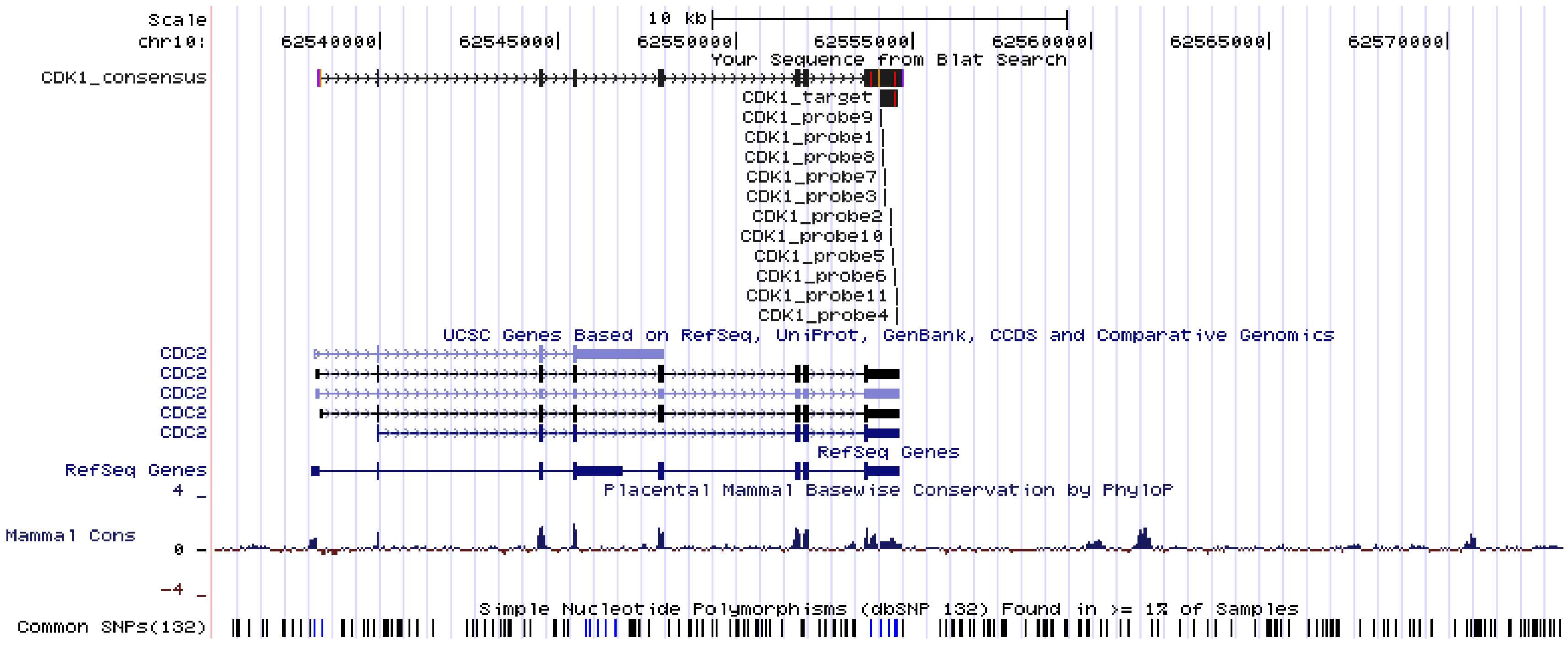

UBE2C (202954_at)

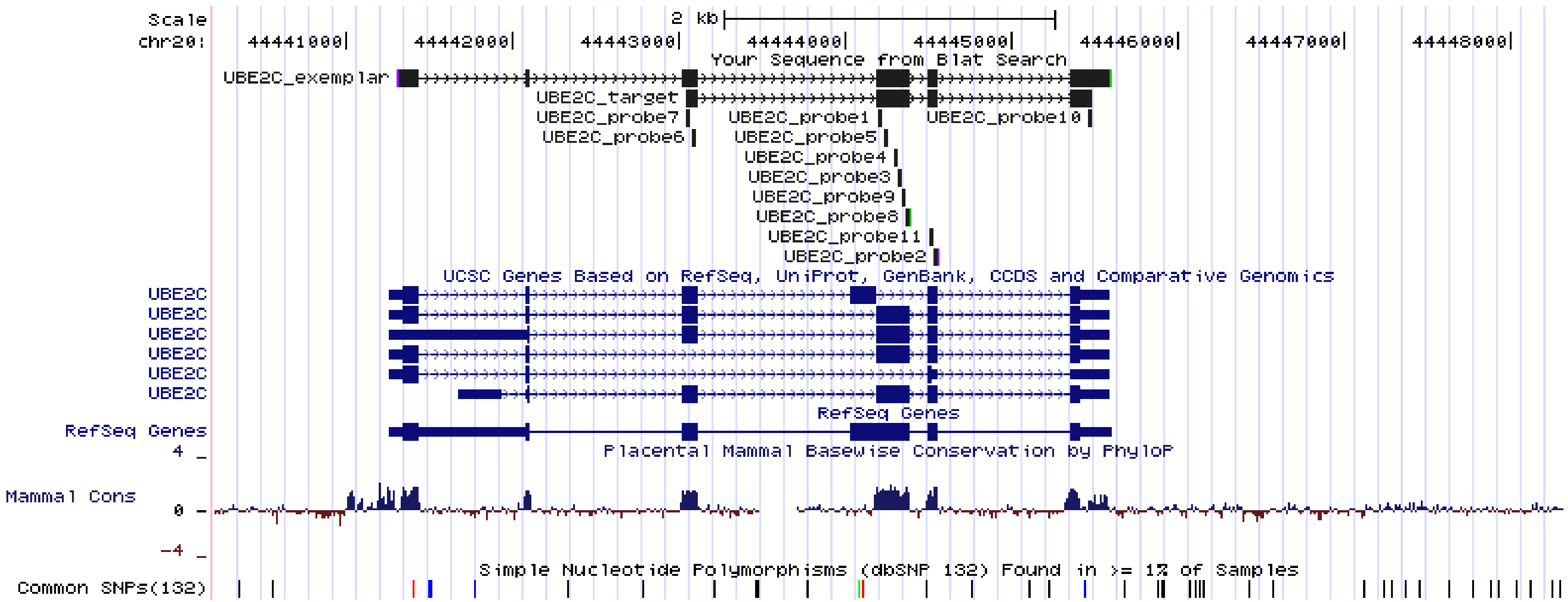

TMEM97 (212281_s_at)

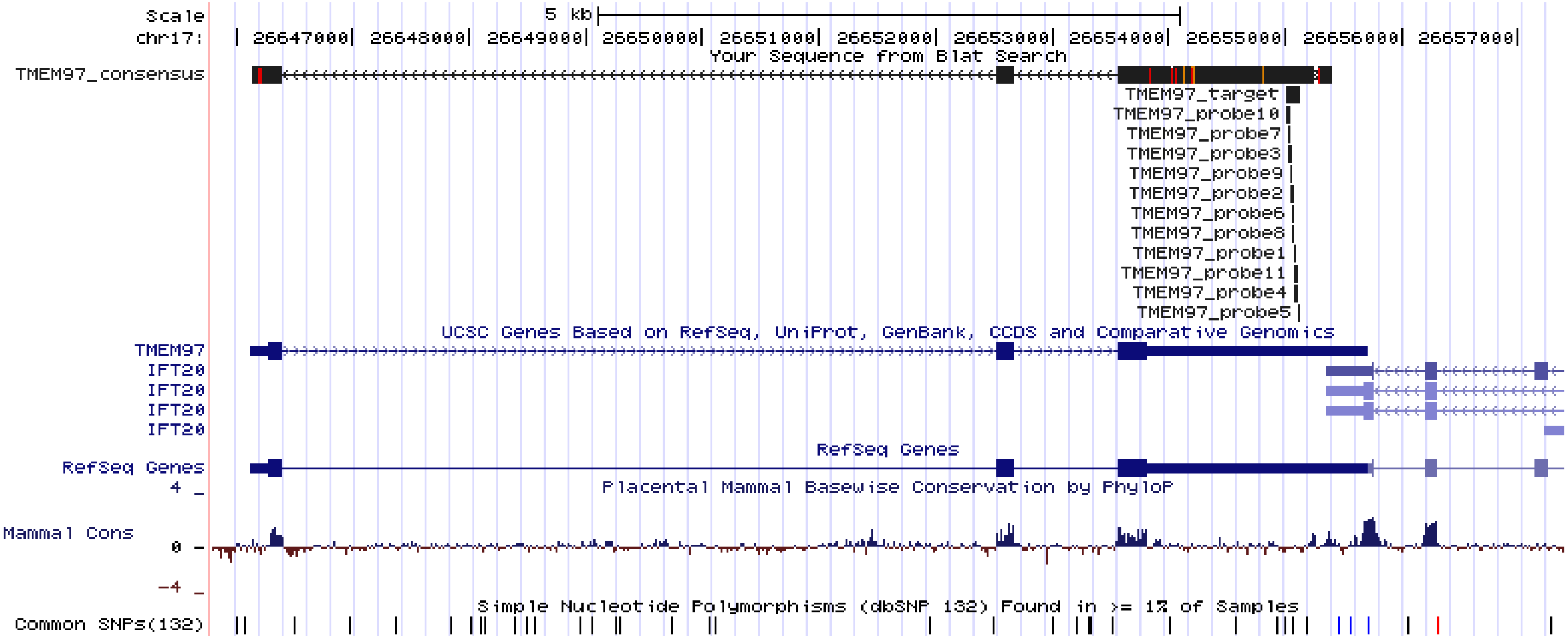

DTL (218585_s_at)

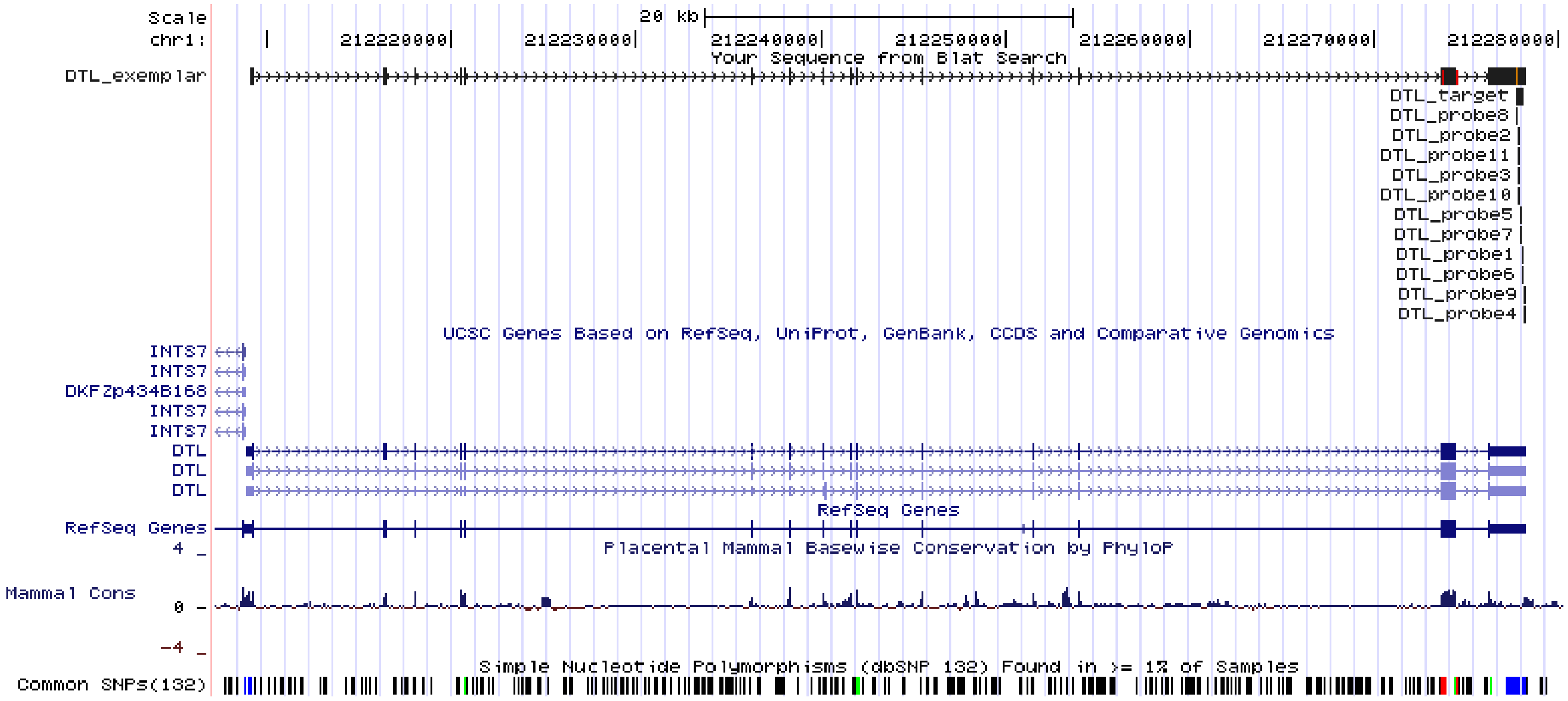

RACGAP1 (29127_at)

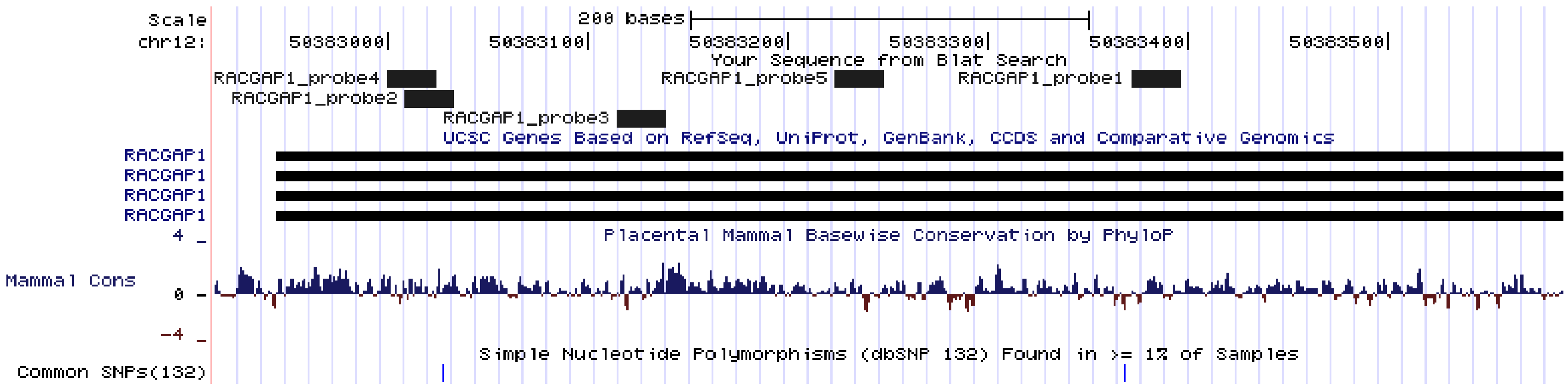

LSM1 (27257_at)

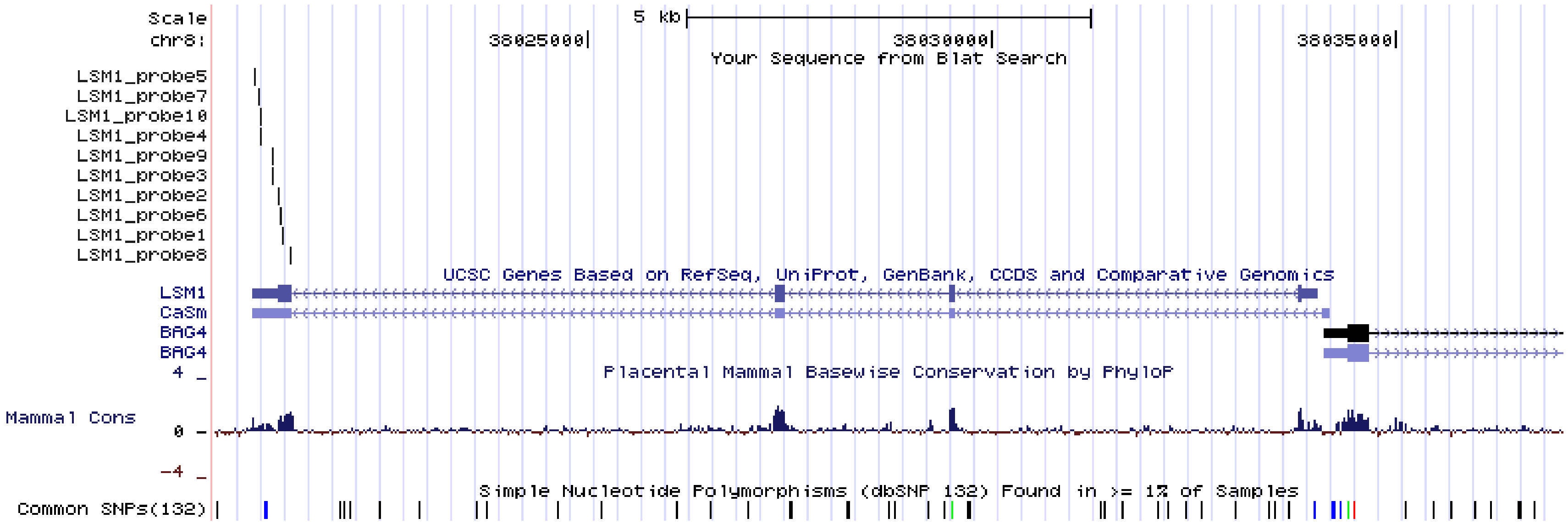

SCD (200832_s_at)

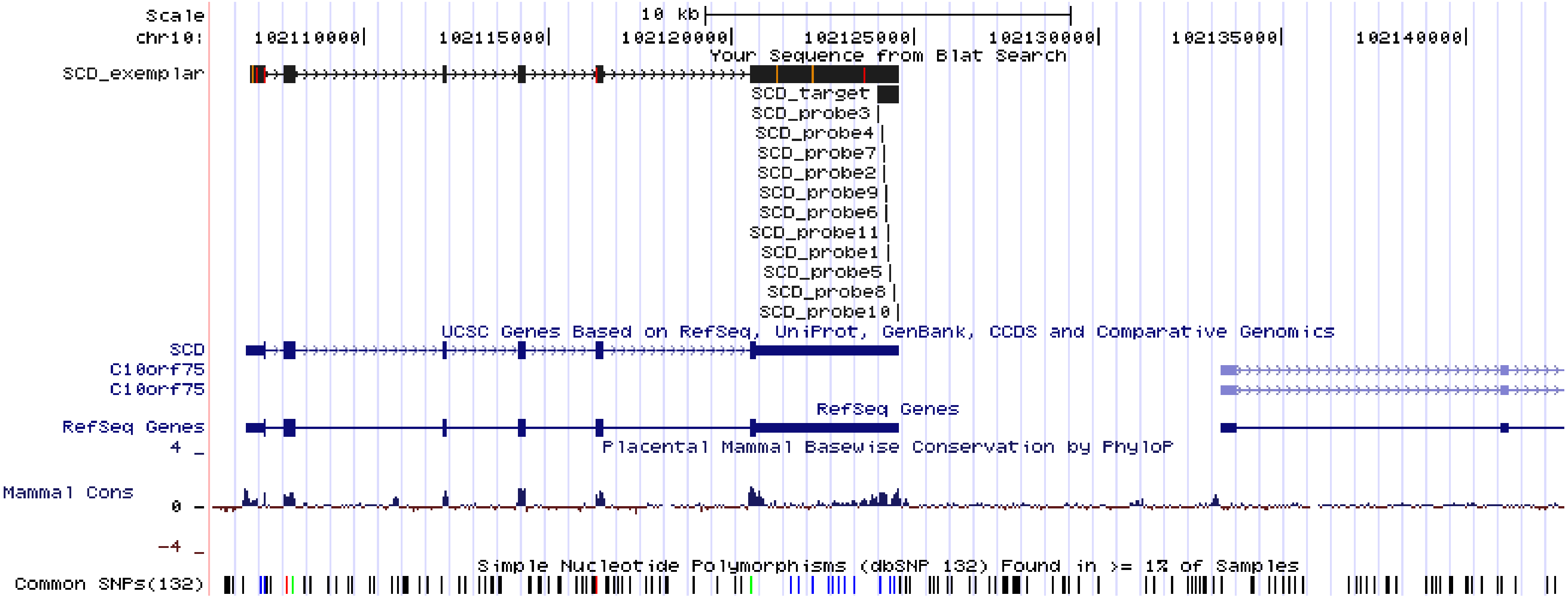

HN1 (51155_at)

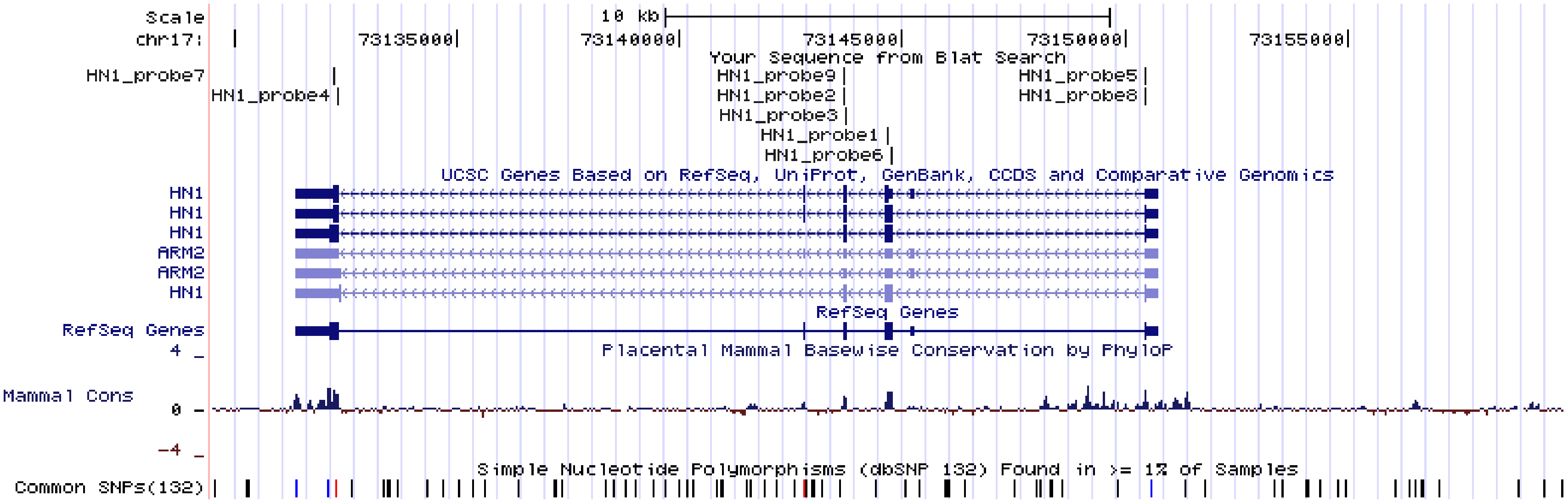
Scale
10 kb
chr17:
73135000
73140000
73145000
73150000
73155000
Your Sequence from Blat Search
HN1_probe7
HN1_probe4
HN1_probe9
HN1_probe2
HN1_probe3
HN1_probe1
HN1_probe6
HN1_probe5
HN1_probe8
UCSC Genes Based on RefSeq, UniProt, GenBank, CCDS and Comparative Genomics
HN1
HN1
HN1
ARM2
ARM2
HN1
RefSeq Genes
RefSeq Genes
Placental Mammal Basewise Conservation by PhyloP
4
Mammal Cons
0
-4
Simple Nucleotide Polymorphisms (dbSNP 132) Found in >= 1% of Samples
Common SNPs(132)

CKS2 (1164_at)

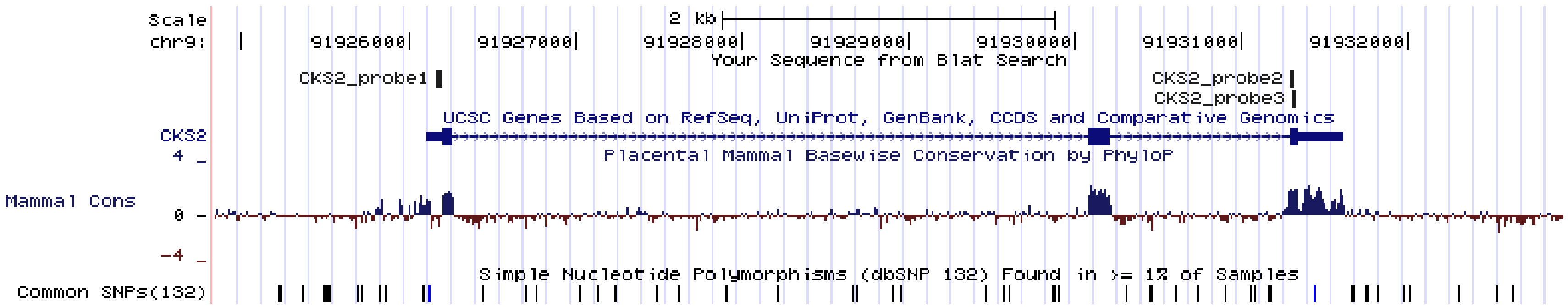

Scale
chr9:
2 kb
91926000
91927000
91928000
91929000
91930000
91931000
91932000
Your Sequence from Blat Search
CKS2_probe1
CKS2_probe2
CKS2_probe3
UCSC Genes Based on RefSeq, UniProt, GenBank, CCDS and Comparative Genomics
CKS2
4
Placental Mammal Basewise Conservation by PhyloP
Mammal Cons
0
-4
Simple Nucleotide Polymorphisms (dbSNP 132) Found in >= 1% of Samples
Common SNPs(132)

NUSAP1 (218039_at)

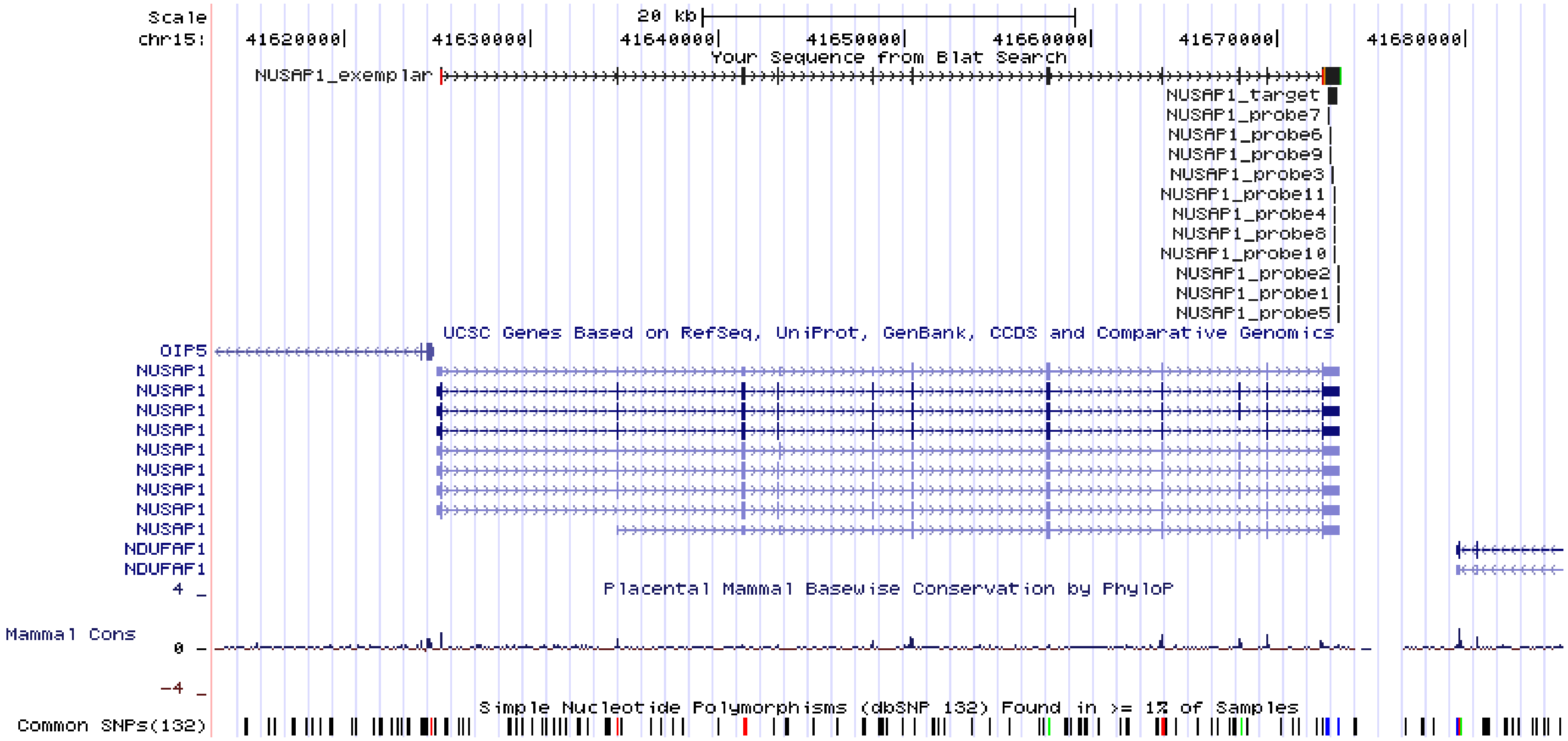

PTTG1 (203554_x_at)

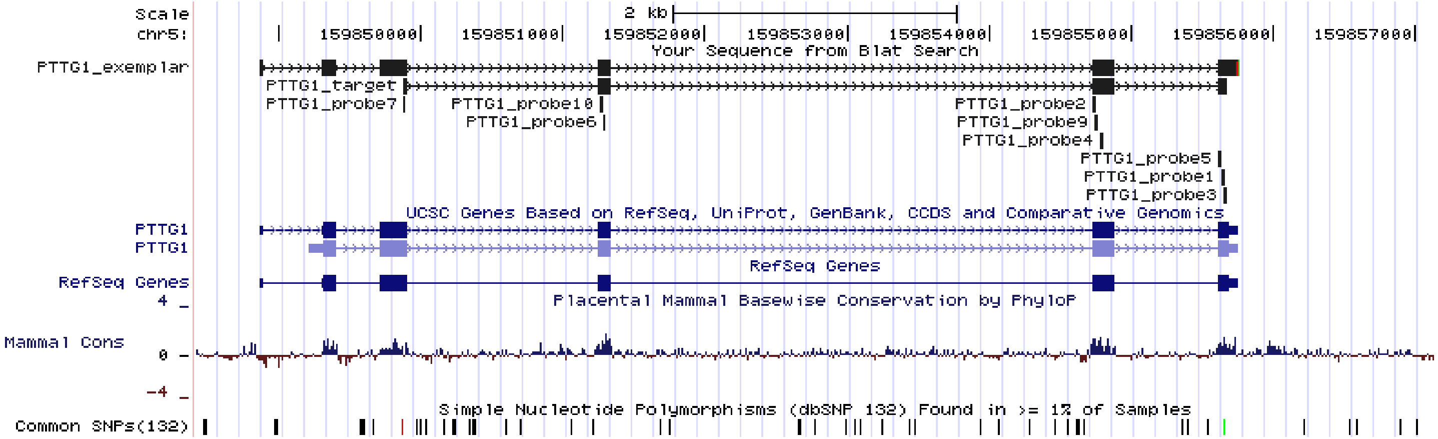

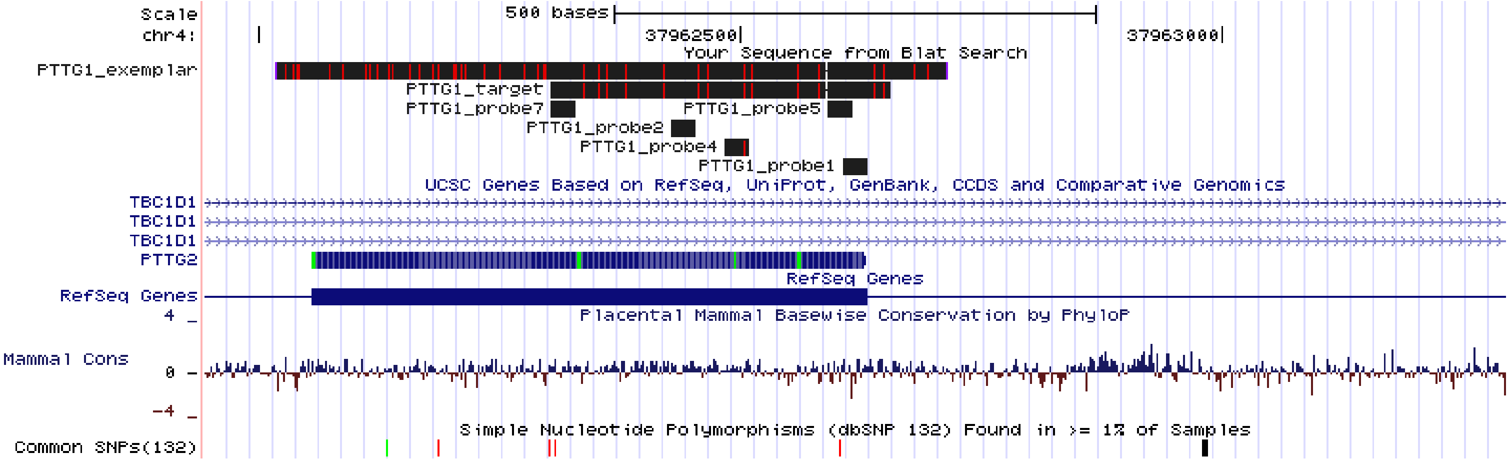

ZWINT (204026_s_at)

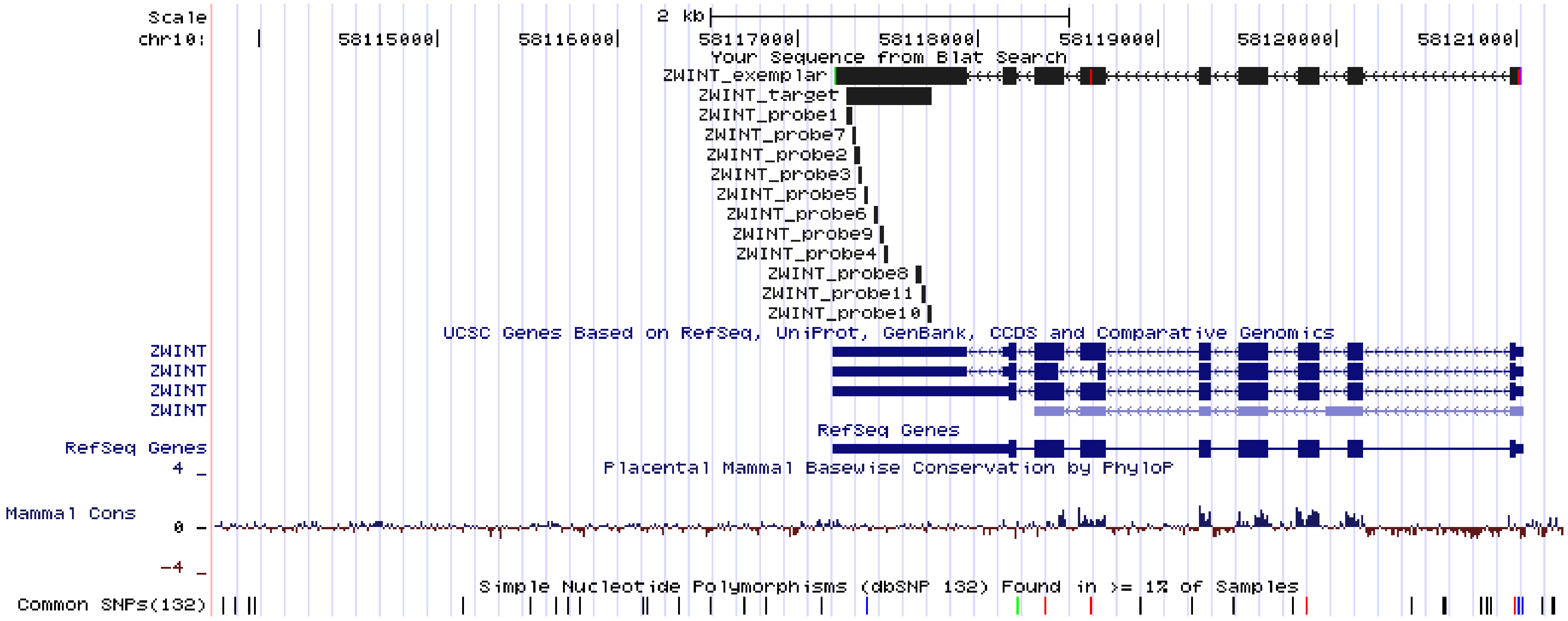

TYMS (7298_at)

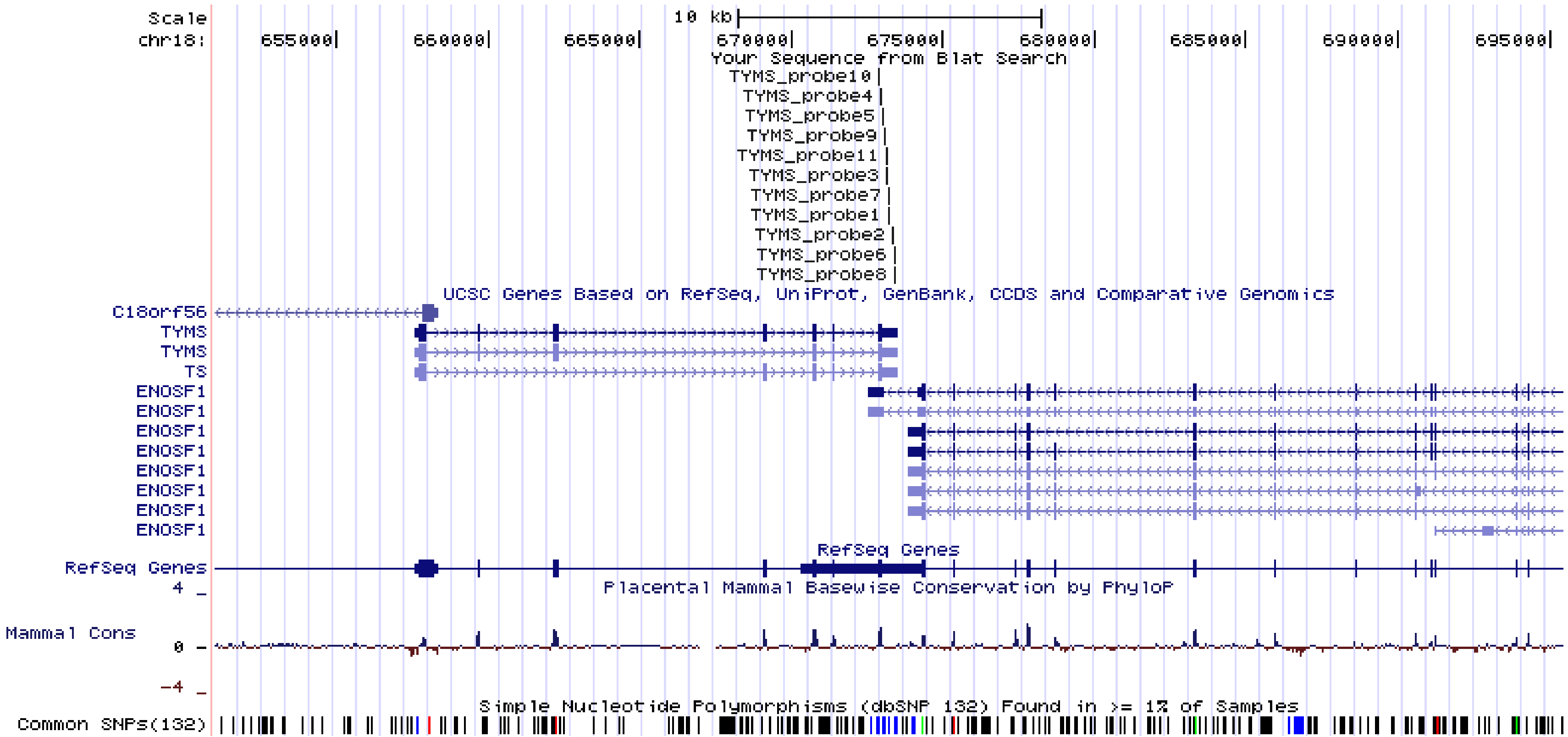
Scale
chr18:
10 kb
655000|
660000|
665000|
670000|
675000|
680000|
685000|
690000|
695000|
Your Sequence from Blat Search
TYMS_probe10
TYMS_probe4
TYMS_probe5
TYMS_probe9
TYMS_probe11
TYMS_probe3
TYMS_probe7
TYMS_probe1
TYMS_probe2
TYMS_probe6
TYMS_probe8
UCSC Genes Based on RefSeq, UniProt, GenBank, CCDS and Comparative Genomics
C18orf56
TYMS
TYMS
TS
ENOSF1
ENOSF1
ENOSF1
ENOSF1
ENOSF1
ENOSF1
ENOSF1
ENOSF1
RefSeq Genes
RefSeq Genes
Placental Mammal Basewise Conservation by PhyloP
4
Mammal Cons
0
-4
Simple Nucleotide Polymorphisms (dbSNP 132) Found in >= 1% of Samples
Common SNPs(132)

MLF1IP (218883_s_at)

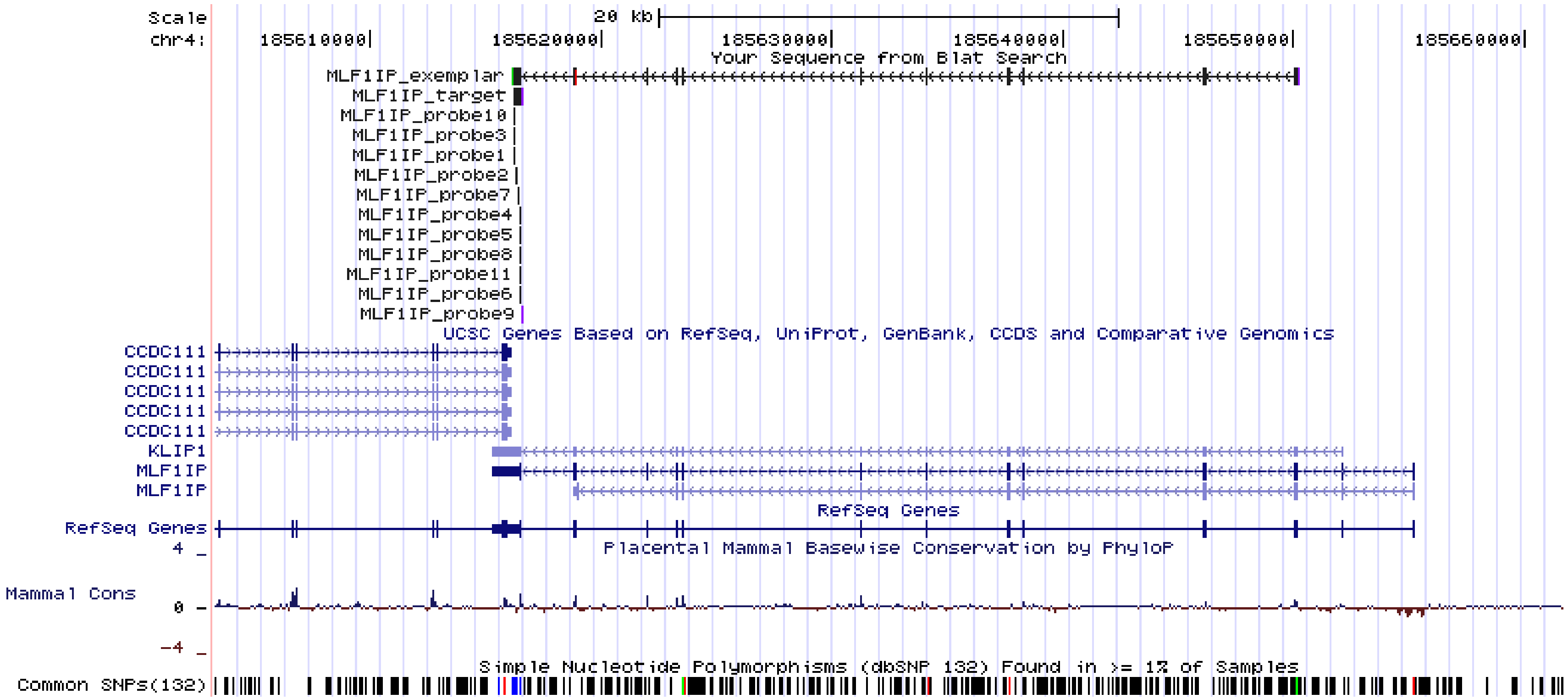

SQLE (209218_at)

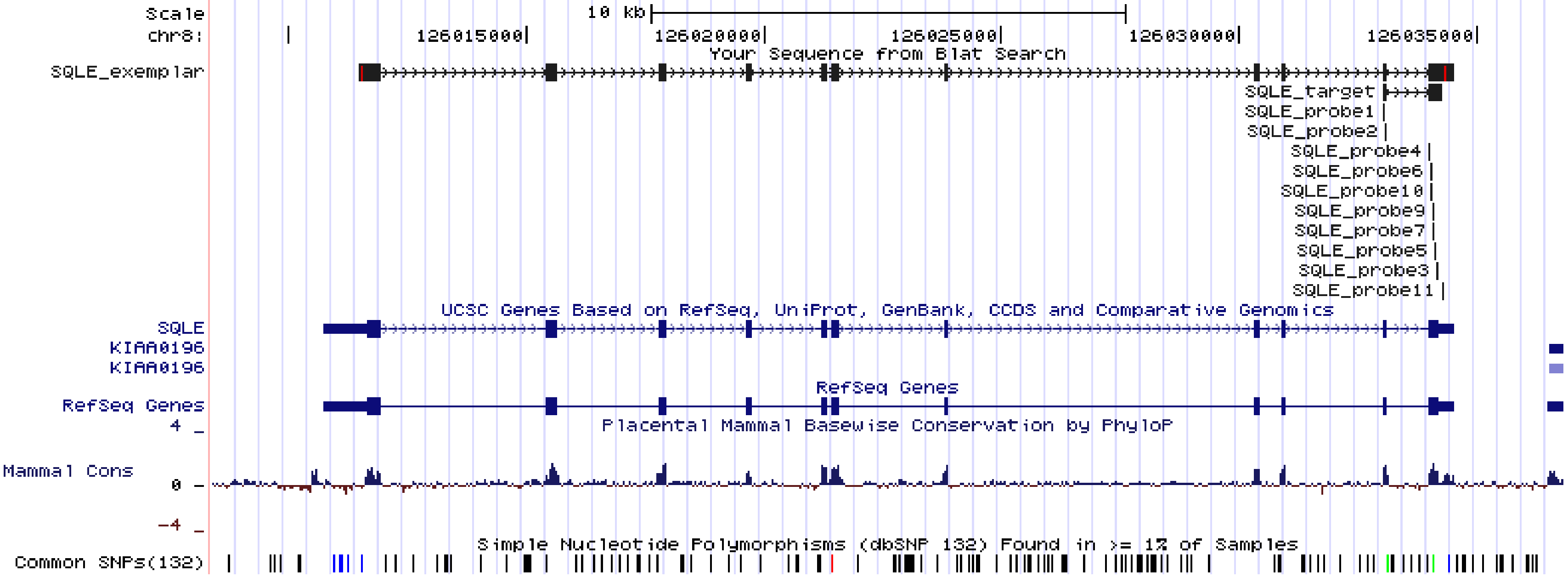

AURKA (208079_s_at)

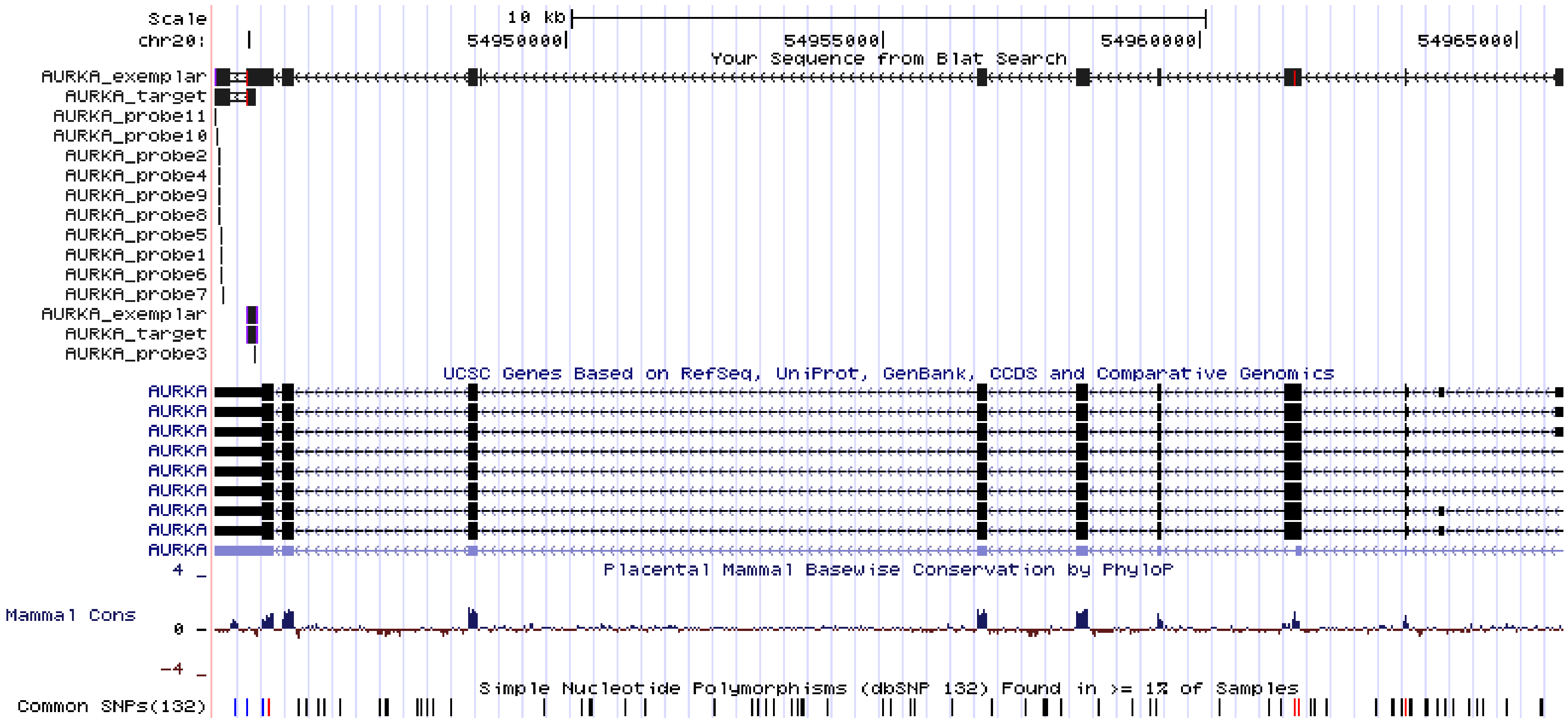

PRC1 (9055_at)

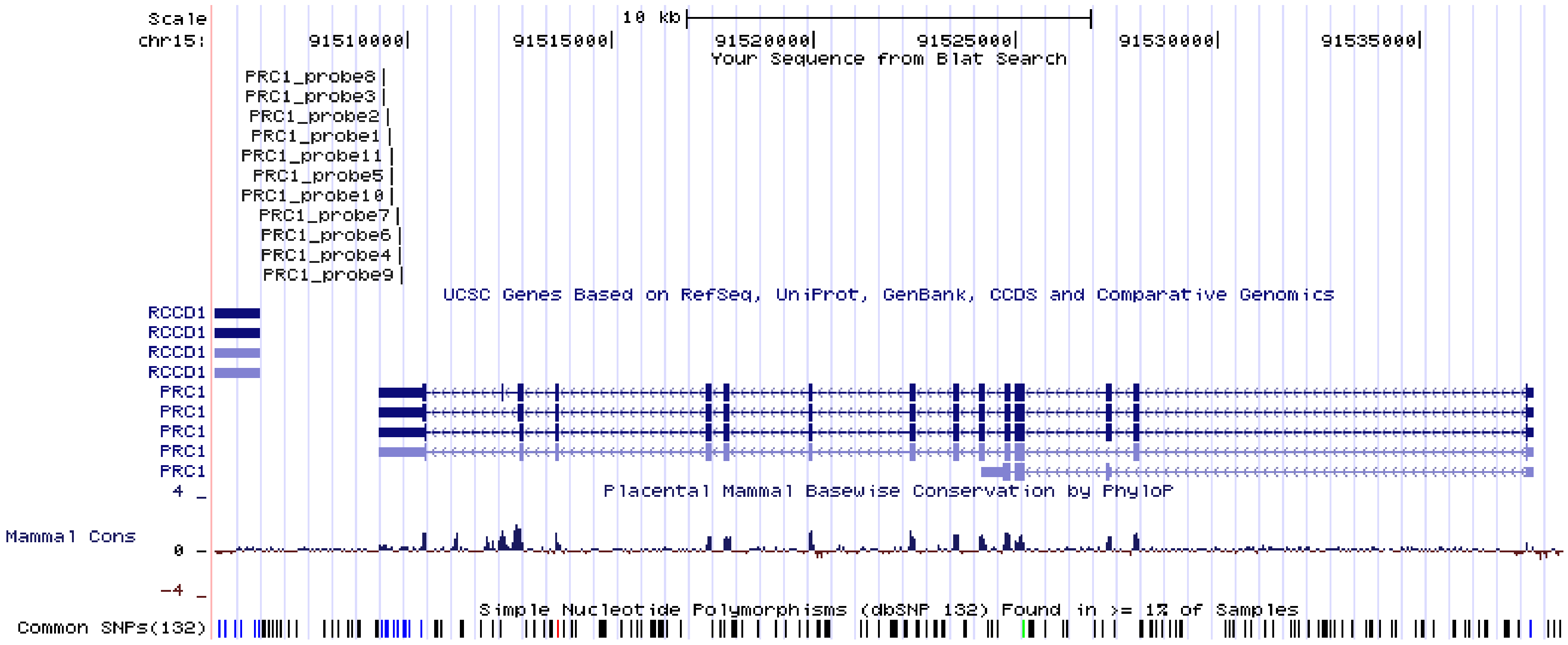
Scale
chr15:
10 kb
91510000
91515000
91520000
91525000
91530000
91535000
Your Sequence from Blat Search
PRC1_probe8
PRC1_probe3
PRC1_probe2
PRC1_probe1
PRC1_probe11
PRC1_probe5
PRC1_probe10
PRC1_probe7
PRC1_probe6
PRC1_probe4
PRC1_probe9
UCSC Genes Based on RefSeq, UniProt, GenBank, CCDS and Comparative Genomics
RCCD1
RCCD1
RCCD1
RCCD1
PRC1
PRC1
PRC1
PRC1
PRC1
4
Placental Mammal Basewise Conservation by PhyloP
Mammal Cons
0
-4
Simple Nucleotide Polymorphisms (dbSNP 132) Found in >= 1% of Samples
Common SNPs(132)

CENPF (207828_s_at)

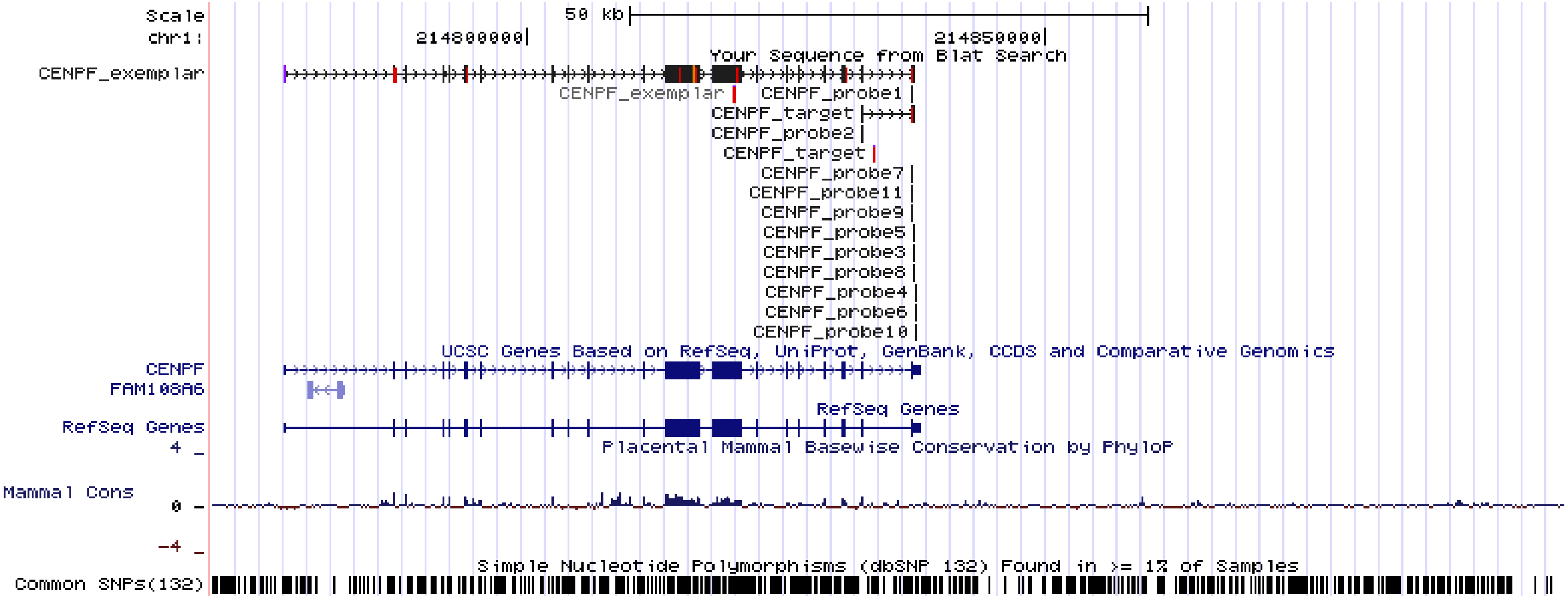

ASPM (219918_s_at)

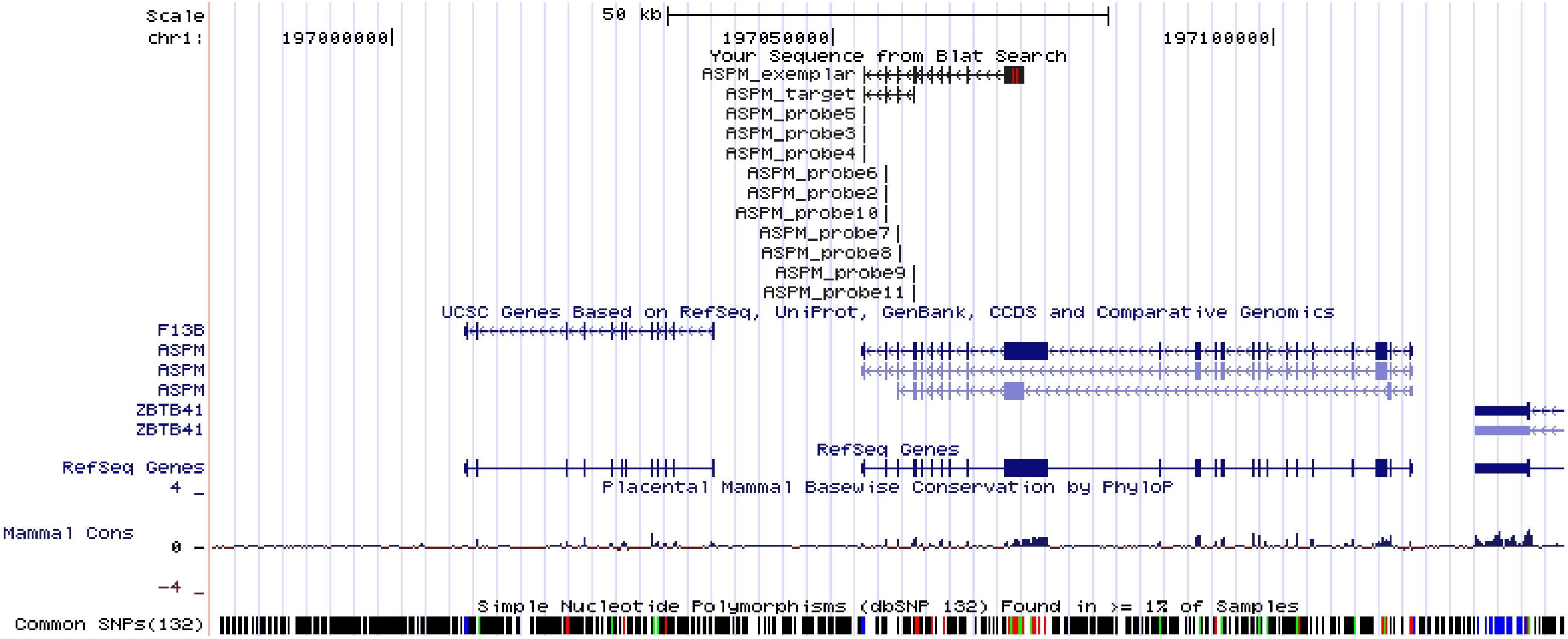

NEK2 (204641_at)

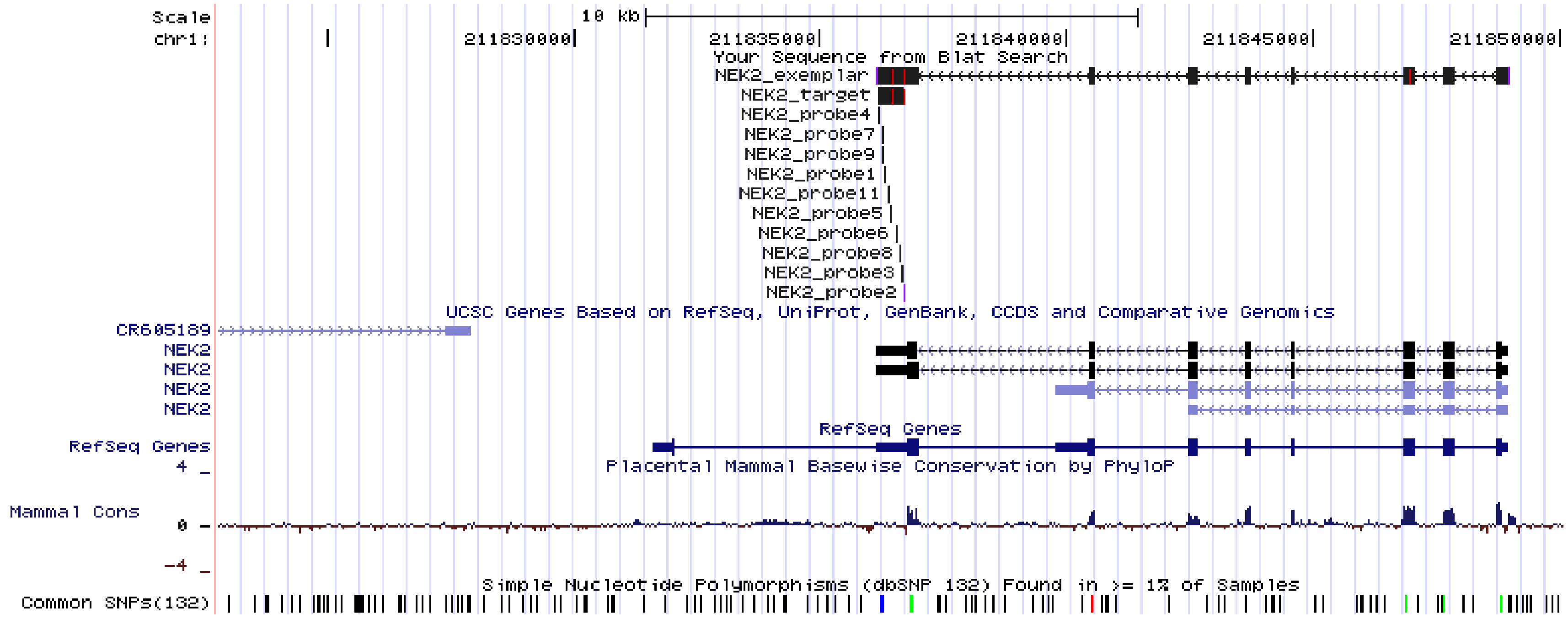

ECT2 (1894_at)

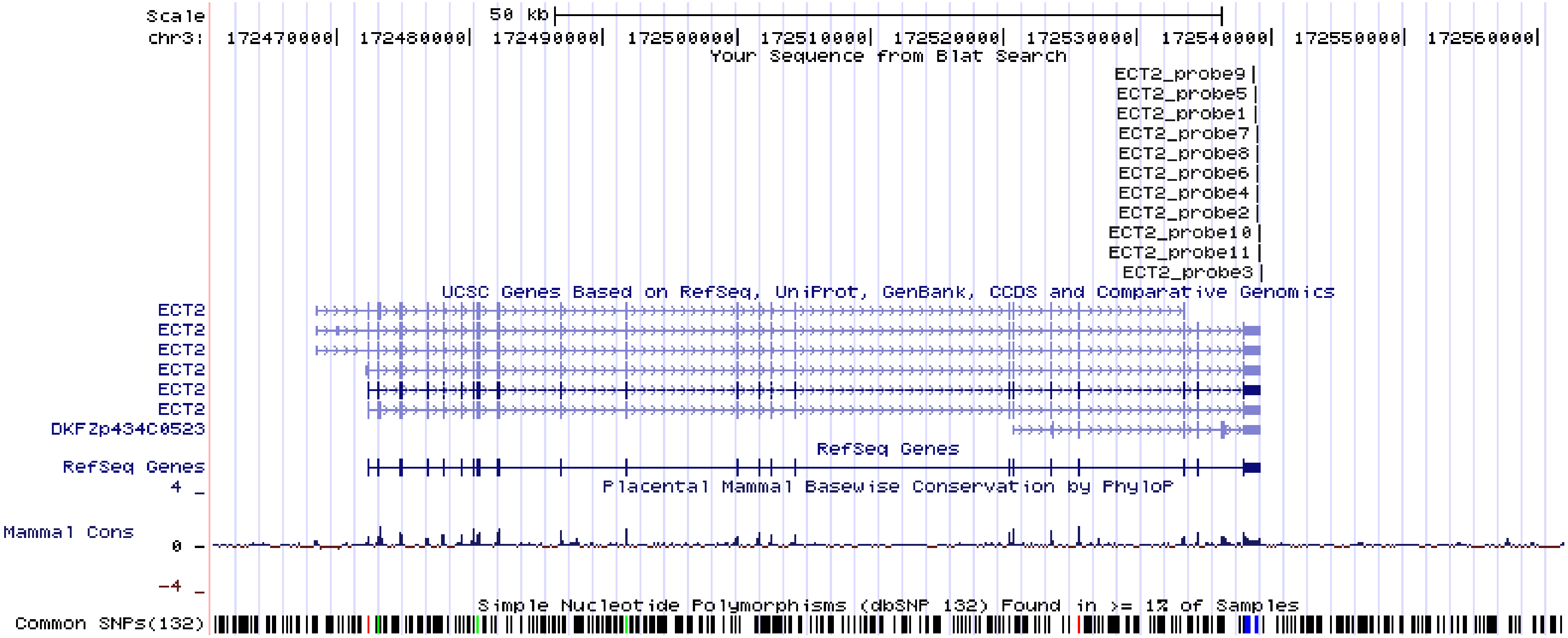

Scale
chr3:
50 kb
172470000
172480000
172490000
172500000
172510000
172520000
172530000
172540000
172550000
172560000
Your Sequence from Blat Search
ECT2_probe9
ECT2_probe5
ECT2_probe1
ECT2_probe7
ECT2_probe8
ECT2_probe6
ECT2_probe4
ECT2_probe2
ECT2_probe10
ECT2_probe11
ECT2_probe3
UCSC Genes Based on RefSeq, UniProt, GenBank, CCDS and Comparative Genomics
ECT2
ECT2
ECT2
ECT2
ECT2
ECT2
DKFZp434C0523
RefSeq Genes
RefSeq Genes
Placental Mammal Basewise Conservation by PhyloP
4
Mammal Cons
0
-4
Simple Nucleotide Polymorphisms (dbSNP 132) Found in >= 1% of Samples
Common SNPs(132)

FEN1 (204767_s_at)

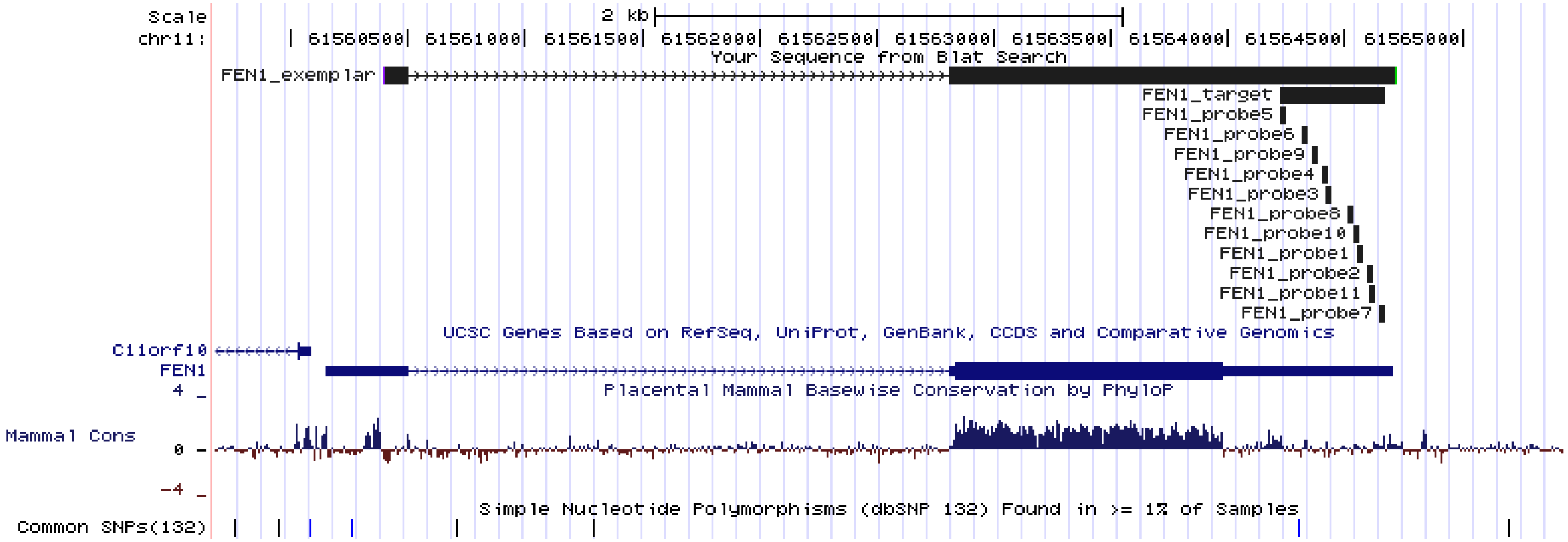

FADD (8772_at)

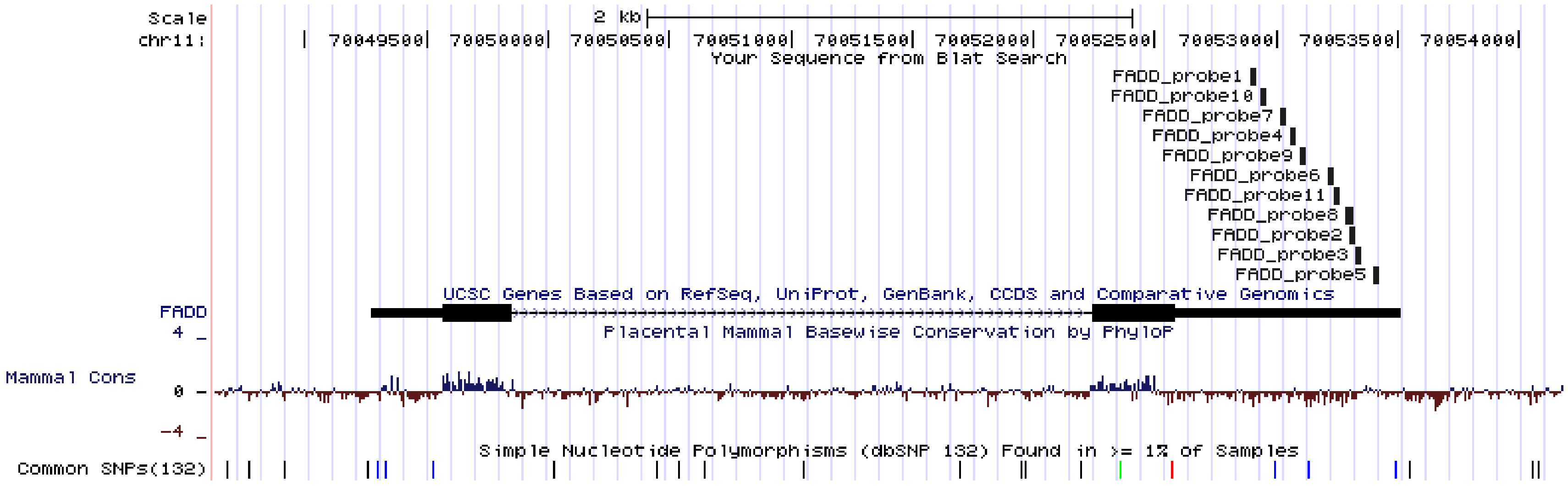
Scale
2 kb
chr11:
70049500
70050000
70050500
70051000
70051500
70052000
70052500
70053000
70053500
70054000
Your Sequence from Blat Search
FADD_probe1
FADD_probe10
FADD_probe7
FADD_probe4
FADD_probe9
FADD_probe6
FADD_probe11
FADD_probe8
FADD_probe2
FADD_probe3
FADD_probe5
UCSC Genes Based on RefSeq, UniProt, GenBank, CCDS and Comparative Genomics
FADD
4
Placental Mammal Basewise Conservation by PhyloP
Mammal Cons
0
-4
Simple Nucleotide Polymorphisms (dbSNP 132) Found in >= 1% of Samples
Common SNPs(132)

SMC4 (10051_at)

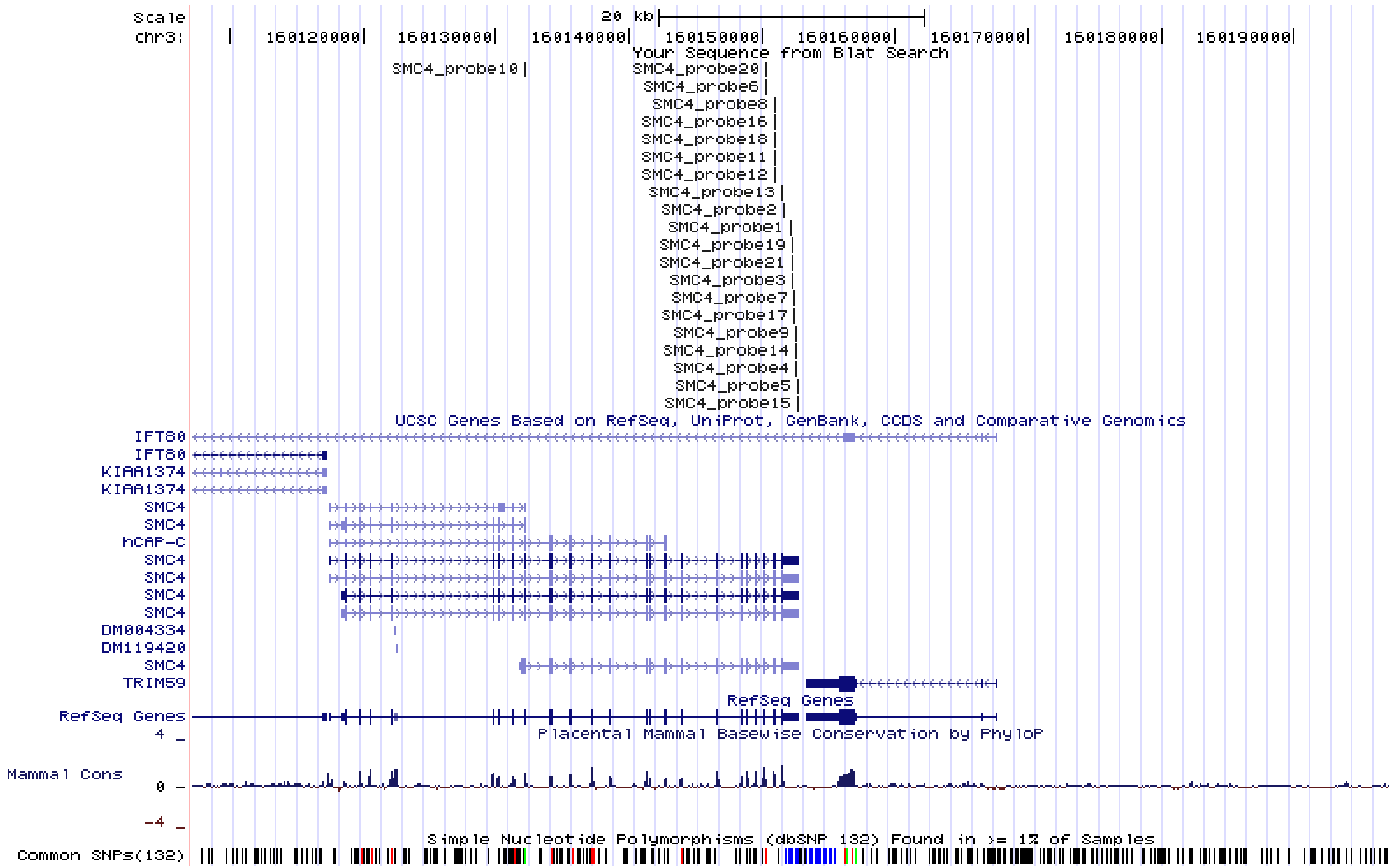

SLC35E3 (55508_at)

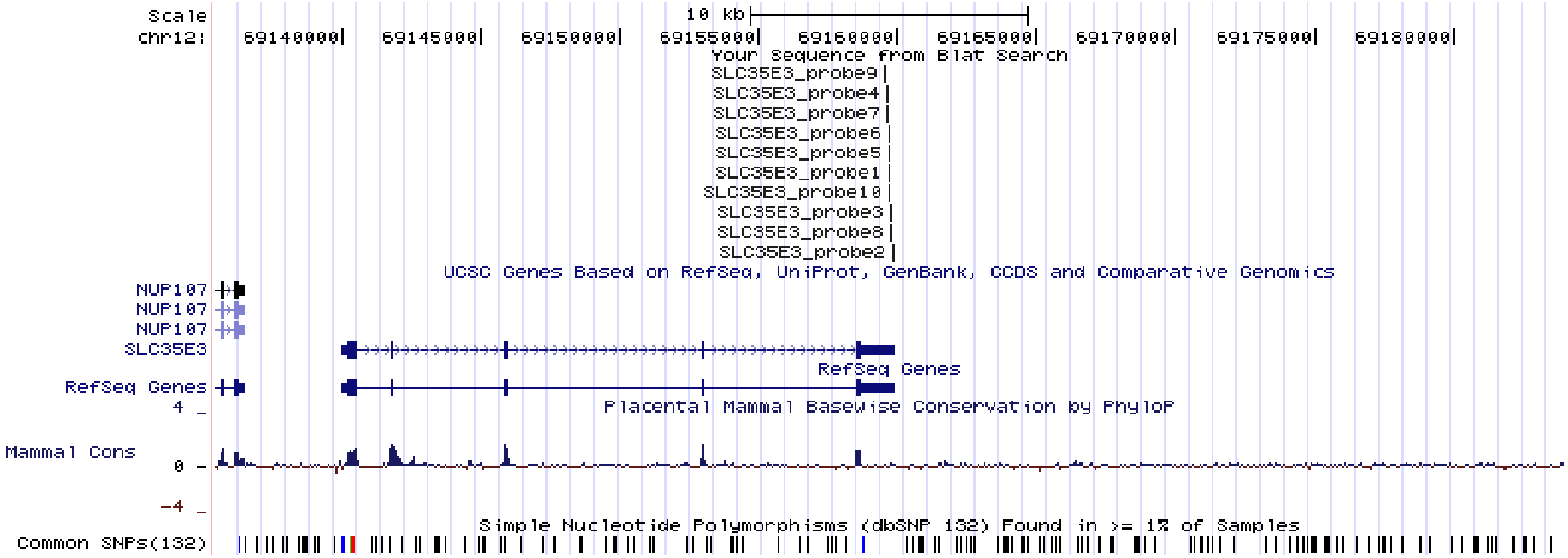

TXNRD1 (7296_at)

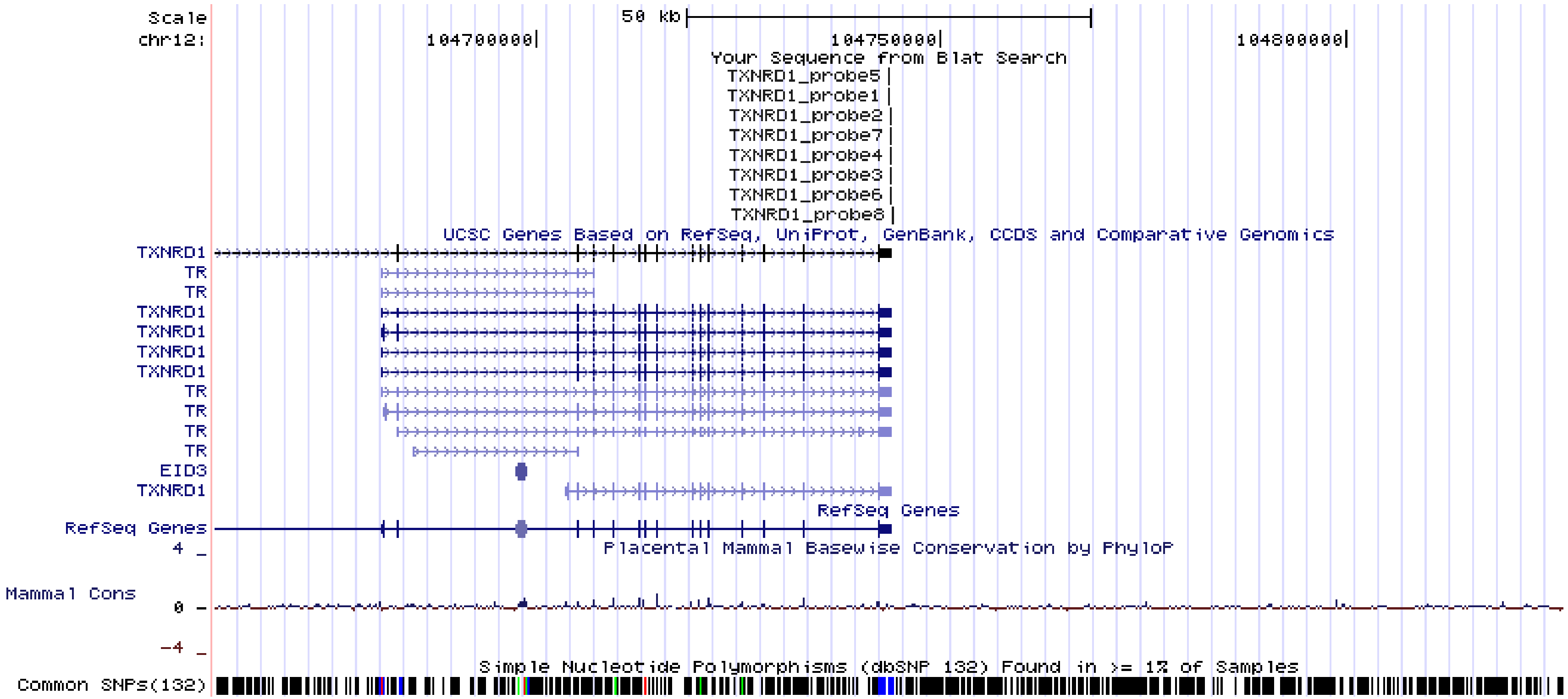

RAE1 (211318_s_at)

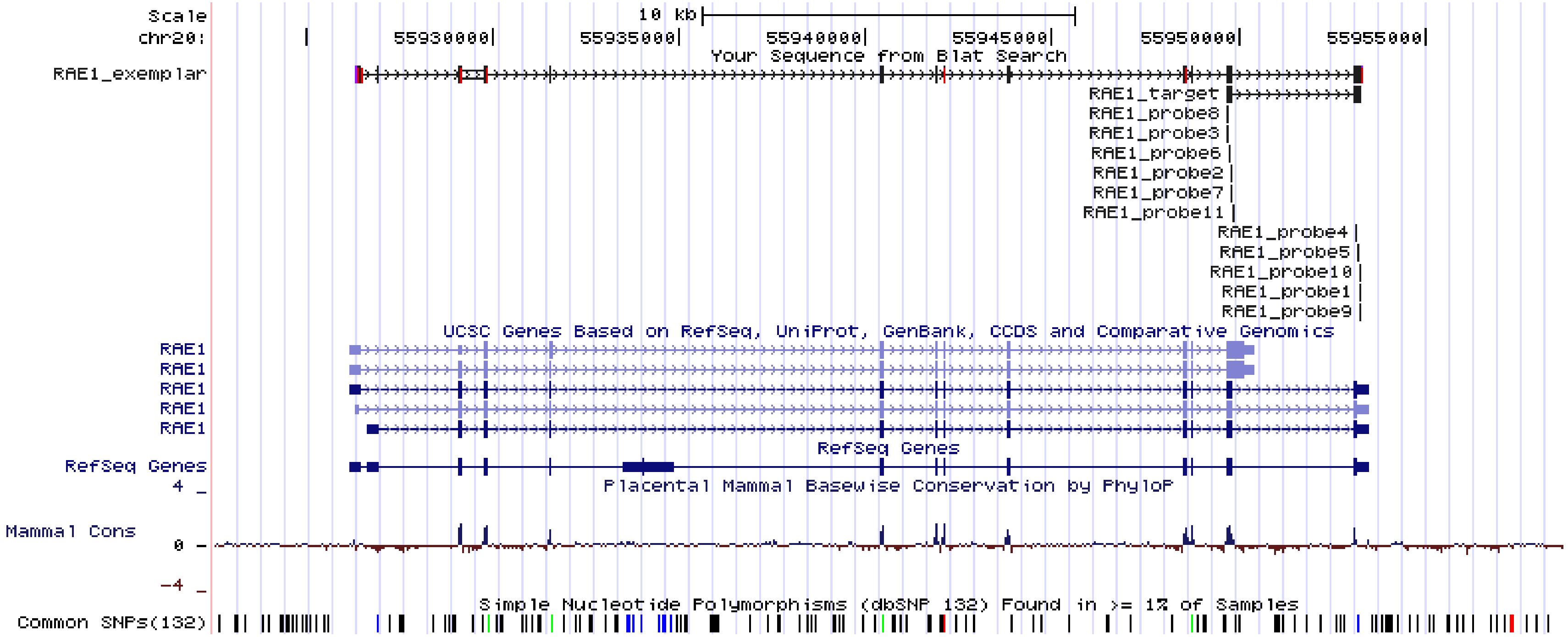

ACBD3 (202323_s_at)

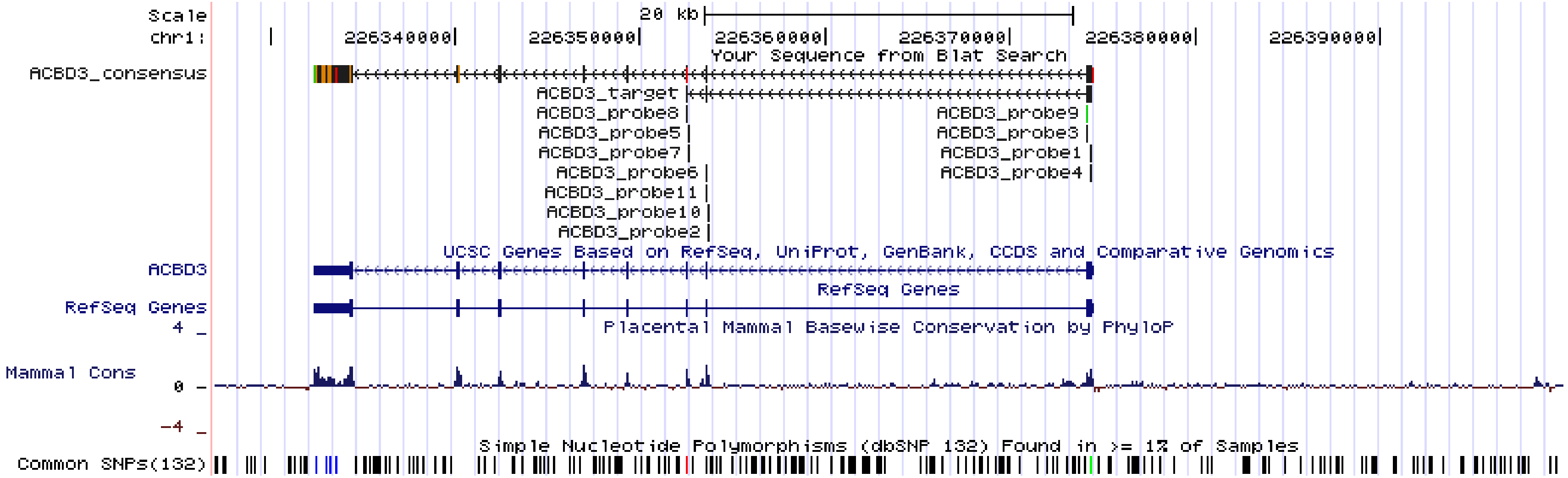

ZNF274 (204937_s_at)

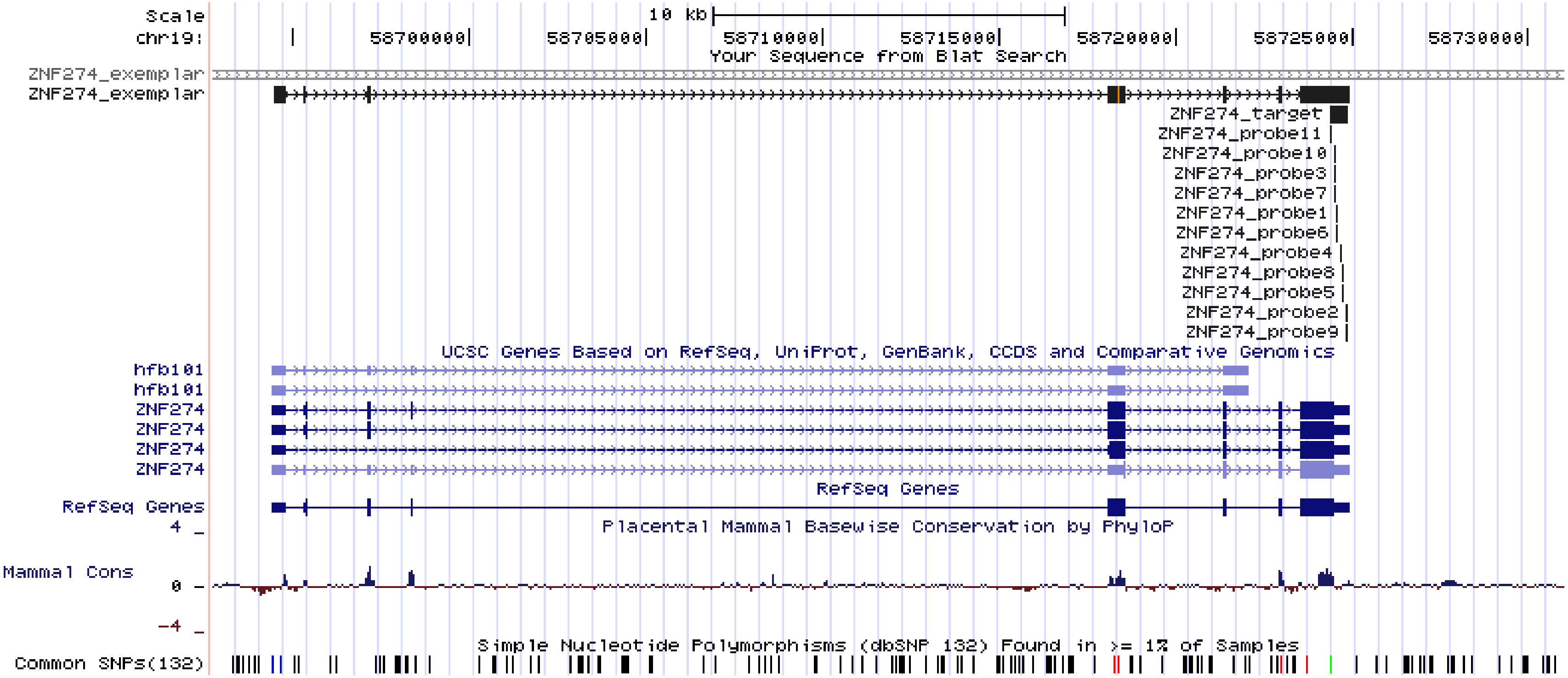

FRG1 (2483_at)

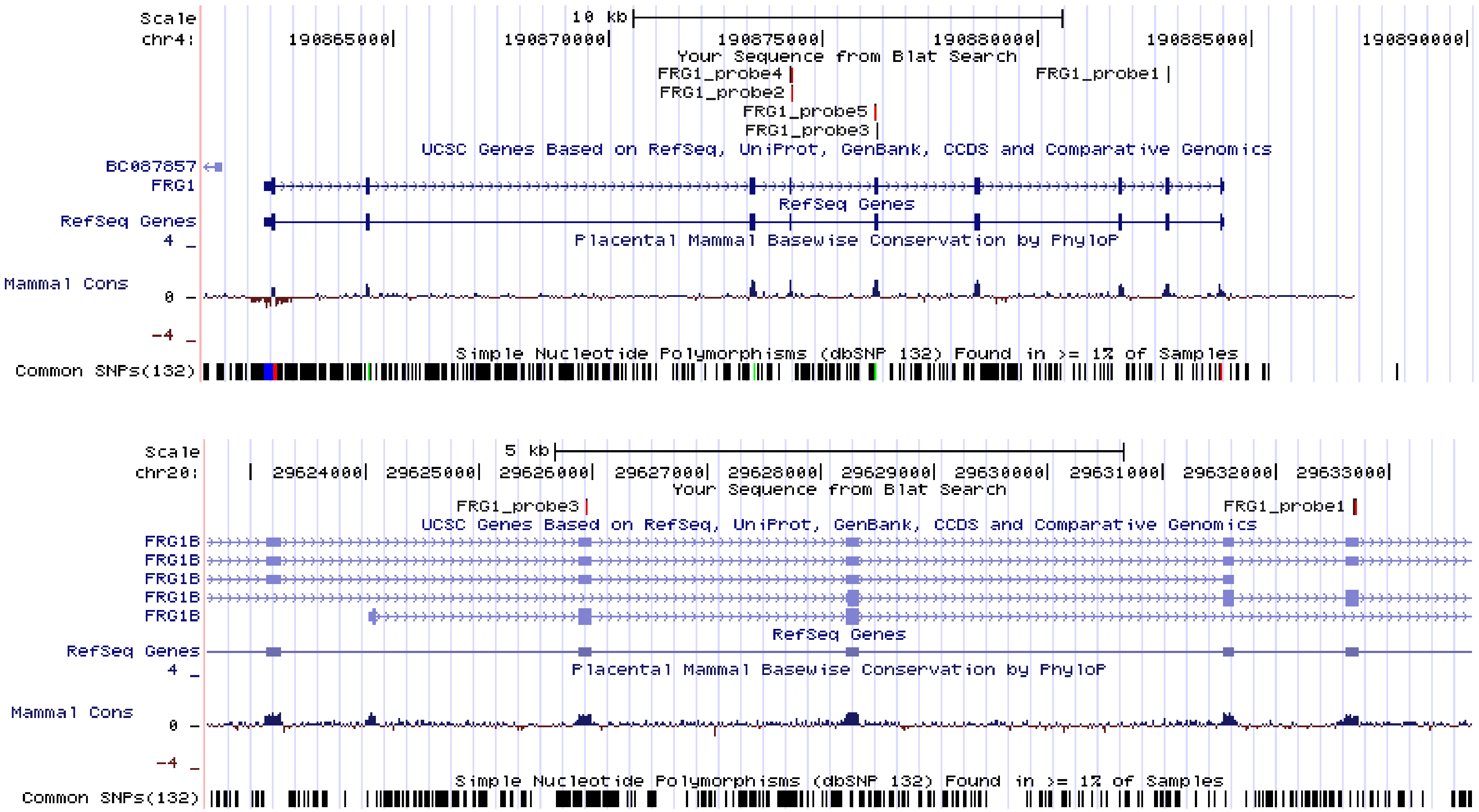
Scale
chr4:
10 kb
190865000
190870000
190875000
190880000
190885000
190890000
Your Sequence from Blat Search
FRG1_probe4
FRG1_probe2
FRG1_probe5
FRG1_probe3
FRG1_probe1
UCSC Genes Based on RefSeq, UniProt, GenBank, CCDS and Comparative Genomics
BC087857
FRG1
RefSeq Genes
RefSeq Genes
4
Placental Mammal Basewise Conservation by PhyloP
Mammal Cons
0
-4
Simple Nucleotide Polymorphisms (dbSNP 132) Found in >= 1% of Samples
Common SNPs(132)
Scale
chr20:
5 kb
29624000
29625000
29626000
29627000
29628000
29629000
29630000
29631000
29632000
29633000
Your Sequence from Blat Search
FRG1_probe3
FRG1_probe1
UCSC Genes Based on RefSeq, UniProt, GenBank, CCDS and Comparative Genomics
FRG1B
FRG1B
FRG1B
FRG1B
FRG1B
RefSeq Genes
RefSeq Genes
4
Placental Mammal Basewise Conservation by PhyloP
Mammal Cons
0
-4
Simple Nucleotide Polymorphisms (dbSNP 132) Found in >= 1% of Samples
Common SNPs(132)

LPCAT1 (201818_at)

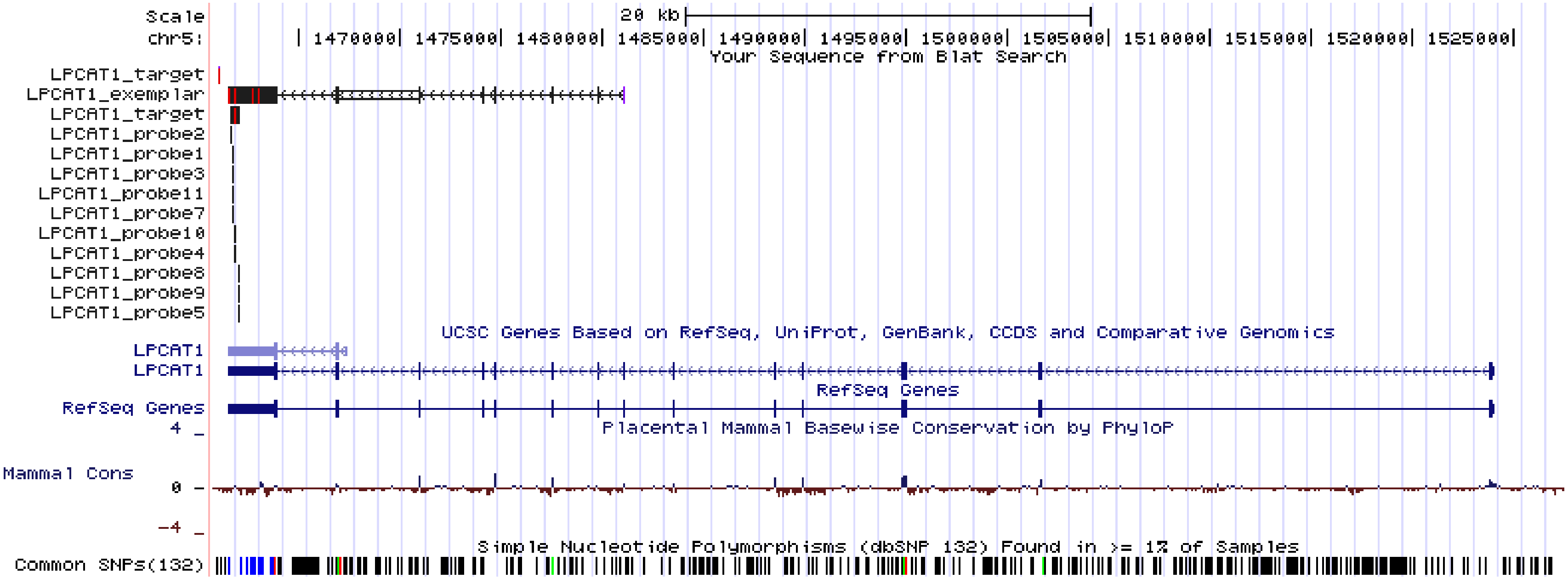

EBP (10682_at)

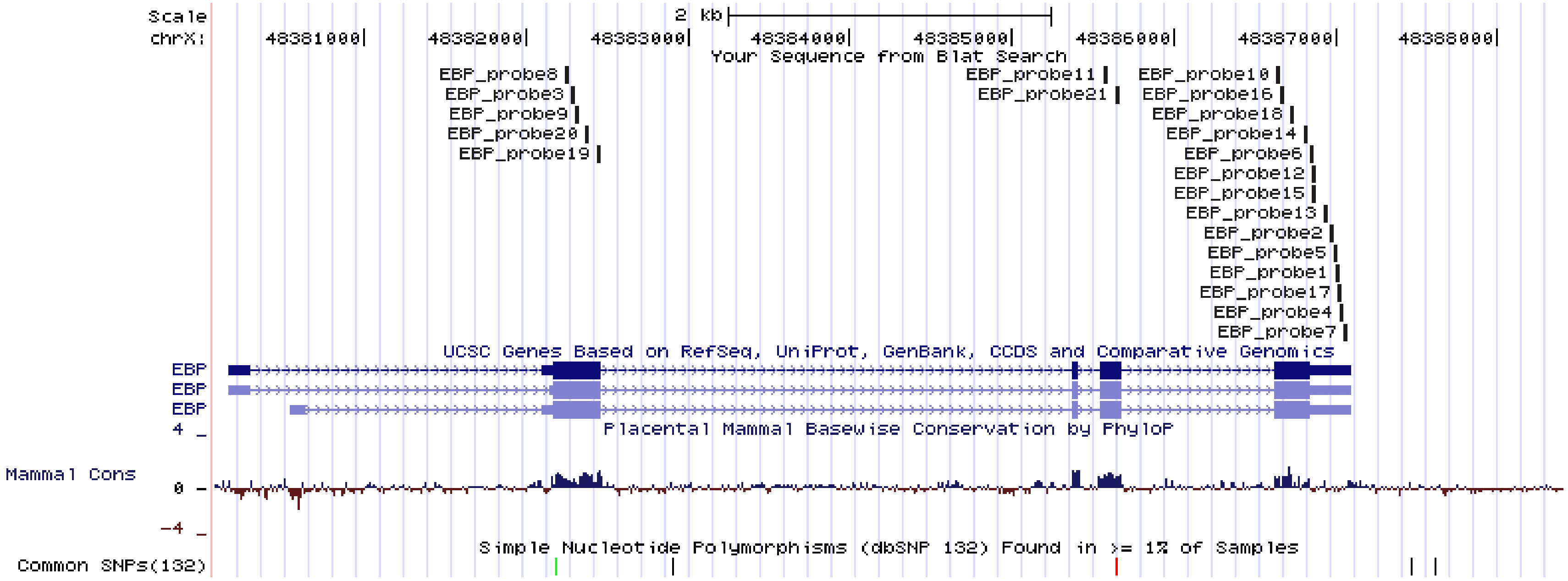
Scale
chrX:
2 kb
48381000
48382000
48383000
48384000
48385000
48386000
48387000
48388000
Your Sequence from Blat Search
EBP_probe8
EBP_probe3
EBP_probe9
EBP_probe20
EBP_probe19
EBP_probe11
EBP_probe21
EBP_probe10
EBP_probe16
EBP_probe18
EBP_probe14
EBP_probe6
EBP_probe12
EBP_probe15
EBP_probe13
EBP_probe2
EBP_probe5
EBP_probe1
EBP_probe17
EBP_probe4
EBP_probe7
UCSC Genes Based on RefSeq, UniProt, GenBank, CCDS and Comparative Genomics
EBP
EBP
EBP
4
Placental Mammal Basewise Conservation by PhyloP
Mammal Cons
0
-4
Simple Nucleotide Polymorphisms (dbSNP 132) Found in >= 1% of Samples
Common SNPs(132)

RFC4 (204023_at)

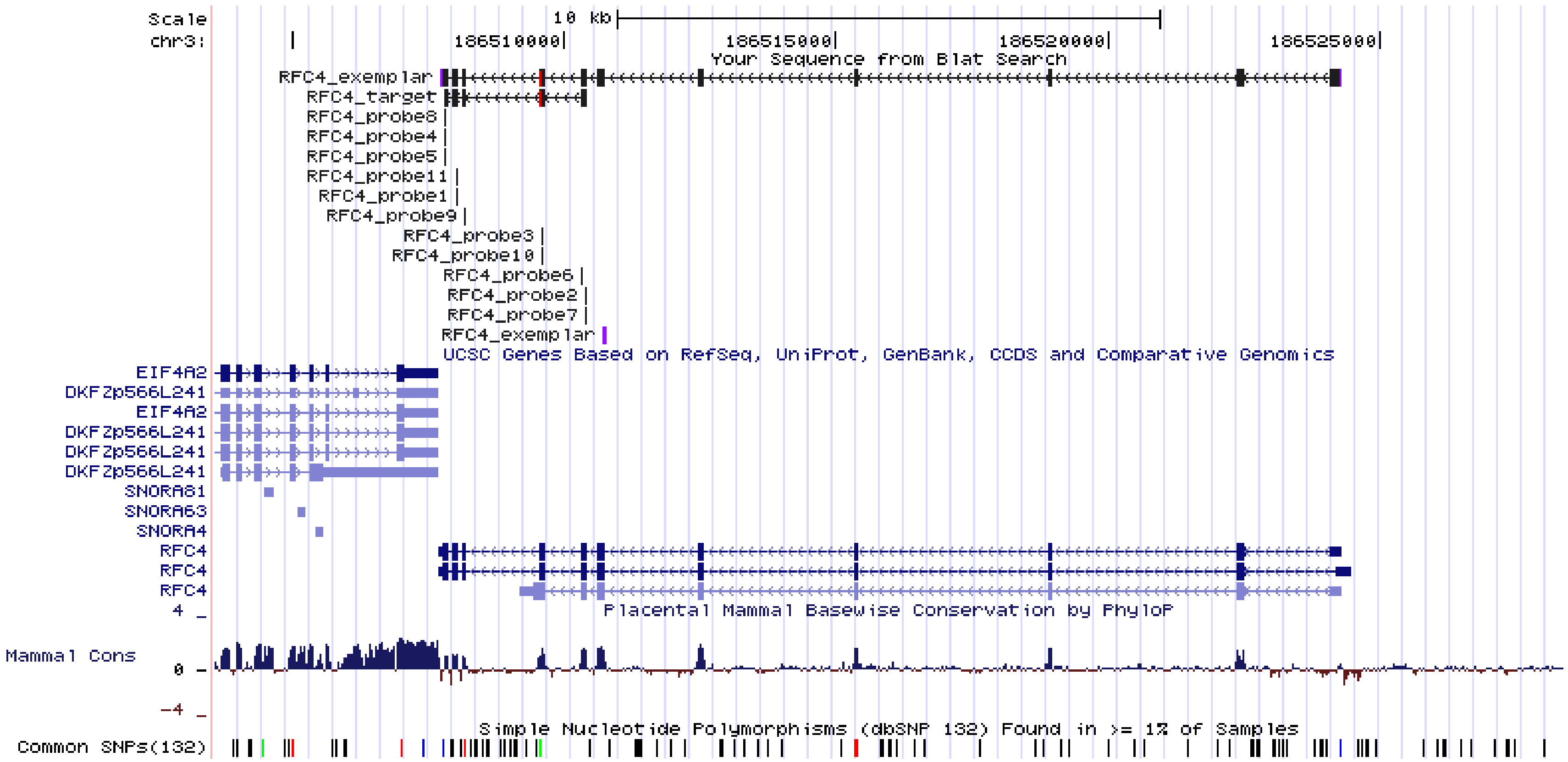

NCAPG (218662_s_at)

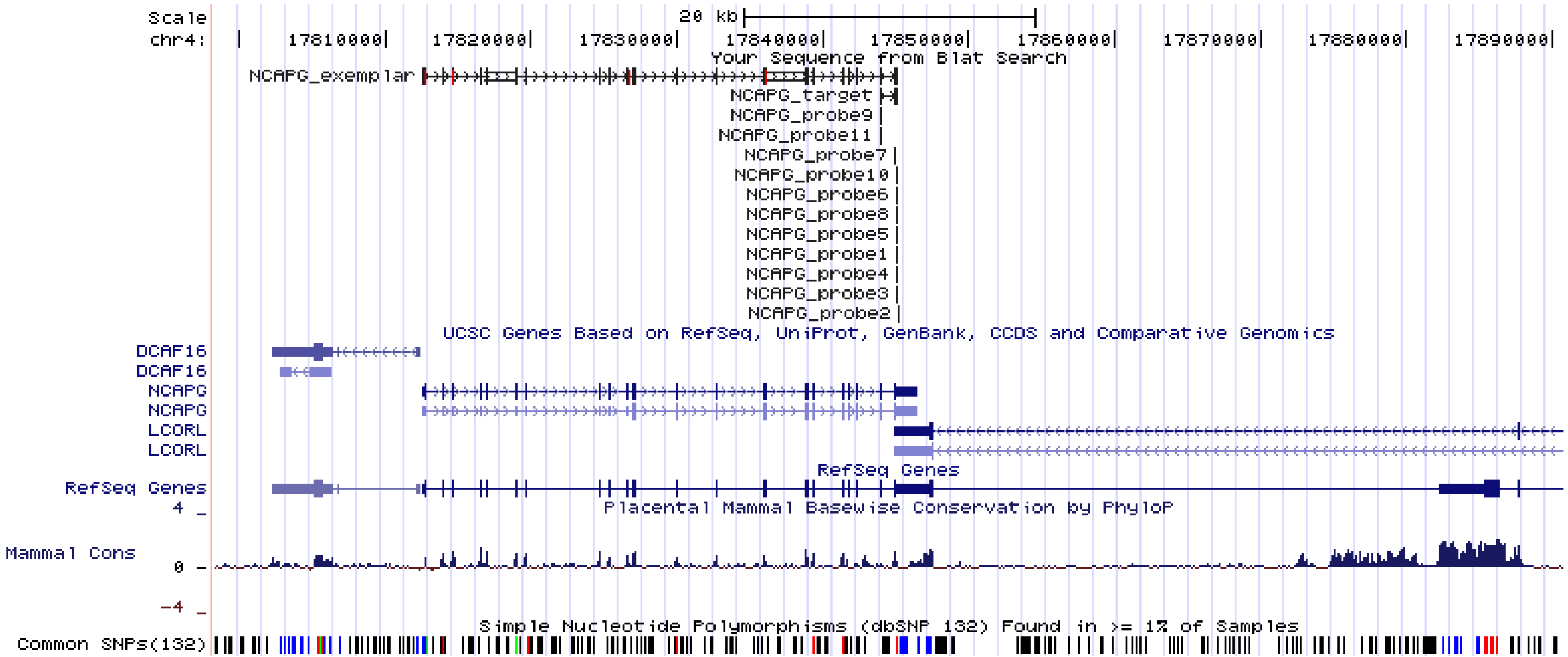

RNASEH2A (10535_at)

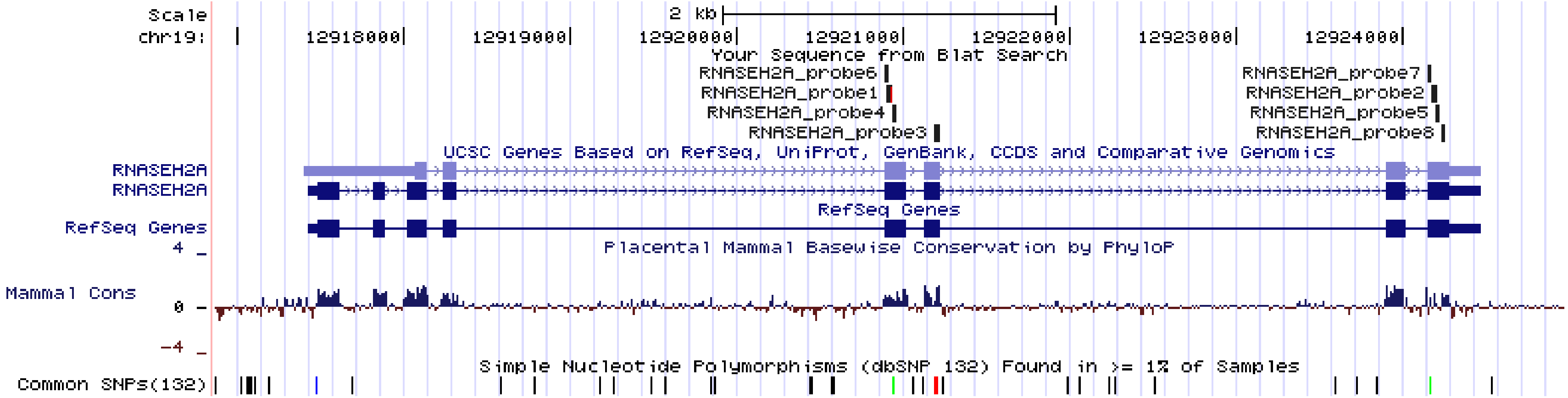

MED24 (9862_at)

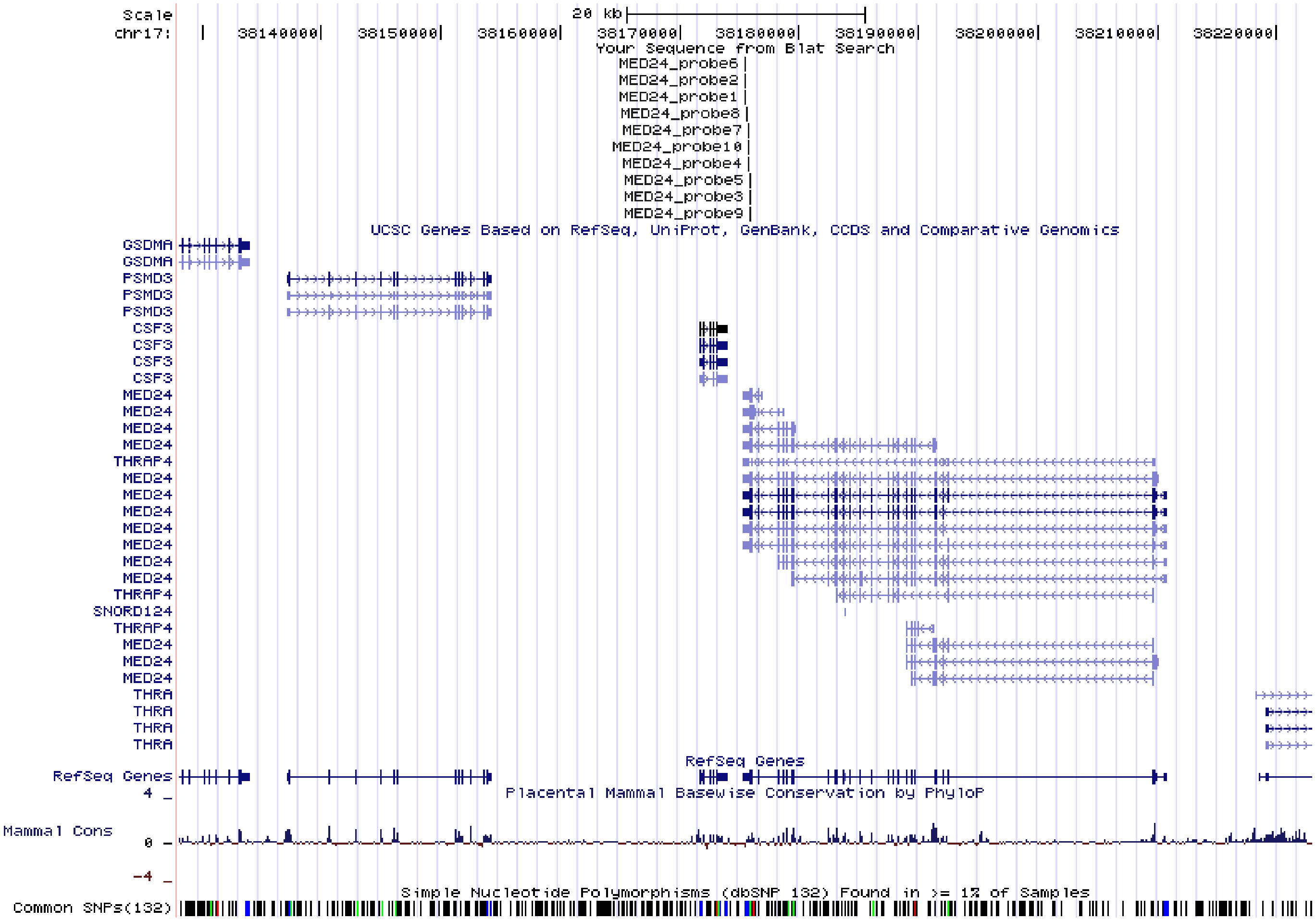

DONSON (29980_at)

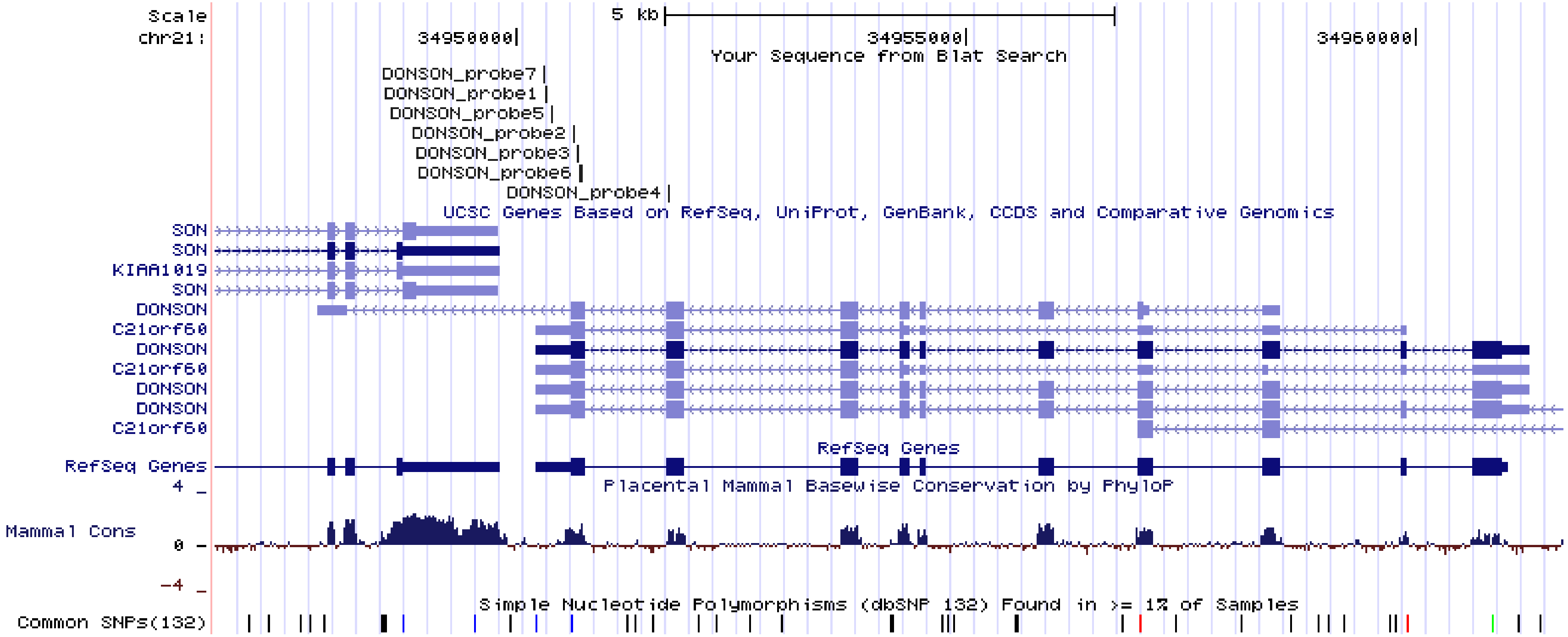

RMI1 (80010_at)

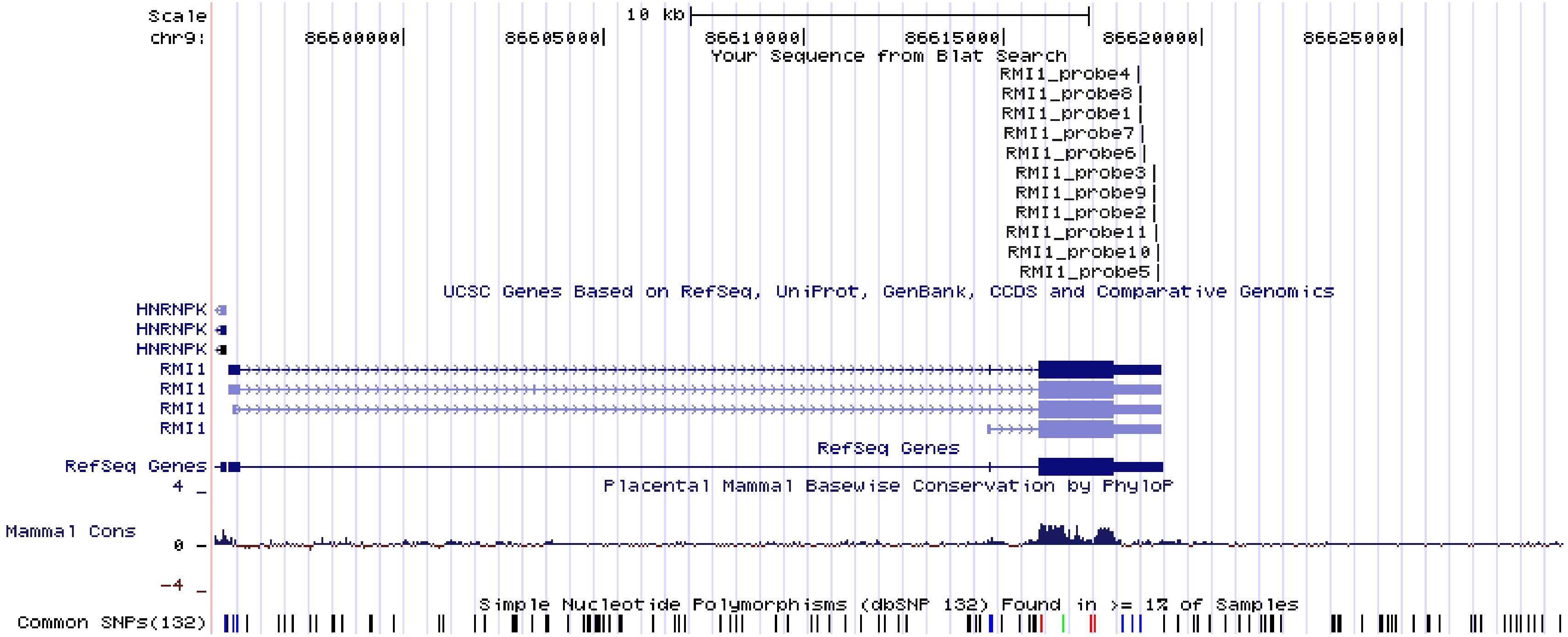

PTGES (9536_at)

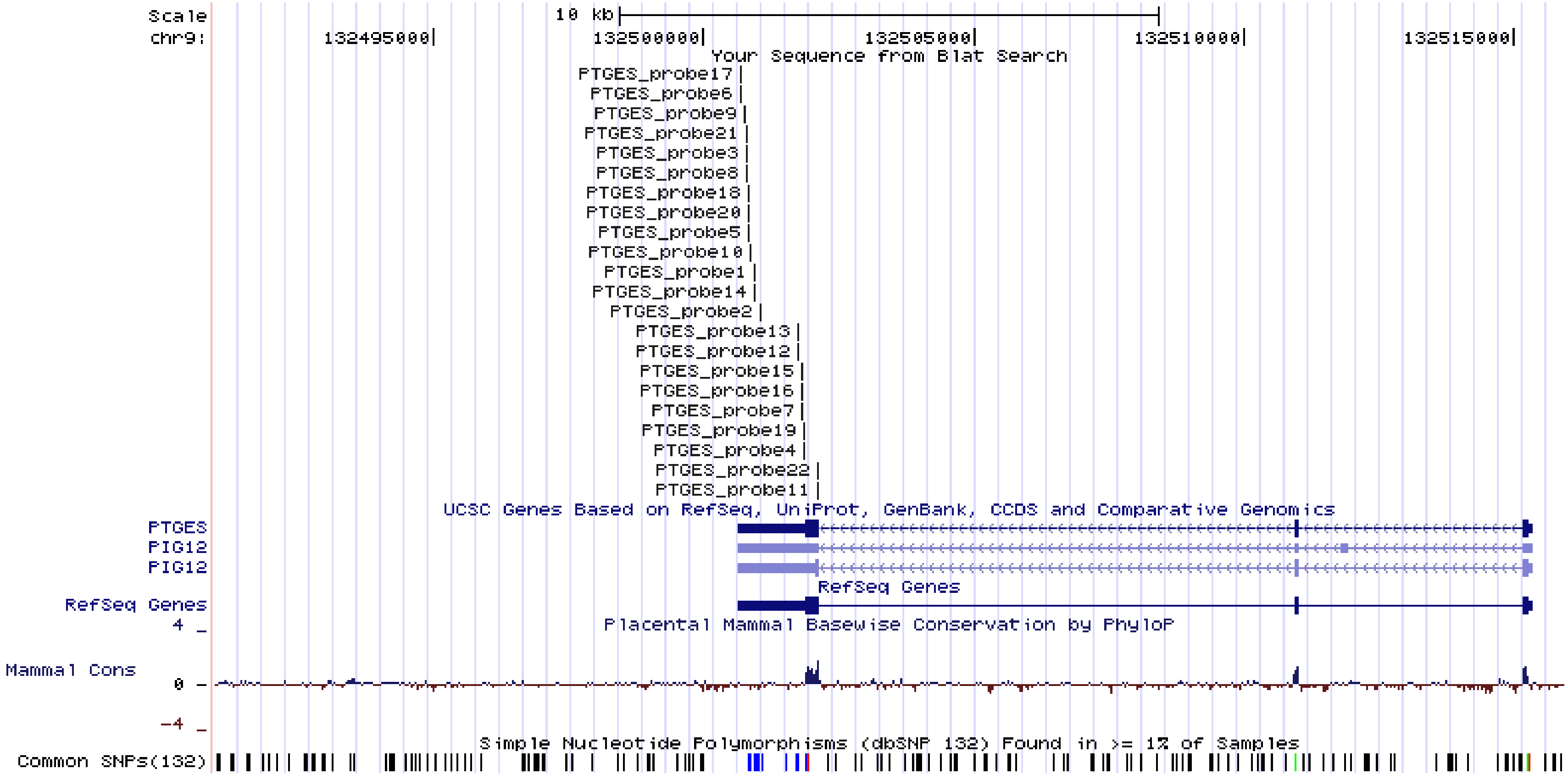

C19orf60 (51200_at)

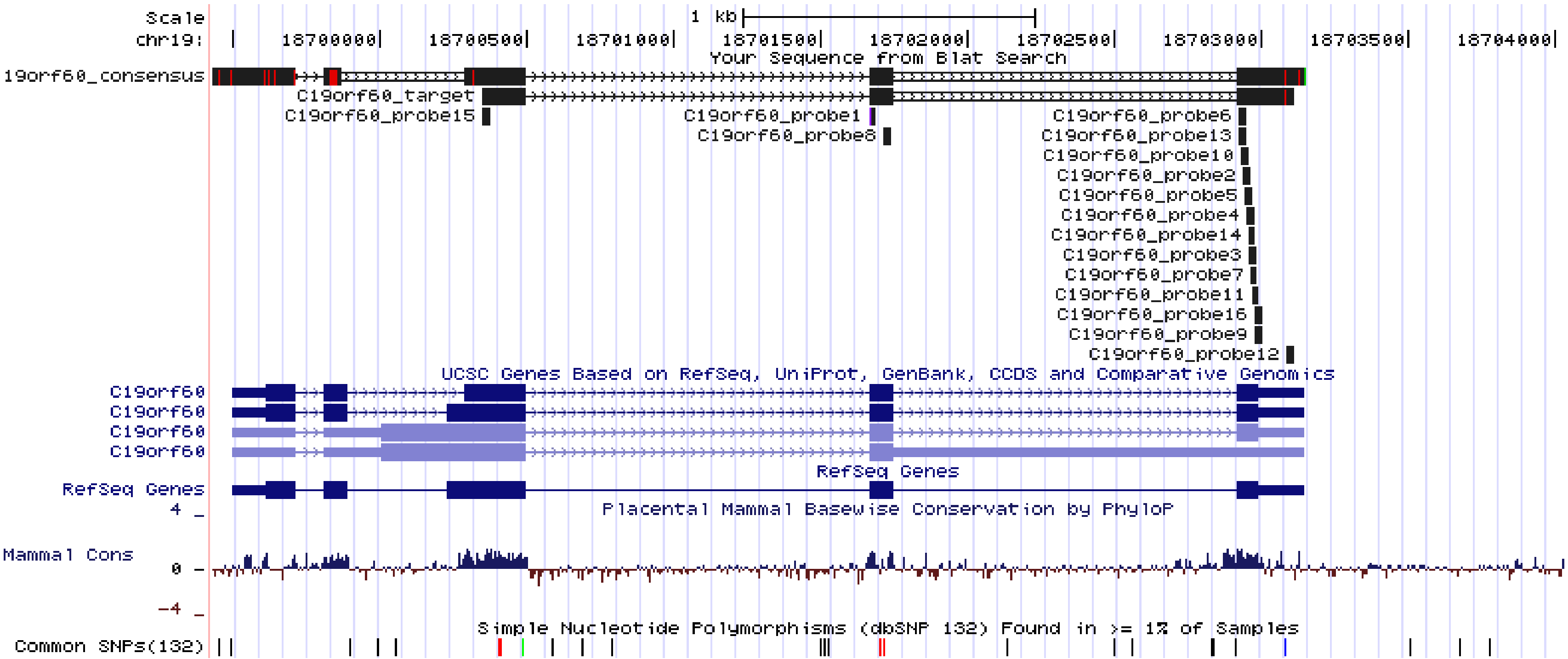

ISYNA1 (222240_s_at)

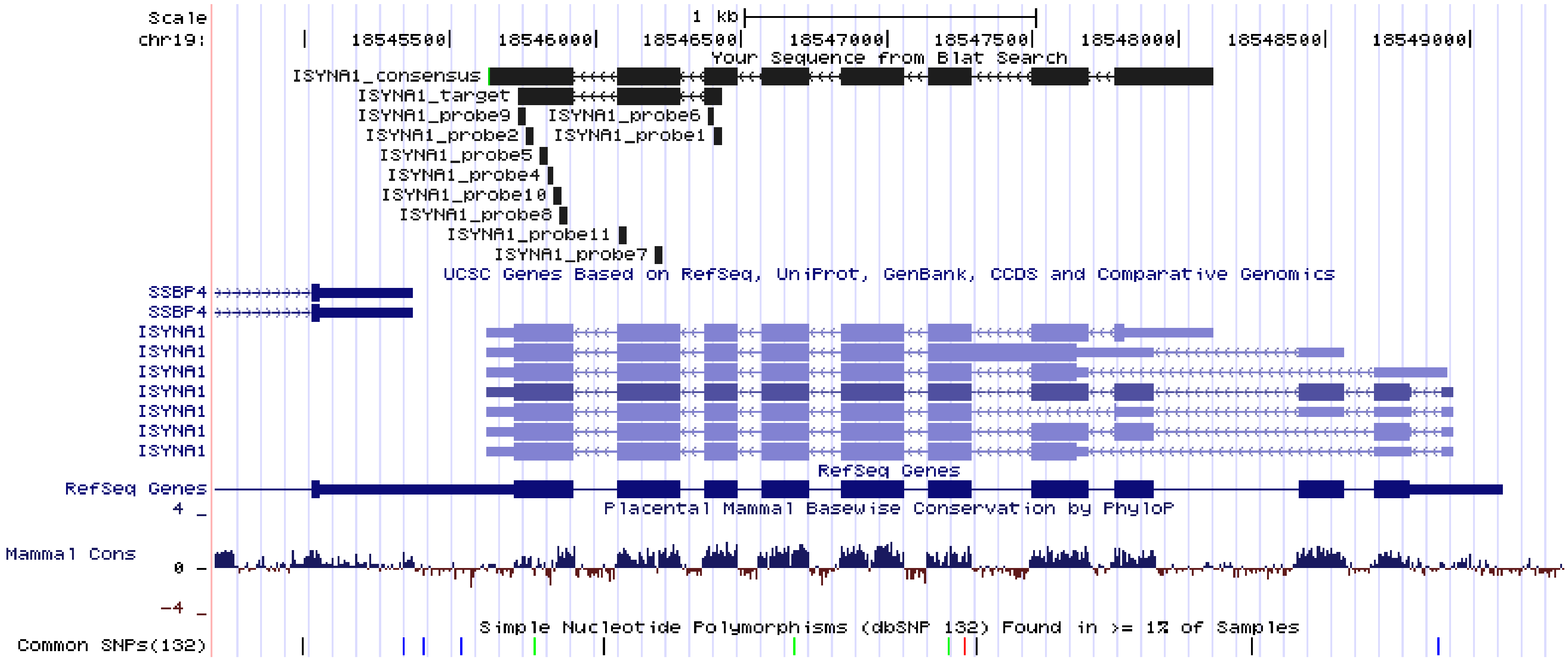

SKP2 (203625_x_at)

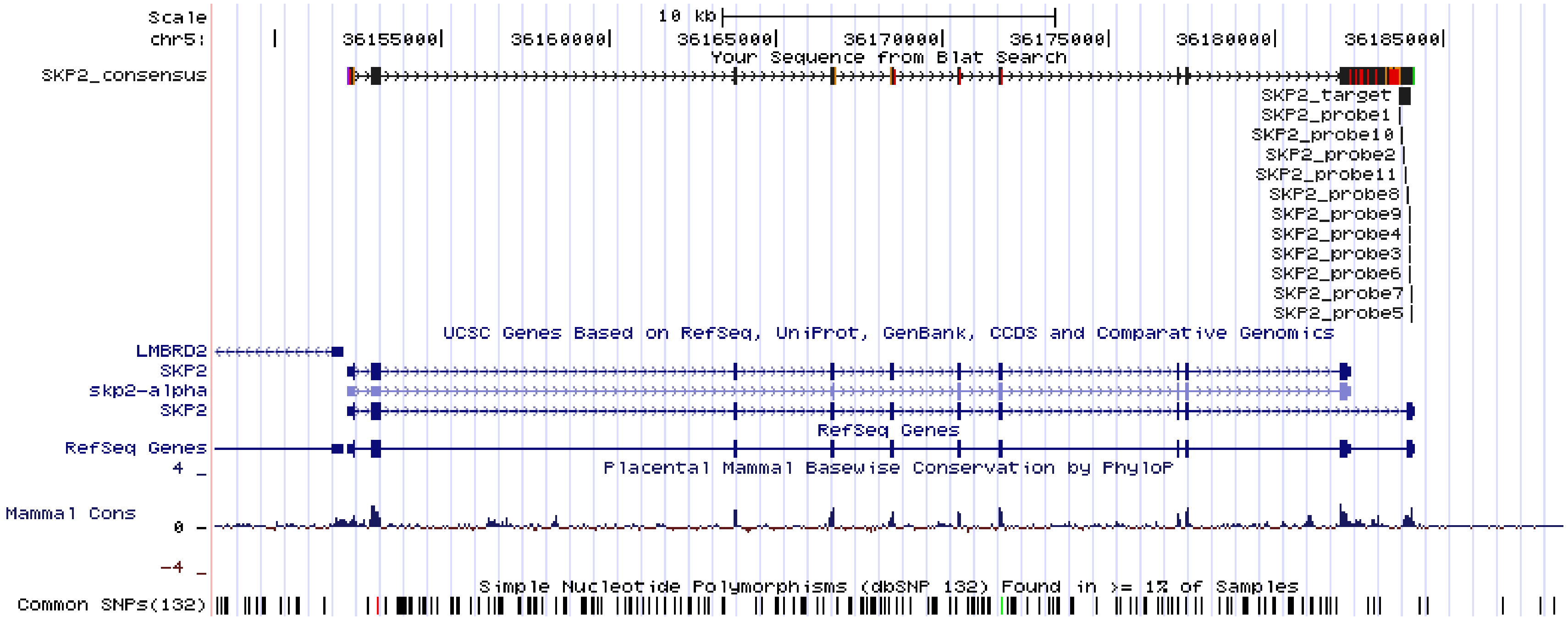

DPP3 (218567_x_at)

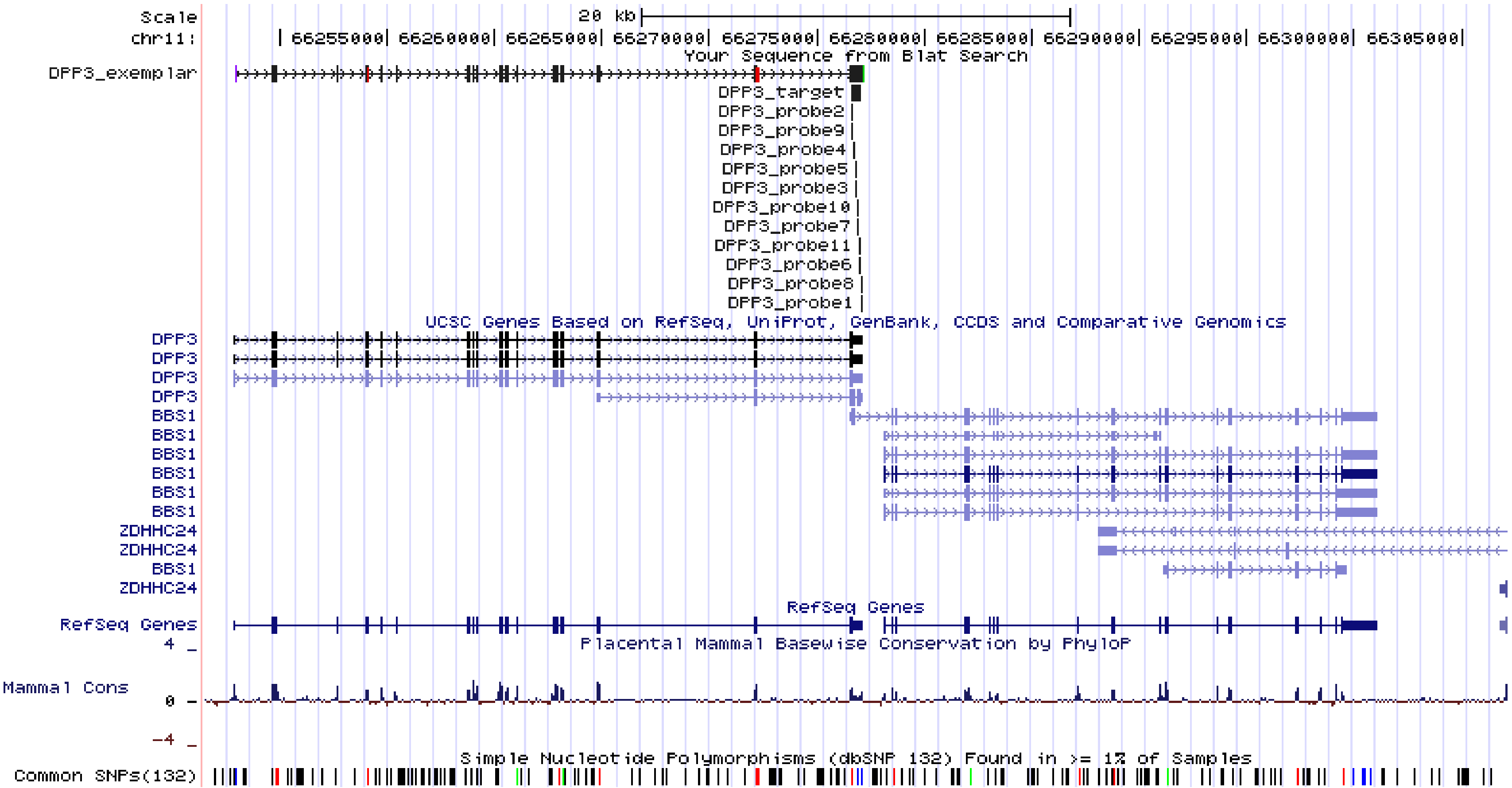

TYMP (204858_s_at)

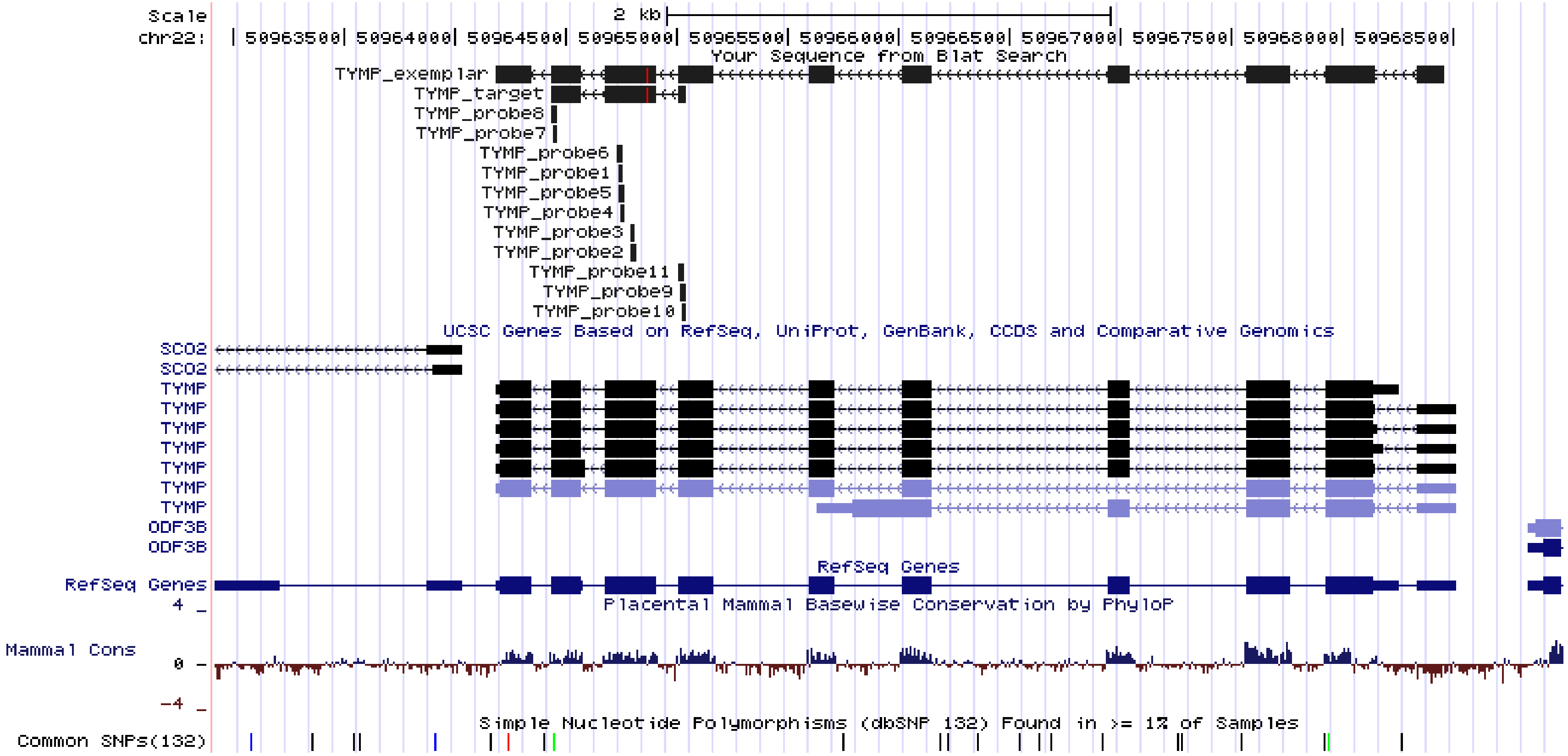

SNRPA1 (216977_x_at)

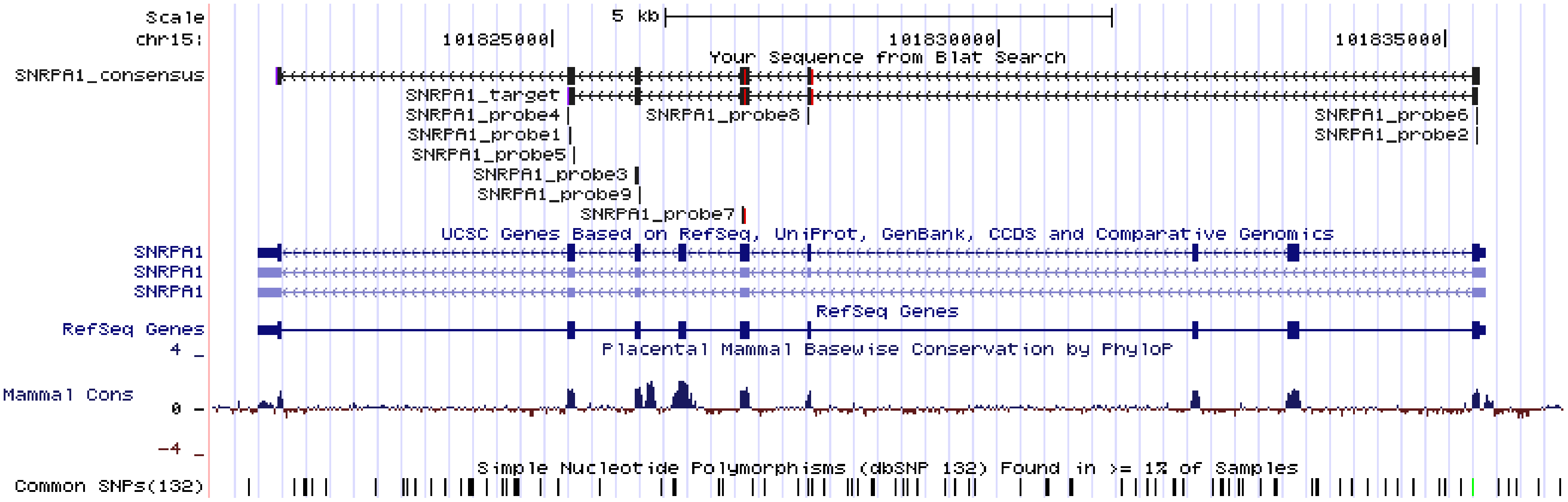

DHCR7 (201791_s_at)

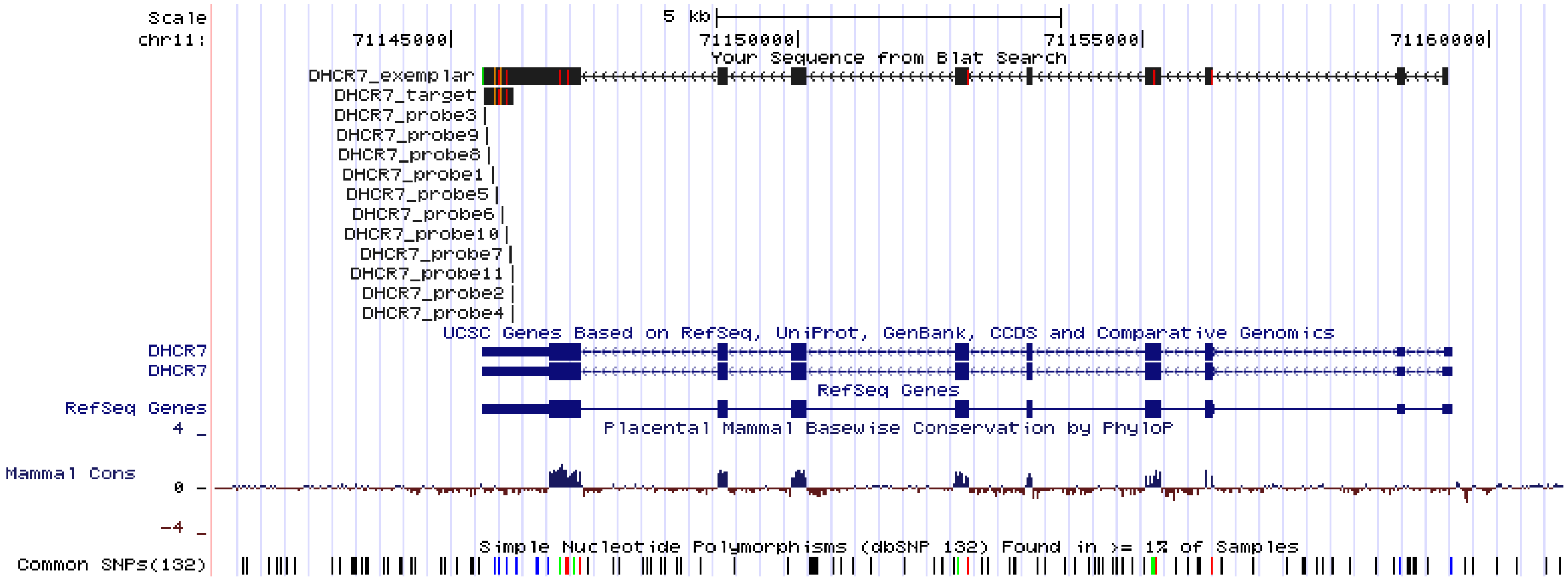

TFPT (218996_at)

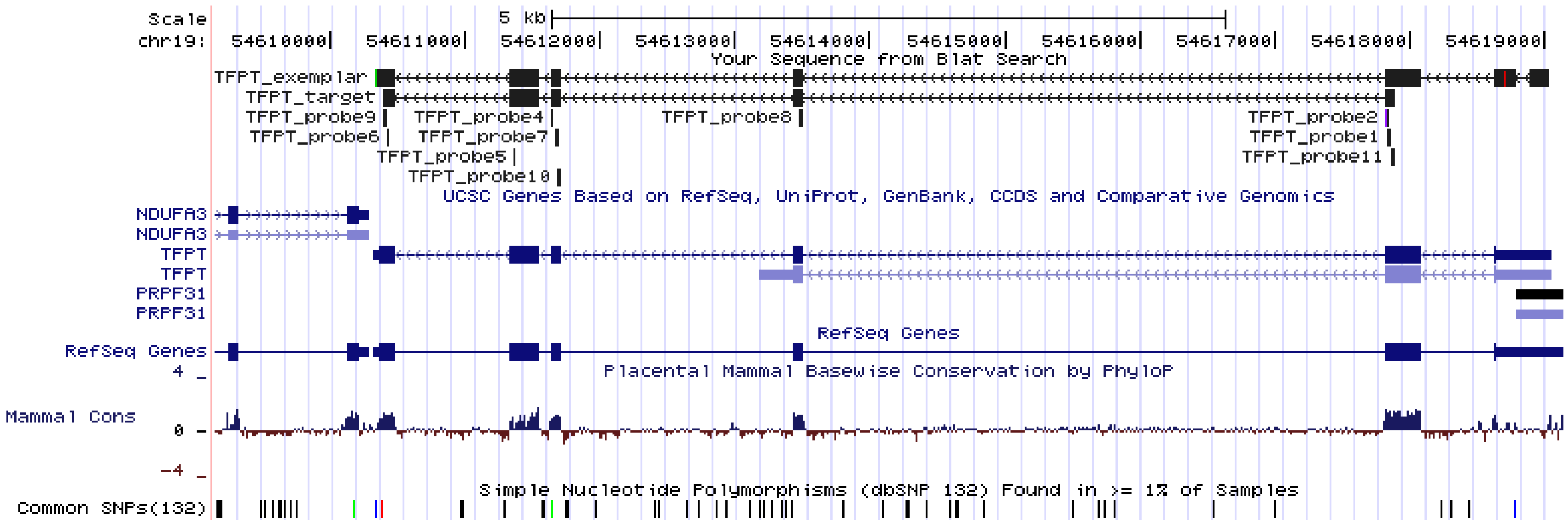

CTTN (2017_at)

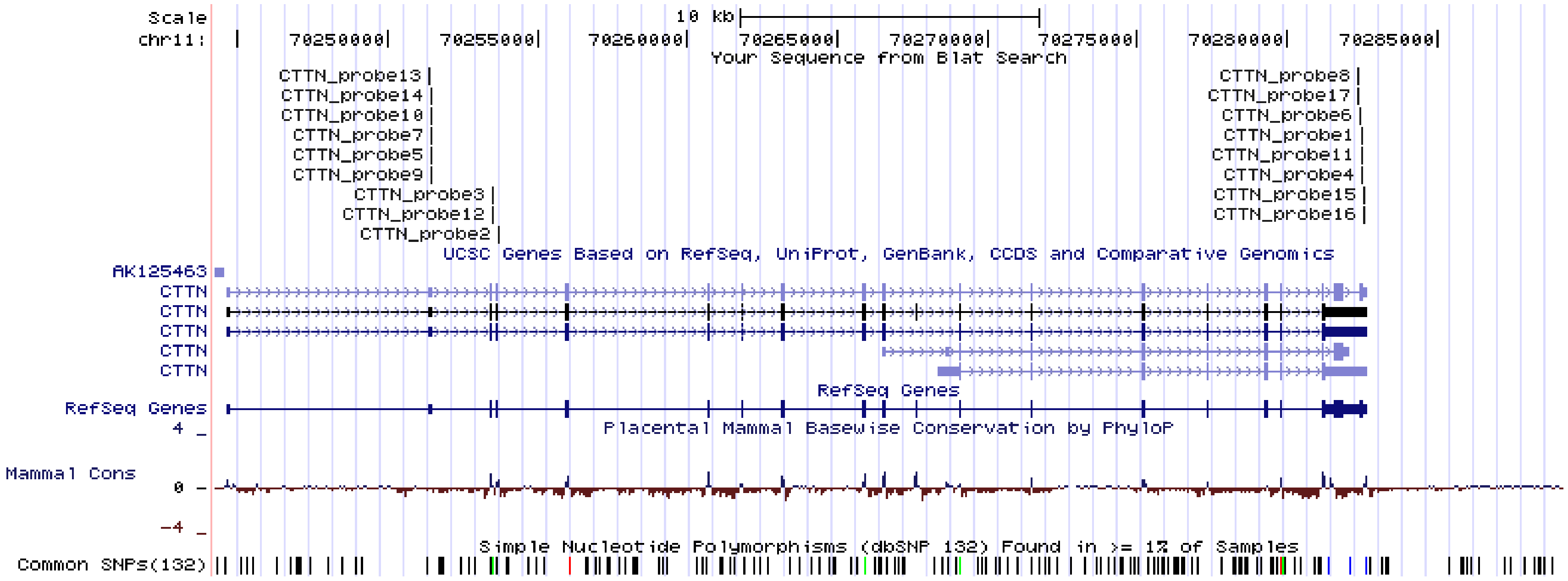
Scale
10 kb
chr11:
70250000
70255000
70260000
70265000
70270000
70275000
70280000
70285000
Your Sequence from Blat Search
CTTN_probe13
CTTN_probe14
CTTN_probe10
CTTN_probe7
CTTN_probe5
CTTN_probe9
CTTN_probe3
CTTN_probe12
CTTN_probe2
CTTN_probe8
CTTN_probe17
CTTN_probe6
CTTN_probe1
CTTN_probe11
CTTN_probe4
CTTN_probe15
CTTN_probe16
UCSC Genes Based on RefSeq, UniProt, GenBank, CCDS and Comparative Genomics
AK125463
CTTN
CTTN
CTTN
CTTN
CTTN
RefSeq Genes
RefSeq Genes
4
Placental Mammal Basewise Conservation by PhyloP
Mammal Cons
0
-4
Simple Nucleotide Polymorphisms (dbSNP 132) Found in >= 1% of Samples
Common SNPs(132)

MCM5 (216237_s_at)

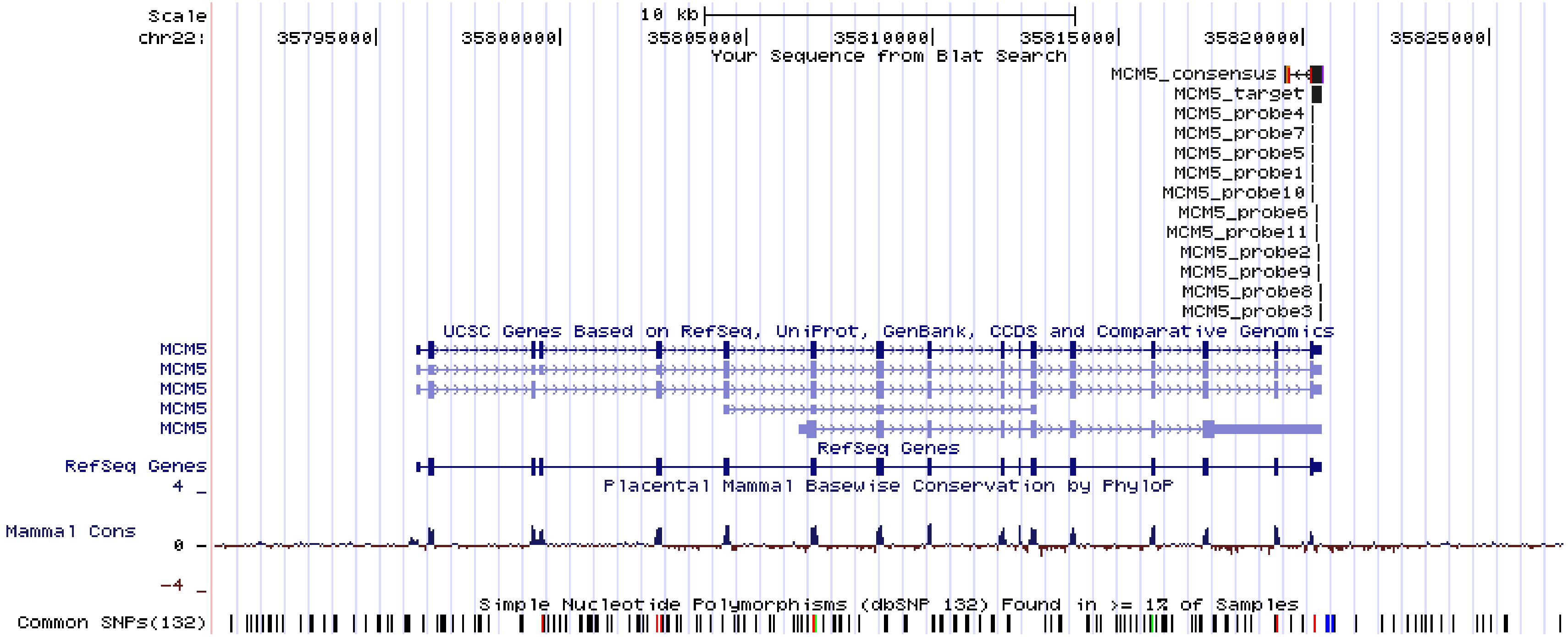

TXNIP (10628_at)

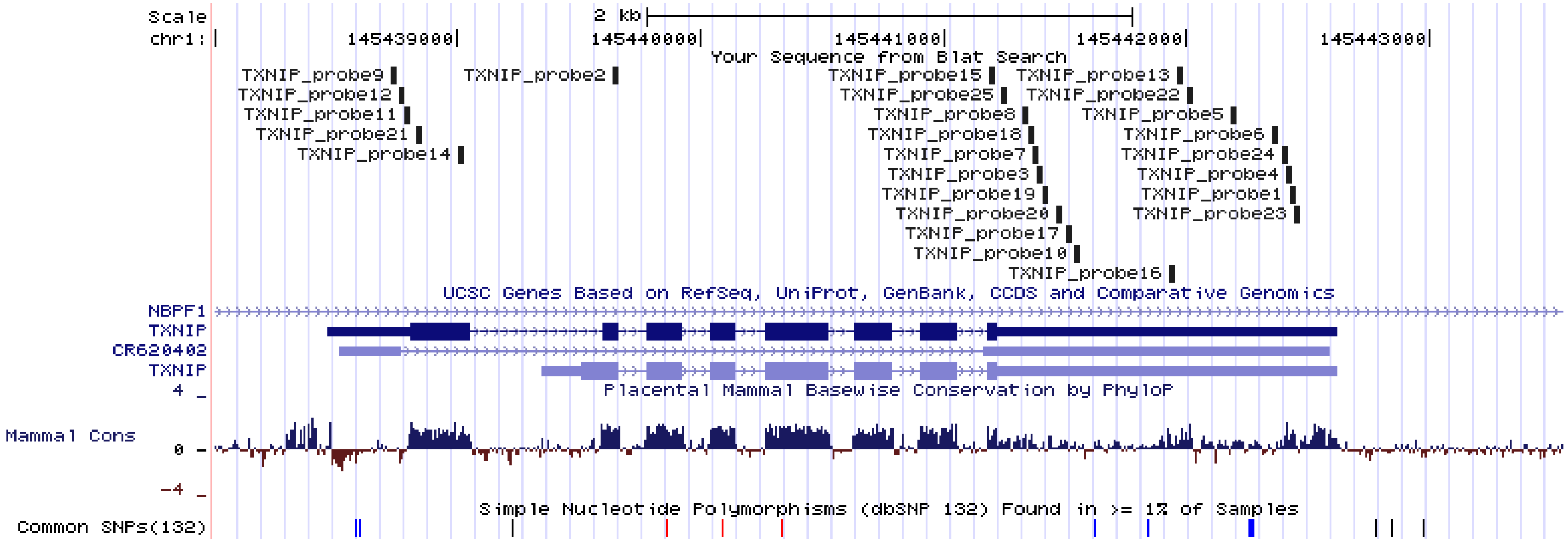

SYNE2 (23224_at)

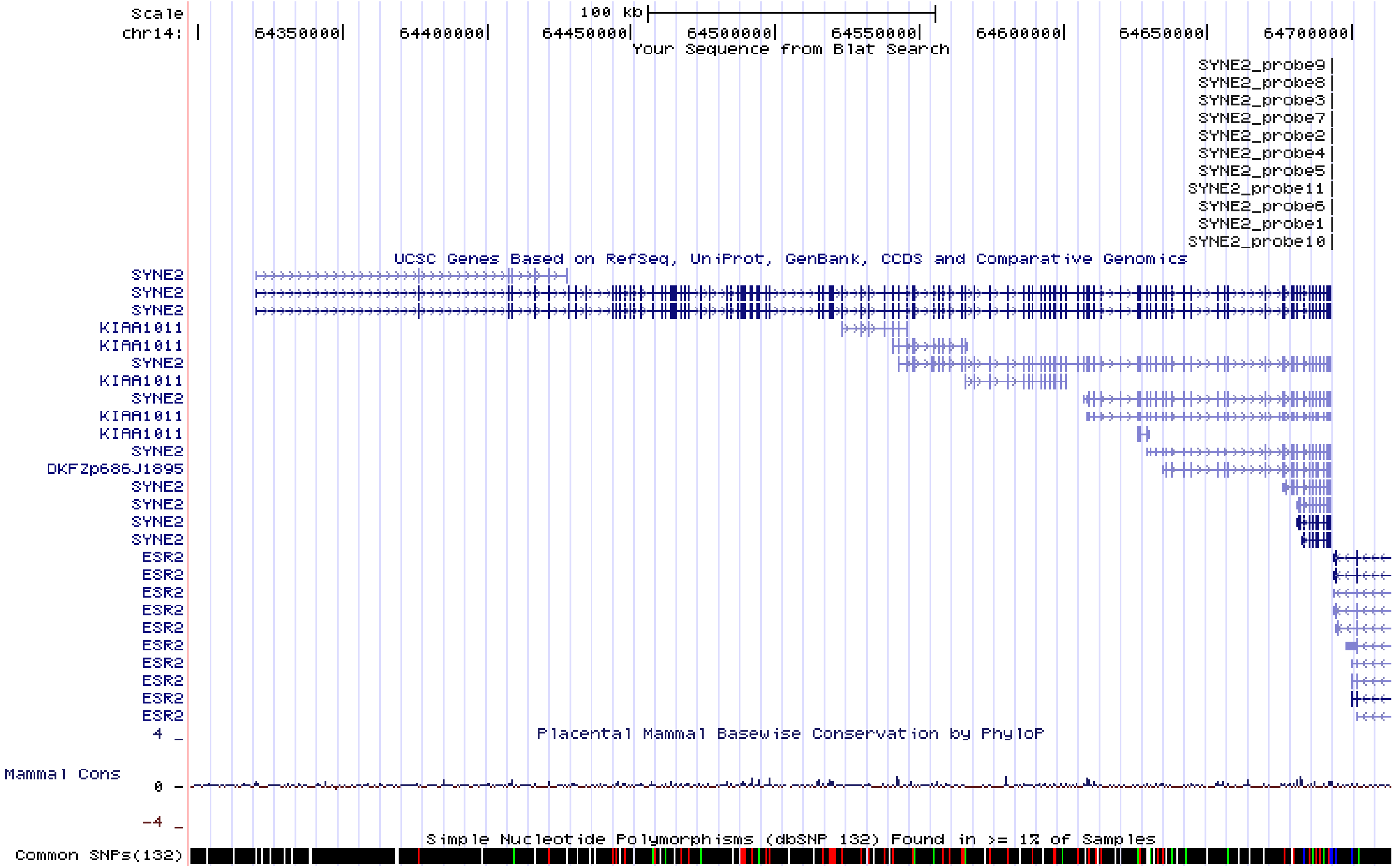

SCARB2 (201646_at)

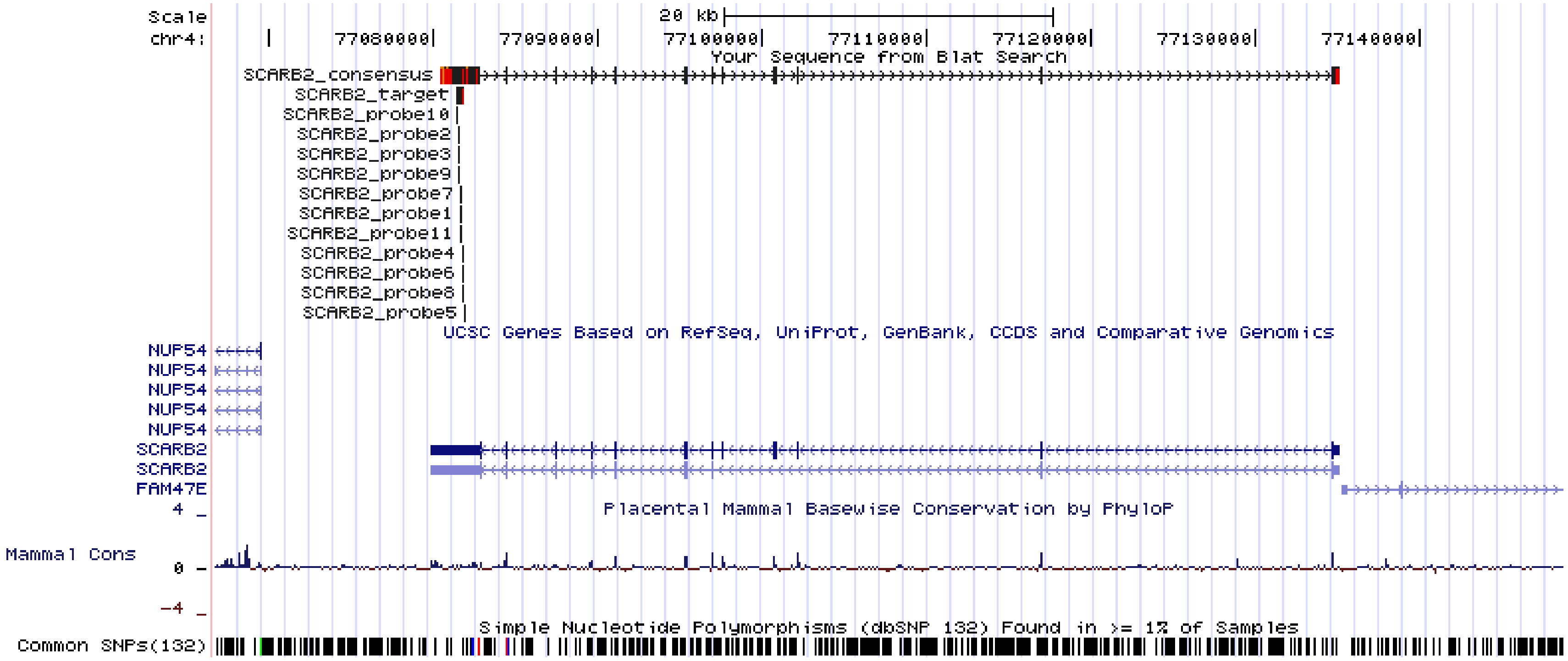

PDLIM5 (216804_s_at)

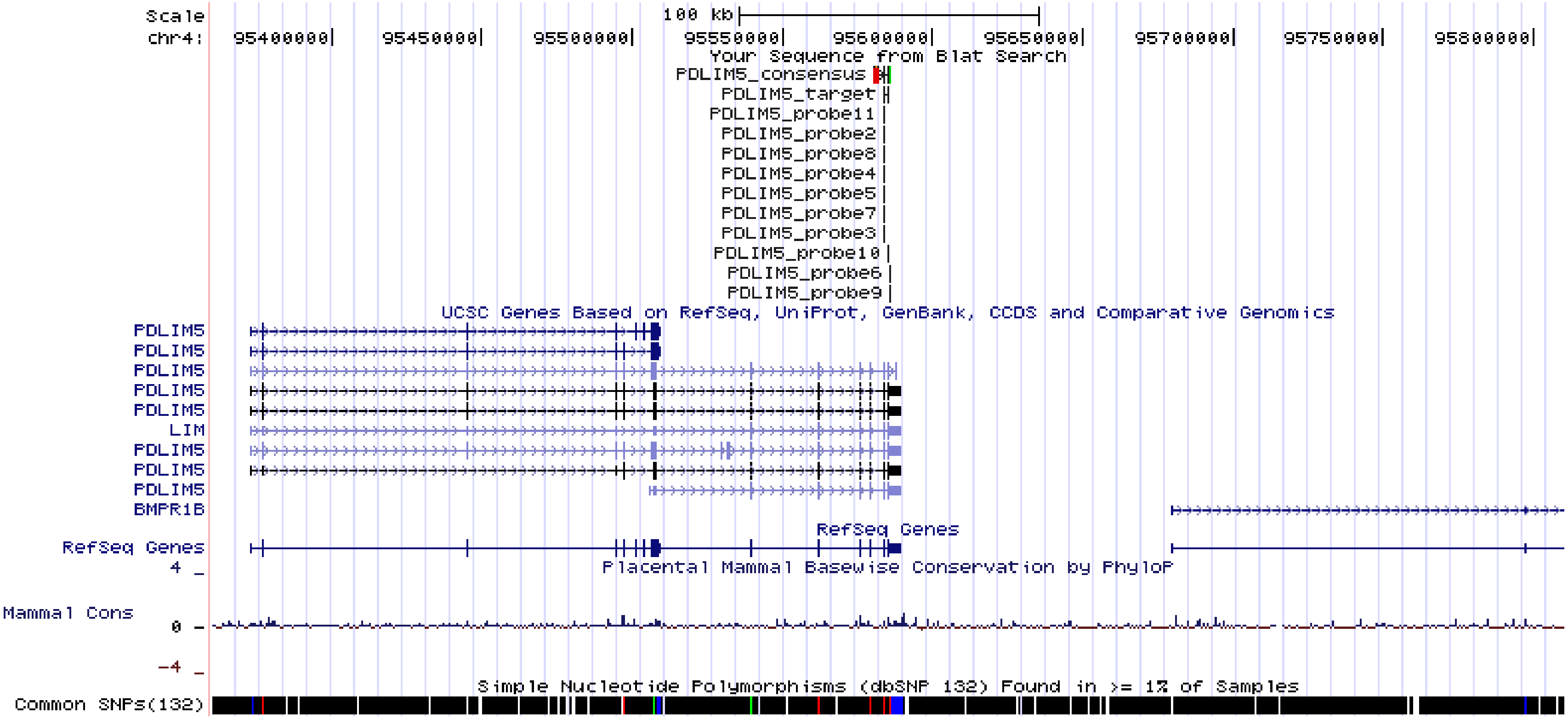

TSC2 (7249_at)

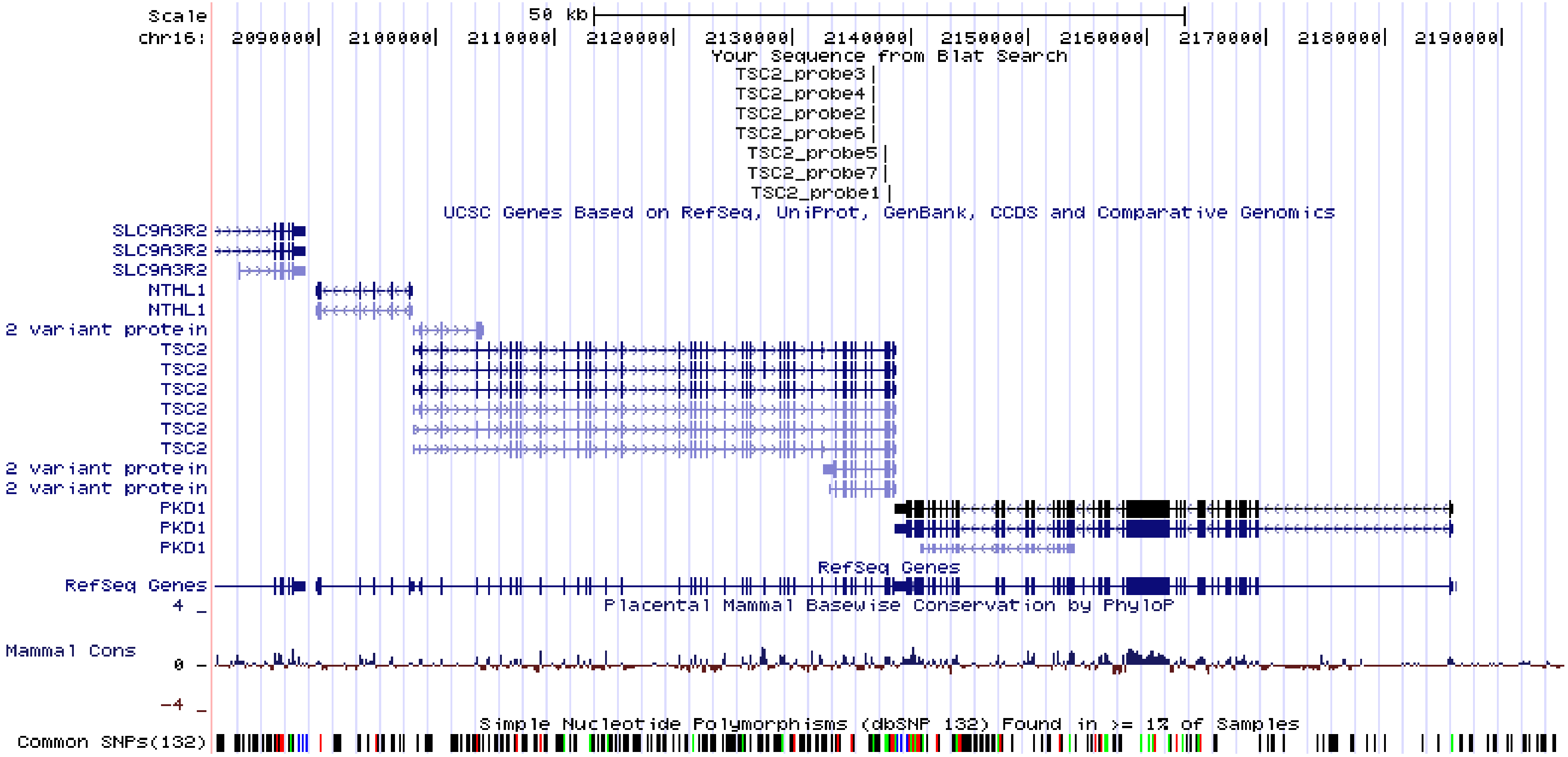

Scale
50 kb
chr16:
2090000 2100000 2110000 2120000 2130000 2140000 2150000 2160000 2170000 2180000 2190000
Your Sequence from Blat Search
TSC2_probe3
TSC2_probe4
TSC2_probe2
TSC2_probe6
TSC2_probe5
TSC2_probe7
TSC2_probe1
UCSC Genes Based on RefSeq, UniProt, GenBank, CCDS and Comparative Genomics
SLC9A3R2
SLC9A3R2
SLC9A3R2
NTHL1
NTHL1
2 variant protein
TSC2
TSC2
TSC2
TSC2
TSC2
TSC2
2 variant protein
2 variant protein
PKD1
PKD1
PKD1
RefSeq Genes
RefSeq Genes
4 _
Placental Mammal Basewise Conservation by PhyloP
Mammal Cons
0 _
-4 _
Simple Nucleotide Polymorphisms (dbSNP 132) Found in >= 1% of Samples
Common SNPs(132)

ELF1 (212420_at)

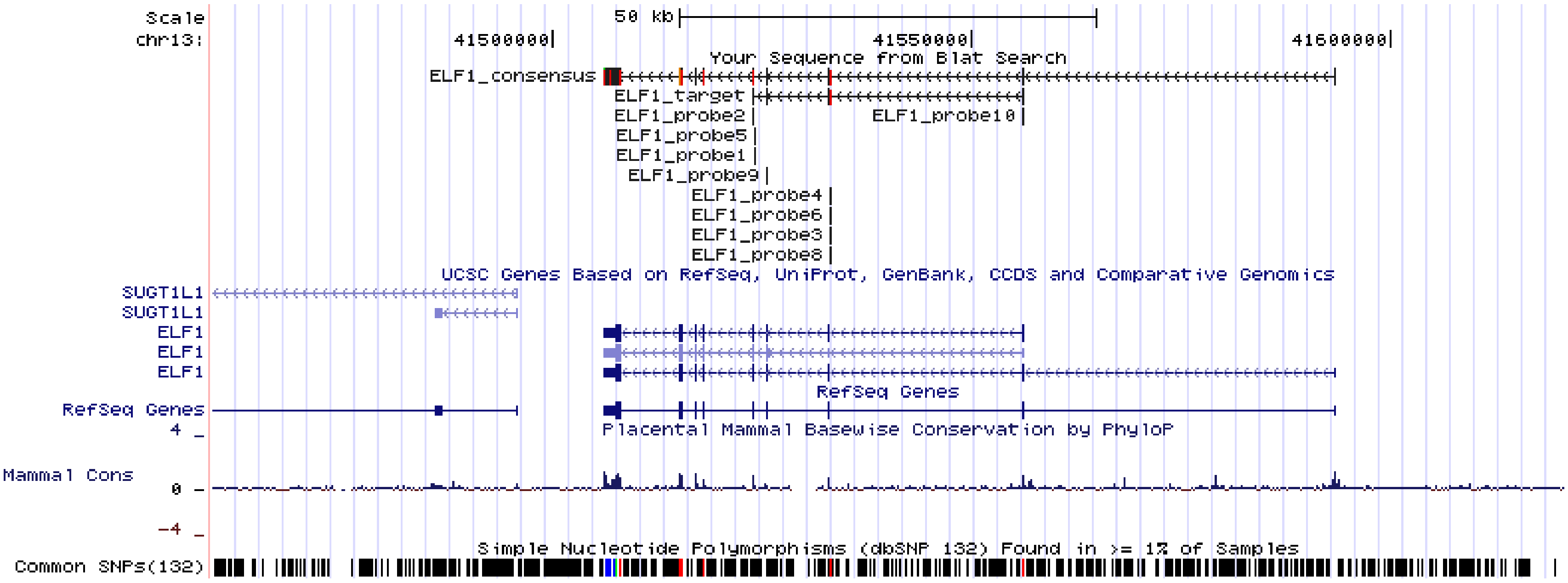

DICER1 (23405_at)

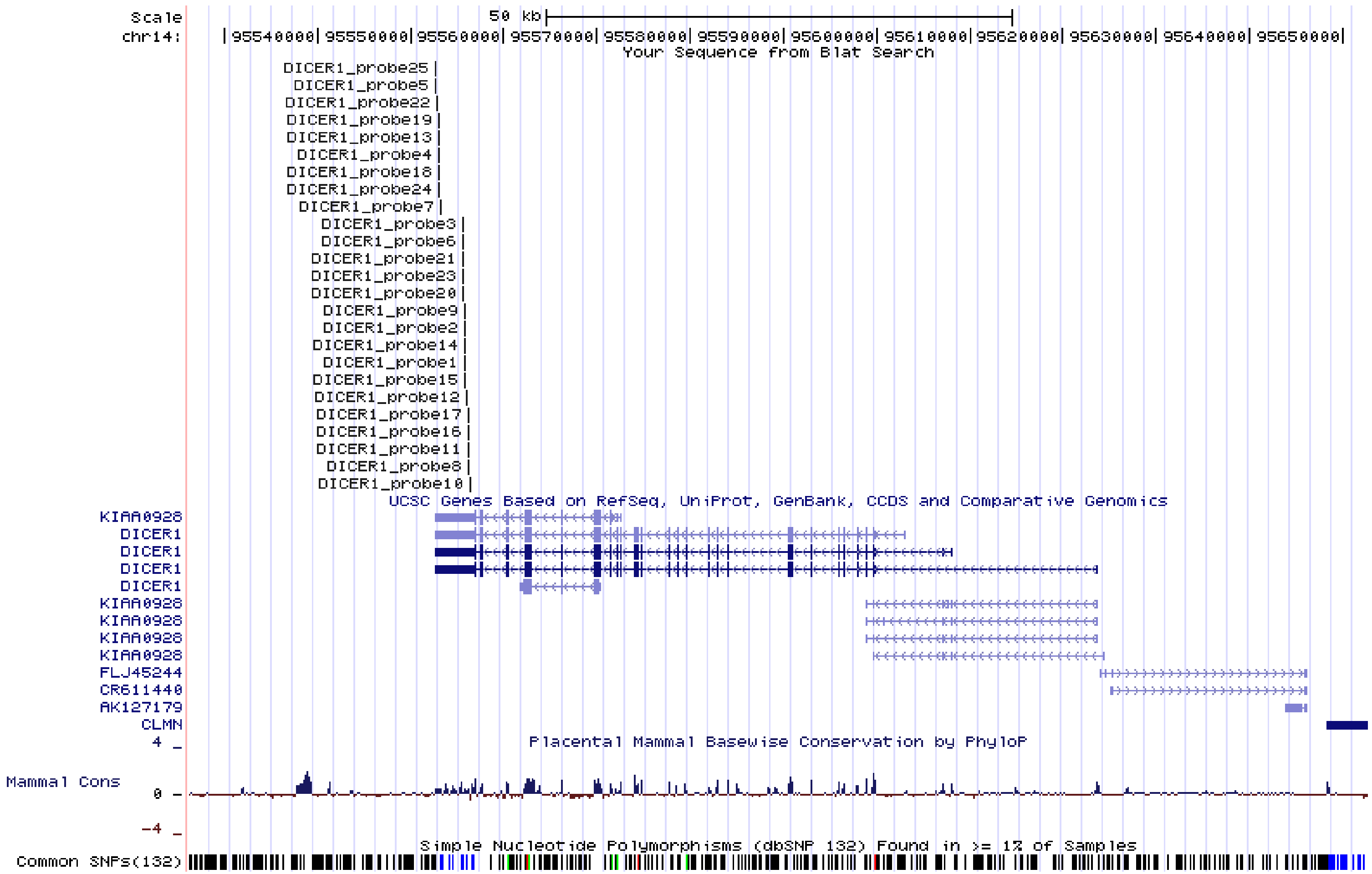

CALD1 (201616_s_at)

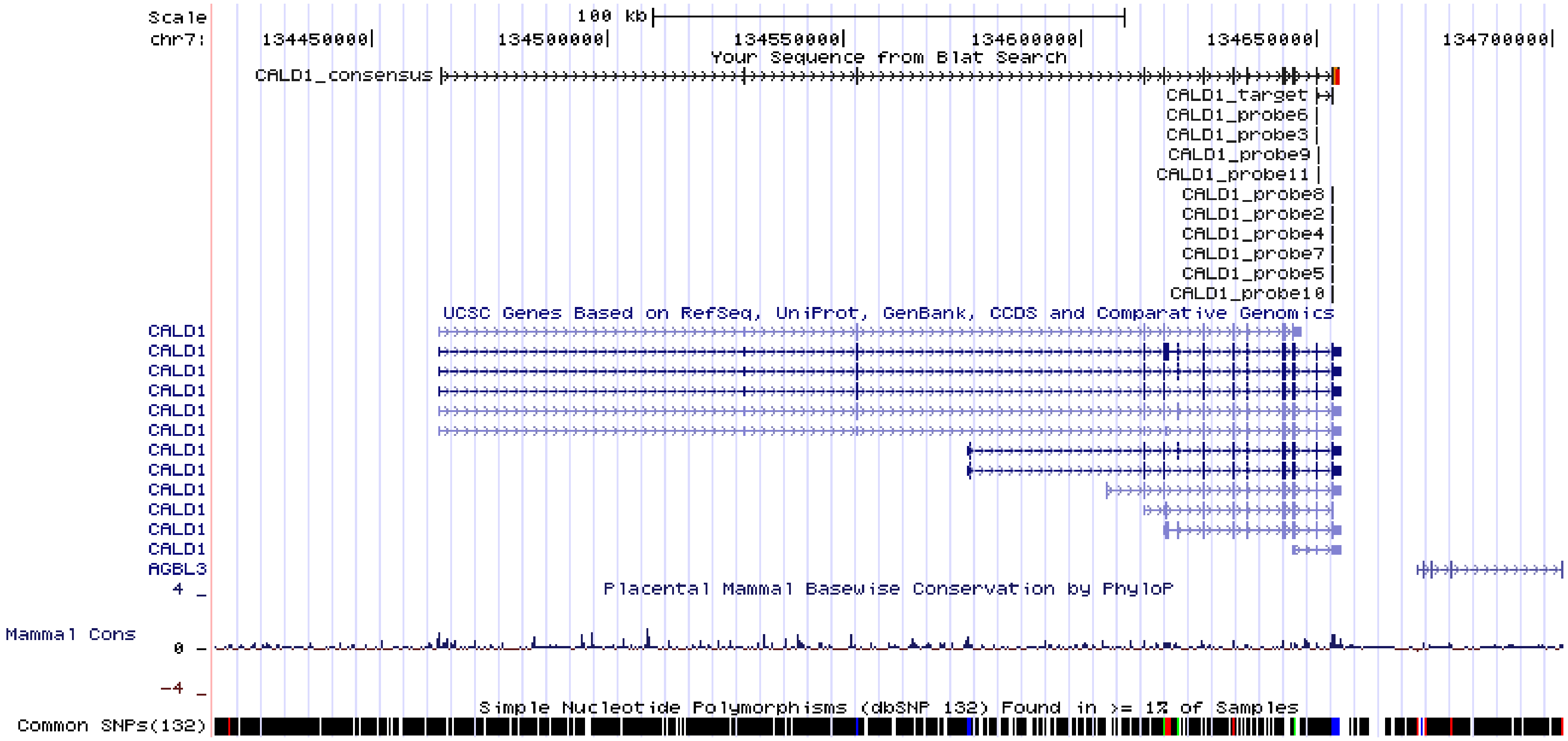

SOX9 (6662_at)

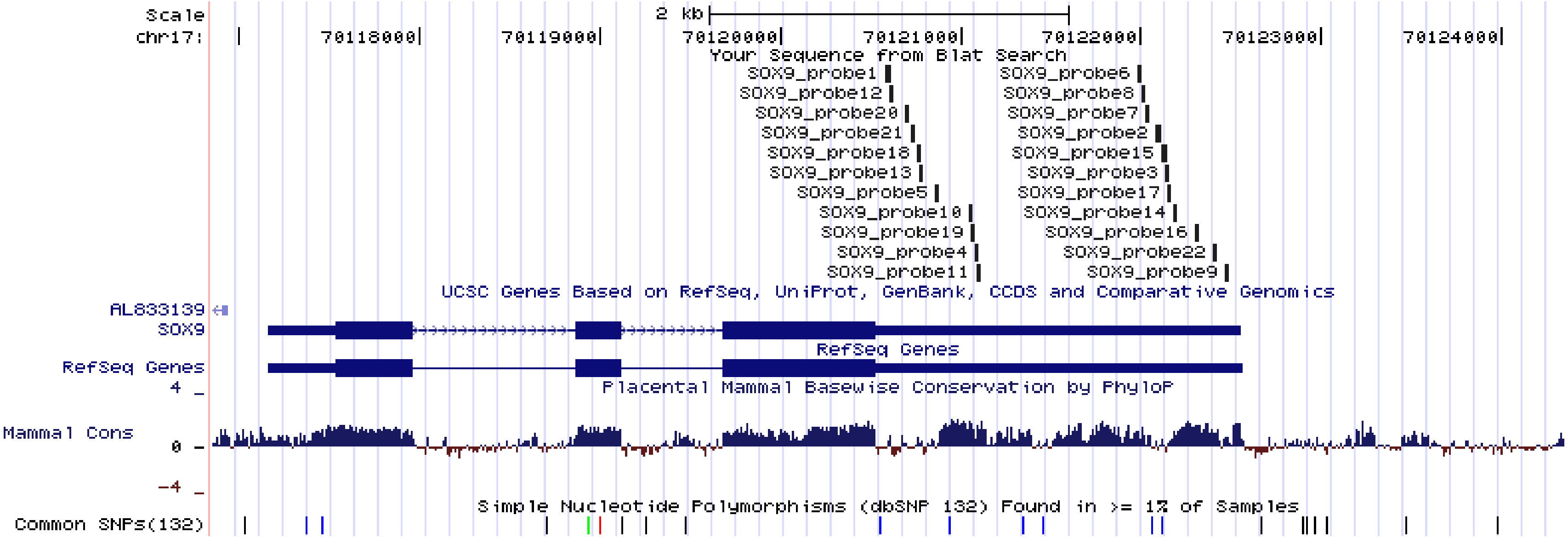
Scale
chr17:
2 kb
70118000
70119000
70120000
70121000
70122000
70123000
70124000
Your Sequence from Blat Search
SOX9_probe1
SOX9_probe12
SOX9_probe20
SOX9_probe21
SOX9_probe18
SOX9_probe13
SOX9_probe5
SOX9_probe10
SOX9_probe19
SOX9_probe4
SOX9_probe11
SOX9_probe6
SOX9_probe8
SOX9_probe7
SOX9_probe2
SOX9_probe15
SOX9_probe3
SOX9_probe17
SOX9_probe14
SOX9_probe16
SOX9_probe22
SOX9_probe9
UCSC Genes Based on RefSeq, UniProt, GenBank, CCDS and Comparative Genomics
AL833139
SOX9
RefSeq Genes
RefSeq Genes
4
Placental Mammal Basewise Conservation by PhyloP
Mammal Cons
0
-4
Simple Nucleotide Polymorphisms (dbSNP 132) Found in >= 1% of Samples
Common SNPs(132)

FAM20B (202915_s_at)

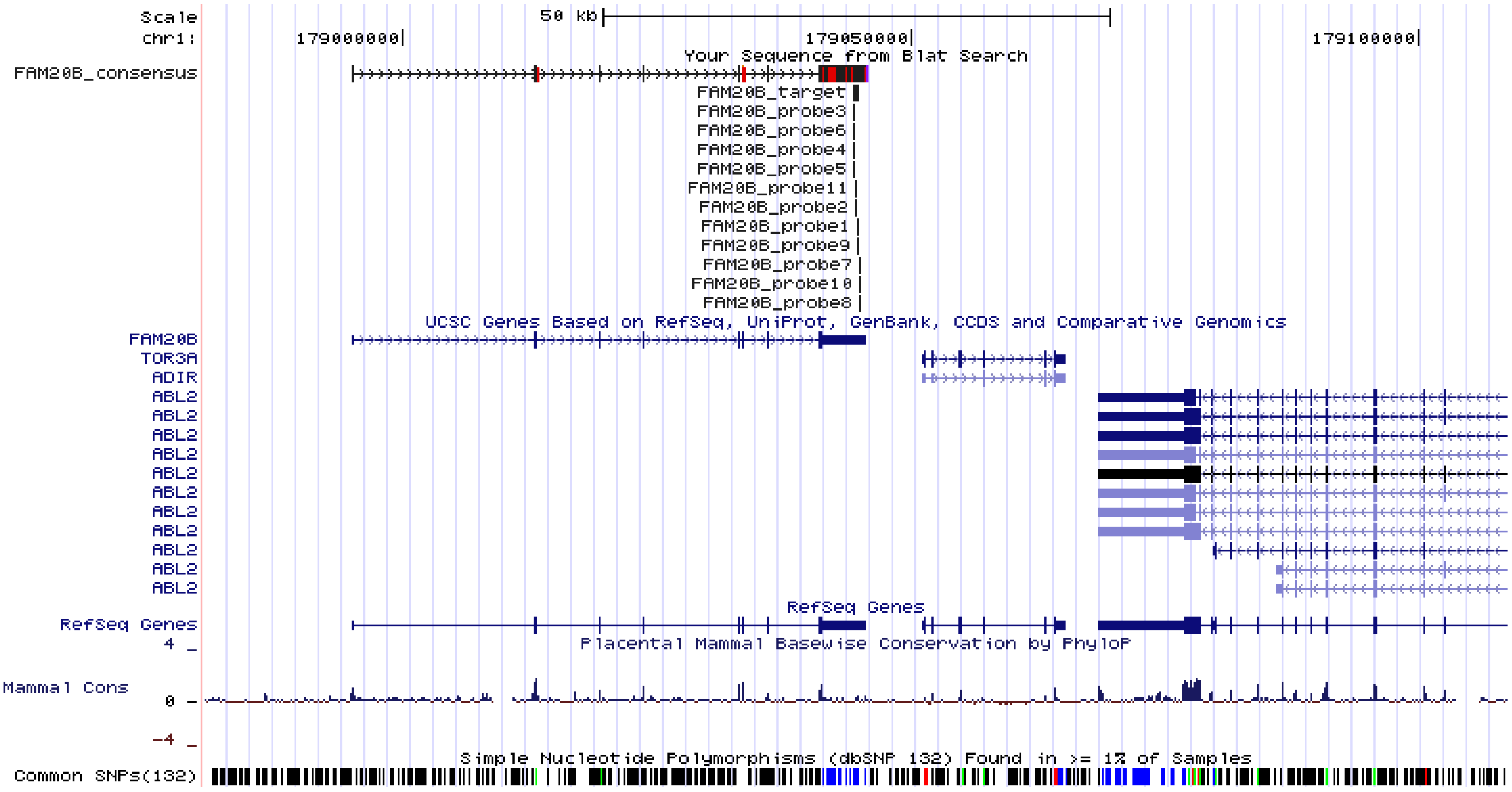

APH1A (218389_s_at)

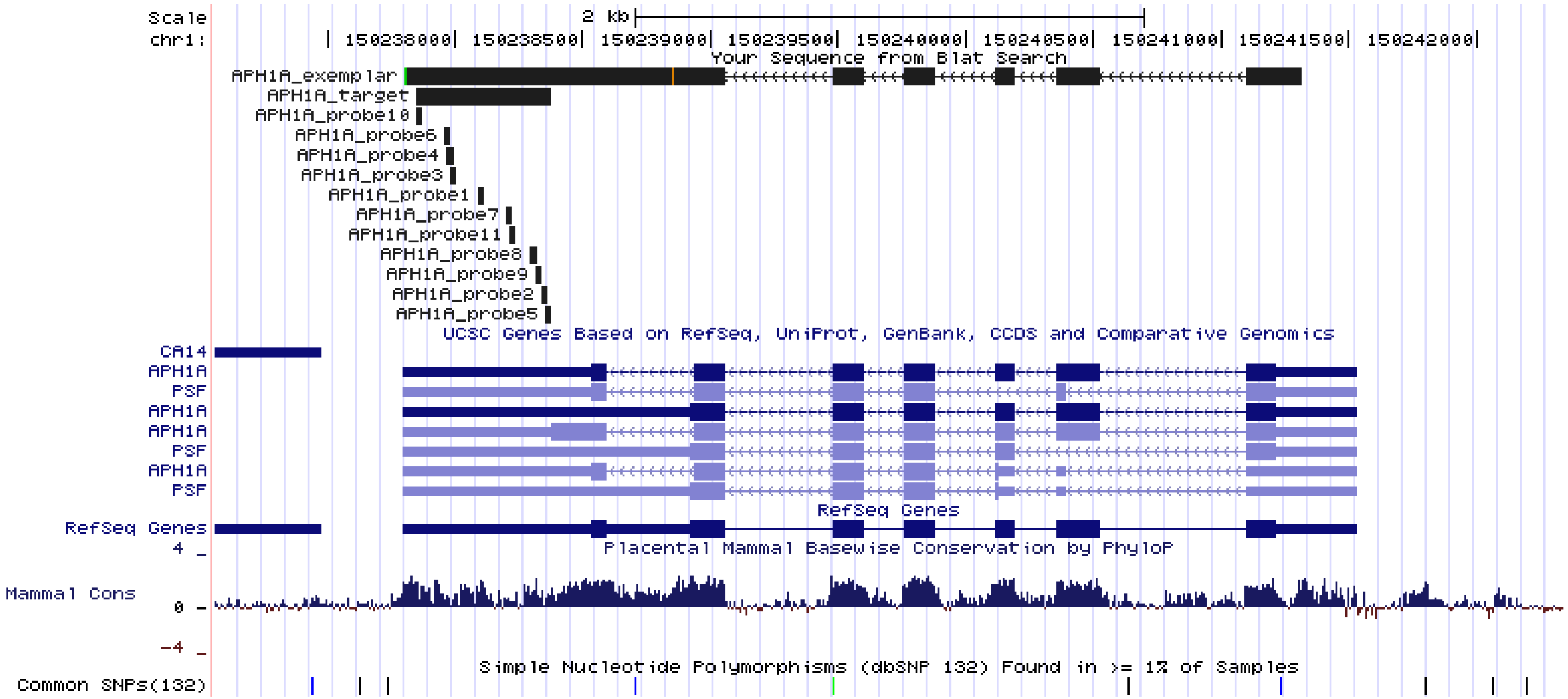

AP1AR (55435_at)

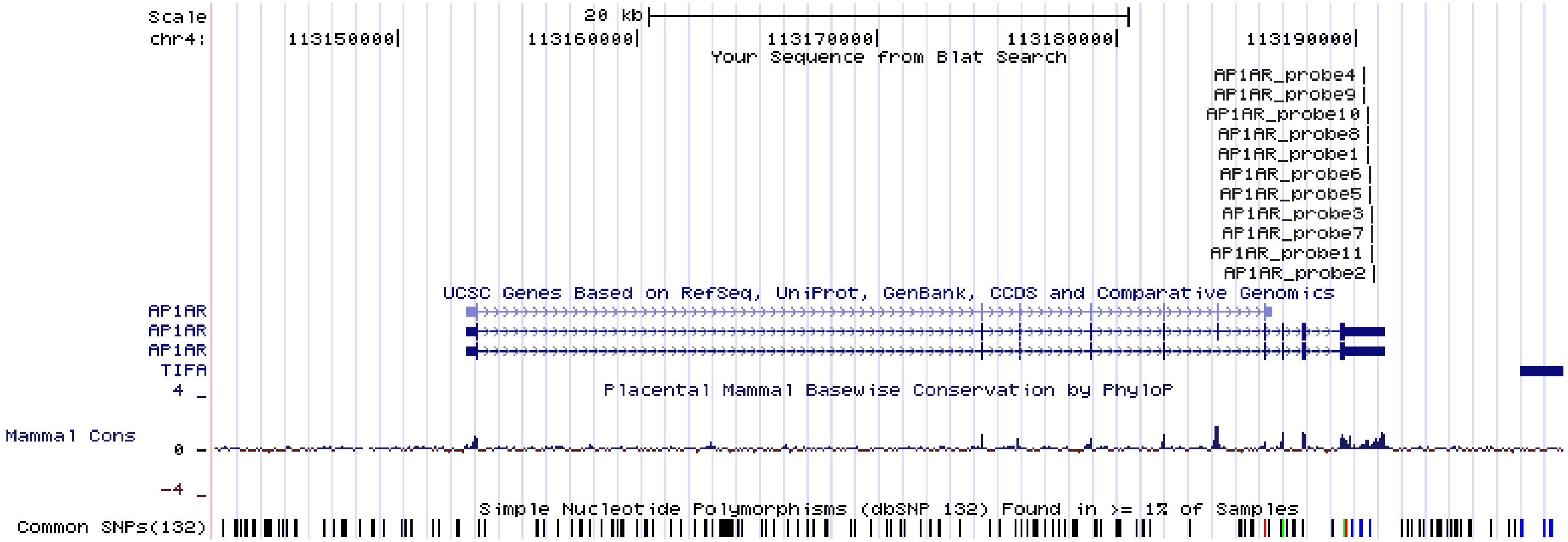

PDCD6 (222380_s_at)

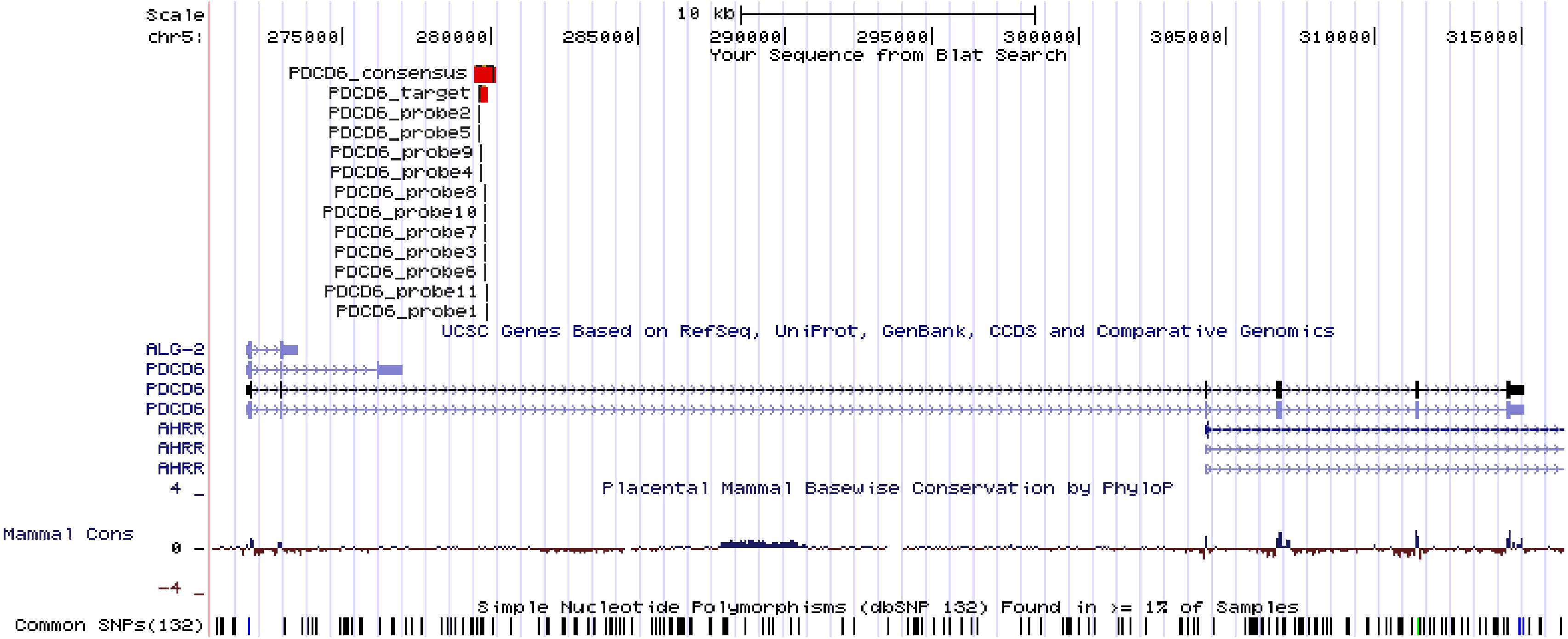

PBX2 (202876_s_at)

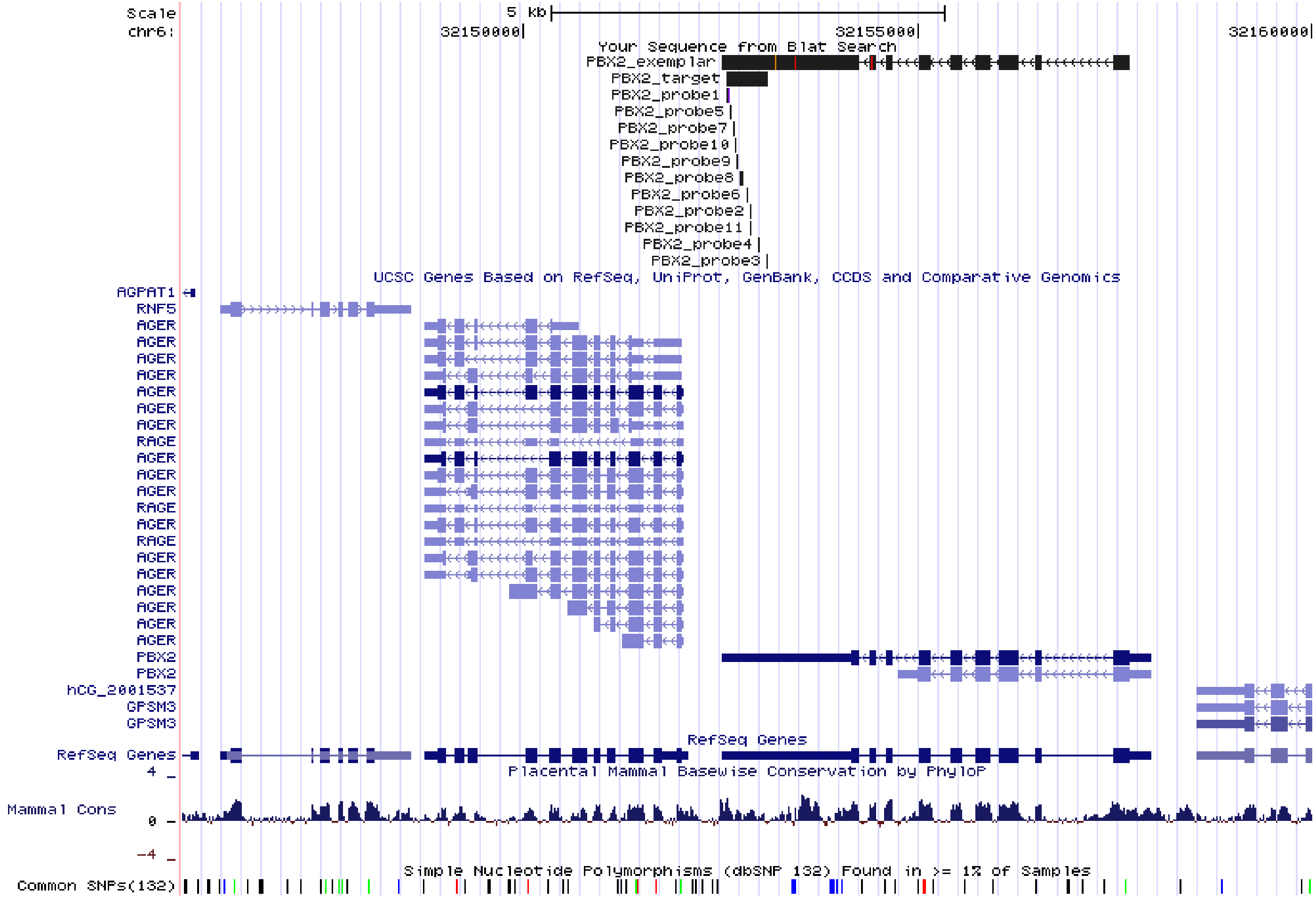

WASL (205809_s_at)

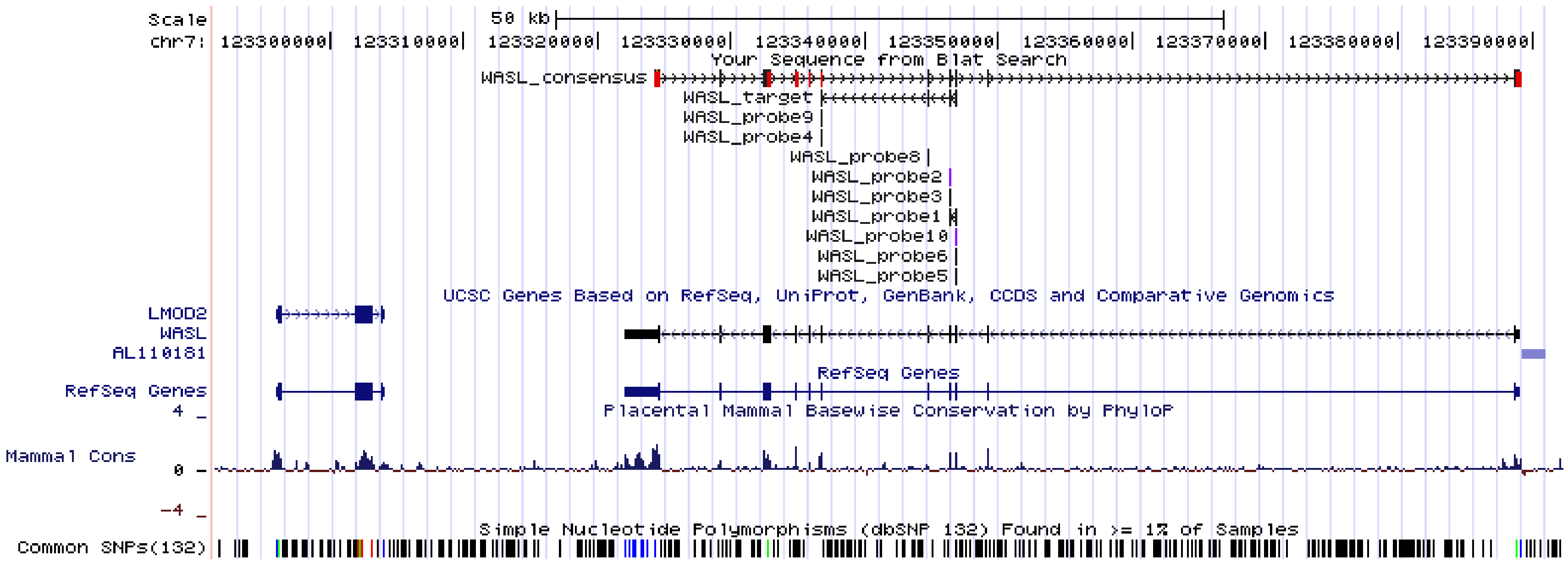

SLC11A2 (203123_s_at)

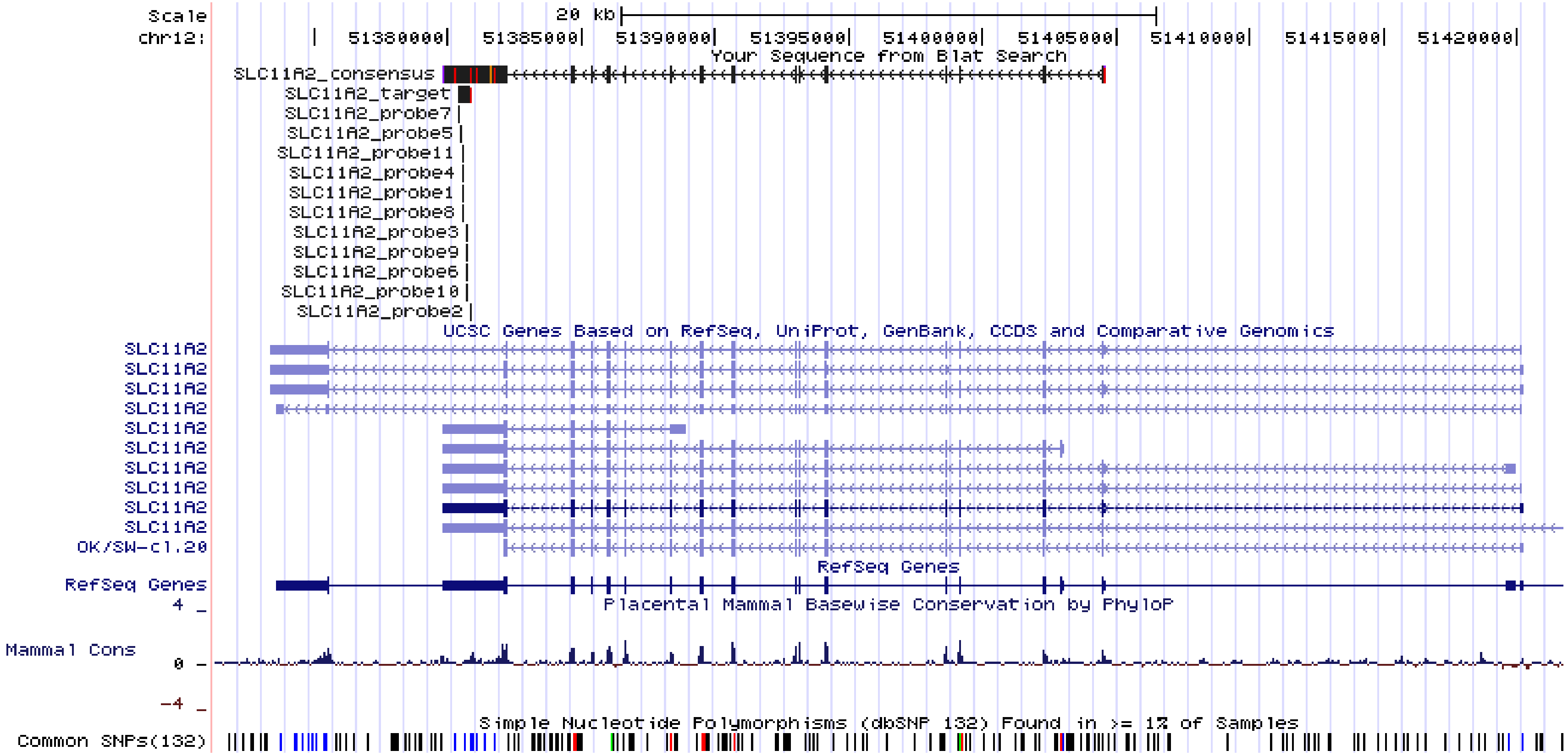

KIAA0776 (212634_at)

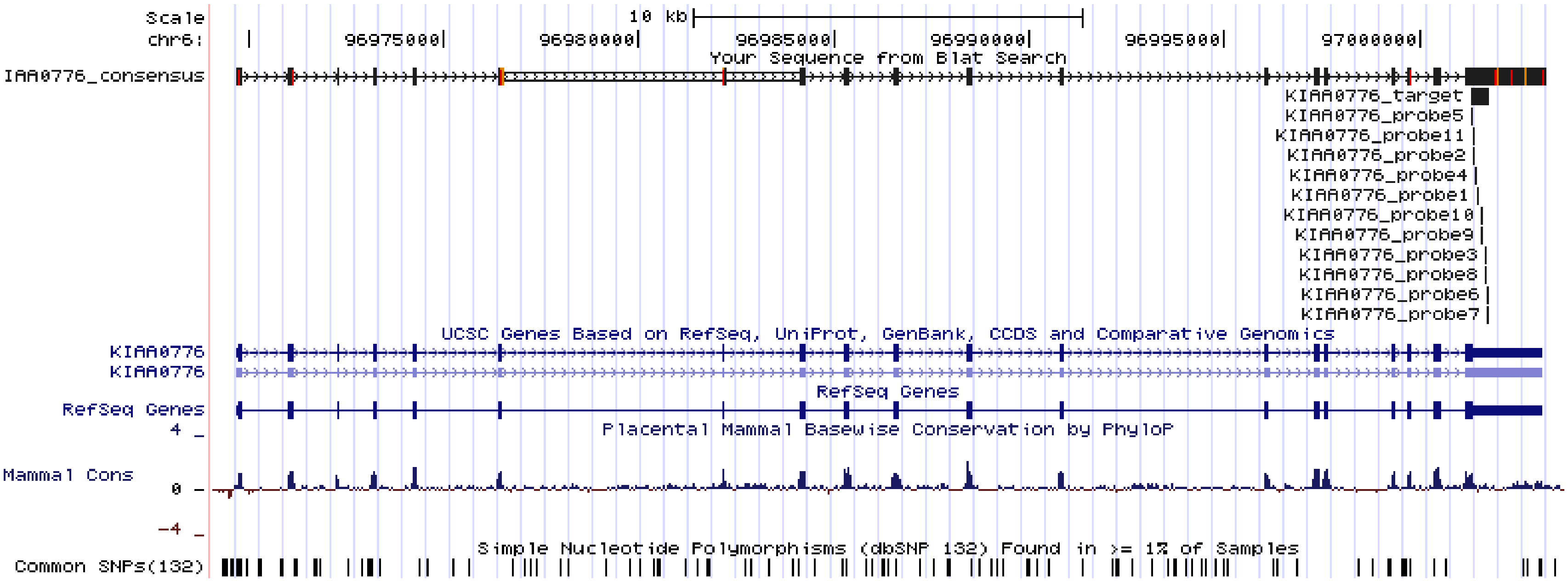

C14orf101 (54916_at)

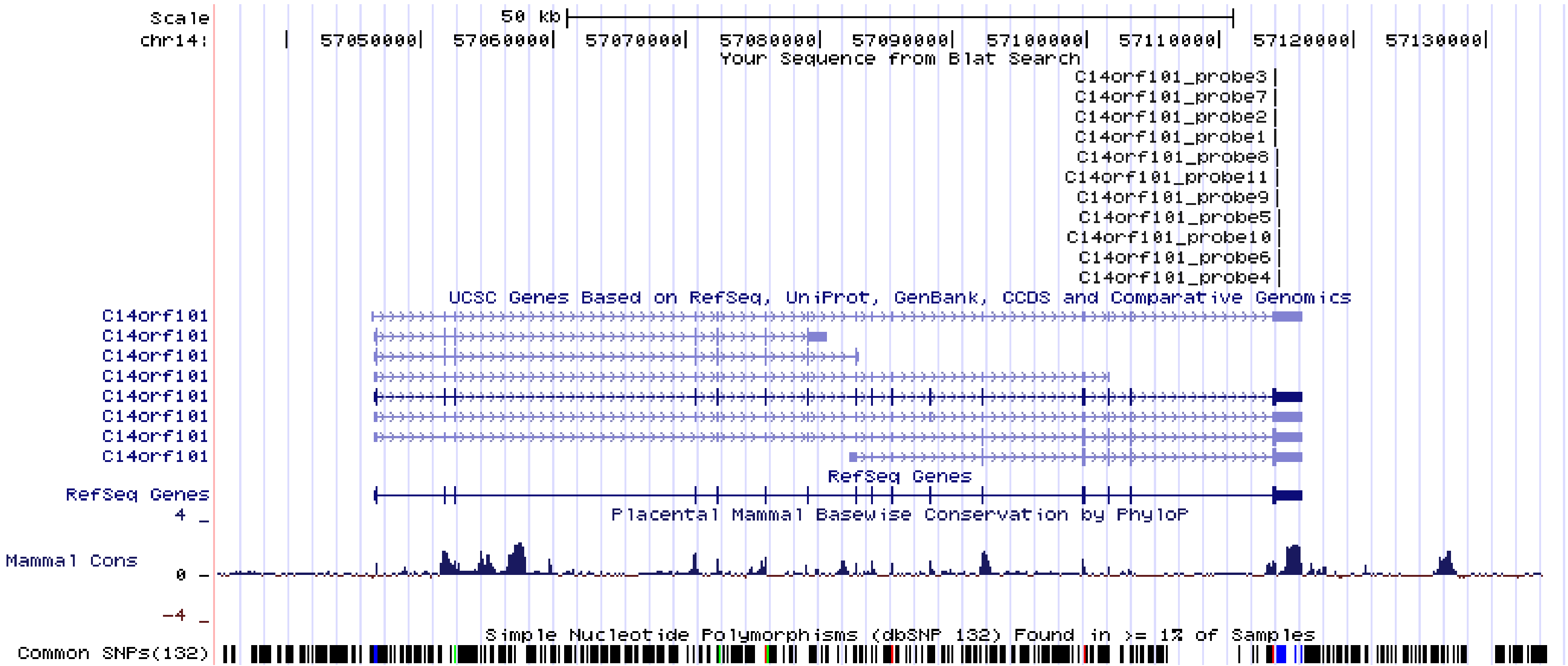

NUP107 (57122_at)

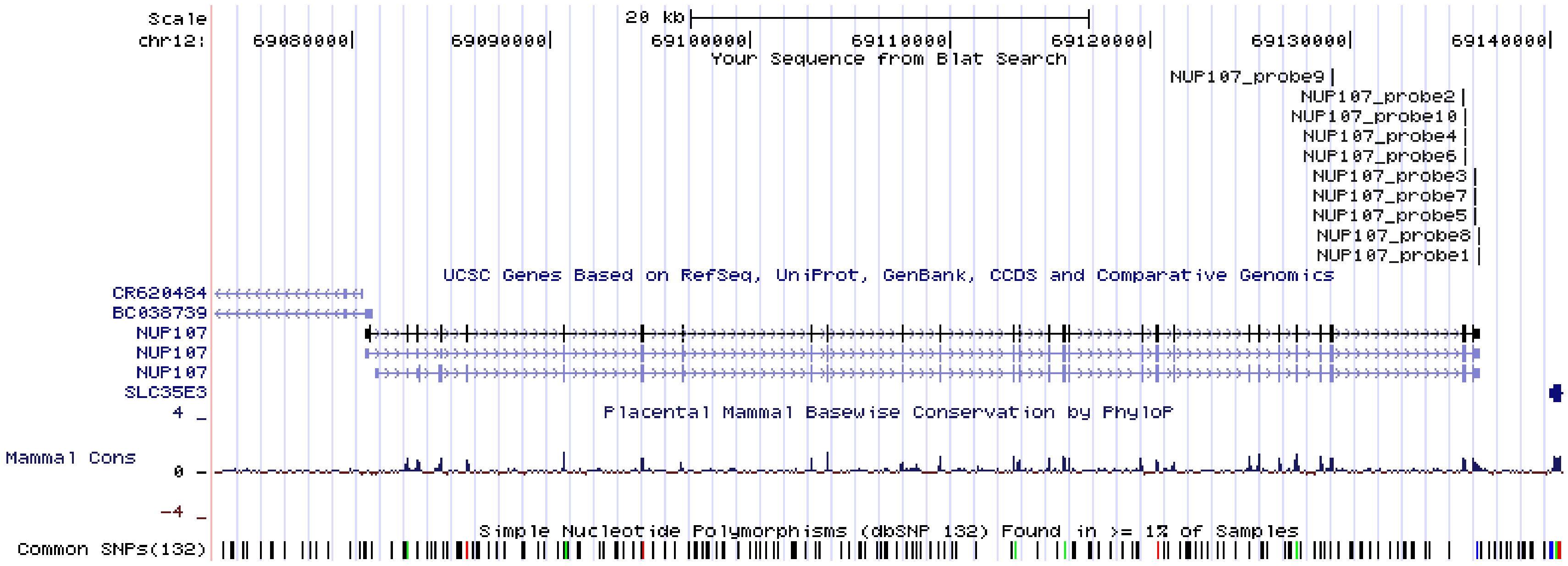

FAM38A (202771_at)

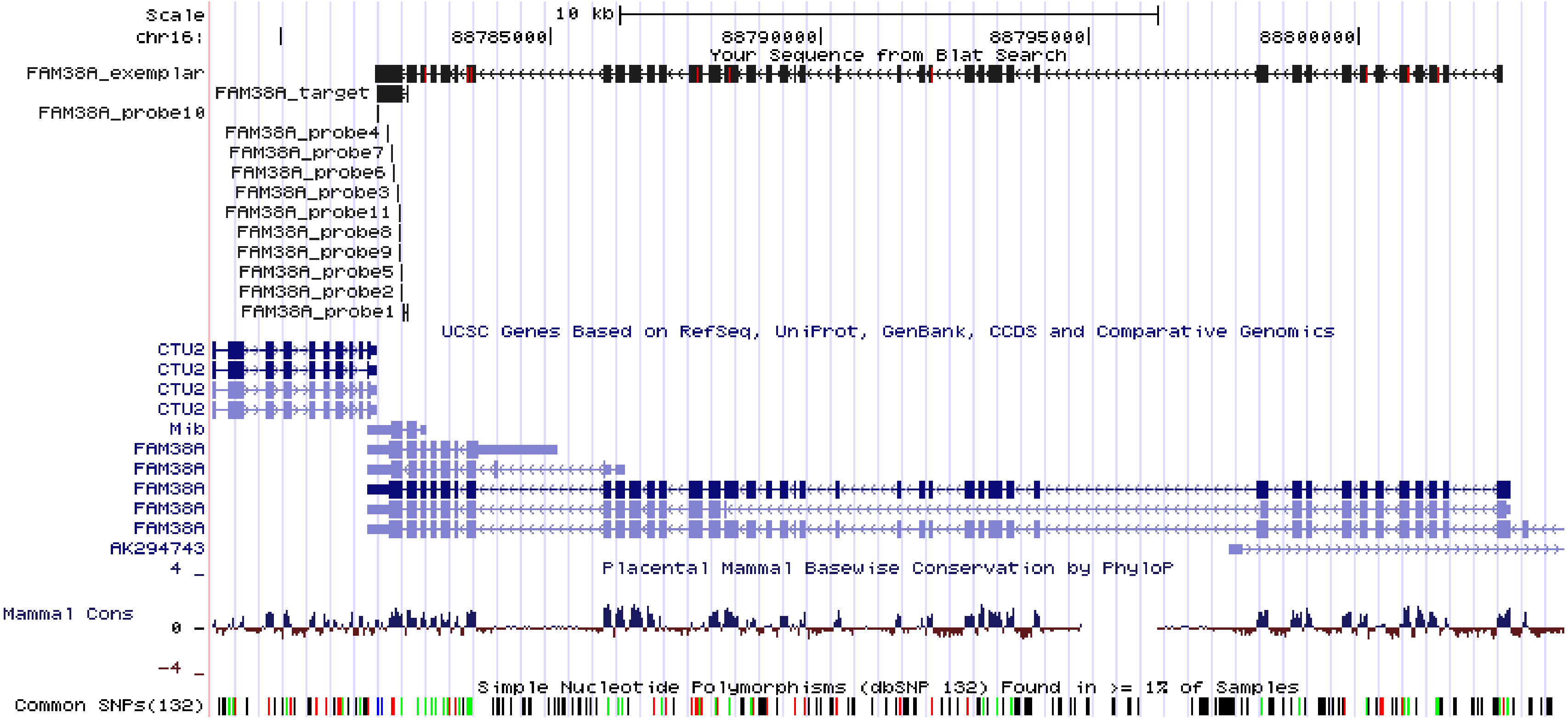

PLIN2 (209122_at)

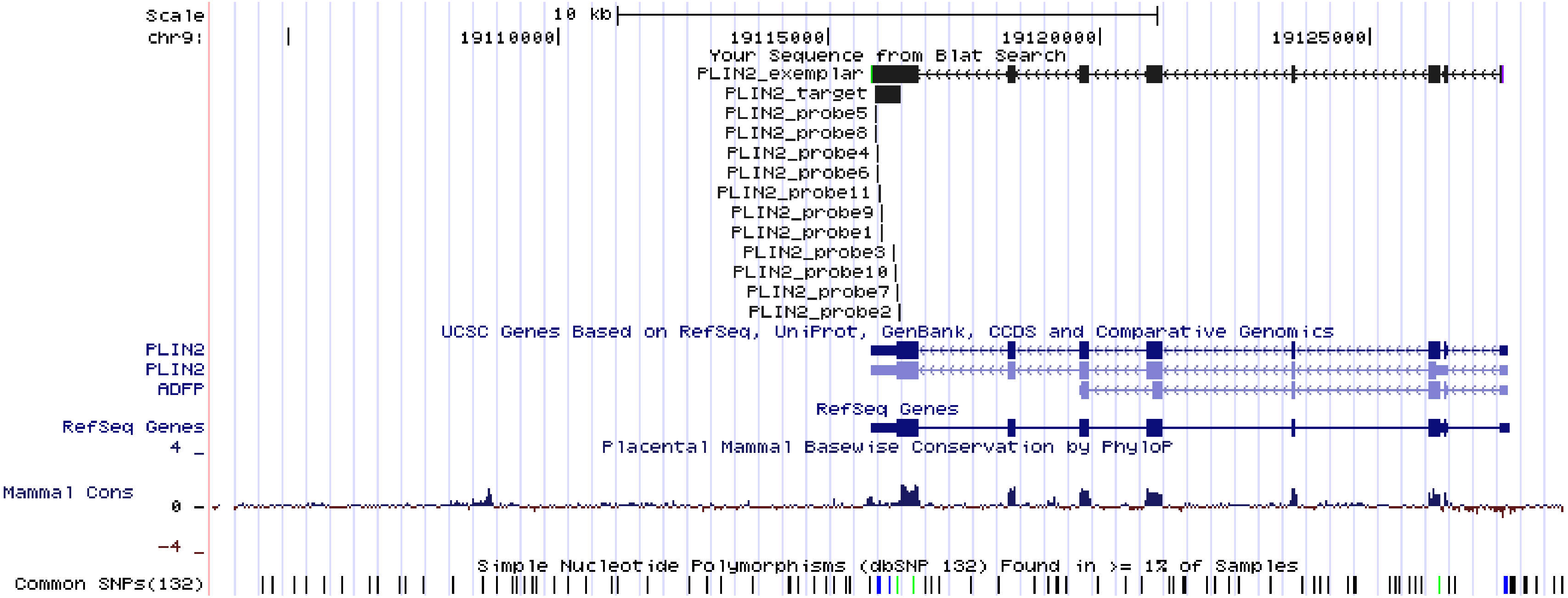

AIM1 (212543_at)

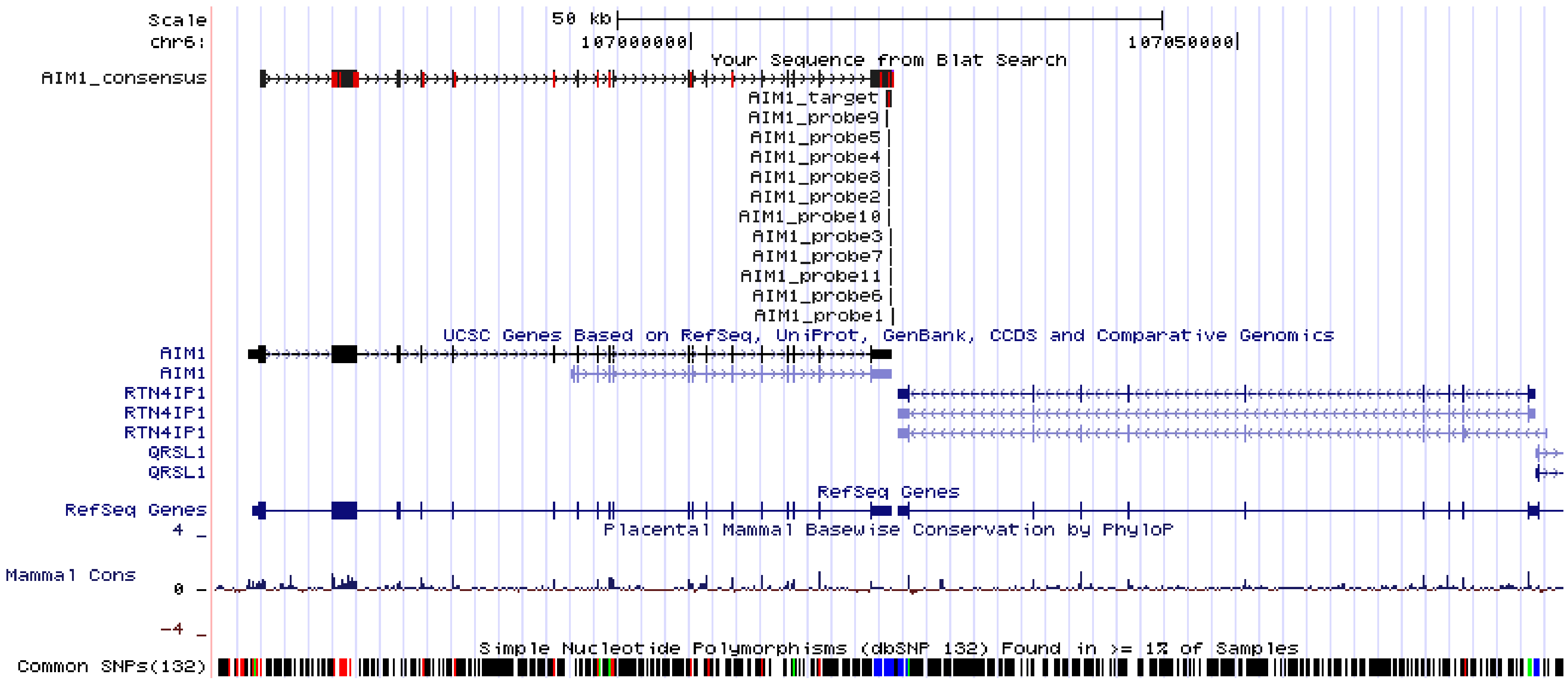

APOC1 (204416_x_at)

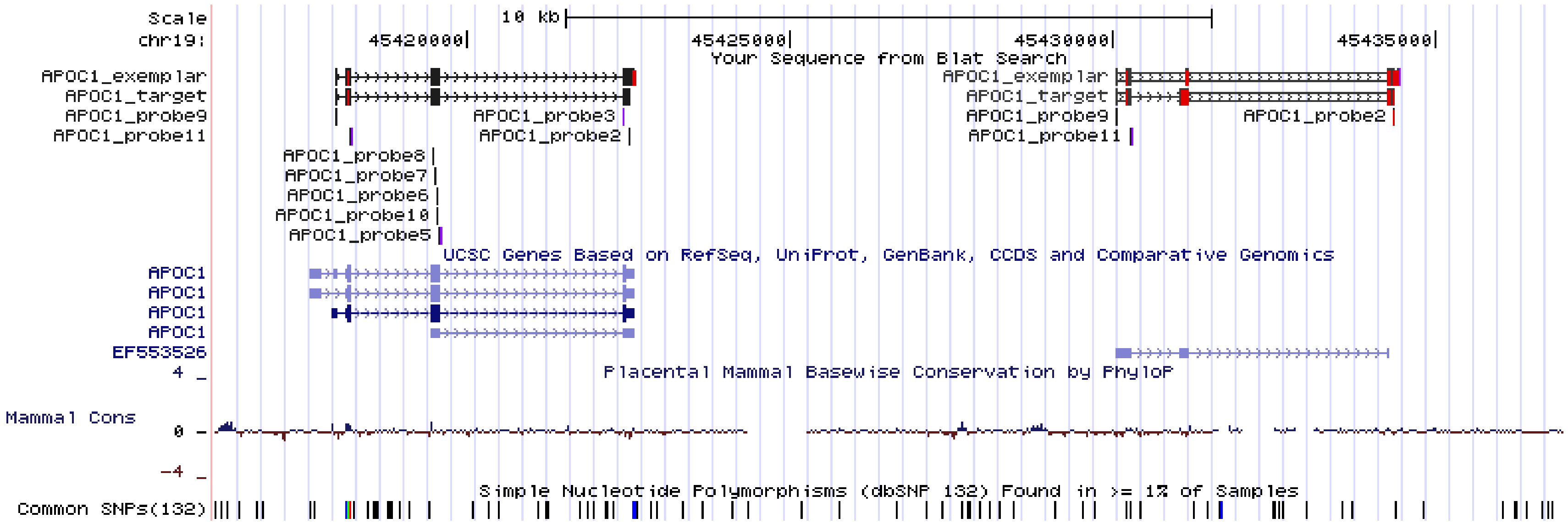

APOE (203382_s_at)

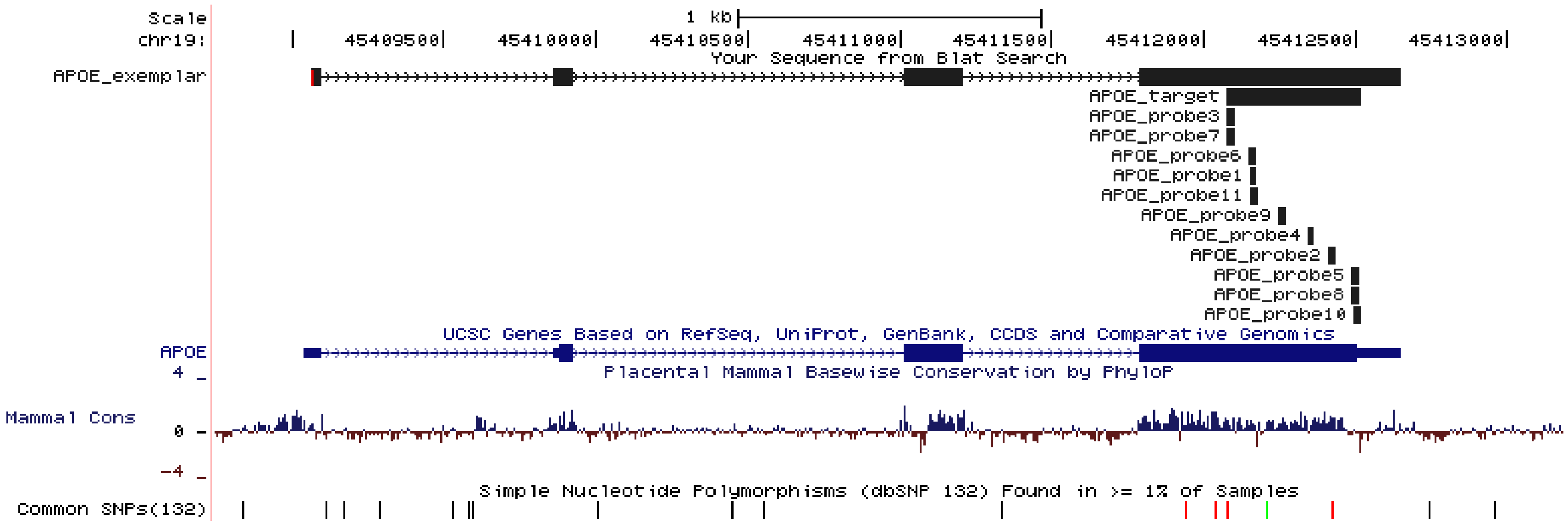

DTX4 (23220_at)

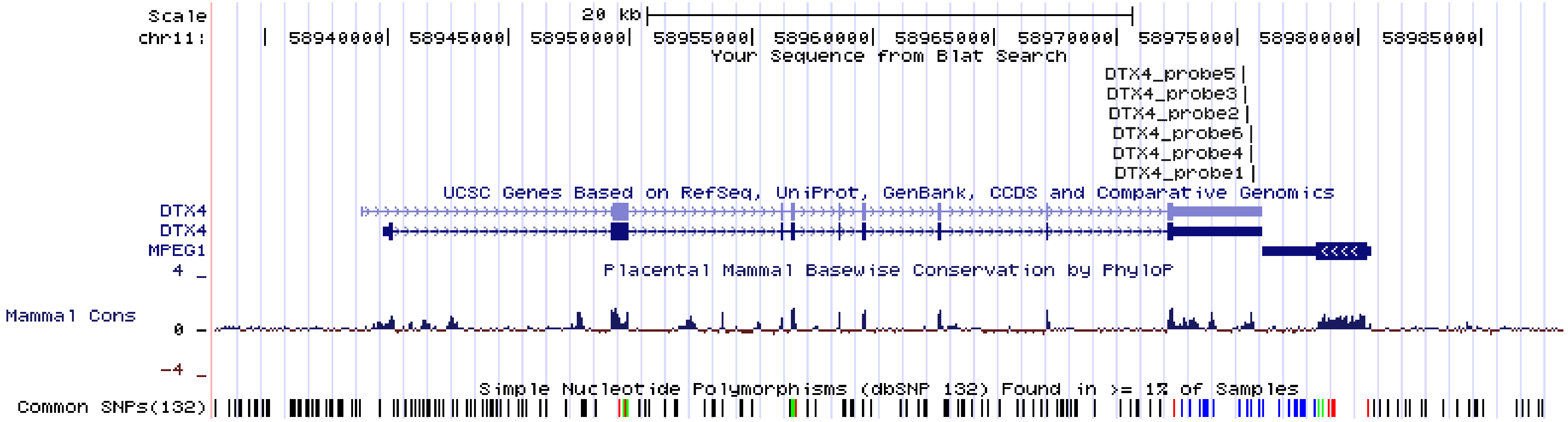

AQP1 (358_at)

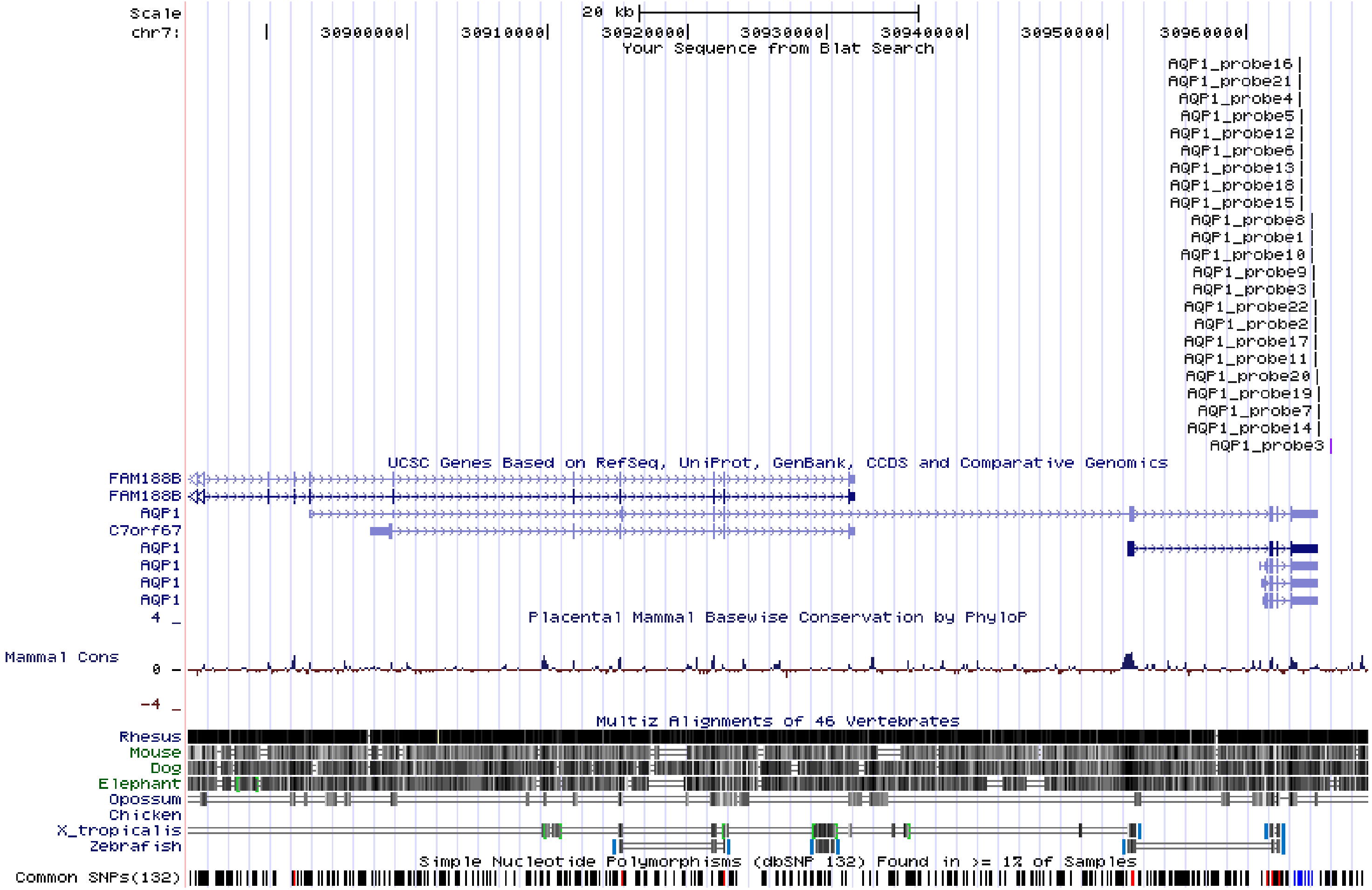
Scale
chr7:
20 kb
30900000
30910000
30920000
30930000
30940000
30950000
30960000
Your Sequence from Blat Search
AQP1_probe16
AQP1_probe21
AQP1_probe4
AQP1_probe5
AQP1_probe12
AQP1_probe6
AQP1_probe13
AQP1_probe18
AQP1_probe15
AQP1_probe8
AQP1_probe1
AQP1_probe10
AQP1_probe9
AQP1_probe3
AQP1_probe22
AQP1_probe2
AQP1_probe17
AQP1_probe11
AQP1_probe20
AQP1_probe19
AQP1_probe7
AQP1_probe14
AQP1_probe3
UCSC Genes Based on RefSeq, UniProt, GenBank, CCDS and Comparative Genomics
FAM188B
FAM188B
AQP1
C7orf67
AQP1
AQP1
AQP1
AQP1
Placental Mammal Basewise Conservation by PhyloP
4
Mammal Cons
0
-4
Multiz Alignments of 46 Vertebrates
Rhesus
Mouse
Dog
Elephant
Opossum
Chicken
X_tropicalis
Zebrafish
Simple Nucleotide Polymorphisms (dbSNP 132) Found in >= 1% of Samples
Common SNPs(132)

LMO4 (209205_s_at)

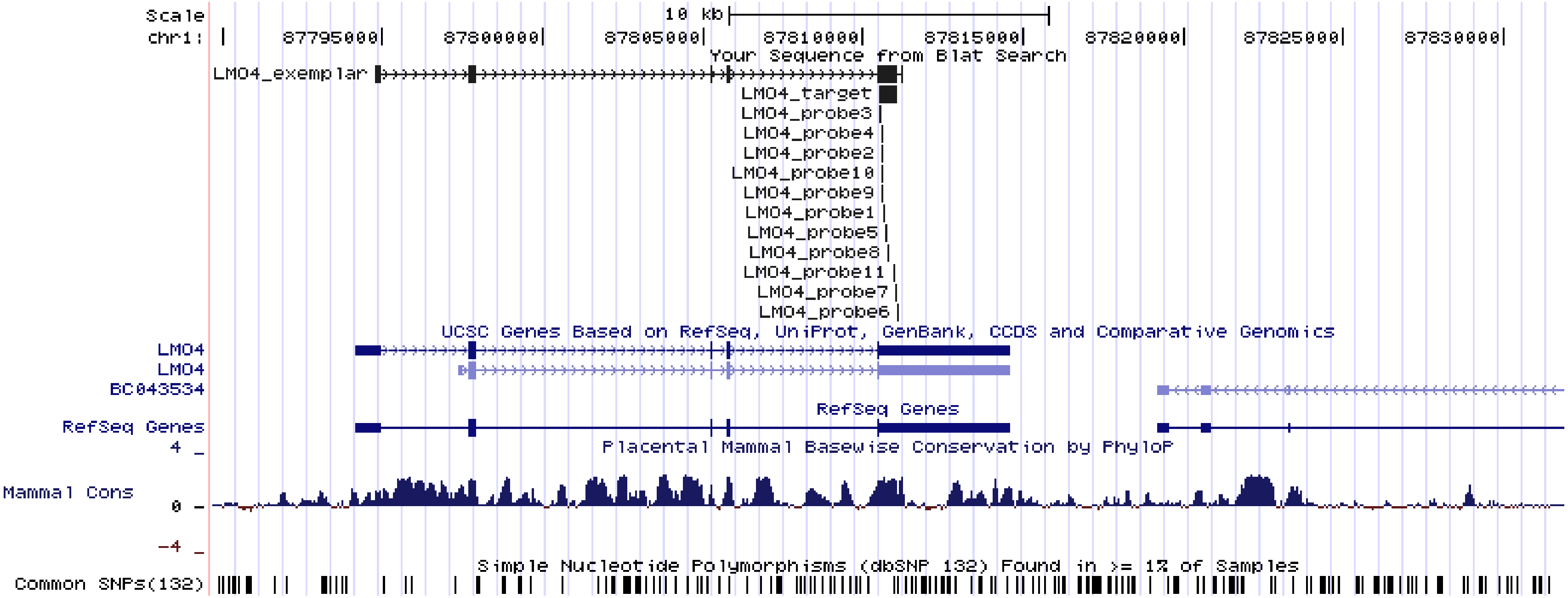

TAF1D (218750_at)

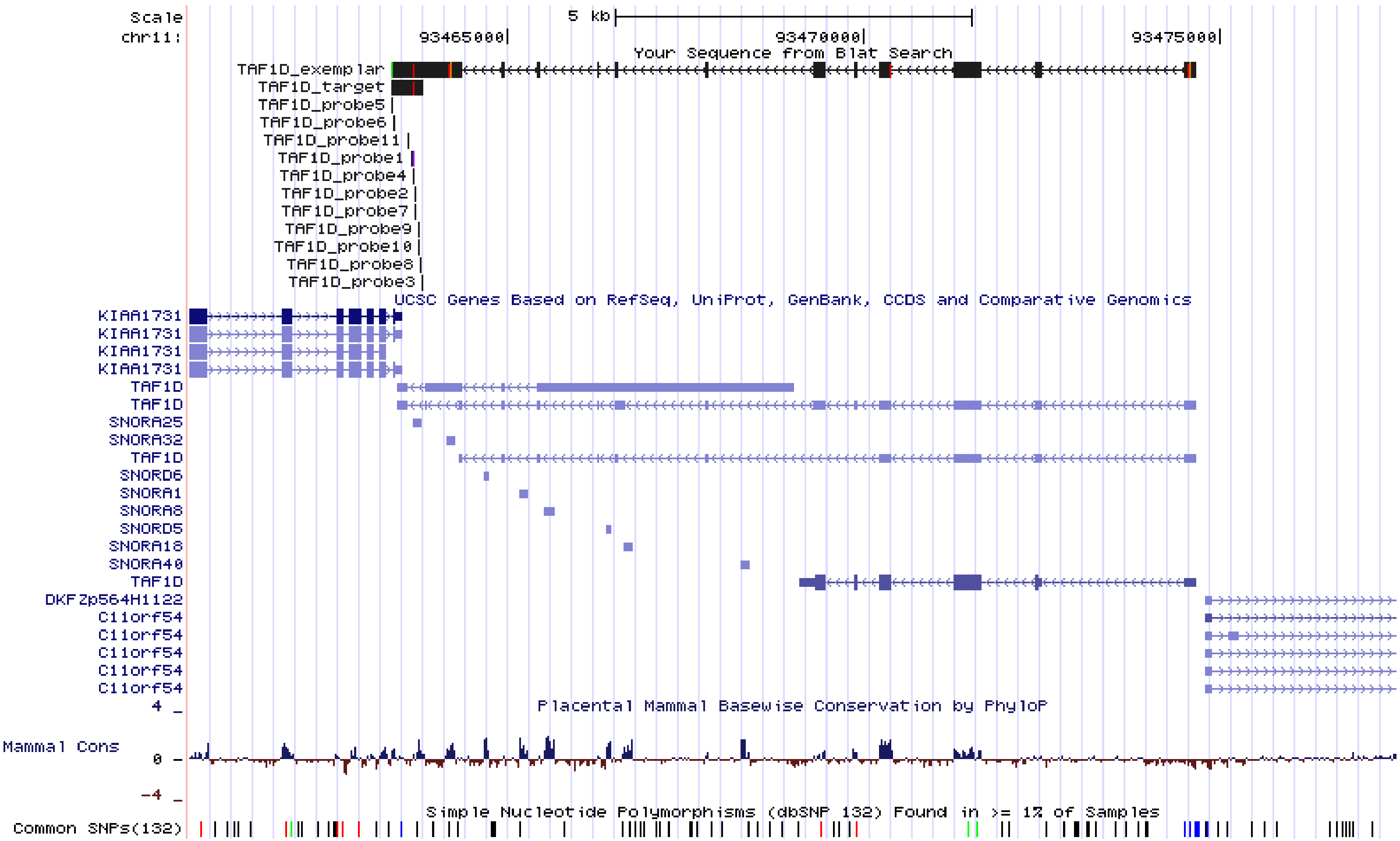

SNORA25 (684959_at)

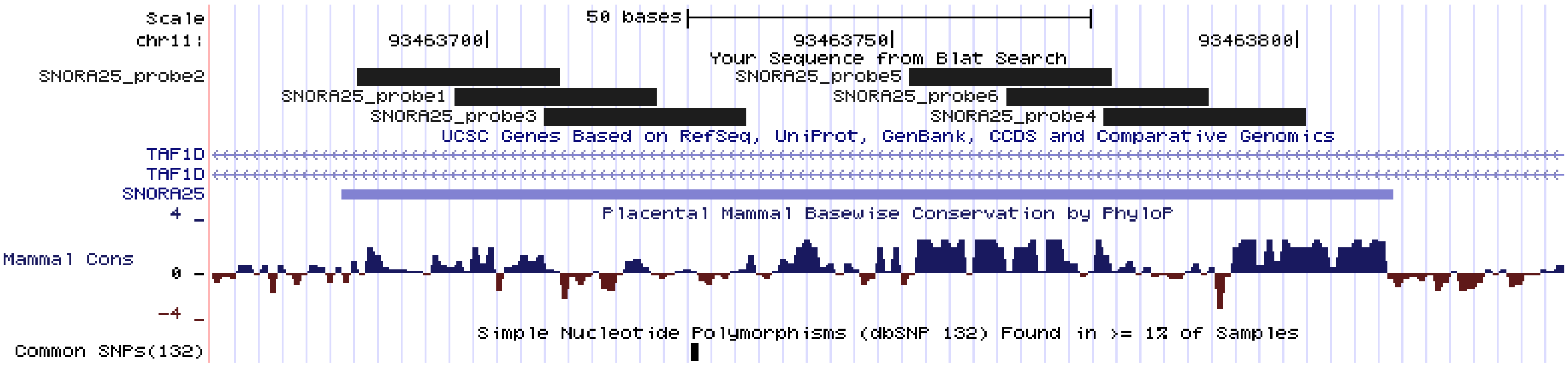

FMOD (202709_at)

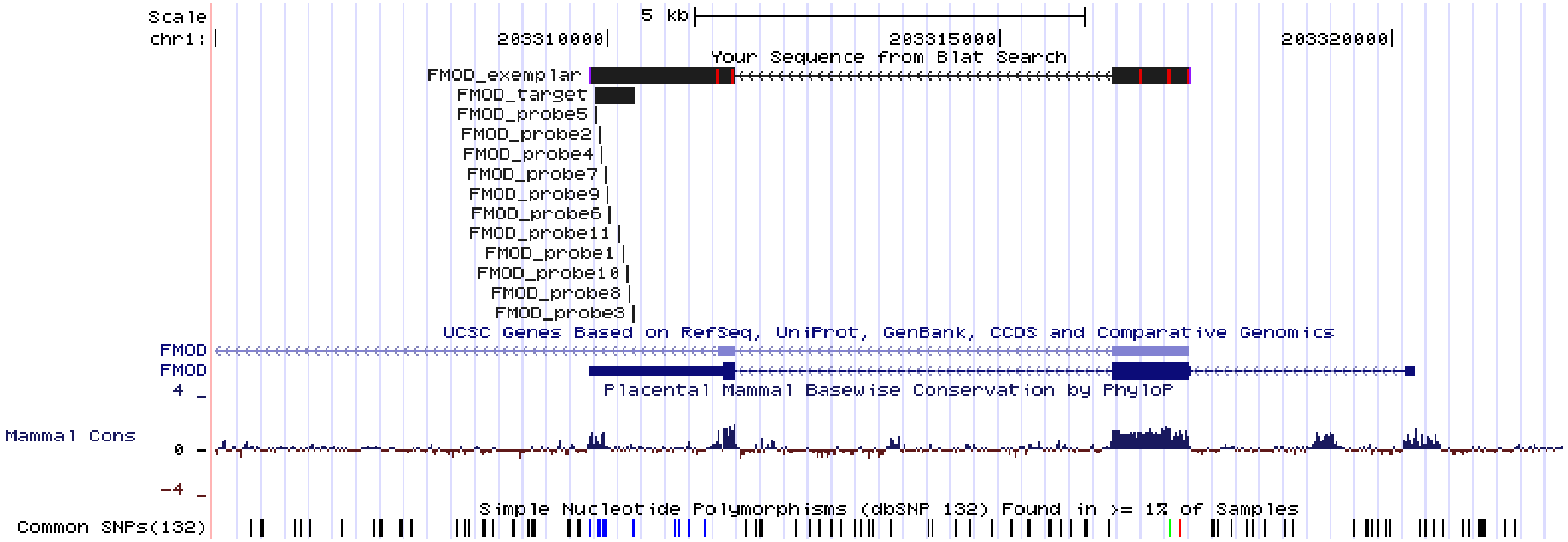

RGS5 (8490_at)

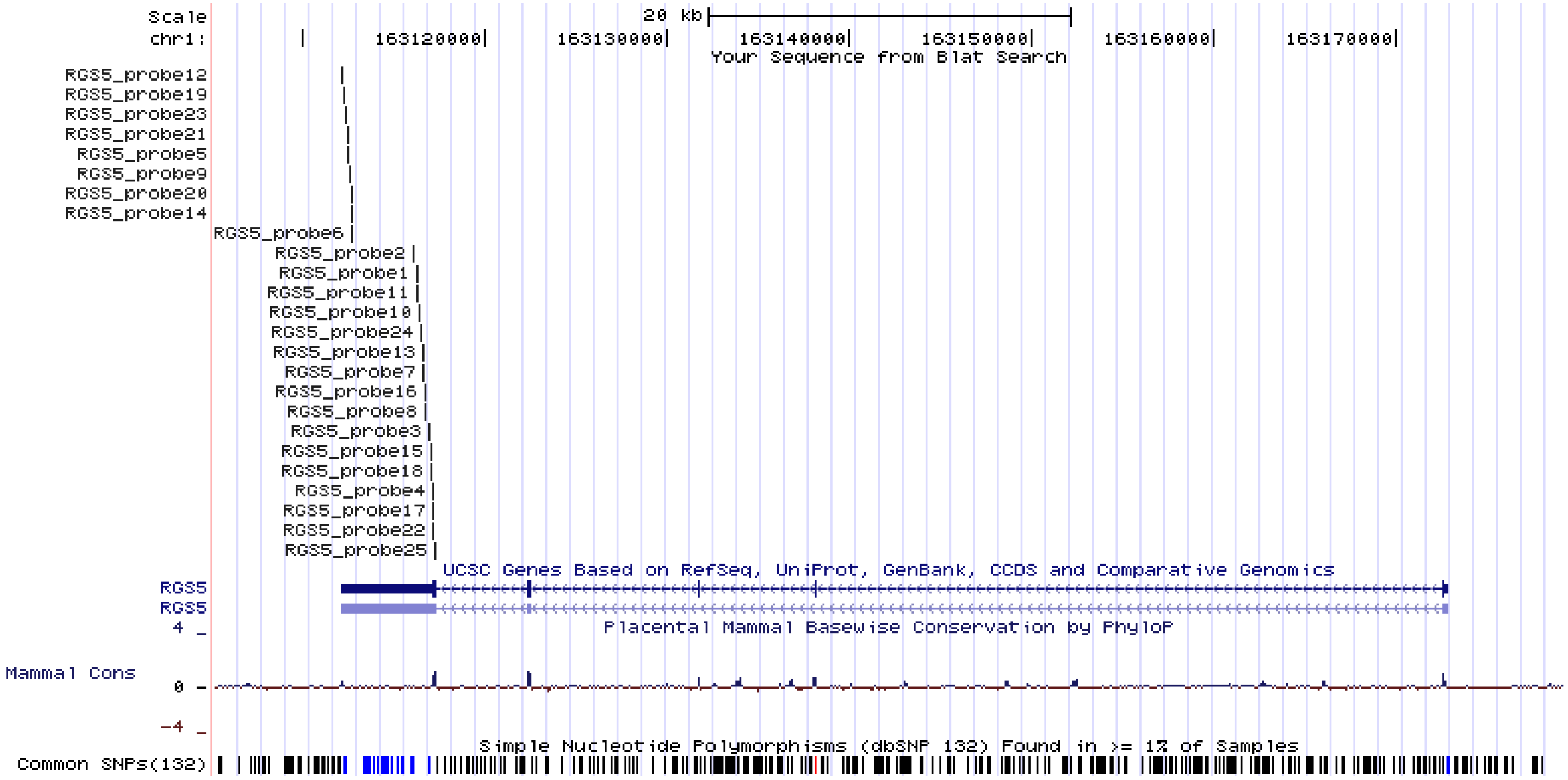
Scale
chr1:
20 kb
163120000
163130000
163140000
163150000
163160000
163170000
Your Sequence from Blat Search
RGS5_probe12
RGS5_probe19
RGS5_probe23
RGS5_probe21
RGS5_probe5
RGS5_probe9
RGS5_probe20
RGS5_probe14
RGS5_probe6
RGS5_probe2
RGS5_probe1
RGS5_probe11
RGS5_probe10
RGS5_probe24
RGS5_probe13
RGS5_probe7
RGS5_probe16
RGS5_probe8
RGS5_probe3
RGS5_probe15
RGS5_probe18
RGS5_probe4
RGS5_probe17
RGS5_probe22
RGS5_probe25
UCSC Genes Based on RefSeq, UniProt, GenBank, CCDS and Comparative Genomics
RGS5
RGS5
Placental Mammal Basewise Conservation by PhyloP
4
Mammal Cons
0
-4
Simple Nucleotide Polymorphisms (dbSNP 132) Found in >= 1% of Samples
Common SNPs(132)

PIK3R1 (212239_at)

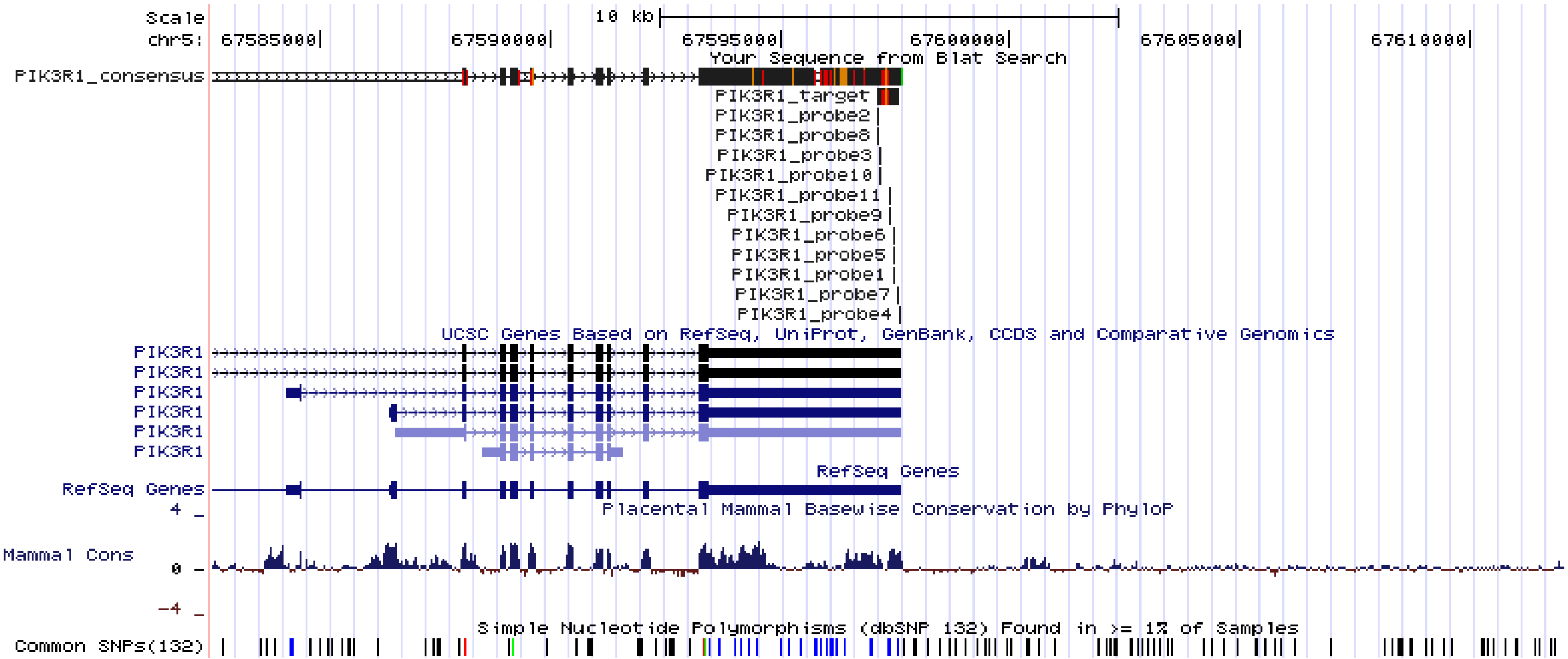

MBNL2 (203640_at)

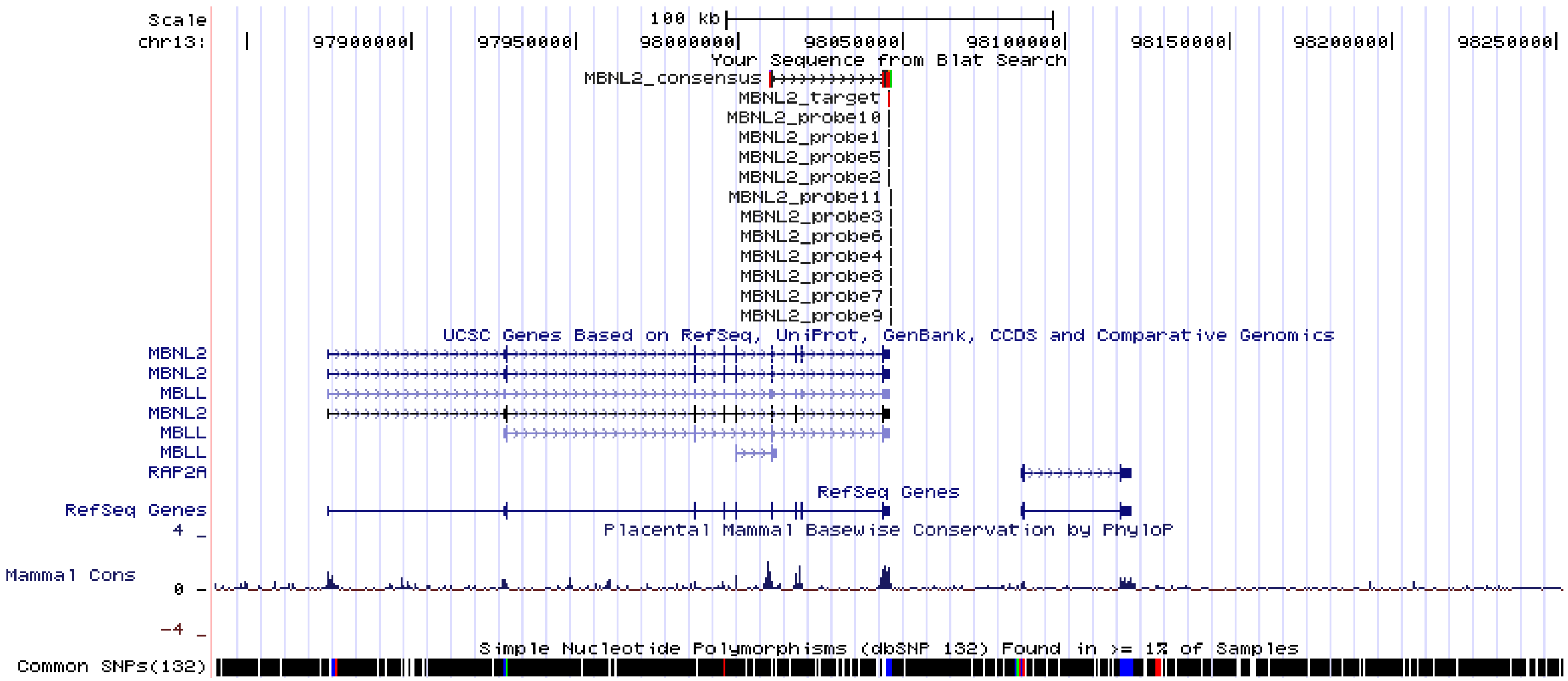

MAPKAPK2 (201461_s_at)

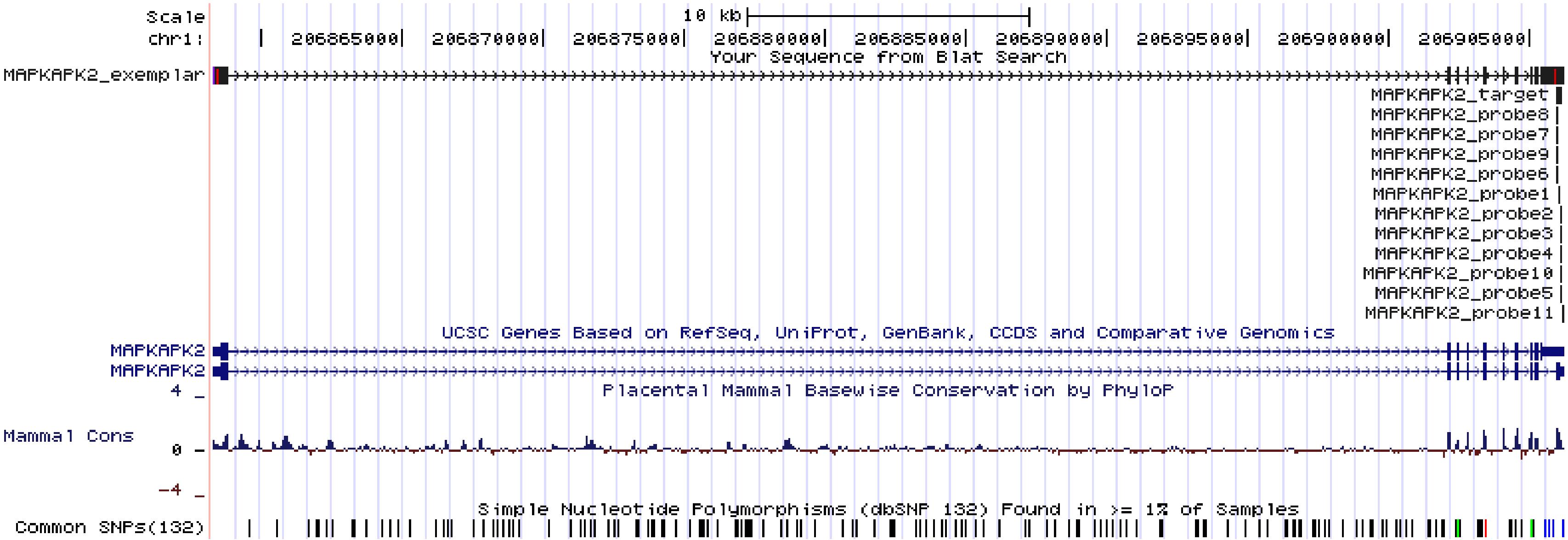

MTUS1 (212093_s_at)

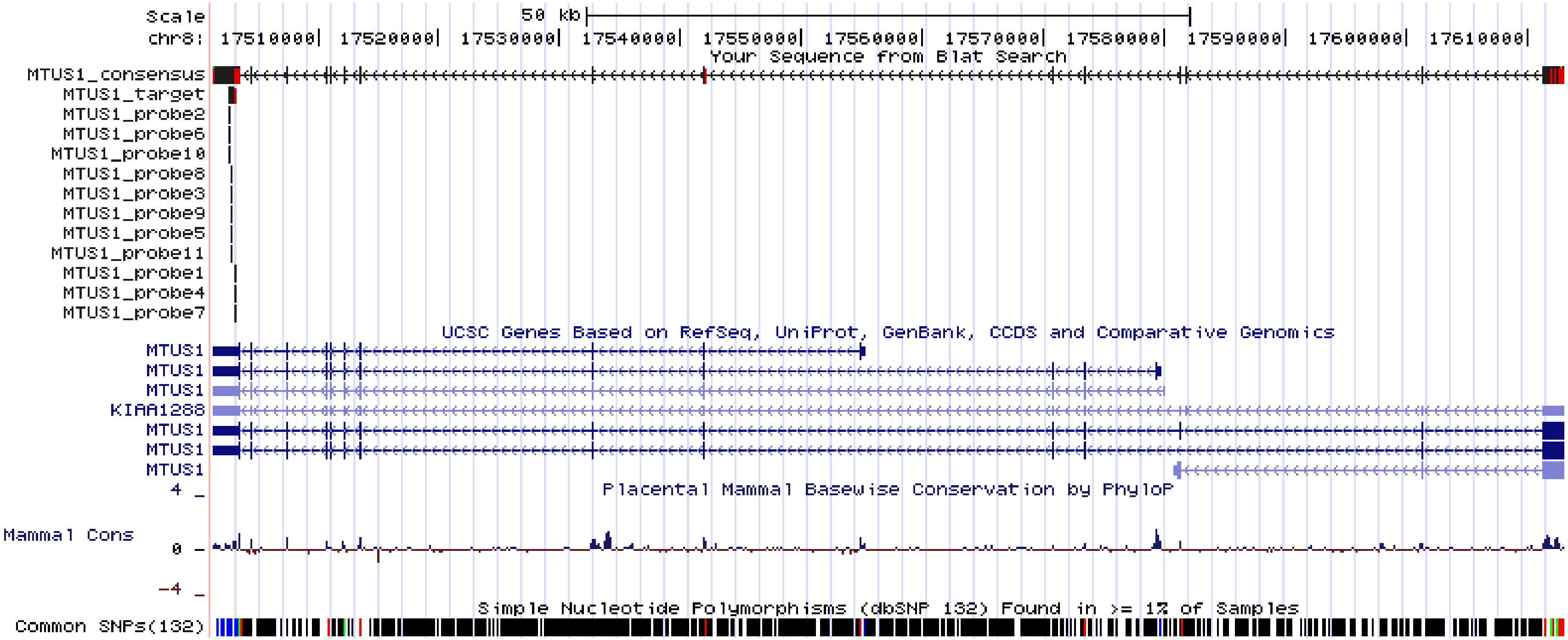

DHX9 (212107_s_at)

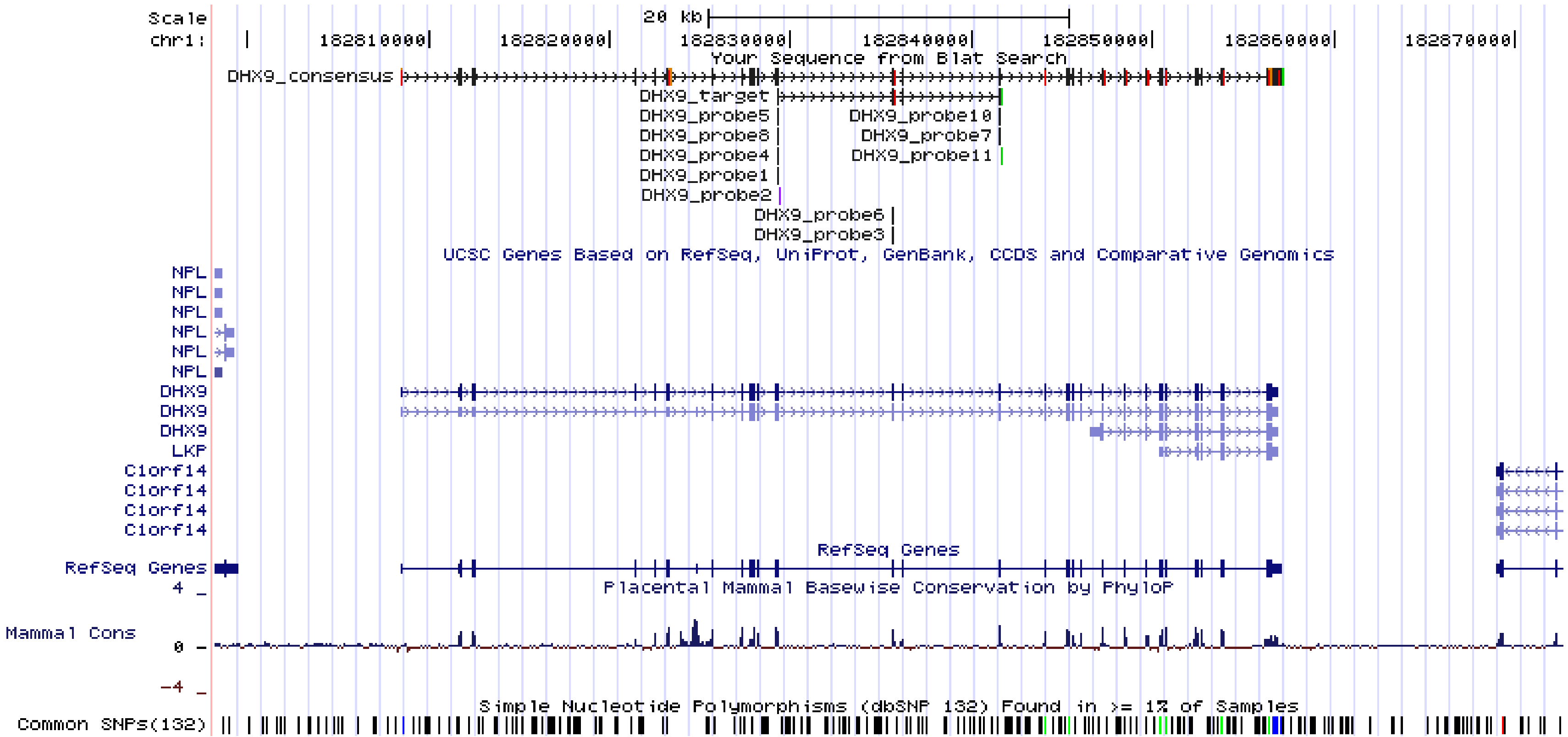

PPIF (201490_s_at)

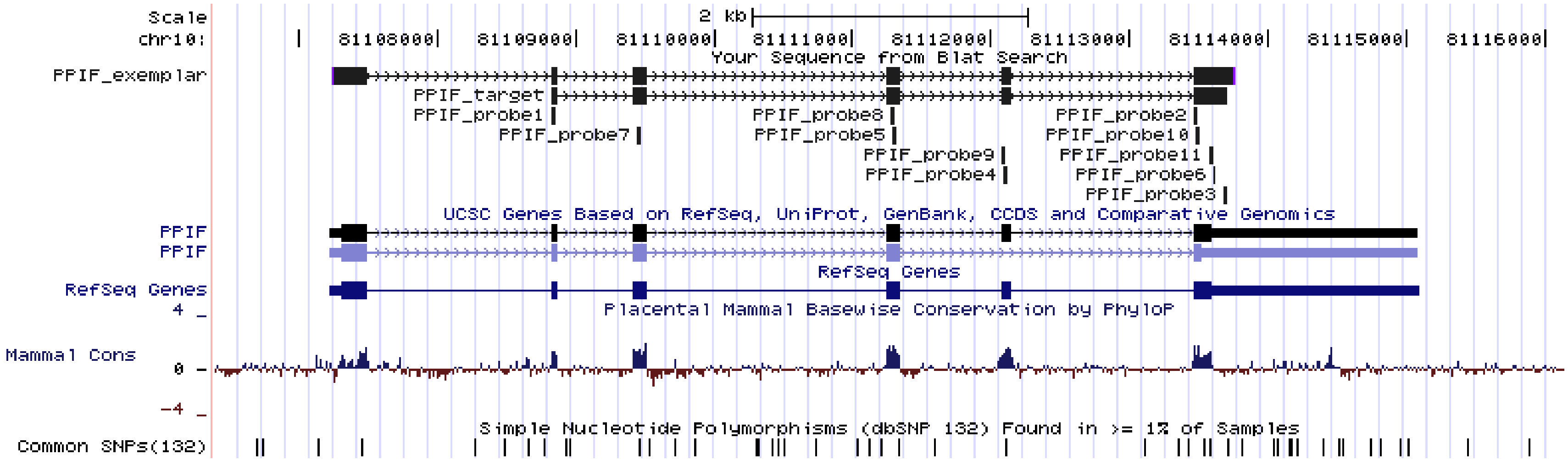

FOLR1 (211074_at)

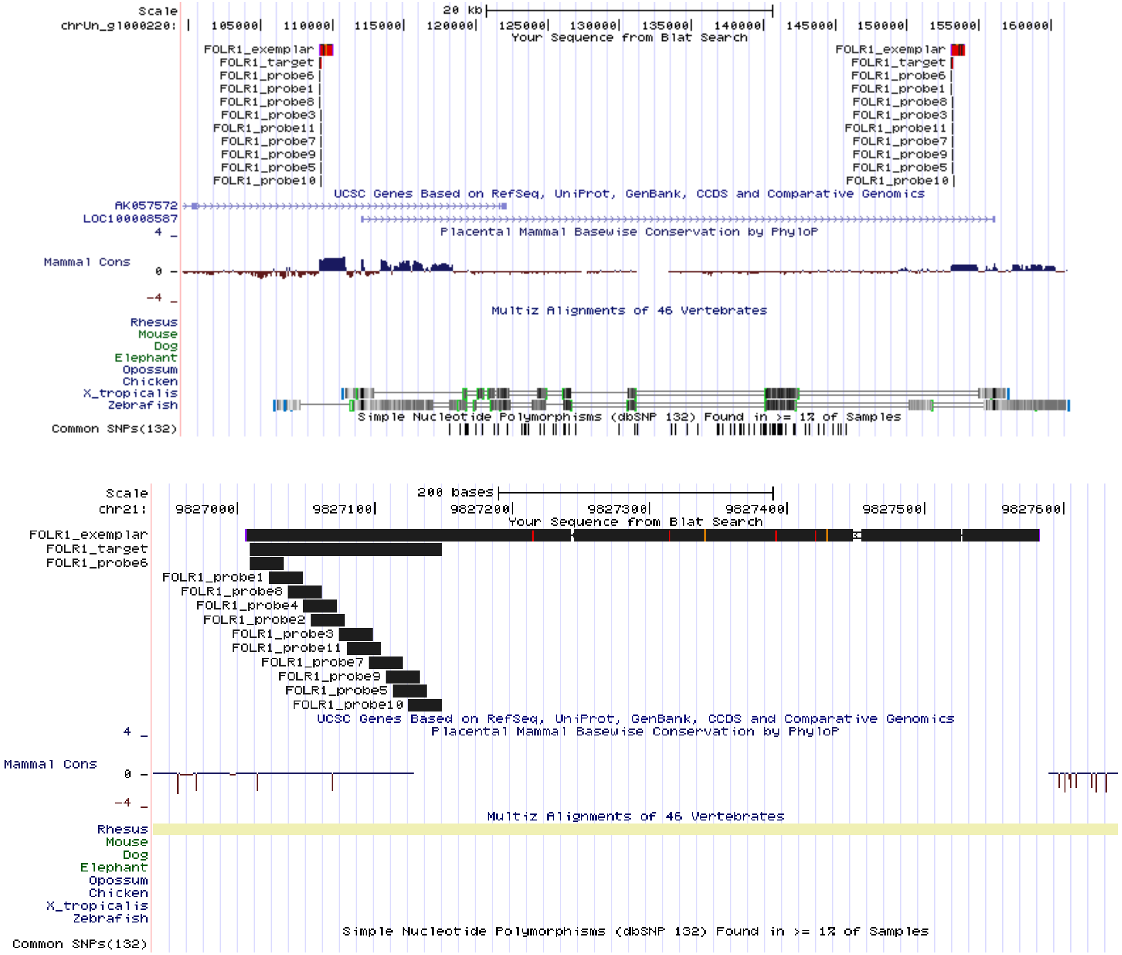

KIAA1467 (57613_at)

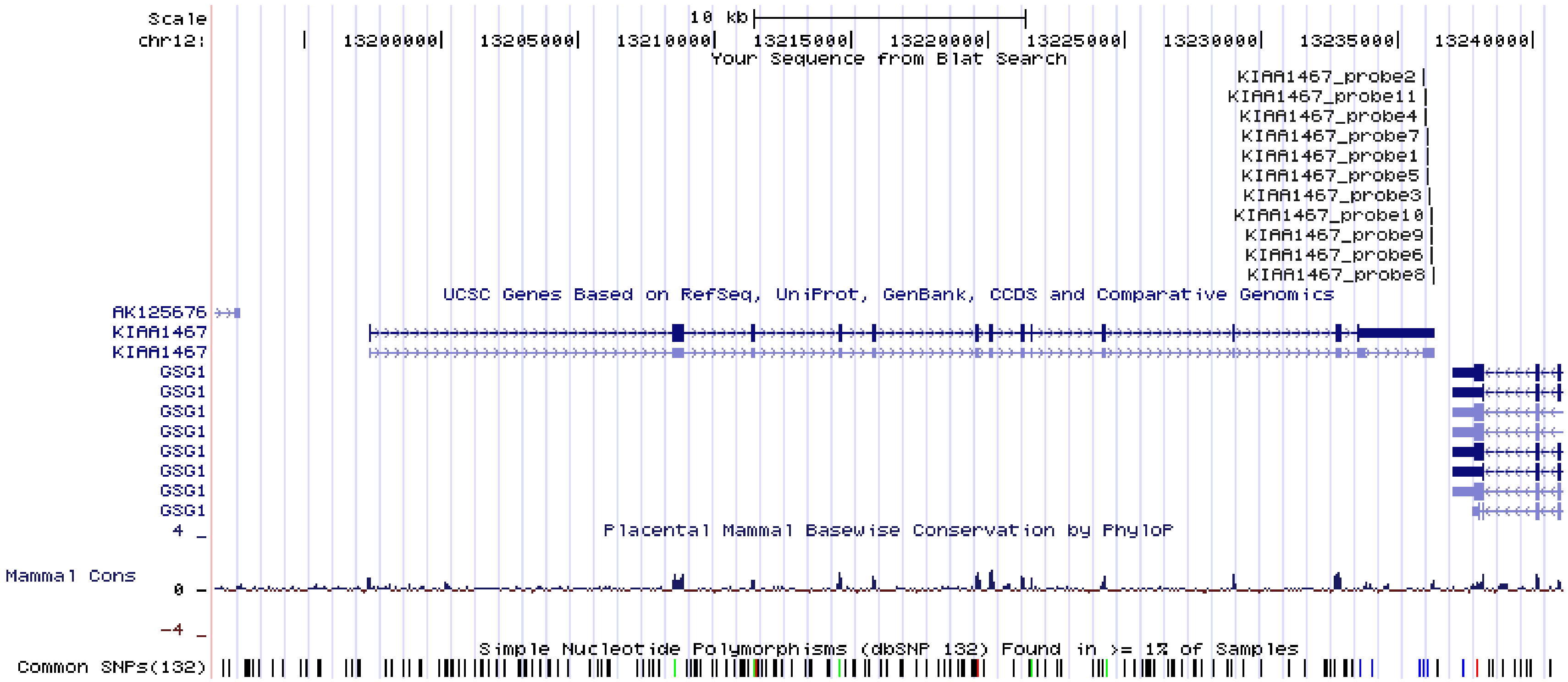

SUPT4H1 (201483_s_at)

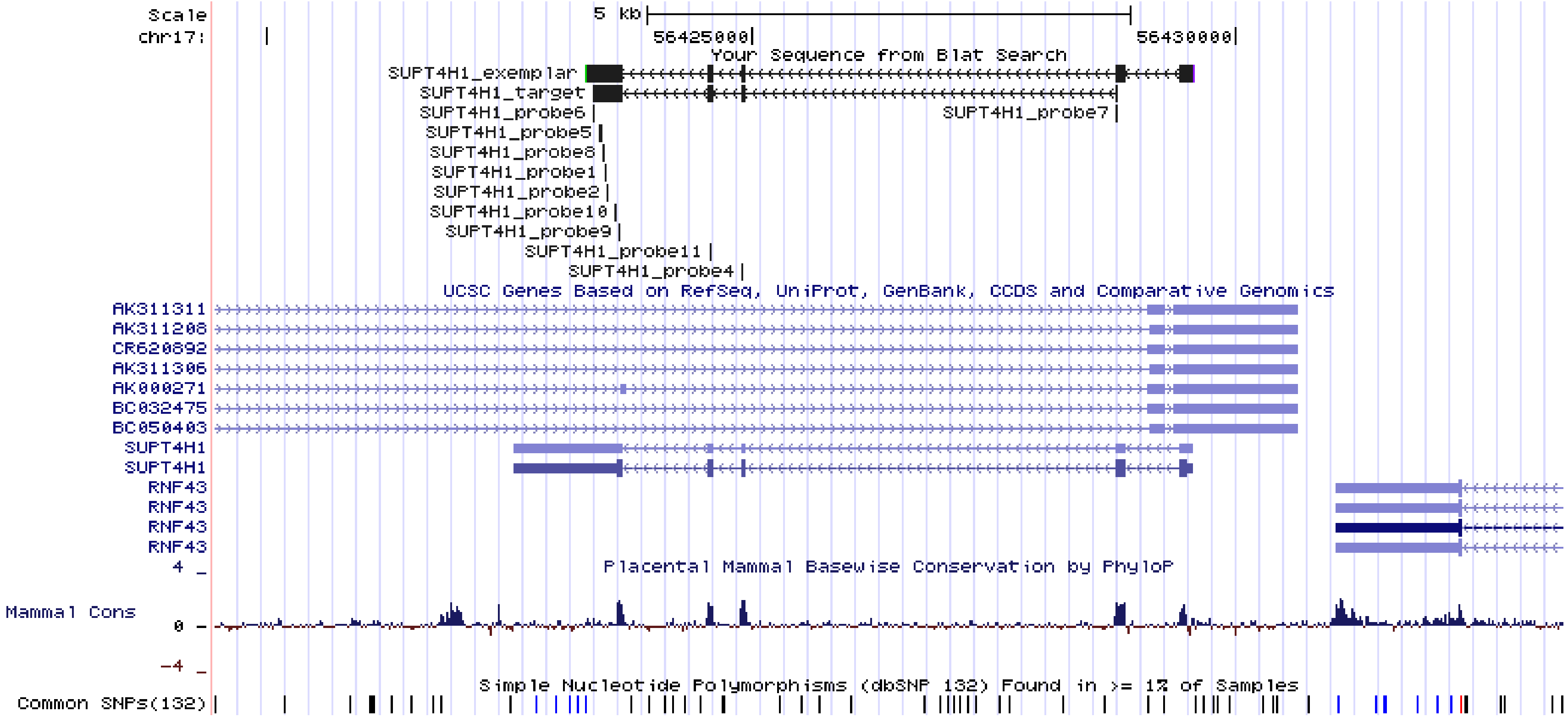

PHB (200658_s_at)

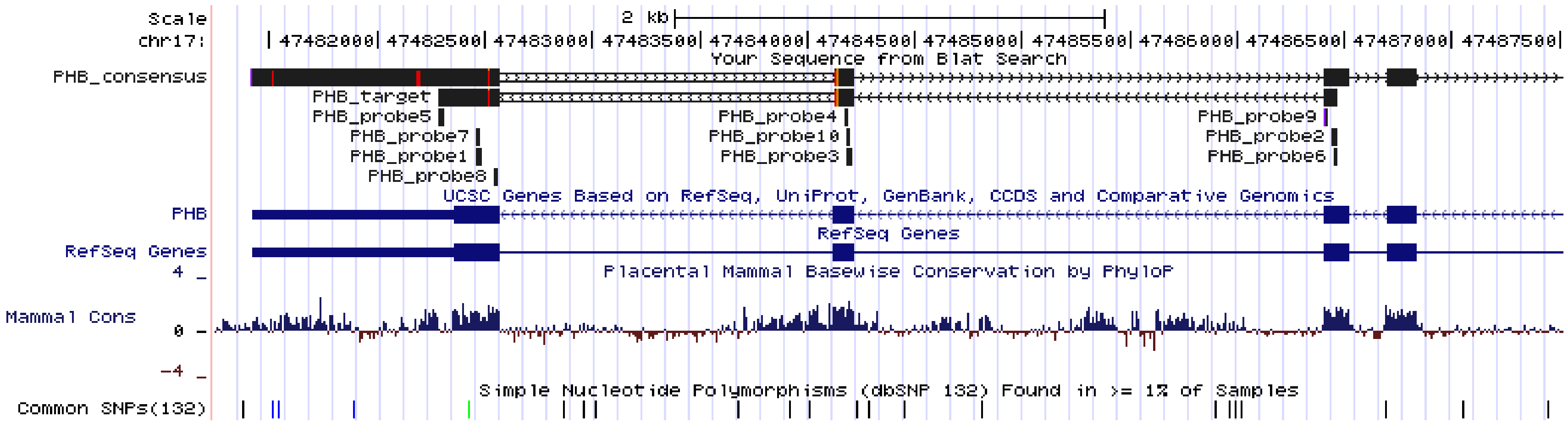

CD44 (204489_s_at)

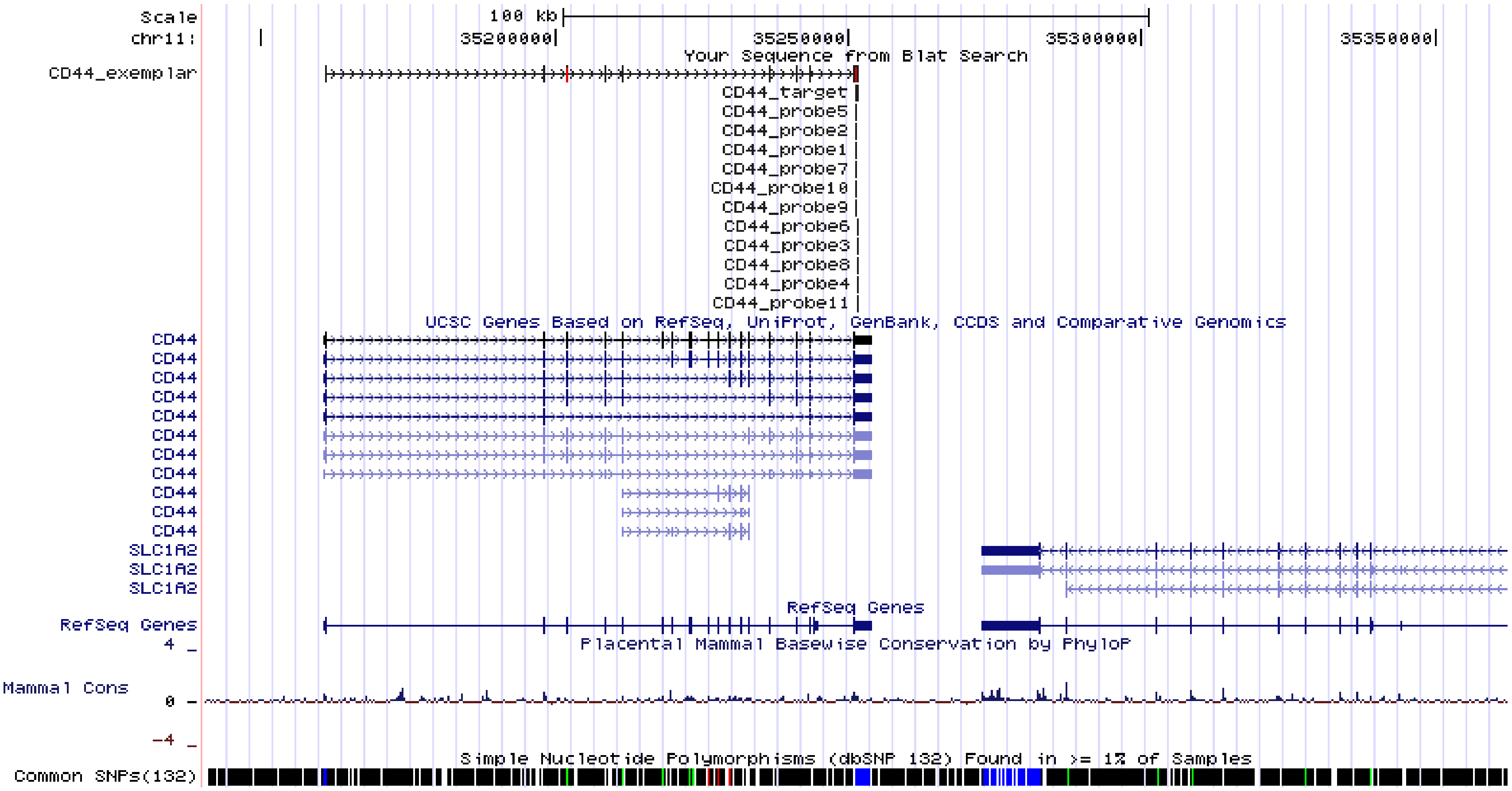

Supplement: Additional file 6 — Describes alignment of the top 100 probe sets to the reference genome. [file gm496-S6.zip › Addfile 6 and 7 (5)/1217714361945551_add7.pdf]
