# Supplementary material for: A robust prognostic signature for hormone-positive node-negative breast cancer
Source: Genome Med. 2013 Oct 11;5(10):92. doi: 10.1186/gm496 (PMC3961800; doi:10.1186/gm496)
Supplement: Additional file 7 — Contains actual probe sequences for the top 100 probe sets. [file gm496-S7.docx]

**Appendix 4. Probe sequences for top 100 probesets**>CCNB2_probe1

ATGGAGCTGACTCTCATCGACTATG

>CCNB2_probe2

ATATGGTGCATTATCATCCTTCTAA

>CCNB2_probe3

AGTCCTCTGGTCTATCTCATGAAAC

>CCNB2_probe4

CTTGCCTCCCCACTGATAGGAAGGT

>CCNB2_probe5

CAAAAGCCGTCAAAGACCTTGCCTC

>CCNB2_probe6

GATTTTGTACATAGTCCTCTGGTCT

>CCNB2_probe7

GCCACTACACTTCTTAAGGCGAGCA

>CCNB2_probe8

GATAGGAAGGTCCTAGGCTGCCGTG

>CCNB2_probe9

ATCCTTCTAAGGTAGCAGCAGCTGC

>TOP2A_probe1

ACTCCGTAACAGATTCTGGACCAAC

>TOP2A_probe2

GACCAACCTTCAACTATCTTCTTGA

>TOP2A_probe3

GAAAGATGAACTCTGCAGGCTAAGA

>TOP2A_probe4

ACAAGATGAACAAGTCGGACTTCCT

>TOP2A_probe5

TGGCTCCTAGGAATGCTTGGTGCTG

>TOP2A_probe6

GATATGATTCGGATCCTGTGAAGGC

>TOP2A_probe7

AAAGAAAGAGTCCATCAGATTTGTG

>TOP2A_probe8

GAATAATCAGGCTCGCTTTATCTTA

>TOP2A_probe9

CTTGGTGCTGAATCTGCTAAACTGA

>TOP2A_probe10

AAGAACAAGAGCTGGACACATTAAA

>TOP2A_probe11

GAGACTTTTTTGAACTCAGACTTAA

>TOP2A_target

gagacttttttgaactcagacttaaatattatggattaagaaaagaatggctcctaggaatgcttggtgctgaatctgctaaactgaataatcaggctcgctttatcttagagaaaatagatggcaaaataatcattgaaaataagcctaagaaagaattaattaaagttctgattcagaggggatatgattcggatcctgtgaaggcctggaaagaagcccagcaaaaggttccagatgaagaagaaaatgaagagagtgacaacgaaaaggaaactgaaaagagtgactccgtaacagattctggaccaaccttcaactatcttcttgatatgcccctttggtatttaaccaaggaaaagaaagatgaactctgcaggctaagaaatgaaaaagaacaagagctggacacattaaaaagaaagagtccatcagatttgtggaaagaagacttggctacatttattgaagaattggaggctgttgaagccaaggaaaaacaagatgaacaagtcggacttcct

>TOP2A_consensus

ctaaccgacgcgcgtctgtggagaagcggcttggtcgggggtggtctcgtggggtcctgcctgtttagtcgnnnncannnnnnnnnnnncccttcacgaccgtcaccatggaagtgtcaccattgcagcctgtaaatgaaaatatgcaagtcaacaaaataaagaaaaatgaagatgctaagaaaagactgtctgttgaaagaatctatcaaaagaaaacacaattggaacatattttgctccgcccagacacctacattggttctgtggaattagtgacccagcaaatgtgggtttacgatgaagatgttggcattaactatagggaagtcacttttgttcctggtttgtacaaaatctttgatgagattctagttaatgctgcggacaacaaacaaagggacccaaaaatgtcttgtattagagtcacaattgatccggaaaacaatttaattagtatatggaataatggaaaaggtattcctgttgttgaacacaaagttgaaaagatgtatgtcccagctctcatatttggacagctcctaacttctagtaactatgatgatgatgaaaagaaagtgacaggtggtcgaaatggctatggagccaaattgtgtaacatattcagtaccaaatttactgtggaaacagccagtagagaatacaagaaaatgttcaaacagacatggatggataatatgggaagagctggtgagatggaactcaagcccttcaatggagaagattatacatgtatcacctttcagcctgatttgtctaagtttaaaatgcaaagcctggacaaagatattgttgcactaatggtcagaagagcatatgatattgctggatccaccaaagatgtcaaagtctttcttaatggaaataaactgccagtaaaaggatttcgtagttatgtggacatgtatttgaaggacaagttggatgaaactggtaactccttgaaagtaatacatgaacaagtaaaccacaggtgggaagtgtgtttaactatgagtgaaaaaggctttcagcaaattagctttgtcaacagcattgctacatccaagggtggcagacatgttgattatgtagctgatcagattgtgactaaacttgttgatgttgtgaagaagaagaacaagggtggtgttgcagtaaaagcacatcaggtgaaaaatcacatgtggatttttgtaaatgccttaattgaaaacccaacctttgactctcagacaaaagaaaacatgactttacaacccaagagctttggatcaacatgccaattgagtgaaaaatttatcaaagctgccattggctgtggtattgtagaaagcatactaaactgggtgaagtttaaggcccaagtccagttaaacaagaagtgttcagctgtaaaacataatagaatcaagggaattcccaaactcgatgatgccaatgatgcagggggccgaaactccactgagtgtacgcttatcctgactgagggagattcagccaaaactttggctgtttcaggccttggtgtggttgggagagacaaatatggggttttccctcttagaggaaaaatactcaatgttcgagaagcttctcataagcagatcatggaaaatgctgagattaacaatatcatcaagattgtgggtcttcagtacaagaaaaactatgaagatgaagattcattgaagacgcttcgttatgggaagataatgattatgacagatcaggaccaagatggttcccacatcaaaggcttgctgattaattttatccatcacaactggccctctcttctgcgacatcgttttctggaggaatttatcactcccattgtaaaggtatctaaaaacaagcaagaaatggcattttacagccttcctgaatttgaagagtggaagagttctactccaaatcataaaaaatggaaagtcaaatattacaaaggtttgggcaccagcacatcaaaggaagctaaagaatactttgcagatatgaaaagacatcgtatccagttcaaatattctggtcctgaagatgatgctgctatcagcctggcctttagcaaaaaacagatagatgatcgaaaggaatggttaactaatttcatggaggatagaagacaacgaaagttacttgggcttcctgaggattacttgtatggacaaactaccacatatctgacatataatgacttcatcaacaaggaacttatcttgttctcaaattctgataacgagagatctatcccttctatggtggatggtttgaaaccaggtcagagaaaggttttgtttacttgcttcaaacggaatgacaagcgagaagtaaaggttgcccaattagctggatcagtggctgaaatgtcttcttatcatcatggtgagatgtcactaatgatgaccattatcaatttggctcagaattttgtgggtagcaataatctaaacctcttgcagcccattggtcagtttggtaccaggctacatggtggcaaggattctgctagtccacgatacatctttacaatgctcagctctttggctcgattgttatttccaccaaaagatgatcacacgttgaagtttttatatgatgacaaccagcgtgttgagcctgaatggtacattcctattattcccatggtgctgataaatggtgctgaaggaatcggtactgggtggtcctgcaaaatccccaactttgatgtgcgtgaaattgtaaataacatcaggcgtttgatggatggagaagaacctttgccaatgcttccaagttacaagaacttcaagggtactattgaagaactggctccaaatcaatatgtgattagtggtgaagtagctattcttaattctacaaccattgaaatctcagagcttcccgtcagaacatggacccagacatacaaagaacaagttctagaacccatgttgaatggcaccgagaagacacctcctctcataacagactatagggaataccatacagataccactgtgaaatttgttgtgaagatgactgaagaaaaactggcagaggcagagagagttggactacacaaagtcttcaaactccaaactagtctcacatgcaactctatggtgctttttgaccacgtaggctgtttaaagaaatatgacacggtgttggatattctaagagacttttttgaactcagacttaaatattatggattaagaaaagaatggctcctaggaatgcttggtgctgaatctgctaaactgaataatcaggctcgctttatcttagagaaaatagatggcaaaataatcattgaaaataagcctaagaaagaattaattaaagttctgattcagaggggatatgattcggatcctgtgaaggcctggaaagaagcccagcaaaaggttccagatgaagaagaaaatgaagagagtgacaacgaaaaggaaactgaaaagagtgactccgtaacagattctggaccaaccttcaactatcttcttgatatgcccctttggtatttaaccaaggaaaagaaagatgaactctgcaggctaagaaatgaaaaagaacaagagctggacacattaaaaagaaagagtccatcagatttgtggaaagaagacttggctacatttattgaagaattggaggctgttgaagccaaggaaaaacaagatgaacaagtcggacttcctgggaaaggggggaaggccaaggggaaaaaaacacaaatggctgaagttttgccttctccgcgtggtcaaagagtcattccacgaataaccatagaaatgaaagcagaggcagaaaagaaaaataaaaagaaaattaagaatgaaaatactgaaggaagccctcaagaagatggtgtggaactagaaggcctaaaacaaagattagaaaagaaacagaaaagagaaccaggtacaaagacaaagaaacaaactacattggcatttaagccaatcaaaaaaggaaagaagagaaatccctggtctgattcagaatcagataggagcagtgacgaaagtaattttgatgtccctccacgagaaacagagccacggagagcagcaacaaaaacaaaattcacaatggatttggattcagatgaagatttctcagattttgatgaaaaaactgatgatgaagattttgtcccatcagatgctagtccacctaagaccaaaacttccccaaaacttagtaacaaagaactgaaaccacagaaaagtgtcgtgtcagaccttgaagctgatgatgttaagggcagtgtaccactgtcttcaagccctcctgctacacatttcccagatgaaactgaaattacaaacccagttcctaaaaagaatgtgacagtgaagaagacagcagcaaaaagtcagtcttccacctccactaccggtgccaaaaaaagggctgccccaaaaggaactaaaagggatccagctttgaattctggtgtctctcaaaagcctgatcctgccaaaaccaagaatcgccgcaaaaggaagccatccacttctgatgattctgactctaattttgagaaaattgtttcgaaagcagtcacaagcaagaaatccaagggggagagtgatgacttccatatggactttgactcagctgtggctcctcgggcaaaatctgtacgggcaaagaaacctataaagtacctggaagagtcagatgaagatgatctgttttaaaatgtgaggcgattattttaagtaattatcttaccaagcccaagactggttttaaagttacctgaagctcttaacttcctcccctctgaatttagtttggggaaggtgtttttagtacaagacatcaaagtgaagtaaagcccaagtgttctttagctttttataatactgtctaaatagtgaccatctcatgggcatngttttcttctctgcttngtctgtgtttngagtctgctttctttngtctttaaaacctgatttttaagttcttctgaactgtagaaatagctatctgatcacttcagcgtaaagcagtgtgtttattaaccatccactaagctaaaactagagcagtttgatttaaaagtgtcactcttcctccttttctactttcagtagatatgagatagagcataattatctgttttatcttagttttatacataatttaccatcagatagaactttatggttctagtacagatactctactacactcagcctcttatgtgccaagtttttctttaagcaatgagaaattgctcatgttcttcatcttctcaaatcatcagaggccgaagaaaaacactttggctgtgtctataacttgacacagtcaatagaatgaagaaaattagagtagttatgtgattatttcagctcttgacctgtcccctctggctgcctctgagtctgaatctcccaaagagagaaaccaatttctaagaggactggattgcagaagactcggggacaacatttgatccaagatcttaaatgttatattgataaccatgctcagcaatgagctattagattcattttgggaaatctccataatttcaatttgtaaactttgttaagacctgtctacattgttatatgtgtgtgacttgagtaatgttatcaacgtttttgtaaatatttactatgtttttctattagctaaattccaacaattttgtactttaataaaatgttctaaacattgcaaaaaaaaaaaaaaaaaaaaaaaaaaannnnnnnnnnnannnaannncgcctatagtgaatcctattaccattacctggccgcgttttacacctcttgattggaaaaccctgggttaccaacttaatgccttggaaaaatccactttg

>RACGAP1_probe1

GTACAACTCGTATTTATCTCTGATG

>RACGAP1_probe2

GAATGTTTGACTTCGTATTGACCCT

>RACGAP1_probe3

GGATGCTGAAATTTTTCCCATGGAA

>RACGAP1_probe4

ACTTCGTATTGACCCTTATCTGTAA

>RACGAP1_probe5

CAATATATCATCCTTTGGCATCCCA

>CKS2_probe1

CGCTCTCGTTTCATTTTCTGCAGCG

>CKS2_probe2

TATTCTTCTCTTTAGACGACCTCTT

>CKS2_probe3

TCTCTTTAGACGACCTCTTCCAAAA

>AURKA_probe1

CTACCTCCATTTAGGGATTTGCTTG

>AURKA_probe2

GTGTCTCAGAGCTGTTAAGGGCTTA

>AURKA_probe3

CCCTCAATCTAGAACGCTACACAAG

>AURKA_probe4

GAGGCCATGTGTCTCAGAGCTGTTA

>AURKA_probe5

TTAGGGATTTGCTTGGGATACAGAA

>AURKA_probe6

GTGCTCTACCTCCATTTAGGGATTT

>AURKA_probe7

AAATAGGAACACGTGCTCTACCTCC

>AURKA_probe8

GGGATACAGAAGAGGCCATGTGTCT

>AURKA_probe9

GAAGAGGCCATGTGTCTCAGAGCTG

>AURKA_probe10

CAGAGCTGTTAAGGGCTTATTTTTT

>AURKA_probe11

CATTGGAGTCATAGCATGTGTGTAA

>AURKA_target

ccctcaatctagaacgctacacaagaaatattttgtttttactcagcaggtgtgccttaacctccctattcagaaagctccacatcaataaacatgacactctgaagtgaaagtagccacgagaattgtgctacttatactggaacataatctggaggcaaggttcgactgcagtcgaaccttgcctccagattatgaaccagtataagtagcacaattctcgtggctactttcacttcagagtgtcatgtttattgatgtggagctttctgaatagggaggttaaggcacacctgctgagtaaaacaaatatttcttgtgtagcgttcttaggaatctggtgtctgtccggccccggtaggcctgttgggtttctagtcctccttaccatcatctccatatgagagtgtgaaaataggaacacgtgctctacctccatttagggatttgcttgggatacagaagaggccatgtgtctcagagctgttaagggcttatttttttaaaacattggagtcatagcatgtgtgtaa

>AURKA_exemplar

gaattccgggactgagctcttgaagacttgggtccttggtcgcaggtggagcgacgggtctcactccattgcccaggccagagtgcgggatatttgataagaaacttcagtgaaggccgggcgcggtgctcatgcccgtaatcccagcattttcggaggccgaggcatcatggaccgatctaaagaaaactgcatttcaggacctgttaaggctacagctccagttggaggtccaaaacgtgttctcgtgactcagcaatttccttgtcagaatccattacctgtaaatagtggccaggctcagcgggtcttgtgtccttcaaattcttcccagcgcgttcctttgcaagcacaaaagcttgtctccagtcacaagccggttcagaatcagaagcagaagcaattgcaggcaaccagtgtacctcatcctgtctccaggccactgaataacacccaaaagagcaagcagcccctgccatcgcacctgaaaataatcctgaggaggaactggcatcaaaacagaaaaatgaagaatcaaaaagaggcagtggctttggaagactttgaaattggtcgccctctgggtaaaggaaagtttggtaatgtttatttggcaagagaaaagcaaagcaagtttattctggctcttaaagtgttatttaaagctcagctggagaaagccggagtggagcatcagctcagaagagaagtagaaatacagtcccaccttcggcatcctaatattcttagactgtatggttatttccatgatgctaccagagtctacctaattctggaatatgcaccacttggaacagtttatagagaacttcagaaactttcaaagtttgatgagcagagaactgctaacttatataacagaattgcaaatgccctgtcttactgtcattcgaagagagttattcatagagacattaagccagagaacttacttcttggatcagctggagagcttaaaattgcagattttgggtggtcagtacatgctccatcttccaggaggaccactctctgtggcaccctggactacctgccccctgaaatgattgaaggtcggatgcatgatgagaaggtggatctctggagccttggagttctttgctatgaatttttagttgggaagcctccttttgaggcaaacacataccaagagacctacaaaagaatatcacgggttgaattcacattccctgactttgtaacagagggagccagggacctcatttcaagactgttgaagcataatcccagccagaggccaatgctcagagaagtacttgaacacccctggatcacagcaaattcatcaaaaccatcaaattgccaaaacaaagaatcagctagcaaacagtcttaggaatcgtgcagggggagaaatccttgagccagggctgccatataacctgacaggaacatgctactgaagtttattttaccattgactgctgccctcaatctagaacgctacacaagaaatattttgtttttactcagcaggtgtgccttaacctccctattcagaaagctccacatcaataaacatgacactctgaagtgaaagtagccacgagaattgtgctacttatactggaacataatctggaggcaaggttcgactgcagtcgaaccttgcctccagattatgaaccagtataagtagcacaattctcgtggctactttcacttcagagtgtcatgtttattgatgtggagctttctgaatagggaggttaaggcacacctgctgagtaaaacaaatatttcttgtgtagcgttcttaggaatctggtgtctgtccggccccggtaggcctgttgggtttctagtcctccttaccatcatctccatatgagagtgtgaaaataggaacacgtgctctacctccatttagggatttgcttgggatacagaagaggccatgtgtctcagagctgttaagggcttatttttttaaaacattggagtcatagcatgtgtgtaaactttaaatatgcaggccttcgtggctcgag

>FEN1_probe1

GAACTTGCTATGTAATTTGTGTCTA

>FEN1_probe2

GATGGTGATGTTCACCTGGCAATCA

>FEN1_probe3

GAGCCACCAGGAAGGCGCATCTTAG

>FEN1_probe4

TTGACCCACCTTGAGAGAGAGCCAC

>FEN1_probe5

GGACACTAAGTCCATTGTTACATGA

>FEN1_probe6

GAAATGATTTCCTGGCTGGCCAACT

>FEN1_probe7

ACACTGGTTTTCATGCGCTGTTTTT

>FEN1_probe8

ACTGATTACTGGCTGTGTCTTGGGT

>FEN1_probe9

TGGACCTAGACTGTGCTTTTCTGTC

>FEN1_probe10

TTGGGTGGGCAGAAACTCGAACTTG

>FEN1_probe11

ACCTGGCAATCAGCTGAGTTGAGAC

>FEN1_target

ggacactaagtccattgttacatgaaagtgatagatagcaacaagttttggagaagagagagggagataaaagggggagacaaaagatgtacagaaatgatttcctggctggccaactggtggccagtgggaggtgatggtggacctagactgtgcttttctgtcttgttcagccttgacccaccttgagagagagccaccaggaaggcgcatcttagcagatgggaggaactgctgagagaagatgggcagaaagctggagcccctggagttggctgtgtctgtgtttgtgactgattactggctgtgtcttgggtgggcagaaactcgaacttgctatgtaatttgtgtctagttattcagaggagtaagatggtgatgttcacctggcaatcagctgagttgagactttggaataagacactggttttcatgcgctgttttt

>FEN1_exemplar

ggcacgaggcaggccacccgccgctaagctgagaagggagagcgagcttaggaccgcctgcccggggcaaccccgaaccaagctttagccgccgaggccgcgtgtcccaaaggccagtcatccctcctctgtgttgccatgggaattcaaggcctggccaaactaattgctgatgtggcccccagtgccatccgggagaatgacatcaagagctactttggccgtaaggtggccattgatgcctctatgagcatttatcagttcctgattgctgttcgccagggtggggatgtgctgcagaatgaggagggtgagaccaccagccacctgatgggcatgttctaccgcaccattcgcatgatggagaacggcatcaagcccgtgtatgtctttgatggcaagccgccacagctcaagtcaggcgagctggccaaacgcagtgagcggcgggctgaggcagagaagcagctgcagcaggctcaggctgctggggccgagcaggaggtggaaaaattcactaagcggctggtgaaggtcactaagcagcacaatgatgagtgcaaacatctgctgagcctcatgggcatcccttatcttgatgcacccagtgaggcagaggccagctgtgctgccctggtgaaggctggcaaagtctatgctgcggctaccgaggacatggactgcctcaccttcggcagccctgtgctaatgcgacacctgactgccagtgaagccaaaaagctgccaatccaggaattccacctgagccggattctgcaggagctgggcctgaaccaggaacagtttgtggatctgtgcatcctgctaggcagtgactactgtgagagtatccggggtattgggcccaagcgggctgtggacctcatccagaagcacaagagcatcgaggagatcgtgcggcgacttgaccccaacaagtaccctgtgccagaaaattggctccacaaggaggctcaccagctcttcttggaacctgaggtgctggacccagagtctgtggagctgaagtggagcgagccaaatgaagaagagctgatcaagttcatgtgtggtgaaaagcagttctctgaggagcgaatccgcagtggggtcaagaggctgagtaagagccgccaaggcagcacccagggccgcctggatgatttcttcaaggtgaccggctcactctcttcagctaagcgcaaggagccagaacccaagggatccactaagaagaaggcaaagactggggcagcagggaagtttaaaaggggaaaataaatgtgtttccccattatacctccttcaccccagaatatttgccgtcttgtacccttaagagctacagctagagaaaccttcacggggtggagagaggattctaaggcttttctagcgtgacccttttcagtagtgctagtcccttttttacttgatcttaatggcaagaaggccacagaggtacttttccttttttagctcaggaaaatatgtcaggctcaaaccacttctcaggcagtttaatggacactaagtccattgttacatgaaagtgatagatagcaacaagttttggagaagagagagggagataaaagggggagacaaaagatgtacagaaatgatttcctggctggccaactggtggccagtgggaggtgatggtggacctagactgtgcttttctgtcttgttcagccttgacccaccttgagagagagccaccaggaaggcgcatcttagcagatgggaggaactgctgagagaagatgggcagaaagctggagcccctggagttggctgtgtctgtgtttgtgactgattactggctgtgtcttgggtgggcagaaactcgaacttgctatgtaatttgtgtctagttattcagaggagtaagatggtgatgttcacctggcaatcagctgagttgagactttggaataagacactggttttcatgcgctgtttttgttttaaagttatgaagaaaaaagtcaataaaattctaaaagtaaaaaaaaaaaaaaaaaa

>EBP_probe1

GAAGGCACTGCTGGGAGCCATTAGA

>EBP_probe2

CAGGCTCATGGGCAGGCACAAGAAG

>EBP_probe3

GTCTTAGTCGTGACCACATGGCTGT

>EBP_probe4

CACAGATACAAGAGAAGCCAGGAGG

>EBP_probe5

AAGGGGCTGTGTGAAGGCACTGCTG

>EBP_probe6

AGAAGAACTGAGGAGTGGTGGACCA

>EBP_probe7

GCCAGGAGGTCTATGATGGTGACGA

>EBP_probe8

CCCACCTGGCATATACTGGCTGGCC

>EBP_probe9

ACATGGCTGTTGTCAGGTCGTGCTG

>EBP_probe10

TCTATGGGGATGTGCTCTACTTCCT

>EBP_probe11

GCATGGAAACCATCACAGCTTGCCT

>EBP_probe12

GAGTGGTGGACCAGGCTCGAACACT

>EBP_probe13

TTGGAGGGACAAAGCTAATTGATCT

>EBP_probe14

GATGCCAAGGCCACAAAAGCCAAGA

>EBP_probe15

CCAGGCTCGAACACTGGCCGAGGAG

>EBP_probe16

TGACAGAGCACCGCGACGGATTCCA

>EBP_probe17

GGGAGCCATTAGAACACAGATACAA

>EBP_probe18

TTTGTCTTCATGAATGCCCTGTGGC

>EBP_probe19

GGAGACCAAGCCTTCTTATCTCAAC

>EBP_probe20

TGCAGTGTGTGGGTTCATTCACCTG

>EBP_probe21

CTCCGCTTCATTCTACAGCTTGTGG

>TXNIP_probe1

TGTGTCAGAGCACTGAGCTCCACCC

>TXNIP_probe2

TACAAGTTCGGCTTTGAGCTTCCTC

>TXNIP_probe3

AAAGGATGCGGACTCATCCTCAGCC

>TXNIP_probe4

ACTTTGTTCACTGTCCTGTGTCAGA

>TXNIP_probe5

GAAAGGGTTGCTGCTGTCAGCCTTG

>TXNIP_probe6

AGATAGGGATATTGGCCCCTCACTG

>TXNIP_probe7

GGCAATCTCCTGGGCCTTAAAGGAT

>TXNIP_probe8

CTTAGCCTCTGACTTCCTAATGTAG

>TXNIP_probe9

GCAAAGGGGTTTCCTCGATTTGGAG

>TXNIP_probe10

AAATGGCCTCCTGGCGTAAGCTTTT

>TXNIP_probe11

AAACCAACTCAGTTCCATCATGGTG

>TXNIP_probe12

TTCCACCGTCATTTCTAACTCTTAA

>TXNIP_probe13

GGTTTTCTCTTCATGTAAGTCCTTG

>TXNIP_probe14

CGGAGTACCTGCGCTATGAAGACAC

>TXNIP_probe15

CCCTGCATCCTCAACAACAATGTGC

>TXNIP_probe16

GTGTTCTCCTACTGCAAATATTTTC

>TXNIP_probe17

AATTGAGGCCTTTTCGATAGTTTCG

>TXNIP_probe18

GGAGGTGGTCAGCAGGCAATCTCCT

>TXNIP_probe19

CCAGCGCCCATGTTGTGATACAGGG

>TXNIP_probe20

GAAAAACTCAGGCCCATCCATTTTC

>TXNIP_probe21

TGAGGTGGTCTTTAACGACCCTGAA

>TXNIP_probe22

TGTTCTTAGCACTTTAATTCCTGTC

>TXNIP_probe23

AGCTCCACCCTTTTCTGAGAGTTAT

>TXNIP_probe24

CACTCTCAGCCATAGCACTTTGTTC

>TXNIP_probe25

GAAGCAGCTTTACCTACTTGTTTCT

>TXNIP_probe26

GAAGTTACTCGTGTCAAAGCCGTTA

>TXNIP_probe27

GGTGGATGTCAATACCCCTGATTTA

>TXNIP_probe28

CCGAGCCAGCCAACTCAAGAGACAA

>TXNIP_probe29

TGGATGCAGGGATCCCAGCAGTGCA

>TXNIP_probe30

GATCCTGGCTTGCGGAGTGGCTAAA

>TXNIP_probe31

GCTGAAACTGGTCTACTGTGTCTCT

>SYNE2_probe1

TTTCTAAGACTTTTTCACATCCAAA

>SYNE2_probe2

GTTTTACTCCAATCAGCTGGCAATT

>SYNE2_probe3

GGCACCCTTAGCTGATGGAAACAAT

>SYNE2_probe4

ATTTTGAGCTGCCGGTTATACACCA

>SYNE2_probe5

TGTTCTGTTCAGTACCTAGCTCTGC

>SYNE2_probe6

GTAAATGCCAAACTACCGACTTGAT

>SYNE2_probe7

TACGCTTAGAATCAGTTTTACTCCA

>SYNE2_probe8

GTTCAGAAACTCATAGGCACCCTTA

>SYNE2_probe9

TGAGCAGTGGTGTCCATCACATATA

>SYNE2_probe10

ATGTACAACTCAGATGTTTCTCATT

>SYNE2_probe11

GCTCTGCTCTTTTATATTGCTTTAA

>DICER1_probe1

AATTTCTTACTATACTTTTCATAAT

>DICER1_probe2

ATTTCACCTACCAAAGCTGTGCTGT

>DICER1_probe3

ACTAGCTCATTATTTCCATCTTTGG

>DICER1_probe4

AAATGATTTTTCACAACTAACTTGT

>DICER1_probe5

TTGCAGTCTGCACCTTATGGATCAC

>DICER1_probe6

TGATACATCTGTGATTTAGGTCATT

>DICER1_probe7

GGAGACGCCAATAGCAATATCTAGG

>DICER1_probe8

CTGATGCCACATAGTCTTGCATAAA

>DICER1_probe9

AGCTGTGCTGTTAATGCCGTGAAAG

>DICER1_probe10

GAAGTGCGCCAATGTTGTCTTTTCT

>DICER1_probe11

GTGAAACCTTCATGGATAGTCTTTA

>DICER1_probe12

TTTACTAAAGTCCTCCTGCCAGGTA

>DICER1_probe13

GGACATCAACCACAGACAATTTAAA

>DICER1_probe14

TGTTGCATGCATATTTCACCTACCA

>DICER1_probe15

ATAAACCTTAGACATATCACACCTA

>DICER1_probe16

TAGTCTTTAATCTCTGATCTTTTTG

>DICER1_probe17

GAGACAGCGTGATACTTACAACTCA

>DICER1_probe18

GACCATTGTATTTTCCACTAGCAGT

>DICER1_probe19

CTGCAGCAGCAGGTTACATAGCAAA

>DICER1_probe20

GCCGTGAAAGTTTAACGTTTGCGAT

>DICER1_probe21

AACTGCCGTAATTTTGATACATCTG

>DICER1_probe22

TATTTACCATCACATGCTGCAGCTG

>DICER1_probe23

AACGTTTGCGATAAACTGCCGTAAT

>DICER1_probe24

GGAAATTTGCATTGAGACCATTGTA

>DICER1_probe25

GCACCTTATGGATCACAATTACCTT

>DICER1_probe26

AGAAGCAAAACACAGCACCTTTACC

>DICER1_probe27

CCCTTAGTCTCCTCACATAAATTTC

>DICER1_probe28

TGTGTAAGGTGATGTTCCCGGTCGC

>DICER1_probe29

CTGCCAGGTAGTTCCCACTGATGGA

>AP1AR_probe1

GCCTTCCTTTACCTTGTAGTACAAG

>AP1AR_probe2

TTTTTCCTCTTGCAACAATGACGGT

>AP1AR_probe3

GTCAATTTACAAGGCCAGGGATAGA

>AP1AR_probe4

TTCCACTTCATTTTACATGCCACTA

>AP1AR_probe5

GTGCTAGACAATTACTGTTCTTTTC

>AP1AR_probe6

AATATCTATAACTGCATTTTGTGCT

>AP1AR_probe7

GATAGAAAACACTCCATAATTGCTT

>AP1AR_probe8

CATTGATTTTATTAAGCCTTCCTTT

>AP1AR_probe9

TACATGCCACTATATTGACTTTAAT

>AP1AR_probe10

TCTGGTATGAAAGGCTCCATTGATT

>AP1AR_probe11

GCTTTCCTTGATTTTGCTGAGGATT

>NUP107_probe1

GGATATCAGCGTTTCTCTGTGTGCT

>NUP107_probe2

GAAAGCTTTGTCTGCCAATGTTGTG

>NUP107_probe3

CAGAGAGTCCTCTCTAATGCTCCTA

>NUP107_probe4

GATATTGCACAGTACTGGTCAGTAT

>NUP107_probe5

GACCAGGGACTTGACCCATTAGGGT

>NUP107_probe6

AGATATGGTATCCTCTGAGCGCCAC

>NUP107_probe7

AATGCTCCTAGACCAGGGACTTGAC

>NUP107_probe8

ATCGTGACACTTTCAACATGTAGGG

>NUP107_probe9

TTGGATGCCCTAACTGCTGATGTGA

>NUP107_probe10

GTGTTTTCTGCTTCATACGATATTG

>APOC1_probe1

AAGGGTGACATCCAGGAGGGGCCTC

>APOC1_probe2

CAGGAGGGGCCTCTGAAATTTCCCA

>APOC1_probe3

GATGCGGGAGTGGTTTTCAGAGACA

>APOC1_probe4

CAGCAAGGATTCAGGAGTGCCCCTC

>APOC1_probe5

GTGAACTTTCTGCCAAGATGCGGGA

>APOC1_probe6

CAAGGCTCGGGAACTCATCAGCCGC

>APOC1_probe7

AACACACTGGAGGACAAGGCTCGGG

>APOC1_probe8

GACGTCTCCAGTGCCTTGGATAAGC

>APOC1_probe9

CCAAGCCCTCCAGCAAGGATTCAGG

>APOC1_probe10

TCATCAGCCGCATCAAACAGAGTGA

>APOC1_probe11

GTTCTGTCGATCGTCTTGGAAGGCC

>APOC1_target

ccaagccctccagcaaggattcaggagtgcccctcgggcctcgccatgaggctcttcctgtcgctcccggtcctggtggtggttctgtcgatcgtcttggaaggcccagccccagcccaggggaccccagacgtctccagtgccttggataagctgaaggagtttggaaacacactggaggacaaggctcgggaactcatcagccgcatcaaacagagtgaactttctgccaagatgcgggagtggttttcagagacatttcagaaagtgaaggagaaactcaagattgactcatgaggacctgaagggtgacatccaggaggggcctctgaaatttccca

>APOC1_exemplar

acctcccaaccaagccctccagcaaggattcaggagtgcccctcgggcctcgccatgaggctcttcctgtcgctcccggtcctggtggtggttctgtcgatcgtcttggaaggcccagccccagcccaggggaccccagacgtctccagtgccttggataagctgaaggagtttggaaacacactggaggacaaggctcgggaactcatcagccgcatcaaacagagtgaactttctgccaagatgcgggagtggttttcagagacatttcagaaagtgaaggagaaactcaagattgactcatgaggacctgaagggtgacatccaggaggggcctctgaaatttcccacaccccagcgcctgtgctgaggactcccgccatgtggccccaggtgccaccaataaaaatcctaccg

>DTX4_probe1

ATCGCCACCTGGTGCTCATGAGGTG

>DTX4_probe2

ACTCGTCTTGGTATTGCACTGTTGT

>DTX4_probe3

ATTCTCTTCCCATTTTTGTACATTT

>DTX4_probe4

TGCTCCGTGAAAGGACATCGCCACC

>DTX4_probe5

GGAGACAAACCTCGTCAGATGCTCA

>DTX4_probe6

TGAAGTCTTTGGTGTTGCTCCGTGA

>TAF1D_probe1

TGATTGTTGCCATGTGAGAGTTTTA

>TAF1D_probe2

ACTCCTAATGTTTGGTGCTATGTTT

>TAF1D_probe3

GTATGGGTCATTTCAAAGAGGGCTT

>TAF1D_probe4

TGGTGCTATGTTTTCCTGAGGAGAT

>TAF1D_probe5

AAGTTTCTCTAGTGTTTTCTGTGGA

>TAF1D_probe6

GTATTTTTGGCTCGAAGTTTCTCTA

>TAF1D_probe7

GAAGCCATAGCACTCCTAATGTTTG

>TAF1D_probe8

AAGAGGGCTTATGAGGCTGTGAAAC

>TAF1D_probe9

CCCAGAGCTCTTAACGCTGTGACCA

>TAF1D_probe10

GAGGCTGTGAAACCCAGAGCTCTTA

>TAF1D_probe11

ATTTCTCTTCTTCAGGGCAAACTTG

>TAF1D_target

gtatgggtcatttcaaagagggcttatgaggctgtgaaacccagagctcttaacgctgtgaccaaagatggaagttctctataggaagccatagcactcctaatgtttggtgctatgttttcctgaggagatataaaacgtaataatccatgattgttgccatgtgagagttttaaaggttaatcaaaatttctcttcttcagggcaaacttgaagataaatcttttgactccagctctttagaggatctaaagtgaccttgatggacagtggaagaaatcacaacatggaattcctcgaataacaatttattgactttaaataattttgtctaatgctacatatacacaattaaaaaacctttacactatttctagaaagtcagcatgtatttttggctcgaagtttctctagtgttttctgtgga

>TAF1D_exemplar

cttttcttccgcacggttggaggaggtcggctggttatcgggagttggagggctgaggtcgggagggtggtgtgtacagagctctaggactcacgcaccaggccagtcgcggattttgggccgaggcctgggttacaagcagcaagtgcgcggttggggccactgcgaggccgttttagaaaactgtttaaaacaaagagcaattgatggataaatcaggaatagattctcttgaccatgtgacatctgatgctgtggaacttgcaaatcgaagtgataactcttctgatagcagcttatttaaaactcagtgtatcccttactcacctaaaggggagaaaagaaaccccattcgaaaatttgttcgtacacctgaaagtgttcacgcaagtgattcatcaagtgactcatcttttgaaccaataccattgactataaaagctatttttgaaagattcaagaacaggaaaaagagatataaaaaaaagaaaaagaggaggtaccagccaacaggaagaccacggggaagaccagaaggaaggagaaatcctatatactcactaatagataagaagaaacaatttagaagcagaggatctggcttcccatttttagaatcagagaatgaaaaaaacgcaccttggagaaaaattttaacgtttgagcaagctgttgcaagaggattttttaactatattgaaaagctgaagtatgaacaccacctgaaagaatcattgaagcaaatgaatgttggtgaagatttagaaaatgaagattttgacagtcgtagatacaaatttttggatgatgatggatccatttctcctattgaggagtcaacagcagaggatgaggatgcaacacatcttgaagataacgaatgtgatatcaaattggcaggggatagtttcatagtaagttctgaattccctgtaagactgagtgtatacttagaagaagaggatattactgaagaagctgctttgtctaaaaagagagctacaaaagccaaaaatactggacagagaggcctgaaaatgtgacaggatcatgaatgtcaaaggcttttatcttgagaacatggtgtctggagttaaaggtattggcatactccacacatctgtaccattcttgagtgatcgcttaggaatgaatgtgatttgaactcattcatgttgagagggtgtcaaattgagaaccaggtagatccccaccacctacagtaaaaaggaccctaaagtaaattggttgaagaaattagatcccaaagattcttggtgaattttgaagtcttcatcagtatatccatattaaaacgagatgacagaagccaaagtaattatggcaagtaatggtttttatcttaactataagttatttgctcaagggtgtaatggtcattaccaaggcttttagaatgcagtttctcatttgctgtggacatgaccataaaaaaaaatttcccagtaggttttctatctgctacgttgctagcaatcagcttattgggaacagttgattaactgtaatagaaatgcaatacaaataaaatgtgaaccacatgtgatttttctttaaaatcagtgagatttgaaaattctcctagatctcttgaatcatgcaaatttgctttgcctttatattgtaacccttgtgggttgctaataaccaagcagtttgtagtagagttaactcaggctcgttctagggactcattcatgttcactcactgtacactcatctctggaaatgtaaaatttacttttatactattgttatgtagggctgacaggacaactggatcagtttcattaaaaaggtatgtatgcattagaaaagacatttgtatgggtcatttcaaagagggcttatgaggctgtgaaacccagagctcttaacgctgtgaccaaagatggaagttctctataggaagccatagcactcctaatgtttggtgctatgttttcctgaggagatataaaacgtaataatccatgattgttgccatgtgagagttttaaaggttaatcaaaatttctcttcttcagggcaaacttgaagataaatcttttgactccagctctttagaggatctaaagtgaccttgatggacagtggaagaaatcacaacatggaattcctcgaataacaatttattgactttaaataattttgtctaatgctacatatacacaattaaaaaacctttacactatttctagaaagtcagcatgtatttttggctcgaagtttctctagtgttttctgtggaaggaataaaaatttgagtttcaaaaaaaaaaaaaaaaaaaaaaaaaaaaaaaaaaaaaaaaaaa

>FMOD_probe1

GCTGGGGAGCACTTAATTCTTCCCA

>FMOD_probe2

GGAGCTCCGATGTGAGGGGCAAGGC

>FMOD_probe3

TCTGGCTGGGGTCCGTGAAGCCCAG

>FMOD_probe4

GCCAAACCAGCTCATTTCAACAAAG

>FMOD_probe5

ATGTGAACACCATCATGCCTTTATA

>FMOD_probe6

TGCCATCACATCCCTGATACTGTGT

>FMOD_probe7

TTTGGACTACGTTCTTGGCTCCAGA

>FMOD_probe8

GCAGCCAAATCTTGCCTGTGCTGGG

>FMOD_probe9

GCTTTGAAGCACCTTCCCTGAGAAG

>FMOD_probe10

TCTGCTTTCACATCTCTGAGCTATA

>FMOD_probe11

TAATGTTGCCTGGGGCTTAACCCAC

>FMOD_target

tctggctggggtccgtgaagcccaggccaggcagccaaatcttgcctgtgctgggcatacaaccctctgctttcacatctctgagctatatcctcattagtgaaggtggcttttgctttatagtttggctggggagcacttaattcttcccatttcaaaaggtaatgttgcctggggcttaacccacctgccctttgggcaaggttgggacaaagccatctgggcagtcaggggcaaggactgttggaggagagttagcccaagtataggctctgcccagatgccatcacatccctgatactgtgtatgctttgaagcaccttccctgagaagggaagaggggatctttggactacgttcttggctccagacctggaatccacaaaagccaaaccagctcatttcaacaaaggagctccgatgtgaggggcaaggctgccccctgccccagggctcttcagaaagcatctgcatgtgaacaccatcatgcctttata

>FMOD_exemplar

cggaattcaagaaacacaaaatgcagtgggcgtccctcctgctgctggcagggctcttctccctctcccaggcccagtatgaagatgaccctcattggtggttccactacctccgcagccagcagtccacctactacgatccctatgacccttacccgtatgagacctacgagccttacccctatggggtggatgaagggccagcctacacctacggctctccatcccctccagatccccgcgactgcccccaggaatgcgactgcccacccaacttcctcacggccatgtactgtgacaatcgcaacctcaagtacctgcccttcgttccctcccgcatgaagtatgtgtacttccagaacaaccagatcacctccatccaggaaggcgtctttgacaatgccacagggctgctctggattgctctccacggcaaccagatcaccagtgataaggtgggcaggaaggtcttctccaagctgaggcacctggagaggctgtacctggaccacaacaacctgacccggatgcccggtcccctgcctcgatccctgagagagctccatctcgaccacaaccagatctcacgggtccccaacaatgctctggaggggctggagaacctcacggccttgtacctccaacacgatgagatccaggaagtgggcagttccatgaggggcctccggtcactgatcttgctggacctgagttataaccaccttcggaaggtgcctgatgggctgccctcagctcttgagcagctgtacatggagcacaacaatgtctacaccgtccccgatagctacttccggggggcgcccaagctgctgtatgtgcggctgtcccacaacagtctaaccaacaatggcctggcctccaacaccttcaattccagcagcctccttgagctagacctctcctacaaccagctgcagaagatccccccagtcaacaccaacctggagaacctctacctccaaggcaataggatcaatgagttctccatcagcagcttctgcaccgtggtggacgtcgtgaacttctccaagctgcaggtcgtgcgcctggacgggaacgagatcaagcgcagcgccatgcctgccgacgcgcccctctgcctgcgccttgccagcctcatcgagatctgagcagccctggcaccgggtactgggcggagagcccccgtggcatttggcttgatggtttggtttggcttatggaagatctgggacagaccgtgtgacagaagtccacgggcaccctctgtagtcttctttcctgtaggtggggttagggggggcgatcagggacaggcagccttctgctgaggacataggcagaagctcactcttttccagggacagaagtggtggtagatggaaggatccctggatgttccaaccccataaatctcacggctcttaagttcttcccaatgatctgaggtcatggaacttcaaaagtggcatgggcaatagtatataaccatacttttctaacaatccctggctgtctgtgagcagcacttgacagctctccctctgtgctgggctggtcgtgcagttactctgggctcccatttgttgcttctcaaaatatacctcttgcccagctgcctcttctgaaatccacttcacccactccactttcctccacagatgcctcttctgtgccttaagcagagtcaggagaccccaaggcatgtgagcatctgcccagcaacctgtggagacaacccacactgtgtctgagggtgaaaggacaccaggagtcacttctatacctccctaacctcacccctggaaagccaccagattggaggtcaccagcatgatgataatattcatgacctgatgtgggaggagacagccaacctcaggcttagatcaatgtatagggctatattttggcagctgggtagctctttgaaggtggataagacttcagaagaggaaaggccagactttgcttaccatcagcatctgcaatgggccaaacacacctcaaattggctgagttgagaaagcagccccagtagttccattcttgcccagcactttctgcattccaaacagcatcctacctgggtttttatccacaaaggtagcggccacatggtttttaaagtatgagaaacacagtttgtcctctccttttatccaagcaggaagattctatatcctgatggtagagacagactccaggcagccctggacttgctagcccaaagaaggaggatgtggttaatctgtttcacctggtttgtcctaaggccatagttaaaaagtaccagctctggctggggtccgtgaagcccaggccaggcagccaaatcttgcctgtgctgggcatacaaccctctgctttcacatctctgagctatatcctcattagtgaaggtggcttttgctttatagtttggctggggagcacttaattcttcccatttcaaaaggtaatgttgcctggggcttaacccacctgccctttgggcaaggttgggacaaagccatctgggcagtcaggggcaaggactgttggaggagagttagcccaagtataggctctgcccagatgccatcacatccctgatactgtgtatgctttgaagcaccttccctgagaagggaagaggggatctttggactacgttcttggctccagacctggaatccacaaaagccaaaccagctcatttcaacaaaggagctccgatgtgaggggcaaggctgccccctgccccagggctcttcagaaagcatctgcatgtgaacaccatcatgcctttataaaggatccttattacaggaaaagcatgagtggtggctaacctgaccaataaagttattttatgattgcc

>MAPKAPK2_probe1

GCTGAAGAGGCGGAAGAAAGCTCGG

>MAPKAPK2_probe2

CTCCTGCCCACGGGAGGACAAGCAA

>MAPKAPK2_probe3

CCTGCCCACGGGAGGACAAGCAATA

>MAPKAPK2_probe4

GGACAAGCAATAACTCTCTACAGGA

>MAPKAPK2_probe5

AACTCTCTACAGGAATATATTTTTT

>MAPKAPK2_probe6

GTTGACTACGAGCAGATCAAGATAA

>MAPKAPK2_probe7

AATGCGCGTTGACTACGAGCAGATC

>MAPKAPK2_probe8

CACAATGCGCGTTGACTACGAGCAG

>MAPKAPK2_probe9

GCGCGTTGACTACGAGCAGATCAAG

>MAPKAPK2_probe10

AAGCAATAACTCTCTACAGGAATAT

>MAPKAPK2_probe11

AGACAGAACTGTCCACATCTGCCTC

>MAPKAPK2_target

cacaatgcgcgttgactacgagcagatcaagataaaaaagattgaagatgcatccaaccctctgctgctgaagaggcggaagaaagctcgggccctggaggctgcggctctggcccactgagccaccgcgccctcctgcccacgggaggacaagcaataactctctacaggaatatattttttaaacgaagagacagaactgtccacatctgcctc

>MAPKAPK2_exemplar

gatatcacagcaacattgaaatgctaaaaagtttttaaacactctcaatttctaattcaccatgtcacagactggtgaaaaaaaaaaaaaaagcggccgcttccccccggccgggcccccgccgccccgcggtccccagagcgccaggcccccggggggagggagggagggcgccgggccggtgggagccagcggcgcgcggtgggacccacggagccccgcgacccgccgagcctggagccgggccggctcggggaagccggctccagcccggagcgaacttcgcagcccgtcggggggcggcggggagggggcccggagccggaggagggggcggccgcgggcacccccgcctgtgccccggcgtccccgggcaccatgctgtccaactcccagggccagagcccgccggtgccgttccccgccccggccccgccgccgcagccccccacccctgccctgccgcaccccccggcgcagccgccgccgccgcccccgcagcagttcccgcagttccacgtcaagtccggcctgcagatcaagaagaacgccatcatcgatgactacaaggtcaccagccaggtcctggggctgggcatcaacggcaaagttttgcagatcttcaacaagaggacccaggagaaattcgccctcaaaatgcttcaggactgccccaaggcccgcagggaggtggagctgcactggcgggcctcccagtgcccgcacatcgtacggatcgtggatgtgtacgagaatctgtacgcagggaggaagtgcctgctgattgtcatggaatgtttggacggtggagaactctttagccgaatccaggatcgaggagaccaggcattcacagaaagagaagcatccgaaatcatgaagagcatcggtgaggccatccagtatctgcattcaatcaacattgcccatcgggatgtcaagcctgagaatctcttatacacctccaaaaggcccaacgccatcctgaaactcactgactttggctttgccaaggaaaccaccagccacaactctttgaccactccttgttatacaccgtactatgtggctccagaagtgctgggtccagagaagtatgacaagtcctgtgacatgtggtccctgggtgtcatcatgtacatcctgctgtgtgggtatccccccttctactccaaccacggccttgccatctctccgggcatgaagactcgcatccgaatgggccagtatgaatttcccaacccagaatggtcagaagtatcagaggaagtgaagatgctcattcggaatctgctgaaaacagagcccacccagagaatgaccatcaccgagtttatgaaccacccttggatcatgcaatcaacaaaggtccctcaaaccccactgcacaccagccgggtcctgaaggaggacaaggagcggtgggaggatgtcaaggggtgtcttcatgacaagaacagcgaccaggccacttggctgaccaggttgtgagcagaggattctgtgttcctgtccaaactcagtgctgtttcttagaatccttttattccctgggtctctaatgggaccttaaagaccatctggtatcatcttctcattttgcagaagagaaactgaggcccagaggcggagggcagtctgctcaaggtcacgcagctggtgactggttggggcagaccggacccaggtttcctgactcctggcccaagtctcttcctcctatcctgcgggatcactggggggctctcagggaacagcagcagtgccatagccaggctctctgctgcccagcgctggggtgaggctgccgttgtcagcgtggaccactaaccagcccgtcttctctctctgctcccacccctgccgcctcacctgcccttgttgtctctgtctctcactgtctcttctgctgtctctctactgtcttctggctctctctgtacccttcctggtgctgccgtgcccccaggaggagatgaccagtgccttggccacaatgcgcgttgactacgagcagatcaagataaaaaagattgaagatgcatccaaccctctgctgctgaagaggcggaagaaagctcgggccctggaggctgcggctctggcccactgagccaccgcgccctcctgcccacgggaggacaagcaataactctctacaggaatatattttttaaacgaagagacagaactgtccacatctgcctcctctcctcctcagctgcatggagcctggaactgcatcagtgactgaattc

>FOLR1_probe1

AATCTTTGAGACAAGCATATGCTAC

>FOLR1_probe2

CGGCCGTGCGTACTTAGACATGCAT

>FOLR1_probe3

CCATTCGCAGTTTCACTGTACCGGC

>FOLR1_probe4

GTGCGTACTTAGACATGCATGGCTT

>FOLR1_probe5

GGAGCGAGCGACCAAAGGAACCATA

>FOLR1_probe6

GCATATGCTACTGGCAGGATCAACC

>FOLR1_probe7

AACCATAACTGATTTAATGAGCCAT

>FOLR1_probe8

GACATGCATGGCTTAATCTTTGAGA

>FOLR1_probe9

GAGCGACCAAAGGAACCATAACTGA

>FOLR1_probe10

CAAGTAGGAGAGGAGCGAGCGACCA

>FOLR1_probe11

AATGAGCCATTCGCAGTTTCACTGT

>FOLR1_target

caagtaggagaggagcgagcgaccaaaggaaccataactgatttaatgagccattcgcagtttcactgtaccggccgtgcgtacttagacatgcatggcttaatctttgagacaagcatatgctactggcaggatcaacc

>FOLR1_exemplar

ggcccccggcgtccctcttaatcatggcctcagttccgaaaaccaacaaaatagaaccgcggtcctattccattattcctagctgcagtatcaggcggctcgggcctgctttgaacactccaatttttcaaagtaaacgcaacgggccccgcggacactcagcttacagcatcgaggggcgccagaggcaaggggcgggacgggcggtggtccctcgcgcggaccgcccgcccgctcccaagatccaactacgagctttttacctgcagcaactttactatacgctattggagctggaattaccgcggctgctggcaccagacttgccctccaatggctcctcgttaaaggatttaaagtggactcattccaattacagggcctcgaaagagtcctgtattgttattttcgtcactacctccccgggtcgggagtgggtaatttgcgcgcctgctgccttccttggatgtggtagcctccaggctccctctccggaatctgaaccctcattccccgtcacccgtggtcaccatggtcggcacggcgactaccatcgaaagttgatagggcagacgttcgaatgggtcgtcgtccgccgccacggggggcgtgcgatcggcccgaggttatctagagtcaccaaagccgccggcgcccgccccccggccggggccggagaggggctgagggttggttttgatctgataaatgcaccgatcccccccgcgaagggggtcagcgcccgtcggcatgtattagctctagaattaccacagttatccaagtaggagaggagcgagcgaccaaaggaaccataactgatttaatgagccattcgcagtttcactgtaccggccgtgcgtacttagacatgcatggcttaatctttgagacaagcatatgctactggcaggatcaaccaggacacctgcctctacgagtgctcccccaacttggggccctggatccagcaggtggatcagagctggcgcaaagagcgggtactgaacgtgcccctgtgcaaagaggactgtgagcaatggtgggaagattgtcgcacctcctacacctgcaagagcaactggcacaagggctgcaactggacttcagggtttaacaagtgcgcagtgggagctgcctgccaacctttccatttctacttccccacacccattgcccg

>KIAA1467_probe1

TCTCTAATCCCATCCTGAGGTTGCC

>KIAA1467_probe2

GGAAGCTTCATCTGACCAATGTGGG

>KIAA1467_probe3

AAATGCAAGGGTCTTACCCTCCTCT

>KIAA1467_probe4

CCACCCACCCAGGTGTCTAAGATAG

>KIAA1467_probe5

GCAAAGCCAATATGACCACTACTGA

>KIAA1467_probe6

ATCCCCTGAATGTGAATTGCTATCC

>KIAA1467_probe7

AGATAGGACATGCTCCTTTCTTTCT

>KIAA1467_probe8

TTGCTATCCTTATTGCCCTATTAAA

>KIAA1467_probe9

TGGTATGGTGAAACTAATCCCCTGA

>KIAA1467_probe10

TTGCCATCCCCCAAATGTGTGGTAT

>KIAA1467_probe11

CTTGTGAAATGTGTCCCTAAGCCTC

>SUPT4H1_probe1

TACCCTCCAATTCAGACTCAGCTGA

>SUPT4H1_probe2

CAGAACTTCAAATACTTCCTACCCT

>SUPT4H1_probe3

CCTGCCCCAAGGAATCGTGCGGGAG

>SUPT4H1_probe4

GACAGCTGGGTCTCCAAGTGGCAGC

>SUPT4H1_probe5

ATCTTCTTTGGACTACAGGTGGGGT

>SUPT4H1_probe6

TAGGATGCTGATTTTCCTACCCGTG

>SUPT4H1_probe7

GTATATGACTGCACTAGCTCTTCCT

>SUPT4H1_probe8

GAGAGCAGCACATCATTTTATCATT

>SUPT4H1_probe9

GTCGAGGAGTGGCCTACAAATCCAG

>SUPT4H1_probe10

TGCAAGGCTGCCAGCATCTTTGCTC

>SUPT4H1_probe11

ATATGCGGTGTCAGTCACTGGTCGC

>SUPT4H1_target

gtatatgactgcactagctcttcctttgatggaatcattgcgatgatgagtccagaggacagctgggtctccaagtggcagcgagtcagtaactttaagccaggtgtatatgcggtgtcagtcactggtcgcctgccccaaggaatcgtgcgggagctgaaaagtcgaggagtggcctacaaatccagagacacagctataaagacctagcaagatgcaaggctgccagcatctttgctctccacctcctgcctctgcttatttcttgttctggaactaaatgaacagaacttcaaatacttcctaccctccaattcagactcagctgactgttgagagagcagcacatcattttatcattttatcttctttggactacaggtggggtgggagggatttgggttggtggattaacagatggaattgaggagagagtaggatgctgattttcctacccgtg

>SUPT4H1_exemplar

ggcacgaggcttcctgcgggtgcacaggctgtggtcgtctatctccctgttgttcttcccatcggcgaagatggccctggagacggtgccgaaggacctgcggcatctgcgggcctgtttgctgtgttcgctggtcaagactatagaccagtttgaatatgatggttgtgacaattgtgatgcatatctacaaatgaagggtaaccgagagatggtatatgactgcactagctcttcctttgatggaatcattgcgatgatgagtccagaggacagctgggtctccaagtggcagcgagtcagtaactttaagccaggtgtatatgcggtgtcagtcactggtcgcctgccccaaggaatcgtgcgggagctgaaaagtcgaggagtggcctacaaatccagagacacagctataaagacctagcaagatgcaaggctgccagcatctttgctctccacctcctgcctctgcttatttcttgttctggaactaaatgaacagaacttcaaatacttcctaccctccaattcagactcagctgactgttgagagagcagcacatcattttatcattttatcttctttggactacaggtggggtgggagggatttgggttggtggattaacagatggaattgaggagagagtaggatgctgattttcctacccgtggcccaggtctgtgccttccccatgccaaggactctaggtcaaatgtcaataaatatgaacctcgagaaagttaaaaaaaaaaaaaaaaaa

>MELK_probe1

AAGACTGTTATGATCGCTTTGATTT

>MELK_probe2

GCCCATCTGTCATTATGTTACTGTC

>MELK_probe3

AGGGCGATGCCTGGGTTTACAAAAG

>MELK_probe4

AGCTCTTAACTATGTCTCTTTGTAA

>MELK_probe5

GATTCTTCCATCCTGCCGGATGAGT

>MELK_probe6

GAATCTAAATCAAGCCCATCTGTCA

>MELK_probe7

GAGCTATCTTAAGACCAATATCTCT

>MELK_probe8

GGAAGACATCCTATCTAGCTGCAAG

>MELK_probe9

GTGTGGGTGTGATACAGCCTACATA

>MELK_probe10

ATGTGGTGGGTATCAGGAGGCAGCG

>MELK_probe11

GGAGGCAGCGGCTTAAGGGCGATGC

>MCM2_probe1

TTGTGCTTCTCACCTTTGGGTGGGA

>MCM2_probe2

GGATGCCTGCGTGTGGTTTAGGTGT

>MCM2_probe3

TAGCAGGATGTCTGGCTGCACCTGG

>MCM2_probe4

TCTCCACTCAGTACCTTGGATCAGA

>MCM2_probe5

GAGTCATGCGGATTATCCACTCGCC

>MCM2_probe6

CTGGCATGACTGTTTGTTTCTCCAA

>MCM2_probe7

CCCCACTCTCTTATTTGTGCATTCG

>MCM2_probe8

AGCACTTGATGAACTCGGGGTACTA

>MCM2_probe9

GCCAGTGTGTCTTACTTGGTTGCTG

>MCM2_probe10

CCCTCTTGGCGTGAGTTGCGTATTC

>MCM2_probe11

TTGGTTGCTGAACATCTTGCCACCT

>LSM1_probe1

GAAGGACCGAGGTCTTTCCATTCCT

>LSM1_probe2

GAGTACTAATCTTTTGCCCAGAGGC

>LSM1_probe3

AGTGAAAGTGACATCCTGGCCACCT

>LSM1_probe4

ACAGTGGCATAGACTCCTTCACACA

>LSM1_probe5

ACAGGGACAGTCTTCATTTACTTGT

>LSM1_probe6

TCCATTCCTCGAGCAGATACTCTTG

>LSM1_probe7

GCACCAGCAACTACTTCTTTATATT

>LSM1_probe8

AAAAGGAGAGTGACACACCCCTCCA

>LSM1_probe9

CACCTCACGCATTTGATCACAGACT

>LSM1_probe10

CCTTCACACATCACTGTGGCACCAG

>NUSAP1_probe1

CCTTCACCTCAGTGGAGCTTCTGAG

>NUSAP1_probe2

GGCTTTGCTTAGTATCATGTCCATG

>NUSAP1_probe3

TGTACCTTCGTTCAAATATCCTCAT

>NUSAP1_probe4

CATCTGTCACTCACTATATTCACAA

>NUSAP1_probe5

GTTTTATACTGCTCAAGATCGTCAT

>NUSAP1_probe6

GGGATAGAAAGGCCACCTCTTCACT

>NUSAP1_probe7

AACTGCAGTCTTCTGCTAGCCAATA

>NUSAP1_probe8

ACTCATTCTAACATTGCTTACTTAA

>NUSAP1_probe9

CACCTCTTCACTCTCTATAGAATAT

>NUSAP1_probe10

GCTACATAGCCCTATCGAAATGCGA

>NUSAP1_probe11

TCCTCATGTAATTGCCATCTGTCAC

>NUSAP1_target

aactgcagtcttctgctagccaatagcatttacctgatggcagctagttatgcaagcttcaggagaatttgaacaataacaagaatagggtaagctgggatagaaaggccacctcttcactctctatagaatatagtaacctttatgaaacggggccatatagtttggttatgacatcaatattttacctaggtgaaattgtttaggcttatgtaccttcgttcaaatatcctcatgtaattgccatctgtcactcactatattcacaaaaataaaactctacaactcattctaacattgcttacttaaaagctacatagccctatcgaaatgcgaggattaatgctttaatgcttttagagacagggtctcactgtgttgcccaggctggtctcaaactccaccaaatgtacttcttattcattttatggaaaagactaggctttgcttagtatcatgtccatgtttccttcacctcagtggagcttctgagttttatactgctcaagatcgtcat

>NUSAP1_exemplar

gggatttgaaccncgctgacgaagtttggtgatccatcttccgagtatcgccgggatttcgaatcgcgatgatcatcccctctctagaggagctggactccctcaagtacagtgacctgcagaacttagccaagagtctgggtctccgggccaacctgagggcaaccaagttgttaaaagccttgaaaggctacattaaacatgaggcaagaaaaggaaatgagaatcaggatgaaagtcaaacttctgcatcctcttgtgatgagactgagatacagatcagcaaccaggaagaagctgagagacagccacttggccatgtcaccaaaacaaggagaaggtgcaagactgtccgtgtggaccctgactcacagcagaatcattcagagataaaaataagtaatcccactgaattccagaatcatgaaaagcaggaaagccaggatctcagagctactgcaaaagttccttctccaccagacgagcaccaagaagctgagaatgctgtttcctcaggtaacagagattcaaaggtaccttcagaaggaaagaaatctctctacacagatgagtcatccaaacctggaaaaaataaaagaactgcaatcactactccaaactttaagaagcttcatgaagctcattttaaggaaatggagtccattgatcaatatattgagagaaaaagaaacattttgaagaacacaattccatgaatgaactgaagcagcagcccatcaataagggaggggtcaggactccagtacctccaagaggaagactctctgtggcttctactcccatcagccaacgacgctcgcaaggccggtcttgtggccctgcaagtcagagtaccttgggtctgaaggggtcactcaagcgctctgctatctctgcagctaaaacgggtgtcaggttttcagctgctactaaagataatgagcataagcgttcactgaccaagactccagccagaaagtctgcacatgtgaccgtgtctgggggcaccccaaaaggcgaggctgtgcttgggacacacaaattaaagaccatcacggggaattctgctgctgttattaccccattcaagttgacaactgaggcaacgcagactccagtctccaataagaaaccagtgtttgatcttaaagcaagtttgtctcgtcccctcaactatgaaccacacaaaggaaagctaaaaccatgggggcaatctaaagaaaataattatctaaatcaacatgtcaacagaattaacttctacaagaaaacttacaaacaaccccatctccagacaaaggaagagcaacggaagaaacgcgagcaagaacgaaaggagaagaaagcaaaggttttgggaatgcgaaggggcctcattttggctgaagattaataattttttaatatcttgtaaatattcctgtattctcaacttttttccttttgtaaatttttttttttttgctgtcatccccactttagtcacgagatctttttctgctaactgttcatagtctgtgtagtgtccatgggttcttcatgtgctatgatctctgaaaagacgttatcaccttaaagctcaaattctttgggatggtttttacttaagtccattaacaattcaggtttctaacgagacccatcctaaaattctgtttctagatttttaatgtcaagttcccaagttccccctgctggttctaatattaacagaactgcagtcttctgctagccaatagcatttacctgatggcagctagttatgcaagcttcaggagaatttgaacaataacaagaatagggtaagctgggatagaaaggccacctcttcactctctatagaatatagtaacctttatgaaacggggccatatagtttggttatgacatcaatattttacctaggtgaaattgtttaggcttatgtaccttcgttcaaatatcctcatgtaattgccatctgtcactcactatattcacaaaaataaaactctacaactcattctaacattgcttacttaaaagctacatagccctatcgaaatgcgaggattaatgctttaatgcttttagagacagggtctcactgtgttgcccaggctggtctcaaactccaccaaatgtacttcttattcattttatggaaaagactaggctttgcttagtatcatgtccatgtttccttcacctcagtggagcttctgagttttatactgctcaagatcgtcataaataaaattttttctcattgtcaaaaaaaaaaaaaaa

>PRC1_probe1

TTGCACATGTCACTACTGGGGAGGT

>PRC1_probe2

CCTCTCAATCACTACTCTTCTTGAA

>PRC1_probe3

GTTCTCAAAAGCTTACCAGTGTGGA

>PRC1_probe4

GTGTTCAGTTCTGTTACACAGTGCA

>PRC1_probe5

GAGCTGTCTTTGTCGTGGAGATCTG

>PRC1_probe6

ACACAGTGCATTGCCCTTTGTTGGG

>PRC1_probe7

ACACATGCTTGTCGGAACGCTTTCT

>PRC1_probe8

ACTTGGTGTTAGCCACGCTGTTTAC

>PRC1_probe9

GTGTCCGAAGTTGAGATGGCCTGCC

>PRC1_probe10

GGGAGTCTGTTTGTTCCAATGGGTT

>PRC1_probe11

GGAGATCTGGAACTTTGCACATGTC

>FADD_probe1

GATGAGCAGTCACACTGTTACTCCA

>FADD_probe2

GCACTCTCTAAATCTTCCTTGTGAG

>FADD_probe3

GGATTATGGGTCCTGCAATTCTACA

>FADD_probe4

GAAAGGATGTTTTGTCCCATTTCCT

>FADD_probe5

AATTGCCAAGGCAGCGGGATCTCGT

>FADD_probe6

TCCTCTCTGAGACTGCTAAGTAGGG

>FADD_probe7

TGCTCAACCACTGTGGCGTTCTGCT

>FADD_probe8

TGATTGACACACAGCACTCTCTAAA

>FADD_probe9

CTGGACACTAGGGTCAGGCGGGGTG

>FADD_probe10

AGAGGCCCAGGAATCGGAGCGAAGC

>FADD_probe11

GGGGCAGTGATGGTTGCCAGGACGA

>RFC4_probe1

TCATGCAGCAACTCAGCTCGTCAAT

>RFC4_probe2

ATGTTCAAAATTCCGCTTCAAGCCT

>RFC4_probe3

AAAGCGCTACTCGATTAACAGGTGG

>RFC4_probe4

ACTCATCAGCCTTTGTGCAACTGTG

>RFC4_probe5

GAACATTTGCAACTCATCAGCCTTT

>RFC4_probe6

TCAACAGCAGCGATTACTAGACATT

>RFC4_probe7

ACCCCTGACCTCTAGATGTTCAAAA

>RFC4_probe8

GTGATGCAGCAGTTATCTCAGAATT

>RFC4_probe9

GATGGAGTATTTGCTGCCTGTCAGA

>RFC4_probe10

AAGCCATTACATTTCTTCAAAGCGC

>RFC4_probe11

TCAGCTCGTCAATCAACTCCATGAT

>RFC4_target

acccctgacctctagatgttcaaaattccgcttcaagcctctgtcagataaaattcaacagcagcgattactagacattgccaagaaggaaaatgtcaaaattagtgatgagggaatagcttatcttgttaaagtgtcagaaggagacttaagaaaagccattacatttcttcaaagcgctactcgattaacaggtggaaaggagatcacagagaaagtgattacagacattgctggggtaataccagctgagaaaattgatggagtatttgctgcctgtcagagtggctcttttgacaaactagaagctgtggtcaaggatttaatagatgagggtcatgcagcaactcagctcgtcaatcaactccatgatgtggttgtagaaaataacttatctgataaacagaagtctattatcacagaaaaacttgccgaagttgacaaatgcctagcagatggtgctgatgaacatttgcaactcatcagcctttgtgcaactgtgatgcagcagttatctcagaatt

>RFC4_exemplar

ctgccatttaggacaagctggatgatgatggtttgatagctccaggggttcgtgtataggagatgatgaatctgcttcatccagaatcacaatcttaaaaggcgggaactgaggcgactgtggggacatcagtgatcgtaagtctcctgggcccgttattctcagattaggtgacggagctaagacttcgagaccatctcgtcctttttgtatcgcggaaacctgaggaacgagccggcggcggtgacctgcacgagaagccaggctaactgggtgaagtaccatgcaagcatttcttaaaggtacatccatcagtactaaacccccgctgaccaaggatcgaggagtagctgccagtgcgggaagtagcggagagaacaagaaagccaaacccgttccctgggtggaaaaatatcgcccaaaatgtgtggatgaagttgctttccaggaagaagtggttgcagtgctgaaaaaatctttagaaggagcagatcttcctaatctcttgttttacggaccacctggaactggaaaaacatccactattttggcagcagctagagaactctttgggcctgaacttttccgattaagagttcttgagttaaatgcatctgatgaacgtggaatacaagtagttcgagagaaagtgaaaaattttgctcaattaactgtgtcaggaagtcgctcagatgggaagccgtgtccgccttttaagattgtgattctggatgaagcagattctatgacctcagctgctcaggcagctttaagacgtaccatggagaaggagtcgaaaaccacccgattctgtcttatctgtaactatgtcagtcgaataattgaacccctgacctctagatgttcaaaattccgcttcaagcctctgtcagataaaattcaacagcagcgattactagacattgccaagaaggaaaatgtcaaaattagtgatgagggaatagcttatcttgttaaagtgtcagaaggagacttaagaaaagccattacatttcttcaaagcgctactcgattaacaggtggaaaggagatcacagagaaagtgattacagacattgctggggtaataccagctgagaaaattgatggagtatttgctgcctgtcagagtggctcttttgacaaactagaagctgtggtcaaggatttaatagatgagggtcatgcagcaactcagctcgtcaatcaactccatgatgtggttgtagaaaataacttatctgataaacagaagtctattatcacagaaaaacttgccgaagttgacaaatgcctagcagatggtgctgatgaacatttgcaactcatcagcctttgtgcaactgtgatgcagcagttatctcagaattgttaacgtgatatatctggatggggggttttgtaaataatgaagttgtaataaaaataaaatgaccaaaagcaccg

>SCARB2_probe1

GTGACAATCATTTTGCTGACAGAAT

>SCARB2_probe2

AAGGGCATTTTCTTTGATTCTCAAA

>SCARB2_probe3

GGAGCCATCATATGTCACAGTGTTC

>SCARB2_probe4

AGAGAAACGTGTGCCCTATACTTCC

>SCARB2_probe5

GAAATCCATCTATCTACAGCCTAAG

>SCARB2_probe6

TAGCTCACTGTCACTCACTGAATAG

>SCARB2_probe7

GAGACACCACTTTTCAAAGGACTTC

>SCARB2_probe8

AGTTCTTTCCAGTGTTTTGTAGCTC

>SCARB2_probe9

GGACTTCTTGGTTTCAGCATAACCT

>SCARB2_probe10

GAGAAGCCTATACATTTAGCTGACA

>SCARB2_probe11

TGCCCTATACTTCCTGTGACAATCA

>SCARB2_target

gaaatccatctatctacagcctaagttacacataagtttcagaaagtctgattagactaaagagatatttcttctgggacagccnncttcttggtaattttgaagttctttttacaagttccttcctcagtttcagttctttccagtgttttgtagctcactgtcactcactgaatagagaaacgtgtgccctatacttcctgtgacaatcattttgctgacagaatgatggatgtttaaaatattgcacaaagtactttaaagaaaggtctgttaggaccagaagcagagacaccacttttcaaaggacttcttggtttcagcataacctaagacagggaattgggagccatcatatgtcacagtgttcagaattcaagcatatttaagggcattttctttgattctcaaagttcagcattcattttgaattgagaagcctatacatttagctgaca

>SCARB2_consensus

acctttggggttctttctaagataactccgcacagaaaatgagcatgagaaaataattcatcttccactttaaaccacaataggtgcattcattcctcgttaatctataattttgactgtagtttgtagatctgtctcaagacttttcagaaggatagtatcaaatgtgatttaaaccacagtaactattaagaatcataaaacacggccaggcccggtggcttacgcctgtaatcccagcactttgggaggctgagnnggcagatcgcagggtcaggagatngagaccatcctggccaacatggtgaaaccccgtctccnctaaaantacaaantttagctgggcgtggtggcacgcacctgtagtcccagctacttgggtggctgaggcaggagaattgcttgaacctgggagncagaggtnncagtgagnccgagatcangccactgtactccagcctggcgacagagtgagactctctcaaaaaaaanngaatcatgacacaagtatggaagtaatgtttttggtctccctgccagtgtgcaactcagtctaaatgtgtatatcattaactgttatcatttacagtatttaaaatatcctcctcacagatgattatatcctgataataggactaaaccaataagtatgcactagcattgtcaattttattagtatggataagtacatgcataantaagacatgcccaattatctagttaaatgttgataaattctaaaaatgtagatatttagcattattgaagaccaaatatttttgttagtttttttacattttggtgagttggagtatctaaaggttctatcttgattctatagaaaatttgagagtttaatgaattgcctcttcaagaggggaaatatatcacatattgttttagtgtagaaaaaggaaaaattgtcagtaaaacaatttattacctcaatacagggataagaagctatggaaatgaaatctatatttttttcttcatttgcttacattgaaatccagttttaaacaaaaaaaatccttttgaatggttcagttgttaagaaattctataagcactttgtcagctaaatgtataggcttctcaattcaaaatgaatgctgaactttgagaatcaaagaaaatgcccttaaatatgcttgaattctgaacactgtgacatatgatggctcccaattccctgtcttaggttatgctgaaaccaagaagtcctttgaaaagtggtgtctctgcttctggtcctaacagacctttctttaaagtactttgtgcaatattttaaacatccatcattctgtcagcaaaatgattgtcacaggaagtatagggcacacgtttctctattcagtgagtgacagtgagctacaaaacactggaaagaactgaaactgaggaaggaacttgtaaaaagaacttcaaaattaccaagaagnnggctgtcccagaagaaatatctctttagtctaatcagactttctgaaacttatgtgtaacttaggctgtagatagatggatttcccatattcataattttacaaataatcactcaatattagattaattaatacagtaacatatttggcaagggcgactcaggaagaaaaatcagttactcattacattctctcagaagcatcttttcgtagcatgatcattttgactnaaaaaanaaacagttaactatccacaagcatcataactagcaattaattctacatgaaatatctgtactttatcctcaccaacattatttatgcattctcttcgttcacaaaagtacaagttaaaaatccttaggagcttatcactatcaactcatgggtattgccccacccaacccctttcccatttgattcctaccccagtattttattgcagttggaagactgagttttcctggaagaattatgaactgaagatcagcataaaatgtaatatgttttagaaatcagatgcatttgaatgatatattggatgaagctaccattttaccctgggtcccgaactcttatttacttctgttttgcaagtgaaatggaggaatacaacataaaatggtatatacgcattttgagcacaaagttcggtgaatgaagcatactgaatgaagttagaccagtagcattaacaagtgatgcagagacattttacccacaatagtggtcacaaaattctggagctaccagcacccaacaacnannnnnttactatacaagggtttattaaatacttgctagacacataaaagaacaatttcagagcccacatgattttataaagcttaatggccagccatgtcagcctgctctttaacctctggccagaatgttcctatcacttgccagcgccgtgtctttttccttctttcaacaggcaacaagcctgcaaggaggtggagggtttccccacgtcatcgtccaggtcaggacagctcacacagtttcttcaccaagcaaaggcaatgtttaggttcgaatgaggggtgctctttcatccgctgttccctcatccatggatccctgtcctttgcatgcaagccaggtaaaaaccaaaccaaagaacacacccagcgccatgatgatgtagggtatgttggtgatgatcaaagtagtgttaatcatagacttcagtcgactcgccgtctctttatcaatgtgaacactctcattgaggtacatcactgggaaaaccatggttctaatgtctcccgtttcaacaaagtcatctaattttttgacataaatgttgatttggaacctcttggctgcttttaggattattccagtcaaaggattaatgtccacaaatgtctcatggtcttcctgatttgggtgcatgccttctatggcagaaacaaacctctcatctgcttggtaaaagtgtgggaaagacataatgatgggtgcaccattcttgcagatgctgacattcagaactcctgagcccaggcagtttccctcaggtatacagaagccggcattgtctgacgtattggctaatatttctgcaggaactttataccgaaaggcaggcagtccctgtacactctcatagtcactgaaagtaatatacactgacctgcaaaagtcagatgggaagacataaaggacctcatctttggttattagtgggtgaaaagaatctccatctgttccattaatcatattgcacttgtctgttatccaccagtcaagtgacgttttcccattccattccacaatttttgtaaagttaaggtaactgtcttctccagttagaaaaacatagtctccatcattagtcccatttttctcatagaataggccaaaatagggagagatatcgggcctgaaaacatggataagggacaagatttcatctttgtagccccagagcaattcgtcaactgtgtgagtcacaaagagcttctgctgataggctttcaacatggcctcgatgatctccctgaggaagtgcacctgggaccactctatgacagtcaatacaggaatatttaatgttctaattaagtcaattttagggtctccaacagattggtctcgttcaaaaacataggccttgttgctaacagcagatattgttgttccattatctccaaattgaatatttgctttgtttctgagttccctgtaggtgtatggccccacttcttccacccgaggggtctcccctctgaggatctcctctggattggtgacattgaagaaatagaactgagtatacacaggcagagggggcttctcccaggagtcaaatgcctcagtaccattccttaacacaattttcttctcgatactctggtctacagccttctggaagacccgggccaccagcagcgtgacgctggtcaccagcaggagcagggacaacgtccccgccgtgtagaagcagcatcggcccattctgtgcgccgctcacgggccgggccgggccgcacccgccagggatccaactgcaaggagggaggagccgccgcagaggcgtcgaagacncgggacccttcggcgccacgcccacgccctcccgncgcacggttcgtgcgcgcagctctggnctccgcggcctggcgagcgcgnccccgggtgcaccgggcggatggggccgcggagggacgggcccggactcggtttcggtttccttcgccgggcagccgtggcgcccgcctagcgcagctcgggagagtgcngggngcgcgcagggacntgacgttcgcgggc

>CALD1_probe1

CTTCCCCCACTAAGGTTTGAGACAG

>CALD1_probe2

GACGCAGGACGAGCTCAGTTGTAGA

>CALD1_probe3

GACGTATCCAGCAAGCGGAACCTCT

>CALD1_probe4

TTCAATATCCCAGTAAACCCATGTA

>CALD1_probe5

AGCAGTGATACCAACCACATCTGAA

>CALD1_probe6

CTTGAGACCAGGAGACGTATCCAGC

>CALD1_probe7

ACTGATCATCATAACTCTGTATCTG

>CALD1_probe8

GAACCCAAGCTCAAGACGCAGGACG

>CALD1_probe9

GCAAGCGGAACCTCTGGGAAAAGCA

>CALD1_probe10

GCGGAATGTGTGCAGTATCTAGAAA

>CALD1_probe11

TCTGTGGATAAGGTCACTTCCCCCA

>CALD1_target

cttgagaccaggagacgtatccagcaagcggaacctctgggaaaagcaatctgtggataaggtcacttcccccactaaggtttgagacagttccagaaagaacccaagctcaagacgcaggacgagctcagttgtagagggctaattcgctctgttttgtatttatgttgatttactaaattgggttcattatcttttatttttcaatatcccagtaaacccatgtatattatcactatatttaataatcacagtctagagatgttcatggtaaaagtactgcctttgcacaggagcctgtttctaaagaaacccatgctgtgaaatagagacttttctactgatcatcataactctgtatctgagcagtgataccaaccacatctgaagtcaacagaagatccaagtttaaaattgcctgcggaatgtgtgcagtatctagaaa

>CALD1_consensus

gaatagagcagtgtgtattcggctgcctgcctgcccgcctgcttgctctctggctgtgctcctgcttaaagaaatcagtccttcctttccgacttagtcctcgggaagaagtttcagactacaaggtatcattggaacatttcaagatcatcaaatcaaattccacagggattggtgaccaaccagaaggctcagacatctgattgctgacctgtccagacatcatctggtctccctgaacctgaaatcacaccatggatgattttgagcgtcgcagagaacttagaaggcaaaagagggaggagatgcgactcgaagcagaaagaatcgcctaccagaggaatgacgatgatgaagaggaggcagcccgggaacggcgccgccgagcccgacaggaacggctgcggcagaagcaggaggaagaatccttgggacaggtgaccgaccaggtggaggtgaatgcccagaacagtgtgcctgacgaggaggccaagacaaccaccacaaacactcaagtggaaggggatgatgaggccgcattcctggagcgcctggctcggcgtgaggaaagacgccaaaaacgccttcaggaggctctggagcggcagaaggagttcgacccaacaataacagatgcaagtctgtcgctcccaagcagaagaatgcaaaatgacacagcagaaaatgaaactaccgagaaggaagaaaaaagtgaaagtcgccaagaaagatacgagatagaggaaacagaaacagtcaccaagtcctaccagaagaatgattggagggatgctgaagaaaacaagaaagaagacaaggaaaaggaggaggaggaagaggagaagccaaagcgagggagcattggagaaaatcagatcaaagatgaaaagattaaaaaggacaaagaacccaaagaagaagttaagagcttcatggatcgaaagaagggatttacagaagttaagtcgcagaatggagaattcatgacccacaaacttaaacatactgagaatactttcagccgccctggagggagggccagcgtggacaccaaggaggctgagggcgccccccaggtggaagccggcaaaaggctggaggagcttcgtcgtcgtcgcggggagaccgagagcgaagagttcgagaagctcaaacagaagcagcaggaggcggctttggagctggaggaactcaagaaaaagagggaggagagaaggaaggtcctggaggaggaagagcagaggaggaagcaggaggaagccgatcgaaaactcagagaggaggaagagaagaggaggctaaaggaagagattgaaaggcgaagagcagaagctgctgagaaacgccagaagatgccagaagatggcttgtcagatgacaagaaaccattcaagtgtttcactcctaaaggttcatctctcaagatagaagagcgagcagaatttttgaataagtctgtgcagaaaagcagtggtgtcaaatcgacccatcaagcagcaatagtctccaagattgacagcagactggagcagtataccagtgcaattgagggaacaaaaagcgcaaaacctacaaagccggcagcctcggatcttcctgttcctgctgaaggtgtacgcaacatcaagagtatgtgggagaaagggaatgtgttttcatcccccactgcagcaggcacaccaaataaggaaactgctggcttgaaggtaggggtttctagccgcatcaatgaatggctaactaaaaccccagatggaaacaagtcacctgctcccaaaccttctgacttgagaccaggagacgtatccagcaagcggaacctctgggaaaagcaatctgtggataaggtcacttcccccactaaggtttgagacagttccagaaagaacccaagctcaagacgcaggacgagctcagttgtagagggctaattcgctctgttttgtatttatgttgatttactaaattgggttcattatcttttatttttcaatatcccagtaaacccatgtatattatcactatatttaataatcacagtctagagatgttcatggtaaaagtactgcctttgcacaggagcctgtttctaaagaaacccatgctgtgaaatagagacttttctactgatcatcataactctgtatctgagcagtgataccaaccacatctgaagtcaacagaagatccaagtttaaaattgcctgcggaatgtgtgcagtatctagaaaaatgaaccgtagtttttgtttttttaaatacagaagtcatgttgtttctgcactttataataaagcatggaagaaattatcttagtaggcaattgtaacactttttgaaagtaacccatttcagatttgaaatactgcaataatggttgtctttaaaaaaaaaaaagagaaatgtactgttaaggtattactttttttcatgctgatgattcatatctaaattacattattatgttagctgacagtggntactgattttttaggttggttgttttgtggatttctttagtagtgatagtagcctgaaccacattttagataactcaattatgtatgtatgtgcatacacatatacaaacacactaatggtagaatgcttttttatgtgctagactattatatttagtagtatgtcattgtaactagccaatatcacagcttttgaaaaattaaaaaatcatcactantattaatatttcatatttgccaacagaaacatggcagataggtatcaatatgttttcaatgcctgatgacctataagaagaaagtattgaaaagaagagagattagaactgttagaaggagttgaaattttctaaaagacatagtatttagtttataattaaatgcattcttgaagtccagtgtgaattttattaatgctatcatctcgaccaagctcaaagcctacttattagaaacaatgaagttcacaataggtcataaggtctcttccttttctaaaattgaaagacaagaaatttagtgccaatattgtacagacagaaattccatgtatgagtctcaacaaagactacctttggctaaatgtctagaagcagagaagtaaagtgagcaaaatccagtgttgaggagtcatgacagtactttgatctttatatactctgaagcatttcttcaaacttttctacttttnnnngtcattgatacctgtagtaagttgacaatgtggtgaaatttcaaaattatatgtaacttctactagttttactttctcccccaagtctntnnnaactcatgatttttacacacacaatccagaacttattatatagcctctaagtcttnattcttcnannagataantganagagtcntncagngnntggcaaaatgnnctagtatagctggatacataccgtggagtctataaactcatacatcagtggacttaaccnaaantggggtagctcaattcctccacactgaggg

>PDCD6_probe1

GGTTGGTGCAGCAGTCATTAAAAGT

>PDCD6_probe2

GAGTCAAGGCCAGACTAGATCAGCC

>PDCD6_probe3

TTCTCATGGAGCTTCCTTTCTAGAG

>PDCD6_probe4

CAAAGGGGCGTGTCATGTGCCTCAT

>PDCD6_probe5

CAAGGCCAGACTAGATCAGCCTAAG

>PDCD6_probe6

CATGGAGCTTCCTTTCTAGAGGGGA

>PDCD6_probe7

CTCTATTCTCATGGAGCTTCCTTTC

>PDCD6_probe8

ATTTGAGTAGATTTGGCCTCTATTC

>PDCD6_probe9

GACTTTCAAAGGGGCGTGTCATGTG

>PDCD6_probe10

TTGGCCTCTATTCTCATGGAGCTTC

>PDCD6_probe11

GATTCTAATAGGTTGGTGCAGCAGT

>PDCD6_target

gagtcaaggccagactagatcagcctaagcccagccacgccangggtgcaggagtgaagagcaaatgctaactgtccatggaattgactttcaaaggggcgtgtcatgtgcctcatcccagcaacagggaaggcanttctctatcagtcagttggtaaatgattattgacaaacaatgtgctgggaaggtggagtgatttgagtagatttggcctctattctcatggagcttcctttctagaggggaaggcagatgatggatggataaatataaatgattctaataggttggtgcagcagtcattaaaagt

>PDCD6_consensus

tccctgcgcacccccctcacttggacttaaccttggttaggttgccaacccccgtctcctgactccgggaagctagatgctctcctagcactcggaacttgcccatcgccacatttgcacacccgtggttactgggtcaggttggcgcacaagtcgtcgcgggttttgccattactatcaatgaacggcagcaacggntnntnnnnntttnttttnntttttttttgccatnacttttaatgactgctgcaccaacctattagaatcatttatatttatccatccatcatctgccttcccctctagaaaggaagctccatgagaatagaggccaaatctactcaaatcactccaccttcccagcacattgtttgtcaataatcatttaccaactgactgatagagaantgccttccctgttgctgggatgaggcacatgacacgcccctttgaaagtcaattccatggacagttagcatttgctcttcactcctgcacccntggcgtggctgggcttaggctgatctagtctggccttgactccaggctaagnatgggaaccatgcctgctccacangcctctcatcccacagccagagcngccgttccctggggcacgtgcatctcntggggaaaatcaagagccttagacggcaggcctggcagtgcccacacattccagg

>FAM38A_probe1

GCTACGGCATCATGGGGCTGTACGT

>FAM38A_probe2

ATCATGGGGCTGTACGTGTCCATCG

>FAM38A_probe3

CATTATGTTCGAGGAGCTGCCGTGC

>FAM38A_probe4

GCTGGCGCCCGAGAGGGAAGGAGCC

>FAM38A_probe5

GCTGGTCATCGGCAAGTTCGTGCGC

>FAM38A_probe6

GAGGAGTTGTACGCCAAGCTCATCT

>FAM38A_probe7

CGCTCACCGGAGACCATGATCAAGT

>FAM38A_probe8

GCGGATTCTTCAGCGAGATCTCGCA

>FAM38A_probe9

TTCGTGCGCGGATTCTTCAGCGAGA

>FAM38A_probe10

TCCCCCACGTGTACTGTAGAGTTTT

>FAM38A_probe11

AGATCTCGCACTCCATTATGTTCGA

>FAM38A_target

gctacggcatcatggggctgtacgtgtccatcgtgctggtcatcggcaagttcgtgcgcggattcttcagcgagatctcgcactccattatgttcgaggagctgccgtgcgtggaccgcatcctcaagctctgccaggacatcttcctggtgcgggagactcgggagctggagctggaggaggagttgtacgccaagctcatcttcctctaccgctcaccggagaccatgatcaagtggactcgtgagaaggagtaggagctgctgctggcgcccgagagggaaggagccggcctgctgggcagcgtggccacaaggggcggcactcctcaggccgggggagccactgccccgtccaaggccgccagctgtgatgcatcctcccggcctgcctgagccctgatgctgctgtcagagaaggacactgcgtccccacggcctgcgtggcgctgccgtcccccacgtgtactgtagagtttt

>FAM38A_exemplar

ccatggacctgcgccctgagctgcccaccaccctgggccccgtcagcctgcgccagctggggctggagcacacccgctacccctgtctggaccttggtgccatgttgctctacaccctgaccttctggctcctgctgcgccagtttgtgaaagagaagctgctgaagtgggcagagtctccagctgcgctgacggaggtcaccgtggcagacacagagcctacgcggacgcagacgctgttgcagagcctgggggagctggtgaagggcgtgtacgccaagtactggatctatgtgtgtgctggcatgttcatcgtggtcagcttcgccggccgcctcgtggtctacaagattgtctacatgttcctcttcctgctctgcctcaccctcttccaggtctactacagcctgtggcggaagctgctcaaggccttctggtggctcgtggtggcctacaccatgctggtcctcatcgccgtctacaccttccagttccaggacttccctgcctactggcgcaacctcactggcttcaccgacgagcagctgggggacctgggcctggagcagttcagcgtatccgagctcttctccagcatcctggtgcccggcttcttcctcctggcctgcatcctgcagctgcactacttccacaggcccttcatgcagctcaccgacatggagcacgtgtccctgcctggcacgcgcctcccgcgctgggctcacaggcaggatgcagtgagtgggaccccactgctgcgggaggagcagcaggagcatcagcagcagcagcaggaggaggaggaggaggaggactccagggacgaggggctgggcgtggccactccccaccaggccacgcaggtgcctgaaggggcagccaagtggggcctggtggctgagcgcctgctggagctggcagccggcttctcggacgtcctctcacgcgtgcaggtgttcctgcggcggctgctggagcttcacgttttcaagctggtggccctgtacaccgtctgggtggccctgaaggaggtgtcggtgatgaacctgctgctggtggtgctgtgggccttcgccctgccctacccacgcttccggcccatggcctcctgcctgtccaccgtgtggacctgcgtcatcatcgtgtgtaagatgctgtaccagctcaaggttgtcaacccccaggagtattccagcaactgcaccgagcccttccccaacagcaccaacttgctgcccacggagatcagccagtccctgctgtaccgggggcccgtggaccctgccaactggtttggggtgcggaaagggttccccaacctgggctacatccagaaccacctgcaagtgctgctgctgctggtattcgaggccatcgtgtaccggcgccaggagcactaccgccggcagcaccagctggccccgctgcctgcccaggccgtgtttgccagcggcacccgccagcagctggaccaggatctgctcggctgcctcaagtacttcatcaacttcttcttctacaaattcgggctggagatctgcttcctgatggccgtgaacgtgatcgggcagcgcatgaactttctggtgaccctgcacggttgctggctggtggccatcctcacccgcaggcaccgccaggccattgcccgcctctggcccaactactgcctcttcctggcgctgttcctgctgtaccagtacctgctgtgcctggggatgcccccggccctgtgcattgattatccctggcgctggagccgggccgtccccatgaactccgcactcatcaagtggctgtacctgcctgatttcttccgggcccccaactccaccaacctcatcagcgactttctcctgctgctgtgcgcctcccagcagtggcaggtgttctcagctgagcgcacagaggagtggcagcgcatggctggcgtcaacaccgaccgcctggagccgctgcggggggagcccaaccccgtgcccaactttatccactgcaggtcctaccttgacatgctgaaggtggccgtcttccgatacctgttctggctggtgctggtggtggtgtttgtcacgggggccacccgcatcagcatcttcgggctgggctacctgctggcctgcttctacctgctgctcttcggcacggccctgctgcagagggacacacgggcccgcctcgtgctgtgggactgcctcattctgtacaacgtcaccgtcatcatctccaagaacatgctgtcgctcctggcctgcgtcttcgtggagcagatgcagaccggcttctgctgggtcatccagctcttcagccttgtatgcaccgtcaagggctactatgaccccaaggagatgatggacagagaccaggactgcctgctgcctgtggaggaggctggcatcatctgggacagcgtctgcttcttcttcctgctgctgcagcgccgcgtcttccttagccattactacctgcacgtcagggccgacctccaggccaccgccctgctagcctccaggggcttcgccctctacaacgctgccaacctcaagagcattgacttccaccgcaggatagaggagaagtccctggcccagctgaaaagacagatggagcgtatccgtgccaagcaggagaagcacaggcagggccgggtggaccgcagtcgcccccaggacaccctgggccccaaggaccccggcctggagccagggcccgacagtccagggggctcctccccgccacggaggcagtggtggcggccctggctggaccacgccacagtcatccactccggggactacttcctgtttgagtccgacagtgaggaagaggaggaggctgttcctgaagacccgaggccgtcggcacagagtgccttccagctggcgtaccaggcatgggtgaccaacgcccaggcggtgctgaggcggcggcagcaggagcaggagcaggcaaggcaggaacaggcaggacagctacccacaggaggtggtcccagccaggaggtggagccagcagagggccccgaggaggcagcggcaggccggagccatgtggtgcagagggtgctgagcacggcgcagttcctgtggatgctggggcaggcgctagtggatgagctgacacgctggctgcaggagttcacccggcaccacggcaccatgagcgacgtgctgcgggcagagcgctacctcctcacacaggagctcctgcagggcggcgaagtgcacaggggcgtgctggatcagctgtacacaagccaggccgaggccacgctgccaggccccaccgaggcccccaatgccccaagcaccgtgtccagtgggctgggcgcggaggagccactcagcagcatgacagacgacatgggcagccccctgagcaccggctaccacacgcgcagtggcagtgaggaggcagtcaccgaccccggggagcgtgaggctggtgcctctctgtaccagggactgatgcggacggccagcgagctgctcctggacaggcgcctgcgcatcccagagctggaggaggcagagctgtttgcggaggggcagggccgggcgctgcggctgctgcgggccgtgtaccagtgtgtggccgcccactcggagctgctctgctacttcatcatcatcctcaaccacatggtcacggcctccgccggctccctggtgctgcccgtgctcgtcttcctgtgggccatgctgtcgatcccgaggcccagcaagcgcttctggatgacggccatcgtcttcaccgagatcgcggtggtcgtcaagtacctgttccagtttgggttcttcccctggaacagccacgtggtgctgcggcgctacgagaacaagccctacttcccgccccgcatcctgggcctggagaagactgacggctacatcaagtacgacctggtgcagctcatggcccttttcttccaccgctcccagctgctgtgctatggcctctgggaccatgaggaggactcaccatccaaggagcatgacaagagcggcgaggaggagcagggagccgaggaggggccaggggtgcctgcggccaccaccgaagaccacattcaggtggaagcgagggtcggacccacggacgggaccccagaaccccaagtggagctcaggccccgtgatacgaggcgcatcagtctacgttttagaagaaggaagaaggagggcccagcacggaaaggagcggcagccatcgaagctgaggacagggaggaagaagagggggaggaagagaaagaggcccccacggggagagagaagaggccaagccgctctggaggaagagtaagggcggccgggcggcggctgcagggcttctgcctgtccctggcccagggcacatatcggccgctacggcgcttcttccacgacatcctgcacaccaagtaccgcgcagccaccgacgtctatgccctcatgttcctggctgatgttgtcgacttcatcatcatcatttttggcttctgggcctttgggaagcactcggcggccacagacatcacgtcctccctatcagacgaccaggtacccgaggctttcctggtcatgctgctgatccagttcagtaccatggtggttgaccgcgccctctacctgcgcaagaccgtgctgggcaagctggccttccaggtggcgctggtgctggccatccacctatggatgttcttcatcctgcccgccgtcactgagaggatgttcaaccagaatgtggtggcccagctctggtacttcgtgaagtgcatctacttcgccctgtccgcctaccagatccgctgcggctaccccacccgcatcctcggcaacttcctcaccaagaagtacaatcatctcaacctcttcctcttccaggggttccggctggtgccgttcctggtggagctgcgggcagtgatggactgggtgtggacggacaccacgctgtccctgtccagctggatgtgtgtggaggacatctatgccaacatcttcatcatcaaatgcagccgagagacagagaagaaatacccgcagcccaaagggcagaagaagaagaagatcgtcaagtacggcatgggtggcctcatcatcctcttcctcatcgccatcatctggttcccgctgctcttcatgtcgctggtgcgctccgtggttggggttgtcaaccagcccatcgatgtcaccgtcaccctcaagctgggcggctatgagccgctgttcaccatgagcgcccagcagccgtccatcatccccttcacggcccaggcctatgaggagctgtcccggcagtttgacccccagccgctggccatgcagttcatcagccagtacagccctgaggacatcgtcacggcgcagattgagggcagctccggggcgctgtggcgcatcagtccccccagccgtgcccagatgaagcgggagctctacaacggcacggccgacatcaccctgcgcttcacctggaacttccagagggacctggcgaagggaggcactgtggagtatgccaacgagaagcacatgctggccctggcccccaacagcactgcacggcggcagctggccagcctgctcgagggcacctcggaccagtctgtggtcatccccaatctcttccccaagtacatccgtgcccccaacgggcccgaagccaaccctgtgaagcagctgcagcccaatgaggaggccgactacctcggcgtgcgtatccagctgcggagggagcagggtgcgggggccaccggcttcctcgaatggtgggtcatcgagctgcaggagtgccggaccgactgcaacctgctgcccatggtcattttcagtgacaaggtcagcccaccgagcctcggcttcctggctggctacggcatcatggggctgtacgtgtccatcgtgctggtcatcggcaagttcgtgcgcggattcttcagcgagatctcgcactccattatgttcgaggagctgccgtgcgtggaccgcatcctcaagctctgccaggacatcttcctggtgcgggagactcgggagctggagctggaggaggagttgtacgccaagctcatcttcctctaccgctcaccggagaccatgatcaagtggactcgtgagaaggagtaggagctgctgctggcgcccgagagggaaggagccggcctgctgggcagcgtggccacaaggggcggcactcctcaggccgggggagccactgccccgtccaaggccgccagctgtgatgcatcctcccggcctgcctgagccctgatgctgctgtcagagaaggacactgcgtccccacggcctgcgtggcgctgccgtcccccacgtgtactgtagagttttttttttaattaaaaaatgttttatttatacaaatgg

>APOE_probe1

GGCCCCTGGTGGAACAGGGCCGCGT

>APOE_probe2

TGGTGGAAGACATGCAGCGCCAGTG

>APOE_probe3

GAAGCGCCTGGCAGTGTACCAGGCC

>APOE_probe4

AGCAGGCCCAGCAGATACGCCTGCA

>APOE_probe5

GTGCCCAGCGACAATCACTGAACGC

>APOE_probe6

TGGGGCCCCTGGTGGAACAGGGCCG

>APOE_probe7

AAGCGCCTGGCAGTGTACCAGGCCG

>APOE_probe8

GCCCAGCGACAATCACTGAACGCCG

>APOE_probe9

GCGCGCGCGGATGGAGGAGATGGGC

>APOE_probe10

GCGACAATCACTGAACGCCGAAGCC

>APOE_probe11

CCCTGGTGGAACAGGGCCGCGTGCG

>APOE_target

gaagcgcctggcagtgtaccaggccggggcccgcgagggcgccgagcgcggcctcagcgccatccgcgagcgcctggggcccctggtggaacagggccgcgtgcgggccgccactgtgggctccctggccggccagccgctacaggagcgggcccaggcctggggcgagcggctgcgcgcgcggatggaggagatgggcagccggacccgcgaccgcctggacgaggtgaaggagcaggtggcggaggtgcgcgccaagctggaggagcaggcccagcagatacgcctgcaggccgaggccttccaggcccgcctcaagagctggttcgagcccctggtggaagacatgcagcgccagtgggccgggctggtggagaaggtgcaggctgccgtgggcaccagcgccgcccctgtgcccagcgacaatcactgaacgccgaagcc

>APOE_exemplar

cgcagcggaggtgaaggacgtccttccccaggagccgactggccaatcacaggcaggaagatgaaggttctgtgggctgcgttgctggtcacattcctggcaggatgccaggccaaggtggagcaagcggtggagacagagccggagcccgagctgcgccagcagaccgagtggcagagcggccagcgctgggaactggcactgggtcgcttttgggattacctgcgctgggtgcagacactgtctgagcaggtgcaggaggagctgctcagctcccaggtcacccaggaactgagggcgctgatggacgagaccatgaaggagttgaaggcctacaaatcggaactggaggaacaactgaccccggtggcggaggagacgcgggcacggctgtccaaggagctgcaggcggcgcaggcccggctgggcgcggacatggaggacgtgtgcggccgcctggtgcagtaccgcggcgaggtgcaggccatgctcggccagagcaccgaggagctgcgggtgcgcctcgcctcccacctgcgcaagctgcgtaagcggctcctccgcgatgccgatgacctgcagaagcgcctggcagtgtaccaggccggggcccgcgagggcgccgagcgcggcctcagcgccatccgcgagcgcctggggcccctggtggaacagggccgcgtgcgggccgccactgtgggctccctggccggccagccgctacaggagcgggcccaggcctggggcgagcggctgcgcgcgcggatggaggagatgggcagccggacccgcgaccgcctggacgaggtgaaggagcaggtggcggaggtgcgcgccaagctggaggagcaggcccagcagatacgcctgcaggccgaggccttccaggcccgcctcaagagctggttcgagcccctggtggaagacatgcagcgccagtgggccgggctggtggagaaggtgcaggctgccgtgggcaccagcgccgcccctgtgcccagcgacaatcactgaacgccgaagcctgcagccatgcgaccccacgccaccccgtgcctcctgcctccgcgcagcctgcagcgggagaccctgtccccgccccagccgtcctcctggggtggaccctagtttaataaagattcaccaagtttcacgc

>AQP1_probe1

CATAAGTCCTTTCAATTCCACCAGG

>AQP1_probe2

GCTAGACAATGATTTGGCCAGGCCT

>AQP1_probe3

CAGTGCATCACATCTGCACACTCTC

>AQP1_probe4

CTGACCTTGGAATCGTCCCTATATC

>AQP1_probe5

TGGAATCGTCCCTATATCAGGGCCT

>AQP1_probe6

GCAGCCCCTAAGTGCAAACACAGCA

>AQP1_probe7

TCTGCATATATGTCTCTTTGGAGTT

>AQP1_probe8

GAAGGCTGGATTCTATCTACATAAG

>AQP1_probe9

GCCCTTAACTATCACCAGTGCATCA

>AQP1_probe10

CACCACTGTGCACTTAGCCATGATG

>AQP1_probe11

ACCACGAGGCTGATTCCTCTCATTT

>AQP1_probe12

TGCAAAGTGGCAGGGACCGGCAGAG

>AQP1_probe13

GCAAACACAGCATGGGTCCAGAAGA

>AQP1_probe14

GCATATATGTCTCTTTGGAGTTGGA

>AQP1_probe15

AGACGTGGTCTAGACCAGGGCTGCT

>AQP1_probe16

ACTTACTGCCTGACCTTGGAATCGT

>AQP1_probe17

GGCCTAGTAACCAAGGCCCTGTCTC

>AQP1_probe18

GCATGGGTCCAGAAGACGTGGTCTA

>AQP1_probe19

GCATCTGTCTGCTCTGCATATATGT

>AQP1_probe20

TCTCAGTTTCTGCCTGGGCAATGGC

>AQP1_probe21

TTACTGCCTGACCTTGGAATCGTCC

>AQP1_probe22

GCAGGAACTTCTAGCTCATTTAACA

>SNORA25_probe1

ACTCCTAATGTTTGGTGCTATGTTT

>SNORA25_probe2

TGGTGCTATGTTTTCCTGAGGAGAT

>SNORA25_probe3

GAAGCCATAGCACTCCTAATGTTTG

>SNORA25_probe4

AAGAGGGCTTATGAGGCTGTGAAAC

>SNORA25_probe5

CCCAGAGCTCTTAACGCTGTGACCA

>SNORA25_probe6

GAGGCTGTGAAACCCAGAGCTCTTA

>RGS5_probe1

TGCTCCATTGGAGTAGTCTCCCACC

>RGS5_probe2

GGTAGAGGCCTTCTAGGTGAGACAC

>RGS5_probe3

TACTTATCTACTGTCCGAAGGCCTT

>RGS5_probe4

CCTGCATTTCCCATTAATCTACATA

>RGS5_probe5

AATGCTGAGAAATTTGCCACTGGAG

>RGS5_probe6

TATACAGTTTAATAAGCCTCTTGCA

>RGS5_probe7

ATTTAAAATATTGATCCTTCCCTTG

>RGS5_probe8

ATCTCACTTGTTTTAGTTCTGATCC

>RGS5_probe9

ATTTGGGTCCAACTTCAATAATGTA

>RGS5_probe10

GACTGTGGGTCAAATGTTTCCATTT

>RGS5_probe11

AAATGAAACTGTTGCTCCATTGGAG

>RGS5_probe12

GTATCTGTAACCACAATCACACATA

>RGS5_probe13

GGACCACCTTCATGTTAGTTGGGTA

>RGS5_probe14

TTGCAAGTTACTTGTTCTCTCACCT

>RGS5_probe15

CTTTTTGCCCACACTGCTTTGGATA

>RGS5_probe16

AGATCACCCCTCTAATTATTTCTGA

>RGS5_probe17

TATTTCCTCCATAATAACCCTGCAT

>RGS5_probe18

GGGATGTTGCTTACTCTTTTTGCCC

>RGS5_probe19

GTACTATGTGACTCATGCTTCTGGA

>RGS5_probe20

GTTCTCTCACCTGAGGTATTTTTTT

>RGS5_probe21

GCCACTGGAGACAAGCAATCTGAAT

>RGS5_probe22

TCATCCTGTGAGTTATTTCCTCCAT

>RGS5_probe23

TGCAACTAGCAACTCATCTTCGGAA

>RGS5_probe24

CTGCCCATAGTCACCAAATTCTGTT

>RGS5_probe25

TGGAAAAGGATTCTCTGCCTCGCTT

>RGS5_probe26

GCTAATTGTCCTATGATGCTATTAT

>RGS5_probe27

TTCCTCTTCTCCCTTTGCAAGAGGA

>RGS5_probe28

ATGACATTTATCTTCAAAACACCAA

>RGS5_probe29

GAGTAGTCTCCCACCTAAATATCAA

>RGS5_probe30

TTCCCACAGCAGCTTTGCTCAGTGA

>RGS5_probe31

CTCGCTTTGTGCGCTCTGAGTTTTA

>RGS5_probe32

ATCCATTTGTAAGCATTTATCCCAT

>RGS5_probe33

ATGTATTTATGCTGCTAGACTGTGG

>MTUS1_probe1

TCTTCACCACAGACACCTTCTTGTG

>MTUS1_probe2

GAGCCTAACACTATCCTGTAATTCA

>MTUS1_probe3

GTCCCTGTCTATACATTCTCTGTAT

>MTUS1_probe4

TAACCTTTGTAATGTTCTTCACCAC

>MTUS1_probe5

ACTCTGCTCAGCCCTGTAACAGGGT

>MTUS1_probe6

TTTTACTTACCCATGTGAGCCTAAC

>MTUS1_probe7

TTCATTGCCTTTTTCACCTAAGCAT

>MTUS1_probe8

TTCTCTGTATCTTTTGGGGGTAACT

>MTUS1_probe9

AGGAAGAGCTTTGACTTGTCCCTGT

>MTUS1_probe10

GTTTTTCAGTGTTCAGCCATGTCAG

>MTUS1_probe11

ATTATGATCATCTACCACCAACTCT

>MTUS1_target

ttcattgcctttttcacctaagcatanggggaaaaactctcagggccctattaagatttataacctttgtaatgttcttcaccacagacaccttcttgtgagttttcagtctgactgtgggggtggggggtgtgaatgaaatggatgtcacagagtgtcatgtgtctgatgcagcctcctctgctgtgtattaaatgtcaaaatctgaatatatctggatatgtactaatcaaataataatcaatcaatcagcatatacatttcagccaaagccatagaagaaaaagcaatagttgcttgaattatgatcatctaccaccaactctgctcagccctgtaacagggtagggagagggtataacaggaagagctttgacttgtccctgtctatacattctctgtatcttttgggggtaacttcttggcagtttttcagtgttcagccatgtcagttgaaactagatttttctgtagattttttacttacccatgtgagcctaacactatcctgtaattca

>MTUS1_consensus

tgcacttgtaggtactgagaagcccaaatatctgcaacacagtggtcattccctagaagcagttgagggccagagtgttgagccatctttgccttttgtgtggaagcctaatgacaatttgaacngtncaggctactgtgatgccttggagctaaaccaaacatttgacatgacagtggataaagttaactgcacctttatatcacatcatgccatcggaaagagtcagtccttccatactgctggaagcctgccaccaactggtaggagaagtggaagtacatcttctttatcctattccncttggacatcttcccattctgatnagacgnnnnnnnnagaaactacttatgatagnagaaagctttgnaaaccctcaagtcacaccatcagaagnccnagacatgacttacacagcattntctgatgtggtgatgcaaagtgaggtttttgtntcagatattggaaatcagtgtgcangttcttcaggaaaggtcaccagtgagtacacagatggatcacaacaaagactagttggagaaaangagacacaagcactaacaccagtttctgatggcatggaagtccccaatgattctgcattacaagagttcttttgtttatcccatgatgaatccaatagcgaaccacattcacagagctcatacaggcacaaggaaatgggccaaaatctgagagagacagtgtcctattgtcttattgatgatgaatgccctttaatggtgccagcttttgataagagcgaagctcaagtgctgaacccagagcataaagtcactgagactgaagacncacaaatggtctccaaaggaaaggatttgggaacccaaaatcatacctcagaattgattctaagtagcccgccaggacaaaaggtgggctcgtcatttggactgacttgggatgcaaatgatatggtcattagcacagacaaaacgatgtgcatgtcaacaccagtcctagaacccacaaaagtaaccttttctgtttcaccgattgaagcgacggagaaatgtaagaaagtggagaagggtaatcgagggcttaaaaacataccagactcgaaggaggcacctgtgaacctgtgtaaacctagtttaggaaaatcaacaatcaaaacgaataccccnnnnnnnngcaaagttagaaaaactgaaattataagttacccaagaccaaacttcaagaatgtcaaagcaaaagttatgtctagagcagtgttgcagcccaaagatgctgctttatcaaaggtcacgcccagacctcagcagaccagtgcctcatcaccctcatcagtgaattcaagacaacaaacagtcttgagcagaacaccgagatctgacttgaatgcagacaaaaaagcagaaattctaattaacaagacacataagcagcagtttaataaactcattactagccaggctgtgcatgttacaactcattctaaaaatgcttcacacagggttccaagaacaacatctgccgtgaaatcgaatcaggaagatgttgacaaagccagttcttctaactcagcatgcgagaccgggtccgtttctgcgttgtttcagaagatcaaaggcatactccctgttaaaatggaaagtgcagaatgtttggaaatgacctatgttcccaacattgataggattagccctgaaaagaagggtgaaaaagaaaatgggacatctatggaaaaacaagagctgaaacaagagattatgaatgagacttttgaatatggttctctgtttttgggctctgcttcaaaaacaacgaccacctcaggtaggaatatatccaagcctgactcctgcggtttgaggcaaatagctgctccaaaagccaaagtggggccccctgtttcctgtttgaggcggaacagtgacaatagaaatcccagtgctgatcgagccgtatctcctcagaggatcaggcgtgtgtccagttctgttttgccaaaagctgctttctcatgattattctgattgatcatctccttcagagaacatgtgaaagcctacatccttgaaaactgcacagtcgtcatgggtgaatttgcctagaccacttcctaaatccaaagcatctttgaaaagtcctgcgctgcggaggacaggaagcaccccctcaatagccagcacccacagtgagctgagcacttacagcaacaattctggtaatgccgctgtcatcaaatatgaggagaaacctccaaaaccagcatttcagaatggttcctcaggatccttttatttgaagcctttggtatccagggctcatgttcacttgatgaaaactcctccaaaaggtccttcgagaaaaaatttatttacagctcttaatgcagtngaaaagagcaggcaaaagaatcctcgaagcttatgtatccagccacagacngctcccgatgcgctgccccctgagaaaacacttgaattgacgcaatataaaacaaaatgtgaaaaccaaagtggatttatcctgcagctcaagcagcttcttgcctgtggtaataccaagtttgaggcattgacagttgtgattcagcacctgctgtctgagcgggaggaagcactgaaacaacacaaaaccctatctcaagaacttgttaacctccggggagagctagtcactgcttcaaccacctgtgagaaattagaaaaagccaggaatgagttacaaacagtgtatgaagcattcgtccagcagcaccaggctgaaaaaacagaacgagagaatcggcttaaagagttttacaccagggagtatgaaaagcttcgggacacttacattgaagaagcagagaagtacaaaatgcaattgcaagagcagtttgacaacttaaatgctgcgcatgaaacctctaagttggaaattgaagctagccactcagagaaacttgaattgctaaagaaggcctatgaagcctccctttcagaaattaagaaaggccatgaaatagaaaagaaatcgcttgaagatttactttctgagaagcaggaatcgctagagaagcaaatcaatgatctgaagagtgaaaatgatgctttaaatgaaaaattgaaatcagaagaacaaaaaagaagagcaagagaaaaagcaaatttgaaaaatcctcagatcatgtatctagaacaggagttagaaagcctgaaagctgtgttagagatcaagaatgagaaactgcatcaacaggacatcaagttaatgaaaatggagaaactggtggacaacaacacagcattggttgacaaattgaagcgtttccagcaggagaatgaagaattgaaagctcggatggacaagcacatggcaatctcaaggcagctttccacggagcaggctgttctgcaagagtcgctggagaaggagtcgaaagtcaacaagcgactctctatggaaaacgaggagcttctgtggaaactgcacaatggggacctgtgtagccccaagagatcccccacatcctccgccatccctttgcagtcaccaaggaattcgggctccttccctagccccagcatttcacccagatgacacntccccaaagtccacagactctctgaaagcattttgatgcaggtctgcaggactgaccccaaggaggaacgtgggcacaagaggtatatcagcacacgtgtgatcaccgtagggtaactggagcgtcaccaccggcggaatcgcagcttctgagactggaantctggaggaagacttttgcctccgtccaaaagattcctccaaaaaaagatttaaaaaaagatttcggcatcgacacggacgttgttgcacaaagcacttaaagaacgagagcatcttgttcattgcctttttcacctaagcatanggggaaaaactctcagggccctattaagatttataacctttgtaatgttcttcaccacagacaccttcttgtgagttttcagtctgactgtgggggtggggggtgtgaatgaaatggatgtcacagagtgtcatgtgtctgatgcagcctcctctgctgtgtattaaatgtcaaaatctgaatatatctggatatgtactaatcaaataataatcaatcaatcagcatatacatttcagccaaagccatagaagaaaaagcaatagttgcttgaattatgatcatctaccaccaactctgctcagccctgtaacagggtagggagagggtataacaggaagagctttgacttgtccctgtctatacattctctgtatcttttgggggtaacttcttggcagtttttcagtgttcagccatgtcagttgaaactagatttttctgtagattttttacttacccatgtgagcctaacactatcctgtaattcattttctcaggctatgtgtaaatgtagaaccctaatttttctataaaaaaacaaactaactaactgtgtaaagaaagaaaaagggaagtaccaatgggtttttccaccttatttttacctttgatctacccttgcagatttaacctgtcttcttccctcccattattctcattttccttttacctttctccaccatccagagccacaaaagcaaaccttctacctcctacctacttttctctgggacaaggataaaggaatatgattttccagagccccagagccagctcatcttccaggtgctgaaaccactttccaaataaactaaagcctggatttgatattacaaattttgggaaatcttagaataaagaacgagaacaaggaagtcattggctagtataattaagaaaggtaggattcagtgcttaccgatgatgcagtacttgatagaagaaaacagtctgggaggatagcgctcatttttcagttaccctttaaggagtccctttgtctttgggaaagtagcagaatggtccgcttctttcccatgagtggaaaatgtggcttgtccaactctcctccaggttgcatttcagtttctttccaaaacttattacctcccctaatcctgagactttggaaaaggtggaaggaagaactgttgctttatctccccctccctgcatgtgtcaacattgtgatgtcagtatttactaatctacattcagtggctgtacaaataacagctgtagtaagaagagattcaggatgctagaggtgaatatttgggtcatttacatgtacactacatagcaagttgatactcatgttgcatgttcttttaaattagtgattttgtgtcttaagtctttaacttccaatacttcatcatgtatgtaaccttccatgtttgcttctgataaatggaaatgtaggttcactgccacttcatgagatatctctgctcacgcttccaagttgttctcaatgacattagccaaagttgggtttgccattcatcccctaggcatggtaaatcttgtgttgttccctgctgtcctccgtattacgtgaccggcaaataaatctcatagcagttaatataaaacatctttggaggatgggagagaacaggagggaagatgggaaacaaaatagagaattcttaagattttgtttaaaccaaatgtttcatgtagaatgcaaaatgttggcacgtcaaaaatatgaatgtgtagacaactgtagttgtgctcagtttgtagtgatgggaagtgtattttactctgatcaaataaataatgctggaatactcaanaaaaaaaaaaaaaaanaaaaaaaaaaaaaaaa

>PHB_probe1

GCAGGGGATGGCCTGATCGAGCTGC

>PHB_probe2

TGAGCGACGACCTTACAGAGCGAGC

>PHB_probe3

GACCTTCGGGAAGGAGTTCACAGAA

>PHB_probe4

GAGTTCACAGAAGCGGTGGAAGCCA

>PHB_probe5

CAGCCCCGATGATTCTTAACACAGC

>PHB_probe6

GCAGGTGAGCGACGACCTTACAGAG

>PHB_probe7

CAGGGGATGGCCTGATCGAGCTGCG

>PHB_probe8

GAGCAACAGAAAAAGGCGGCCATCA

>PHB_probe9

TCCTGGATGACGTGTCCTTGACACA

>PHB_probe10

TCGGGAAGGAGTTCACAGAAGCGGT

>PHB_probe11

TGGATGACGTGTCCTTGACACATCT

>PHB_target

gcaggtgagcgacgaccttacagagcgagccgccacctttgggctcatcctggatgacgtgtccttgacacatctgaccttcgggaaggagttcacagaagcggtggaagccaaacaggtggctcagcaggaagcagagagggnccagatttgtnggtgngaanaanggctgagcaacagaaaaaggcggccatcatctctgctgagggcgacntccaaggncagctgagctgattgccaactcactggccactgcaggggatggcctgatcgagctgcgcaagctggaagctgcagaggacatcgcgtaccagctctcacgctctcggaacatcacctacctgccagcggggcagtccgtgctcctccagctgccccagtgagggcccaccctgcctgcacctccgcgggctgactgggccacagccccgatgattcttaacacagc

>PHB_consensus

ttttttttttttttttgagccggaaggtctgggtgtcatttattgacagcagtttatacacatttgtttccttcccagtgcaggcatagagcccgtgagaagggcagtctctgaacgagggggccaggaacgtaggtcggacacgtctttggcaggcatgttcagccccagcaggatgcaatccttcaagttggaattccactgggtatctgagagggaccaaggtggggggtagagaggaagtcttcaacaggactcacatctcggtccaggcctcctctctcactatacaagtccatccaagttttaggaccctggggggcaacagtcaggcccacccacctcaacaggggcagattcttcaatctgtgcccagttcacacacacctgtttccgctggtcacacagttaaagagaatcagacttatggtaacagacagaccacttcctctctgcccctccccggcacctctcttcagcagcatctgcacaggaaccgctgggaagacggaggcctgccttctcagttcagccgcacatgccctcatccgcaaaccttccatcagtgacaggacggaggccacacgtggatctaggcagacacctgtgctccccagtccatcacatactgccccgcctgcgtgctgccaaatgctggtgcccaggccggaactccagcaggccccgaattgggacctaaagctggtttgcataggcacttggcaatttttggtagggaggtggataaaaaagtagatggatgtgagaagaatgaaagagtttcatttnntnnnntnnttagagatctgaagtgattttacctttatttccttcactttaagccaatcatgaaatttcacagtgatttctggggtgggagcagaaggaaggctgtgttaagaatcatcggggctgtggcccagtcagcccgcggaggtgcaggcagggtgggccctcactggggcagctggaggagcacggactgccccgctggcaggtaggtgatgttccgagagcgtgagagctggtacgcgatgtcctctgcagcttccagcttgcgcagctcgatcaggccatcccctgcagtggccagtgagttggcaatcagctcagctgnccttggangtcgccctcagcagagatgatggccgcctttttctgttgctcagccnttnttcncaccnacaaatctggnccctctctgcttcctgctgagccacctgtttggcttccaccgcttctgtgaactccttcccgaaggtcagatgtgtcaaggacacgtcatccaggatgagcccaaaggtggcggctcgctctgtaaggtcgtcgctcacctgcctggagaccagctctctctgggtgattagttctccagcatcaaagcgagccaccactgacttgaggatctcagttgtgatggacggcagcacacgctcatcatagtcctctccgatgctggtgaagatgcgaggaagctggctggcgacaggccggaagaggatgcgcagtgtgatgttgacattctgtaaatctttgctaccagtgatgactggcacattacgtggtcgagaacggcagtcaaagataattggtttctgtacccacgggatgagaaaatgagtcccttcccctaccacaatgtcctgcactccacggaatcggtcaaagatgacagctctgtgcccagcatccacattatataaggcagagttcaccacgcctcctgcaacagctaaggccaggccaaacttgccaatggactcaaacactttggcagccatgtttccttctgctggaccctctcacacctgcttccanctctgacctccacatgaattccccaaccnnncatnntgcnnannnncnncncnncnnnctccatgtcagctttcgtaatcaacttgttcaaacatcctcatctctcttgagcatgacaccctggcctactggtatattacaagaagcacagagctgggtataatataaggaccaacttaccaacttggacaaagacttgtaggagagactacctgctctgcattataaaaaagttaaactagccataaaaacctgttgttctgaccccagggtgtgccatcagctactttataaagcagctgtgagtctgaggaccannnttg

>GINS1_probe1

TGTTGAACTTGTATCCTTCAGCCTT

>GINS1_probe2

TAATATTGAGTCTTCTGGCCTATAA

>GINS1_probe3

GGTCTGTCTTCCTAGGTATTAATGT

>GINS1_probe4

AGTTTTCAGTGTACAGGTCTACCAT

>GINS1_probe5

GCCTTGCTAAACTGTGAGTTCTCAT

>GINS1_probe6

GGCCTATAAACAAGGTCTGTCTTCC

>GINS1_probe7

GTAGTCACAGTTACACGGCAGGCTG

>GINS1_probe8

GTTGGGCACCTTGATTGAGATTGCA

>GINS1_probe9

AATTCTAACCACTTGTTGCTAGTAA

>GINS1_probe10

AGGTCTACCATGTCAGCATTTCATA

>KIAA0101_probe1

AATGGTGCCATATTGTCACTCCTTC

>KIAA0101_probe2

ACCAGCCCAGGCAACATAGCGTAAA

>KIAA0101_probe3

GTGTTTGTTCCAATTAGCTTTGTTG

>KIAA0101_probe4

TAGGTTGTCCCCTAAAGATTCTGAA

>KIAA0101_probe5

TGCTTAGATTGTTGTACTGCTGCCA

>KIAA0101_probe6

TTAAACGGTTGATAATGCCTCTACA

>KIAA0101_probe7

TATTCTACCCTCTTTTTTGGCAAGG

>KIAA0101_probe8

CAAGTCATTGCATTGTGTTCTAATT

>KIAA0101_probe9

CATAGCGTAAACCCTATCTCTAAAA

>KIAA0101_probe10

AACCTTGGATGGATATCTTCTCTTT

>KIAA0101_probe11

ATTGTTGTACTGCTGCCATTTTTAT

>KIAA0101_probe12

CACAGTGGCTTCTCAGGAGGCTGAG

>KIAA0101_probe13

GGATAGAATCATGGTGGGCACAGTG

>KIAA0101_probe14

TCTCCTTGTTTACCCTGGTATTCTA

>KIAA0101_probe15

AAGTGTCTAGTTCTTGCTAAAATCA

>KIAA0101_probe16

TGGAGAATTCTTTAGGTTGTCCCCT

>KIAA0101_probe17

GGAGGGAGGTTTGCTTGAGTCCAGG

>KIAA0101_probe18

TGGCAAGGAGGACAAATACGCAATG

>KIAA0101_probe19

TCATCTTTGAATAACGTCTCCTTGT

>KIAA0101_probe20

GATAATGCCTCTACAACAACAAGAA

>SCD_probe1

TGAACTTGATACGTCCGTGTGTCCC

>SCD_probe2

GGGCAGTTTTGAGGCATGACTAATG

>SCD_probe3

AAAAGCGAGGTGGCCATGTTATGCT

>SCD_probe4

TAACTATAAGGTGCCTCAGTTTTCC

>SCD_probe5

AGATGCTGTCATTAGTCTATATGGT

>SCD_probe6

GGAATTCTCAAGACCTGAGTATTTT

>SCD_probe7

CTGACCTACCTCAAAGGGCAGTTTT

>SCD_probe8

ACAACGCATTGCCACGGAAACATAC

>SCD_probe9

AGCATTTTGGGATCCTTCAGCACAG

>SCD_probe10

GAAGCTAATTGTACTAATCTGAGAT

>SCD_probe11

ATGTCCACCATGAACTTGATACGTC

>SCD_target

aaaagcgaggtggccatgttatgctggtggttgccagggcctccaaccactgtgccactgacttgctgtgtgaccctgggcaagtcacttaactataaggtgcctcagttttccttctgttaaaatggggataataatactgacctacctcaaagggcagttttgaggcatgactaatgctttttagaaagcattttgggatccttcagcacaggaattctcaagacctgagtattttttataataggaatgtccaccatgaacttgatacgtccgtgtgtcccagatgctgtcattagtctatatggttctccaagaaactgaatgaatccattggagaagcggtggataactagccagacaaaatttgagaatacataaacaacgcattgccacggaaacatacagaggatgccttttctgtgattgggtgggattttttccctttttatgtgggatatagtagttacttgtgacaagaataattttggaataatttctattaatatcaactctgaagctaattgtactaatctgagat

>SCD_exemplar

gtggtgtcggtgtcggcagcatccccggcgccctgctgcggtcgccggagccctcggcctctgttctcctccccctcccgcccttacctccacgcgggaccgcccgcgccagtcaactcctcgcactttgcccctgcttggcagcggataaaagggggctgaggaaataccggacacgtccacccgttgccagctctagcctttaaattcccggctcgggacctccacgcaccgggctagcgccgacaaccagctagcgtgcaaggcgccgcggctcagcgcgtaccggcgggcttcgaaaccgcagtcctccggcgaccccgaactccgctccggagcctcagccccctggaaagtgatcccggcatcggagagccaagatgccggcccacttgctgcaggacgatatctctagctcctataccaccaccaccaccattacagcgcctccctccagggtcctgcagaatggaggagataagttggagacgatgcccctctacttggaagacgacattcgccctgatataaaagatgatatatatgaccccacctacaaggataaggaaggcccaagccccaaggttgaatatgtctggagaaacatcatccttatgtctctgctacacttgggagccctgtatgggatcactttgattcctacctgcaagttctacacctggctttggggggtattctactattttgtcagtgccctgggcataacagcaggagctcatcgtctgtggagccaccgctcttacaaagctcggctgcccctacggctctttctgatcattgccaacacaatggcattccagaatgatgtctatgaatgggctcgtgaccaccgtgcccaccacaagttttcagaaacacatgctgatcctcataattcccgacgtggctttttcttctctcacgtgggttggctgcttgtgcgcaaacacccagctgtcaaagagaaggggagtacgctagacttgtctgacctagaagctgagaaactggtgatgttccagaggaggtactacaaacctggcttgctgctgatgtgcttcatcctgcccacgcttgtgccctggtatttctggggtgaaacttttcaaaacagtgtgttcgttgccactttcttgcgatatgctgtggtgcttaatgccacctggctggtgaacagtgctgcccacctcttcggatatcgtccttatgacaagaacattagcccccgggagaatatcctggtttcacttggagctgtgggtgagggcttccacaactaccaccactcctttccctatgactactctgccagtgagtaccgctggcacatcaacttcaccacattcttcattgattgcatggccgccctcggtctggcctatgaccggaagaaagtctccaaggccgccatcttggccaggattaaaagaaccggagatggaaactacaagagtggctgagtttggggtccctcaggttcctttttcaaaaaccagccaggcagaggttttaatgtctgtttattaactactgaataatgctaccaggatgctaaagatgatgatgttaacccattccagtacagtattcttttaaaattcaaaagtattgaaagccaacaactctgcctttatgatgctaagctgatattatttcttctcttatcctctctctcttctaggcccattgtcctccttttcactttaatcgccctcctttcccttattgcctcccaggcaagcagctggtcagtctttgctcagtgtccagcttccaaagcctagacaacctttctgtagcctaaaacgaatggtctttgctccagataactctctttccttgagctgttgtgagctttgaagtaggtggcttgagctagagataaaacagaatcttctgggtagtcccctgttgattatcttcagcccaggcttttgctagatggaatggaaaagcaacttcatttgacacaaagcttctaaagcnaggtaaattgtcgggggagagagttagcatgtatgaatgtaaggatgagggaagcgaaggaacctctcgccatgatcagacatacagctgcctacctaatgaggacttcaagccccaccacatagcatgcttcctttctctcctggctcggggtaaaaagtggctgcggtgtttggcaatgctaattcaatgccgcaacatatagttgaggccgaggataaagaaaagacattttaagtttgtagtaaaagtggtctctgctggggaagggttttcttttctttttttctttaataacaaggagatttcttagttcatatatcaagaagtcttgaagttgggtgtttccagaattggtaaaaacagcagctcatagaattttgagtattccatgagctgctcattacagttctttcctctttctgctctgccatcttcaggatattggttcttcccctcatagtaataagatggctgtggcatttccaaacatccaaaaaaagggaaggatttaaggaggtgaagtcgggtcaaaaataaaatatatatacatatatacattgcttagaacgttaaactattagagtatttcccttccaaagagggatgtttggaaaaaactctgaaggagaggaggaattagttgggatgccaatttcctctccactgctggacatgagatggagaggctgagggacaggatctataggcagcttctaagagcgaacttcacataggaagggatctgagaacacgttcaggggttgagaaggttactgagtgagttattgggagtcttaataaactagatattaggtccattcattaattagttccagtttctccttgaaatgagtaaaaactagaaggcttctctccacagtgttgtgccccttcactcatttttttttgaggagaagggggtctctgttaacatctagcctaaagtatacaaactgcctggggggcagggttaggaatctcttcactaccctgattcttgattcctggctctaccctgtctgtcccttttctttgaccagatctttctcttccctgaacgttttcttctttccctggacaggcagcctcctttgtgtgtattcagaggcagtgatgacttgctgtccaggcagctccctcctgcacacagaatgctcagggtcactgaaccactgcttctcttttgaaagtagagctagctgccactttcacgtggcctccgcagtgtctccacctacacccctgtgctcccctgccacactgatggctcaagacaaggctggcaaaccctcccagaaacatctctggcccagaaagcctctctctccctccctctctcatgagaagccaagcgctcatgttgagccagtgggccagccacagagcaaaagagggtttattttcagtcccctctctctgggtcagaaccagagggcatgctgaatgccccctgcttacttggtgagggtgccccgcctgagtcagtgctctcagctggcagtgcaatgcttgtagaagtaggaggaaacagttctcactgggaagaagcaagggcaagaacccaagtgcctcacctcgaaaggaggccctgttccctggagtcagggtgaactgcaaagctttggctgagacctgggatttgagataccacaaaccctgctgaacacagtgtctgttcagcaaactaaccagcattccctacagcctagggcagacaatagtatagaagtctggaaaaaaacaaaaacagaatttgagaaccttggaccactcctgtccctgtagctcagtcatcaaagcagaagtctggctttgctctattaagattggaaatgtacactaccaaacactcagtccactgttgagccccagtgctggaagggaggaaggcctttcttctgtgttaattgcgtagaggctacaggggttagcctggactaaaggcatccttgtctttgagctattcacctcagtagaaaaggatctaagggaagatcactgtagtttagttctgttgacctgtgcacctaccccttggaaatgtctgctggtatttctaattccacaggtcatcagatgcctgcttgataatatataaacaataaaaacaactttcacttcttcctattgtaatcgtgtgccatggatctgatctgtaccatgaccctacataaggctggatggcacctcaggctgagggccccaatgtatgtgtggctgtgggtgtgggtgggagtgtgtctgctgagtaaggaacacgattttcaagattctaaagctcaattcaagtgacacattaatgataaactcagatctgatcaagagtccggatttctaacagtccttgctttggggggtgtgctggcaacttagctcaggtgccttacatcttttctaatcacagtgttgcatatgagcctgccctcactccctctgcagaatccctttgcacctgagaccctactgaagtggctggtagaaaaaggggcctgagtggaggattatcagtatcacgatttgcaggattcccttctgggcttcattctggaaacttttgttagggctgcttttcttaagtgcccacatttgatggagggtggaaataatttgaatgtatttgatttataagtttttttttttttttgggttaaaagatggttgtagcatttaaaatggaaaattttctccttggtttgctagtatcttgggtgtattctctgtaagtgtagctcaaataggtcatcatgaaaggttaaaaaagcgaggtggccatgttatgctggtggttgccagggcctccaaccactgtgccactgacttgctgtgtgaccctgggcaagtcacttaactataaggtgcctcagttttccttctgttaaaatggggataataatactgacctacctcaaagggcagttttgaggcatgactaatgctttttagaaagcattttgggatccttcagcacaggaattctcaagacctgagtattttttataataggaatgtccaccatgaacttgatacgtccgtgtgtcccagatgctgtcattagtctatatggttctccaagaaactgaatgaatccattggagaagcggtggataactagccagacaaaatttgagaatacataaacaacgcattgccacggaaacatacagaggatgccttttctgtgattgggtgggattttttccctttttatgtgggatatagtagttacttgtgacaagaataattttggaataatttctattaatatcaactctgaagctaattgtactaatctgagattgtgtttgttcataataaaagtgaagtgaatctgattgcactg

>PTTG1_probe1

CATTCTGTCGACCCTGGATGTTGAA

>PTTG1_probe2

TTGAGAGTTTTGACCTGCCTGAAGA

>PTTG1_probe3

AATTGCCACCTGTTTGCTGTGACAT

>PTTG1_probe4

GTGCCTCTCATGATCCTTGACGAGG

>PTTG1_probe5

TGCAGTCTCCTTCAAGCATTCTGTC

>PTTG1_probe6

CCTGCCTCAGATGATGCCTATCCAG

>PTTG1_probe7

AAAACAGCCAAGCTTTTCTGCCAAA

>PTTG1_probe8

GGGAATCCAATCTGTTGCAGTCTCC

>PTTG1_probe9

TGAAGAGCACCAGATTGCGCACCTC

>PTTG1_probe10

AAGCAAAAAGCTCTGTTCCTGCCTC

>PTTG1_probe11

TTCCCTTCAATCCTCTAGACTTTGA

>PTTG1_target

aaaacagccaagcttttctgccaaaaagatgactgagaagactgttaaagcaaaaagctctgttcctgcctcagatgatgcctatccagaaatagaaaaattctttcccttcaatcctctagactttgagagttttgacctgcctgaagagcaccagattgcgcacctccccttgagtggagtgcctctcatgatccttgacgaggagagagagcttgaaaagctgtttcagctgggccccccttcacctgtgaagatgccctctccaccatgggaatccaatctgttgcagtctccttcaagcattctgtcgaccctggatgttgaattgccacctgtttgctgtgacat

>PTTG1_exemplar

gcggcctcagatgaatgcggctgttaagacctgcaataatccagaatggctactctgatctatgttgataaggaaaatggagaaccaggcacccgtgtggttgctaaggatgggctgaagctggggtctggaccttcaatcaaagccttagatgggagatctcaagtttcaacaccacgttttggcaaaacgttcgatgccccaccagccttacctaaagctactagaaaggctttgggaactgtcaacagagctacagaaaagtctgtaaagaccaagggacccctcaaacaaaaacagccaagcttttctgccaaaaagatgactgagaagactgttaaagcaaaaagctctgttcctgcctcagatgatgcctatccagaaatagaaaaattctttcccttcaatcctctagactttgagagttttgacctgcctgaagagcaccagattgcgcacctccccttgagtggagtgcctctcatgatccttgacgaggagagagagcttgaaaagctgtttcagctgggccccccttcacctgtgaagatgccctctccaccatgggaatccaatctgttgcagtctccttcaagcattctgtcgaccctggatgttgaattgccacctgtttgctgtgacatagatatttaaatttcttagtgcttcagagtttgtgtgtatttgtattaataaagcattcttcaacagaaaaaaaaaaaaaaaaa

>CENPF_probe1

GGTCAAAGTTGCTCAGCGGAGCCCA

>CENPF_probe2

TGCACAGAAGTTAGCGCTATCCCCA

>CENPF_probe3

TACCCCTGGGAGGTGCCAGTCATTG

>CENPF_probe4

GTTTGGAAGCACTGATCACCTGTTA

>CENPF_probe5

GAAGGCACTTTGTGTGTCAGTACCC

>CENPF_probe6

GATCACCTGTTAGCATTGCCATTCC

>CENPF_probe7

GAGCCCAGTAGATTCAGGCACCATC

>CENPF_probe8

GTACTCTTTAGATCTCCCATGTGTA

>CENPF_probe9

TGAGGGTCAAGCGAGGCCGACTTGT

>CENPF_probe10

TTGCCATTCCTCTACTGCAATGTAA

>CENPF_probe11

CGAAATCCGTCCCAGTCAATAATCT

>CENPF_target

tgcacagaagttagcgctatccccactgagtctcggcaaagaaaatcttgcagagtcctccaaaccaacagctggtggcagcagatcacaaaaggtcaaagttgctcagcggagcccagtagattcaggcaccatcctccgagaacccaccacgaaatccgtcccagtcaataatcttcctgagagaagtccgactgacagccccagagagggcctgagggtcaagcgaggccgacttgtccccagccccaaagctggactggagtccaagggcagtgagaactgtaaggtccagtgaaggcactttgtgtgtcagtacccctgggaggtgccagtcattgaatagataaggctgtgcctacaggacttctctttagtcagggcatgctttattagtgaggagaaaacaattccttagaagtcttaaatatattgtactctttagatctcccatgtgtaggtattgaaaaagtttggaagcactgatcacctgttagcattgccattcctctactgcaatgtaa

>CENPF_exemplar

ggagaagcgggcgaattgggcaccggtggcggctgcgggcagtttgaattagactctgggctccagcccgccgaagccgcgccagaactgtactctccgagaggtcgttttcccgtccccgagagcaagtttatttacaaatgttggagtaataaagaaggcagaacaaaatgagctgggctttggaagaatggaaagaagggctgcctacaagaactcttcagaaaattcaagagcttgaaggacagcttgacaaactgaagaaggaaaagcagcaaaggcagtttcagcttgacagtctcgaggctgcgccgcagaagcaaacacagaaggttgaaaatgaaaaaaccgagggtacaaacctgaaaagggagaatcaaagattgatggaaatatgtgaaagtctggagaaaactaagcagaagatttctcatgaacttcaagtcaaggagtcacaagtgaatttccaggaaggacaactgaattcaggcaaaaaacaaatagaaaaactggaacaggaacttaaaaggtgtaaatctgagcttgaaagaagccaacaagctgcgcagtctgcagatgtctctctgaatccatgcaatacaccacaaaaaatttttacaactccactaacaccaagtcaatattatagtggttccaagtatgaagatctaaaagaaaaatataataaagaggttgaagaacgaaaaagattagaggcagaggttaaagccttgcaggctaaaaaagcaagccagactcttccacaagccaccatgaatcaccgcgacattgcccggcatcaggcttcatcatctgtgttctcatggcagcaagagaagaccccaagtcatctttcatctaattctcaaagaactccaattaggagagatttctctgcatcttacttttctggggaactagaggtgactccaagtcgatcaactttgcaaatagggaaaagagatgctaatagcagtttctttggcaattctagcagtcctcatcttttggatcaattaaaagcgcagaatcaagagctaagaaacaagattaatgagttggaactacgcctgcaaggacatgaaaaagaaatgaaaggccaagtgaataagtttcaagaactccaactccaactggagaaagcaaaagtggaattaattgaaaaagagaaagttttgaacaaatgtagggatgaactagtgagaacaacagcacaatacgaccaggcgtcaaccaagtatactgcattggaacaaaaactgaaaaaattgacggaagatttgagttgtcagcgacaaaatgcagaaagtgccagatgttctctggaacagaaaattaaggaaaaagaaaaggagtttcaagaggagctctcccgtcaacagcgttctttccaaacactggaccaggagtgcatccagatgaaggccagactcacccaggagttacagcaagccaagaatatgcacaacgtcctgcaggctgaactggataaactcacatcagtaaagcaacagctagaaaacaatttggaagagtttaagcaaaagttgtgcagagctgaacaggcgttccaggcgagtcagatcaaggagaatgagctgaggagaagcatggaggaaatgaagaaggaaaacaacctccttaagagtcactctgagcaaaaggccagagaagtctgccacctggaggcagaactcaagaacatcaaacagtgtttaaatcagagccagaattttgcagaagaaatgaaagcgaagaatacctctcaggaaaccatgttaagagatcttcaagaaaaaataaatcagcaagaaaactccttgactttagaaaaactgaagcttgctgtggctgatctggaaaagcagcgagattgttctcaagaccttttgaagaaaagagaacatcacattgaacaacttaatgataagttaagcaagacagagaaagagtccaaagccttgctgagtgctttagagttaaaaaagaaagaatatgaagaattgaaagaagagaaaactctgttttcttgttggaaaagtgaaaacgaaaaacttttaactcagatggaatcagaaaaggaaaacttgcagagtaaaattaatcacttggaaacttgtctgaagacacagcaaataaaaagtcatgaatacaacgagagagtaagaacgctggagatggacagagaaaacctaagtgtcgagatcagaaaccttcacaacgtgttagacagtaagtcagtggaggtagagacccagaaactagcttatatggagctacagcagaaagctgagttctcagatcagaaacatcagaaggaaatagaaaatatgtgtttgaagacttctcagcttactgggcaagttgaagatctagaacacaagcttcagttactgtcaaatgaaataatggacaaagaccggtgttaccaagacttgcatgccgaatatgagagcctcagggatctgctaaaatccaaagatgcttctctggtgacaaatgaagatcatcagagaagtcttttggcttttgatcagcagcctgccatgcatcattcctttgcaaatataattggagaacaaggaagcatgccttcagagaggagtgaatgtcgtttagaagcagaccaaagtccgaaaaattctgccatcctacaaaatagagttgattcacttgaattttcattagagtctcaaaaacagatgaactcagacctgcaaaagcagtgtgaagagttggtgcaaatcaaaggagaaatagaagaaaatctcatgaaagcagaacagatgcatcaaagttttgtggctgaaacaagtcagcgcattagtaagttacaggaagacacttctgctcaccagaatgttgttgctgaaaccttaagtgcccttgagaacaaggaaaaagagctgcaacttttaaatgataaggtagaaactgagcaggcagagattcaagaattaaaaaagagcaaccatctacttgaagactctctaaaggagctacaacttttatccgaaaccctaagcttggagaagaaagaaatgagttccatcatttctttaaataaaagggaaattgaagagctgacccaagagaatgggactcttaaggaaattaatgcatccttaaatcaagagaagatgaacttaatccagaaaagtgagagttttgcaaactatatagatgaaagggagaaaagcatttcagagttatctgatcagtacaagcaagaaaaacttattttactacaaagatgtgaagaaaccggaaatgcatatgaggatcttagtcaaaaatacaaagcagcacaggaaaagaattctaaattagaatgcttgctaaatgaatgcactagtctttgtgaaaataggaaaaatgagttggaacagctaaaggaagcatttgcaaaggaacaccaagaattcttaacaaaattagcatttgctgaagaaagaaatcagaatctgatgctagagttggagacagtgcagcaagctctgagatctgagatgacagataaccaaaacaattctaagagcgaggctggtggtttaaagcaagaaatcatgactttaaaggaagaacaaaacaaaatgcaaaaggaagttaatgacttattacaagagaatgaacagctgatgaaggtaatgaagactaaacatgaatgtcaaaatctagaatcagaaccaattaggaactctgtgaaagaaagagagagtgagagaaatcaatgtaattttaaacctcagatggatcttgaagttaaagaaatttctctagatagttataatgcgcagttggtgcaattagaagctatgctaagaaataaggaattaaaacttcaggaaagtgagaaggagaaggagtgcctgcagcatgaattacagacaattagaggagatcttgaaaccagcaatttgcaagacatgcagtcacaagaaattagtggccttaaagactgtgaaatagatgcggaagaaaagtatatttcagggcctcatgagttgtcaacaagtcaaaacgacaatgcacaccttcagtgctctctgcaaacaacaatgaacaagctgaatgagctagagaaaatatgtgaaatactgcaggctgaaaagtatgaactcgtaactgagctgaatgattcaaggtcagaatgtatcacagcaactaggaaaatggcagaagaggtagggaaactactaaatgaagttaaaatattaaatgatgacagtggtcttctccatggtgagttagtggaagacataccaggaggtgaatttggtgaacaaccaaatgaacagcaccctgtgtctttggctccattggacgagagtaattcctacgagcacttgacattgtcagacaaagaagttcaaatgcactttgccgaattgcaagagaaattcttatctttacaaagtgaacacaaaattttacatgatcagcactgtcagatgagctctaaaatgtcagagctgcagacctatgttgactcattaaaggccgaaaatttggtcttgtcaacgaatctgagaaactttcaaggtgacttggtgaaggagatgcagctgggcttggaggaggggctcgttccatccctgtcatcctcttgtgtgcctgacagctctagtcttagcagtttgggagactcctccttttacagagctcttttagaacagacaggagatatgtctcttttgagtaatttagaaggggctgtttcagcaaaccagtgcagtgtagatgaagtattttgcagcagtctgcagacctatgttgactcattaaaggccgaaaatttggtcttgtcaacgaatctgagaaactttcaaggtgacttggtgaaggagatgcagctgggcttggaggaggggctcgttccatccctgtcatcctcttgtgtgcctgacagctctagtcttagcagtttgggagactcctccttttacagagctcttttagaacagacaggagatatgtctcttttgagtaatttagaaggggttgtttcagcaaaccagtgcagtgtagatgaagtattttgcagcagtctgcaggaggagaatctgaccaggaaagaaaccccttcggccccagcgaagggtgttgaagagcttgagtccctctgtgaggtgtaccggcagtccctcgagaagctagaagagaaaatggaaagtcaagggattatgaaaaataaggaaattcaagagctcgagcagttattaagttctgaaaggcaagagcttgactgccttaggaagcagtatttgtcagaaaatgaacagtggcaacagaagctgacaagcgtgactctggagatggagtccaagttggcggcagaaaagaaacagacggaacaactgtcacttgagctggaagtagcacgactccagctacaaggtctggacttaagttctcggtctttgcttggcatcgacacagaagatgctattcaaggccgaaatgagagctgtgacatatcaaaagaacatacttcagaaactacagaaagaacaccaaagcatgatgttcatcagatttgtgataaagatgctcagcaggacctcaatctagacattgagaaaataactgagactggtgcagtgaaacccacaggagagtgctctggggaacagtccccagataccaattatgagcctccaggggaagataaaacccagggctcttcagaatgcatttctgaattgtcattttctggtcctaatgctttggtacctatggatttcctggggaatcaggaagatatccataatcttcaactgcgggtaaaagagacatcaaatgagaatttgagattacttcatgtgatagaggaccgtgacagaaaagttgaaagtttgctaaatgaaatgaaagaattagactcaaaactccatttacaggaggtacaactaatgaccaaaattgaagcatgcatagaattggaaaaaatagttggggaacttaagaaagaaaactcagatttaagtgaaaaattggaatatttttcttgtgatcaccaggagttactccagagagtagaaacttctgaaggcctcaattctgatttagaaatgcatgcagataaatcatcacgtgaagatattggagataatgtggccaaggtgaatgacagctggaaggagagatttcttgatgtggaaaatgagctgagtaggatcagatcggagaaagctagcattgagcatgaagccctctacctggaggctgacttagaggtagttcaaacagagaagctatgtttagaaaaagacaatgaaaataagcagaaggttattgtctgccttgaagaagaactctcagtggtcacaagtgagagaaaccagcttcgtggagaattagatactatgtcaaaaaaaaccacggcactggatcagttgtctgaaaaaatgaaggagaaaacacaagagcttgagtctcatcaaagtgagtgtctccattgcattcaggtggcagaggcagaggtgaaggaaaagacggaactccttcagactttgtcctctgatgtgagtgagctgttaaaagacaaaactcatctccaggaaaagctgcagagtttggaaaaggactcacaggcactgtctttgacaaaatgtgagctggaaaaccaaattgcacaactgaataaagagaaagaattgcttgtcaaggaatctgaaagcctgcaggccagactgagtgaatcagattatgaaaagctgaatgtctccaaggccttggaggccgcactggtggagaaaggtgagttcgcattgaggctgagctcaacacaggaggaagtgcatcagctgagaagaggcatcgagaaactgagagttcgcattgaggccgatgaaaagaagcagctgcacatcgcagagaaactgaaagaacgcgagcgggagaatgattcacttaaggataaagttgagaaccttgaaagggaattgcagatgtcagaagaaaaccaggagctagtgattcttgatgccgagaattccaaagcagaagtagagactctaaaaacacaaatagaagagatggccagaagcctgaaagtttttgaattagaccttgtcacgttaaggtctgaaaaagaaaatctgacaaaacaaatacaagaaaaacaaggtcagttgtcagaactagacaagttactctcttcatttaaaagtctgttagaagaaaaggagcaagcagagatacagatcaaagaagaatctaaaactgcagtggagatgcttcagaatcagttaaaggagctaaatgaggcagtagcagccttgtgtggtgaccaagaaattatgaaggccacagaacagagtctagacccaccaatagaggaagagcatcagctgagaaatagcattgaaaagctgagagcccgcctagaagctgatgaaaagaagcagctctgtgtcttacaacaactgaaggaaagtgagcatcatgcagatttacttaagggtagagtggagaaccttgaaagagagctagagatagccaggacaaaccaagagcatgcagctcttgaggcagagaattccaaaggagaggtagagaccctaaaagcaaaaatagaagggatgacccaaagtctgagaggtctggaattagatgttgttactataaggtcagaaaaagaagatctgacaaatgaattacaaaaagagcaagagcgaatatctgaattagaaataataaattcatcatttgaaaatattttgcaagaaaaagagcaagagaaagtacagatgaaagaaaaatcaagcactgccatggagatgcttcaaacacaattaaaagagctcaatgagagagtggcagccctgcataatgaccaagaagcctgtaaggccaaagagcagaatcttagtagtcaagtagagtgtcttgaacttgagaaggctcagttgctacaaggccttgatgaggccaaaaataattatattgttttgcaatcttcagtgaatggcctcattcaagaagtagaagatggcaagcagaaactggagaagaaggatgaagaaatcagtagactgaaaaatcaaattcaagaccaagagcagcttgtctctaaactgtcccaggtggaaggagagcaccaactttggaaggagcaaaacttagaactgagaaatctgacagtggaattggagcagaagatccaagtgctacaatccaaaaatgcctctttgcaggacacattagaagtgctgcagagttcttacaagaatctagagaatgagcttgaattgacaaaaatggacaaaatgtcctttgttgaaaaagtaaacaaaatgactgcaaaggaaactgagctgcagagggaaatgcatgagatggcacagaaaacagcagagctgcaagaagaactcagtggagagaaaaataggctagctggagagttgcagttactgttggaagaaataaagagcagcaaagatcaattgaaggagctcacactagaaaatagtgaattgaagaagagcctagattgcatgcacaaagaccaggtggaaaaggaagggaaagtgagagaggaaatagctgaatatcagctacggcttcatgaagctgaaaagaaacaccaggctttgcttttggacacaaacaaacagtatgaagtagaaatccagacataccgagagaaattgacttctaaagaagaatgtctcagttcacagaagctggagatagaccttttaaagtctagtaaagaagagctcaataattcattgaaagctactactcagattttggaagaattgaagaaaaccaagatggacaatctaaaatatgtaaatcagttgaagaaggaaaatgaacgtgcccaggggaaaatgaagttgttgatcaaatcctgtaaacagctggaagaggaaaaggagatactgcagaaagaactctctcaacttcaagctgcacaggagaagcagaaaacaggtactgttatggataccaaggtcgatgaattaacaactgagatcaaagaactgaaagaaactcttgaagaaaaaaccaaggaggcagatgaatacttggataagtactgttccttgcttataagccatgaaaagttagagaaagctaaagagatgttagagacacaagtggcccatctgtgttcacagcaatctaaacaagattcccgagggtctcctttgctaggtccagttgttccaggaccatctccaatcccttctgttactgaaaagaggttatcatctggccaaaataaagcttcaggcaagaggcaaagatccagtggaatatgggagaatggtggaggaccaacacctgctaccccagagagcttttctaaaaaaagcaagaaagcagtcatgagtggtattcaccctgcagaagacacggaaggtactgagtttgagccagagggacttccagaagttgtaaagaaagggtttgctgacatcccgacaggaaagactagcccatatatcctgcgaagaacaaccatggcaactcggaccagcccccgcctggctgcacagaagttagcgctatccccactgagtctcggcaaagaaaatcttgcagagtcctccaaaccaacagctggtggcagcagatcacaaaaggtcaaagttgctcagcggagcccagtagattcaggcaccatcctccgagaacccaccacgaaatccgtcccagtcaataatcttcctgagagaagtccgactgacagccccagagagggcctgagggtcaagcgaggccgacttgtccccagccccaaagctggactggagtccaagggcagtgagaactgtaaggtccagtgaaggcactttgtgtgtcagtacccctgggaggtgccagtcattgaatagataaggctgtgcctacaggacttctctttagtcagggcatgctttattagtgaggagaaaacaattccttagaagtcttaaatatattgtactctttagatctcccatgtgtaggtattgaaaaagtttggaagcactgatcacctgttagcattgccattcctctactgcaatgtaaatagtataaagctatgtatataaagctttttggtaatatgttacaattaaaatgacaagcactatat

>SMC4_probe1

GGACAGTGTTTCAACAAGCCTAGGC

>SMC4_probe2

GCATCTAAGGGACTTTGTTGAACTT

>SMC4_probe3

GATGGCCTCTGATTTACACTGGTTC

>SMC4_probe4

AGAAGTCTGCCCTAGCTGTTAAATT

>SMC4_probe5

GAGTTAATTGTTCCTTTCTTCAGTG

>SMC4_probe6

TAGACAGCTTGGATCCTTTCTCTGA

>SMC4_probe7

GGTTTACCAGGATGTAGTCCCACTG

>SMC4_probe8

GAAAACACTTAGTTCATTGGCTTTA

>SMC4_probe9

GCGTATTTTTACACTATTGGCTCAA

>SMC4_probe10

ATTTACACAGCTAGATTTGGAAGAT

>SMC4_probe11

GGATGAGATTGATGCAGCCCTTGAT

>SMC4_probe12

ATTGATGCAGCCCTTGATTTTAAAA

>SMC4_probe13

AGTTCATAATAATTTCTCTTCGAAA

>SMC4_probe14

GGAAGGACTTTCGGTATTGTATTAG

>SMC4_probe15

CCTTTCTTCAGTGGGCCATTGTTTT

>SMC4_probe16

TTAGTATTTGCTCTTCACCACTACA

>SMC4_probe17

GATACCTTGAGTAATGTTTGCCTAT

>SMC4_probe18

CACTCCCCTTTACTTCATGGATGAG

>SMC4_probe19

AAGCCTAGGCTATCTCGTAAGTTGA

>SMC4_probe20

GGACGCCGAACTCGAGCTTGTAGAC

>SMC4_probe21

AATATCCCACTATAGTTGCTTCATG

>NCAPG_probe1

CCCAATTTCTCAATGAAGATCTAAG

>NCAPG_probe2

GATTATGTCCAGTTATTTGCTTTAA

>NCAPG_probe3

GGTGGAATCCTTTAAGATTATGTCC

>NCAPG_probe4

AAGACGATGGAGGTGGAATCCTTTA

>NCAPG_probe5

GAGCCAAAACCGCAGCACTAGAAAA

>NCAPG_probe6

GAGACTACCAAGACGAGCCAAAACC

>NCAPG_probe7

TTCCAGAACCAGAATCAGAAATGAA

>NCAPG_probe8

CCAAGACGAGCCAAAACCGCAGCAC

>NCAPG_probe9

GGACGAACAGGAGGTGTCAGACTGC

>NCAPG_probe10

GAAGATGAGACTACCAAGACGAGCC

>NCAPG_probe11

GTGTCAGACTGCTGAAGCCGACTCT

>NCAPG_target

ggacgaacaggaggtgtcagactgctgaagccgactctgaaagtgatcatgaagttccagaaccagaatcagaaatgaagatgagactaccaagacgagccaaaaccgcagcactagaaaaaagtaaacttaaccttgcccaatttctcaatgaagatctaagttaggaaagacgatggaggtggaatcctttaagattatgtccagttatttgctttaa

>NCAPG_exemplar

atagaagactcctcggagagcgctgcctctgggttggcgggctggcaggctgtagccgagcgcgggcaggactcgtcccggcagggttccagagccatgggagcggaaaggaggctgctgtcgattaaggaggcctttcggctggcgcagcagccgcaccagaaccaggcgaagctggtggtggcgctgagccgcacctaccgcacgatggatgataagacagtttttcatgaggagttcattcattaccttaaatatgttatggtggtctataaacgtgaaccagctgtggagagggtaatagaatttgcagcaaagtttgttacctcatttcaccaatcagatatggaagatgatgaggaagaggaagatggtggccttttaaattatttgtttacttttctcttaaagtctcatgaagcaaacagcaatgcagtgggatttagagtgtgcctgctcataaacaagcttttgggaagtatgccagaaaatgctcagattgatgatgatgtgtttgataaaattaataaagccatgcttattagattgaaagataagattccaaatgtgagaatacaggcagttctggcgctttcacgacttcaggatcccaaggatgatgaatgcccagtggttaatgcatatgctactttgattgaaaatgattcaaatccagaagttagacgggcagtgttatcatgtattgcaccatcagcaaagactttgccaaaaattgtagggcgcaccaaggatgtgaaagaggctgtcagaaagctggcttatcaggttttagctgaaaaggttcatatgagagctatgtccattgctcagagagtaatgctccttcaacaaggtcttaatgacagatcagacgctgtgaaacaagctatgcagaagcatcttcttcaaggctggttacggttctctgaaggaaatatcttagagttgctccatcggttggatgtagaaaattcttctgaagtggcagtctctgttctcaatgccttgttttcaataactcctctcagtgaactggtgggactctgtaaaaacaatgatggcaggaaattgattccagtggaaacattaactcctgaaattgctttgtattggtgtgccctttgtgaatatttgaaatcaaaaggagatgaaggtgaagaatttttagagcagattttgccagagcctgtagtatatgcagactatttattgagttacatccagagcattccagttgttaatgaagaacacagaggtgatttttcctatattggaaatttgatgacaaaagaattcataggtcaacaattgattctaattattaagtctttggataccagtgaagaaggaggaagaaaaaaactgctggctgttttacaggagattcttattttacccacaatcccaatatccctggtttcttttcttgttgaaagactactccacatcattatagatgataataagagaacacaaattgttacagaaattatctcagagattcgagcgcccattgttactgttggtgttaataacgatccagctgatgtaagaaagaaagaactcaagatggctgaaataaaagttaagcttatcgaagccaaagaagctttggaaaattgcattaccttacaggattttaatcgggcatcagaattaaaagaagaaataaaagcattagaagatgccagaataaaccttttgaaagagacagagcaacttgaaattaaagaagtccacatagagaagaatgatgctgaaacattgcagaaatgtcttattttatgctatgaactgttgaagcagatgtccatttcaacaggcttaagtgcaaccatgaatggaatcatcgaatctttgattcttcctggaataataagtattcatcctgttgtaagaaacctggctgttttatgcttgggatgctgtggactacagaatcaggattttgcaaggaaacacttcgtattactattgcaggttttgcaaattgatgatgtcacaataaaaataagtgctttaaaggcaatctttgaccaactgatgacgttcgggattgaaccatttaaaactaaaaaaatcaaaacacttcattgtgaaggtacagaaataaacagtgatgttgagcaagaatcaagagaagttgaagagactgctacagctaagaatgttctgaaactcctttctgatttcttagatagtgaggcatctgaacttaggactggagctgcagaaggactagccaagctgatgttctctgggcttttggtcagcagcaggattctttctcgtcttattttgttatggtacaatcctgtgactgaagaggatgttcaacttcgacattgcctaggcgtgttcttccccgtgtttgcttatgcaagcaggactaatcaggaatgctttgaagaagcttttcttccaaccctgcaaacactggccaatgcccctgcatcttctcctttagctgaaattgatatcacaaatgttgctgagttacttgtagatttgacaagaccaagtggattaaatcctcaggccaagacttcccaagattatcaggccttaacagtacatgacaatttggctatgaaaatttgcaatgagatcttaacaagtccgtgctcgccagaaattcgagtctatacaaaagccttgagttctttagaactcagtagccatcttgcaaaagatcttctggttctattgaatgagattctggagcaagtaaaagataggacatgtctgagagctttggagaaaatcaagattcagttagaaaaaggaaataaagaatttggtgaccaagctgaagcagcacaggatgccaccttgactacaactactttccaaaatgaagatgaaaagaataaagaagtatatatgactccactcaggggtgtaaaagcaacccaagcatcaaagtctactcagctaaagactaacagaggacagagaaaagtgacagtttcagctaggacgaacaggaggtgtcagactgctgaagccgactctgaaagtgatcatgaagttccagaaccagaatcagaaatgaagatgagactaccaagacgagccaaaaccgcagcactagaaaaaagtaaacttaaccttgcccaatttctcaatgaagatctaagttaggaaagacgatggaggtggaatcctttaagattatgtccagttatttgctttaataaagaagaagttacccttgtcaaaatcag

>PDLIM5_probe1

CTTGCTTTGTATGCTCAGTGTGTTG

>PDLIM5_probe2

GCCCTCTTTGGTACTATATGCCATG

>PDLIM5_probe3

ACACCTGGCATGACACTTGCTTTGT

>PDLIM5_probe4

GAATTTCCCATAGAAGCTGGTGACA

>PDLIM5_probe5

GAAGCTGGTGACATGTTCCTGGAAG

>PDLIM5_probe6

CAAGAAGGACAAGCCCCTGTGTAAG

>PDLIM5_probe7

TGACATGTTCCTGGAAGCTCTGGGC

>PDLIM5_probe8

ATATGCCATGGATGTGAATTTCCCA

>PDLIM5_probe9

GCCCCTGTGTAAGAAACATGCTCAT

>PDLIM5_probe10

GGAAGGTCAGACCTTTTTCTCCAAG

>PDLIM5_probe11

TTATTATGCCCTCTTTGGTACTATA

>PDLIM5_target

ttattatgccctctttggtactatatgccatggatgtgaatttcccatagaagctggtgacatgttcctggaagctctgggctacacctggcatgacacttgctttgtatgctcagtgtgttgtgaaagtttggaaggtcagacctttttctccaagaaggacaagcccctgtgtaagaaacatgctcat

>PDLIM5_consensus

tttaattactaaatattacttataggaaaggaaaaacattattgaatatttatttaaatgtcacatctaatcaacttggtctgtttttgttctttttcttcttttaacctccaaaccatttcttaacccagtttcctctatctcaataaataaccccatcgtccactatccaaaaacctgcaggccatgttggattctgcttttccccttgttctttccttatccaatctaaataggaaattctggcagctctacctccaacgtatatcatattcatttatttctctccatctccactaaagctaactaaagctaaagtcactgaagctaacttgctttcaccctttttctggacattgtaattggcacctaaatggtatagcccctcaccttccttccaacctccaaacagcaggcatagaagctgtagtatatatgtgtgtatgtgtgtgtgtgtgtgtgtgtgtgtgtgtgtgtgtgtgtgtgtattgtttagacggaatcttgctctgtcgcccaggctggagtgcagtggcgcaatcttggctcactgcaagctctgcctcccgggttcatgccattctcctgcctcagcctcctgagtagctgggactacaggcacccgtcaccacgcctggctaatttttttgtatttttagtatagacggggtttaccatgttagccaggatggtctcgatctcctgacctcgtgatccgtccacctcagcctcccaaagtgctgggattacaagcgtgagccaccacgcctgaccagtaatagtttttgaaatgtgaattagatcaagcttaaaccctttagtgggttccacctagaaaaaaattacactttttttttttttgagatggagtctcactctgtcacccaggctgcagtgcagtggcccgatctcggctcaccgtaacctccacctcccgggttcaggcgattcttctgcctcacctctcaagtggctgggattacaggcacctgtcaccactcccggctaatatttttgtatttttagtggagacggggtttcaccatgttggccagggtagtgtagaactcctgacctcaagtgatccacctgcctcggcctcccaaagtgctgggattacaggtgtgagccaccgcgcccggccaaattacacttttgaaggcctgttttgccatccttatctcctctcaccttaccttctgtcaccagattctacccataagcagccctttggcatttttctgaacatgctgagatccttccttctttggggcttttgtgcttactgtttcttctaacttgaatgttctttgtcagtttttctgcggcgattcctctatattcacgtttcattgcaactattacctcttcagagaggccttttctaattttccttaatagtgtagtgccccccccaattattttgtattaaattactattttctttccttcatggtacttatcataatctgaaatttatttattatcatttattttttaaattcaataaatcatgacatcctctttccaatgagaatatgagccccatgaggaaagcaacctcaactgtcttcttcaccactgtacccctgcagtgccttgaacaatgcctgacacacagtagactgcatgaatctatgctgagcaaatggtgctaaggaatactaaagattgacaattgccaaattattatgccctctttggtactatatgccatggatgtgaatttcccatagaagctggtgacatgttcctggaagctctgggctacacctggcatgacacttgctttgtatgctcagtgtgttgtgaaagtttggaaggtcagacctttttctccaagaaggacaagcccctgtgtaagaaacatgctcattctgtgaatttttgaaagtcaacagttcaggagaagagaaggaatttgaagagaaaaaggaaaattaaaattactaattaatttttagattcaatatttatatggagttttgaaaaataatagtggccctgaaggaataaattccagctttaaaaaccaaaaaaaaaaaaaaaaaaaaaaaaaaaaaaaaaaaaaaaaaaaaaaaaaaaaaa

>SOX9_probe1

GAGAGGACCAACCAGAATTCCCTTT

>SOX9_probe2

AAGCATGTGTCATCCATATTTCTCT

>SOX9_probe3

CTACCTGGAGGGGATCAGCCCACTG

>SOX9_probe4

AGTTGAACAGTGTGCCCTAGCTTTT

>SOX9_probe5

GGAGAATCGTGTGATCAGTGTGCTA

>SOX9_probe6

GTAGTGTATCACTGAGTCATTTGCA

>SOX9_probe7

TGGGCTGCCTTATATTGTGTGTGTG

>SOX9_probe8

TGTTTTCTGCCACAGACCTTTGGGC

>SOX9_probe9

TGTTCTCTCCGTGAAACTTACCTTT

>SOX9_probe10

AAATGCTCTTATTTTTCCAACAGCT

>SOX9_probe11

CCTAGCTTTTCTTGCAACCAGAGTA

>SOX9_probe12

GAATTCCCTTTGGACATTTGTGTTT

>SOX9_probe13

GCCAACCTTGGCTAAATGGAGCAGC

>SOX9_probe14

ATTACTGCTGTGGCTAGAGAGTTTG

>SOX9_probe15

TTGGAGTGAGGGAGGCTACCTGGAG

>SOX9_probe16

ATATGGCATCCTTCAATTTCTGTAT

>SOX9_probe17

CAGCCCACTGACAGACCTTAATCTT

>SOX9_probe18

ATCAGTGGCCAGGCCAACCTTGGCT

>SOX9_probe19

TTTTCCAACAGCTAAACTACTCTTA

>SOX9_probe20

GCAACTCGTACCCAAATTTCCAAGA

>SOX9_probe21

ACATGACCTATCCAAGCGCATTACC

>SOX9_probe22

GTAAAAGCTTTGGTTTGTGTTCGTG

>PBX2_probe1

TAGTTCTCTCCTCACTTGTAAACTT

>PBX2_probe2

GTATATGTATCTTCCTCAATTTCCC

>PBX2_probe3

GGAGGCAGTGAAGGGCTTGCCCTGC

>PBX2_probe4

CATCTTCCCCTGTGAGTGACATGTC

>PBX2_probe5

AGGTTGGAAGTGTGATGGGTGGGGG

>PBX2_probe6

GGTATCTTTTTGTCACACCAAAATC

>PBX2_probe7

CCCCTCCCATTAAAGATCCGGGCAG

>PBX2_probe8

AAAGTAACATCAACACTGTCCCATC

>PBX2_probe9

GATCCCCTCAGACATTCTCAGGATT

>PBX2_probe10

GACTGTCAGAGTGGGGAACCCCTCC

>PBX2_probe11

GGGTTGGGGTGCTTGTATATGTATC

>PBX2_target

ggaggcagtgaagggcttgccctgctggcctctcatcccccttcttcccacaacccttgggcagggctggactcagtaattttgaggaaattgaagatgccatcttcccctgtgagtgacatgtctttaattttttaaaaaactactatttgaaaattggagggggaagaatgggaagggagttattgccaaatatgttaaatatgggttggggtgcttgtatatgtatcttcctcaatttccccataaatgaggtatctttttgtcacaccaaaatcaaggggtagggagagggaggaggttgcaaaaagccagatgtggggaaaagtaacatcaacactgtcccatcctcagccctgaactagctaccatctgatcccctcagacattctcaggattttacaagactgtcagagtggggaacccctcccattaaagatccgggcaggactgggacaggttggaagtgtgatgggtgggggggtgggaggcatgggccggggggctagttctctcctcacttgtaaactt

>PBX2_exemplar

atggacgaacggctactggggccgccccctccaggcgggggccgggggggcctgggattggtgagtggggagcctgggggccctggcgagcctcccggtggcggagaccccggtgggggtagcgggggggtcccgggaggccgagggaagcaagacatcggggacattctgcagcagataatgaccatcaccgaccagagcctggacgaggcccaggccaagaaacacgccctaaactgccaccgaatgaagcctgctctctttagcgtcctgtgtgaaatcaaggagaaaactggcctcagcattcggagctcccaggaggaggagccggtggacccacagctgatgcgcttggacaacatgcttctggcagagggtgtggctgggcccgagaaagggggcggctcagcagcagcagctgcagccgctgcagcctctggtggtggtgtgtcccctgacaactccatcgaacactcggactatcgcagcaaacttgcccagatccgtcacatataccactcggagctggagaagtatgagcaggcatgtaatgagttcacgacccatgtcatgaacctgctgagggagcagagccgcaccaggcccgtggcccccaaagagatggaacgcatggtgagcatcatccatcgaaagttcagcgccatccagatgcagctgaagcagagcacctgcgaggctgtgatgatcctgcgctcccgtttcctggatgccagacgaaagcgccgtaacttcagcaaacaggccactgaggtcctaaatgagtatttctactcccacctgagtaacccatatcctagtgaggaggccaaggaggagcttgccaagaagtgtggcatcaccgtgtctcaggtctccaactggtttggcaacaagaggattcgctataagaaaaacatcggaaagttccaagaggaggcaaacatctatgctgtcaagaccgccgtgtcagtcacccaggggggccacagccgcaccagctccccgacacccccttcctctgcaggctctggcggctctttcaatctctcaggatctggagacatgtttctggggatgcctgggctcaacggagattcctattctgcttcccaggtggaatcactccgacactcgatggggccagggggctatggggataacctcgggggaggccagatctacagcccacgggaaatgagggcaaatggcagctggcaagaggctgtgaccccctcttcagtgacatccccaacggagggaccagggagtgttcactctgatacctccaactgatcttgcccctcagggtcacaggggtgggggctctcacaaggcgacttgaagaggacgcaggcttccagaggacaaaccccaatacaggagaagcacaagacagagaagggccaatggggtcatcccctccctaacgagactctctgtgctgggggtgctaattacatggcaggaagaatggggcctctaaggggagtgtggggtctgtctctcccttttttccatctttttcctctctcgctttctttcttacacagaaacatacacataccgagaaacctatttctcagacccctttttctcctctgtctttctctctccctctcccacacctcacacacacatactcccacttgcaactattctgtttctctcctgggctcccccactttcccttccccaccccacttgtatgctctggaatctgtggagacgccagccctgcccaatcagagatgccaaaaatggggacatgacttctggacagaggacatgggccacgcccccatgcatccccacccccgcccctccggacggcttacttacctcatacgcagctcatcttaaaccaatagaatcgctcggtggacgagagtgtctgactcagatatctacctcggagggagtttctgctactttagggaattattgactgggctttggggttgaacttttttttttttaaagaaagaaaaagaaaccctgggatccatctgtttttttttttgttgttgttgtttttgttgttgttggtggtggtggtggtggtggttcttaatttttaatttagtttggggaagtagcttgtttttttttttataaatatgttgatttcttgtcttttttttttatttcttactttcccatattaggggtgatagccaaaggggttctggtaagagaaagggggacaaacagaactggtaaagaggcccccctggctccaggcctgtccatcaggaaggtaaattttacagggcaccaagctttgccccctaaaatcccttaggtgttctttgttcatgcaggcaggtttctgccgcatttgatgtggaggcagtgaagggcttgccctgctggcctctcatcccccttcttcccacaacccttgggcagggctggactcagtaattttgaggaaattgaagatgccatcttcccctgtgagtgacatgtctttaattttttaaaaaactactatttgaaaattggagggggaagaatgggaagggagttattgccaaatatgttaaatatgggttggggtgcttgtatatgtatcttcctcaatttccccataaatgaggtatctttttgtcacaccaaaatcaaggggtagggagagggaggaggttgcaaaaagccagatgtggggaaaagtaacatcaacactgtcccatcctcagccctgaactagctaccatctgatcccctcagacattctcaggattttacaagactgtcagagtggggaacccctcccattaaagatccgggcaggactgggacaggttggaagtgtgatgggtgggggggtgggaggcatgggccggggggctagttctctcctcacttgtaaacttgtgtagtttcacagaaaaaaaacaaaatgcagttttaaataaagaaatttcttttt

>PLIN2_probe1

TATGTTCTCATTCTATGGCCATTGT

>PLIN2_probe2

GAGTCTCAGAATGCTCAGGACCAAG

>PLIN2_probe3

TGTGGCCAGACAGATGACACCTTTT

>PLIN2_probe4

GTCTGCTCTGGTGTGATCTGAAAAG

>PLIN2_probe5

GCTTTATCTCATGATGCTTGCTTGT

>PLIN2_probe6

GGGGTAGAAACTGGTGTCTGCTCTG

>PLIN2_probe7

CAGGAGACCCAGCGATCTGAGCATA

>PLIN2_probe8

GAAAAGGCGTCTTCACTGCTTTATC

>PLIN2_probe9

TATGGCCATTGTGTTGCCTCTGTTA

>PLIN2_probe10

ATCACTAGTGCATGCTGTGGCCAGA

>PLIN2_probe11

AACATCTTCATGTGGGCTGGGGTAG

>PLIN2_target

gagtctcagaatgctcaggaccaaggtgcagagatggacaagagcagccaggagacccagcgatctgagcataaaactcattaaacctgcccctatcactagtgcatgctgtggccagacagatgacaccttttgttatgttgaaattaacttgctaggcaaccctaaattgggaagcaagtagctagtataaaggccctcaattgtagttgtttccagctgaattaagagctttaaagtttctggcattagcagatgatttctgttcacctggtaagaaaagaatgataggcttgtcagagcctatagccagaactcagaaaaaattcaaatgcacttatgttctcattctatggccattgtgttgcctctgttactgtttgtattgaataaaaacatcttcatgtgggctggggtagaaactggtgtctgctctggtgtgatctgaaaaggcgtcttcactgctttatctcatgatgcttgcttgt

>PLIN2_exemplar

ggcacgagggccggagtcgtcttcgggacgcgcctgctcttcgcctttcgctgcagtccgtcgatttctttctccaggaagaaaaatggcatccgttgcagttgatccacaaccgagtgtggtgactcgggtggtcaacctgcccttggtgagctccacgtatgacctcatgtcctcagcctatctcagtacaaaggaccagtatccctacctgaagtctgtgtgtgagatggcagagaacggtgtgaagaccatcacctccgtggccatgaccagtgctctgcccatcatccagaagctagagccgcaaattgcagttgccaatacctatgcctgtaaggggctagacaggattgaggagagactgcctattctgaatcagccatcaactcagattgttgccaatgccaaaggcgctgtgactggggcaaaagatgctgtgacgactactgtgactggggccaaggattctgtggccagcacgatcacaggggtgatggacaagaccaaaggggcagtgactggcagtgtggagaagaccaagtctgtggtcagtggcagcattaacacagtcttggggagtcggatgatgcagctcgtgagcagtggcgtagaaaatgcactcaccaaatcagagctgttggtagaacagtacctccctctcactgaggaagaactagaaaaagaagcaaaaaaagttgaaggatttgatctggttcagaagccaagttattatgttagactgggatccctgtctaccaagcttcactcccgtgcctaccagcaggctctcagcagggttaaagaagctaagcaaaaaagccaacagaccatttctcagctccattctactgttcacctgattgaatttgccaggaagaatgtgtatagtgccaatcagaaaattcaggatgctcaggataagctctacctctcatgggtagagtggaaaaggagcattggatatgatgatactgatgagtcccactgtgctgagcacattgagtcacgtactcttgcaattgcccgcaacctgactcagcagctccagaccacgtgccacaccctcctgtccaacatccaaggtgtaccacagaacatccaagatcaagccaagcacatgggggtgatggcaggcgacatctactcagtgttccgcaatgctgcctcctttaaagaagtgtctgacagcctcctcacttctagcaaggggcagctgcagaaaatgaaggaatctttagatgacgtgatggattatcttgttaacaacacgcccctcaactggctggtaggtcccttttatcctcagctgactgagtctcagaatgctcaggaccaaggtgcagagatggacaagagcagccaggagacccagcgatctgagcataaaactcattaaacctgcccctatcactagtgcatgctgtggccagacagatgacaccttttgttatgttgaaattaacttgctaggcaaccctaaattgggaagcaagtagctagtataaaggccctcaattgtagttgtttccagctgaattaagagctttaaagtttctggcattagcagatgatttctgttcacctggtaagaaaagaatgataggcttgtcagagcctatagccagaactcagaaaaaattcaaatgcacttatgttctcattctatggccattgtgttgcctctgttactgtttgtattgaataaaaacatcttcatgtgggctggggtagaaactggtgtctgctctggtgtgatctgaaaaggcgtcttcactgctttatctcatgatgcttgcttgtaaaacttgattttagtttttcatttctcaaataggaatactacctttgaattcaataaaattcactgcaggataaataaaaaaaaaaaaaaaaaaaaaaaaaaaaaaaaaaaa

>LMO4_probe1

CCCTTCCCGCATTTATTGGTGTATT

>LMO4_probe2

ACCTTTGTAGCTAGCACCAGTGCCA

>LMO4_probe3

TTCATCTCAGATTTGTTCATCACAG

>LMO4_probe4

GTCTTCAGTAGACAAGTCACCTTTG

>LMO4_probe5

TTAAGGACTCCATGAACCTGGGCTA

>LMO4_probe6

TAATGTTGCTACTCCCATGGCAAAG

>LMO4_probe7

GTTTTTGTCCTAATGTTGCTACTCC

>LMO4_probe8

CAGAGGACATCTTGGGGAGGGGGAG

>LMO4_probe9

CACCTTCTTTAGTCTTGATTGCCCT

>LMO4_probe10

CCATTGCACCTTCTTTAGTCTTGAT

>LMO4_probe11

GATGTGGCTTTTGTGATATTCTATC

>LMO4_target

ttcatctcagatttgttcatcacaggtggatcccatgtgtcttcagtagacaagtcacctttgtagctagcaccagtgccagctccatgccattgcaccttctttagtcttgattgcccttcccgcatttattggtgtattaaaatgactgaatatgaacattaaggactccatgaacctgggctaatgggagactgtagagaaaatgaaaaaagatccaccagaggacatcttggggagggggagggagctgggggggagggaaatgactaatgaagctaattaaaagaagcattcaaatctgctttctaccctcattaacaattagcagggcactggccagagtttgtaccctgtgttttaccttaacaacattctatttgctctttgtatatttaagtgttgtaaggaaacgtgtttcaatcaaaactgaccatgagataaaggaaagagatgtggcttttgtgatattctatcacaaacacttattgtatctctgtaaaatacaatgtatgtatgcatgtaagtgtttttgtcctaatgttgctactcccatggcaaag

>LMO4_exemplar

cccctttcctgcttctgcgagaactccctccctccctccagctccgccagcccaggcgccccttccctggaagccgagcggcttcgctcgcatttcaccgccgccgcctctcgcaatattgcaatataggggaaaagcagaccatggtgaatccgggcagcagctcgcagccgcccccggtgacggccggctccctctcctggaagcggtgcgcaggctgcgggggcaagattgcggaccgctttctgctctatgccatggacagctattggcacagccggtgcctcaagtgctcctgctgccaggcgcagctgggcgacatcggcacgtcctgttacaccaaaagtggcatgatcctttgcagaaatgactacattaggttatttggaaatagcggtgcttgcagcgcttgcggacagtcgattcctgcgagtgaactcgtcatgagggcgcaaggcaatgtgtatcatcttaagtgttttacatgctctacctgccggaatcgcctggtcccgggagatcggtttcactacatcaatggcagtttattttgtgaacatgatagacctacagctctcatcaatggccatttgaattcacttcagagcaatccactactgccagaccagaaggtctgctaaaaggtcagagtaatgcagaatgcgtgccttcatctcagatttgttcatcacaggtggatcccatgtgtcttcagtagacaagtcacctttgtagctagcaccagtgccagctccatgccattgcaccttctttagtcttgattgcccttcccgcatttattggtgtattaaaatgactgaatatgaacattaaggactccatgaacctgggctaatgggagactgtagagaaaatgaaaaaagatccaccagaggacatcttggggagggggagggagctgggggggagggaaatgactaatgaagctaattaaaagaagcattcaaatctgctttctaccctcattaacaattagcagggcactggccagagtttgtaccctgtgttttaccttaacaacattctatttgctctttgtatatttaagtgttgtaaggaaacgtgtttcaatcaaaactgaccatgagataaaggaaagagatgtggcttttgtgatattctatcacaaacacttattgtatctctgtaaaatacaatgtatgtatgcatgtaagtgtttttgtcctaatgttgctactcccatggcaaagaaaaaaaaaagaatgaaaaaaaaaaaaaaaaaa

>PIK3R1_probe1

CACGGTCAGTTGTAACTTTGCCTTC

>PIK3R1_probe2

GACTATCCAACTTAACATGAAACTT

>PIK3R1_probe3

GAGATAGCATTAGCTGCCCAGGATG

>PIK3R1_probe4

AATGGAGCTATGTCTTGTTTTAAGT

>PIK3R1_probe5

GAGAGGGAGGATGTCACGGTCAGTT

>PIK3R1_probe6

AGTTGGTCTTTTGACGAGAGGGAGG

>PIK3R1_probe7

GTGCCTCCTTGACATTTCGTTCAAG

>PIK3R1_probe8

GAAACTTGTCACCATGAGATAGCAT

>PIK3R1_probe9

AAAGCTACAATCTGTTCAATGTTTT

>PIK3R1_probe10

CTGCCCAGGATGCTGCTATATATAT

>PIK3R1_probe11

AAAAACTCATTTATACCTGTGTATT

>PIK3R1_target

gactatccaacttaacatgaaacttgtcaccatgagatagcattagctgcccaggatgctgctatatatatatatatatatatatannnntntgtgtgtgtgtntntntntntntatatatatatnnnnnnnnnnnnnnnnnnnatatatatntntgtgtgtnnnnatatatatatatatgtgtatatatatatgtatatacatatatgtatatatangcacatatatatatgtatttaaaaaaatcaaaacaaaaaaaaactcatttatacctgtgtattttttaaagctacaatctgttcaatgtttttaaaaatctgtttatatgacattgttaaaataaagttggtcttttgacgagagggaggatgtcacggtcagttgtaactttgccttcacaaggcaactggggtggggggtgggggtagtgtgcctccttgacatttcgttcaagttatagattcaatggagctatgtcttgttttaagt

>PIK3R1_consensus

gggtgaagctcgtgtgtggagtgccncggtacaatcngacgacagatggacagtgtgacaaaagtgtcagaaaggattgggcctcgctgtgagagtcagcctggattcaaagtgttgacaagttgctgaaaaggaagccagtgagaggactgtggcacgcagaggaagtggagccctgtcttcggtcacaccattgatggaggacagatggacagccgtatggccagtcacctctcctcttaaacctttggagagtggtcctttgtcctctgctggacacataataggaattctaacacattctctgaattcacttttcataaaaacgtaaaatcagactgctctgtacaaccaggctcaactgttgcatggtagcagatttgcaaacatgagtgctgaggggtaccagtacagagcgctgtatgattataaaaannaaanagaagaagatattgacttgcacttgggtgacatattgactgtgaataaagggtccttagtagctcttggattcagtgatggacaggaagccaggcctgaagaaattggctggttaaatggctataatgaaaccacaggggaaaggggggactttccgggaacttacgtagaatatattggaaggaaaaaaatctcgcctcccacaccaaagccccggccacctcggcctcttcctgttgcaccaggttcttcgaaaactgaagcagatgttgaacaacaagctttgactctcccggatcttgcagagcagtttgcccctcctgacattgccccgcctcttcttatcaagctcgtggaagccattgaaaagaaaggtctggaatgttcaactctatacagaacacagagctccagcaacctggcagaattacgacagcttcttgattgtgatacaccctccgtggacttggaaatgatcgatgtgcacgttttggctgacgctttcaaacgctatctcctggacttaccaaatcctgtcattccagcagccgtttacagtgaaatgatttctttagctccagaagtacaaagctccgaagaatatattcagctattgaagaagcttattaggtcgcctagcatacctcatcagtattggcttacgcttcagtatttgttanaacatttcttcaagctctctcaaacctccagcaaaantctgttgaatgcaagagtactctctgaaattttcagccctatgcttttcagattctcagcagccagctctgataatactgaaaacctnataaaagntnnnnnannnnnantnnnaanngaannnnntgaannncannnnnnncnancactgcctcctaaaccaccaaaacctactactgtagccaacaacggtatgaataacaatatgtccttacaanatgctgaatggtactggggagatatctcgagggaagaagtgaatgaaaaacttcgagatacagcagacgggacctttttggtacgagatgcgtctactaaaatgcatggtgattatactcttacactaaggaaagggggaaataacaaattaatcaaaatatttcatcgagatgggaaatatggcttctctgacccattaaccttcagttctgtggttgaattaataaaccactaccggaatgaatctctagctcagtataatcccaaattggatgtgaaattactttatccagtatccaaataccaacaggntcaagttgtcaaagaagntaatattgaagctgtagggaaaaaattacatgaatataacactcagntttcaagaaaaaagtcgagaatatgatagattatatgaagaatatacccgcacatcccaggaaatccaaatgaaaaggacagctattgaagcatttaatgaaaccataaaaatatttgaagaacagtgccagacccaagagcggtacagcaaagaatacatagaaaagtttaaacgtgaaggcaatgagaaagaaatacaaaggattatgcataattatgataagttgaagtctcgaatcagtgaaattattgacagtagaagaagattggaagaagacttgaagaagcaggcagctgagtatcgagaaattgacaaacgtatgaacagcattaaaccagaccttatccagctgagaaagacgagagaccaatacttgatgtggttgactcaaaaaggtgttcggcaaaagaagttgaacgagtggttgggcaatgaaaacactgaagaccaatattcactggtggaagatgatgaagatttgccccatcatgatgagaagacatggaatgttggaagcagcaaccgaaacaaagctgaaaacctgttgcgagggaagcgagatggcacttttcttgtccgggagagcagtaaacagggctgctatgcctgctctgtagtggtggacggcgaagtaaagcattgtgtcataaacaaaacagcaactggctatggctttgccgagccctataacttgtacagctctctgaaagaactggtgctacattaccaacacacctcccttgtgcagcacaacgactccctcaatgtcacactagcctacccagtatatgcacagcagaggcgatgaagcgcttactctttgatccttctcctgaagttcagccaccctgaggcctctggaaagcaaagggctcctctccagtctgatctgtgaattgagctgcagaaacgaagccatctttctttggatgggactagagctttctttcacaaaaaagaagtaggggaagacatgcagcctaaggctgtatgatgaccacacgttcctaagctggagtgcttatcccttctttttctttttttctttggtttaatttaaagccacaaccacatacaacacaaagagaaaaagaaatgcaaaaatctctgcgtgcagggacaaagaggcctttaaccatggtgcttgttaatgctttctgaagctttaccagctgaaagttgggactctggagagcggaggagagagaggcagaagaaccctggcctgagaaggtttggtccagcctggtttagcctggatgttgctgtgcacggtggacccagacacatcgcactgtggattatttcattttgtaacaaatgaacgatatgtagcagaaaggcacgtccactcacaagggacgctttgggagaatgtcagttcatgtatgttcagaagaaattctgtcatagaaagtgccagaaagtgtttaacttgtcaaaaaacaaaaacccagcaacagaaaaatggagtttggaaaacaggacttaaaatgacattcagtatataaaatatgtacataatattggatgactaactatcaaatagatggatttgtatcaataccaaatagcttctgttttgttttgctgaaggctaaattcacagcgctatgcaattcttaattttcattaagttgttatttcagttttaaatgtaccttcagaataagcttccccaccccagtttttgttgcttgaaaatattgttgtcccggatttttgttaatattcatttttgttatccttttttaaaagtaaatgtacaggatgccagtaaaaaaaaaaaantggcttcagaattaaaactatgaaatattttacagtttttcttgtacagagtacttggctgttagcccaaggttaaaaagttcataacagattttttttggactgttttgttgggcagtgcctgataagcttcaaagctgctttattcaataaaaaaaagaaatgaaaaagatatatgaatatgacaaagtannnntgagtccaacaatgttgttttaagactcttaaaatacggtacctggcaatgtttatttcataaagaattgtgaacttcttgaatctagggagggggaatgtagtgaagggatgtatcaagtggggtggtgggagggggaggcaaggttatatgcactttctcatgatttacagagaagtgaataactgcaaagtgaagttgcttcttctacttcagtcttctctcactttgatttgctagttgttatcaattaatgacaattacaaacctactgtatctctaatacagtgtgactggtcaggtatttcagttcttaggaaggaagtgccaagtttgtttttgggttcctggaacagcgctcacctttgtttagaacactggtttaaagggataatcatctctgtcacattagactatccatcatgaccagcaaatactcattttaggaaaaaaaaaagcatgatctgaaaaatacttttggtggtatgttggttaccctcctagctttccatttggtttagaacataaagcaaatagacacagtcatactgtcactgctctggactgtgtggagctcgctaaagtcatggtcattgcaggaatccaagtggcagtccttctcattcattctaatcattgtatgtgcttcactacgggggggagaaggaaancgttagcatncantgtttcccatttagggcaggagtgagaggtctctcttcctgatttagatatgcaaaagctggtatgttcagtaggaactgtacatgtgttgggaggcataaagactaattagcaaccataatatggtcactaccctaatagactaaatgaaatcttgcaatttcaaattactctttctccatattagatttacccacagctatatttctgtttaagtactagggtgagggttttctgttactttgttttttaatgttgttccttttgaaagaatcagtcttgcagctgagtgaaaaatctgtggaatgtattatttgtcctctttacatgaaactactcatacttaagcaaaagtcagtcttatagcaagactgttagccctcaaacttgactctactgatctgaccatttccctctcatcgccagacaactgacgntttcccnggnntttnngtctgnnngtctctgnnntttaaagntnattgtgnatatccnttctagnnnncanacnnncangnctaacngnntnaattagnnttaacngnnnttttnaaacnnggnntttgtgggnnnttttntnnnnncncatgtatgntattacatacaaatttttatttctaaaatataagatctgagattgaatattttcattaaaagctacagttttgtgaatctttgtgcntcaacattctttgcaagatgatacggtatttaggcatttgcnttatttttgcatctcacaaacataagtgcaatagatcttttcattgaacagcaaagtaggattcatcattccatatgacttgagttacaccagacctattctgcccaatgcctttttgattacagtgtagcttgcccaccgcatttgtcgttttagatactttgctagccggccactttggnatttcatcagacagtcctaacaatattgtctgaacggctgaatatgaatagatacagcagaggcactcctgatatatgatttttatccatgcgtcagtttttcccacccagtgtagcatcctaaanngataaagccagaagctaagctgcnagtgaggctgtgnattgggcgtagaagtgggnagcattgggnacctcacattacacacacgagagatcataaccatgtgaaaaggncaaaaagcatgtgtttgcaacatctgataacttcatggcctttgataaatgtatatatgtatatgtgcatggactgtgtttccagtacacctttcagccaaaacagatccacagtagttgttgagttcaagtacataaagtacataacaagcgaacgtctagtacaattcttacttatgtgtatgggattttnccctttgaggttgctttgttttgtcttacaaaggtgaaaattgtttgtaagtgaagtgagaagttcatatttctttggcttttttgtgtttttaaaagttactccttttagggagctggtctgatgacttgcttagcttggaaatccttgttttcagtgtgtcgagtcaaaatgtgtttatgtgagctgtcactgtgggganccaattngctttgtcatatagctggttatgaactagtaacatgtttgggaagtcctactgatgttcctttggaagaaaaaatctgctggttttaacaactgtgcttttgctatgtatggtatccaagttagttgaaacgcagacactgagatctgtttgagtttagggtcatttttagaaaggggcagtttaaagcacaatgtctcacatgggacaaagttccaaaatgccaaattcttattttttaaaaagctagttctataaaatactggtattatgggtggggaggaaatagaattgagtcaattggaaagactatccaacttaacatgaaacttgtcaccatgagatagcattagctgcccaggatgctgctatatatatatatatatatatatannnntntgtgtgtgtgtntntntntntntatatatatatnnnnnnnnnnnnnnnnnnnatatatatntntgtgtgtnnnnatatatatatatatgtgtatatatatatgtatatacatatatgtatatatangcacatatatatatgtatttaaaaaaatcaaaacaaaaaaaaactcatttatacctgtgtattttttaaagctacaatctgttcaatgtttttaaaaatctgtttatatgacattgttaaaataaagttggtcttttgacgagagggaggatgtcacggtcagttgtaactttgccttcacaaggcaactggggtggggggtgggggtagtgtgcctccttgacatttcgttcaagttatagattcaatggagctatgtcttgttttaagttgctttaatgcattgtattagatcttcaaacagaataaaggttgttttgaaactgaaaaaaaaaaaaaaaaaaaaaaaaaaaaaaaaaaa

>DHX9_probe1

TGACCGAGCAGCAGAGTGTAACATC

>DHX9_probe2

GTAACATCGTAGTAACTCAGCCCAG

>DHX9_probe3

ATCAGTGCGGTTTCTGTGGCAGAGC

>DHX9_probe4

GACTTTATCCAGAATGACCGAGCAG

>DHX9_probe5

TAGAGGGGCTACTGGATGTGGGAAA

>DHX9_probe6

CTCAGCCCAGAAGAATCAGTGCGGT

>DHX9_probe7

GGCTTATCCTGAAGTTCGCATTGTT

>DHX9_probe8

TGGGAAAACCACACAGGTTCCCCAG

>DHX9_probe9

TGTACTGTAGGTGTGCTCCTGAGAA

>DHX9_probe10

GCGTGATGTTGTTCAGGCTTATCCT

>DHX9_probe11

GGAGGACTTACCCAGTTCAAGAATA

>DHX9_target

tagaggggctactggatgtgggaaaaccacacaggttccccagttcattctagatgactttatccagaatgaccgagcagcagagtgtaacatcgtagtaactcagcccagaagaatcagtgcggtttctgtggcagagcgagttgcatttgaaagaggagaagagcctggaaaaagctgtggctacagcgttcgatttgagtctatacttcctcntcctcatgccagtataatgttttgtactgtaggtgtgctcctgagaaaattagaagcaggcattcgaggaatcagtcatgtaattgtagatgaaatacatgaaagagatattaatactgacttccttttggtagtactgcgtgatgttgttcaggcttatcctgaagttcgcattgttcttatgtctgctactattgataccagcatgttttgtgaatatttcttcaattgccccatcattgaagtttatgggaggacttacccagttcaagaata

>DHX9_consensus

agcgccgcggtcggagccatttcgcngatncctccatgncgagttgctgtgcgtttctctgttgtctcggtagaaggccagagtcacacacggtcctaagagctgggcaccaggaagcgaaggctgatctgaagaagacacttgaatcatgggtgacgttaaaaattttctgtatgcctggtgtggcaaaaggaagatgaccccatcctatgaaattagagcagtggggaacaaaaacaggcagaaattcatgtgtgaggttcaggtggaaggttataattacactggcatgggaaattccaccaataaaaaagatgcacaaagcaatgctgccagagactttgttaactatttggttcgaataaatgaaataaagagtgaagaagttccagcttttggggtagcatctccgcccccacttactgatactcctgacactacagcaaatgctgaaggagatttaccaacaaccatgggaggacctcttcctccacatctggctctcaaagcagaaaataattctgaggtaggggcctctggctatggtgttcctgggcccacctgggaccgaggagccaacttgaaggattactactcaagaaaggaagaacaagaagtgcaagcgactctagaatcagaagaagtggatttaaantgctngggcttcatggaaactggaccttggaaaatgctaaagctcgtctaaaccaatattttcagaaagaaaagatccanggnagaatataagtacnacccaagtgggtcctgatcacaacaggagctttattgcagaaatgaccatttatatcaagcagctgggcagaaggatttttgcacgagaacatggatcaaataagaaattggcagcacagtcctgtgccctgtcacttgtcagacaactgtaccatcttggagtggttgaagcttactccggacttacaaagaagaaggaaggagagacagtggagccttacaaagtaaacctctctcaagatttagagcatcagctgcaaaacatcattcaagagctaaatcttgagattttgcccccgcctgaagatccttctgtgccagttgcactcaacattggcaaattggctcagttcgaaccatctcagcgacaaaaccaagtgggtgtggttccttggtcacctccacaatccaactggaatccttggactagtagcaacattgatgaggggcctctggcttttgctactccagagcaaataagcatggacctcaagaatgaattgatgtaccagttggaacaggatcatgatttgcaagcaatcttgcaggagagagagttactgcctgtgaagaaatttgaaagtgagattctggaagcaatcagccaaaattcagttgtcattattagaggggctactggatgtgggaaaaccacacaggttccccagttcattctagatgactttatccagaatgaccgagcagcagagtgtaacatcgtagtaactcagcccagaagaatcagtgcggtttctgtggcagagcgagttgcatttgaaagaggagaagagcctggaaaaagctgtggctacagcgttcgatttgagtctatacttcctcntcctcatgccagtataatgttttgtactgtaggtgtgctcctgagaaaattagaagcaggcattcgaggaatcagtcatgtaattgtagatgaaatacatgaaagagatattaatactgacttccttttggtagtactgcgtgatgttgttcaggcttatcctgaagttcgcattgttcttatgtctgctactattgataccagcatgttttgtgaatatttcttcaattgccccatcattgaagtttatgggaggacttacccagttcaagaatattttctggaagactgcattcagatgacncactttgttcctccaccaaaagacaaaaagaagaaggataaggatgatgatggtggtgaggatgatgatgcaaattgcaacttgatctgtggtgatgaatatggtccagaaacaaggttgagcatgtctcaattgaacgaaaaggaaactccttttgaactcatcgaggctctacttaagtacattgaaacccttaatgttcctggagctgtgttggtttttttgcctggctggaatctgatttatactatgcagaagcatttggaaatgaatccacattttggaagccatcggtatcagattctacccctgcattctcagattcctcgagaggaacagcgcaaagtgtttgatccagtaccagttggagtaaccaaggttattttgtccacaaatattgctgaaacaagcattaccataaacgatgttgtttatgtcattgactcctgcaagcagaaagtgaaactcttcactgctcacaacaatatgaccaactatgctaccgtatgggcatcaaaaacaaaccttgagcaacggaaagggcgagctggccgagtacggnctggattctgctttcacctgtgcagccgagctcgttttgagagacttgaaacccacatgacaccagagatgttccgaacaccattgcatgaaattgctcttagcataaaacttctgcgtctaggaggaatnggccaatttctggccaaagcaattgaacctccccctttggatgctgtgattgaagcagaacacactcttagagagcttgatgcattagatgccaatgatgagttgactcctttgggacgaatcctggctaaactccccattgagcctcgtttnggcaaaatgatgataatggggtgtattttctacgtgggagatgctatctgtaccattgctgctgctacctgctttccagagcctttcatcaatgaaggaaagcggctgggctatatccatcgaaattttgctggaaacagattttctgatcacgtagcccttttatcagtattccaagcctgggatgatgctagaatgggtggagaagaagcagagatacgtttttgtgagcacaaaagacttaatatggctacactaagaatgacctgggaagccaaagttcagctcaaagagattttgattaattctgggtttccagaagattgtttgttgacacaagtgtttactaacactggaccagataataatttggatgttgttatctccctcctggcctttggtgtgtaccccaatgtatgctatcataaggaaaagaggaagattctcaccactgaagggcgtaatgcacttatccacaaatcatctgttaattgtccttttagtagccaagacatgaagtacccatctcccttctttgtatttggtgaaaagattcgaactcgagccatctctgctaaaggcatgactttagtcacccccctgcagttgcttctctttgcctccaagaaagtccaatctgatgggcagattgtgcttgtagatgactggattaaactgcaaatatctcatgaagctgctgcctgtatcactggtctccgggcagccatggaggcttnggttgttgaagtaaccaaacaacctgctatcatcagccagttggaccccgtaaatgaacgtatgctgaacatgatccgtcagatctctagaccctcagctgctggtatcaaccttatgattggcagtacacggtatggagatggtccacgtcctcccaagatggcccgatacgacaatggaagcggatatagaaggggaggttctagttacagtggtggaggctatggcggtggctatagcagtggaggctatggtagcggaggctatggtggcagcgccaactccntttcgggcaggatatggtgcaggtgtnggtggaggctatagaggagtttcccgaggntggctttagaggcaactcntggaggagactacagagggcctagtggaggctacnagaggatctgggggattccnagcgaggaggtggtaggggggcctatggaactggctactttggacagggaagaggaggtggcggctattaaaacttggttatgtcagttcctgtgtgtagacagtaaggaaaaaaaggcatgctatgtgttacgtgttttttccagtatgtttatttgccaccaaaaagtaaatgcattttcacccattctgtggttcattgtagtttaaggaaaccaagcatatagatgcattagtgattttgtttatattatgtaaaatataacgatctcttaaaaataccacagtttgtattttttctttaaggagtaaagatttgcctttaaataacttggtattttcctggctttcgtttaatacaatagaaaataaagtattacaccgaanannnnnnnnnnannnnnnnnnnnnacctcgtatgttagaaaattttacaatgccagctacatctgttgattttaaatgtcagagaagttgtaccctgtttcaaaagtatactaagtgatactacttgtaatagaataaatcatcttggaattgaattgttaccttttgaagtaaatactggcaagtgcacaagccacataaacctgaataaaacttttgacctagggttgaaaaaaaaaaaaaaaaaaaaaaaaaaaaaaaaaaaaaaaaaaaaaaaaaaaaaaaaaa

>CD44_probe1

GGATGGCTTCTAACAAAAACTACAC

>CD44_probe2

GTGTGCTATGGATGGCTTCTAACAA

>CD44_probe3

TAGTTACACATCTTCAACAGACCCC

>CD44_probe4

AGGGTGAAGCTATTTATCTGTAGTA

>CD44_probe5

TTAGGGCCCAATTAATAATCAGCAA

>CD44_probe6

CTTCCATAGCCTAATCCCTGGGCAT

>CD44_probe7

CACATATGTATTCCTGATCGCCAAC

>CD44_probe8

CAGACCCCCTCTAGAAATTTTTCAG

>CD44_probe9

TTGAATGGGTCCATTTTGCCCTTCC

>CD44_probe10

CAGGGTTAATAGGGCCTGGTCCCTG

>CD44_probe11

TTAAACCCTGGATCAGTCCTTTGAT

>CD44_target

ttagggcccaattaataatcagcaagaatttgatcgttcagttccacttggaggccttcatcctcgggtgtgctatggatggcttctaacaaaaactacacatatgtattcctgatcgccaacctttcccccaccagctaaggacatttcccagggttaatagggcctggtccctgggaggaaatttgaatgggtccattttgcccttccatagcctaatccctgggcattgctttccactgaggttggggtgtactagttacacatcttcaacagaccccctctagaaatttttcagatgcttctgggagacaccaaagggtgaagctatttatctgtagtaaactatttatctgtgtttttgaaatattaaaccctggatcagtcctttgat

>CD44_exemplar

cccgcgccctccgttcgctccggacaccatggacaagttttggtggcacgcagcctggggactctgcctcgtgccgctgagcctggcgcagatcgatttgaatataacctgccgctttgcaggtgtattccacgtggagaaaaatggtcgctacagcatctctcggacggaggccgctgacctctgcaaggctttcaatagcaccttgcccacaatggcccagatggagaaagctctgagcatcggatttgagacctgcaggtatgggttcatagaagggcatgtggtgattccccggatccaccccaactccatctgtgcagcaaacaacacaggggtgtacatcctcacatacaacacctcccagtatgacacatattgcttcaatgcttcagctccacctgaagaagattgtacatcagtcacagacctgcccaatgcctttgatggaccaattaccataactattgttaaccgtgatggcacccgctatgtccagaaaggagaatacagaacgaatcctgaagacatctaccccagcaaccctactgatgatgacgtgagcagcggctcctccagtgaaaggagcagcacttcaggaggttacatcttttacaccttttctactgtacaccccatcccagacgaagacagtccctggatcaccgacagcacagacagaatccctgctaccagagaccaagacacattccaccccagtggggggtcccataccactcatggatctgaatcagatggacactcacatgggagtcaagaaggtggagcaaacacaacctctggtcctataaggacaccccaaattccagaatggctgatcatcttggcatccctcttggccttggctttgattcttgcagtttgcattgcagtcaacagtcgaagaaggtgtgggcagaagaaaaagctagtgatcaacagtggcaatggagctgtggaggacagaaagccaagtggactcaacggagaggccagcaagtctcaggaaatggtgcatttggtgaacaaggagtcgtcagaaactccagaccagtttatgacagctgatgagacaaggaacctgcagaatgtggacatgaagattggggtgtaacacctacaccattatcttggaaagaaacaaccgttggaaacataaccattacagggagctgggacacttaacagatgcaatgtgctactgattgtttcattgcgaatcttttttagcataaaattttctactctttttgttttttgtgttttgttctttaaagtcaggtccaatttgtaaaaacagcattgctttgtaaattagggcccaattaataatcagcaagaatttgatcgttcagttccacttggaggccttcatcctcgggtgtgctatggatggcttctaacaaaaactacacatatgtattcctgatcgccaacctttcccccaccagctaaggacatttcccagggttaatagggcctggtccctgggaggaaatttgaatgggtccattttgcccttccatagcctaatccctgggcattgctttccactgaggttggggtgtactagttacacatcttcaacagaccccctctagaaatttttcagatgcttctgggagacaccaaagggtgaagctatttatctgtagtaaactatttatctgtgtttttgaaatattaaaccctggatcagtcctttgatcagtataattttttaaagttactttgtcagaggcacaaaagggtttaaactgattcataataaatatctgtacttcttcgatcttc

>RRM2_probe1

GTATTCAGTATTTGAACGTCGTCCT

>RRM2_probe2

GTCTTGCATTGTGAGGTACAGGCGG

>RRM2_probe3

TTTTACCTTGGATGCTGACTTCTAA

>RRM2_probe4

GTACAGGCGGAAGTTGGAATCAGGT

>RRM2_probe5

GACCCTTTAGTGAGCTTAGCACAGC

>RRM2_probe6

CCTGGCTGGCTGTGACTTACCATAG

>RRM2_probe7

GAACGTCGTCCTGTTTATTGTTAGT

>RRM2_probe8

CTCACAACCAGTCCTGTCTGTTTAT

>RRM2_probe9

GAAGTGTTACCAACTAGCCACACCA

>RRM2_probe10

ATGTGAGGATTAACTTCTGCCAGCT

>RRM2_probe11

CTAGCCACACCATGAATTGTCCGTA

>RRM2_probe12

CAGCCTCACTGCTTCAACGCAGATT

>RRM2_probe13

TTAGGATTCTGTCTCTCATTAGCTG

>RRM2_probe14

GTGCTGGTAGTATCACCTTTTGCCA

>RRM2_probe15

TATGGTCCTTATATGTGTACAACAT

>RRM2_probe16

GAAGATGTGCCCTTACTTGGCTGAT

>RRM2_probe17

TAAACAGTCCTTTAACCAGCACAGC

>CDK1_probe1

TGAAGTATTTTTATGCTCTGAATGT

>CDK1_probe2

CAAAGATCAAGGGCTGTCCGCAACA

>CDK1_probe3

GATGAATATTTTTCTACTGGTATTT

>CDK1_probe4

GACATAGTGTTTATTAGCAGCCATC

>CDK1_probe5

GAAAGCTTTTTGTCTAAGTGAATTC

>CDK1_probe6

GTGAATTCTTATGCCTTGGTCAGAG

>CDK1_probe7

TGTTAACTATACAACCTGGCTAAAG

>CDK1_probe8

AAATGTTCTCATCAGTTTCTTGCCA

>CDK1_probe9

TGCTAAGTTCAAGTTTCGTAATGCT

>CDK1_probe10

AAGGGCTGTCCGCAACAGGGAAGAA

>CDK1_probe11

CTTATCTTGGCTTTCGAGTCTGAGT

>CDK1_target

tgctaagttcaagtttcgtaatgctttgaagtatttttatgctctgaatgtttaaatgttctcatcagtttcttgccatgttgttaactatacaacctggctaaagatgaatatttttctactggtattttaatttttgacctaaatgtttaagcattcggaatgagaaaactatacagatttgagaaatgatgctaaatttataggagttttcagtaacttaaaaagctaacatgagagcatgccaaaatttgctaagtcttacaaagatcaagggctgtccgcaacagggaagaacagttttgaaaatttatgaactatcttatttttaggtaggttttgaaagctttttgtctaagtgaattcttatgccttggtcagagtaataactgaaggagntgcttatcttggctttcgagtctgagtttaaaactacacattttgacatagtgtttattagcagccatc

>CDK1_consensus

ggggggggggggcacttggcttcaaagctggctcttggaaattgagcggagagcgacgcggcttgttgtagctgccgctgcggccgccgcggaataataagccgggatctaccatacccattgactaactatggaagattataccaaaatagagaaaattggagaaggtacctatggagttgtgtataagggtagacacaaaactacaggtcaagtggtagccatgaaaaaaatcagactagaaagtgaagaggaaggggttcctagtactgcaattcgggaaatttctctattaaaggaacttcgtcatccaaatatagtcagtcttcaggatgtgcttatgcaggattccaggttatatctcatctttgagtttctttccatggatctgaagaaatacttggattctatccctcctggtcagtacatggattcttcacttgttaagagttatttataccaaatcctacaggggattgtgttttgtcactctagaagagttcttcacagagacttaaaacctcaaaatctcttgattgatgacaaaggaacaattaaactggctgattttggccttgccagagcttttggaatacctatcagagtatatacacatgaggtagtaacactctggtacagatctccagaagtattgctggggtcagctcgttactcaactccagttgacatttggagtataggcaccatatttgctgaactagcaactaagaaaccacttttccatggggattcagaaattgatcaactcttcaggattttcagagctttgggcactcccaataatgaagtgtggccagaagtggaatctttacaggactataagaatacatttcccaaatggaaaccaggaagcctagcatcccatgtcaaaaacttggatgaaaatggcttggatttgctctcgaaaatgttaatctatgatccagccaaacgaatttctggcaaaatggcactgaatcatccatattttaatgatttggacaatcagattaagaagatgtagctttctgacaaaaagtttccatatgttatgtcaacagatagttgtgtttttattgttaactcttgtctatttttgtcttatatatatttctttgttatcaaacttcagctgtacttcgtcttctaatttcaaaaatataacttaaaaatgtaaatattctatatgaatttaaatataattctgtaaatgtgtgtaggtctcactgtaacaactatttgttactataataaaactataatattgatgtcaggaatcaggaaaaaantttgagttggcttaaatcatctcagtccttatggcagttttattttcctgtagttggaactactaaaatttaggaaaatgctaagttcaagtttcgtaatgctttgaagtatttttatgctctgaatgtttaaatgttctcatcagtttcttgccatgttgttaactatacaacctggctaaagatgaatatttttctactggtattttaatttttgacctaaatgtttaagcattcggaatgagaaaactatacagatttgagaaatgatgctaaatttataggagttttcagtaacttaaaaagctaacatgagagcatgccaaaatttgctaagtcttacaaagatcaagggctgtccgcaacagggaagaacagttttgaaaatttatgaactatcttatttttaggtaggttttgaaagctttttgtctaagtgaattcttatgccttggtcagagtaataactgaaggagntgcttatcttggctttcgagtctgagtttaaaactacacattttgacatagtgtttattagcagccatctaaaaaggctctaatgtatatttaactaaaattactagctttgggaattaaactgtttaacaaataaaaaaaaaaaaaanannnntacctataagcagcctaatttgaattatttgctccatctacatagtg

>HN1_probe1

GGCCTCTAATATCTTTGGGACACCT

>HN1_probe2

GAAGGAACTCCTCTGAAGCAAGCTC

>HN1_probe3

GACTTGGAGTCATCTGGACTGCAGA

>HN1_probe4

AGAGTGAAGAGAAGCCCGTGCCTGC

>HN1_probe5

GACCCCAACAGCAGGAATAGCTCCC

>HN1_probe6

GTGGTGGATCCAATTTTTCATTAGG

>HN1_probe7

CATCCAGAAGAAATCCCCCTGGCGG

>HN1_probe8

ACAACCACCACCTTCAAGGGAGTCG

>HN1_probe9

GCAAGCTCCGGAGACTTCTTAGATC

>ZWINT_probe1

GATGTACCTTTTTTGTCAACTCTTA

>ZWINT_probe2

TAGTGATACCTTGATCTTTCCCACT

>ZWINT_probe3

GTTTCATTGACCTCTAGTGATACCT

>ZWINT_probe4

GTACAGCCTAGTGTTAACATTCTTG

>ZWINT_probe5

GATTGGCTTTTGTCATCCACTATTG

>ZWINT_probe6

AACATTTCTCGATCACTGGTTTCAG

>ZWINT_probe7

TTTCCCACTTTCTGTTTTCGGATTG

>ZWINT_probe8

GGCCTCCTATGATGCAGACATGGTG

>ZWINT_probe9

TCTTGGTATCTTTTTGTGCCTTATC

>ZWINT_probe10

AGGAGCTGGGACTGGTTTGAACACA

>ZWINT_probe11

CAGATGGGGAGGGGGTACTGGCCTT

>ZWINT_target

aggagctgggactggtttgaacacagggtgtgcagatggggagggggtactggccttgggcctcctatgatgcagacatggtgaatttaattcaaggaggaggagaatgttttaggcaggtggttatatgtgggaagataattttattcatggatccaaatgtttgttgagtcctttctttgtgctaaggttcttgcggtgaaccagaattataacagtgagctcatctgactgttttaggatgtacagcctagtgttaacattcttggtatctttttgtgccttatctaaaacatttctcgatcactggtttcagatgttcatttattatattcttttcaaagattcagagattggcttttgtcatccactattgtatgttttgtttcattgacctctagtgataccttgatctttcccactttctgttttcggattggagaagatgtaccttttttgtcaactctta

>ZWINT_exemplar

ggcacgagctcggctcctggaaagatggaggcagcggagacagaggcggaagctgcagccctagaggtcctggctgaggtggcaggcatcttggaacctgtaggcctgcaggaggaggcagaactgccagccaagatcctggttgagtttgtggtggactctcagaagaaagacaagctgctctgcagccagcttcaggtagcggatttcctgcagaacatcctggctcaggaggacactgctaagggtctcgaccccttggcttctgaagacacgagccgacagaaggcaattgcagctaaggaacaatggaaagagctgaaggccacctacagggagcacgtagaggccatcaaaattggcctcaccaaggccctgactcagatggaggaagcccagaggaaacggacacaactccgggaagcctttgagcagctccaggccaagaaacaaatggccatggagaaacgcagagcagtccagaaccagtggcagctacaacaggagaagcatctgcagcatctggcggaggtttctgcagaggtgagggagcgtaagacagggactcagcaggagcttgacggggtgtttcagaaacttggaaacctgaagcagcaggcagaacaggagcgggacaagctgcagaggtatcagaccttcctccagcttctgtataccctgcagggtaagctgttgttccctgaggctgaggctgaggcagagaatcttccagatgataaaccccagcagccgactcgaccccaggagcagagtacaggagacaccatggggagagaccctggtgtgtccttcaaggctgttggtctacaacctgctggagatgtaaatttgccatgacttcctggaggacagcagcatggagaaagatcctagaaaaggcctctgacttccctcacctcccaaccatcattacaggaaagactgtgaactcctgagttcagcttgatttctgactacatcccagcaagctctggcatctgtggattaaaatccctggatctctctcagttgtgtatttgttcatcttcatatgctggcaggaacaactattaatacagatactcagaagccaataacatgacaggagctgggactggtttgaacacagggtgtgcagatggggagggggtactggccttgggcctcctatgatgcagacatggtgaatttaattcaaggaggaggagaatgttttaggcaggtggttatatgtgggaagataattttattcatggatccaaatgtttgttgagtcctttctttgtgctaaggttcttgcggtgaaccagaattataacagtgagctcatctgactgttttaggatgtacagcctagtgttaacattcttggtatctttttgtgccttatctaaaacatttctcgatcactggtttcagatgttcatttattatattcttttcaaagattcagagattggcttttgtcatccactattgtatgttttgtttcattgacctctagtgataccttgatctttcccactttctgttttcggattggagaagatgtaccttttttgtcaactcttacttttatcagatgatcaactcacgtatttggatctttatttgttttctcaaataaatatttaaggttaaaaaaaaaaaaaaaaaaa

>ASPM_probe1

ATAGAGCCTCTGATGTACGAAGTAG

>ASPM_probe2

CAGTCTCTACAAACTTACAGCTCAT

>ASPM_probe3

AATCCCCTGCAAGCTATTCAAATGG

>ASPM_probe4

GAAGAAATCACAAATCCCCTGCAAG

>ASPM_probe5

GTGATGGATACGCTTGGCATTCCTT

>ASPM_probe6

GCATTCCTTTTATCCCAGAAACACC

>ASPM_probe7

GTTGTTTGTTGGCTATTTTACTGAA

>ASPM_probe8

GGAGCTTTTGCAGATATACCGAGAA

>ASPM_probe9

TCAGATATGCTGTGCAAGTCTTGCT

>ASPM_probe10

GTTGTTGACCGTATTTACAGTCTCT

>ASPM_probe11

GTTGTAATCGCAGTATTCCTTGTAT

>ASPM_target

gttgtaatcgcagtattccttgtatggaagtcatcagatatgctgtgcaagtcttgcttaatgtatctaagtatgagaaaactacttcagcagtttatgatgtagaaaattgtatagatatactattggagcttttgcagatataccgagaaaagcctggtaataaagttgcagacaaaggcggaagcatttttacaaaaacttgttgtttgttggctattttactgaagacaacaaatagagcctctgatgtacgaagtaggtccaaagttgttgaccgtatttacagtctctacaaacttacagctcataaacataaaatgaatactgaaagaatactttacaagcaaaagaagaattcttctataagcattccttttatcccagaaacacctgtaaggaccagaatagtttcaagacttaagccagattgggttttgagaagagataacatggaagaaatcacaaatcccctgcaagctattcaaatggtgatggatacgcttggcattcctt

>ASPM_exemplar

gcaaacatactttaataagttaaagaaaataacaaaaacagtacagcaaagatactgggcaatgaaagaaagaaacatacaatttcaaaggtataacaaactgaggcattctgtaatatacattcaggctatttttaggggaaagaaagctagaagacatttaaaaatgatgcatatagccgcaactctcattcagaggagatttagaactctaatgatgagaagaagattcctctctctcaagaaaactgctattttgattcagagaaaatatcgggcacatctttgtacaaagcatcacttacagttccttcaggtacaaaatgcagttattaaaatccagtcatcatacagaagatggatgataaggaaaaggatgcgagagatgcacagggctgctactttcatccagtctactttcagaatgcacagattacatatgagatatcaggctttgaaacaggcctccgttgtgatccaacagcaataccaagcaaatagagctgcaaaactgcagaggcagcattatctcagacaaagacactctgctgtgatccttcaggctgcattcaggggtatgaaaactagaagacatttgaagagtatgcattcctctgcaacccttattcagagtaggtttagatcattactggtgaggagaagattcatttccctcaaaaaagctactatttttgttcagaggaaatatcgagccaccatttgtgccaaacataaattgtaccaattcttgcacttaagaaaggcagccattacaatacagtcatcttacagaagactgatggtaaagaagaagttacaagaaatgcaaagggctgcagttctcattcaggctactttcaggatgcacagaaaaaaaaatatattacatttcagacttggaaacatgcttcaattctaattcagcaacattatcgaacatatagagctgcaaaattgcaaagagaaaattatatcagacaatggcattctgctgtggttattcaggctgcatataaaggaatgaaagcaagacaacttttaagggaaaaacacaaagcttctattgtaatacaaggcacctacagaatgtataggcagtattgtttctaccaaaagcttcagtgggctacaaaaatcatacaagaaaaatatagagcaaataaaaagaaacagaaagtatttcaacacaatgaacttaagaaagagacttgtgttcaggcaggttttcaggacatgaacataaaaaaacagattcaggaacagcaccaggctgccattattattcagaagcattgtaaagcctttaaaataaggaagcattatctccacattagagcaacagtagtttctattcaaagaagatacagaaaactaactgcagtgcgtacccaagcagttatttgtatacagtcttattacagaggctttaaagtacgaaaggatattcaaaatatgcaccgggctgccacactaattcagtcattctatcgaatgcacagggccaaagttgattatgaaacaaagaaaactgcaattgtggttatacagaattattataggttgtatgttagagtaaaaacagaaagaaaaaactttttagcagttcagaaatctgtacgaactattcaggctgcttttagaggcatgaaagttagacaaaaattgaaaaatgtatcagaggaaaagatggcagccattgttaaccaatctgcactctgctgttacagaagtaaaactcagtatgaagctgttcaaagtgaaggtgttatgattcaagagtggtataaagcttctggccttgcttgttcacaggaagcagagtatcattctcaaagtagggctgcagtaacaattcaaaaagctttttgtagaatggtcacaagaaaactggaaacacagaaatgtgctgccctacggattcagttcttccttcagatggctgtgtatcggagaagatttgttcagcagaaaagagctgctatcactttacagcattattttaggacgtggcaaaccagaaaacagtttttactatatagaaaagcagcagtggttttacaaaatcactacagagcatttctgtctgcaaaacatcaaagacaagtctatttacagatcagaagcagtgttatcattattcaagctagaagtaaaggatttatacagaaacggaagtttcaggaaattaaaaatagcaccataaaaattcaggctatgtggaggagatatagagccaagaaatatttatgtaaagtgaaagctgcctgcaagattcaagcctggtatagatgttggagagcacacaaagaatatctagctatattaaaagctgttaaaattattcaaggttgcttctataccaaactagagagaacacggtttttgaatgtgagagcatcagcaattatcattcagagaaaatggagagctatacttcctgcaaagatagctcatgaacacttcttaatgataaaaagacatcgagctgcttgtttgatccaagcacattatagaggatataaaggaaggcaggtctttcttcggcagaaatctgctgctttgatcatacaaaaatatatacgagccagggaggctggaaagcatgaaaggataaaatatattgaatttaaaaaatctacagttatcctacaagcactggtgcgtggttggctagtacgaaaaagatttttagaacagagagccaaaattcgacttcttcacttcactgcagctgcatattatcacctgaatgctgttagaattcaaagagcctataaactttacctggctgtgaagaatgctaacaagcaggttaattcagtcatctgtattcagagatggtttcgagcaagattacaagaaaagagatttattcagaaatatcatagcatcaaaaagattgagcatgaaggtcaagaatgtctgagccagcgaaatagggctgcatcagtaatacagaaagcagtgcgccattttctcctccgtaaaaagcaggaaaaattcactagtggaatcattaaaattcaggcattatggagaggctattcttggaggaagaaaaatgattgtacaaaaattaaagctatacgactaagtcttcaagttgttaatagggagattcgagaagaaaacaaactctacaaaagaactgcacttgcacttcattaccttttgacatataagcacctttctgccattcttgaggccttaaaacacctagaggtagttactagattgtctccactttgttgtgagaacatggcccagagtggagcaatttctaaaatatttgttttgatccgaagttgtaatcgcagtattccttgtatggaagtcatcagatatgctgtgcaagtcttgcttaatgtatctaagtatgagaaaactacttcagcagtttatgatgtagaaaattgtatagatatactattggagcttttgcagatataccgagaaaagcctggtaataaagttgcagacaaaggcggaagcatttttacaaaaacttgttgtttgttggctattttactgaagacaacaaatagagcctctgatgtacgaagtaggtccaaagttgttgaccgtatttacagtctctacaaacttacagctcataaacataaaatgaatactgaaagaatactttacaagcaaaagaagaattcttctataagcattccttttatcccagaaacacctgtaaggaccagaatagtttcaagacttaagccagattgggttttgagaagagataacatggaagaaatcacaaatcccctgcaagctattcaaatggtgatggatacgcttggcattccttattagtaaatgtaaacattttcagtatgtatagtgtaaagaaatattaaagccaatcatgagtacgt

>SLC35E3_probe1

AGTAGCTCTCTGCTTGCTGATAGAT

>SLC35E3_probe2

ATTTTAGTTTAGCTTCCTGATTTAT

>SLC35E3_probe3

GATGGTTTCCCAGTGTGAGATTTGT

>SLC35E3_probe4

ATGGTTTGGTTGGTCCCAGCAAAGT

>SLC35E3_probe5

GTTCTCGGTGTTCAAACTCTTCTAA

>SLC35E3_probe6

TGCAAATATGCTGTGGGTTCTCGGT

>SLC35E3_probe7

AAGGAATTGCTGTTACTGTACTGCA

>SLC35E3_probe8

TAAATCTAGTGTTTCTATTTTAGTT

>SLC35E3_probe9

ATATACCATCCCATATATATGTGGG

>SLC35E3_probe10

CTGCTTGCTGATAGATGGTTTCCCA

>RNASEH2A_probe1

ATGCCAGAGACATACCAGGCGCAGC

>RNASEH2A_probe2

GGAAGATCACATCCTACTTCCTCAA

>RNASEH2A_probe3

ACTGATTATGGCTCAGGCTACCCCA

>RNASEH2A_probe4

TGCAGCAAAGTTTTCCCGGGATTGA

>RNASEH2A_probe5

CTCAATGAAGGGTCCCAAGCCCGTC

>RNASEH2A_probe6

TCGTGGACACCGTAGGGATGCCAGA

>RNASEH2A_probe7

GAGGACTCAGCATCCGAGAATCAGG

>RNASEH2A_probe8

TCTTCCCACCGATATTTCCTGGAAC

>TSC2_probe1

TGGCCTCACAGGTGCATCATAGCCG

>TSC2_probe2

GGGCAACGACTTTGTGTCCATTGTC

>TSC2_probe3

CCCTGATGCCCACCAAGGACGTGGA

>TSC2_probe4

AGCACCGCTGCGACAAGAAGCGCCA

>TSC2_probe5

TCAACTTTGTCCACGTGATCGTCAC

>TSC2_probe6

GTGAGGACTTCAAGCTTGGCACCAT

>TSC2_probe7

TGGACTACGAGTGCAACCTGGTGTC

>FAM20B_probe1

GTATTAATAAGGCATTGCCCCCTGT

>FAM20B_probe2

TGTAAGGCTGCATTGTGGGTTTGGG

>FAM20B_probe3

GTTTTGTAACACTGTCCTACTTTAT

>FAM20B_probe4

AGTCGTTGCAGGGTTTGGATCAGCT

>FAM20B_probe5

GGATCAGCTGTAAGTTAGGTATGCC

>FAM20B_probe6

AAGGCTGTTACAATCAAGTCGTTGC

>FAM20B_probe7

AGATGAGTCCTATACGTGGCAATTT

>FAM20B_probe8

TCTGAAGCCAGCATTATCTTCCAAA

>FAM20B_probe9

GCCCCCTGTTTGCACTCAGGGTTAA

>FAM20B_probe10

CGTGGCAATTTTTCAATGTCATCTG

>FAM20B_probe11

TCAGAACTCATGGCCATTTCCTGCC

>FAM20B_target

gttttgtaacactgtcctactttatttattagaatctaaggctgttacaatcaagtcgttgcagggtttggatcagctgtaagttaggtatgcctaccaaacatccaaaggtagacgtggagacattttaatactacaaaactaggaaaatcagaactcatggccatttcctgccctcctccaacttgttaaaacatgtttattctaaagttcgaatggataaatttgagtataaaggttttgttataaaactgttctttagtgtaaggctgcattgtgggtttgggggaaatgtaaataattttctgtgtaaaacaaattcataggatctgatttgctcagagtattattcaagaatgtattaataaggcattgccccctgtttgcactcagggttaatatgtcaaatgaaatttaagaaggaaatggaagaattcaggtacattaattgcatattattttgggaaagatgagtcctatacgtggcaatttttcaatgtcatctgaagccagcattatcttccaaa

>FAM20B_consensus

gtgtgggactgccgctctgcgcggcgagaggtggcctgggaatggccgggccgggggtgggccggagccgctgtggcggcggcggcggctgggggcggtgagcgcggcgtggggctgcccctccccggaggcggcgggggcggccggggccgcgccgcaccgcaccgcgcgggcggccatggagcgagcctagggcccgacaggaactgtggaaggtgcatcagtgaagaaatggaccaatgtgtataatcatggaatctccttgctaaccatcaccaccagctctccttaatacatgagcaagagtgggtcaggggagaaggaaaagaggtcaacatgaagctaaagcagcgagtcgtgctgttagcaattctccttgtcatttttatcttcaccaaagttttcctgattgacaacttagatacatcagctgccaaccgggaggaccagagggcctttcaccgaatgatgactggcttgcgggtggagctggcacccaagctggaccataccttgcagtctccctgggagattgcagcccagtgggtggttccccgggaagtgtaccctgaagagacaccagagctgggggcagtcatgcatgccatggccnnnnngnaaatcattaaagctgatgtgggttataaagggacacagctgaaanncttactgatacttgaaggaggccagaaagttgttttcaaacctaagcggtatagccgagaccatgtggtggaaggggaaccgtatgctggttatgatagacacaatgcagaggtagcagcctttcacttggacaggattctgggtttccaccgagcccccttggtagttggcagatttgttaatcttcggacagagatcaaacctgtcgccacagagcagctgttgagcaccttcctaactgtaggaaacaatacttgtttttatgggaagtgctattactgccgagaaacagaaccagcttgtgctgatggagacataatggagggatctgtcacactttggcttccagatgtgtggcctctgcagaagcaccgtcacccatggggcaggacttaccgagaaggcaaattggccaggtgggagtatgatganagctactgtgatgctgngaanaaaacntccccttatnanctntggcccgcncctnttggacatnattgacancagctgnctttgattacctgattggcaatgctgaccgccatcactatgagagctttcaagatgatgaaggcgctagtatgctcatccttcttgataatgccaaaagctttgggaacccctcgctggatgaaagaagcattcttgcccctctctatcagtgttgcatcattcgggtgtccacctggaacagactgaactacctaaagaatggtgtgctaaagtctgccttaaaatctgccatggcccatgaccccatctccccagtgctctctgatcctcatctggacgccgtggaccagcggctcctgagtgtcctggccaccgtgaagcagtgcaccgaccagtttgggatggacacagtactggtggaagacaggatgcctctctcacacttgtaattctcgacacaaaataagtgaaacttctttttacaaagatagagaaacagcacaatcaattccaaatggtatgagatggattggaagtggccagcagcaagttctggtgacnggacagngtggccttggatgtctttggtattttctgtagtagaaactaaagcaaagaccacaagtttcagagcatggagacattcctgctgaatcgccttctcacctcctcggcaattgctcattctagggttgggcatcatagttggtcagtcttaattcccatgccaaaggacaaacaggtgtgacatttggatagatgaatactgggattggctctggagcatgtgttttgagttgaaccttgcagtcctttctctacgcccgtggattttgtggaaacactttgcaatctctttgtctttttttttttaccagaactagttacattggaatgcttactgtcctacagagtggcagcaaataaaaccttgcattccatcaagccaaaatagcacactctgttagaggagatacatgtttaagatagaattggagggaaggacaaaaacagaaaaatgtttgggcttttaagccattgggtagtattgntttgatgatcttagaggagggaagaagagagagagacccaatggtagaaccagaatcagggagatgactgaactactgnaaaacaggttcccttgtatttaggatcttaaggtgtataaaaagcaaacatgactttgcacctaagtaaattctgcattctcatagttgtgtcccaattaaccaaaaagttgtctctagagaaaatantattacaatctaagcatgattctctgtggagactaattttttccccttttgccaaaagcagtccttcccaaattaacaaagcaaactgaaataataccttgaataacaggttgcctgtggtctctgtcatcctcgtctctcttctgaaatgaatttccacctctgcctttaaggcatttttgtcactgaagctgctgttcccaagagataggcaacctttttgtccctttctcataagaaagggacactcctacaggtgagagtgtataccttactctctcagataagtggctggacttatcttgtgatttggggccatggaagattggaaacaaagattttaagccttcttcttttttgcttttttcttttttttttgagaccaagtctcactctgttgcccaggctggagtgcagtggcacgatcttggctcactgcaacctccgtctcccaggttcaagcgattctcttgcctcagcctccagagtagctgggattacaggcgcccgccatcgtgcccagctaatttttatatttttagtggagacagggtttcgggtttcaccatgttggccaggttgatcttggactcctgaccccaggtgatccacctgcctcagccttccaaagtgctgggattacaggcatgagccaccgtggccggccaagattttaagccttctgagccttgaaattgaggaggttaaaaggaagagccttaagattttgatttatgtcaaatcctaattctatcattcagtcttgtttggagttctgaacccatgatgttgtattatgcttctttctcctcttagcactctcaaatttcaggtttgtaaaacacagtttttgttttgtgttctggcaaagtgatctcaacatgtaagtagttgcagtaaaacacaggggcaaaggaagacaggcctgatgtgcccactcatctatggactcagagctgtgtgctttgctcctgcatcttgttgaggtgctgttccagctttgcatttctgtcaagtagaggcgaatatataaacagtgtggttgaatacatttaatgccagccattggaaactagttttaggcaaccactctcaaaaacagctttagaatttatgcccagttttcttgcattgaaagataactgagtaataacctgtaactatttttaaatggcatgaaattaggaaacttttgtacattttatatacattttgagatgaacagaacaatgggctgagttataaaaagcgtgtattgaatttaagaagacagactagcacaaaacacagaattcgtgttaaccaaaggaggcattgatttcagttttaaggctactcagtgttgtgtgtccagggaaattcacagctcagtatgagaataccttggttagtgctcacccacaagcttccaggagccagctgggaggagacaataggaagagatgtcagctctgctctccctgtaaatgttagttgaactaagttatggatttgtggtctttcaaatacatgacgcctttagtatgccacactgaaatgaataagaagtcttctgaaactgggaacttcataacattgaaggcagaagattctgctaaggaaaaaagcaggcaggaaagaaaatgtctcatcctttcttgaaagcatttgcagaaaatatatcatttcattttattcccatctgttttcaaactcgtgatcttaaaaggcattctgatgataaatttagaattttcatctataaaatttagaactctaatccataaagttagaattgagctaatagagtggtatgacatggcactaaaaatataaatttttgttgtaagtcaggattggagtaagctggaaaagtatgtttaggcaaatcttggagaaaaccaaccataaacttacagctctaaaattcagaaagccctaaaatttcaaacactgtttgaaagaagaggtgggggccgggtgccgtggctcatgcctgtcatcccagcatttgggaggctgaggcnggcagatcacctgaggccaggagttcgagaccagcctggctggctagcatggtgagaccgtctctactaaaaatgcaaaaattaacagggcacggtggcatgcgcctgtagtcccagctactcgggaggctgaggcaggagaatcacttgaatccaggaggcgaaggttgcagtgagctgagattgtgctgctgcactccagcctgggagacagagcgagactctgtcttaaaaaaaaaaaaaggaggtgaatttttttttaagttttgtaacactgtcctactttatttattagaatctaaggctgttacaatcaagtcgttgcagggtttggatcagctgtaagttaggtatgcctaccaaacatccaaaggtagacgtggagacattttaatactacaaaactaggaaaatcagaactcatggccatttcctgccctcctccaacttgttaaaacatgtttattctaaagttcgaatggataaatttgagtataaaggttttgttataaaactgttctttagtgtaaggctgcattgtgggtttgggggaaatgtaaataattttctgtgtaaaacaaattcataggatctgatttgctcagagtattattcaagaatgtattaataaggcattgccccctgtttgcactcagggttaatatgtcaaatgaaatttaagaaggaaatggaagaattcaggtacattaattgcatattattttgggaaagatgagtcctatacgtggcaatttttcaatgtcatctgaagccagcattatcttccaaagaaatcgatcttttttttctaaaaaaaaaaaatgcttttgccttcccttcccttcccatccgccatatttcttcagcctttcttctcgatcacccgtgtattctttgaccagtaaatgaccacacctcaatgatggtaaaacagcatcatcagtaagctatcttatatgcctcatcctgtgagtttgagcttcaggaaacatgagtaaaagtatatgtaatgtatatagtcgtatatgtattctagcaagaaaaacatatttattttgacaaaggggaacactgactttctgaaggattcagaaagaaccttagtgaaaggttctcagtctctgagagtggaccctaattaacataaagaccattcatcagcgaataactactgagcaactctagtgtgccagcacaggccagacatactagtgagccaggcacatctggccttgggaaactcatcctacaggggaaggccagtttttttcccttcaattcctcaagtctgggtggtgacaaggtaggggctaggtactggactaccacaggtttttaggaactaaggtgtttctcataaacacaaaatgttgggtgaaactgggaacaactactcagaagctnatttatttgcttaaatggaaagtgtgggagccactaccctctcttttgatctgccaaggatttcctctcagagctgttgcacagacagagattgtacttggtaagataccaaacaagacagatatggatctaaatttctaatgtgttctatgggtttcaattctgaaaaaagaaaatgaataaagattttaataaatattgaaaaaaaaaaaaaaaaannnccgctcggacgcgt

>WASL_probe1

GAGATACTTGTCAAGTTGCTCTTAA

>WASL_probe2

TGGGCCGTCGACAAAGGAAATCTGA

>WASL_probe3

AATTTCGAAAAGCAGTTACAGACCT

>WASL_probe4

AATCTACCCATGGCTACAGTTGATA

>WASL_probe5

GTGGGAACAAGAGCTATACAATAAC

>WASL_probe6

TAGTCCTAGAGGATATTTTCATACC

>WASL_probe7

GATCCCCCAAATGGTCCTAATCTAC

>WASL_probe8

AGAAAAGACGAGATCCCCCAAATGG

>WASL_probe9

TACCCATGGCTACAGTTGATATAAA

>WASL_probe10

TTCATACCTTTGCTGGAGATACTTG

>WASL_probe11

ATACCTTTGCTGGAGATACTTGTCA

>WASL_target

gtgggaacaagagctatacaataactttgtatataatagtcctagaggatattttcatacctttgctggagatacttgtcaagttgctcttaattttgccaatgaagaagaagcaaaaaaatttcgaaaagcagttacagaccttttgggccgtcgacaaaggaaatctgagaaaagacgagatcccccaaatggtcctaatctacccatggctacagttgatataaa

>WASL_consensus

agggtatgaaatagtgtttccttctgcattataagtaaattacttaaggtttttgtcagtctcacatatagtatttaaactccctngttgatggaatgnaaaatanttcacaantcaagtgagcatcctggtgtaaacttgcacacantaacagggagtagctttgtagaggattacgacaancctttacagattaaatgaggtattgcacagattaanaaaaaagcaaaanaggcancanaaatatacagcaanttggtagaacatttacatgataattttacagaatccacagacagaaagtagtgtttagtatttcaccttaaaaatatntatatatataatatatagatcagtcttcccactcatcatcatcctcaaaatcttcttcatcatcttcatcttcatcttcatctgaagaatgaatggctttgctccttttctgcatcacttccattaatgcacccacaattcctgaagtgggtgcaggtgttggtggtgtagactcttggccatcagccacagattttagttggataccctgtcgtatctggtctaacagtgcatctcgtccagagcaggacactggccgactgttctgctccactttttttagctgagcaccctctctaatttgatctaaaagagctgctttgtttcctgcagtagttggaacctgatggtccccatcagaaggcaggccaggcgggggcggtggcccaggaggaggtggaggtggaggcggtgggggtggtgccactggccctacccccaacacagatggaggtggtggtggaggccctgaaggtgctgaggagggaagggctggaggtggaggagggtacatcctatttggcggtggtggagggacttctacacttggcctggaaggaggcggtggtggaggtgcagctgtgggagctcttgaaggtggtgggggaggagcgcctcttcccctagcaggaggaggaggaggacccgagctatgtggagggggaggaggaggaggtggccctccccttgatggtggtggaggtggtggtgcttgcctccgcagttcatttttaacagcttcaacacctcctgttttttcaataaagtcatatataacttttaatgtttctctttctttaagttgtgcctctaagattccacacatatcaaaaagattcttcaattctggatccaaattattcagatcagagcctgtatttggatcccaaccaacatgtccaatgtgctggaaattgcttggtgttcctatatctnccttggttaatctcttctttttagcttttcccttcttcttttctttggtatgggagatgttgttgacttgtggaccataaaatctatttgttgtgatttctggattttttatatcaactgtagccatgggtagattaggaccatttgggggatctcgtcttttctcagatttcctttgtcgacggcccaaaaggtctgtaactgcttttcgaaatttttttgcttcttcttcattggcaaaattaagagcaacttgacaagtatctccagcaaaggtatgaaaatatcctctaggactattatatacaaagttattgtatagctcttgttcccacaatagtttcccatccttaatgtcaaatattcttaaaaaataagatctctgtggattgtccttaacaagacaagcaacaccactgcacttctttgaccacatacagttccgatctgctgcatataactgcaccactgctgaagacatagtcacacatttcttgccgaggaaagtgaagagggactcgttctcctgcggggtgagcaacagngaccccacgttggtgaccctccgcggcggcggcggctgctgctggacggagctcatggtttcgccggcggggttgggagtccagggccgtctcntccggcgagtgggcgagagctcgttccccctctcggtgacaggggcggggagaagtggagtcagaggcgccacatctcgnngctcntccggagcggggaggaggacgaggtcgagggaagcaggcgctgacggcgagccaccttcgcgccgcgctgagaaagggaagctcccggcacccgcccggccaggctagggccggatggtcgttgtccnnnnncnccggcgactgcgctaaactcncaactcctcccccaaccctcccgcagcggcggcggcggccgcacaatcc

>AIM1_probe1

GCCTGTGCTGAACTGATCTCTTAAA

>AIM1_probe2

AATACTGGTGCTCTTGTCACAGGTA

>AIM1_probe3

AAAATGCTGATCTTCTCTGGAGTCT

>AIM1_probe4

TGCTCTTCCAACAGTGGGTTCTAGC

>AIM1_probe5

ACAACTGACAAGACACCAGCCCATA

>AIM1_probe6

TTAGGCCTTTTGTGCATACCATTAC

>AIM1_probe7

GGTGCATGTACAACAGCATCCAACA

>AIM1_probe8

TCTTTTTGTCCTCATCACTCAATAC

>AIM1_probe9

GCAATCTTGGAATCCTCAACTGCAG

>AIM1_probe10

GGTAGAACAGCTTGTTTCTTTTCCA

>AIM1_probe11

GCATCCAACATATCTGTCTTGTTCC

>AIM1_target

gcaatcttggaatcctcaactgcagtaagcatttcaaaatgcaaacaaactgcttaacaactgacaagacaccagcccatangctgctcttccaacagtgggttctagctttgaacaaaagtgctaaacatttccttgaatatattcttcctctttttgtcctcatcactcaatactggtgctcttgtcacaggtagaacagcttgtttcttttccatctattcaagtgtgtttctaattctaaaatgctgatcttctctggagtctatggtaggcaattatggtcactggaatagtttgtcttgttttaaaatattattggtgcatgtacaacagcatccaacatatctgtcttgttcctagatatatagctctgattttaggccttttgtgcataccattacaatatggtggggtaagacattctacagtagcctgtgctgaactgatctcttaaa

>AIM1_consensus

cagctccgaggggagtcggaccggagcaaacagccacccccggcttcgtcccccacgaagaggaagggcaggagccgtgccctcgaggccgtgcccgccccgcccgccagcggcccccgggctcccgccaaggagtccccacccaagagggtgcccgatcccagcccagtcaccaagggcactgcggccgagagcggggaggaggcggcgcgggccatcccccgcgagctcccggtcaagagcagctcgctgctgccggagatcaagcccgagcacaagaggggcccgctccccaaccacttcaacggccgggcagagggaggtcgaagcagagagctgggcagagcggccggagcgcctggagcttctgacgccgacggcttgaagcccaggaaccatttcggcgtgggcaggtcgacagtgaccactaaagtgaccctccctgccaagcccanacatgtggaactaaatcttaaaacccctaagaatcttgacagtttgggaaatgagcacaatccatttagccagccagttcacaaagncaacactgccaccaaantctcctnatttgaaaacaaacggacaaacagtagcccaagacacactgacattcgaggccnaaggaatactcctgcctctagtaaaacgtttgttgggagggcaaagctgaatttagccaaaaaagccaaagaaatggagcaacctgaaaagaaagtaatgccannnannnnncnnnntggtgtgntggttaaggaaactgctatagaaaccaaagttaccgtctcggaagaagagattctgccagcaaccagaggaatgaatggagactcttctgagaatcaagctcttggtcctcagcctaaccaagatgataaagcagatgtacaaacagatgctggctgcctttcagaaccagtggcttctgctctgattcctgtcaaggatcataagctcttagagaaggaggactcagaggctgcagacagcaaaagccttgtacttgaaaatgtaaccgatacagcacaagacatccccaccactgtggataccaaaganttacctccaacgnccatgnnnnagnnacancanacnnnntctgactcacagtcccctgctgagtcatctcctgggccttctctttcactgtctgcacccgctcctggggatgttcccaaagacacatgtgttcaatcacccataagcagtttcccatgcactgatctaaaagtgtcagaaaaccataaaggatgtgttttgcctgtgtctcgtcagaacaatgagaaaatgccacttttagaacttggaggagaaacaacccctcctttgtccacagagcgtagtccagaagctgtgggaagtgagtgtccatccagagtcctcgtccaggtcaggtccttcgtgctccccgtggagagcacccaggatgtgagctcccaggtcatcccagagagctctgaagttagagaagtgcagttgccaacttgtcacagtaatgaacctgaagtggtttccgttgcaagttgtgctcccccacaagaggaagtactgggcaatgaacactctcattgcacagcagagctcgcggcaaaatctggcccacaagtcataccgccagcatcagagaaaactctgcctattcaggctcaaagtcagggcagcagaacacccctgatggctgaatccagtcccaccaactctcccagcagcggaaatcacttagccactcctcaaaggccagatcagactgttacaaatggccaggatagccctgccagccttttgaacatttctgctggtagtgatgatagtgtatttgattcttcttctgatatggaaaaattcactgaaattataaaacagatggatagcgcagtttgtatgcccatgaaaagaaagaaggccaggatgccaaactctcctgctcctcactttgccatgcctcctattcacgaagaccatttagaaaaggtgtttgatcccaaagtgtttacctttggtttggggaagaagaaggaaagtcagccagaaatgtcaccggctttacatttgatgcagaaccttgacacaaaatccaaactgagacccaaacgtgcatctgctgaacagagcgtcctcttcaagtccctgcacaccaacactaatgggaacagtgagcctctggtgatgccggaaatcaatgacaaagagaacagggacgtcacaaatggtggcattaagagatcgagactagaaaaaagtgcacttttctcaagcttgttatcttctttaccacaagacaaaatcttttctccttctgtgacatcagtcaacactatgaccacggctttcagtacttctcagaacggttccctatctcagtcttcagtgtcacagcccacgactgagggtgccccgccctgtggtttgaacaaagaacagtcaaatcttctgcccgacaactccttaaaggtcttcaatttcaactcgtcaagtncatcacactccagtttgaaaagtccaagccacatggaaaaatacccgcanaaagagaaaaccaaagaagatctggattcacgaagcaacctacacttgccagaaactaaattttctgaattgtcaaaactgaagaatgatgatatggnnnnggctaatcatattgaaagtgttattaaatcaaacttgccaaactgtgcaaacagtgacaccgacttcatgggtcttttcnnntcaagccggtatgacccaagcatttctttttctggaatgtcattatcagacacaatgacacttagaggaagtgtccaaaataaactcaatccccgacctggaaaggtagtgatatatagtgaacccgacgtctctgagaagtgcattgaagttttcagtgacattcaggattgcagttcttggagcctctctccagtgatactcataaaagttgttagaggatgttggattttgtatgagcaaccaaattttgaagggcactccatccccttagaagaaggagaattggnactctctggtctctggggtatagaagacattttggaaaggcacgaagaagcagagtctgataagccagtggtgattggttccatcagacatgtggttcaggattacagagttagtcacattgacttatttactgaaccagaagggttaggaatcctaagttcctactttgatgatactgaagaaatgcagggatttggtgtaatgcagaagacttgttccatgaaagtacattggggnacgtggctgatttatgaagaacctggatttcagggtgttcctttcatcctggaacctggtgaataccctgacttgtccttctgggatacagaagcagcgtacattggatccatgcggcctctgaaaatgggtggccgtaaagttgaattccctacagatccaaaggtagttgtttatgaaaagcctttctttgaaggaaaatgtgtgganctagaaacaggaatgtgtagttttgtcatggagggaggtgaaacagaagaggcgactggagacgatcatttgccgtttacgtcagtggggtctatgaaagttctaagaggcatttgggttgcatatgagaagcctggatttaccggtcatcagtatttgctagaagaaggagaatacagggactggaaagcctggggaggttacaatggagagcttcagtctttacgacctatattaggtgatttttcaaatgctcacatgataatgtacagtgaaaaaaactttggatccaaaggttccagtattgatgtattgggaattgttgctaatttaaaggagactggatatggagtgaagacacagtctattaatgtactgagtggagtatgggtagcctatgaaaatcctgacttcacaggagaacagtatatactggataaaggattttataccagttttgaggactggggaggcaaaaattntaagatctcttctgttcaacctatatgtttggattctttcactggcccaaggagacgaaatcagattcacttgttttcagaaccacagtttcaaggtcacagtcaaagttttgaagaaacaacaagtcaaattgatgattcattttctaccaagtcttgcagagtttcaggaggcagctgggttgtatatgatggagaaaatttcactggtaatcaatacgtgttggaaganggccattatccttgtctgtctgcaatgggatgcccgcctggagcaactttcaagtctcttcgttttatagatgttgaattttctgaaccaacaattattctctttgaaagagaagacttcaaaggaaaaaagattgaacttaatgcagaaactgtcaatctccgatccctgggattcaacacacaaatacgctctgttcaggttattggtggcatatgggttacttatgaatatggcagttacagagggcgacagttcctattgtcacctgcagaagtacctaattggtatgaattcagtggctgtcgccaaataggttctctacgaccttttgttcagaagcgaatttatttcagacttcgaaacaaagcaacagggttattcatgtcaaccaatggaaacttagaggatctgaagcttctgaggatacaggtcatggaggatgtcggggccgatgatcagatttggatctatcaagaaggatgtatcaaatgcaggatagcagaagactgctgcctgacgattgtgggcagcctggtaacatctggctccaagctaggcctggccctggaccagaatgctgacagccagttctggagcttgaagtccgatggcaggatttacagcaagttgaagccaaatttagttttagacattaaagggggcacacagtatgatcaaaatcacattatcctcaacactgtcagcaaagagaagtttacacaagtgtgggaagccatggtcctatatacctgaacaaagaaggaagaagaatcttctggaggtccttccagccaccttatttcttaaaaaggacaatgctgatggaagaccagactggaaagtggatcgactcctccttcattgattctaaattcaaccttaaatcatgctgccatgactcagagaacttactcatcgtttcaaaagactatcatagctttaaaccaataatttgtcctcctttcatttcttgcctttcatttttggtagctgcttaaacaggttgcctaattagcagcttttgggtgattttgtaaaatgttatatcaagatttcaagactgtgtacattttaaattatttccaaagatagtgacaggagagaactggaacaaatttaccaactttgtggacctacaaagcccttacactttaaagggtaagacaaaggcttaagtttgaaaggtagagaactgtttagcatctgagaagaaatactttattaggcctgtaattttggttcttggccttaaacactttctggaacctttaaatatgctgcatagcacaatgggaaagccttaggtattcacacatttaaggaactctaaacaaaatactattttcctttagttcatattaaaaattaatacattttaaaaatttaatgtcaaagtctggtaacatttgttagtaggatttgagttattattttttgagacaggatctcaggctggagtgcagtggcacaatcacggctcactgcagcctctacctccccaggctcaggtgatcctcccacctcagcctcccaagtagctgggactataggcacacatcaccaagcccagccaaatttngttttttttttgtagagatggggtttcatcacgttgcccaggctgatctcgaacctctgggctcaagcaattcactcgcctcggcctcccaaaatgctgggattacaggcctgagccactgcgcccagccaggatttgaattattttaactcatccatgggctgccctagaatgtcacaaatgagggttgtttaatgcctttcttatagctgctactggaacactattatgacctaatttatgagccatccttactcatctacaagtgctgaagcaatgttacatacttttttgctaaactcagattttttagcctaatttcttgtcctcctatccacctgcatccacacatggcctgcatggggctgccttccctgcagtgttctgcagccatgcttcagggtatagctgttggtggacagcctcaggtcttgggggcactatagccactaaacgaggtgtgaaaggctcaagaggatgaccagcaattaattatccccagaaagtgaaggaaaagagacctttagggatgttgctggtcaagtcttgatttgaccggagtcaaatcaatcttcaagcaatcttggaatcctcaactgcagtaagcatttcaaaatgcaaacaaactgcttaacaactgacaagacaccagcccatangctgctcttccaacagtgggttctagctttgaacaaaagtgctaaacatttccttgaatatattcttcctctttttgtcctcatcactcaatactggtgctcttgtcacaggtagaacagcttgtttcttttccatctattcaagtgtgtttctaattctaaaatgctgatcttctctggagtctatggtaggcaattatggtcactggaatagtttgtcttgttttaaaatattattggtgcatgtacaacagcatccaacatatctgtcttgttcctagatatatagctctgattttaggccttttgtgcataccattacaatatggtggggtaagacattctacagtagcctgtgctgaactgatctcttaaataaacttgcttctggttaannannnnnaaaaaaaaaaaaaaaaaantcgacc

>MBNL2_probe1

TTCAGCCCTTTAATAATGGAGCATC

>MBNL2_probe2

TTTACTATGATATCCATTTTCCAGA

>MBNL2_probe3

GAGACTAACTCTCCACTTGTATGGG

>MBNL2_probe4

GGGAACTACATTTCACTCTTGGTTT

>MBNL2_probe5

ACCTGTAACCCCAAGCAAATATAGA

>MBNL2_probe6

AACTCTCCACTTGTATGGGAACTAC

>MBNL2_probe7

TCAGGATATAACAGCACTTCACCGA

>MBNL2_probe8

ATTTCACTCTTGGTTTTCAGGATAT

>MBNL2_probe9

TAACAGCACTTCACCGAAATATTCT

>MBNL2_probe10

TATTAGCACACAACTATTTTCAGCC

>MBNL2_probe11

AAGTTTGTTTATATTCAGAAGTCTG

>MBNL2_target

tattagcacacaactattttcagccctttaataatggagcatcaaaaacatcacctgtaaccccaagcaaatatagaagactgtattttttactatgatatccattttccagaatngtgattacaatatgcaaagagtcataaatatgccatttacaataaggaggaggcaaggcaaatgcatagatgtacaaatatatgtacaacagatttngctttttatttatttataatgtaattttatagaataattctgggatttgagaggatctaaaactatttttctgtataaatattatttgccaaaagtttgtttatattcagaagtctgactatgatgaataaatcttaaatgctttgtttaattaaaaaacaaaaatcaccaatatccaagacatgaagatatcagttcaacaaatactgtagttaagagactaactctccacttgtatgggaactacatttcactcttggttttcaggatataacagcacttcaccgaaatattct

>MBNL2_consensus

gcgnccgcggcacgagtcatgactcagtcgactgccaaagcaatgaagnnncntnnngnngcnncnnnagncntggcctttccccctggtgctcttcatcctttaccaaagagacaagcacttgaaaaaagcaatggtaccagcgcggtctttaaccccagcgtcttgcactaccagcaggctctcaccagcgcacagttgcagcaacacgccgcgttcattccaacagataattctgaaataatcagcagaaacggaatggaatgccaagaatctgcattgagaataactaaacattgttactgtacatactatcctgtttcctcctcaatagaattgccacaaactgcatgctaaataaagatgtagttcttctggacagaccacaactctaagaagctagtgctgctatctcatatatgagtattaaatatggtatgcttagtatattccaacctaagatagttaactacctgagaccagctgtgatgtttaaagacataaaggataaagtttacttttaaagggtttctaaacatagtttctgtcctaggaatattgtcttatctccataactatagctgatgcanaaagtccagccagtttactcatttcgattcagaatatttcaaatttagcaataaacaattagcattagttaaaaaagaaacatattccaagggcaggttcgattctagctctaattactgtcatgtcatttacccactggatcaaanggtatgtttcacttcttgacaatataaatgctgcagcaaagatgagaggtgaagtaaaaccgatacctgtcctgcaggtctaaaatttgaatggaaattcaagcacaagtactggggacacatcaaagtgtggtgtttggtttgcctggagatgccacgttgaatcatgtgattctagattaacattaaatagattgaaaaagaaactttgcacggtatgagcttcataccccaccaaacaaagtcttgaaggtattattttacaagtatatttttaaagttgttttataagagagactttgtagaagtgcctagattttgccagacttcatccagcttgacaagattgagaggcccatgccaacagtctaatctaagagattagtctttcaaactcaccatccagttgcctgttacagaataactcttcttaactaaaaacctagtcaaacaaggaagctgtaggtgaggagatctgtataatattctaatttaagtaagtttgagtttagtcactgcaaatttgactgtgactttaatctaaattactatgtaaacaaaaagtagatagtttcactttttaaaaaatccattactgttttgcatttcaaaagttggattaaagggttgtaactgactacagcatggaaaaaaatagttcttttaattctttcaccttaaagcatattttatgtctcaaaagtataaaaaactttaatacaagtacatacatattatatatacacatacatatatatactatatatggatgaaacatattttaatgttgtttacttttttaaatacttggttgatcttcaaggtaatagcgatacaattaaattttgttcagaaagtttgttttaaagtttattttaagcactatcgtaccaaatatttcatatttcacattttatatgttgcacatagcctatacagtacctacatagtttttaaattattgtttaaaaaacaaaacagctgttataaatgaatattatgtgtaattgtttcaaacatccattttctttgtgaacatattagtgattgaagtatttngacttttgagattgaatgtaaaatattttaaatttgggatcatcgcctgttctgaaaactagatgcaccaaccgtatcattattngtttgaggaaaaaaagaaatctgcattttaattcatgttggtcaaagtcgaattactatctatttatcttatatcgtagatctgataaccctatctaaaagaaagtcacacgctaaatgtattcttacatagtgcttgtatcgttgcattngttttaattngtggaaaagtattgtatctaacttgtattacttnggtagtttcatctttatgtattattgatatttgtaattttctcaactataacaatgtagttacgctacaactntgcctaaaacattcaaactntgttttcttttttctgtttttttctttgttaattcatttaaactcattgaaaacatagtatacattactaaaaggtaaattatgggaatcactgaaatattttngtagattaatngtngtaacatngtctttcttttttttctttngtttcatgatttngatttttaaaattattagcacacaactattttcagccctttaataatggagcatcaaaaacatcacctgtaaccccaagcaaatatagaagactgtattttttactatgatatccattttccagaatngtgattacaatatgcaaagagtcataaatatgccatttacaataaggaggaggcaaggcaaatgcatagatgtacaaatatatgtacaacagatttngctttttatttatttataatgtaattttatagaataattctgggatttgagaggatctaaaactatttttctgtataaatattatttgccaaaagtttgtttatattcagaagtctgactatgatgaataaatcttaaatgctttgtttaattaaaaaacaaaaatcaccaatatccaagacatgaagatatcagttcaacaaatactgtagttaagagactaactctccacttgtatgggaactacatttcactcttggttttcaggatataacagcacttcaccgaaatattctttcagccataccactggtaacatttctactaaatctttctgtaacacttaaagaattccctcattcattaccttacagtgtaaacaggagtctaatttgtatcaatactatgttttggttgtaatattcagttcactcacccaatgtacaaccaatgaaataaaagaagcatttaaaaggaaaaaaaaaaaaaaaaaaaaaaaaaaaa

>PPIF_probe1

GCTGAAGGCAGATGTCGTCCCAAAG

>PPIF_probe2

TGGCAAGCATGTTGTGTTCGGTCAC

>PPIF_probe3

GCCTGAAACGATACGTGTGCCCACT

>PPIF_probe4

TAATGCTGGTCCTAACACCAACGGC

>PPIF_probe5

CCGCTTTCCTGACGAGAACTTTACA

>PPIF_probe6

GTGGCCAGGGTGCTGGCATGGTGGC

>PPIF_probe7

AGAAGGGCTTCGGCTACAAAGGCTC

>PPIF_probe8

AAGTCCATCTACGGAAGCCGCTTTC

>PPIF_probe9

TGTCCTGTCCATGGCTAATGCTGGT

>PPIF_probe10

GTTCGGTCACGTCAAAGAGGGCATG

>PPIF_probe11

GACTGTGGCCAGTTGAGCTAATCTG

>PPIF_target

gctgaaggcagatgtcgtcccaaagacagctgagaacttcagagccctgtgcactggtgagaagggcttcggctacaaaggctccaccttccacagggtgatcccttccttcatgtgccaggcgggcgacttcaccaaccacaatggcacaggcgggaagtccatctacggaagccgctttcctgacgagaactttacactgaagcacgtggggccaggtgtcctgtccatggctaatgctggtcctaacaccaacggctcccagttcttcatctgcaccataaagacagactggttggatggcaagcatgttgtgttcggtcacgtcaaagagggcatggacgtcgtgaagaaaatagaatctttcggctctaagagtgggaggacatccaagaagattgtcatcacagactgtggccagttgagctaatctgtggccagggtgctggcatggtggcagctgcaaatgtccatgcacccaggtggccgcgttgggctgtcagccaaggtgcctgaaacgatacgtgtgcccact

>PPIF_exemplar

gaattccggagttccgggcgcgcgcgacgtcagtttgagttctgtgttctccccgcccgtgtcccgcccgacccgcgcccgcgatgctggcgctgcgctgcggctcccgctggctcggcctgctctccgtcccgcgctccgtgccgctgcgcctccccgcggcccgcgcctgcagcaagggctccggcgacccgtcctcttcctcctcctccgggaacccgctcgtgtacctggacgtggacgccaacgggaagccgctcggccgcgtggtgctggagctgaaggcagatgtcgtcccaaagacagctgagaacttcagagccctgtgcactggtgagaagggcttcggctacaaaggctccaccttccacagggtgatcccttccttcatgtgccaggcgggcgacttcaccaaccacaatggcacaggcgggaagtccatctacggaagccgctttcctgacgagaactttacactgaagcacgtggggccaggtgtcctgtccatggctaatgctggtcctaacaccaacggctcccagttcttcatctgcaccataaagacagactggttggatggcaagcatgttgtgttcggtcacgtcaaagagggcatggacgtcgtgaagaaaatagaatctttcggctctaagagtgggaggacatccaagaagattgtcatcacagactgtggccagttgagctaatctgtggccagggtgctggcatggtggcagctgcaaatgtccatgcacccaggtggccgcgttgggctgtcagccaaggtgcctgaaacgatacgtgtgcccactccactgtcacagtgtgcctgaggaaggctgctagggatgttagacggaattcc

>GINS2_probe1

TACAGCAGGAGTGGCCATGTGGTCC

>GINS2_probe2

GATGAGGTACTCGTGGTTCTGGAGC

>GINS2_probe3

GGCAGATGGTGCAGCCAACAATGCT

>GINS2_probe4

AACAATGCTGACCGGTGCTTATCCT

>GINS2_probe5

GGACATTCTTCAATTCCACATCTGT

>GINS2_probe6

GGGCTGAATTTAGACTCTCTCACAG

>GINS2_probe7

CAGCCTCTGGAGAGTACTCAGTCTC

>GINS2_probe8

AAATAAGTCATTCTCCCTAGCAGAG

>GINS2_probe9

CTCTAAGCCCTGATCCACAATAAAA

>GINS2_probe10

GGAGCTCTAGAAACACTTCTGATGC

>UBE2C_probe1

TATAAGCTCTCGCTAGAGTTCCCCA

>UBE2C_probe2

GAGCTCTGGAAAAACCCCACAGCTT

>UBE2C_probe3

GGAAAAGTGGTCTGCCCTGTATGAT

>UBE2C_probe4

GGGTAACATATGCCTGGACATCCTG

>UBE2C_probe5

CAATGCGCCCACAGTGAAGTTCCTC

>UBE2C_probe6

GGGTAGGGACCATCCATGGAGCAGC

>UBE2C_probe7

GCCTTCCCTGAATCAGACAACCTTT

>UBE2C_probe8

TCCATCCAGAGCCTTCTAGGAGAAC

>UBE2C_probe9

ATGATGTCAGGACCATTCTGCTCTC

>UBE2C_probe10

AGATGGTCTGTCCTTTTTGTGATTT

>UBE2C_probe11

TGATAGTCCCTTGAACACACATGCT

>UBE2C_target

gccttccctgaatcagacaaccttttcaaatgggtagggaccatccatggagcagctggaacagtatatgaagacctgaggtataagctctcgctagagttccccagtggctacccttacaatgcgcccacagtgaagttcctcacgccctgctatcaccccaacgtggacacccagggtaacatatgcctggacatcctgaaggaaaagtggtctgccctgtatgatgtcaggaccattctgctctccatccagagccttctaggagaacccaacattgatagtcccttgaacacacatgctgccgagctctggaaaaaccccacagcttttaagaagtacctgcaagaaacctactcaaagcaggtcaccagccaggagccctgacccaggctgcccagcctgtccttgtgtcgtctttttaatttttccttagatggtctgtcctttttgtgattt

>UBE2C_exemplar

ggcacgagcgagttcctgtctctctgccaacgccgcccggatggcttcccaaaaccgcgacccagccgccactagcgtcgccgccgcccgtaaaggagctgagccgagcgggggcgccgcccggggtccggtgggcaaaaggctacagcaggagctgatgaccctcatgatgtctggcgataaagggatttctgccttccctgaatcagacaaccttttcaaatgggtagggaccatccatggagcagctggaacagtatatgaagacctgaggtataagctctcgctagagttccccagtggctacccttacaatgcgcccacagtgaagttcctcacgccctgctatcaccccaacgtggacacccagggtaacatatgcctggacatcctgaaggaaaagtggtctgccctgtatgatgtcaggaccattctgctctccatccagagccttctaggagaacccaacattgatagtcccttgaacacacatgctgccgagctctggaaaaaccccacagcttttaagaagtacctgcaagaaacctactcaaagcaggtcaccagccaggagccctgacccaggctgcccagcctgtccttgtgtcgtctttttaatttttccttagatggtctgtcctttttgtgatttctgtataggactctttatcttgagctgtggtatttttgttttgtttttgtcttttaaattaagcctcggttgagcccttgtatattaaataaatgcatttttgtccttttttaaaaaaaaaaaaaaaaaaa

>TYMS_probe1

GAGGGTATCTGACAATGCTGAGGTT

>TYMS_probe2

GCATTTCAATCCCACGTACTTATAA

>TYMS_probe3

ATCTGTCCGTGACCTATCAGTTATT

>TYMS_probe4

GTACAATCCGCATCCAACTATTAAA

>TYMS_probe5

AAATGGCTGTTTAGGGTGCTTTCAA

>TYMS_probe6

TCACAAGCTATTCCCTCAAATCTGA

>TYMS_probe7

AACTGTGCCAGTTCTTTCCATAATA

>TYMS_probe8

GAGGGAGCTGAGTAACACCATCGAT

>TYMS_probe9

GGGGTTGGGCTGGATGCCGAGGTAA

>TYMS_probe10

AAAGCTCAGGATTCTTCGAAAAGTT

>TYMS_probe11

GAACTAGGTCAAAAATCTGTCCGTG

>NEK2_probe1

ATTAATACCATGACATCTTGCTTAT

>NEK2_probe2

GCTGTAGTGTTGAATACTTGGCCCC

>NEK2_probe3

GCCATGCCTTTCTGTATAGTACACA

>NEK2_probe4

TGAGCTGTCTGTCATTTACCTACTT

>NEK2_probe5

GTAGCACTCACTGAATAGTTTTAAA

>NEK2_probe6

TTGGTTGGGCTTTTAATCCTGTGTG

>NEK2_probe7

CTCTGTAGTTCAAATCTGTTAGCTT

>NEK2_probe8

GATATTTCGGAATTGGTTTTACTGT

>NEK2_probe9

AAATATTCCATTGCTCTGTAGTTCA

>NEK2_probe10

TGAATACTTGGCCCCATGAGCCATG

>NEK2_probe11

GGTATGCTTACAATTGTCATGTCTA

>NEK2_target

gctgtagtgttgaatacttggccccatgagccatgcctttctgtatagtacacatgatatttcggaattggttttactgttcttcagcaactattgtacaaaatgttcacatttaatttttctttcttcttttaagaacatattataaaaagaatactttcttggttgggcttttaatcctgtgtgtgattactagtaggaacatgagatgtgacattctaaatcttgggagaaaaaataatattaggaaaaaaatatttatgcaggaagagtagcactcactgaatagttttaaatgactgagtggtatgcttacaattgtcatgtctagatttaaattttaagtctgagattttaaatgtttttgagcttagaaaacccagttagatgcaatttggtcattaataccatgacatcttgcttataaatattccattgctctgtagttcaaatctgttagctttgtgaaaattcatcactgtgatgtttgtattctttttttttttctgtttaacagaatatgagctgtctgtcatttacctactt

>NEK2_exemplar

ggcacgagtaggggtggcgggtcagtgctgctcgggggcttctccatccaggtccctggagttcctggtccctggagctccgcacttggcgcgcaacctgcgtgaggcagcgcgactctggcgactggccggccatgccttcccgggctgaggactatgaagtgttgtacaccattggcacaggctcctacggccgctgccagaagatccggaggaagagtgatggcaagatattagtttggaaagaacttgactatggctccatgacagaagctgagaaacagatgcttgtttctgaagtgaatttgcttcgtgaactgaaacatccaaacatcgttcgttactatgatcggattattgaccggaccaatacaacactgtacattgtaatggaatattgtgaaggaggggatctggctagtgtaattacaaagggaaccaaggaaaggcaatacttagatgaagagtttgttcttcgagtgatgactcagttgactctggccctgaaggaatgccacagacgaagtgatggtggtcataccgtattgcatcgggatcttaaaccagccaatgttttcctggatggcaagcaaaacgtcaagcttggagactttgggctagctagaatattaaaccatgacacgagttttgcaaaaacatttgttggcacaccttattacatgtctcctgaacaaatgaatcgcatgtcctacaatgagaaatcagatatctggtcattgggctgcttgctgtatgagttatgtgcattaatgcctccatttacagcttttagccagaaagaactcgctgggaaaatcagagaaggcaaattcaggcgaattccataccgttactctgatgaattgaatgaaattattacgaggatgttaaacttaaaggattaccatcgaccttctgttgaagaaattcttgagaaccctttaatagcagatttggttgcagacgagcaaagaagaaatcttgagagaagagggcgacaattaggagagccagaaaaatcgcaggattccagccctgtattgagtgagctgaaactgaaggaaattcagttacaggagcgagagcgagctctcaaagcaagagaagaaagattggagcagaaagaacaggagctttgtgttcgtgagagactagcagaggacaaactggctagagcagaaaatctgttgaagaactacagcttgctaaaggaacggaagttcctgtctctggcaagtaatccagaacttcttaatcttccatcctcagtaattaagaagaaagttcatttcagtggggaaagtaaagagaacatcatgaggagtgagaattctgagagtcagctcacatctaagtccaagtgcaaggacctgaagaaaaggcttcacgctgcccagctgcgggctcaagccctgtcagatattgagaaaaattaccaactgaaaagcagacagatcctgggcatgcgctagccaggtagagagacacagagctgtgtacaggatgtaatattaccaacctttaaagactgatattcaaatgctgtagtgttgaatacttggccccatgagccatgcctttctgtatagtacacatgatatttcggaattggttttactgttcttcagcaactattgtacaaaatgttcacatttaatttttctttcttcttttaagaacatattataaaaagaatactttcttggttgggcttttaatcctgtgtgtgattactagtaggaacatgagatgtgacattctaaatcttgggagaaaaaataatattaggaaaaaaatatttatgcaggaagagtagcactcactgaatagttttaaatgactgagtggtatgcttacaattgtcatgtctagatttaaattttaagtctgagattttaaatgtttttgagcttagaaaacccagttagatgcaatttggtcattaataccatgacatcttgcttataaatattccattgctctgtagttcaaatctgttagctttgtgaaaattcatcactgtgatgtttgtattctttttttttttctgtttaacagaatatgagctgtctgtcatttacctacttctttcccactaaataaaagaattcttcagtta

>TXNRD1_probe1

ACCTGTATTTCTCAGTTGCAGCACT

>TXNRD1_probe2

CCCATGCATCTGCCTGGCATTTAGG

>TXNRD1_probe3

GCAATTGAGGCAGTTGACCATATTC

>TXNRD1_probe4

TCCTCATCTCATTTGGCTGTGTAAA

>TXNRD1_probe5

CCTGCCAGCAGTTCTTGAAGCTTCT

>TXNRD1_probe6

TCCAAGTCCACCAGTCTCTGAAATT

>TXNRD1_probe7

TGGCATTTAGGCAGCAGAGCCCCTG

>TXNRD1_probe8

GGAGTGGAATGTTCTATCCCCACAA

>MED24_probe1

CAGCCCAGGAGTAGTCTTACCTCTG

>MED24_probe2

CTTACCTCTGAGGAACTTTCTAGAT

>MED24_probe3

GGCTGCTAAAGCCATTGCTGCACTC

>MED24_probe4

GATGAGGCATCGTGCCTCACATCCG

>MED24_probe5

TAAAGCCATTGCTGCACTCTGAGGG

>MED24_probe6

GTGAGGGAAATCTACCTTCGTTCAT

>MED24_probe7

TGGTGCAAGAGCCTCTAGCGGCTTC

>MED24_probe8

TTCTTCTTTCAAAATTTCCTCTCCA

>MED24_probe9

AACTGGTGAAGGTGTCAGCCATGTC

>MED24_probe10

CATCCGCTCCACATGGTGCAAGAGC

>ELF1_probe1

AGATGACATGGTTGTTGCCCCAGTC

>ELF1_probe2

AAATATGCAGACTCACCGGGAGCCT

>ELF1_probe3

CATGTTCCTGGTGCTGATATTCTCA

>ELF1_probe4

TTATGCCGGTCTAGCCTGTGTGGAA

>ELF1_probe5

TCACCCATGTGTCCGTCACATTAGA

>ELF1_probe6

GATATTCTCAATAGTTATGCCGGTC

>ELF1_probe7

GATGAACGACAGCTTGGTGATCCAG

>ELF1_probe8

TTGGTGATCCAGCTATTTTTCCTGC

>ELF1_probe9

GGCACTCCTCAATATGGATTCCCCT

>ELF1_probe10

GCTGCCTGATACGTGAATCTTCTTG

>ELF1_probe11

GACATCACCCTTACAGTTGAAGCTT

>ELF1_target

gctgcctgatacgtgaatcttcttggaatttttctcatgtggatctaaggggaatgctttattatggctgctgttgtccaacagaacgacctagtatttgaatttgctagtaacgtcatggaggatgaacgacagcttggtgatccagctatttttcctgccgtaattgtggaacatgttcctggtgctgatattctcaatagttatgccggtctagcctgtgtggaagagcccantgacatgattactgagagttcactggatgttgctgaagaagaaatcatagacgatgatgatgatgacatcacccttacagttgaagcttcttgtcatgacggggatgaaacaattgaaactattgaggctgctgaggcactcctcaatatggattcccctggccctatgctggatgaaaaacgaataaataataatatatttagttcacctgaagatgacatggttgttgccccagtcacccatgtgtccgtcacattagatgggattcctgaagtgatggaaacacagcaggtgcaagaaaaatatgcagactcaccgggagcct

>ELF1_consensus

atctacaggagccattttaacagctaaaacttgtcggattgctttttattttcaagctcaaaagacgatagagaaagaatacttgaaggccaagaagcttgagagaagaaaaatttcagaaaaattgtctcaatttgactagaatatcaatgaaccaggaaaactgaagcaccttccctaaagaaaacttgggtatacaattactccacagacagagctgagggttttttacccaaatcagtcactggattttgctgcctgatacgtgaatcttcttggaatttttctcatgtggatctaaggggaatgctttattatggctgctgttgtccaacagaacgacctagtatttgaatttgctagtaacgtcatggaggatgaacgacagcttggtgatccagctatttttcctgccgtaattgtggaacatgttcctggtgctgatattctcaatagttatgccggtctagcctgtgtggaagagcccantgacatgattactgagagttcactggatgttgctgaagaagaaatcatagacgatgatgatgatgacatcacccttacagttgaagcttcttgtcatgacggggatgaaacaattgaaactattgaggctgctgaggcactcctcaatatggattcccctggccctatgctggatgaaaaacgaataaataataatatatttagttcacctgaagatgacatggttgttgccccagtcacccatgtgtccgtcacattagatgggattcctgaagtgatggaaacacagcaggtgcaagaaaaatatgcagactcaccgggagcctcatcaccagaacagcctaagaggaaaaaannaagaaaaactaaaccaccacgaccagattccccagccactacgccaaatatatctgtgaagaagaaaaacaaaganggaaagggaaacacaatttatctttgggagtttttactggcactgctccaggacaaggctacttgtcctaaatacatcaagtggacccagcgagagaaaggcatttttaaattggtggattctaaagcagtgtccaggttgtgggggaagcacaaaaacaaacctgatatgaattatgagaccatgggaagagcactcaggtactattaccaaaggggtattctggcaaaagtggaaggtcagcgcttggtgtatcagtttaaagaaatgccaaaagatcttatatatataaatgatgaggatccaagttccagcatagagtcttcagatccatcnctatcttcatcagccacttcaaataggaatcaaaccagccggtcgagagtatcttcaagtccaggggtaaaaggaggagccactncagttctaaaaccagggaattctaaagctgcaaaacccaaagatcctgtggaagttgcacaaccatcagaagttttgaggacagtgcagcccacgcagtctccatatcctacccagctcttccggactgttcatgtagtacagccagtacaggctgtcccagagggnagaagcagctagaaccagtaccatgcaggatgaaacattaaattcttccgttcagagtattaggactatacaggctccaacccaagttccagtggttgtgtctcctaggaatcagcagttgcatacagtaacactccaaacagtgccactcacaacagttatagccagcacagatccatcagcaggtactggatctcagaagtttattttacaagccattccatcatcacagcccatgacagtactgaaagaaaatgtcatgctgcagtcacaaaaggcgggctctcctccttcaattgtcttgggccctgccnaggttcagcaggtccttactagcaatgttcagaccatttgcaatggaaccgtcagtgtggcttcctctccatccttcagtgctactgcacctgtggtgaccttttctcctcgcagttcacagctggttgctcacccacctggcactgtaatcacttcagttatcaaaactcaagaaacaaaaactcttacacaggaagtagagaaaaaggaatctgaagatcatttgaaagagaacactgagaaaacggagcagcagccacagccttatgtgatggtagtgtccagttccaatggatttacttctcaggtagctatgaaacaaaacgaactgctggaacccaactctttttagttaatataccaaagcttatgaataattgtttgttaattgaacattttcaattatatgcagactgactgattctaagataaattctaaggaggtttctaattttgtaattgttaaaaatagagttaattttgactttgttagatgagggaggaaaactcaactgtttctctttgttatctaaatgtttcagaattcaatcgtgaaggaacaggcattttacactatgaagacattcttttgagatttttatttcagttgctatatcataagcatttttaaagtttcttttctaattttacattgtattagattttctgattcttttgtaaatacagaacttaaatagaaggcaacaggaaatttatataggaactattttcattccacttgtgtaagttaagtcttgactctttcaaatgcaaaaaacctattttatgctttgttaaaattatggtgtcacttagattgactttagttgactgcactatataatatagaactatgaatatgtagaataacatgaaaaattggaggtgctggtggtatggctgaccctgtttcagaagcaggatagtataaaagcatcagcctaagaatggcactcccactaactagctatgtaatcttgacctctttgggctttagttcctctcataaaaggaagagatgtattggantagactagatgatcaccactttctcttctagttctaatttttttaattctaatacctatattttcaagttatgtcaattaaatcattatcaggttatttcctaatgtaagaatagctaaaatgttgcagagaaataagtgacccaacaaaatttattcatctnttatgggtaagatctgccataaattcttcctaaataatttgtttactaactctttaggccactgtgctttgcggtccattagtaaacttgtgttgctaagtgctaaacagaatactgctattttgagagagtcaagactctttcttaagggccaagaaagcaacttgagccttgggctaatctggctgagtagtcagttataaaagcataattgctttatattttggatcattttttactgggggcggacttggggggggttgcatacaaagataacatatatatccaactttctgaaatgaaatgtttttagattactttttcaactgtaaataatgtacatttaatgtcacaagaaaaaaatgtcttctgcaaattttctagtataacagaaatttttgtagatgaaaaaaatcattatgtttagaggtctaatgctatgttttcatattacagagtgaatttgtatttaaacaaaaatttaaattttggaatcctctaaacatttttgtatctttaattggtttattattaaataaatcatataaaaattnnnaaaaaaaaaaaaaaaaaaaaaaaaaaaaaaaaaaaaaaaaaaaaaaaaaaaaaaa

>APH1A_probe1

GTGCATGTTTGGGAACTGGCATTAC

>APH1A_probe2

TTCTCAGTACTCCCTCAAGACTGGA

>APH1A_probe3

ACTCCAGAGCTGCAGTGCCACTGGA

>APH1A_probe4

GTGCCACTGGAGGAGTCAGACTACC

>APH1A_probe5

ATAGATGAGCTCTGAGTTTCTCAGT

>APH1A_probe6

GTCAGACTACCATGACATCGTAGGG

>APH1A_probe7

TCTTCTAACCTCCTTGGGCTATATT

>APH1A_probe8

CAGGCCTGAGGGGGAACCATTTTTG

>APH1A_probe9

GGACATCTTGGTCTTTTTCTCAGGC

>APH1A_probe10

TGCTGAGGGTGGAGTGTCCCATCCT

>APH1A_probe11

GAGGTATATTGGAACTCTTCTAACC

>APH1A_target

atagatgagctctgagtttctcagtactccctcaagactggacatcttggtctttttctcaggcctgagggggaaccatttttggtgtgataaataccctaaactgcctttttttcttttttgaggtggggggagggaggaggtatattggaactcttctaacctccttgggctatattttctctcctcgagttgctcctcatggctgggctcatttcggtccctttctccttggtcccagaccttgggggaaaggaaggaagtgcatgtttgggaactggcattactggaactaatggttttaacctccttaaccaccagcatccctcctctccccaaggtgaagtggagggtgctgtggtgagctggccactccagagctgcagtgccactggaggagtcagactaccatgacatcgtagggaaggaggggagatttttttgtagtttttaattggggtgtgggaggggcggggaggttttctataaactgtatcattttctgctgagggtggagtgtcccatcct

>APH1A_exemplar

gtggggtcgcgttgccaccccacgcggactccccagctggcgcgcccctcccatttgcctgtcctggtcaggcccccaccccccttcccacctgaccagccatgggggctgcggtgtttttcggctgcactttcgtcgcgttcggcccggccttcgcgcttttcttgatcactgtggctggggacccgcttcgcgttatcatcctggtcgcaggggcatttttctggctggtctccctgctcctggcctctgtggtctggttcatcttggtccatgtgaccgaccggtcagatgcccggctccagtacggcctcctgatttttggtgctgctgtctctgtccttctacaggaggtgttccgctttgcctactacaagctgcttaagaaggcagatgaggggttagcatcgctgagtgaggacggaagatcacccatctccatccgccagatggcctatgtttctggtctctccttcggtatcatcagtggtgtcttctctgttatcaatattttggctgatgcacttgggccaggtgtggttgggatccatggagactcaccctattacttcctgacttcagcctttctgacagcagccattatcctgctccataccttttggggagttgtgttctttgatgcctgtgagaggagacggtactgggctttgggcctggtggttgggagtcacctactgacatcgggactgacattcctgaacccctggtatgaggccagcctgctgcccatctatgcagtcactgtttccatggggctctgggccttcatcacagctggagggtccctccgaagtattcagcgcagctcttgtgtaaggactgactacctggactgatcgcctgacagatcccacctgcctgtccactgcccatgactgagcccagccccagcccggggtccattgcccacattctctgtctccttctcgtcggtctaccccactacctccagggttttgctttgtccttttgtgaccgttagtctctaagctttaccaggagcagcctgggttcagccagtcagtgactggtgggtttgaatctgcacttatccccaccacctggggacccccttgttgtgtccaggactccccctgtgtcagtgctctgctctcaccctgcccaagactcacctcccttcccctctgcaggccgacggcaggaggacagtcgggtgatggtgtattctgccctgcgcatcccacccgaggactgagggaacctaggggggacccctgggcctggggtgccctcctgatgtcctcgccctgtatttctccatctccagttctggacagtgcaggttgccaagaaaagggacctagtttagccattgccctggagatgaaattaatggaggctcaaggatagatgagctctgagtttctcagtactccctcaagactggacatcttggtctttttctcaggcctgagggggaaccatttttggtgtgataaataccctaaactgcctttttttcttttttgaggtggggggagggaggaggtatattggaactcttctaacctccttgggctatattttctctcctcgagttgctcctcatggctgggctcatttcggtccctttctccttggtcccagaccttgggggaaaggaaggaagtgcatgtttgggaactggcattactggaactaatggttttaacctccttaaccaccagcatccctcctctccccaaggtgaagtggagggtgctgtggtgagctggccactccagagctgcagtgccactggaggagtcagactaccatgacatcgtagggaaggaggggagatttttttgtagtttttaattggggtgtgggaggggcggggaggttttctataaactgtatcattttctgctgagggtggagtgtcccatccttttaatcaaggtgattgtgattttgactaataaaaaagaatttgtaaaaaa

>SLC11A2_probe1

ACTGACCATACATTTTTCTTAGCCC

>SLC11A2_probe2

GACATTTTTACATACCGAGCCTGAG

>SLC11A2_probe3

TTCATCTGAGCCCCCAAAAGCATTA

>SLC11A2_probe4

TTCTTAGCCCCTCAAGTAATATAGC

>SLC11A2_probe5

AAACTGGTCATAAAGGCACTCTGTG

>SLC11A2_probe6

CCTTCCAGAGTCCTGGCTGATTGGT

>SLC11A2_probe7

GGGACTGACATCTTAAGCTCTCACC

>SLC11A2_probe8

TACACTACTGTGTTTCACTGACCAT

>SLC11A2_probe9

TGATTGGTGTTCGCTGTTCATCTGA

>SLC11A2_probe10

GGACTTCTCATTTTTGGAGCTTTCC

>SLC11A2_probe11

GAATGACAATTCCCCTAACCATTCC

>SLC11A2_target

gacatttttacataccgagcctgagattntgtgattatctcaaatcaaatcactttgatggagataaataatcaaaactgttttatagtcattgatttggtgagaacagtaatggaaaatggtgttgaaggacttctcatttttggagctttccttccagagtcctggctgattggtgttcgctgttcatctgagcccccaaaagcattattactgatacttgcacacagtcaaaagcgcagactggatggatggtcttttataaggcatttaagggtacactactgtgtttcactgaccatacatttttcttagcccctcaagtaatatagcacagagttatgaatgacaattcccctaaccattcctcttcatatctgcctcttccccttaccatcgtaattctccaaactggtcataaaggcactctgtgaagatattggggactgacatcttaagctctcacc

>SLC11A2_consensus

agcnngnangnnggcnccctgaggaatatggagccctgaaggcgcggnggnggcgngnannngnnnnnncnnngnnnnnnanaatcatattctaagaactcagccactcaggtatccaccatggtgctgggtcctgaacagaagatgtcagatgacagtgtttctggagatcatggggagtctgccagtcttggtaacatcaaccctgcctatagtaatccctctctttcacagtcccctggggactcagaggagtacttcgccacttactttaatgagaagatctccattcctgaggaggagtactcttgttttagctttcgtaaactctgggctttcaccggaccaggttttcttatgagcattgcctacctggatccaggaaatattgaatccgatttgcagtctggagcagtggctggatttaagttgctctggatccttctgttggccacccttgtggggctgctgctccagcggcttgcagctagactgggagtggttactgggctgcatcttgctgaagtatgtcaccgtcagtatcccaaggtcccacgagtcatcctgtggctgatggtggagttggctatcatcggctcagacatgcaagaagtcattggctcagccattgctatcaatcttctgtctgtaggaagaattcctctgtggggtggcgttctcatcaccattgcagatacttttgtatttctcttcttggacaaatatggcttgcggaagctagaagcattttttggctttctcatcactattatggccctcacatttggatatgagtatgttacagtgaaacccagccagagccaggtactcaagggcatgttcgtaccatcctgttcaggctgtcgcactccacagattgaacaggctgtgggcatcgtgggagctgtcatcatgccacacaacatgtacctgcattctgccttagtcaagtctagacaggtaaaccggaacaataagcaggaagttcgagaagccaataagtactttttcattgaatcctgcattgcactctttgtttccttcatcatcaatgtctttgttgtctcagtctttgctgaagcattttttgggaaaaccaacgagcaggtggttgaagtctgtacaaataccagcagtcctcatgctggcctctttcctaaagataactcgacactggctgtggacatctacaaagggggtgttgtgctgggatgttactttgggcctgctgcactctacatttgggcagtggggatcctggctgcaggacagagctccaccatgacaggaacctattctggccagtttgtcatggagggattcctgaacctaaagtggtcacgctttgcccgagtggttctgactcgctctattgccatcatccccactctgcttgttgctgtcttccaagatgtagagcatctaacagggatgaatgactttctgaatgttctacagagcttacagcttccctttgctctcatacccatcctcacatttacgagcttgcggccagtaatgagtgactttgccaatggactaggctggcggattgcaggaggaatcttggtccttatcatctgttccatcaatatgtactttgtagtggtttatgtccgggacctagggcatgtggcattatatgtggtggctgctgtggtcagcgtggcttatctgggctttgtgttctacttgggttggcaatgtttgattgcactgggcatgtccttcctggactgtgggcatacggtaagcatctctaaaggcctgctgacagaagaagccacccgtggctacgttaaataacactggattagtctgtcttctgcaggtagccatcagagccagtgtgtttctatggtttactgtgtgaacatagccaaaagtatgtgccgttgcacagactgtgtttatgactcaaccgttggttggaaaagactttgtttcatgtgtatttgaaagatggaattattttttccttcctgacctaaccttagaactggattagggtgggatctttgaaaagctgacatttgctgctatcattccaacactaaattcttaagtagttgcccaagggccagctcagtttatccttcggagagacaaggatatgcatgattcttaaccaggctatatgttaaaaaaaaattggaaaatgcaatacattttttattatacaaactacagaatgantatgcaagttttatttatcaaaatgtaatggatttttaaaggctgagaaattttccttatacctaccttttcagttattttaattataccaaattatcaactagaatagcttcatccatatgaaatataaaatgaagagacacctagnctctatcaggcttaggattctttgaacttatttccactttaatttctcagtggaagttaagaggggtgagaaaacaaagaaggggaaaaactgacaactaacaaaaccagcaccacatcgctaggtggtgcttactaattaccttctcaggattttcctcagattgaaaagcttatgaggatttcttgggagtcttaataacctgcctgttagtacagagctttcctgatgatatttactcttgagcacatgtggttgtaaaaccttaactttctttctccaggagggtggtgatagaaacagatggtagtatttatgaactgatgttctcgtgaaatgttgagggtggggagaaaagactttaagggaggagagccatctattttgttcctaaagccacctctcagcagaatcgtcatgtttttctgatgcaccgctctgcttcatgcccaagatgacttgcgaggcaatctcaggagctgtggacttaaccattgcaaagcacactgtctttctcagcgttctctgcaagtcagtaggtgttagtatggttgcaaagttcactgtctcagcaaagttgaactgggctacctctctacagctgtttcctcagagggaaaaatcttgagaccagatggtggagctctggagtcagaggaaatgggtgtcttcagcacaaagctgctgcttttacttcagccacttctgacatttttacataccgagcctgagattntgtgattatctcaaatcaaatcactttgatggagataaataatcaaaactgttttatagtcattgatttggtgagaacagtaatggaaaatggtgttgaaggacttctcatttttggagctttccttccagagtcctggctgattggtgttcgctgttcatctgagcccccaaaagcattattactgatacttgcacacagtcaaaagcgcagactggatggatggtcttttataaggcatttaagggtacactactgtgtttcactgaccatacatttttcttagcccctcaagtaatatagcacagagttatgaatgacaattcccctaaccattcctcttcatatctgcctcttccccttaccatcgtaattctccaaactggtcataaaggcactctgtgaagatattggggactgacatcttaagctctcacctggctgcagtaggaaaggccaaactgacgacaaaaaaaaaattctttataaagatgatatggtaacatgtatctttgccctgggtctgggtgggtccagtcagtctcagatttacaagcatttaggagcctaggtaaaagctgctagtattcttttaaaagttanatttatgacttgcaatgatagaaaactccttccaattaaatggcattttataatattatgtgtgtacttcacagtgttaaaaataccctcatacgttattgcatttgatcttcacagaaagtgcattttaaccagtactctgggtgcaataaataatatgtagaaatttaagtcctccaattccagcatatccagtgagttttgacagtgtgtttatgtggaatgtttaaggatatacaattgtactttatataaattggttcttgttcttcttaaatgtgacatgaaataattgtgctgctacattatactggaaattaacaggggaaaagggaagagctcttggctcccttgaggttctgctagtggtgttaggagtggttacaactgagcttttagtaaccatttaaccgtatgtaaacttggtttctaattaaaaaaaaatttctttttccaaaaaaaaaaaaannnnnctaattaaaaaaaaatttctttttccaaaaaaa

>CCNB1_probe1

TTTGCACTTCCTTCGGAGAGCATCT

>CCNB1_probe2

CTTCCAGTTATGCAGCACCTGGCTA

>CCNB1_probe3

CAACATTACCTGTCATATACTGAAG

>CCNB1_probe4

TGGACACCAACTCTACAACATTACC

>CCNB1_probe5

TGGCACCATGTGCCATCTGTACATA

>CCNB1_probe6

ACTATGACATGGTGCACTTTCCTCC

>CCNB1_probe7

GGAACTAACTATGTTGGACTATGAC

>CCNB1_probe8

GAATCTCTTCTTCCAGTTATGCAGC

>CCNB1_probe9

GCACCTGGCTAAGAATGTAGTCATG

>CCNB1_probe10

TCCTCCTTCTCAAATTGCAGCAGGA

>CCNB1_probe11

AGATTGGAGAGGTTGATGTCGAGCA

>CCNB1_target

tttgcacttccttcggagagcatctaagattggagaggttgatgtcgagcaacatactttngccaaatacctgatggaactaactatgttggactatgacatggtgcactttcctccttctcaaattgcagcaggagctttttgcttagcactgaaaattctggataatggtgaatggacaccaactctacaacattacctgtcatatactgaagaatctcttcttccagttatgcagcacctggctaagaatgtagtcatggtaaatcaaggacttacaaagcacatgactgtcaagaacaagtatgccacatcgaagcatgctaagatcagcactctaccacagctgaattctgcactagttcaagatttagccaaggctgtggcaaaggtgtaacttgtaaacttgagttggagtactatatttacaaataaaattggcaccatgtgccatctgtacata

>CCNB1_consensus

cgccantnggnagngngtgagtgccacgaacaggccaataaggagggagcagtgcgggnntttaaatctgaggctaggctggctcttctcggcgtgctgcggcggaacggctgttggtttctgctgggtgtaggtccttggctggtcgggcctccggtgttctgcttctccccgctgagctgctgcctggtgaagaggaagccatggcgctccgagtcaccaggaactcgaaaattaatgctgaaaataaggcgaagatcaacatggcaggcgcaaagcgcgtncctacggcccctgctgcaacctccaagcccggactgaggccaagaacagctcttggggacattggtaacaaagtcagtgaacaactgcaggccaaaatgcctatgaagaaggaagcaaaaccttcagctactggaaaagtcattgataaaaaactaccaaaacntcttgaaaaggtacctatgctggtgccagtgccagtgtctgagccagtgccagagccagaacctgagccagaacctgagcctgttaaagaagaaaaactttcgcctgagcctatttnggttgatactgcctctccaagcccaatggaaacatctggatgtgcccctgcagaagaagacctgtgtcaggctttctctgatgtaattcttgcagtaaatgatgtggatgcagaagatggagctgatccaaaccttngtagtgaatatgtgaaagatatttatgcttatctgagacaacttgaggaagagcaagcagtcagaccaaaatacctactgggtcgggaagtcactggaaacatgagagccatcctaattgactggctagtacaggttcaaatgaaattcaggttgttgcaggagaccatgtacatgactgtctccattattgatcggttcatgcagaataattgtgtgcccaagaagatgctgcagctggttggtgtcactgcnatgtttattgcaancaaatatgaagaaatgtaccctncagaaattggtgactttgctttngtgactgacnaacancttnatactaagcaccaaatcagacagatggaaatgaagattctaagagctttaaactntggtctgggtcggcctctacctttgcacttccttcggagagcatctaagattggagaggttgatgtcgagcaacatactttngccaaatacctgatggaactaactatgttggactatgacatggtgcactttcctccttctcaaattgcagcaggagctttttgcttagcactgaaaattctggataatggtgaatggacaccaactctacaacattacctgtcatatactgaagaatctcttcttccagttatgcagcacctggctaagaatgtagtcatggtaaatcaaggacttacaaagcacatgactgtcaagaacaagtatgccacatcgaagcatgctaagatcagcactctaccacagctgaattctgcactagttcaagatttagccaaggctgtggcaaaggtgtaacttgtaaacttgagttggagtactatatttacaaataaaattggcaccatgtgccatctgtacatannanngttgcatttacttttaataaagcttgtggccccttnnanannannnnnnnnnnannnaagaatctcttcttccagttatgcagcactggctaagaatgt

>TMEM97_probe1

ATTCCATCAGCTTTCTCTAAGTCTT

>TMEM97_probe2

ATAGAGGGTCTCTTCACGTTGATGC

>TMEM97_probe3

ATCCACTGTGTGCATAGAGGGTCTC

>TMEM97_probe4

TCAGCTTTCTCTAAGTCTTTGCTCA

>TMEM97_probe5

AAGTTCAACCTTAAAATGATGTTAG

>TMEM97_probe6

TCTCTTCACGTTGATGCTTGGCATT

>TMEM97_probe7

GCCAGGCATAACATATCCACTGTGT

>TMEM97_probe8

TCACGTTGATGCTTGGCATTCCATC

>TMEM97_probe9

GTGTGCATAGAGGGTCTCTTCACGT

>TMEM97_probe10

TACAGCCAGGCATAACATATCCACT

>TMEM97_probe11

CCATCAGCTTTCTCTAAGTCTTTGC

>TMEM97_target

tacagccaggcataacatatccactgtgtgcatagagggtctcttcacgttgatgcttggcattccatcagctttctctaagtctttgctcaagttcaaccttaaaatgatgttag

>TMEM97_consensus

gtaaattcacaaagctttggaaggagaagcaataaatttttgttttcaaatggcttgatgtaccttttttcctgttgctcttgaaatatgtttaactcctcatgagagaaccctggnttntntntnnnntttttttttttttnnnttntttnntggtcagcagctacctttatttggatcacacacgtgagtcagacagtaccactaatcgggccaatatggcttgtgagcaaaatgggtgaaaatagagggaactgactgggacctgttgtctaacatcattttaaggttgaacttgagcaaagacttagagaaagctgatggaatgccaagcatcaacgtgaagagaccctctatgcacacagtggatatgttatgcctggctgtagccaccttctcttctgaaaacaagatgggaatgacggaactgtattaaaagattaagttatccactagggagcatactagcacacttccagatcagccttgagatgctagacttcagatacagggacttgtgtcaggtggcctctagaaatgtggaagactaagatttagaaataaactaatttaaaagcaaacaaacaaaaaaaanctttcgcctaggatgatctgtttacaacagaaacaaaaacattttccagtttaatccatttgactgaaagtcattgttgggctgacccttaaggaaaacttaagtttaaacttcctttgcatagtatgttttctcttttttccttgggttagaggtgggaacaagaaaacattgatatagttttgaattaacattaaagaggcaaaatttcacatttaaaatggcccaaaatatcccactattcattttgctgacaggtgatacactcttgagaatcaggagtggagggtttagtcataagcatatatgctgtgggtcaggtgactcatgaacatgtcaaacaggccatgttctttagcagtttctggtctggtcctgcttttagtaatcttgcggtggaactcaggcacacagttatttggtaccacttccttataacctagcccacacccctttgcccaccctcaagccgatcggtctgttcatcccagtgcccttagaaggtgaaggtttgggctgggcccagtggttcacgtctgtaatcccagcacttagggagactgaggtgggcagatcacttgagcctaggcgttcgagaccannacctgggcaacatggcnaaanccctgtctccacnancacacnnnaaaaaaannnnaaacnnaattagccatacgtgttggtacacaccntgtagttctagctacttgggaggctaaggaagatggcttgagcccnnggaggcggaggttgcagtgagccgagatcatgccnctgccctttacntccnnnncntgggcnnancagagtgagactgtcttnnaaaaaaaaaaaaaaatggaagaaggtgagggtttgacatggttcctgacaccatcttgctcttgtgcattgctgccagtgtttcaaatgctgctgaagacgtgggttctgagcagtgttccttgtattgtatccaacaagcaaccaccctgtaggcatctctaccntgggccagtggttgtttccttcattttttttttcttttctcttcatacttgtagtaggggctccgcaacatgaaaattaaaagtatgaatgggatgagtaagtagggggcatagacagacacaagggttaaccgttcatgcaaagtctcaggtctttgtcccttgaaaccactggctttggagaaatcctcaaacagaaatgtggagagtatcggaattaaggttgtcatggtgtgaacagagtagatgattgcaggagttcgaatccacttgcagcttcctttgaggaaggcatacgttgcaatgggaaagaaaggcagctgaaacacaagctcgcaaaacagaaaggacttaaaccaggctgggggctcctgtagcagtgggtctttgaactccttagcataccacttcagcaggtttctaaactcgactgggtagagctcgcgcggcagcaccgcctgcaggtccatgaacagggtgatggggatgtggctgaggaagtagaggcccagcagccactccacgcagcgcctggttgccggagcccccatagtctgtcggttgggcctggacccgctgatgtgagaagaggggccaaatccgcgcgccttcccnnnnccnnnnncctcggtcgccgccttgcccccgcgagggctaggcagaccgcttccattggctggcgagaaga

>MLF1IP_probe1

AGGCCATAATCATCTTTTCTGGTTA

>MLF1IP_probe2

AAATAGCATCAGTTTGTCCAATAGT

>MLF1IP_probe3

ATGTTGACACCTTAATCGGTCCCAG

>MLF1IP_probe4

GAAGCTCCTTGACCAGGGATGAGAA

>MLF1IP_probe5

CAACCATCAGTTAGAGAAGCTCCTT

>MLF1IP_probe6

AAGAACACTTCTGGGAGCCGAAAGC

>MLF1IP_probe7

GTGCCTATAGGAAGACTAGTCTCAT

>MLF1IP_probe8

GCCATCTGCGAAATATCAACCATCA

>MLF1IP_probe9

AAACGTATGATTCATCCAGCCTTCC

>MLF1IP_probe10

ATCGGTCCCAGGTATGAGCTATAAT

>MLF1IP_probe11

GGAGCCGAAAGCCATCTGCGAAATA

>MLF1IP_target

aaacgtatgattcatccagccttccagctctgttatttaaagcaagaacacttctgggagccgaaagccatctgcgaaatatcaaccatcagttagagaagctccttgaccagggatgagaagagcagtctactaaaatgtgcctataggaagactagtctcatgctgttaccttctgaaactgtacctttataaatcaattgttttgcaaagaagttatggcctacttagaatctaaaatttgttattcaaattaaatggctgtgaacaatgttaaatagcatcagtttgtccaatagttttaaaggccataatcatcttttctggttaatatcttgagtaattttaaaatgttgacaccttaatcggtcccaggtatgagctataat

>MLF1IP_exemplar

gttgacgtgttcgactttcctgataattctgatgtctcaagcattggcaggctgggtgaaaatgagaaagatgaagaaacttatgagacctttgatcctcctttacatagcacagctatatatgctgatgaagaagaattctccaaacattgtggactgtctctctcttcaactcctccaggaaaagaagcaaaaagaagttcagacacttctggaaatgaagcaagtgaaatcgaatctgtaaaaattagtgcaaaaaagccaggaagaaagctcaggcccattagtgatgactctgaaagcattgaagaaagtgatacaaggagaaaagttaaatcagcagagaaaataagtacacaacgtcatgaggttattcgaaccacagcgtcttcagaactttcagagaaaccagctgagtctgtcacttctaaaaagacaggaccccttagtgcccagccctctgttgaaaaagagaacttggcaatagaaagtcaatcgaaaactcagaaaaaagggaagatatctcatgacaaaaggaagaaatcaagaagtaaagccataggctcagatacttctgacattgtgcacatttggtgtccagaaggaatgaaaaccagtgacatcaaggagttgaatattgttttgcctgaatttgagaaaacccacctagagcatcaacaaagaatagaatctaaagtttgtaaggcagccatcgccacattttatgttaatgttaaagaacaattcatcaaaatgcttaaagaaagccagatgttgacaaatctgaaaaggaagaatgctaagatgatttcagatatcgaaaagaaaaggcagcgtatgattgaagtccaggatgaactgcttcggttagagccacagctgaaacaactacaaacaaaatatgatgaacttaaagagagaaagtcctcccttaggaatgcagcatatttcttatctaatttaaaacagctttatcaagattattcagatgttcaagctcaagaaccaaacgtaaaggaaacgtatgattcatccagccttccagctctgttatttaaagcaagaacacttctgggagccgaaagccatctgcgaaatatcaaccatcagttagagaagctccttgaccagggatgagaagagcagtctactaaaatgtgcctataggaagactagtctcatgctgttaccttctgaaactgtacctttataaatcaattgttttgcaaagaagttatggcctacttagaatctaaaatttgttattcaaattaaatggctgtgaacaatgttaaatagcatcagtttgtccaatagttttaaaggccataatcatcttttctggttaatatcttgagtaattttaaaatgttgacaccttaatcggtcccaggtatgagctataataaacttgtaaaattaaaaaaaaaaaaaaaaaaaaaaaaaaaaaaaaaaaaaaaaaaaaaaaaaaaaaaaaaaaaa

>ECT2_probe1

AGACTGTTTGTACCCTTCATGAAAT

>ECT2_probe2

TCACAATAGCCTTTTTATAGTCAGT

>ECT2_probe3

GTAAATGACTCTTTGCTACATTTTA

>ECT2_probe4

GCATGTTCAACTTTTTATTGTGGTC

>ECT2_probe5

GGTAATTTTATCCACTAGCAAATCT

>ECT2_probe6

GCATAGATATGCGCATGTTCAACTT

>ECT2_probe7

GAAGTTGCCATCAGTTTTACTAATC

>ECT2_probe8

CAGTTTTACTAATCTTCTGTGAAAT

>ECT2_probe9

TAGCTGTTTCAGAGAGAGTACGGTA

>ECT2_probe10

TTCCTATTTCTTTAGGGAGTGCTAC

>ECT2_probe11

GTATGTGCCACTTCTGAGAGTAGTA

>RAE1_probe1

AGCCTTGTTGGGTTGTCAGCCATGG

>RAE1_probe2

AACAGTTAGATCAGCCCATCTCAGC

>RAE1_probe3

ATGGCACCCTTGCAACTGTGGGATC

>RAE1_probe4

AGAAGTAGTGGCTGGAGACTCTGGC

>RAE1_probe5

CTGCCTCATCTCTGTACGAATTTGG

>RAE1_probe6

GATGGTAGATTCAGCTTCTGGGACA

>RAE1_probe7

CTCAGCTTGCTGTTTCAATCACAAT

>RAE1_probe8

AATGGAATCGCGTTCCATCCTGTTC

>RAE1_probe9

TGGATTTCAACCCCTGGAGAAAACG

>RAE1_probe10

ACGAATTTGGGTCCCAGCCTTGTTG

>RAE1_probe11

CATATTTGCATACGCTTCCAGCTAC

>RAE1_target

aatggaatcgcgttccatcctgttcatggcacccttgcaactgtgggatctgatggtagattcagcttctgggacaaagatgccagaacaaaactaaaaacttcggaacagttagatcagcccatctcagcttgctgtttcaatcacaatggaaacatatttgcatacgcttccagctacgactggtcaaagggacatgaattttataatccccagaaaaaaaattacattttcctgcgtaatgcagccgaagagctaaagcccaggaataagaagtagtggctggagactctggctcagcgttgtttctctccactctgcctcatctctgtacgaatttgggtcccagccttgttgggttgtcagccatggacatggatttcaacccctggagaaaacg

>RAE1_exemplar

ttggtaccgagctcggatccactagtaacggccgccagtgtgctggaattcggcttctggttcggcccacctctgaaggttccagatcgatagtgaattcgtggttttttccgcggtagtcagggcagtttctacgcaggcttaaggaggcttcgggctcctaggatttctgtccgcgctcctggccctcgtccttcgcgccagagcaggttcgcaaactcctcagacccttctgctcccggccgccgctttccgccgggggcagacccccaggtccagaatgagcctgtttggaacaacctcaggccttggaaccagtgggaccagcatgtttggcagtgcaactacagacaatcacaatcccatgaagggtattgaagtaacatcatctcctgatgatagcattggttgtctgtcttttagcccaccaaccttgccggggaactctcttattgcaggatcatgggctaatgatgttcgctgctgggaagttcaagacagtggacagaccattccaaaagcccagcagatgcacactgggcctgtgcttgatgtctgctggagtgacgatgggagcaaagtgtttacggcatcgtgtgataaaactgccaaaatgtgggacctcagcagtaaccaagcgatacagatcgcacagcatgatgctcctgttaaaaccatccattggatcaaagctccaaactacagctgtgtgatgactgggagctgggataagactttaaagttttgggataaccgatcgtcaaatcctatgatggttttgcaactccctgaaaggtgttactgtgctgacgtgatataccccatggctgtggtggcaactgcagagaggggcctgattgtctatcagctagagaatcaaccttctgaattcaggaggatagaatctccactgaaacatcagcatcggtgtgtggctatttttaaagacaaacagaacaagcctactggttttgccctgggtagtatcgaggggagagttgctattcactatatcaaccccccgaaccccgccaaagataacttcacctttaaatgtcatcgatctaatggaaccaacacttcagctcctcaggacatttatgcggtaaatggaatcgcgttccatcctgttcatggcacccttgcaactgtgggatctgatggtagattcagcttctgggacaaagatgccagaacaaaactaaaaacttcggaacagttagatcagcccatctcagcttgctgtttcaatcacaatggaaacatatttgcatacgcttccagctacgactggtcaaagggacatgaattttataatccccagaaaaaaaattacattttcctgcgtaatgcagccgaagagctaaagcccaggaataagaagtagtggctggagactctggctcagcgttgtttctctccactctgcctcatctctgtacgaatttgggtcccagccttgttgggttgtcagccatggacatggatttcaacccctggagaaaacgatgtacttgttcagcagtcgagagcccaggcctgctgcgggttctctaagggaactgagactcaatgaagtcggatctcgaggggatcatgagcagcagtgtgtacttgacagcgttgggagggaactgctgatgagccctgtggtgttttataataaaatactttgaagatggcctaactccatcttctccaactc

>DONSON_probe1

GGAAATCACATAGCAGTTACCCCAT

>DONSON_probe2

GTGGTGCTGAGAGACTACATTTATA

>DONSON_probe3

ATACCGTTACTTGGGAAATCATCTT

>DONSON_probe4

CAACTGCTGTATTTAACATCTGCCT

>DONSON_probe5

GATATCTATATCCCTACAACCTAAT

>DONSON_probe6

TAACTGTGGTTTGCACCCTAACACT

>DONSON_probe7

GTTACCCCATGGCATTGTGACTAAT

>KIAA0776_probe1

CCACCTCATACACACACAATTCAGT

>KIAA0776_probe2

GTCATATAGTAAGCATTTTCCCCCA

>KIAA0776_probe3

CATATTTCAGGTTTGTTCTCTTTCC

>KIAA0776_probe4

AAAGTAGTCACTATACAACTCCCCT

>KIAA0776_probe5

AAGACCTTGTTCTCAAATCTAGGAA

>KIAA0776_probe6

GAAGGATTCATTTGTTGCGAGTGCC

>KIAA0776_probe7

GTTGCGAGTGCCAGTATACTTAAAT

>KIAA0776_probe8

TTTTCCAATCCATTTATCCCTGGGG

>KIAA0776_probe9

CCTGATTTTCACACAAATACTATAT

>KIAA0776_probe10

GTAAATTGGGTTCTTCATGGAAGTT

>KIAA0776_probe11

GGAAATCATCTGTGACGGAAGAGTA

>KIAA0776_target

aagaccttgttctcaaatctaggaaatcatctgtgacggaagagtaatgatcttaatttacatttgtcatatagtaagcattttcccccaaggttgaaggtgagtggtcacaaaaaagtagtcactatacaactcccctctccctgcaaaaaccacctcatacacacacaattcagttaaaacagtagtattgtattaaatgtaaatcttaaaaagatgtgaatttttgtaaattgggttcttcatggaagtttttttccacctgattttcacacaaatactatatgaaatttttcacattattttcacataattttaaaaattacatatttcaggtttgttctctttccaaatgttgaatgaaaaacaaattttccaatccatttatccctggggaaggattcatttgttgcgagtgccagtatacttaaat

>KIAA0776_consensus

ggctgacgtgtctgnagttcctccgcgtctactgcgagtcaggccgtgatggcggacgcctgggaagagattaggcggttggcggccgacttccagcgggcgcagttcgccgaggccacgcagaggttgtccgagcggaactgcattgagattgttaataaattgattgctcagaaacagctagaagtagttcatacactcgatggaaaggantatattactccagcccaaattagtaaagaaatgagagatgagctacatgtccgaggtggtcgagtaaacattgttgatctacaacaggtaattaatgtggacctgattcatattgaaaatagaattggtgacattattaaatcagaaaagcatgttcagttagtgttgggacaactgatagatgagaattatttggatcggttggcagaagaggtcaatgataaattgcaagaaagtggtcaggtcaccatatcagaactgtgtaaaacttatgatcttcctgggaactttctgacacaggcactaactcagcgacttggtagaattatcagtggacatattgatcttgataatagaggagtaatttttacggaagctttngtagctcgacataaangcacgtatccgtggactnattcagtgctattnnanccggcntacagnctgtgaattnctttgatttcnananatatnggatttcaggagcagcttctttactcntgtgcttgaggaacttgttaatagcggacgcttacgaggcactgtggttggtgggagacaggataaagctgtgtttgtccctgacatctactccaggacacagagtacttgggtggattcctttttcaggcagaatggctatctagaatttgatgctttgtccagacttggaatcccagatgctgtaagctacataaagaaaagatataagactacacaactcttgtttttgaaagcagcttgtgttggtcaaggacttgtggatcaagtggaagcatcagtagaagaagccatcagctctggaacatgggttgatattgcacctctgctacccacttctttatcagttgaagatgctgccatattgcttcagcaggtgatgagggcattcagcaaacaggcctcaactgtagtctttagcgacactgttgtagtcagtgaaaaatttataaatgactgtacagaactgttccgtgagctgatgcaccagaaagctgaaaaggaaatgaaaaataatcctgtgcatttaatcactgaagaagatctgaaacaaatctccactttagaaagcgttagtacaagtaaaaaggataaaaaagatgagcgaagaaggaaagcaacagagggcagtggaagcatgagaggaggaggtgggggcaatgccagagagtacaaaattaaaaaagtcaagaagaaaggaagaaaagatgatgatagtgatgatgaatctcaatcatcccacactggaaagaagaagccagagatcagttttatgttccaggatgagattgaagattttttaagaaaacacatacaagatgcccctgaggagtttatttcggaacttgctgagtacttaataaaacctcttaataaaacttatctcgaggtggtacgttcagtattcatgtcttcaacaacttctgcttctgggacgggcagaaaacgcacaatcaaggacttgcaagaagaagtttcaaacctgtacaataacattaggttatttgaaaaagggatgaagttttttgcagatgacacacaggctgctcttaccaaacacttgctgaagtcagtgtgtactgatatcactaacctcattttcaacttcttagcttcggatttaatgatggcagtagacgatcctgcagccattacaagtgaaataagaaagaaaattttaagtaaattatcagaagaaaccaaagtagctcttacaaaactccataactctctgaatgaaaagagcatagaagantttatttcttgtctggattctgcagcagaagcttgtgatattatggtgaaaaggggagacaaaaaaagggaaagacagatactgttccaacatcgacaagcactggctgaacagctaaaggtcacagaagaccctgctcttattctgcacctcacatcagtcctgttgtttcagttttcaacccacagcatgctccatgcacctggaagatgtgtcccacagatcattgcttttcttaatagtaaaattccagaggatcagcatgctcttttggtaaagtatcaaggtttggttgtaaagcagctagtcagtcaaagtaagaagactgggcagggagattatcccttgaataatgaattagacaaagaacaagaagatgttgccagtactactcgtaaagagcttcaagaactttcttcatccattaaagaccttgttctcaaatctaggaaatcatctgtgacggaagagtaatgatcttaatttacatttgtcatatagtaagcattttcccccaaggttgaaggtgagtggtcacaaaaaagtagtcactatacaactcccctctccctgcaaaaaccacctcatacacacacaattcagttaaaacagtagtattgtattaaatgtaaatcttaaaaagatgtgaatttttgtaaattgggttcttcatggaagtttttttccacctgattttcacacaaatactatatgaaatttttcacattattttcacataattttaaaaattacatatttcaggtttgttctctttccaaatgttgaatgaaaaacaaattttccaatccatttatccctggggaaggattcatttgttgcgagtgccagtatacttaaatgtatactttatatacatttaagcagaaatttaaaatgtggtcttttttttttaaaagaaacacttctgtgtcagaaatgaaaataaatcatgttttttatttttaaatagaaagcacttcaaaactaaaatacagaaatttacaaaaaanaaaaattattactaagcccttgctcttatttttggcagaataaacattgattttnggttggggctgcaaggatgcctttacaggtgcacaagaacgctagagtggcccatgcattgctgtgttctcttctaaaacaagctactaacaagagagagaacacatgcaatttattaccagtaagcataagtcatatttaacattggaataactaagcattttatacaaagatttttctttgacccagtttgcatttgtacattttatttttttagttcaacagctgctctgtcacataatactttgtaaatactagtgtgaaaaacagataggatgccttttccagtggtaggagcatctgtaagtgcatttaaatcattagaagcttttttccaaaactagaaacccttattaaaaantagacatttttttctaaagattattagttaaaaataactaaaagagttccttagagtaatcatttcaaccaaatgaatttcaagcaacaacttacttactatttatcaaaaagggatttaattattttgaatataattataaattaaatttcaaatatttaaaaatactattttatataattccttatgacattttaaactttatattttaaaatatttgaatacgaaaagatcagtctacctctactccattctagacaaagtattaatccttagtgaaagtaattaattaaattgccaacttggcattattattaaacttgataggaacgcaacattaaatttaaaaatgttttcccgtgggtaattttctattatatatnntttcatatgggcaaagggaaaaaatgataaatcctctgtaatcacaaaccccaatttcgttttgtttattcagcttctaaaatattgaacacccagacttttaattcaacctttaagaaccttatcatttatgtttcagtagatatcaaagtaatccatgtttgtgtcaaatgatcatagaaaataaatagaagagacagtgaagcaagtaaaaagaaaagcattgttttaatttgtttgcattaatttttttcatttgtcaaaatgcttcttttgttgccacagtaaagaacagtttttattgttttgtaagtaaaattacgtagctgattttgtatgtaaagattaatttccataataaaaattattgtatgtttactgtgatcttaatgggcagggttaagaaagttatttaaaataaagttacctattctactaaannnnaaaanannttgaagcttctattaattaacacaaagattaattggtgcatatattttatatatatacattttgaattctcattttgaacattattaaaggattttatttttcttacac

>DTL_probe1

ATGATTTTGTTTGTATCCCTACCCA

>DTL_probe2

GGAAGCCATAGAATTGCTCTGGTCA

>DTL_probe3

AGCACACCATAGCCTTAACTGAATA

>DTL_probe4

TGGGTGCCAAAGGTCAACTGTAATG

>DTL_probe5

GACCAATATCTGCCAGTAACGCTGT

>DTL_probe6

AATTGGGATACATTTGGCTGTCAGA

>DTL_probe7

TAACGCTGTTTATCTCACTTGCTTT

>DTL_probe8

GACAACTTTTTAATTCCTTTGATCT

>DTL_probe9

GTCTACTGGGTATAACATGTCTCAC

>DTL_probe10

GGAAATCTGCCTAATCTGCTTATAT

>DTL_probe11

GCTCTGGTCAAAACCAAGCACACCA

>DTL_target

gacaactttttaattcctttgatcttataagttaaagctgtaacaactgaaattgcatggatcaagtaagcatagttttatccagggagaaaaataaaaggaagccatagaattgctctggtcaaaaccaagcacaccatagccttaactgaatatttaggaaatctgcctaatctgcttatatttggtgtttgttttttgactgttgggctttgggaagatgttatttatgaccaatatctgccagtaacgctgtttatctcacttgctttgaaagccaatgggggaaaaaaatccatgaaaaaaaaaagattgataaagtagatgattttgtttgtatccctacccatctcctggcagccctactgagtgaaattgggatacatttggctgtcagaaattataccgagtctactgggtataacatgtctcacttggaaagctagtacttttaaatgggtgccaaaggtcaactgtaatg

>DTL_exemplar

cgataacgatttgtgttgtgagaggcgcaagctgcgatttctgctgaacttggaggcatttctacgacttttctctcagctgaggcttttcctccgaccctgatgctcttcaattcggtgctccgccagccccagcttggcgtcctgagaaatggatggtcttcacaataccctcttcaatcccttctgactggttatcagtgcagtggtaatgatgaacacacttcttatggagaaacaggagtcccagttcctccttttggatgtaccttctcttctgctcccaatatggaacatgtactagcagttgccaatgaagaaggctttgttcgattgtataacacagaatcacaaagtttcagaaagaagtgcttcaaagaatggatggctcactggaatgccgtctttgacctggcctgggttcctggtgaacttaaacttgttacagcagcaggtgatcaaacagccaaattttgggacgtaaaagctggtgagctgattggaacatgcaaaggtcatcaatgcagcctcaagtcagttgccttttctaagtttgagaaagctgtattctgtacgggtggaagagatggcaacattatggtctgggataccaggtgcaacaaaaaagatgggttttataggcaagtgaatcaaatcagtggagctcacaatacctcagacaagcaaaccccttcaaaacccaagaagaaacagaattcaaaaggacttgctccttctgtggatttccagcaaagtgttactgtggtcctctttcaagacgagaataccttagtctcagcaggagctgtggatgggataatcaaagtatgggatttacgtaagaattatactgcttatcgacaagaacccatagcatccaagtctttcctgtacccaggtagcagcactcgaaaacttggatattcaagtctgattttggattccactggctctactttatttgctaattgcacagacgataacatctacatgtttaatatgactgggttgaagacttctccagtggctattttcaatggacaccagaactctaccttttatgtaaaatccagccttagtccagatgaccagtttttagtcagtggctcaagtgatgaagctgcctacatatggaaggtctccacaccctggcaacctcctactgtgctcctgggtcattctcaagaggtcacgtctgtgtgctggtgtccatctgacttcacaaagattgctacctgttctgatgacaatacactaaaaatctggcgcttgaatagaggcttagaggagaaaccaggaggtgataaactttccacggtgggttgggcctctcagaagaaaaaagagtcaagacctggcctagtaacagtaacgagtagccagagtactcctgccaaagcccccagggtaaagtgcaatccatccaattcttccccgtcatccgcagcttgtgccccaagctgtgctggagacctccctcttccttcaaatactcctacgttctctattaaaacctctcctgccaaggcccggtctcccatcaacagaagaggctctgtctcctccgtctctcccaagccaccttcatctttcaagatgtcgattagaaactgggtgacccgaacaccttcctcatcaccacccatcactccacctgcttcggagaccaagatcatgtctccgagaaaagcccttattcctgtgagccagaagtcatcccaagcagaggcttgctctgagtctagaaatagagtaaagaggaggctagactcaagctgtctggagagtgtgaaacaaaagtgtgtgaagagttgtaactgtgtgactgagcttgatggccaagttgaaaatcttcatttggatctgtgctgccttgctggtaaccaggaagaccttagtaaggactctctaggtcctaccaaatcaagcaaaattgaaggagctggtaccagtatctcagagcctccgtctcctatcagtccgtatgcttcagaaagctgtggaacgctacctcttcctttgagaccttgtggagaagggtctgaaatggtaggcaaagagaatagttccccagagaataaaaactggttgttggccatggcagccaaacggaaggctgagaatccatctccacgaagtccgtcatcccagacacccaattccaggagacagagcggaaagacattgccaagcccggtcaccatcacgcccagctccatgaggaaaatctgcacatacttccatagaaagtcccaggaggacttctgtggtcctgaacactcaacagaattatagattctaatctgagtgagttactgagctttggtccactaaaacaagctgagctttggtccactaaaacaagatgaaaaatacaagagtgactctataactctggtctttaagaaagctgccttttcatttttagacaaaatcttttcaacgctgaaatgtacctaatctggttctactaccataatgtatatgcagcttcccgaggatgaatgctgtgtttaaatttcataaagtaaatttgtcactctagcattttgaatgaatagtcttcactttttaaattattcatcttctctataataatgacatcccagttcatggaggcaaaaaacaagtttcttgttatcctgaaactttctatgctcagtggaaagtatctgccagccacagcatgaggcctgtgaaggctgactgagaaatcctctgctgaagacccctggttctgttctgcctccaacatgtataattttatttgaaatacataatcttttcactatgcttttgtggggttttttttaagtatgtgtaaaaatgtgatgctcagataagtacatttatatcagttcagtgttaaaatgcagtctcttgagttaaagtcatctttattttaaatgcagtgataaatgtcaactcttcggagaaactaggagaacaacaacagaaagctgtgtttgtcttttttctctcaaatatatctcccgtatgagatttcaggtccccatgttttcaccaagcaatctgctatgtcagccaacccaacatcactttctacaggaggttatgatttttgccatttactagaggaagatgttttatgaaatcaatttggggtttgaattcaggtgcagtcatcagttctttaggggctgcaatgttttaaaaaaaataagtcatcagattttaagaaaaaagtgatgatttcttattgatatttttgtaacagaatatagctcttaactgaaaatccagaaccagaaacataaatcttgagtttcttttcatgtacataaaaagcaatagccttttagtatagatagccctgagccaaaaagtaatagaattttctctagatatttaatacagagagtgtatagactgactctaagttaataatgtgcaaaatatcttaaacatccctccccttattcaacaattatgtatcagtgatcttgaaccattgttttatatttttcacctttgtaacctcatggaaagaggctttacatactttctatgtactatttacttagaagggagcccccttccagtcatgaaacttcatttgttttatccatatccctgaggactgtgtagactttatgtcagttctgtgtagactttatgtcagtttttgtcattatttgaaaatctattctgacaactttttaattcctttgatcttataagttaaagctgtaacaactgaaattgcatggatcaagtaagcatagttttatccagggagaaaaataaaaggaagccatagaattgctctggtcaaaaccaagcacaccatagccttaactgaatatttaggaaatctgcctaatctgcttatatttggtgtttgttttttgactgttgggctttgggaagatgttatttatgaccaatatctgccagtaacgctgtttatctcacttgctttgaaagccaatgggggaaaaaaatccatgaaaaaaaaaagattgataaagtagatgattttgtttgtatccctacccatctcctggcagccctactgagtgaaattgggatacatttggctgtcagaaattataccgagtctactgggtataacatgtctcacttggaaagctagtacttttaaatgggtgccaaaggtcaactgtaatgagataattatccctgcctgtgtccatgtcagactttgagctgatcctgaataataaagccttttacctt

>SQLE_probe1

GATTCCCTGCATCAACTAAGAAAAG

>SQLE_probe2

TTGGTGGCGAATGTGTTGCGGGTCC

>SQLE_probe3

GCGTGTTCTGTAATATTTCCTCTAA

>SQLE_probe4

TCTCCTAACCCTCTAGTTTTAATTG

>SQLE_probe5

TCTCAGTAGTGGTGCTGTATTGTAC

>SQLE_probe6

AATTGGACACTTCTTTGCTGTTGCA

>SQLE_probe7

CTTGGATTACAAAACCTCGAGCCCT

>SQLE_probe8

CTGTTGGGCTGCTTTCTGTATTGTC

>SQLE_probe9

ATCTATGCCGTGTATTTTTGCTTTA

>SQLE_probe10

TTGCTGTTGCAATCTATGCCGTGTA

>SQLE_probe11

ATAGCATAGTACCATACCACTTATA

>SQLE_target

gattccctgcatcaactaagaaaagcctgttttctttatttcaaacttggtggcgaatgtgttgcgggtcctgttgggctgctttctgtattgtctcctaaccctctagttttaattggacacttctttgctgttgcaatctatgccgtgtatttttgctttaagtcagaaccttggattacaaaacctcgagcccttctcagtagtggtgctgtattgtacaaagcgtgttctgtaatatttcctctaatttactcagaaatgaagtatatggttcattaagcttaaaggggaaccatttgtgaatgaatatttggaacttaccaagtcctaagagacttttggaagaggatatatatagcatagtaccataccacttata

>SQLE_exemplar

ctggtctgatcggacttctcgtcctgggacacagtttactggagtctggccggctctccgtgctcctcttggtacctcattttggggagaaccttaaacccactcgagcagataatctccgccttgaccggtgccaccaaagaagccttggaaccatgtggacttttctgggcattgccactttcacctatttttataagaagttcggggacttcatcactttggccaacagggaggtcctgttgtgcgtgctggtgttcctctcgctgggcctggtgctctcctaccgctgtcgccaccgaaacgggggtctcctcgggcgccagcagagcggctcccagttcgccctcttctcggatattctctcaggcctgcctttcattggcttcttctgggccaaatccccccctgaatcagaaaataaggagcagctcgaggccaggaggcgcagaaaaggaaccaatatttcagaaacaagcttaataggaacagctgcctgtacatcaacatcttctcagaatgacccagaagttatcatcgtgggagctggcgtgcttggctctgctttggcagctgtgctttccagagatggaagaaaggtgacagtcattgagagagacttaaaagagcctgacagaatagttggagaattcctgcagccgggtggttatcatgttctcaaagaccttggtcttggagatacagtggaaggtcttgatgcccaggttgtaaatggttacatgattcatgatcaggaaagcaaatcagaggttcagattccttaccctctgtcagaaaacaatcaagtgcagagtggaagagctttccatcacggaagattcatcatgagtctccggaaagcagctatggcagagcccaatgcaaagtttattgaaggtgttgtgttacagttattagaggaagatgatgttgtgatgggagttcagtacaaggataaagagactggagatatcaaggaactccatgctccactgactgttgttgcagatgggcttttctccaagttcaggaaaagcctggtctccaataaagtttctgtatcatctcattttgttggctttcttatgaagaatgcaccacagtttaaagcaaatcatgctgaacttattttagctaacccgagtccagttctcatctaccagatttcatccagtgaaactcgagtacttgttgacattagaggagaaatgccaaggaatttaagagaatacatggttgaaaaaatttacccacaaatacctgatcacctgaaagaaccattcttagaagccactgacaattctcatctgaggtccatgccagcaagcttccttcctccttcatcagtgaagaaacgaggtgttcttcttttgggagacgcatataatatgaggcatccacttactggtggaggaatgactgttgcttttaaagatataaaactatggagaaaactgctaaagggtatccctgacctttatgatgatgcagctattttcgaggccaaaaaatcattttactgggcaagaaaaacatctcattcctttgtcgtgaatatccttgctcaggctctttatgaattattttctgccacagatgattccctgcatcaactaagaaaagcctgttttctttatttcaaacttggtggcgaatgtgttgcgggtcctgttgggctgctttctgtattgtctcctaaccctctagttttaattggacacttctttgctgttgcaatctatgccgtgtatttttgctttaagtcagaaccttggattacaaaacctcgagcccttctcagtagtggtgctgtattgtacaaagcgtgttctgtaatatttcctctaatttactcagaaatgaagtatatggttcattaagcttaaaggggaaccatttgtgaatgaatatttggaacttaccaagtcctaagagacttttggaagaggatatatatagcatagtaccataccacttataaagtggaaactcttggaccaagattaggattaatttgtttttgaagttttttgtatataaatatgtaaatacatgctttaatttgcaatttaaaatgaaggggttaaataagttagacatttaaaagaaatgattgttaccataaattagtgctaatgctgaggagaactacagtttttcttttgaatttagtatttgagatgagttgttgggacatgc

>ACBD3_probe1

GAACGCAGAGCGACTCGAGGTGTCC

>ACBD3_probe2

GGCAAAGCATTTCATCCAACTTATG

>ACBD3_probe3

GCCTGGAGGAGTTGTACGGCCTGGC

>ACBD3_probe4

GCAGCAGCCGGAGATGGCGGCGGTG

>ACBD3_probe5

TTAAATAGGTGTTGCCATCTCTTTT

>ACBD3_probe6

GACACTTGTCCTGAGGTTGGATTCT

>ACBD3_probe7

GGCAGCCCTGGGAAACATGTCTAAA

>ACBD3_probe8

TCAACATATGTTGCGTCCCACAAAA

>ACBD3_probe9

TGGCACTGCGCTTCTTCAAAGAAAA

>ACBD3_probe10

TAAGCAAGTTCTTATGGGCCCATAT

>ACBD3_probe11

GGCCCATATAATCCAGACACTTGTC

>ACBD3_target

gcagcagccggagatggcggcggtgctgaacgcagagcgactcgaggtgtccgtcgacggcctcacgctcagcccggacccggaggagcggcctggggcggagggcgccccgctgctgccgccaccgctgccaccgccctcgccacctggatccggtcgcggcccgggcgcctcaggggagcagcccgagcccggggaggcggcggctgggggcgcggcggaggaggcgcggcggctggagcagcgctggggtttcggcctggaggagttgtacggcctggcactgcgcttcttcaaagaaaaagatggcaaagcatttcatccaacttatgaagaaaaattgaagcttgtggcactgcataagcaagttcttatgggcccatataatccagacacttgtcctgaggttggattctttgatgtgttggggaatgacaggaggagagaatgggcagccctgggaaacatgtctaaagaggatgccatggtggagtttgtcaagctcttaaataggtgttgccatctcttttcaacatatgttgcgtcccacaaaa

>ACBD3_consensus

ggaagtcgatacgtggctgccntctgtccccgctgaggaggtgcagcagccggagatggcggcggtgctgaacgcagagcgactcgaggtgtccgtcgacggcctcacgctcagcccggacccggaggagcggcctggggcggagggcgccccgctgctgccgccaccgctgccaccgccctcgccacctggatccggtcgcggcccgggcgcctcaggggagcagcccgagcccggggaggcggcggctgggggcgcggcggaggaggcgcggcggctggagcagcgctggggtttcggcctggaggagttgtacggcctggcactgcgcttcttcaaagaaaaagatggcaaagcatttcatccaacttatgaagaaaaattgaagcttgtggcactgcataagcaagttcttatgggcccatataatccagacacttgtcctgaggttggattctttgatgtgttggggaatgacaggaggagagaatgggcagccctgggaaacatgtctaaagaggatgccatggtggagtttgtcaagctcttaaataggtgttgccatctcttttcaacatatgttgcgtcccacaaaatagagaaggaagagcaaganaaaaaaaggaaggaggaagaggagcgaaggcggcgtgaagaggaagaaagagaacgtctgcaaaaggaggaagagaaacgtaggagagaagaagaggaaaggcttcgacgggaggaagaggaaaggagacggatagaagaagaaaggcttcggttggagcagcaaaagcagcagataatggcagctttaaactcccagactgccgtgcagttccagcagtatgcagcccaacagtatccagggaactacgaacagcagcaaattctcatccgccagttgcaggagcaacactatcagcagtacatgcagcagttgtatcaagtccagcttgcacagcaacaggcagcattacagaaacaacaggaagtagtagtggctgggtcttccttgcctacatcatcaaaagtgaatgcaactgtaccaagtaatatgatgtcagttaatggacaggccaaaacacacactgacagctccgaaaaagaactggaaccagaagctgcagaagaagccctggagaatggaccaaaagaatctcttccagtaatagcagctccatccatgtggacacgacctcagatcaaagacttcaaagagaagattcagcaggatgcagattccgtgattacagtgggccgaggagaagtggtcactgttcgagtacccacccatgaagaaggaatcatatctctttttgggaatttgccacagacaattatgacattgggtttggggtgtattttgaatggacagactctccaaacactgctgtcagcgtgcatgtcagtgagtccagcgatgacgacgaggaggaagaagaaaacatcggttgtgaagagaaagccaaaaagaatgccaacaagcctttgctggatgagattgtgcctgtgtaccgacgggactgtcatgaggaggtgtatgctggcagccatcaatatccagggagaggagtctatctcctcaagtttgacaactcctactctttgtggcggtcaaaatcagtctactacagagtctattatactagataaaaatgttggttacaaagtctggagtctagggttgggcagaagatgacatttaatttggaaatttctttttacttttgtggagcattagagtcacagtttaccttattgatattggtctgatggtttgtgaactcttgctgggaatcaaaatttccttgagactctttagcattcatactttggggttaaaggagattcctcagactcatccagcccttgggtgctgaccagcagagtcactagtggatgctgaagttacatgagctacatgttaaatatttaaagtctccaaaataaaacaccccaacgttgaccttacccggctgatggttagccccttgctgcctgctccatgtgtcttatgagagcccgtagttacagtgtcctctaatttgaaatccataagttaacaagtctatatcaggtgcagctggctttgattaaaggccatttttaaaacttaaaaactcaacacctcacagattataatagaaaaagaaatggcctcagtttgatctcgttcagaatgacccagattgtttctgctttgggtgcagctgtttagttcagagttatattacagagaattattttctgagataatcttaaactagaatgttcaaaactaattgataattgaagtatcaagatacgtagaacacctcagagatttttcttcaggaacttccacaaactttgaatccttgtatctttatttggtattcatactactagtagcaaaatacaggttttttgttttgttttgttttgnnnnngcttcatagagtatctcaaattgaaacttttctgcacaaagaataaaattaaggattttataaactcaaattggcacctactgaattaaaatacataaaatcatttaaatataattcagcatatgggaagtaacattgcactaatatggaaatcactgccagagacagtctattttcttttaatttgttactacttagtcacaaaccccacattattccagtttggaattacttattaaggagaattggaaatacatatgcccatgcttaaattttatagctttaatttgtgttatttctttattgacgggaagaggtacatctttttttccttactgaaaaccaaatatggattaattgcctcaaatttgtataaagtgattggctagtgattcttgttttcaggaagggagagtggtatagatagaaaatgacaaagatggcaatatacacttaatgttgttattgtatgttgttactgaagtacttagatttttaaaatttcaaatcctaaatcacttcttgtaggagggttttcattaactgcagtatatacagttcactacatatgggttgtttgagttttttgtgtgctgtatttctttctgttttttnaatacctggttttgtacatatctaactctgttctcttttggttgttcagaaactggattttttttttttcttaagcagtgcttaatttgtgttttttaattttgattcagaagtagtcccagctcataggtgttcataactgttacatccagaacatttgtcaggctctctgtcagcttttcatgtacatatggtatagaaaccatggagttaggcacttccntggantttttttttttatgagaaaaatactgtatttaaaatgtaaaataaacttttaaaaagcaggcactaatatatatttcttccagcctttgattacaaatttgtccttgcacatgttaagatgaattatctcctaaaaatatcattgttcttgggagcagtgtatgttactttacatagcagcggttcctgtcatgtgttcatgttcagaaatatttttggttttaaactttcttattgcctttggctgttgattagtacagtacaagtngcgatttcaaaaagatcttgaaagtaatatatttaatcaattaaaatgtttatctgtaaaaaaaaaaaaaaaaaaaaaaaaaaa

>RMI1_probe1

CCCTGAACATGCCTGAGCTTGTCAT

>RMI1_probe2

TCCCTTTCTGAATTAGCTGTACATA

>RMI1_probe3

GCATTTATCTATGTCTTTAGGTGTC

>RMI1_probe4

GGTAATTTCCTTCTAATATGTTGGT

>RMI1_probe5

TACCATTCTTCCACTGTGCTGTTAT

>RMI1_probe6

GTAGATCAGAACATCAGGCTTTCAG

>RMI1_probe7

ATGCCTGAGCTTGTCATAATATGTT

>RMI1_probe8

ATGTTGGTACTGTCTATGGCCATAC

>RMI1_probe9

TTAGGTGTCATTGTTCCCTTTCTGA

>RMI1_probe10

AGAGGGACTGTTTACCATTCTTCCA

>RMI1_probe11

GCTGTACATATAAGCCTTCCTTTGG

>C14orf101_probe1

CAGAAAGCACCGAATGACCCACAGC

>C14orf101_probe2

TTCCGTCTGTACTCTCAGAAAGCAC

>C14orf101_probe3

GAAGTGCTGTTATCGGAAACCATCA

>C14orf101_probe4

GACTTCCAGTCTTTCACCAGATGAC

>C14orf101_probe5

GTATCATGGTCCAGCAGTACTGTTT

>C14orf101_probe6

TATCCCGTGTTACCAAATTACCATT

>C14orf101_probe7

GAAACCATCAGACATTTCCGTCTGT

>C14orf101_probe8

ATGAAAAACCTGCTCATCGTTCAGC

>C14orf101_probe9

TTAAAACTAAGTCATCTCCCAGATA

>C14orf101_probe10

GCAAAATTCTGTTTATCCCGTGTTA

>C14orf101_probe11

CTCATCGTTCAGCTTCCAAAATTCT

>ZNF274_probe1

CCTTTTCAGCTTGACCCTGCAATAT

>ZNF274_probe2

AATCTGCACTGATATTACATCCACA

>ZNF274_probe3

ACCTCATAGCTCTCAAGCCAGTTGA

>ZNF274_probe4

AGGAGACTGCCCAGCACATAATGAA

>ZNF274_probe5

GATATTGTTTGTTCACTCATTTAGT

>ZNF274_probe6

ACATGCACAGGCCTGCTTGTGAATC

>ZNF274_probe7

GCCAGTTGAAGAAACCTTGCCTTTT

>ZNF274_probe8

AAGATTTCCCATTCACTTGATATTG

>ZNF274_probe9

TACATCCACAGTACCACAGTATTTA

>ZNF274_probe10

GAAGAAACAGCCTACCTCATAGCTC

>ZNF274_probe11

GAGCGCCCATATGCATGCAACAAAT

>ZNF274_target

gagcgcccatatgcatgcaacaaatgtggaaaggccttcacccagagctcacaccttattgggcaccagagaacccacaataggacaaagcgaaagaagaaacagcctacctcatagctctcaagccagttgaagaaaccttgccttttcagcttgaccctgcaatataacatgcacaggcctgcttgtgaatcaggactgaatgtgaaagggaagtattgagtgaggacattcccaaaaccaaaggacaactgaggagactgcccagcacataatgaataaataagaaaatgagtgaggagttattaacatcatttggaaaaaagatttcccattcacttgatattgtttgttcactcatttagtcattaaaagtgagattaataaaatctgaaaatgttatataataactttaaaaagccaggtaattaataatctgcactgatattacatccacagtaccacagtattta

>ZNF274_exemplar

gtccagcgcctttcctttctccagcttctgctctgccccaagagtggtcgccttcgtggcagggcacgactctctcccagctcgggtcgccgcccacattagtgtggggccctgcggcctagcgtccctcaccagaggcctccccttgcctagctggaccgccgagggacatcgacgagtatcctcctcctgctgtccccggcttcgcctgccgcccctaaccggccagtcaagatggccgccgctgggtgaggcaagctggcgcgccgcgggggcgtctgggagttgtagttcgggacggcgggctgacgcacttcgccgccggccgacgggcgccattgtgcggcgcgcgccgggactctgcccacttccaccagagacacattgagaaggaggaaactatggcctccaggcttccgacggcctggtcctgtgaaccagtgacctttgaagatgtaacactgggttttaccccggaagagtggggactgctggacctcaaacagaagtccctgtacagggaagtgatgctggagaactacaggaacctggtctcagtggagtatcctgagctccagctggaccctaaattggatcctcttcctgctgagagtcccctaatgaacattgaggttgttgaggtcctcacactgaaccaggaggtggctggtccccggaatgcccagatccaggccctatatgctgaagatggaagcctgagtgcagatgcccccagtgagcaggtccaacagcagggcaagcatccaggtgaccctgaggccgcgcgccagaggttccggcagttccgttataaggacatgacaggtccccgggaggccctggaccagctccgagagctgtgtcaccagtggctacagcctaaggcacgctccaaggagcagatcctggagctgctggtgctggagcagttcctaggtgcactgcctgtgaagctccggacatgggtggaatcgcagcacccagagaactgccaagaggtggtggccctggtagagggtgtgacctggatgtctgaggaggaagtacttcctgcaggacaacctgccgagggcaccacctgctgcctcgaggtcactgcccagcaggaggagaagcaggaggatgcagccatctgcccagtgacagtgctccctgaggagccagtgaccttccaggatgtggctgtggacttcagccgggaggagtgggggctgctgggcccgacacagaggaccgagtaccgcgatgtgatgctggagacctttgggcacctggtctctgtggggtgggagactacactggaaaataaagagttagctccaaattctgacattcctgaggaagaaccagcccccagcctgaaagtacaagaatcctcaagggattgtgccttgtcctctacattagaagataccttgcagggtggggtccaggaagtccaagacacagtgttgaagcagatggagtctgctcaggaaaaagaccttcctcagaagaagcactttgacaaccgtgagtcccaggcaaacagtggtgctcttgacacaaaccaagtttcgctccagaaaattgacaaccctgagtcccaggcaaacagtggcgctcttgacacaaaccaagttttgctccacaaaattcctcctagaaaacgattgcgcaaacgtgactcacaagttaaaagtatgaaacataattcacgtgtaaaaattcatcagaagagctgtgaaaggcaaaaggccaaggaaggcaatggttgtaggaaaaccttcagtcggagtactaaacagattacgtttataagaattcacaaggggagccaagtttgccgatgcagtgaatgtggtaaaatattccggaacccaagatacttttctgtgcataagaaaatccataccggagagaggccctatgtgtgtcaagactgtgggaaaggatttgttcagagctcttccctcacacagcatcagagagttcattctggagagagaccatttgaatgtcaggagtgtgggaggaccttcaatgatcgctcagccatctcccagcacctgaggactcacactggcgctaagccctacaagtgtcaggactgtggaaaagccttccgccagagctcccacctcatcagacatcagaggactcacaccggggagcgcccatatgcatgcaacaaatgtggaaaggccttcacccagagctcacaccttattgggcaccagagaacccacaataggacaaagcgaaagaagaaacagcctacctcatagctctcaagccagttgaagaaaccttgccttttcagcttgaccctgcaatataacatgcacaggcctgcttgtgaatcaggactgaatgtgaaagggaagtattgagtgaggacattcccaaaaccaaaggacaactgaggagactgcccagcacataatgaataaataagaaaatgagtgaggagttattaacatcatttggaaaaaagatttcccattcacttgatattgtttgttcactcatttagtcattaaaagtgagattaataaaatctgaaaatgttatataataactttaaaaagccaggtaattaataatctgcactgatattacatccacagtaccacagtatttatgtgtatgaattaaggattaaaagataatgtggataaataaactattgatctatg

>PTGES_probe1

TGGATGTCTTTGCTGCAGTCTTCTC

>PTGES_probe2

GGTCTTGGGTTCCTGTATGGTGGAA

>PTGES_probe3

CAAAGGAACTTTCTGGTCCCTTCAG

>PTGES_probe4

TTGGCCACCAGACCATGGGCCAAGA

>PTGES_probe5

CAAAGGGCAGTGGGTGGAGGACCGG

>PTGES_probe6

TCTCCTAGACCCGTGACCTGAGATG

>PTGES_probe7

CGTGGCTATACCTGGGGACTTGATG

>PTGES_probe8

CAGCCACTCAAAGGAACTTTCTGGT

>PTGES_probe9

GGTTTGGAAACTGCAAATGTCCCCT

>PTGES_probe10

AGGTTTGAGTCCCTCCAAAGGGCAG

>PTGES_probe11

GGCCCACCGGAACGACATGGAGACC

>PTGES_probe12

TCTCTGGGCACAGTGGGCCTGTGTG

>PTGES_probe13

CTCTGGGCACAGTGGGCCTGTGTGT

>PTGES_probe14

TTTGGATGTCTTTGCTGCAGTCTTC

>PTGES_probe15

ACCTGGGGACTTGATGTTCCTTCCA

>PTGES_probe16

GGCTATACCTGGGGACTTGATGTTC

>PTGES_probe17

CTCCTAGACCCGTGACCTGAGATGT

>PTGES_probe18

TGGAGGACCGGGAGCTTTGGGTGAC

>PTGES_probe19

CACCAGACCATGGGCCAAGAGCCGC

>PTGES_probe20

GCAGTGGGTGGAGGACCGGGAGCTT

>PTGES_probe21

TTTCTGGTCCCTTCAGTATCTTCAA

>PTGES_probe22

CACCGGAACGACATGGAGACCATCT

>FRG1_probe1

GGCTCGGAAAGATGGATTTTTGCAT

>FRG1_probe2

GCAGTTTTCGGCTGTCAAATTATCT

>FRG1_probe3

GGGCGTTCAGATGCAATTGGACCAA

>FRG1_probe4

TAGTCCTCCAGAGCAGTTTTCGGCT

>FRG1_probe5

ATTGCCCTGAAGTCTGGCTATGGAA

>C19orf60_probe1

GCACGGTGGCCCTGCTGCAGTTGAT

>C19orf60_probe2

GGCGATCAGCGAGGTTCTCCAGGAC

>C19orf60_probe3

GGACCTTAGGTTTGATGCGGAATCT

>C19orf60_probe4

GGTTCTCCAGGACCTTAGGTTTGAT

>C19orf60_probe5

CAGCGAGGTTCTCCAGGACCTTAGG

>C19orf60_probe6

AATTAAAACCATGGAGGCGATCAGC

>C19orf60_probe7

CCTTAGGTTTGATGCGGAATCTGCC

>C19orf60_probe8

CGCTGTACGGATGCAGCAGCTGAAA

>C19orf60_probe9

TCTGCCGAGTGATGGCGGCTCCCCA

>C19orf60_probe10

CATGGAGGCGATCAGCGAGGTTCTC

>C19orf60_probe11

GTTTGATGCGGAATCTGCCGAGTGA

>C19orf60_probe12

ACTGCGCTGCTGACCTTCCTGCAGT

>C19orf60_probe13

ATTAAAACCATGGAGGCGATCAGCG

>C19orf60_probe14

CCAGGACCTTAGGTTTGATGCGGAA

>C19orf60_probe15

AGTGCACGGGGTGACCCAGGCCTTC

>C19orf60_probe16

ATCTGCCGAGTGATGGCGGCTCCCC

>C19orf60_target

agtgcacggggtgacccaggccttcgccgccgcctcgcgggaggtgctggcggtggaagcagagctgggcgggcctcgcaggcagccgctgctcgccggccacgtgcgcagcctgcaggagctggagcagacgcggctgggcacggtggccctgctgcagttgatggagacgccagagctggcggggcaggaggacgctgtacggatgcagcagctgaaaatgaannnnnnnnnnnnnnnnnnnggtaattaaaaccatggaggcgatcagcgaggttctccaggaccttaggtttgatgcggaatctgccgagtgatggcggctccccagggatgcgccgagggagatgggaaacggggcggatggcgcccagcccagccctaactgccagctggctggggtngcgccccactgcgctgctgaccttcctgcagt

>C19orf60_consensus

attcttcacgagggaaaaacacgatcggaggcgggccctggacaggaacaagaagggtgggcttgactgggcggcccgcggtcttggctcccctttcttccttctggctggccggccgcagggtagggggtccgtggacctcccggcctgtagggggcgtcgggcgcagcggaagggcctgctccggagcctggccagggntgcgcagtcatgatcancgagaccgcggcggancnnnnggtccctgcagtgcctgctgctgaggaggccaccgaagctcggggacgcgaggagccggcgtggccntggaaagacgccccgatccggacgctnnnnnnnnnnnnnnnnnnnnnncaggctgagcgcgcgcagggnnnnnnnnnnnnnnnnnnaggccaccgcagtacctgcgcancggccctgactacgacttcgcgcgctaccggagcacagtgcacggggtgacccaggccttcgccgccgcctcgcgggaggtgctggcggtggaagcagagctgggcgggcctcgcaggcagccgctgctcgccggccacgtgcgcagcctgcaggagctggagcagacgcggctgggcacggtggccctgctgcagttgatggagacgccagagctggcggggcaggaggacgctgtacggatgcagcagctgaaaatgaannnnnnnnnnnnnnnnnnnggtaattaaaaccatggaggcgatcagcgaggttctccaggaccttaggtttgatgcggaatctgccgagtgatggcggctccccagggatgcgccgagggagatgggaaacggggcggatggcgcccagcccagccctaactgccagctggctggggtngcgccccactgcgctgctgaccttcctgcagttccagacacctcccncaataaagagctcctcctctgtaaaaaaaaaaaaaaaaaaaaaaaaaaaaaaaaa

>LPCAT1_probe1

TGTGTGTGAGACAGGACGCAGCGGG

>LPCAT1_probe2

CAGACCCGTGGGCAGGTGGGGCATG

>LPCAT1_probe3

GTTGAGTTAAACCCCTTGTGTGTGA

>LPCAT1_probe4

TCCCTTCCGCAGGTCTGCAGATGAA

>LPCAT1_probe5

AATTTCAGGGCTCTTGGCGTGTTGG

>LPCAT1_probe6

TGAAATGCCACTGCGCATTTTCAGA

>LPCAT1_probe7

TCTTTTCTCTTCGTGGCGACTTAGA

>LPCAT1_probe8

GCCTTTGGTAGCTAACAGTCACTGA

>LPCAT1_probe9

AGAAATCCTAGTGCAGCCTTTGGTA

>LPCAT1_probe10

TGAATGGATGTTTGTTCCTCCTGAT

>LPCAT1_probe11

GAGTTGGCGGATATTCGGAACTGTG

>LPCAT1_target

aatttcagggctcttggcgtgttggacacagaagaaatcctagtgcagcctttggtagctaacagtcactgattttataattggagaatgcgtaaagattcatttttcaaggagaagagcctgcaaatggccaatgaaggaggtaaataaactaagatattccgagggaagggacccaggccacctcccttccgcaggtctgcagatgaagggttttttgaatgaaatgccactgcgcattttcagaaaaaaaaatctctgataaacagactttgaatggatgtttgttcctcctgattctcttttctcttcgtggcgacttagagttggcggatattcggaactgtgaatgtacatagcgttgagttaaaccccttgtgtgtgagacaggacgcagcgggcccctggtggcctgggggccagacccgtgggcaggtggggcatg

>LPCAT1_exemplar

tttacgatatccaaataaactggacaccatcacatggacgtggcaaggacctggagcgctggaaatcctgtggctcacgctgtgtcagtttcacaaccaagtggaaatcgagttccttcctgtgtacagcccttctgaggaggagaagaggaaccccgcgctgtatgccagcaacgtgcggcgagtcatggccgaggccttgggtgtctccgtgactgactacacgttcgaggactgccagctggccctggcggaaggacagctccgtctccccgctgacacttgccttttagaatttgccaggctcgtgcggggcctcgggctaaaaccagaaaagcttgaaaaagatctggacagatactcagaaagagccaggatgaagggaggagagaagataggtattgcggagtttgccgcctccctggaagtccccgtttctgacttgctggaagacatgttttcactgttcgacgagagcggcagcggcgaggtggacctgcgagagtgtgtggttgccctgtctgtcgtctgccggccggcccggaccctggacaccatccagctggctttcaagacgtacggagcgcaagaggacggcagcgtcggcgaaggtgacctgtcctgcatcctcaagacggccctgggggtggcagagctcaccgtgaccgacctattccgagccattgaccaagaggagaaggggaagatcacattcgctgacttccacaggtttgcagaaatgtaccctgccttcgcagaggaatacctgtacccggatcagacacatttcgaaagctgtgcagagacctcacctgcgccaatcccaaacggcttctgtgccgatttcagcccggaaaactcagacgctgggcggaagcctgttcgcaagaagctggattaggacccagggttgcggagagacgcggcccctcccgcgtggacatcaccgccatgagcctctttgcgagtgacctctgggctccgctcctcactcctgctgtacaggcactgtcttcagcccgagttccaggggcctcgggggctgtttgtatcttgttcctttgtgaagtgtgttgcagaaccgacgcttactgtgcgagaatcggagggcgcgcacgcggatcccccgcctggcctggaccccgtggggtcaggttccctgccgggcggggggcaccggtgccgccccgtgttctcccacggggccctggtttcgagtctctgtcacagcctcttccggcggcagcgtgcaccgggcgggcctccgtgcacactcagcacacgcctgccacacagcgtgcgcttgcgtgtcactctggcacgaaacctgtctgcctctgtggatccacagcctggcagagccgagccgtcacctgatttttcagtgtttctacctgtgtgctggagctcatgagtattttataaactccatttaggtacttcaggaaacatgcagcattttttaaaaaatgaaaattgtttttctacttcatttttccttttagagtcaaaggatatttatttataggccttttttttttaatatagaatctgaggctgtttgggctttgacttaaatttccatcaggcctctctccagcaggtaatccctctgcttccgctgggtcccctggggaggtgtgaactcaagggcctagccccaaaacactttttctgcttttcttaatccttttccagtcccctctttttttataaacgttggcagtttgatgtttctgtttcggcataacgtaatccatttcactgtagcctaaactccagtccgaggttggatattgttcaaatgagcagggcccgagctggaagcgcaaggcagccgccgccgtgccgctcctcccttgccctcaggccaggtccctgctggaagcggctgcatcttcctgtcagccctggtttccatggtgactggcgtgacgcagccacctgagtatggctgaccttcctgcagagagaggagccgcagtcttttgcttgtggaaggagacgctgggctgtgcggtgcggagggtgatgaggatgtctggtgacagccgtgcggacaccactcctctctgcagcactgcctcccagcgccagggtcgcgggcacatcccactgagagcgggggtcctgccccatcttagagtcaaaggcagaggggcttccaggccctggatggggtattttggtgtcacctgaagtccctctgacatcaccttgtttcatcattttttatgacagaattagaaacccatccttcaagcacaataatcatcacagacttgagtttgcttcctaaagcaaaggctccgggtttgtttggaaaatttttttgatttctgaaatgaattgatttttatatttggggcatctctatagaaagtgaccaccaaggccagtaagtacgggaaaaaatgtttactaacttcctcagagattcgtgatacgcgtttctccactgacagacatttaaaaacaaccttcagctccgtttcaatcaatcacctcgacttgttttttagcatggacactgccagcaggacagacagggatggagtaaaccgaagtcaatttcagggctcttggcgtgttggacacagaagaaatcctagtgcagcctttggtagctaacagtcactgattttataattggagaatgcgtaaagattcatttttcaaggagaagagcctgcaaatggccaatgaaggaggtaaataaactaagatattccgagggaagggacccaggccacctcccttccgcaggtctgcagatgaagggttttttgaatgaaatgccactgcgcattttcagaaaaaaaaatctctgataaacagactttgaatggatgtttgttcctcctgattctcttttctcttcgtggcgacttagagttggcggatattcggaactgtgaatgtacatagcgttgagttaaaccccttgtgtgtgagacaggacgcagcgggcccctggtggcctgggggccagacccgtgggcaggtggggcatgggccctggcctgcggggacctgctggggtgtgagggcagagggagggttgccatgaaggaacttgggattttcaatggaataaataaaacataaagtctatacttggg

>ISYNA1_probe1

TACCTCGGAGCTGATGCTGGGCGGA

>ISYNA1_probe2

ACCAATGGCTGCACCGGTGATGCCA

>ISYNA1_probe3

CAACACGTGTGAGGACTCGCTGCTG

>ISYNA1_probe4

GCCTCAAGCGAGTTGGACCCGTGGC

>ISYNA1_probe5

GCCACCTACCCTATGTTGAACAAGA

>ISYNA1_probe6

GGAACCAACACACTGGTGCTGCACA

>ISYNA1_probe7

GTGAGCTTCTGCACTGACATGGACC

>ISYNA1_probe8

AACCACATGCTCCTGGAACACAAAA

>ISYNA1_probe9

GGGCATCTGCAAGAGGAGCCCCCAA

>ISYNA1_probe10

CAAAATGGAGCGCCCAGGGCCCAGC

>ISYNA1_probe11

CCAGCGCAGCTGCATCGAGAACATC

>ISYNA1_target

tacctcggagctgatgctgggcggaaccaacacactggtgctgcacaacacgtgtgaggactcgctgctggccgcacccatcatgctggacctagcgctgctgaccgagctgtgccagcgcgtgagcttctgcactgacatggaccccgagccgcagaccttccaccccgtgctgtccctgctcagcttcctcttcaaggcgccactagtgccgcccggcagcccggtggtcaatgcgcttttccgccagcgcagctgcatcgagaacatcctcagggcctgcgtggggctcccgccacagaaccacatgctcctggaacacaaaatggagcgcccagggcccagcctcaagcgagttggacccgtggctgccacctaccctatgttgaacaagaaaggaccggtacccgctgccaccaatggctgcaccggtgatgccaatgggcatctgcaagaggagcccccaa

>ISYNA1_consensus

gggagtcttacaggggcggagcttaaggtgccggaggtctcaggggaggggcctgggggcagcggcttccgtcctggctgtgagcctggaactgaaaaatgcccgaaagtggggcggggcttgagagtctaggtggggcccaggccgggtcgcggccctttcaccactcccacccgtcacctcgccgtgtctgctgacccccaggaggccaactactacggctcgctgactcaggcgggcaccgtgagcctgggcctggacgccgagggccaggaggtgttcgtacccttcagcgcggtgctgcccatggtggcgcccaacgacctcgtgttcgatggctgggacatctcgtcgctgaacctggccgaggcgatgcggcgcgcgaaggtgctggactgggggctgcaggagcaactgtggccgcacatggaggccctgcggccccggccttctgtttacatccccgaattcatcgcggccaaccagagcgcgcgcgcggacaacctcatcccaggctcgcgtgcgcagcagctggagcagatccgcagggacatccgagacttccggtctagcgcggggctggacaaagtcatagtgctgtggacggcgaacacggagcgcttctgtgaggtgattccaggcctcaacgacacagccgagaacctgctgcgcaccattgagctcggtctggaggtgtcgccctccacgctcttcgccgtggccagcatcctggagggctgtgccttcctcaatgggtctccgcagaacaccctggtgcccggagctcttgagctcgcgtggcagcaccgggtttttgtgggcggagatgacttcaagtcaggccagaccaaagtcaagtccgtgcttgtggacttcctcattggctccggcctcaagaccatgtccatcgtgagttacaaccacctgggcaacaacgatggggagaacctatcggcgccattgcagttccgctctaaggaggtgtccaagagcaacgtggtggacgacatggtgcagagcaacccagtgctctatacgcccggcgaagagcctgaccactgcgtggtcatcaagtatgtgccgtacgtgggtgacagcaagcgcgcgctggatgagtatacctcggagctgatgctgggcggaaccaacacactggtgctgcacaacacgtgtgaggactcgctgctggccgcacccatcatgctggacctagcgctgctgaccgagctgtgccagcgcgtgagcttctgcactgacatggaccccgagccgcagaccttccaccccgtgctgtccctgctcagcttcctcttcaaggcgccactagtgccgcccggcagcccggtggtcaatgcgcttttccgccagcgcagctgcatcgagaacatcctcagggcctgcgtggggctcccgccacagaaccacatgctcctggaacacaaaatggagcgcccagggcccagcctcaagcgagttggacccgtggctgccacctaccctatgttgaacaagaaaggaccggtacccgctgccaccaatggctgcaccggtgatgccaatgggcatctgcaagaggagcccccaatgcccaccacctgaggccccggtcacacagtttctcggctcttcctccccgctgccccccacgaccctaccttgaaggcccccacaaataaaggcgctgccactcaaaaaaaaaaaaaaaaaaaaa

>SKP2_probe1

AAATTGATGACTTGTTCGTATGTTC

>SKP2_probe2

GAAGTGCCTTTATCTGCTTAGACCT

>SKP2_probe3

TGCCCTCAAACATACAGAACTTCCA

>SKP2_probe4

CTCTGACATCGGATGCCCTCAAACA

>SKP2_probe5

AGCTATTTTGCCAACATGTCAGAGT

>SKP2_probe6

AGAACTTCCAAACTCAAGTCCAGCC

>SKP2_probe7

AAGTCCAGCCATAAGCTATTTTGCC

>SKP2_probe8

AGAGCTGGGGTTAGGATCCGGTTGG

>SKP2_probe9

TAGGATCCGGTTGGACTCTGACATC

>SKP2_probe10

AAAGCTAACACCAGTCATTTATATT

>SKP2_probe11

GATGATGCTTCAATTTCTTAATAGT

>SKP2_target

aaattgatgacttgttcgtatgttcaaaatgtaacaacaaaaaaagctaacaccagtcatttatattaactttttttttttaaatcaaaaattgttaatgttagaaacatactatgaagtgcctttatctgcttagacctaaggaagattttaaagttgggttgcacaggaaatgatgatgcttcaatttcttaatagttaaaaagtgctaaatactacttgaaattattgtttacagattagtgacaagagctggggttaggatccggttggactctgacatcggatgccctcaaacatacagaacttccaaactcaagtccagccataagctattttgccaacatgtcagagt

>SKP2_consensus

gaattccgggctgtagagcttgcncgcgcagtggggatggaacgttgctaggcttagcgggtctggctgctgggggcccgagcagcacgctcggagccgccgcgcgccaaagcgggaatctgggaggcgaagcagctctgcaggtttaatgcacgtattttaaactcccgggcctgcggacgctatgcacaggaagcacctccaggagattccagacctgagtagcaacgttgccaccagcttcacgtggggatgggattccagcaagacttctgaactgctgtcaggcatgggggtctccgccctggagaaagaggagcccgacagtgagaacatcccccaggaactgctctcaaacctgggccacccggagagccccccacggaaacggctgaagagcaaagggagtgacaaagactttgtgattgtccgcaggcctaagctaaatcgagagaactttccaggtgtttcatgggactcccttccggatgagctgctcttgggaatcttttcctgtctgtgcctccctgagctgctaaaggtctctggtgtttgtaagaggtggtatcgcctagcgtctgatgagtctctatggcagaccttagacctcacaggtaaaaatctgcacccggatgtgactggtcggttgctgtctcaaggggtgattgccttccgctgcccacgatcatttatggaccaaccattggnctgaacatttcagcccttttcgtgtacagcacatggacctatcgaactcagttatangaagtgtccaccctccacggcatactgtctcagtgttccaagttgcagaatctaagcctggaannnctgcggctttcggatcccattgtcaatactctcgcaaaaaactcaaatttagtgcgacttaaccttnctgggtgttctggattctctgaatttnccctgcagactttgctaagcagctgttccagactggatgagctgaacctctcctggtgttttnatttcactgaaaagcatgtacaggtggctgttgcgcatgtntcagagaccatcacccagctgaatcttagcggctacagaaagaatctccagaaatcagatctctctactttagttagaagatgccccaatcttgtccatctagacttaagtgatagtgtcatgctaaagaatgactgctttcaggaatttttccagctcaactacctccaacacctatcactcagtcggtgctatgatataatacctgaaactttacttgaacttggagaaattcccacactaaaaacactacaagtttttggaatcgtgccagatggtacccttcaactgttaaaggaagcccttcctcatctacagattaattgctcccatttcaccaccattgccaggccaactattggcaacaaaaagaaccaggagatatggggcatcaaatgccgactgacactgcaaaagcccagttgtctatgaagtatttattgcaggatggtgtctcttctttagaacagggaaaataggcaggaagcccaattgctggagtacttagctagttttattcttggttttccctttngcctntcattctgcaagtatactagggagcccattttgagagggaaaactatgaaatcttgctttttgaaatgattctaaaagcttctatcactgctttgctcttaagagccaaagttgtaggccttttgaaatnttaggagagtgagcctataatttcaagataccttaaagagcaaaatttgagccacctcttccaagtgcccttcttactaagtctattcagaatcaagcttaaaaattaccaccagcaaacaatcttcatagcccatataacttttatctatttaattttatagtatngctttataagacagcttagaagaacaataagctattngtatnatgagctgaacaaaaagagaatcataggatagtagcgtctgaggccatcttttctaggaataggaaagagaaaaatgtatttgaattttgcctttagatttgaaattaggttaatagaaataagtaaccccatgtaattcaccttaaaacttaacaaaagaccaaacattacaaaacccagaggtatagaatcaatataggatttgaaggcccagcagacagttttctatgacaggttaatctgaagtatcctgtaatgttcattaagttactgtgtttccagaatctaaattagatgagaaatataattgtggttttctaacttgataatcaaattatgttaacatgggtcctttagcttttaaaatgacttgcttngttttagaaaggtggtattaatccactctctattcttgaaaattnggnnggnagaattctgaagttgcctgctgttttcctttagcgctgaggttcttaaggttacttttatattactctggaatcaagtattttaaattgtattttttttttaaatgatctctcagcaataattgtttgaaactatccatatataaggttatcagacctacagttccctaagaggaactgcatgttctcttcaatcagaaatatacagtagaagcaggtatatcttccatgcagtttcagtagtaagcactacttatacctacataagagttaaaatccagatgtgggnaccttttgataccatcagtgatatatatttttttaaactggtacagagaagtgaaaagattaaattctacttctatttttctttttttttttttgganacggagtctcgctctgtcaccaaggnccggantngcagnggtgcgantctcggctcactgncaagctccgcctcccaggttcacgtcattctcctgcctnagcctcccgattagctgggactacnggcgcccaccaccacgcccggctaatttttnngtnnttttnagtagagacggggtttcaccatgttagccaggatggtctcaatctcctgacctcatgatccgcccgtctnggcctcccaaagtgctgggnattacaggcatgagcaactgncgcccagccaaattctacttcttaaaaatcacaaaaactagtnttaaattgatgacttgttcgtatgttcaaaatgtaacaacaaaaaaagctaacaccagtcatttatattaactttttttttttaaatcaaaaattgttaatgttagaaacatactatgaagtgcctttatctgcttagacctaaggaagattttaaagttgggttgcacaggaaatgatgatgcttcaatttcttaatagttaaaaagtgctaaatactacttgaaattattgtttacagattagtgacaagagctggggttaggatccggttggactctgacatcggatgccctcaaacatacagaacttccaaactcaagtccagccataagctattttgccaacatgtcagagtaatctgtatttttgtatgtgatttctacttttatagacttgttttaaaacaataaaacacatttttataaaaatgannnnnnaaaaaaaaaa

>DPP3_probe1

AAACGTTCTCACCAAATCCAATGCT

>DPP3_probe2

ATACGAGGCGTCAGCTGCTGGCCTC

>DPP3_probe3

AGGAGCTTGGACCTTGGTACTACCT

>DPP3_probe4

GATGCCCGATTCTGGAAGGGCCCCA

>DPP3_probe5

CAGACCAAGGCTGCAAGTGGCCCTC

>DPP3_probe6

GCTTACCATCCTGTCTACCAGATGA

>DPP3_probe7

CTCTGTGATCTCATTTCATCTGCAC

>DPP3_probe8

GTGGCACGTGACAGCTAGGGTTCAA

>DPP3_probe9

TGAGCGTTTCCCAGAGGATGGACCC

>DPP3_probe10

TGAGGGTGGTGACACAACCCCTTCC

>DPP3_probe11

TCATCTGCACTGCCATACGTGGAGT

>DPP3_target

atacgaggcgtcagctgctggcctcatccgatccttctctgagcgtttcccagaggatggacccgagttggaggagatcctcacacagctggccacagccgatgcccgattctggaagggccccagtgaggccccatctggccaagcttgaggaagatgtgtggccttgcccccaattccatcagaccaaggctgcaagtggccctccattcgtgtgtgtatttaggggctggggagggggaggggcaggagcttggaccttggtactacctcagctgagggtggtgacacaaccccttccatttgtcagcactttccagcctgccaattgcttcccctctgtgatctcatttcatctgcactgccatacgtggagtgagcaagacagggcttaccatcctgtctaccagatgaggaaatggcagttctgagaagtcactggtctagatcccgcaggtggcacgtgacagctagggttcaaaacgttctcaccaaatccaatgct

>DPP3_exemplar

ggccctcgggccaagattcggcacgaggcgaacggagcagctgctgcagcagggcccatggcggacacccagtacatcctgcccaatgacatcggcgtgtctagcctggactgccgtgaggccttccgcctgctgtcacccacagagcgcctctatgcctaccacctgtcccgtgccgcctggtacggaggcctggctgtgctgcttcagacctcccctgaggccccctacatctatgctctgctcagccgcctcttccgcgcccaggaccccgaccagctgcgccaacatgccctggctgaaggccttaccgaggaggagtatcaggcgttcctggtctatgccgcgggtgtttactccaacatgggcaactacaagtcctttggtgacaccaagtttgttcccaacttgcccaaggaaaagctggaacgggtgatcctagggagtgaggctgctcagcagcacccagaagaagtcaggggcctctggcatacctgcggggagcttatgttctctctggagccaaggcttcgacacctcggactggggaaggagggaatcaccacctatttctctgggaattgtaccatggaagatgccaaattggcccaggactttctggactcacagaacctcagtgcctacaacacccggctcttcaaagaggtcgatggagaagggaagccctactacgaggtgcggctggcttctgtgcttggctcagagccttccctggactctgaggtgacttccaagctgaagagctatgaattccggggaagccctttccaggtgacccggggggactacgcgcccatcctccagaaggtggtggagcagctggagaaagccaaggcctatgcagccaacagccaccaggggcagatgctggcccagtatatagagagcttcacccagggctccatcgaggcccacaagaggggctcccgcttctggatccaggacaaaggccccatcgtggagagttacatcgggttcatcgagagctaccgcgacccctttggttcccgaggagaatttgaaggtttcgtagctgtggtgaacaaggccatgagtgccaagtttgagcggctggtggcgagcgcagagcagctgctgaaggagctgccctggcccccaacctttgagaaggacaagttcctcacccctgacttcacctccctggatgttctcaccttcgctggctccggcatccctgccggcatcaacatccccaactacgatgatctgaggcagacggaaggctttaagaacgtgtcgctggggaatgtgctggctgtggcctacgccacgcagcgggagaagcttacctttctggaggaggatgacaaggacctgtacatcctctggaaggggccctccttcgatgtgcaggtgggcctgcacgagctgctgggccatggcagtggcaagctcttcgtacaggacgaaaaaggagcattcaactttgaccaggaaacagtgatcaacccagagacgggcgagcagattcagagctggtatcggagcggggagacctgggatagcaagttcagcaccatcgcctccagctacgaagagtgccgggctgagagcgtgggtctctacctctgtctccacccgcaagtgctggagatctttggctttgagggggctgatgcggaggacgtgatctacgtgaactggctcaacatggttcgggccgggctgctcgctctggagttctacacacctgaggccttcaactggcgacaggcccatatgcaggcccggtttgtgatcctgagagtcttgctggaggctggcgagggactcgttaccatcactcccaccacaggctccgatgggcgcccagatgcccgggtccgcctcgaccgcagcaagatccggtctgtgggcaagcctgctctagagcgcttcctgcggagacttcaggtgctgaagtccacaggggatgtggccggagggcgggccctgtacgaggggtatgcaacggtcactgatgcgccccccgagtgcttcctcaccctcagggacacggtgctgctgcgtaaggaatctcggaagctcattgttcagcccaacactcaccttgaaggctcagacgtgcagcttctggaatacgaggcgtcagctgctggcctcatccgatccttctctgagcgtttcccagaggatggacccgagttggaggagatcctcacacagctggccacagccgatgcccgattctggaagggccccagtgaggccccatctggccaagcttgaggaagatgtgtggccttgcccccaattccatcagaccaaggctgcaagtggccctccattcgtgtgtgtatttaggggctggggagggggaggggcaggagcttggaccttggtactacctcagctgagggtggtgacacaaccccttccatttgtcagcactttccagcctgccaattgcttcccctctgtgatctcatttcatctgcactgccatacgtggagtgagcaagacagggcttaccatcctgtctaccagatgaggaaatggcagttctgagaagtcactggtctagatcccgcaggtggcacgtgacagctagggttcaaaacgttctcaccaaatccaatgctcctcacatattaattttataaccagacaaataaatattagagacaaccaccaaaaaaaaaaaaaa

>TYMP_probe1

CCTGTGCTCGGGAAGTCCCGCAGAA

>TYMP_probe2

CCTTGGCCGCTTCGAGCGGATGCTG

>TYMP_probe3

CCGCTTCGAGCGGATGCTGGCGGCG

>TYMP_probe4

TGGCCCGAGCCCTGTGCTCGGGAAG

>TYMP_probe5

CCGAGCCCTGTGCTCGGGAAGTCCC

>TYMP_probe6

TGCTCGGGAAGTCCCGCAGAACGCC

>TYMP_probe7

CTGCTGGTCGACGTGGGTCAGAGGC

>TYMP_probe8

CTGGTCGACGTGGGTCAGAGGCTGC

>TYMP_probe9

GCCCGCCAGACTTAAGGGACCTGGT

>TYMP_probe10

CAGGCCCGCCAGACTTAAGGGACCT

>TYMP_probe11

ACTTAAGGGACCTGGTCACCACGCT

>TYMP_target

caggcccgccagacttaagggacctggtcaccacgctcgggggcgccctgctctggctcagcggacacgcggggactcaggctcagggcgctgcccgggtggccgcggcgctggacgacggctcggcccttggccgcttcgagcggatgctggcggcgcagggcgtggatcccggtctggcccgagccctgtgctcgggaagtcccgcagaacgccggcagctgctgcctcgcgcccgggagcaggaggagctgctggcgcccgcagatggcaccgtggagctggtccgggcgctgccgctggcgctggtgctgcacgagctcggggccgggcgcagccgcgctggggagccgctccgcctgggggtgggcgcagagctgctggtcgacgtgggtcagaggctgc

>TYMP_exemplar

gccccgccgccggcagtggaccgctgtgcgcgaaccctgaaccctacggtcccgacccgcgggcgaggccgggtacctgggctgggatccggagcaagcgggcgagggcagcgccctaagcaggcccggagcgatggcagccttgatgaccccgggaaccggggccccacccgcgcctggtgacttctccggggaagggagccagggacttcccgacccttcgccagagcccaagcagctcccggagctgatccgcatgaagcgagacggaggccgcctgagcgaagcggacatcaggggcttcgtggccgctgtggtgaatgggagcgcgcagggcgcacagatcggggccatgctgatggccatccgacttcggggcatggatctggaggagacctcggtgctgacccaggccctggctcagtcgggacagcagctggagtggccagaggcctggcgccagcagcttgtggacaagcattccacagggggtgtgggtgacaaggtcagcctggtcctcgcacctgccctggcggcatgtggctgcaaggtgccaatgatcagcggacgtggtctggggcacacaggaggcaccttggataagctggagtctattcctggattcaatgtcatccagagcccagagcagatgcaagtgctgctggaccaggcgggctgctgtatcgtgggtcagagtgagcagctggttcctgcggacggaatcctatatgcagccagagatgtgacagccaccgtggacagcctgccactcatcacagcctccattctcagtaagaaactcgtggaggggctgtccgctctggtggtggacgttaagttcggaggggccgccgtcttccccaaccaggagcaggcccgggagctggcaaagacgctggttggcgtgggagccagcctagggcttcgggtcgcggcagcgctgaccgccatggacaagcccctgggtcgctgcgtgggccacgccctggaggtggaggaggcgctgctctgcatggacggcgcaggcccgccagacttaagggacctggtcaccacgctcgggggcgccctgctctggctcagcggacacgcggggactcaggctcagggcgctgcccgggtggccgcggcgctggacgacggctcggcccttggccgcttcgagcggatgctggcggcgcagggcgtggatcccggtctggcccgagccctgtgctcgggaagtcccgcagaacgccggcagctgctgcctcgcgcccgggagcaggaggagctgctggcgcccgcagatggcaccgtggagctggtccgggcgctgccgctggcgctggtgctgcacgagctcggggccgggcgcagccgcgctggggagccgctccgcctgggggtgggcgcagagctgctggtcgacgtgggtcagaggctgcgccgtgggaccccctggctccgcgtgcaccgggacggccccgcgctcagcggcccgcagagccgcgccctgcaggaggcgctcgtactctccgaccgcgcgccattcgccgccccctcgcccttcgcagagctcgttctgccgccgcagcaataaagctcctttgccgcgaaa

>SNRPA1_probe1

GGTTGCTGCAGTCTGGTCAGATCCC

>SNRPA1_probe2

AGCTGACGGCGGAGCTGATCGAGCA

>SNRPA1_probe3

GTGGGCCATCTCCAGGGGATGTAGA

>SNRPA1_probe4

GTCAGATCCCTGGCAGAGAACGCAG

>SNRPA1_probe5

TAGCAAATGCTTCAACTCTGGCTGA

>SNRPA1_probe6

TGATCGAGCAGGCGGCGCAGTACAC

>SNRPA1_probe7

AAGGTTCCGCAAGTCAGAGTACTGG

>SNRPA1_probe8

TGGCATCTCTCAAATCGCTGACTTA

>SNRPA1_probe9

TCCAGGTGCTGGTTTGCCAACTGAC

>SNRPA1_probe10

TCCGGGGGTGATCTGAACCCTCTGG

>SNRPA1_probe11

AACGCAGATCAGGGCCCACTGATGA

>SNRPA1_target

agctgacggcggagctgatcgagcaggcggcgcagtacaccaacgcggtgcgcgaccgggagctggacctccgggggtgatctgaaccctctggcatctctcaaatcgctgacttacctaagtatcctaagaaatccggtaaccaataagaagcattacagattgtatgtgatttataaggttccgcaagtcagagtactggatttccagaaagtgaaactaaaattttaatccaggtgctggtttgccaactgacaaaaagaaaggtgggccatctccaggggatgtagaagcaatcaagaatgccatagcaaatgcttcaactctggctgaagtggagaggctgaaggggttgctgcagtctggtcagatccctggcagagaacgcagatcagggcccactgatga

>SNRPA1_consensus

gcaggatggtcaagctgacggcggagctgatcgagcaggcggcgcagtacaccaacgcggtgcgcgaccgggagctggacctccgggggtgatctgaaccctctggcatctctcaaatcgctgacttacctaagtatcctaagaaatccggtaaccaataagaagcattacagattgtatgtgatttataaggttccgcaagtcagagtactggatttccagaaagtgaaactaaaattttaatccaggtgctggtttgccaactgacaaaaagaaaggtgggccatctccaggggatgtagaagcaatcaagaatgccatagcaaatgcttcaactctggctgaagtggagaggctgaaggggttgctgcagtctggtcagatccctggcagagaacgcagatcagggcccactgatgatggtgaagaagagatggaagaagacacagtcacaaacgggtcctgagcagtgaggcagatga

>DHCR7_probe1

TCTCCAGCGAGGAGGTCTCAGTCCC

>DHCR7_probe2

GCGTGCACGGTGTTGAACTGGGACA

>DHCR7_probe3

CTATGCTCCGAGTAGAGTTCATCTT

>DHCR7_probe4

CTCCTTGGTAGCGTGCACGGTGTTG

>DHCR7_probe5

TGACTGTGCAGACTCTGGCTCGAGC

>DHCR7_probe6

AGGTGTAGGCAGGTGGGCTCTGCTT

>DHCR7_probe7

GAAAGGGGCTTTCATGTCGTTTCCT

>DHCR7_probe8

TCTTCCTCATCCCTAGGGTGTTGTG

>DHCR7_probe9

GAACTCTTTTTAAACTCTATGCTCC

>DHCR7_probe10

GTCTGCAGACCTCAGAGAGGTCCCA

>DHCR7_probe11

GAACTGGGACACTGGGGAGAAAGGG

>DHCR7_target

ctccttggtagcgtgcacggtgttgaactgggacactggggagaaaggggctttcatgtcgtttccttcctgctcctgctgcacagctgccaggagtgctctgcctggagtctgcagacctcagagaggtcccagcactggctgtggctttcaggtgtaggcaggtgggctctgcttcccgattccctgtgagcgcccaccctctcgaaagaattttctgtcttgccctgtgactgtgcagactctggctcgagcaacccggggaacttcaccctcaggggcctctccacaccttctccagcgaggaggtctcagtcccagcctcgggagggcacctccttttctgtgctttcttccctgaggcattcttcctcatccctagggtgttgtgtagaactctttttaaactctatgctccgagtagagttcatctt

>DHCR7_exemplar

gagcagcgcgcgcaagcaggccaggggaaggtgggcgcaggtgaggggccgaggtgtgcgcaggactttagccggttgagaaggatcaagcaggcatttggagcacaggtgtctagaaacttttaaggggccggttcaagaaggaaaagttcccttctgctgtgaaactatttggcaagaggctggagggcccaatggctgcaaaattgcaacccaacattcccaaagccaagagtctagatggcgtcaccaatgacagaaccgcatctcaagggcagtggggccgtgcctgggaggtggactggttttcactggcgagcgtcatcttcctactgctgttcgcccccttcatcgtctactacttcatcatggcttgtgaccagtacagctgcgccctgaccggccctgtggtggacatcgtcaccggacatgctcggctctcggacatctgggccaagactccacctataacgaggaaagccgcccagctctataccttgtgggtcaccttccaggtgcttctgtacacgtctctccctgacttctgccataagtttctacccggctacgtaggaggcatccaggagggggccgtgactcctgcaggggttgtgaacaagtatcagatcaacggcctgcaagcctggctcctcacgcacctgctctggtttgcaaacgctcatctcctgtcctggttctcgcccaccatcatcttcgacaactggatcccactgctgtggtgcgccaacatccttggctatgccgtctccaccttcgccatggtcaagggctacttcttccccaccagcgccagagactgcaaattcacaggcaatttcttttacaactacatgatgggcatcgagtttaaccctcggatcgggaagtggtttgacttcaagctgttcttcaatgggcgccccgggatcgtcgcctggaccctcatcaacctgtccttcgcagcgaagcagcgggagctccacagccatgtgaccaatgccatggtcctggtcaacgtcctgcaggccatctacgtgattgacttcttctggaacgaaacctggtacctgaagaccattgacatctgccatgaccacttcgggtggtacctgggctggggcgactgtgtctggctgccttatctttacacgctgcagggtctgtacttggtgtaccaccccgtgcagctgtccaccccgcacgccgtgggcgtcctgctgctgggcctggtgggctactacatcttccgggtggccaaccaccagaaggacctgttccgccgcacggatgggcgctgcctcatctggggcaggaagcccaaggtcatcgagtgctcctacacatccgccgacgggcagaggcaccacagcaagctgctggtgtcgggcttctggggcgtggcccgccacttcaactacgtcggcgacctgatgggcagcctggcctactgcctggcctgtggcggtggccacctgctgccctacttctacatcatctacatggccatcctgctgacccaccgctgcctccgggacgagcaccgctgcgccagcaagtacggccgggactgggagcgctacaccgccgcagtgccttaccgcctgctgcctggaatcttctaagggcacgccctagggagaagccctgtggggctgtcaagagcgtgttctgccaggtccatgggggctggcatcccagctccaactcgaggagcctcagtttcctcatctgtaaactggagagagcccagcacttggcaggtgtccagtacctaatcacgctctgttccttgcttttgccttcaagggaattccgagtgtccagcactgccgtattgccagcacagacggattttctctaatcagtgtccctgggcaggaggatgacccagtcacctttactagtcctttggagacaatttacctgtattaggagcccaggccacgctacactctgcccacactggtgagcaggaggtcttcccacgccctgtcattaggctgcatttactcttgctaaataaaagtgggagtggggcgtgcgcgttatccatgtattgcctttcagctctagatccccctcccctgcctgctctgcagtcgtgggtggggcccgtgcgccgtttctccttggtagcgtgcacggtgttgaactgggacactggggagaaaggggctttcatgtcgtttccttcctgctcctgctgcacagctgccaggagtgctctgcctggagtctgcagacctcagagaggtcccagcactggctgtggctttcaggtgtaggcaggtgggctctgcttcccgattccctgtgagcgcccaccctctcgaaagaattttctgtcttgccctgtgactgtgcagactctggctcgagcaacccggggaacttcaccctcaggggcctctccacaccttctccagcgaggaggtctcagtcccagcctcgggagggcacctccttttctgtgctttcttccctgaggcattcttcctcatccctagggtgttgtgtagaactctttttaaactctatgctccgagtagagttcatctttatattaaacttcccctgttcaaaaaaaaaaaaaaaaa

>TFPT_probe1

AAAGTACCAGGCACTAGGTCGGCGC

>TFPT_probe2

GCGCTGCCGGGAGATCGAGCAGGTG

>TFPT_probe3

TGGCCCCGGTGCAGATTAAGGTTGA

>TFPT_probe4

CAGCCAGTTCACCATTGTGCTGGAG

>TFPT_probe5

GCCGAGCAGGAAATGCGCTGACTCC

>TFPT_probe6

CCTGGATTCCAGTTGGGTTTCTCGG

>TFPT_probe7

GCTGGACTCCTACGGGGATGACTAC

>TFPT_probe8

AACGAGCGGGTCCTGAACAGGCTCC

>TFPT_probe9

TCGGGGTCCAGACAAACTGCTGCCC

>TFPT_probe10

GGTTCCTCATGAGAGTGCTGGACTC

>TFPT_probe11

GGCGGCGCCAGCGGGAATTAAATCG

>TFPT_target

ggcggcgccagcgggaattaaatcgcagaaagtaccaggcactaggtcggcgctgccgggagatcgagcaggtgaacgagcgggtcctgaacaggctccatcaggtgcagaggataactcggaggctgcagcaggaacggaggttcctcatgagagtgctggactcctacggggatgactaccgggccagccagttcaccattgtgctggaggatgagggcagccagggcacggatgcccccaccccaggcaatgcggagaatgagcctccagagaaagagacactgtccccgcccagaaggactcctgcacccccagaacccggcagcccagcccccggtgaggggcccagtgggcggaagaggcggcgagtgccacgggatggacgccgagcaggaaatgcgctgactccagagctggccccggtgcagattaaggttgaggaagactttggctttgaagcagatgaggccctggattccagttgggtttctcggggtccagacaaactgctgccc

>TFPT_exemplar

ttagcacctggcactaggcggagagaggcggtaagccgcgaggaggaaaggactcacgtcccgctgtggaccgatcctgctaagcagagaatcgctgtggccggacgacggggcgtcgagacaagaagaaagacgttggcaactcccacaaggcctagggccacgtcccgccgtcctcggctgctgagcctgatgggacaagtagttttgcgaacggcttaacctactgattgaagaggtcggaagctctgaggcccggggcttccggaggtcgcggagatggaattggagcagagagaagggaccatggcagccgtgggctttgaggagttctcagcgccgccaggctcagagttggcgttgcctcccctatttggtggccacatcctggagagcgagctggagacggaagtggagtttgtgtcaggtggtctgggcggctcagggctccgggagcgagatgaagaggaagaggcagcccggggtcggcggcggcgccagcgggaattaaatcgcagaaagtaccaggcactaggtcggcgctgccgggagatcgagcaggtgaacgagcgggtcctgaacaggctccatcaggtgcagaggataactcggaggctgcagcaggaacggaggttcctcatgagagtgctggactcctacggggatgactaccgggccagccagttcaccattgtgctggaggatgagggcagccagggcacggatgcccccaccccaggcaatgcggagaatgagcctccagagaaagagacactgtccccgcccagaaggactcctgcacccccagaacccggcagcccagcccccggtgaggggcccagtgggcggaagaggcggcgagtgccacgggatggacgccgagcaggaaatgcgctgactccagagctggccccggtgcagattaaggttgaggaagactttggctttgaagcagatgaggccctggattccagttgggtttctcggggtccagacaaactgctgccctacccgaccctggccagcccagcctctgactgacgcatgcccaataaactgaccccacaccaaaaaaaa

>CTTN_probe1

TGTGTCTTTCCAGAAGGTCACGTGG

>CTTN_probe2

CAAAGATGGGGTGCCAAGACGGTGC

>CTTN_probe3

TCGCCCAGGATGACGCGGGGGCCGA

>CTTN_probe4

GTGGAAATGTCTCGGGACTTGGGTC

>CTTN_probe5

CGTGAACAGCCTTTTATCTCCAAGC

>CTTN_probe6

GAAACTCATCTCCTTCCTGAGGAGC

>CTTN_probe7

GAATTTCGTGAACAGCCTTTTATCT

>CTTN_probe8

CCAGGACACCGCTGTCCTGGCATTT

>CTTN_probe9

CAGCCTTTTATCTCCAAGCGGAAAG

>CTTN_probe10

TTCCTCATTGGATTACTGTGTTTTA

>CTTN_probe11

GAAGGTCACGTGGAAATGTCTCGGG

>CTTN_probe12

CTGGGAGACCGACCCTGATTTTGTG

>CTTN_probe13

AATCAGTCCCCAATGCCTGGAAATT

>CTTN_probe14

GCCTGGAAATTCCTCATTGGATTAC

>CTTN_probe15

CCTGAGGTGCATTTTCTCATCATCC

>CTTN_probe16

CATCCTTGCTTTACCACAATGAGCA

>CTTN_probe17

ATTTGTGGCCACTCACTTTGTAGGA

>MCM5_probe1

GCATCGCATGCAGCGCAAGGTTCTC

>MCM5_probe2

GAGGAAGGAGCTGTAGTGTCCTGCT

>MCM5_probe3

CTGGGAAGTGTGCTTTTGGCATCCG

>MCM5_probe4

CGGCGAGATCCAGCATCGCATGCAG

>MCM5_probe5

CCAGCATCGCATGCAGCGCAAGGTT

>MCM5_probe6

CTGCCTGCCATTGACAATGTTGCTG

>MCM5_probe7

GCGAGATCCAGCATCGCATGCAGCG

>MCM5_probe8

GTTCTGGGAAGTGTGCTTTTGGCAT

>MCM5_probe9

GAAGGAGCTGTAGTGTCCTGCTGCC

>MCM5_probe10

TCGCATGCAGCGCAAGGTTCTCTAC

>MCM5_probe11

TTGACAATGTTGCTGGGACCTCTGC

>MCM5_target

cggcgagatccagcatcgcatgcagcgcaaggttctctaccgcctcaagtgagtcgcgccgcctcactggactcatggactcgcccacgcctcgcccctcctgccgctgcctgccattgacaatgttgctgggacctctgcctccccactgcagccctcgaacttcccaggcaccctcctttctgccccagaggaaggagctgtagtgtcctgctgcctctgggcgcccgcctctagcgcggttctgggaagtgtgcttttggcatccg

>MCM5_consensus

ttttttttttacctgaacacaccgtggctttattattaacggatgccaaaagcacacttcccagaaccgcgctagaggcgggcgcccagaggcagcaggacactacagctccttcctctggggcagaaaggagggtgcctgggaagttcgagggctgcagtggggaggcagaggtcccagcaacattgtcaatggcaggcagcggcaggaggggcgaggcgtgggcgagtccatgagtccagtgaggcggcgcgactcacttgaggcggtagagaaccttgcgctgcatgcgatgctggatctcgccgcgccgcagcatgagctgnagcaccttgtggatggcgtgctccgggtntnncnagaatccaaccacctcntnctctcccctccccttncgtgggacagaaaaaatacccccatccctgctcctgatgaagatgtccctagggaaggaatgatggctagaaaag
