# Supplementary material for: A robust prognostic signature for hormone-positive node-negative breast cancer
Source: Genome Med. 2013 Oct 11;5(10):92. doi: 10.1186/gm496 (PMC3961800; doi:10.1186/gm496)
Supplement: Additional file 8 — Describes alignment of all reference probe sets to the reference genome. [file gm496-S8.zip › Add 9 and 10 (7)/1217714361945551_add10.pdf]

Appendix 5B

NACAP1(211445_x_at)

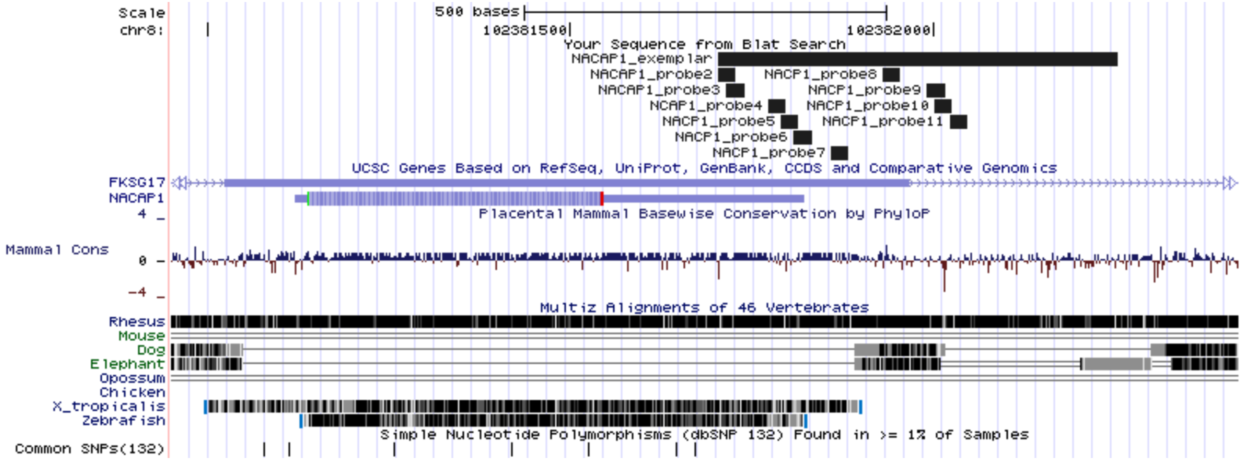

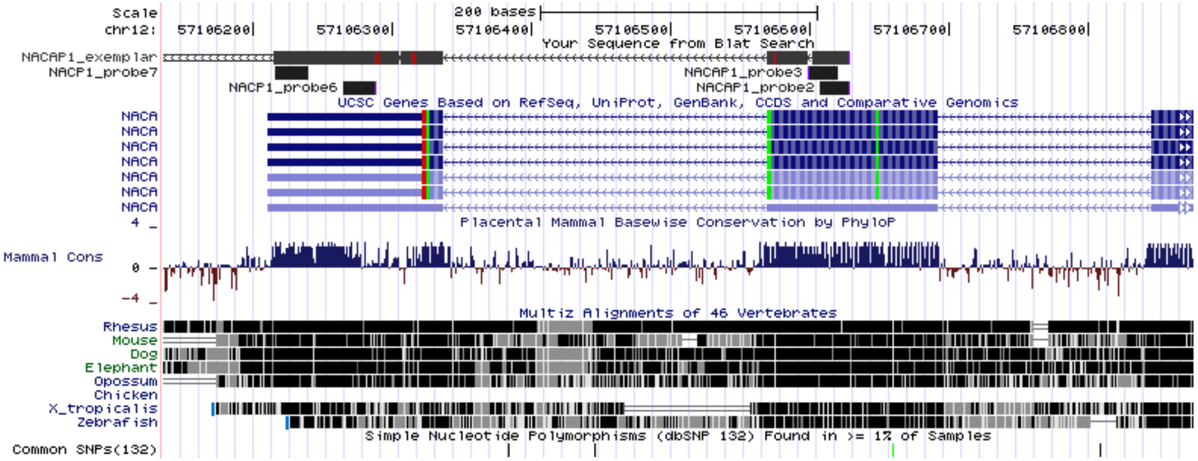

PTMA(216515_x_at)

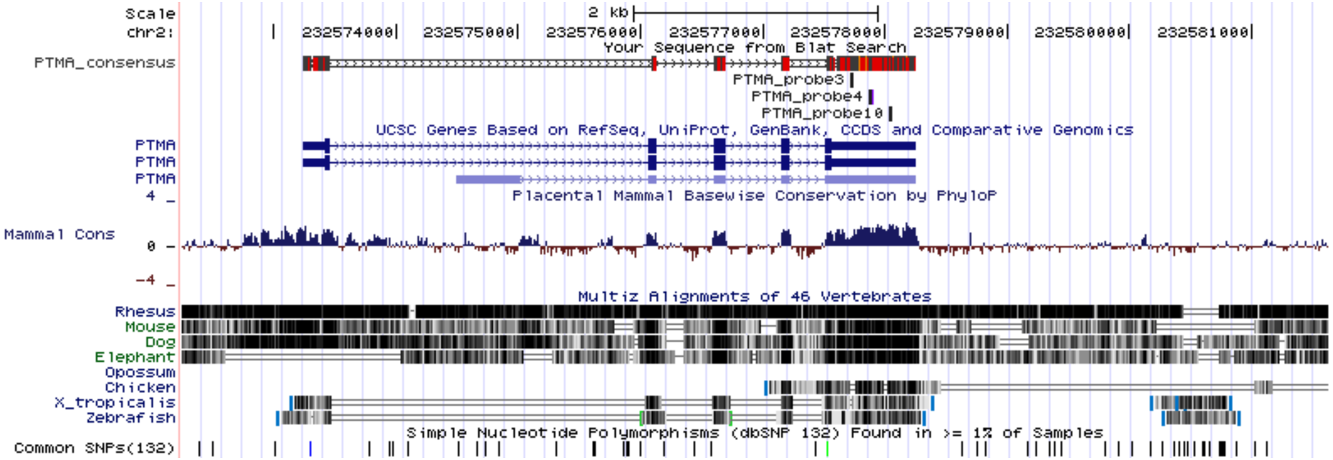

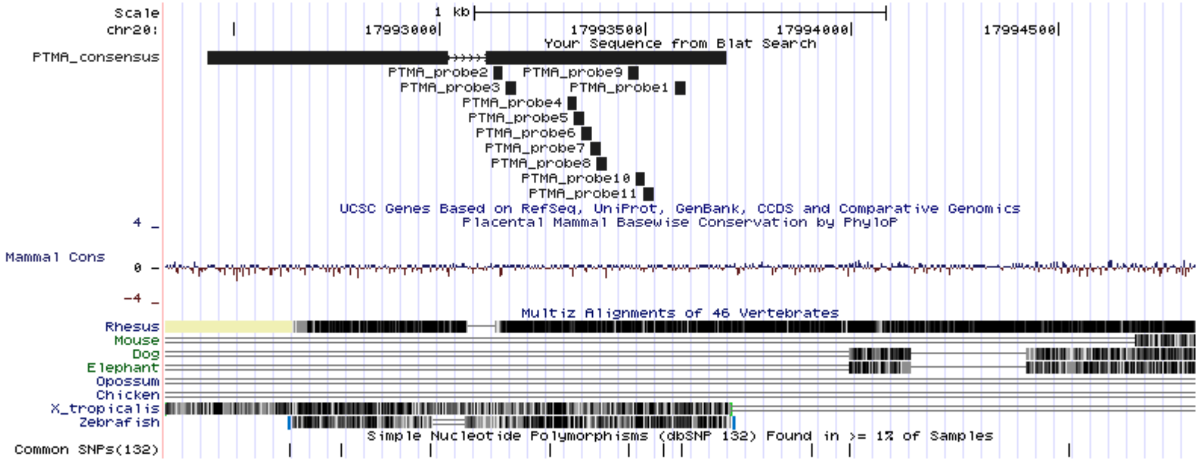

RPL7(217092_x_at)

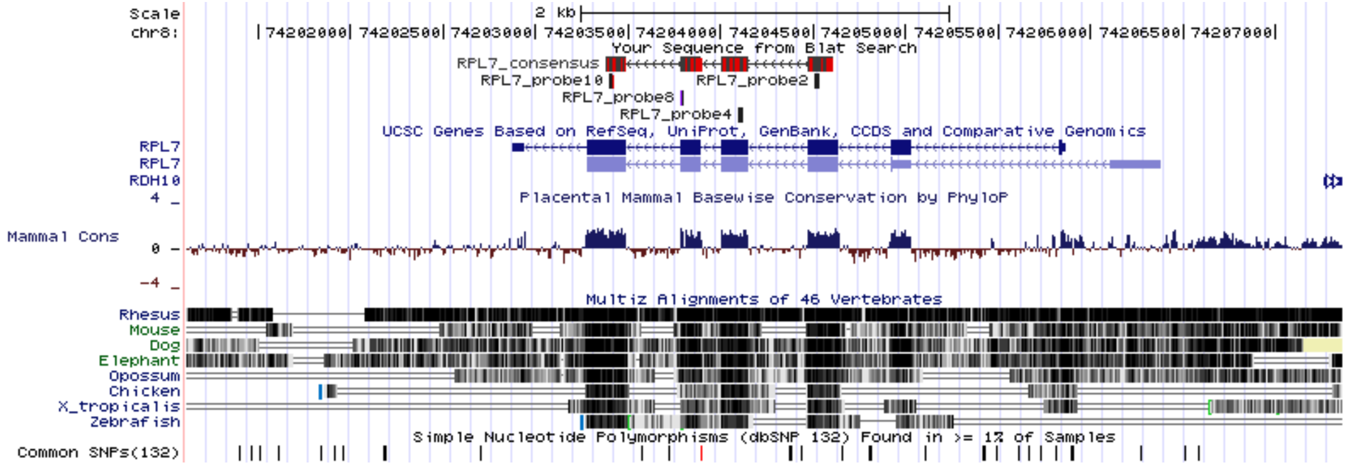

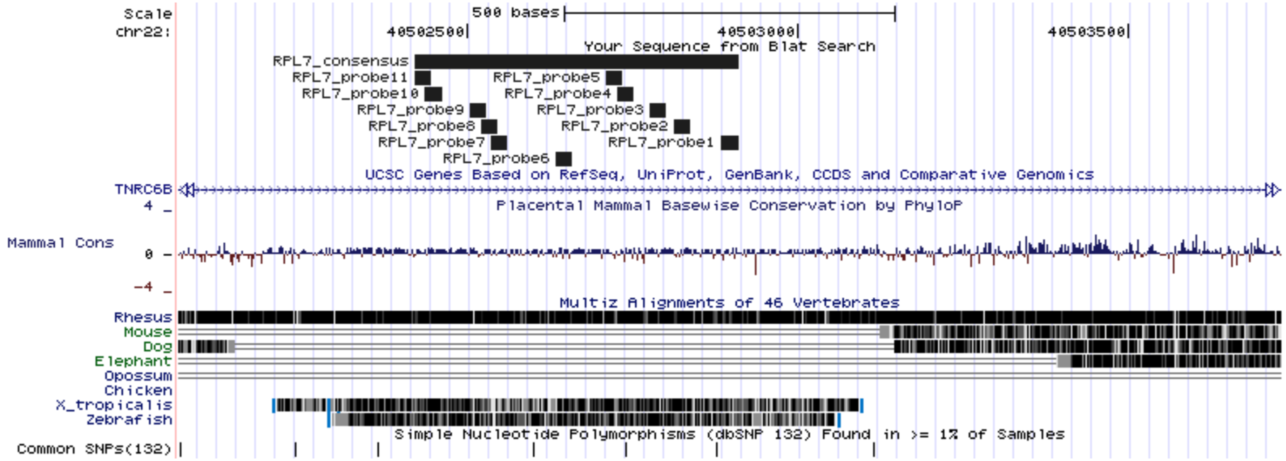

MYL12B(103910_at)

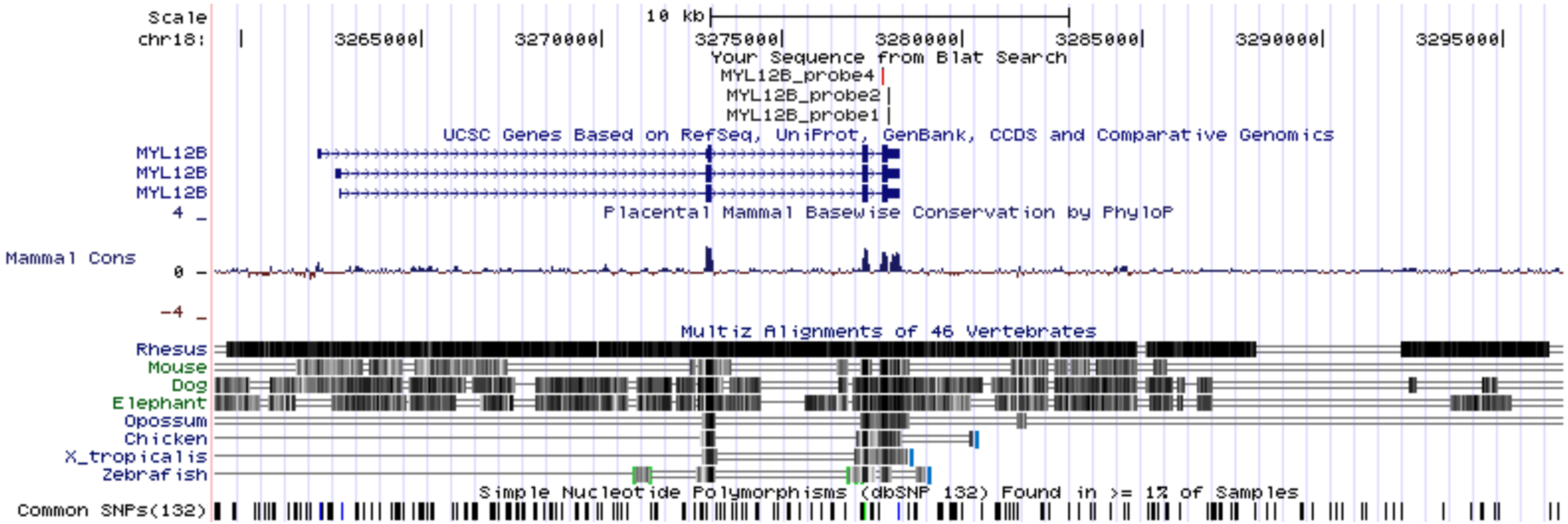

SFRS3(208672_s_at)

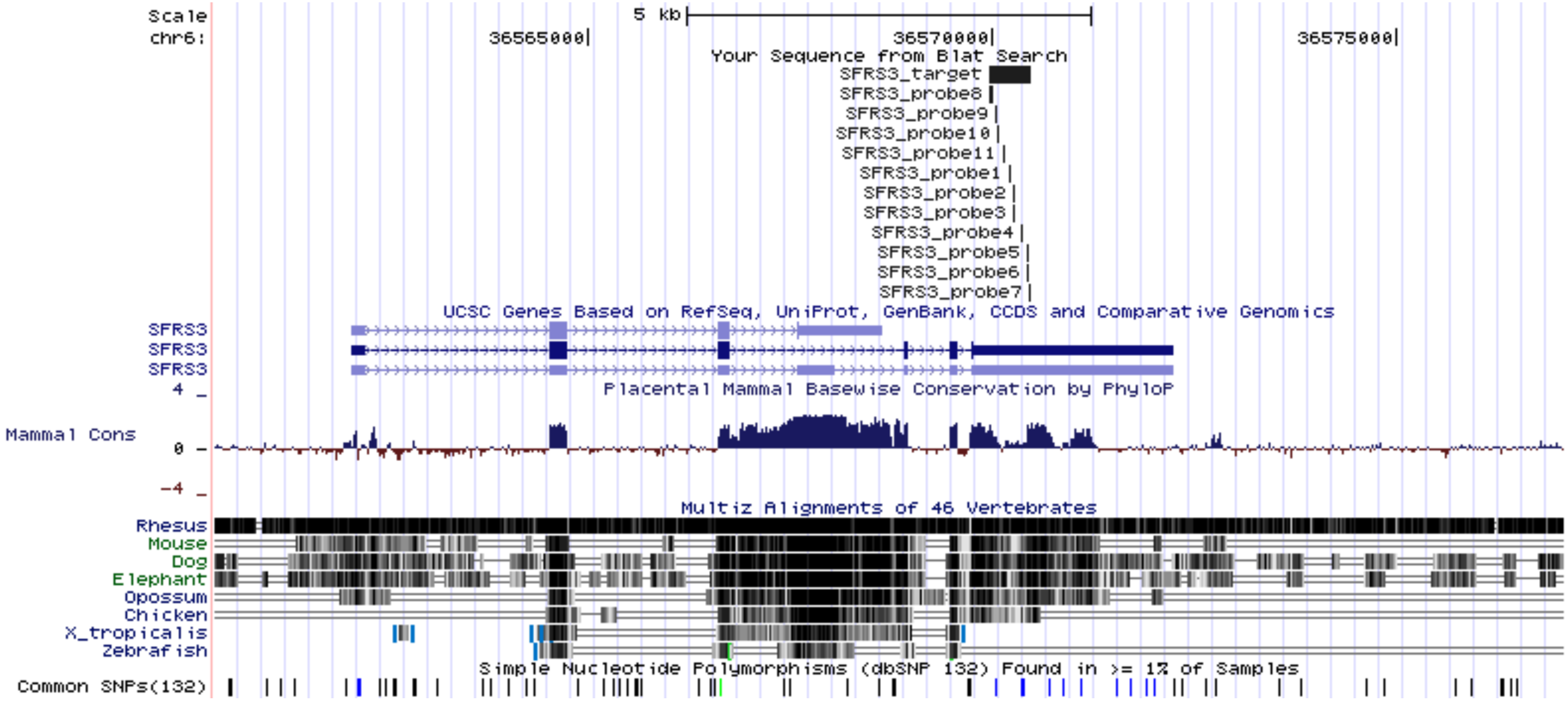

CLTA(200960_x_at)

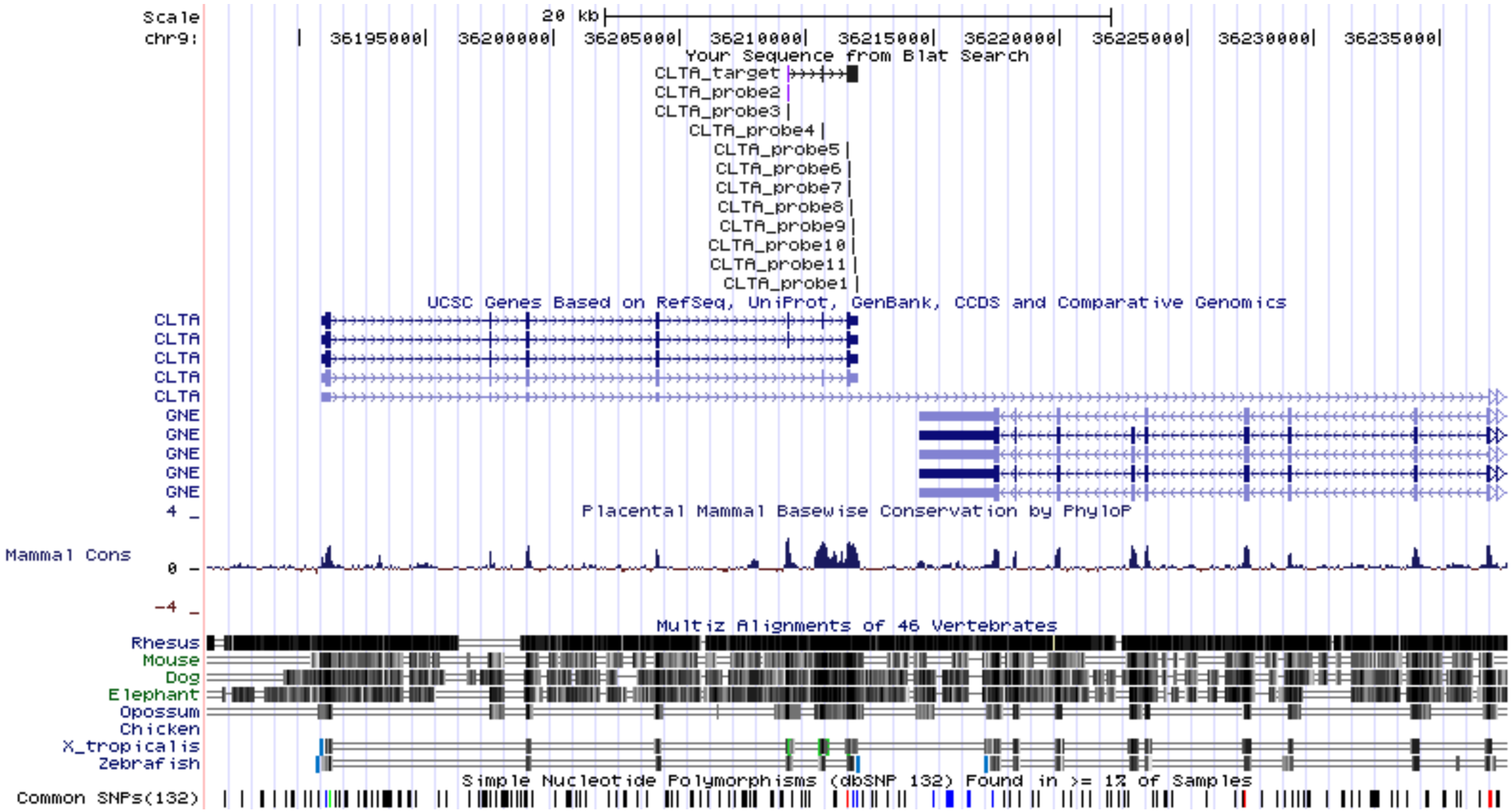

TRA2B(200893_at)

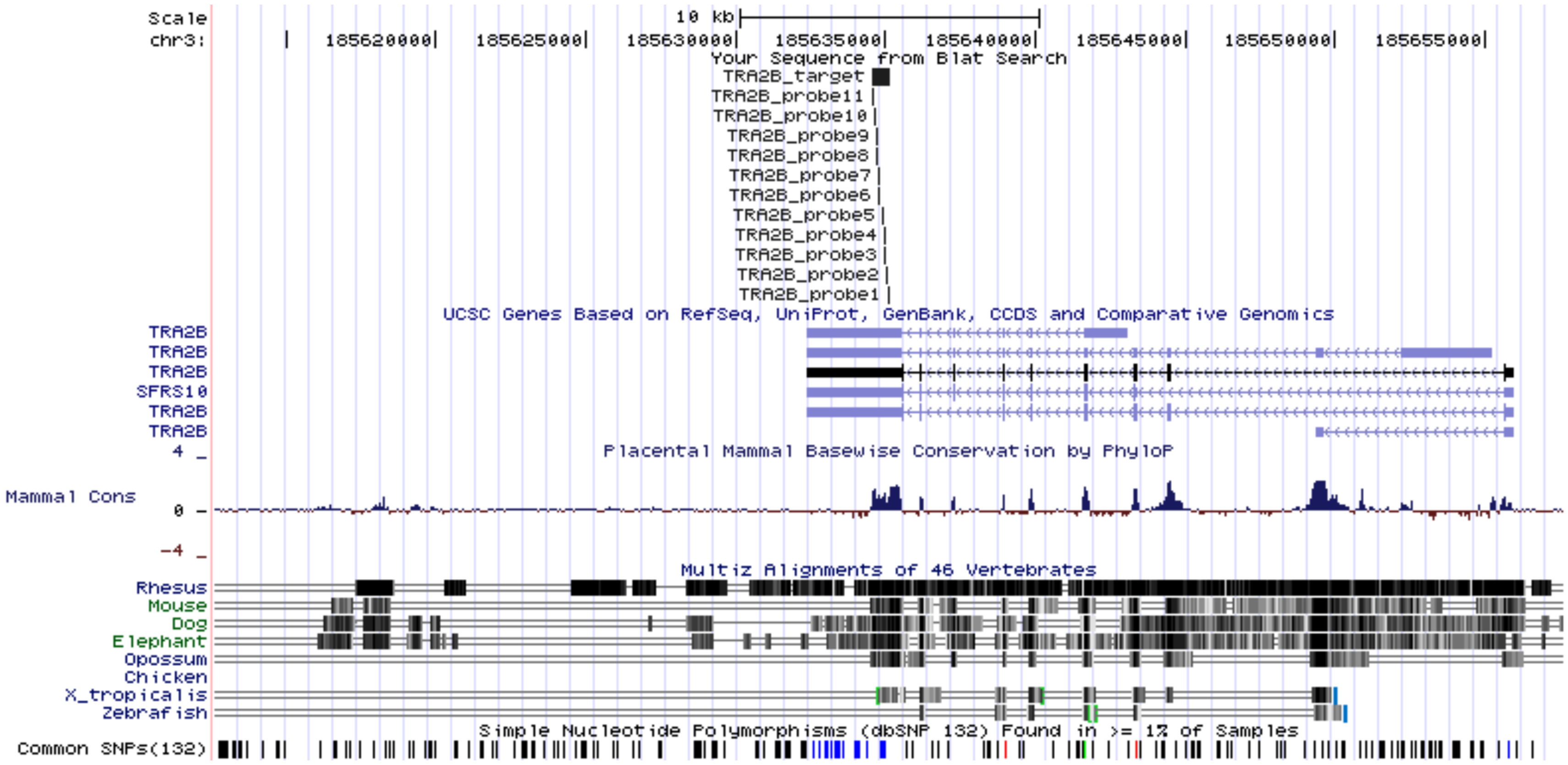

RPS2(217466_x_at)

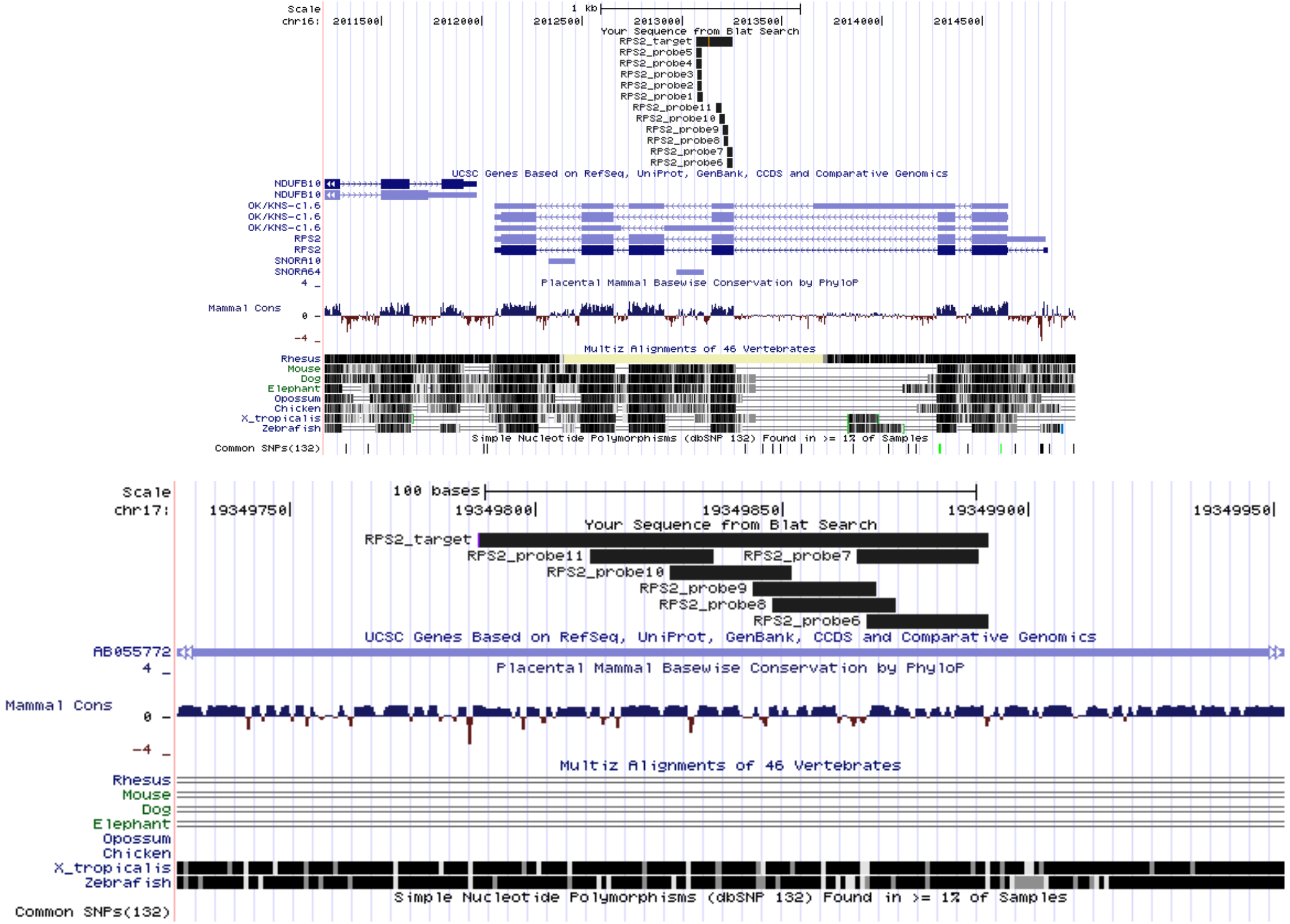

MTCH1(23787_at)

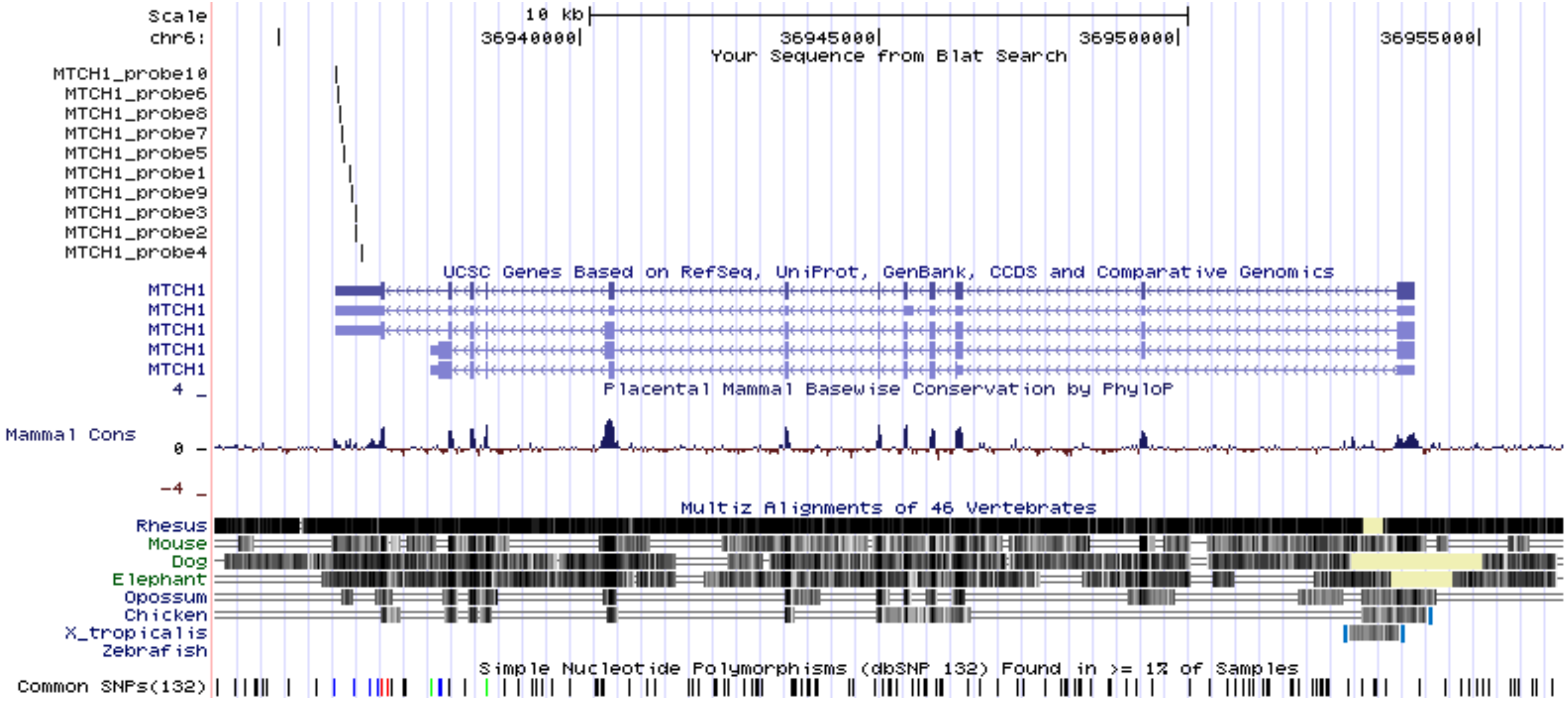

HDLBP(221767_x_at)

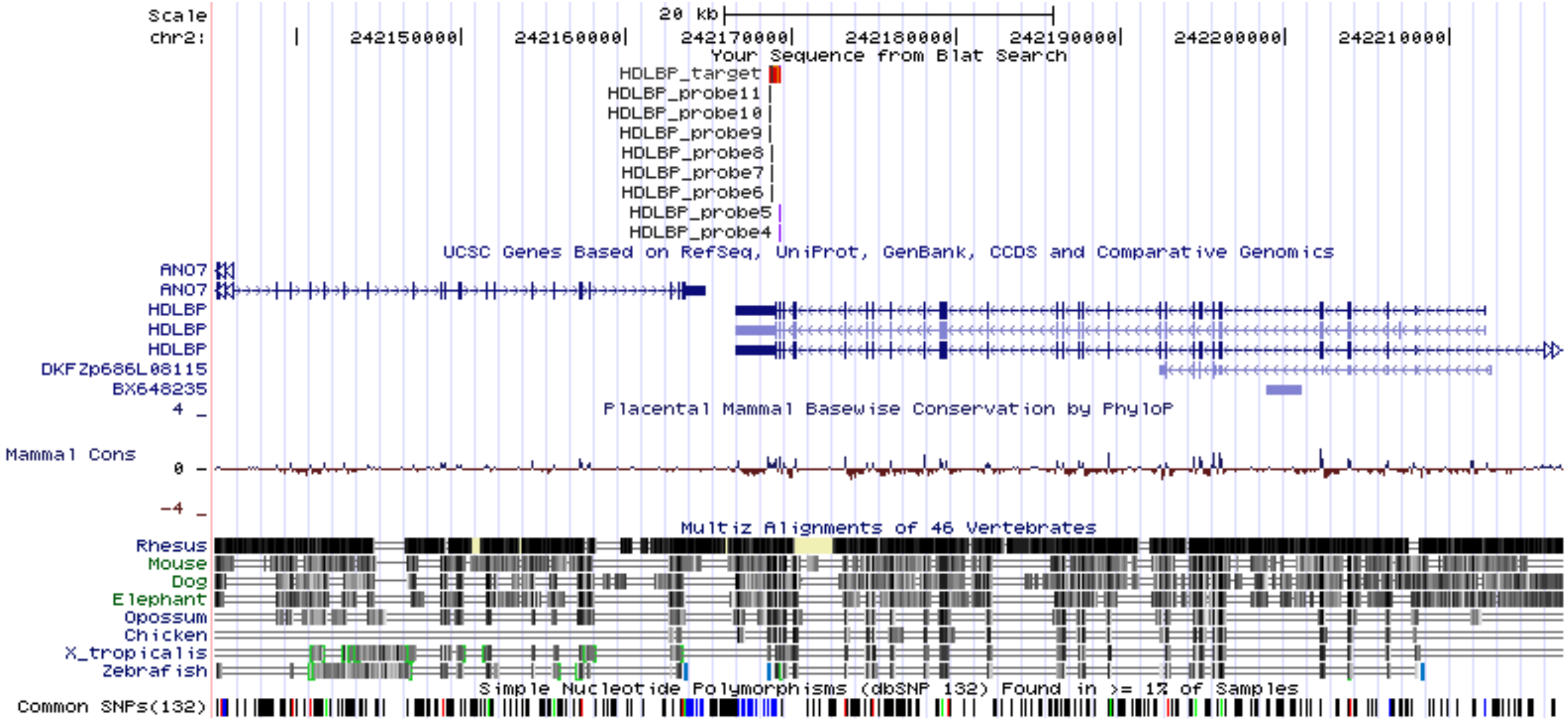

CYFIP1(23191_at)

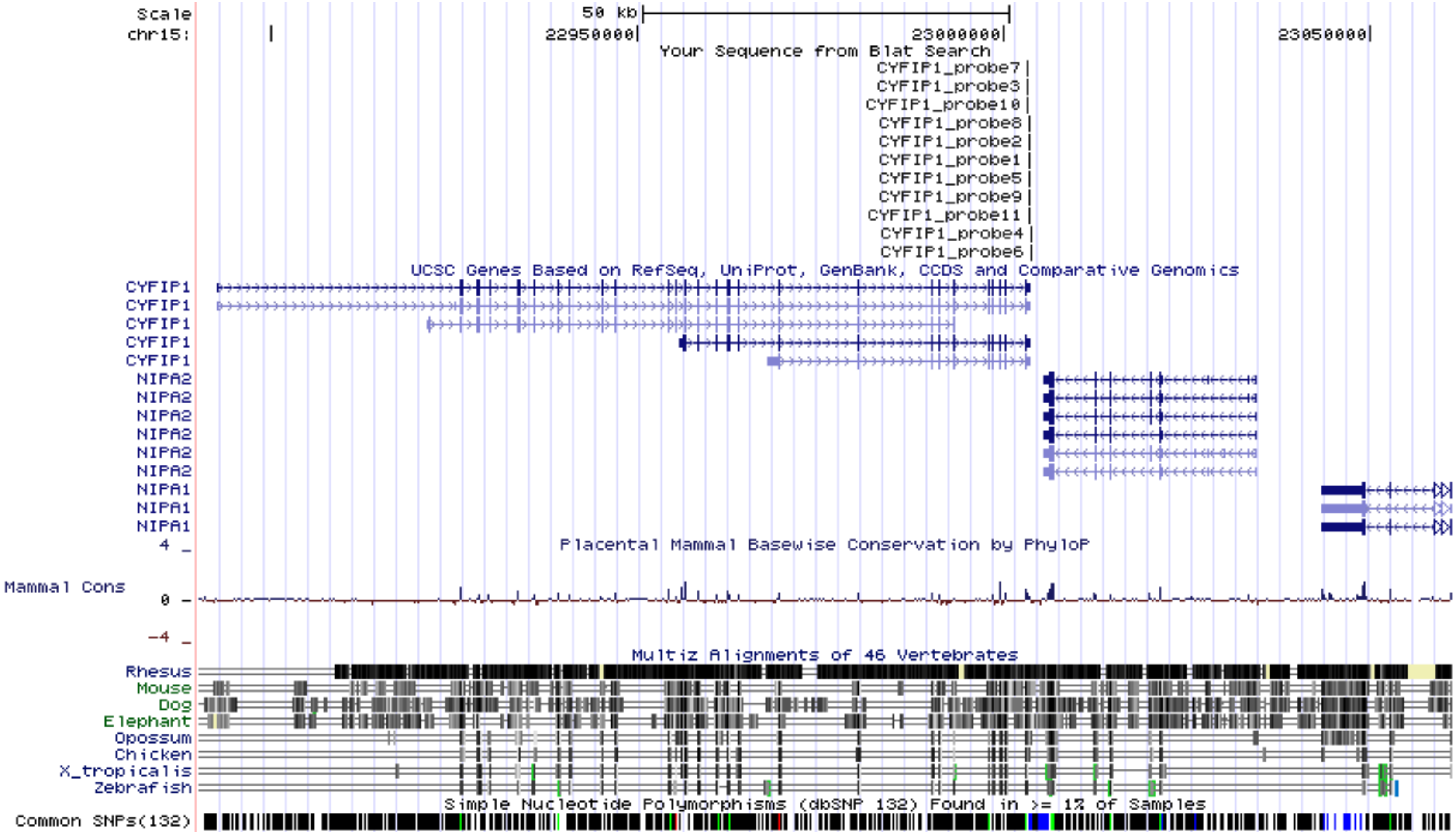

SUMO1(211069_s_at)

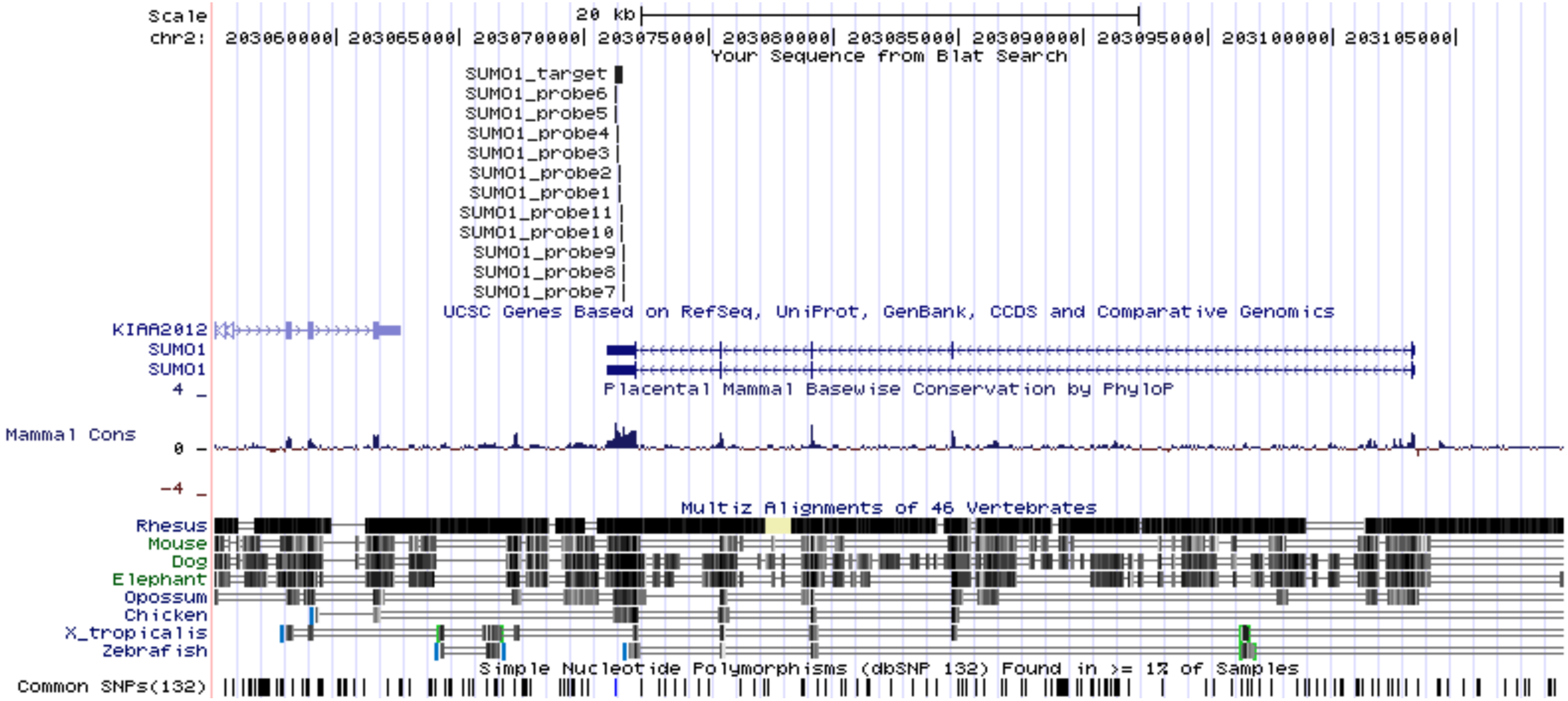

DHX15(201385_at)

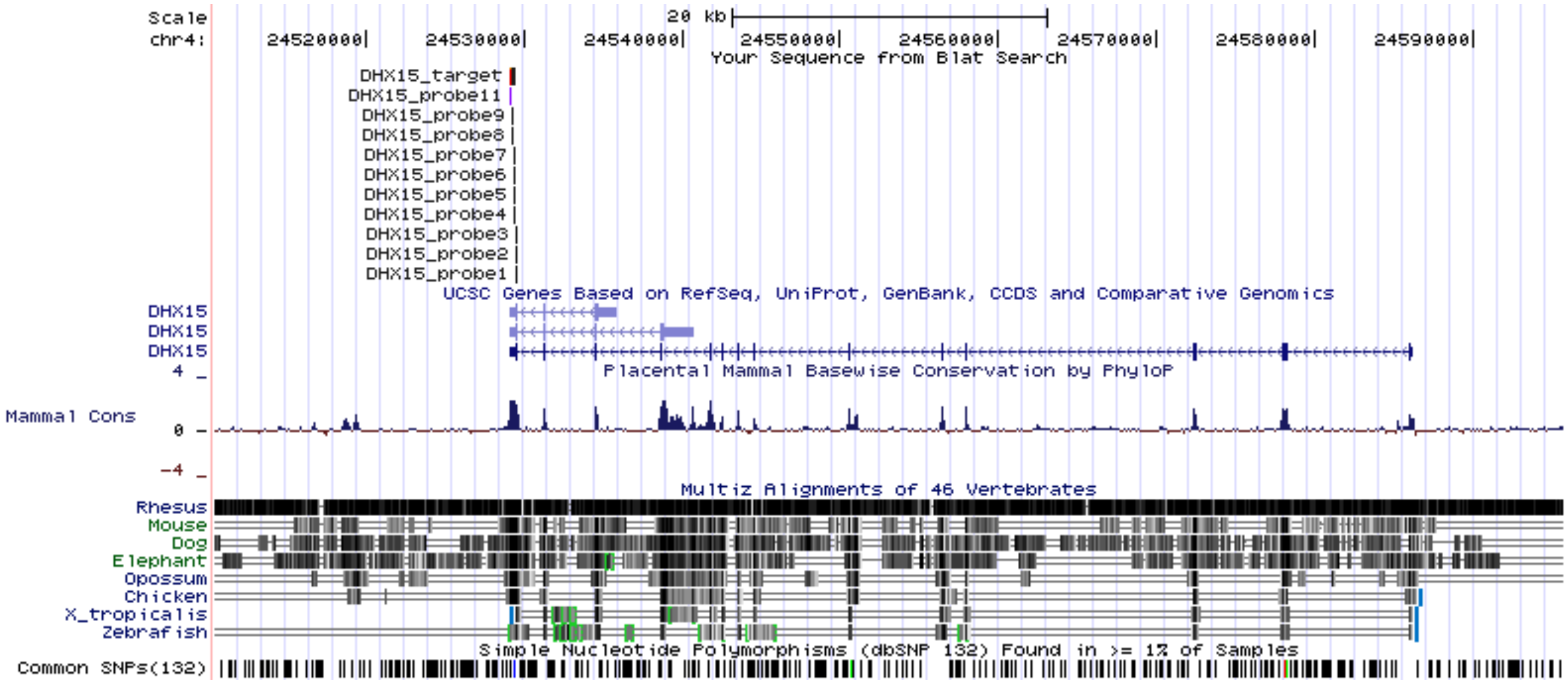

HNRNPC(200014_s_at)

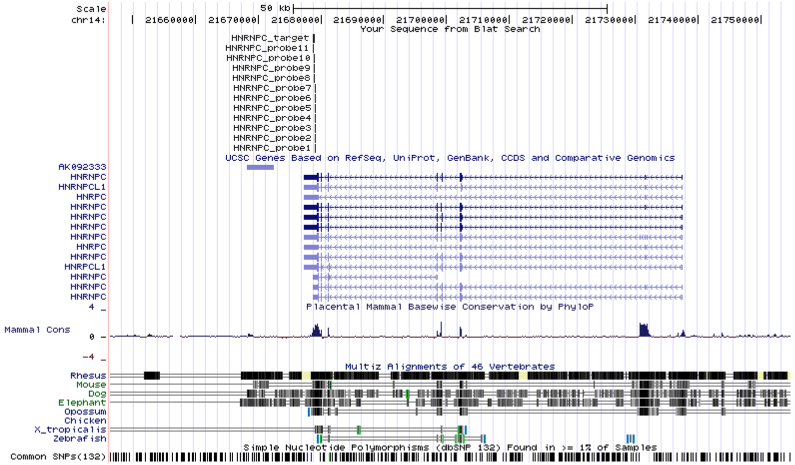

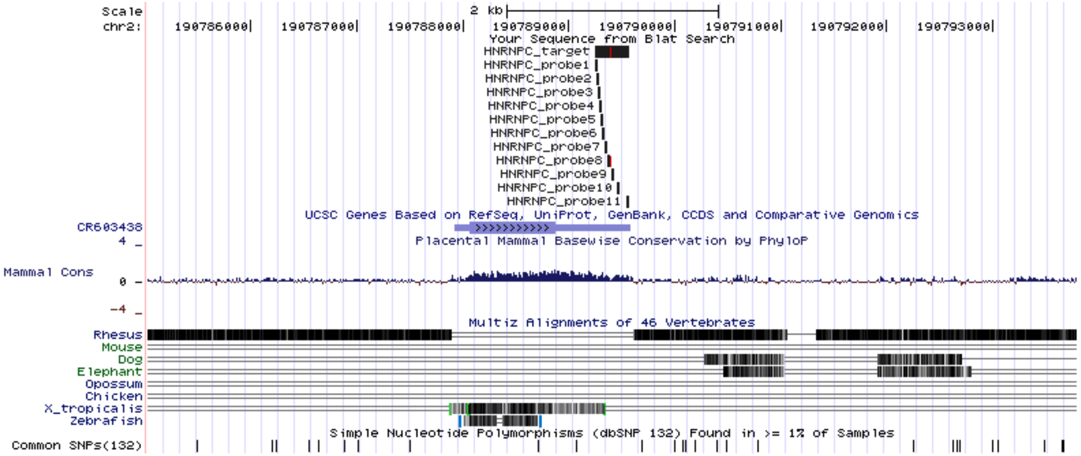

UBE2D3(200667_at)

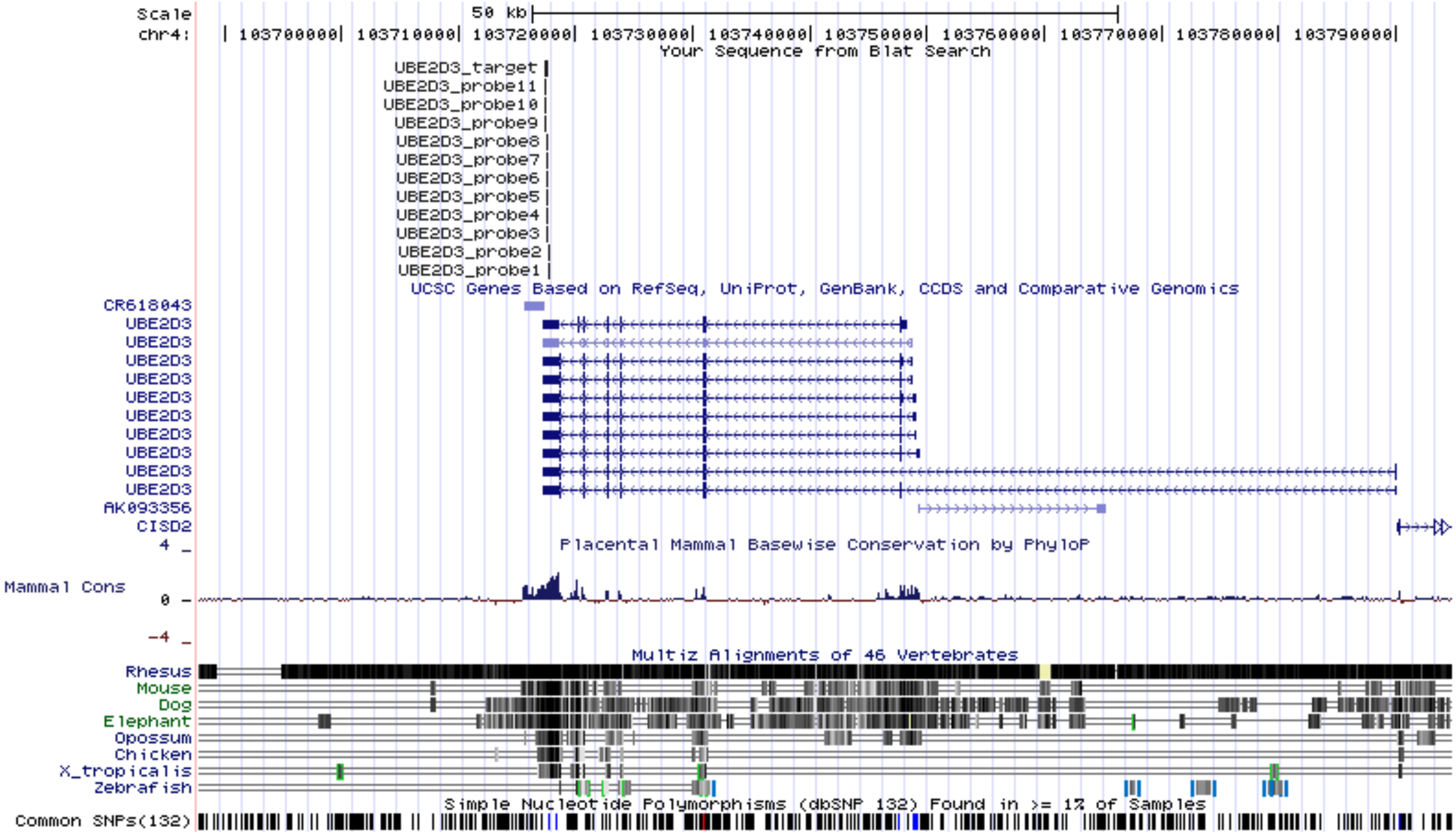

DAZAP2(9802_at)

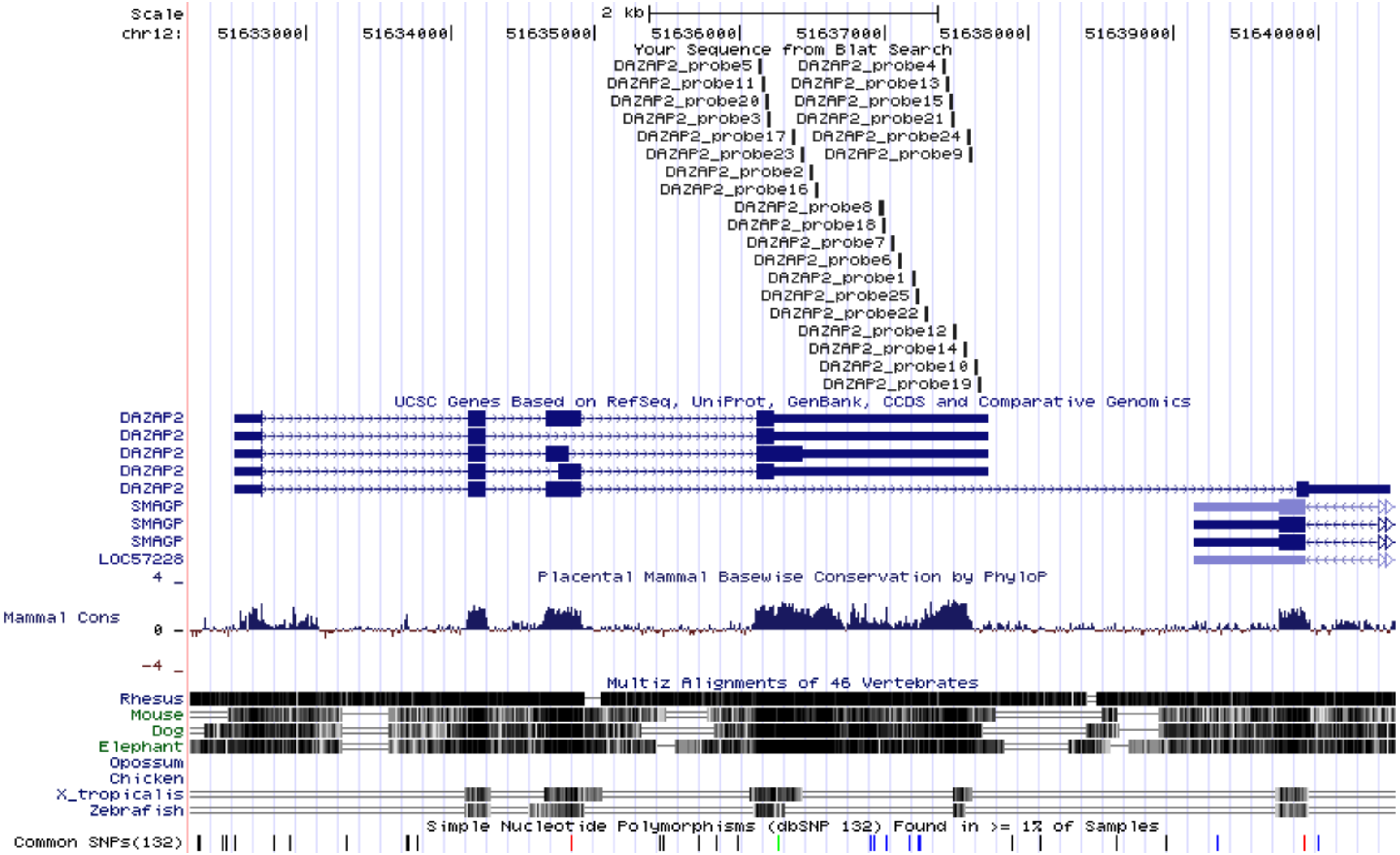

SNRNP200(200058_s_at)

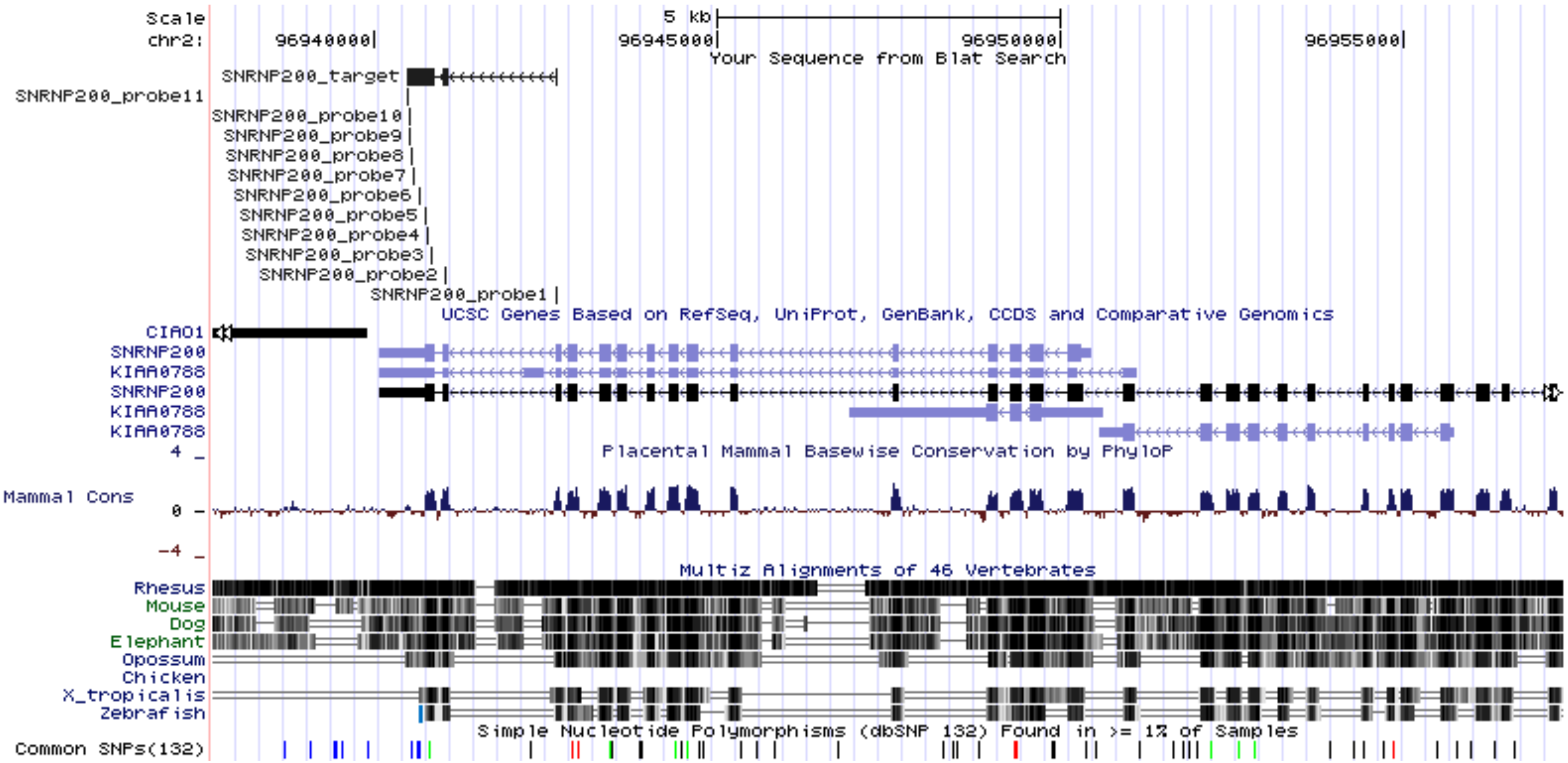

YTHDC1(91746_at)

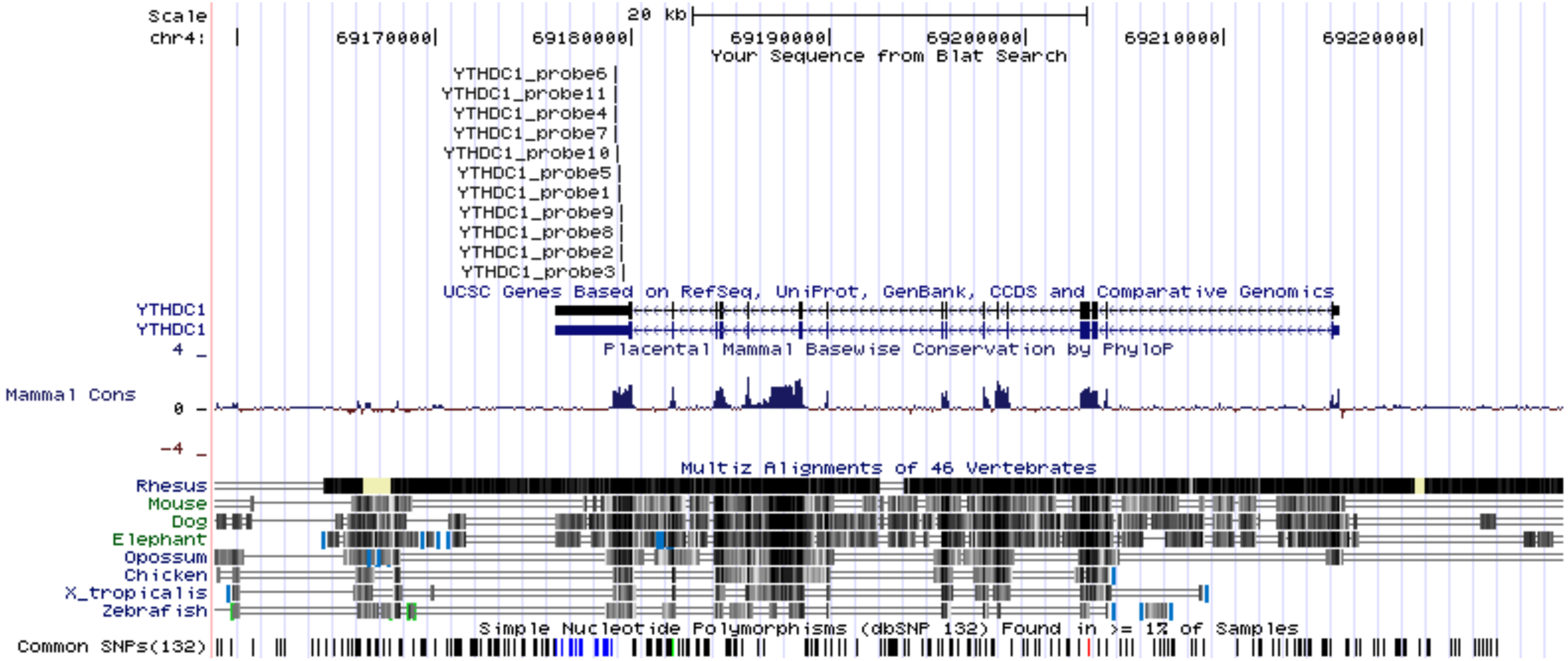

COPB1(1315_at)

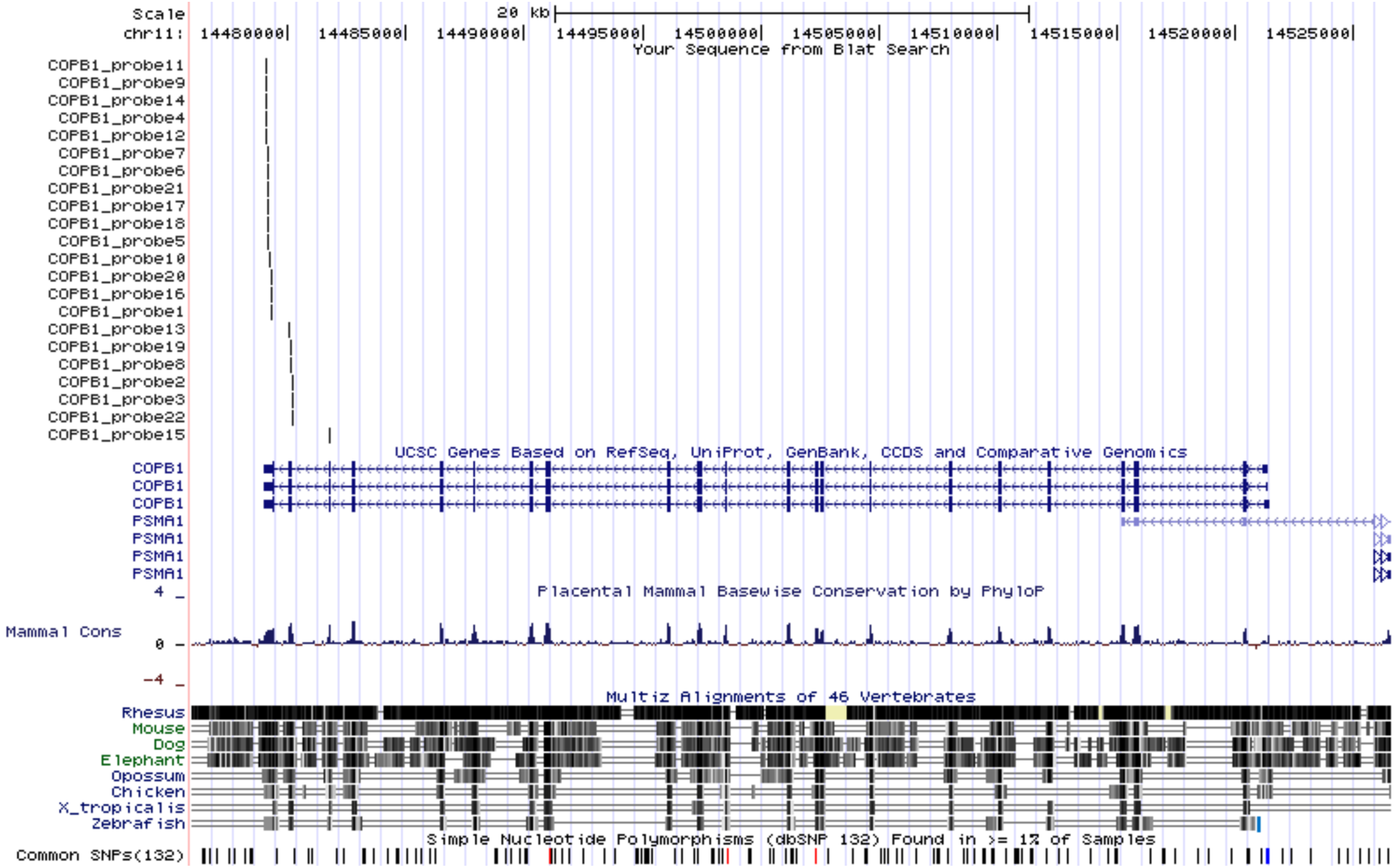

NDUFB8(4714_at)

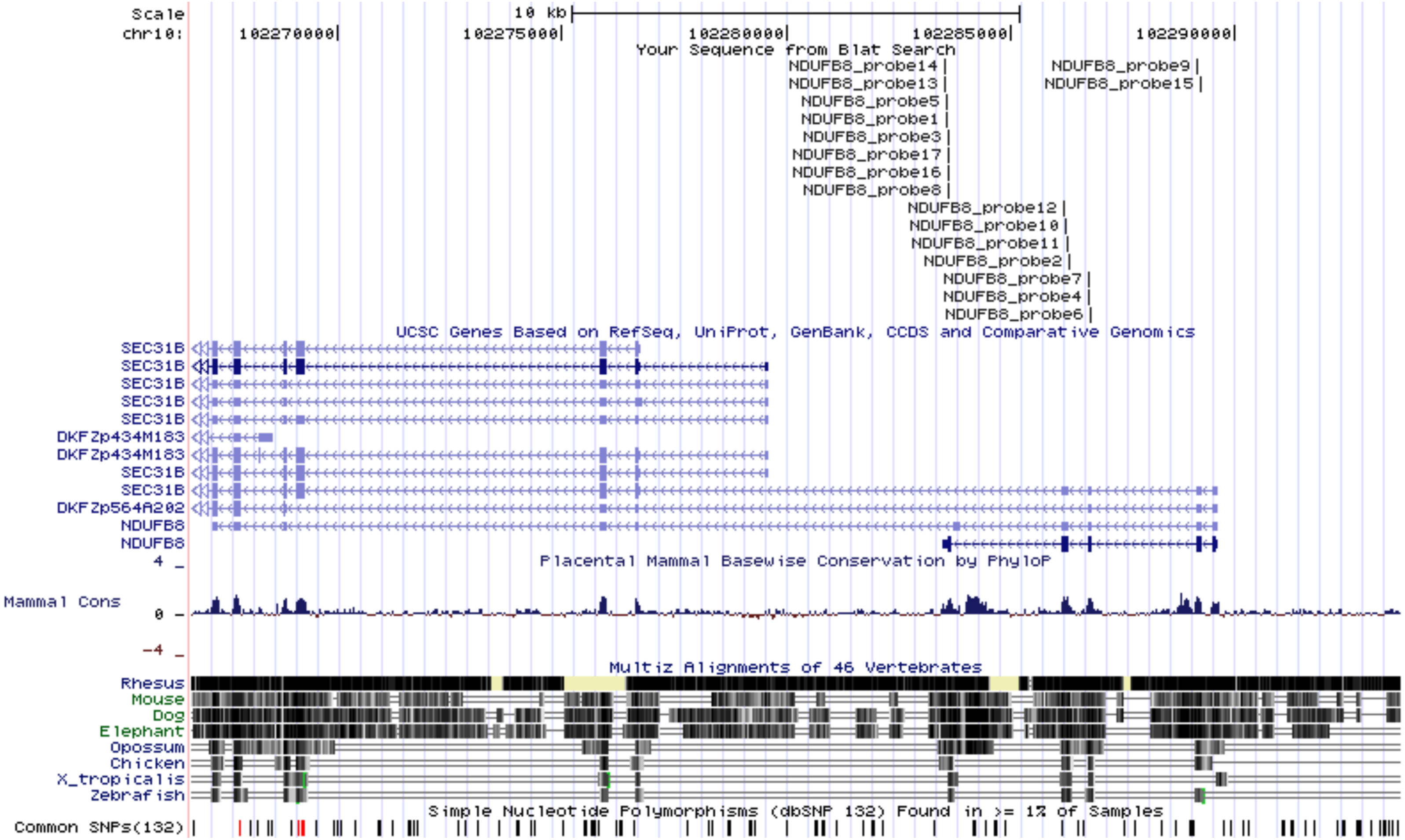

SET(40189_at)

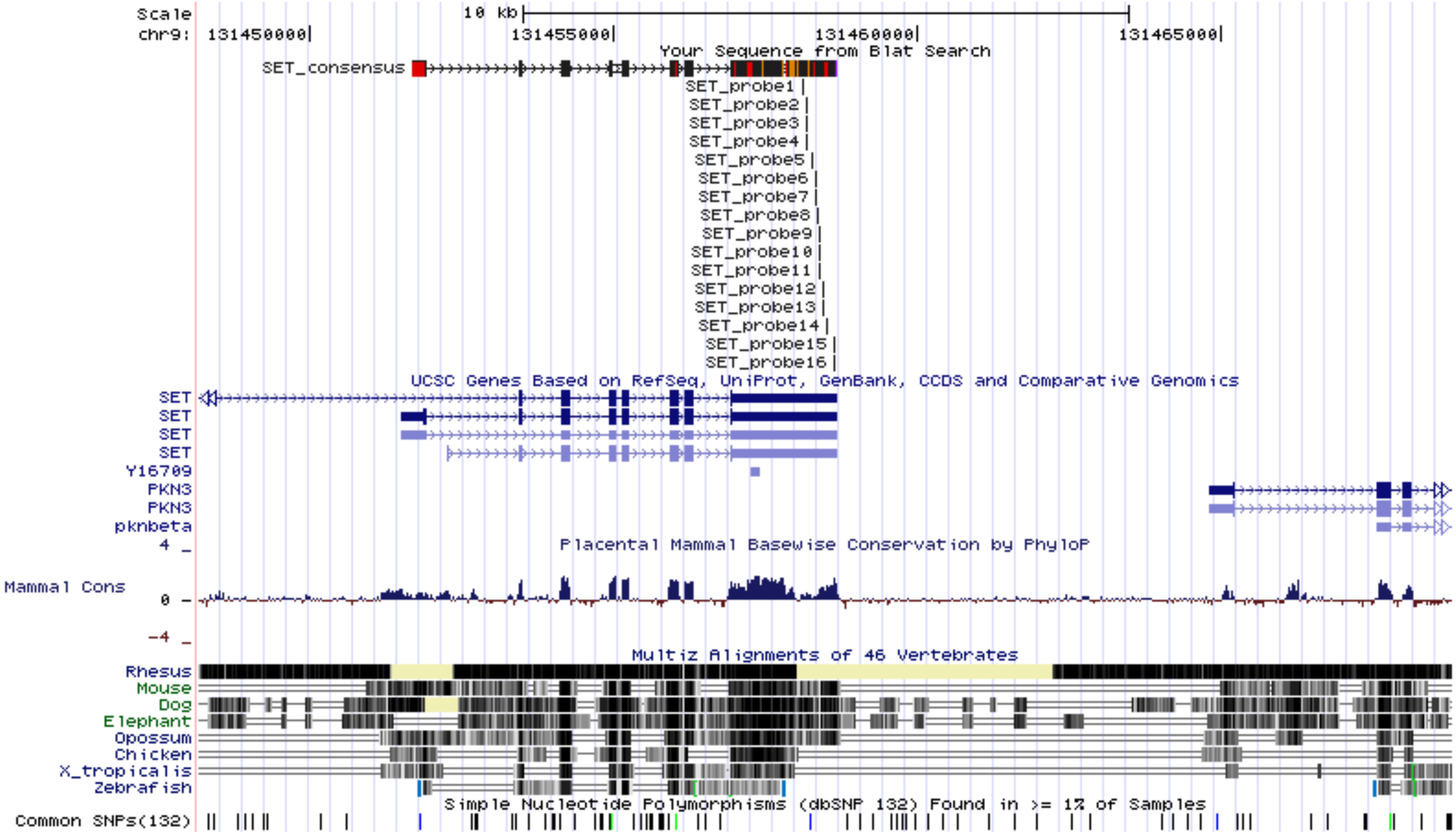
Scale
chr9:
10 kb
131450000
131455000
131460000
131465000
Your Sequence from Blat Search
SET_consensus
SET_probe1
SET_probe2
SET_probe3
SET_probe4
SET_probe5
SET_probe6
SET_probe7
SET_probe8
SET_probe9
SET_probe10
SET_probe11
SET_probe12
SET_probe13
SET_probe14
SET_probe15
SET_probe16
UCSC Genes Based on RefSeq, UniProt, GenBank, CCDS and Comparative Genomics
SET
SET
SET
SET
Y16709
PKN3
PKN3
pknbeta
Placental Mammal Basewise Conservation by PhyloP
4
Mammal Cons
0
-4
Multiz Alignments of 46 Vertebrates
Rhesus
Mouse
Dog
Elephant
Opossum
Chicken
X_tropicalis
Zebrafish
Simple Nucleotide Polymorphisms (dbSNP 132) Found in >= 1% of Samples
Common SNPs(132)

CELF1(221743_at)

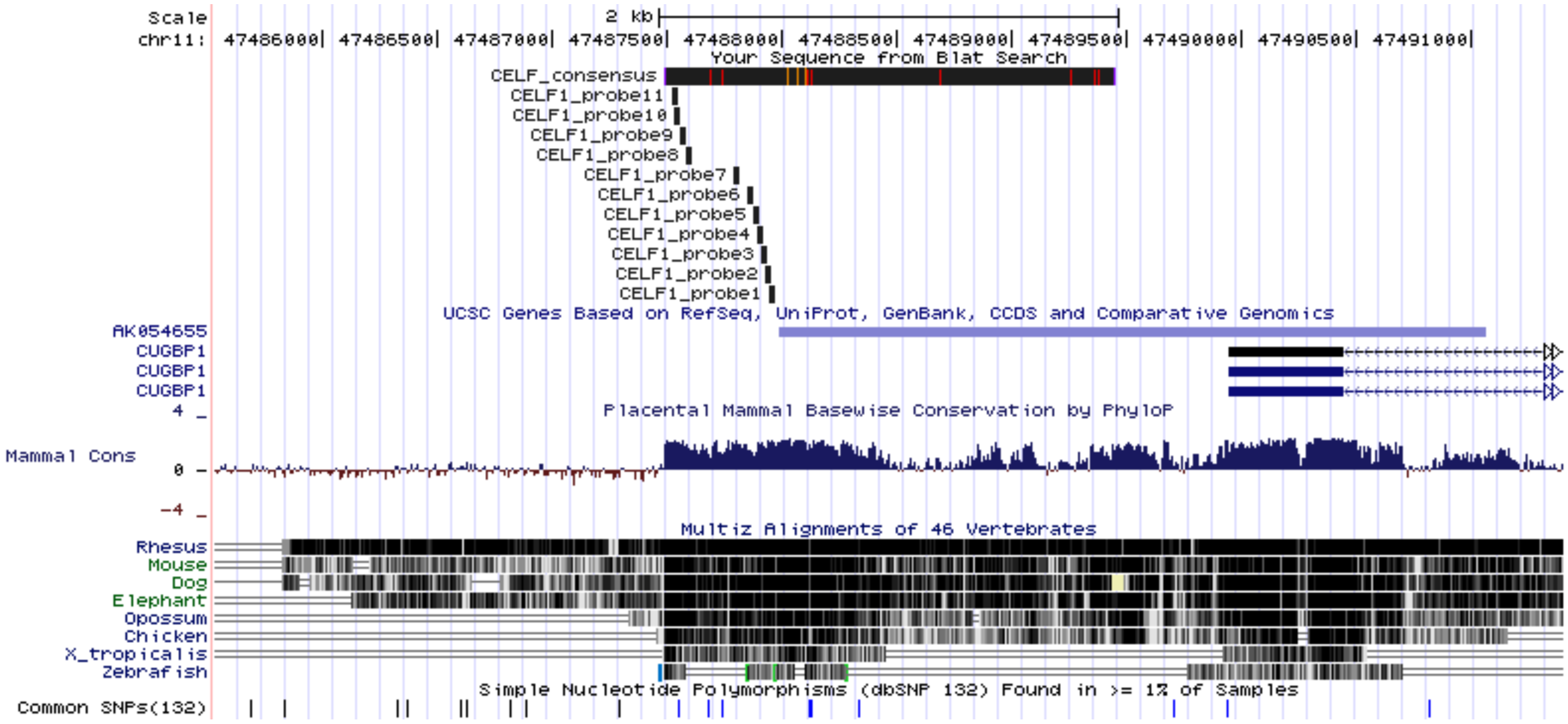

XPO1(208775_at)

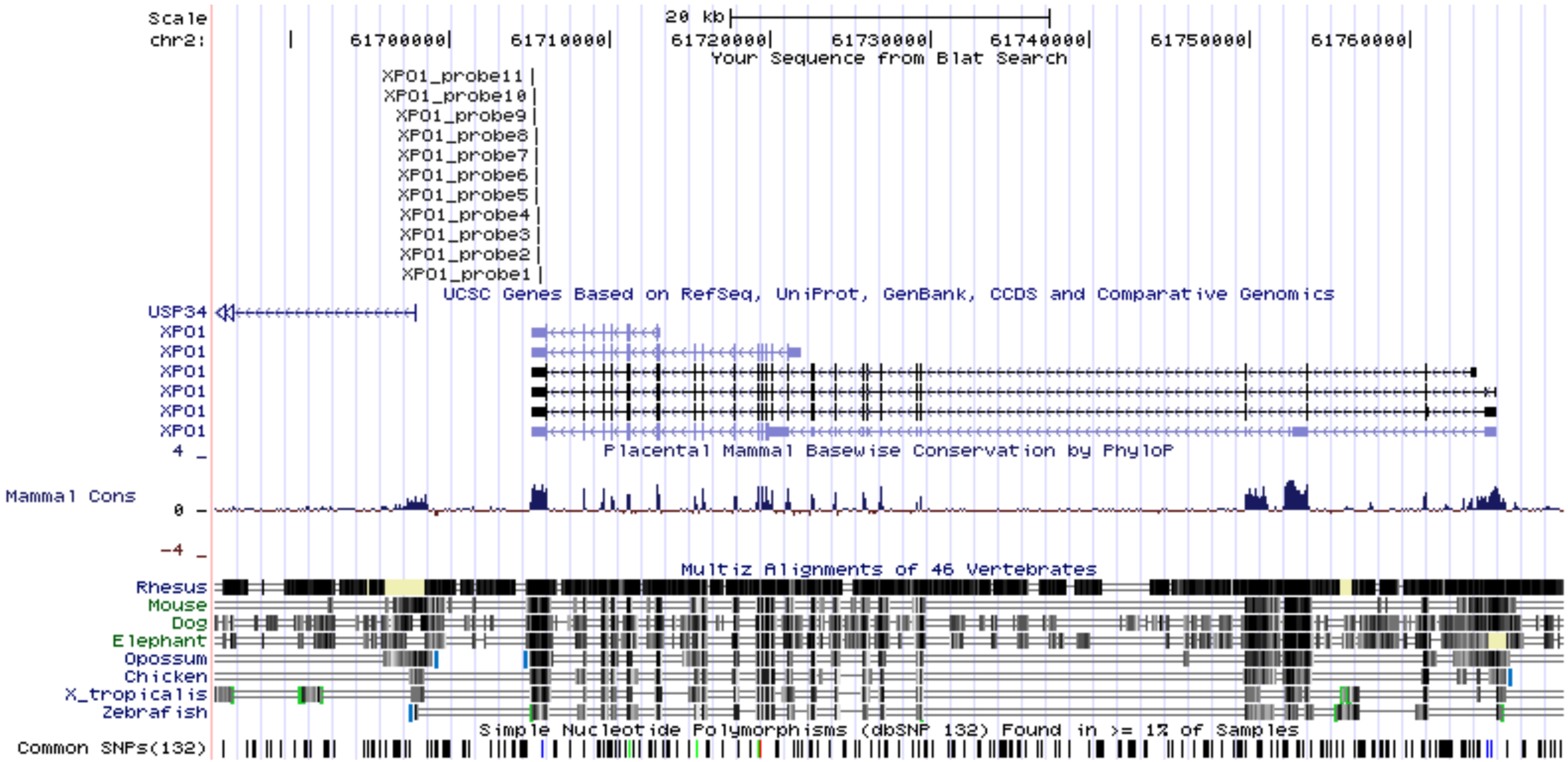

PTBP1(211270_x_at)

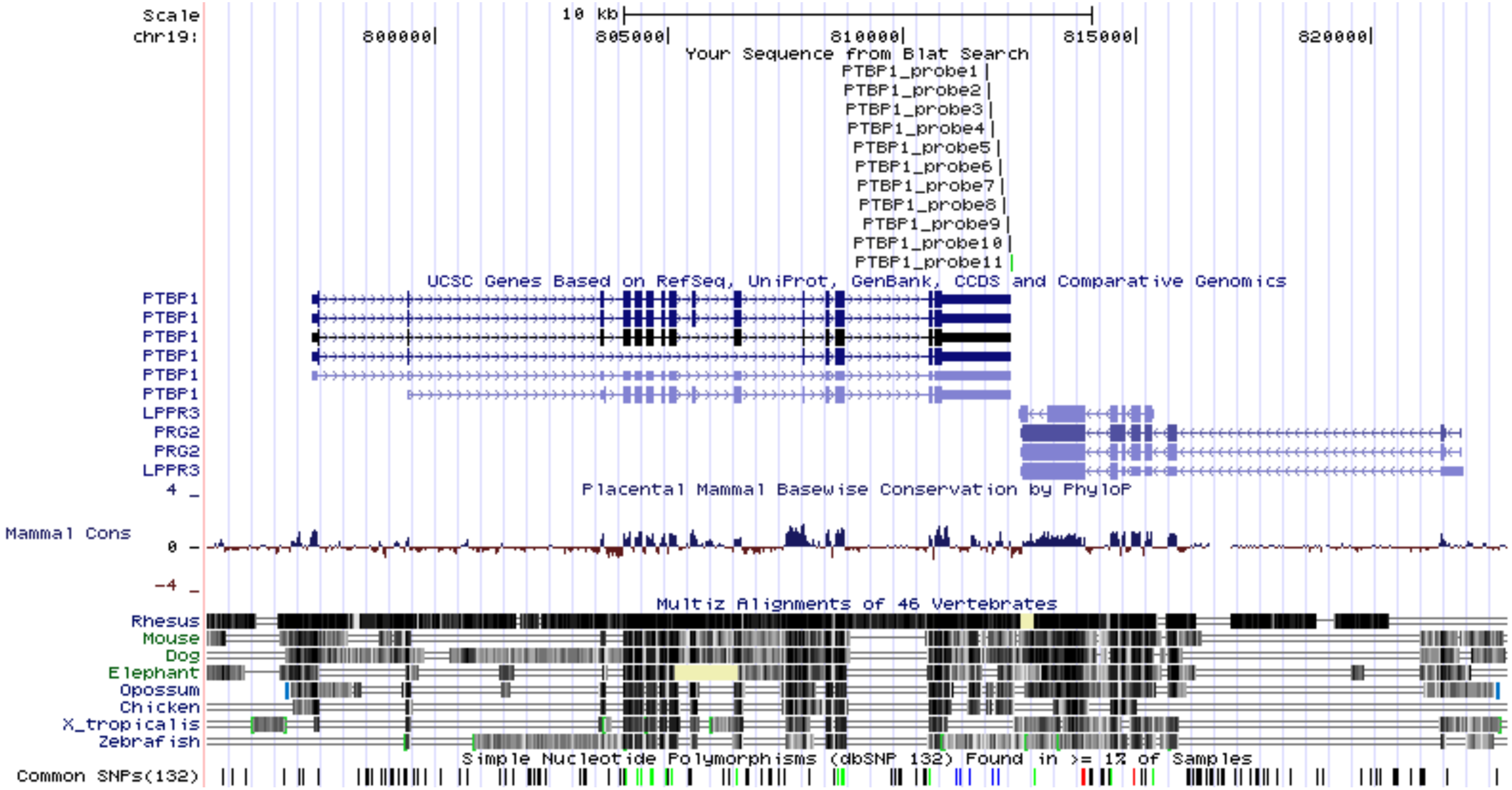

SF3B1(211185_s_at)

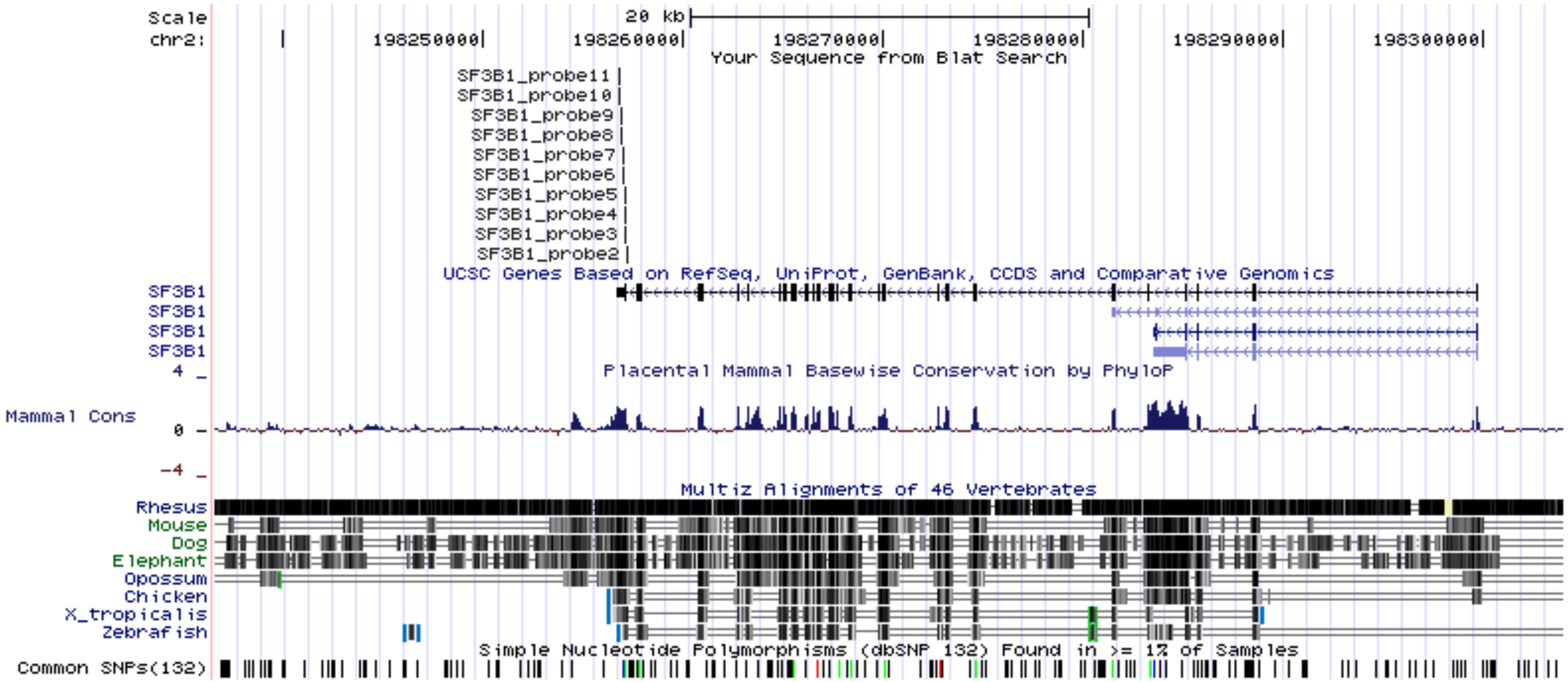

ARPC2(10109_at)

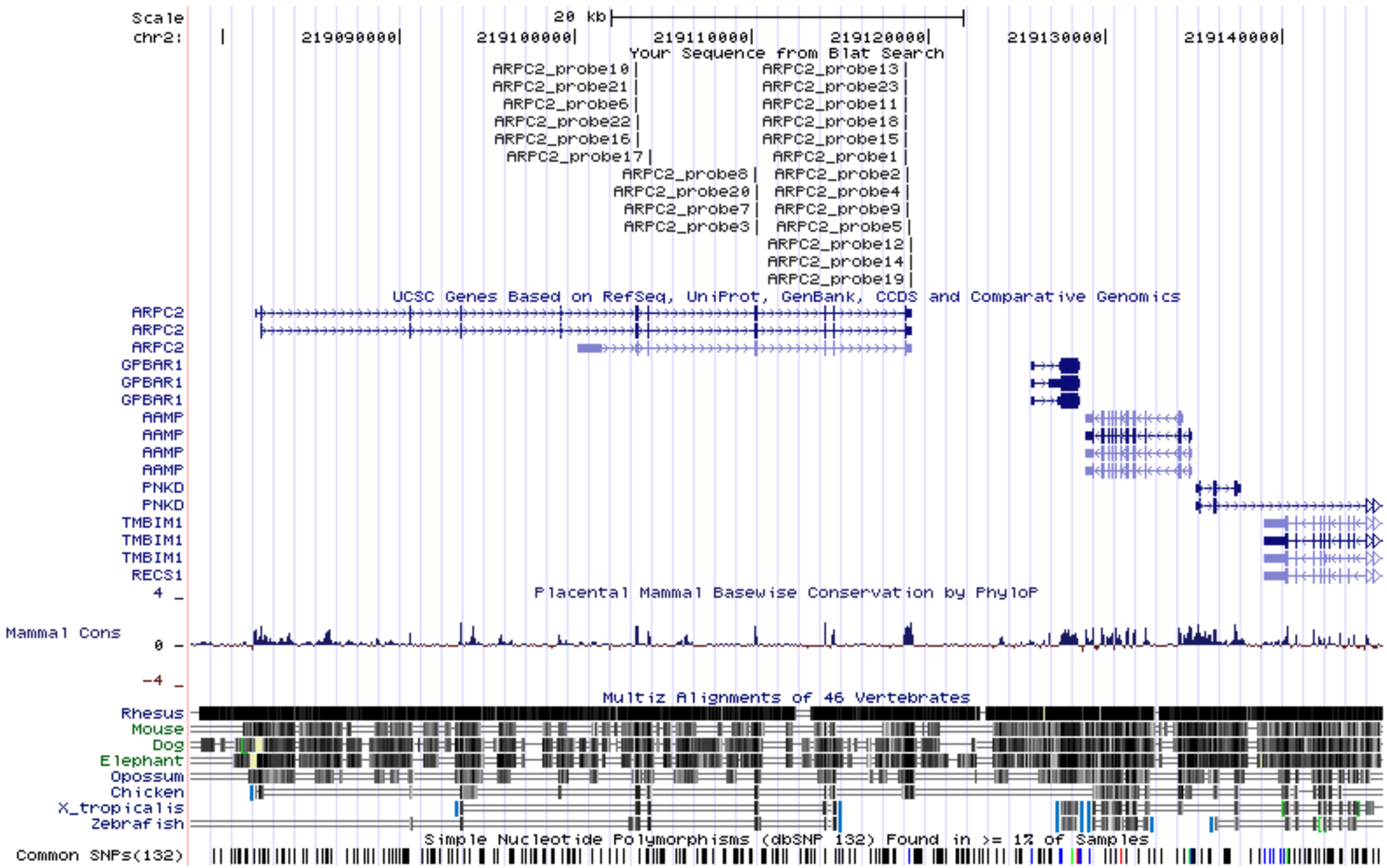

C2orf28(51374_at)

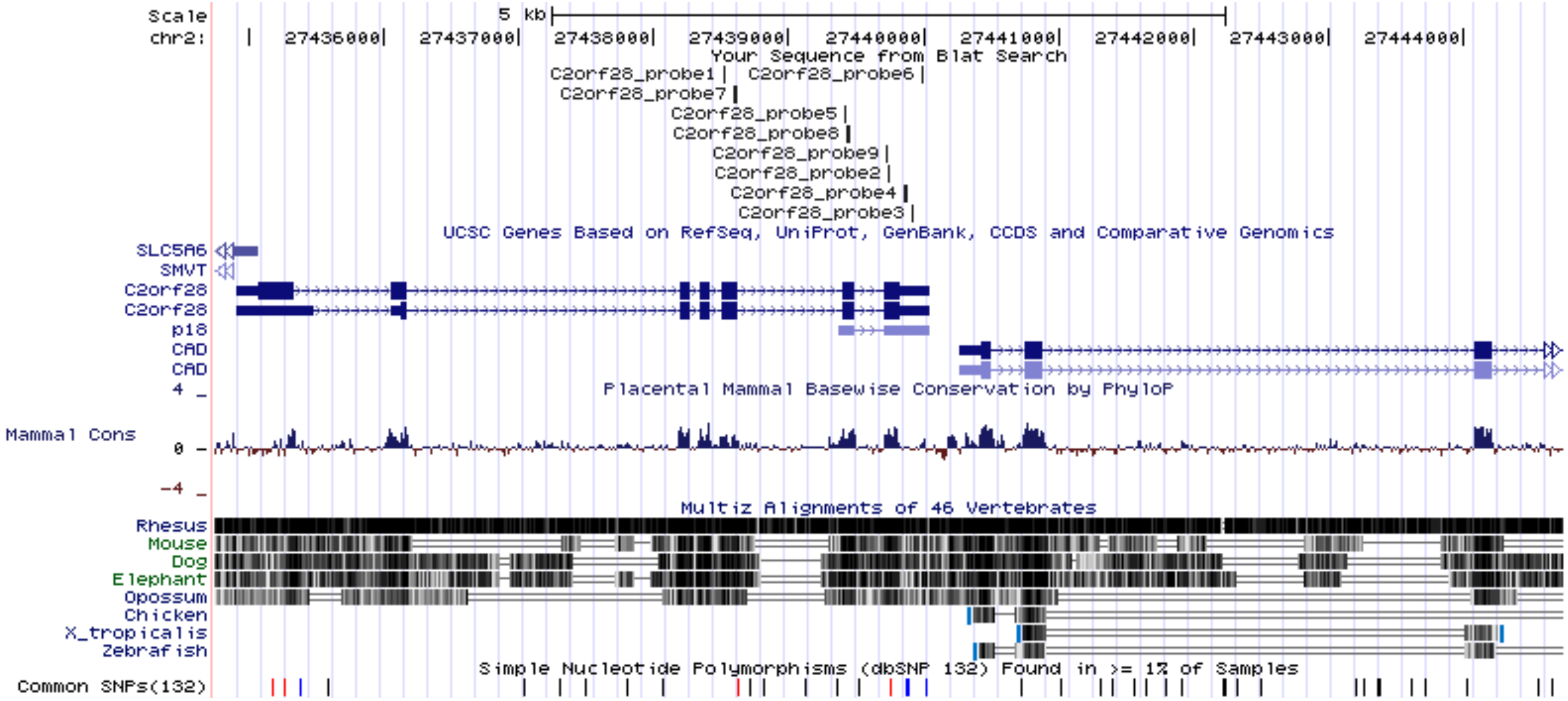

VAMP3(201336_at)

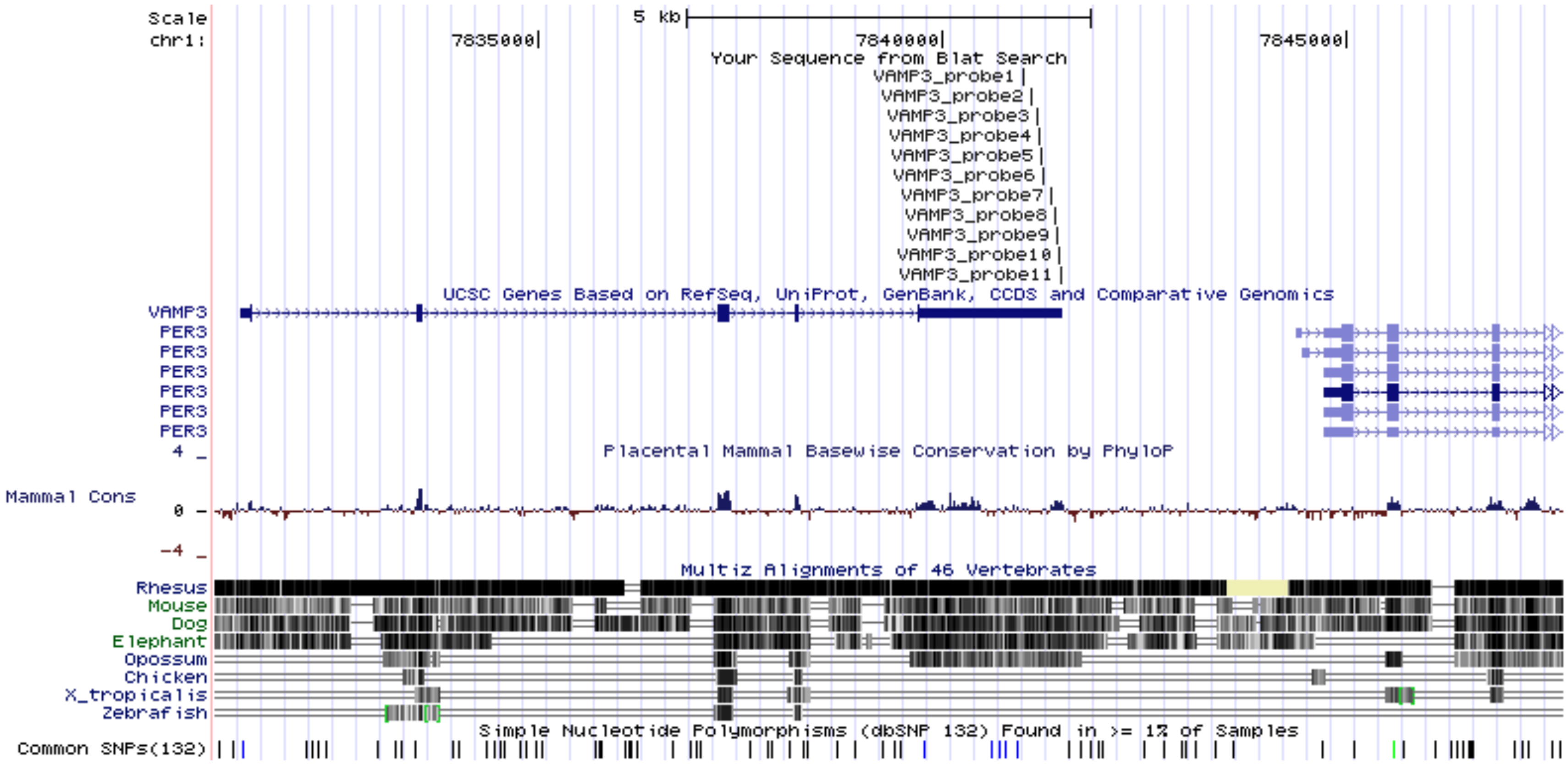

STARD7(200028_s_at)

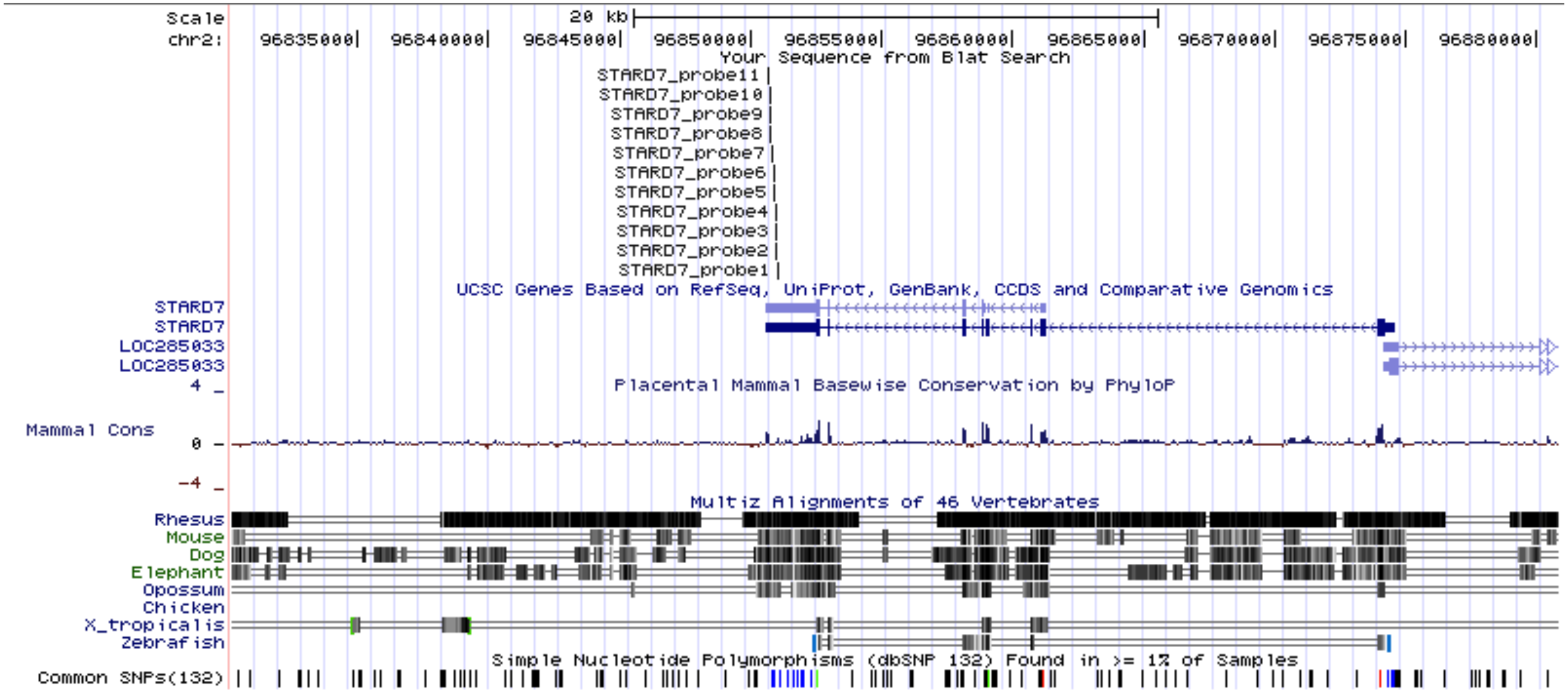

SEC31A(22872_at)

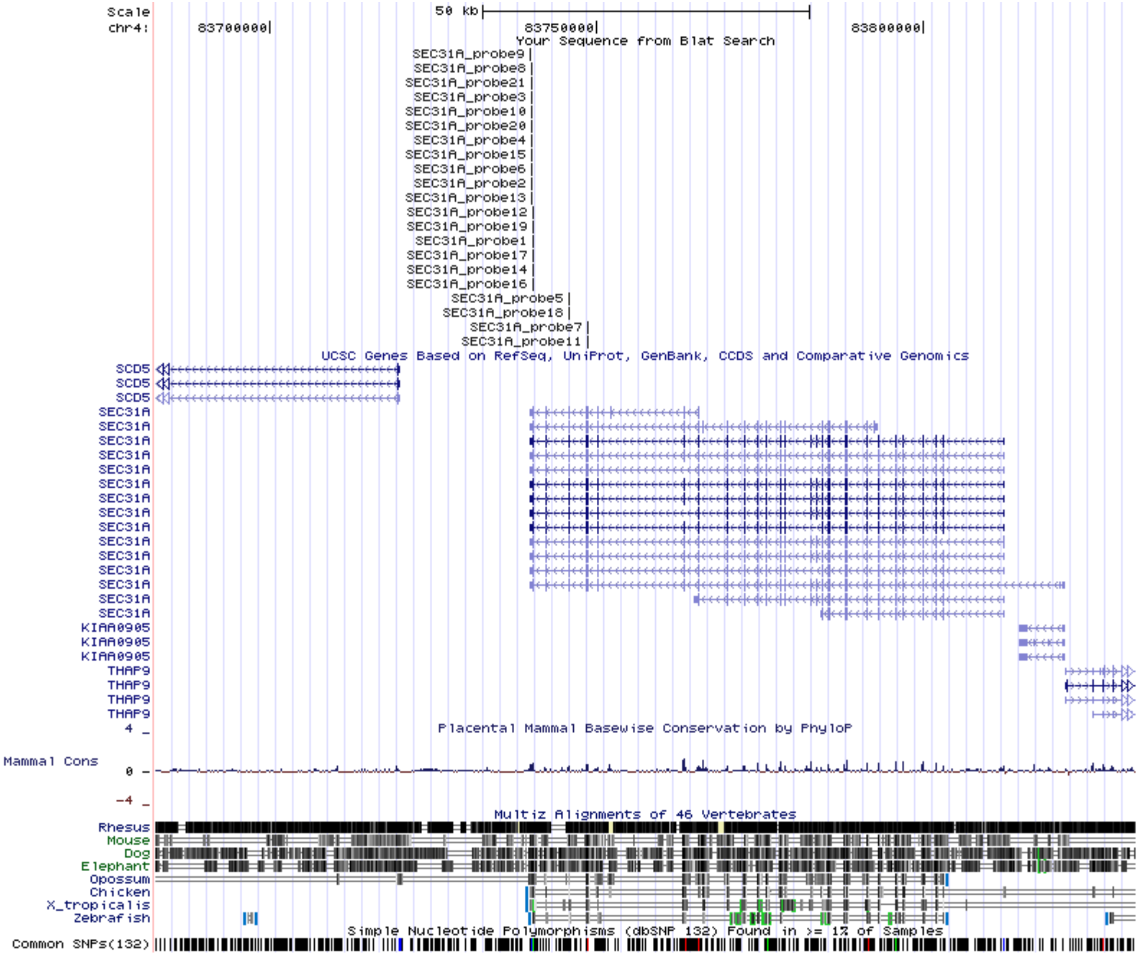

MFN2(9927_at)

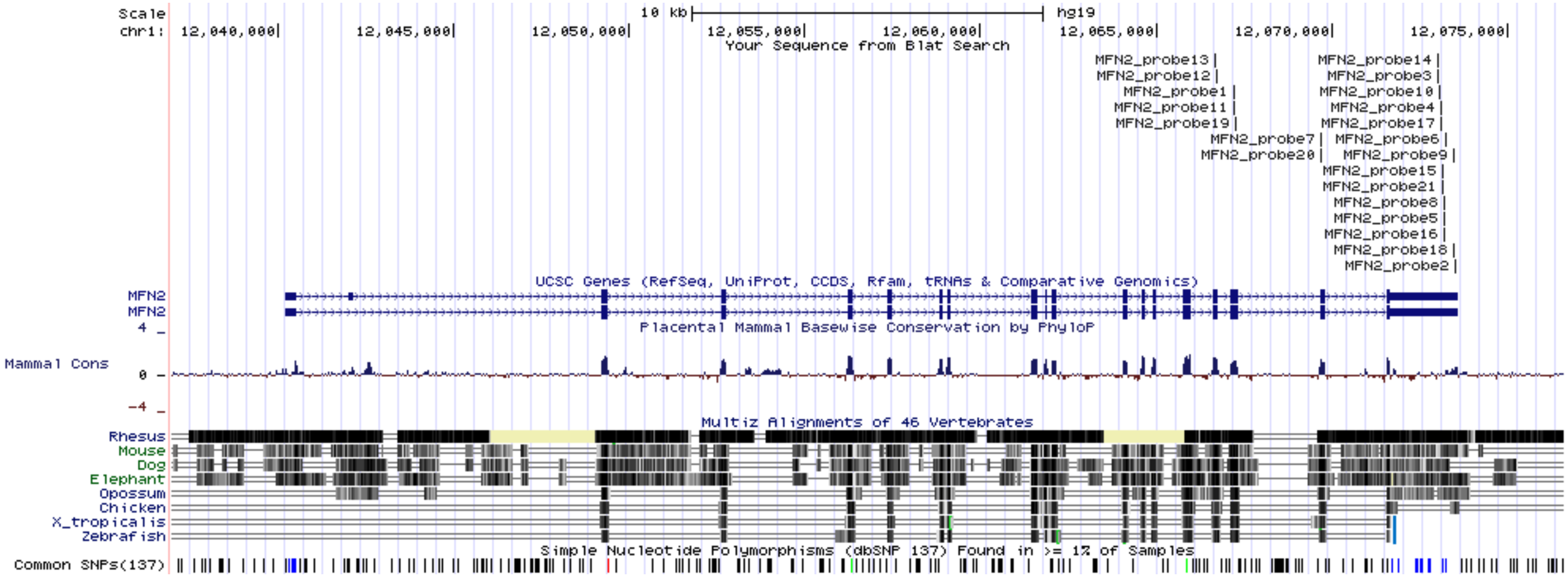

Scale
10 kb
hg19
chr1:
12,040,000
12,045,000
12,050,000
12,055,000
12,060,000
12,065,000
12,070,000
12,075,000
Your Sequence from Blat Search
MFN2_probe13
MFN2_probe12
MFN2_probe1
MFN2_probe11
MFN2_probe19
MFN2_probe7
MFN2_probe20
MFN2_probe14
MFN2_probe3
MFN2_probe10
MFN2_probe4
MFN2_probe17
MFN2_probe6
MFN2_probe9
MFN2_probe15
MFN2_probe21
MFN2_probe8
MFN2_probe5
MFN2_probe16
MFN2_probe18
MFN2_probe2
UCSC Genes (RefSeq, UniProt, CCDS, Rfam, tRNAs & Comparative Genomics)
MFN2
MFN2
Placental Mammal Basewise Conservation by PhyloP
Mammal Cons
4
0
-4
Multiz Alignments of 46 Vertebrates
Rhesus
Mouse
Dog
Elephant
Opossum
Chicken
X_tropicalis
Zebrafish
Simple Nucleotide Polymorphisms (dbSNP 137) Found in >= 1% of Samples
Common SNPs(137)

WIPI2(26100_at)

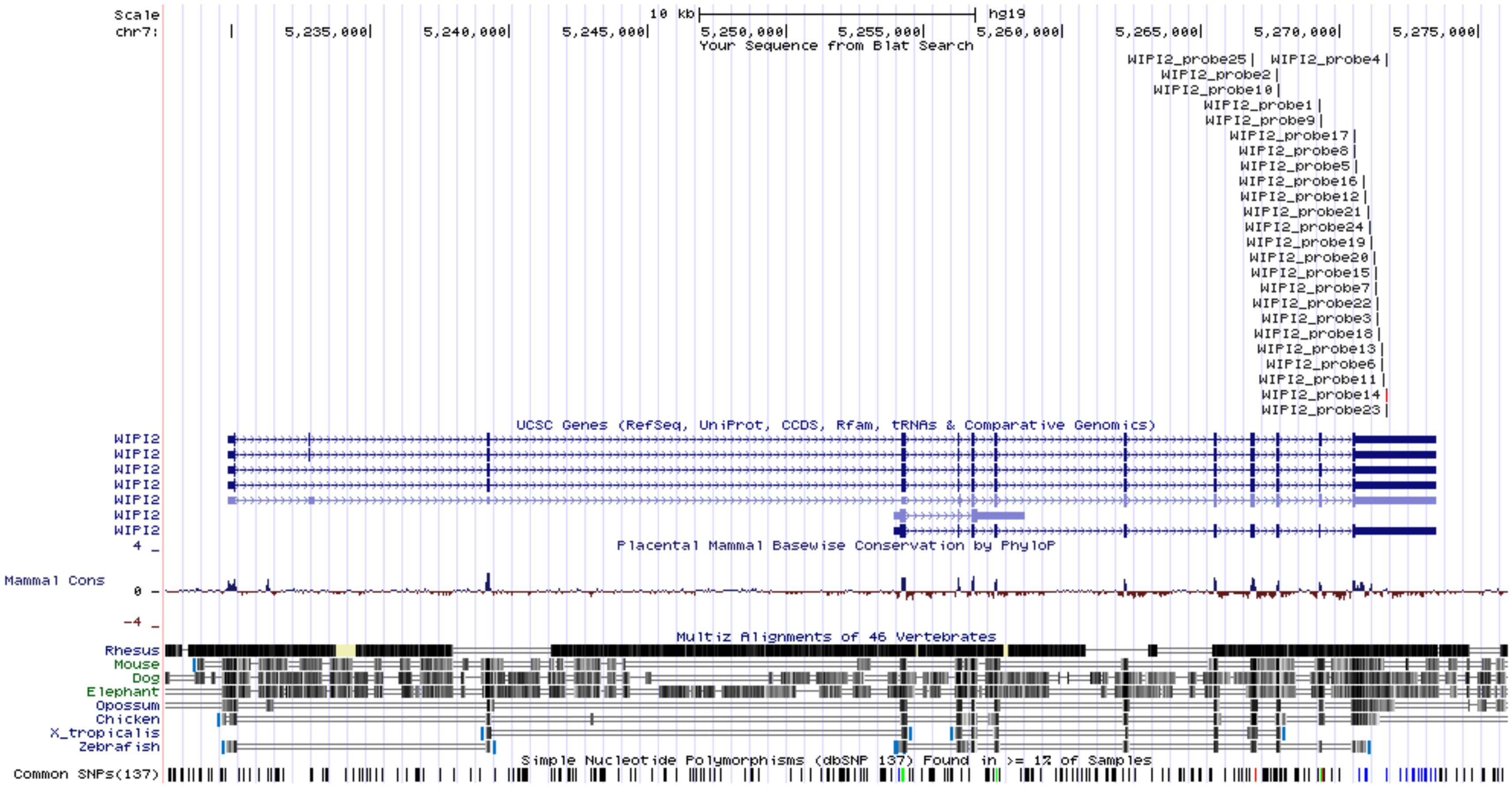

PFDN1(201507_at)

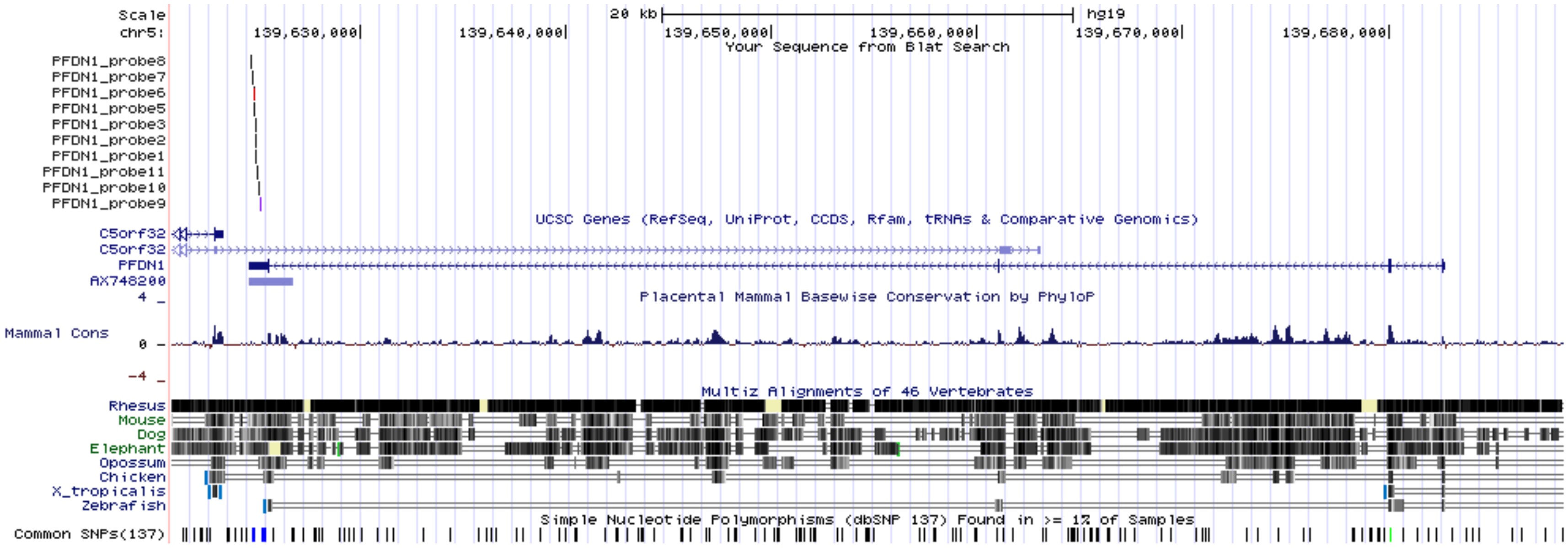

UBE3A(7337_at)

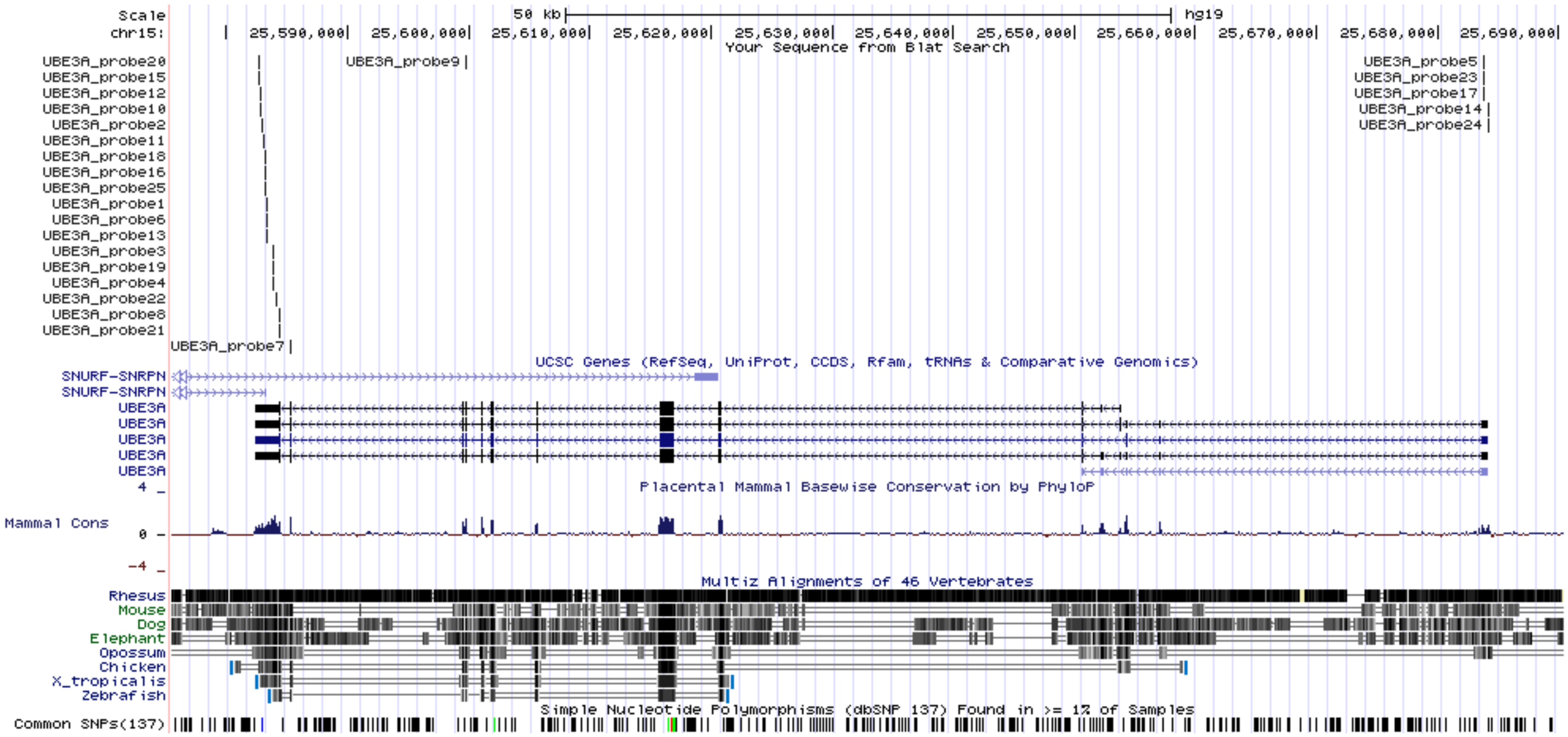

GTF3C2(2976_at)

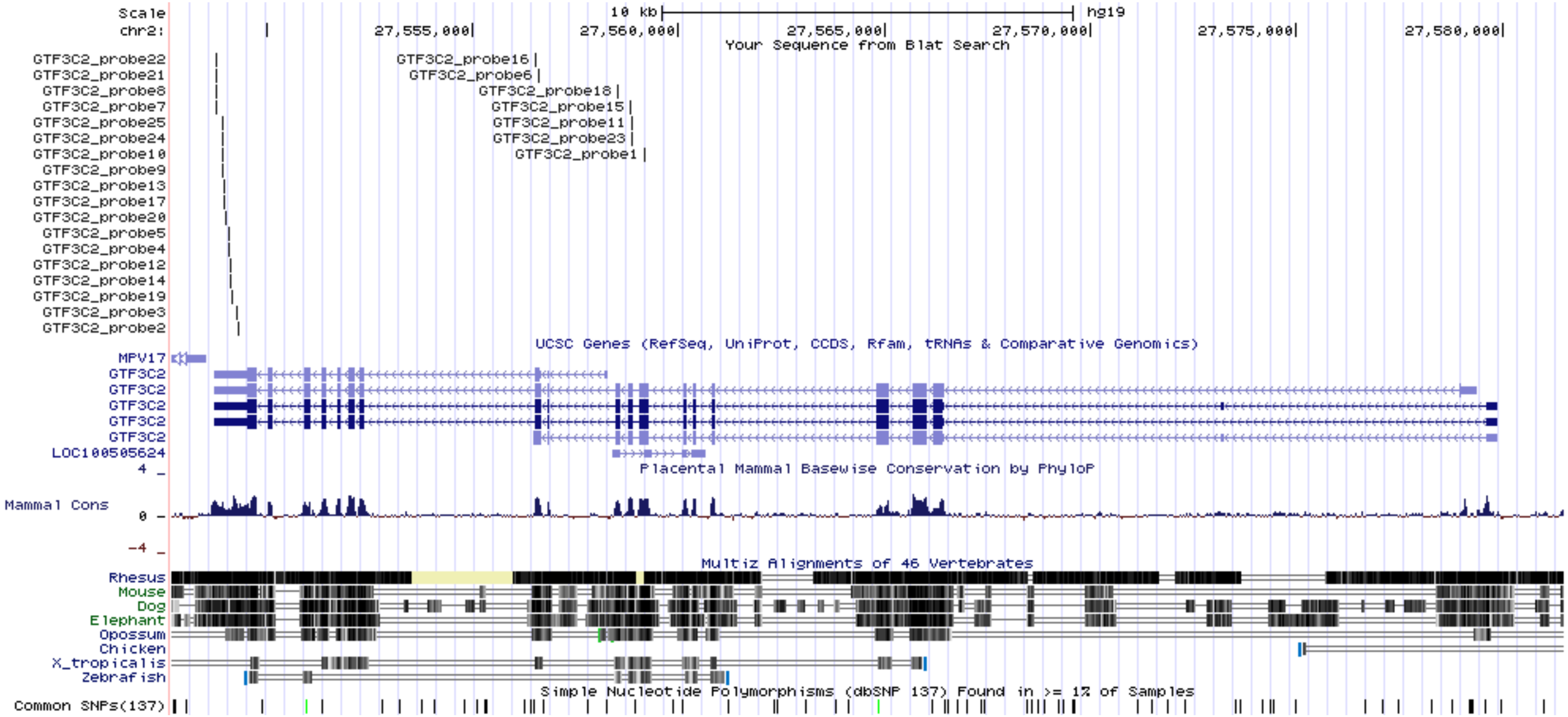

KHDRBS1(10657_at)

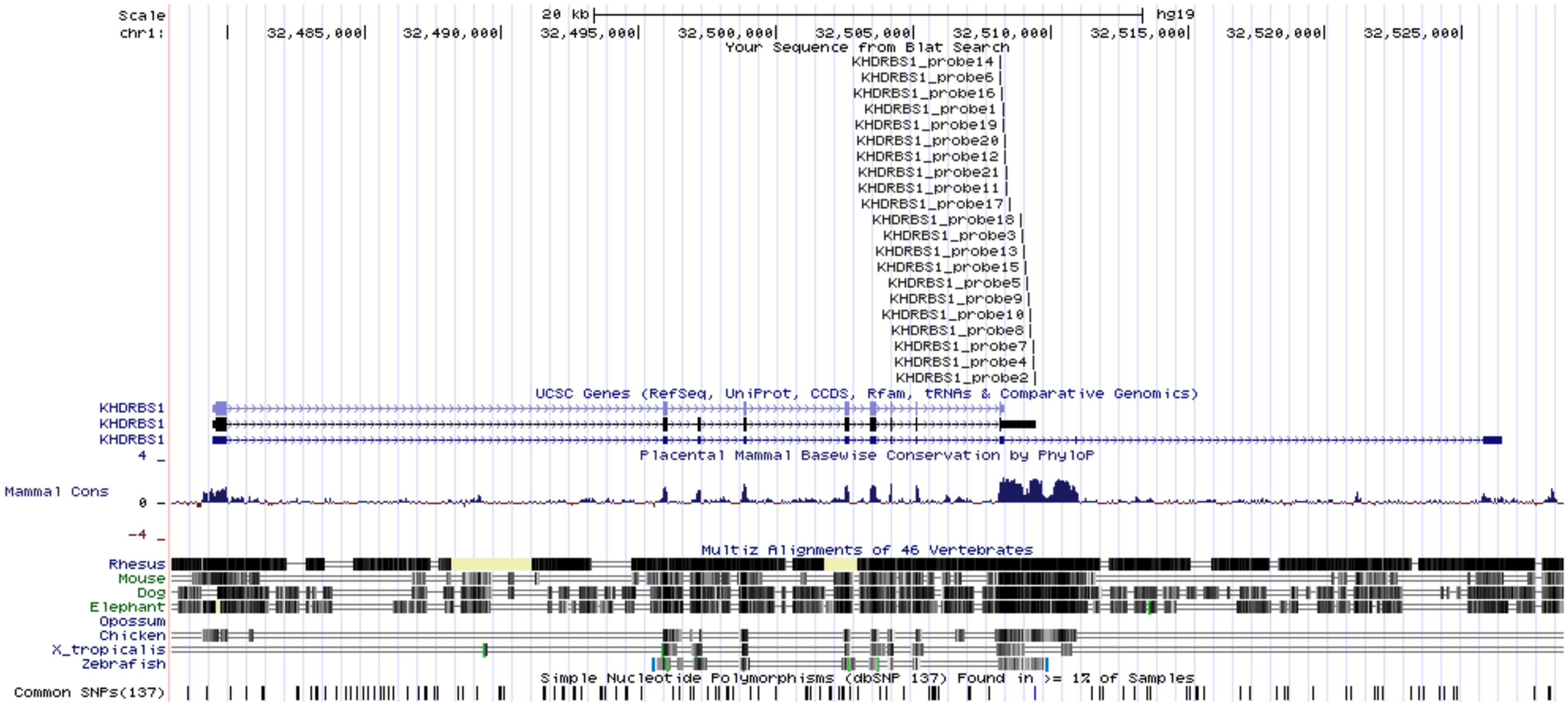

RARS(201330_at)

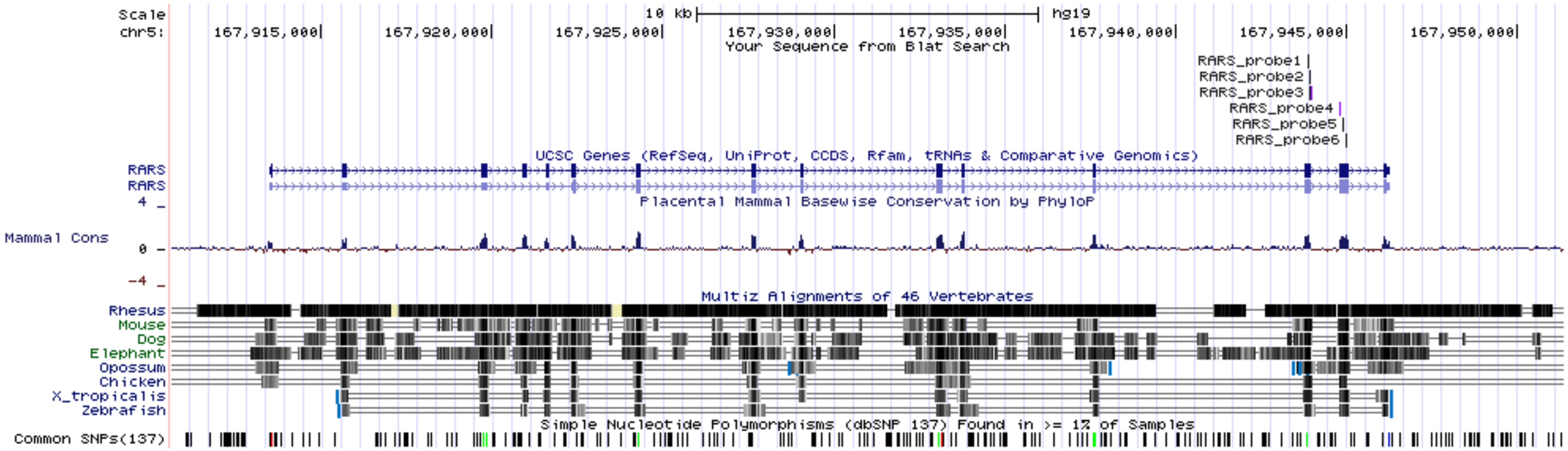

MYL12A(201319_at)

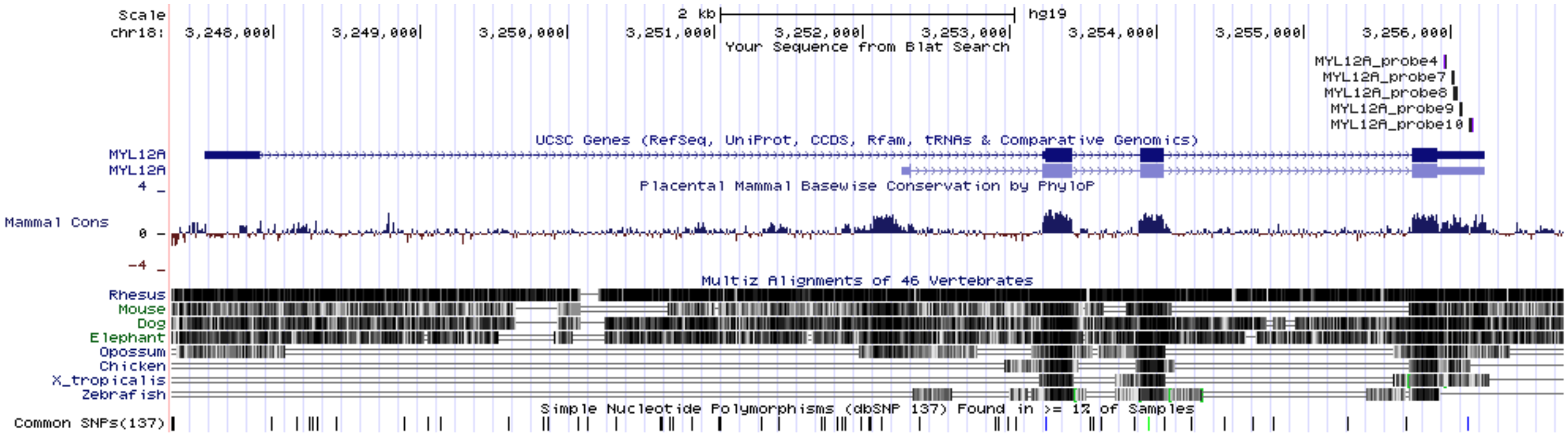

HNRNPD(3184_at)

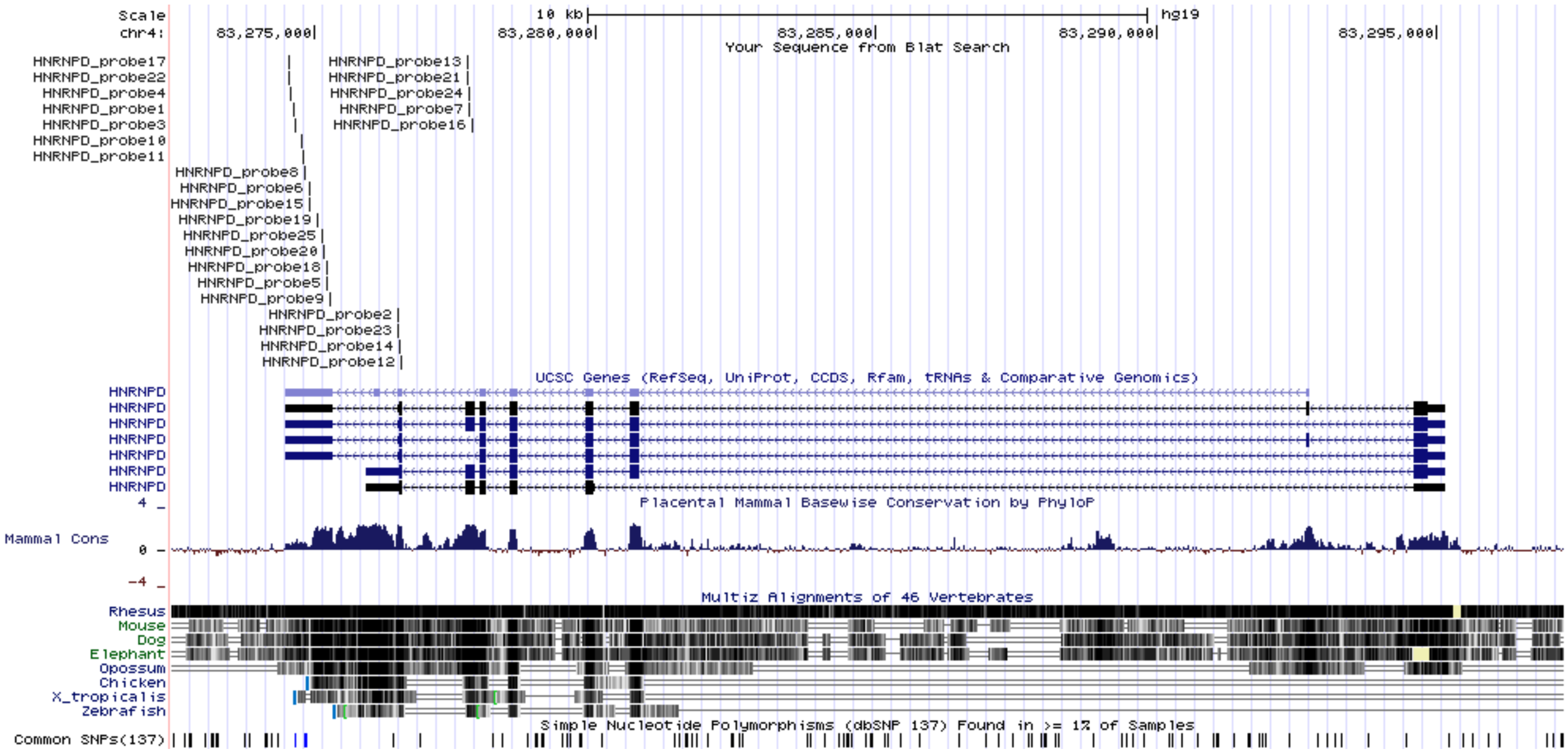

TARDBP(200020_at)

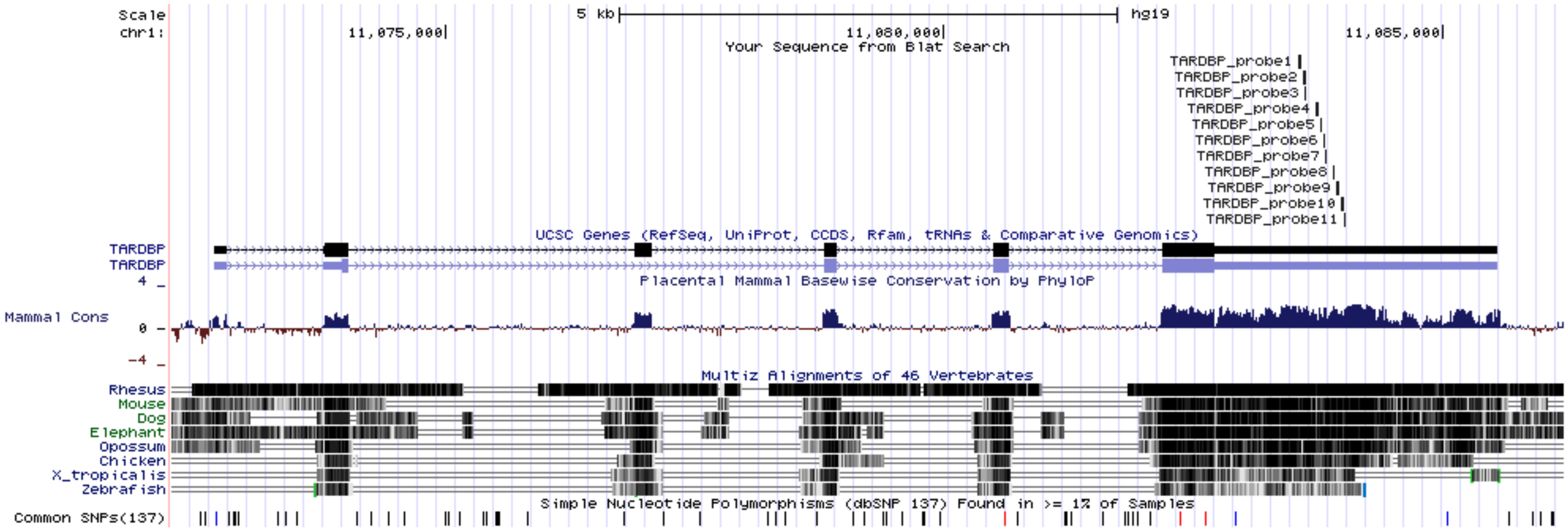

HNRNPR(10236_at)

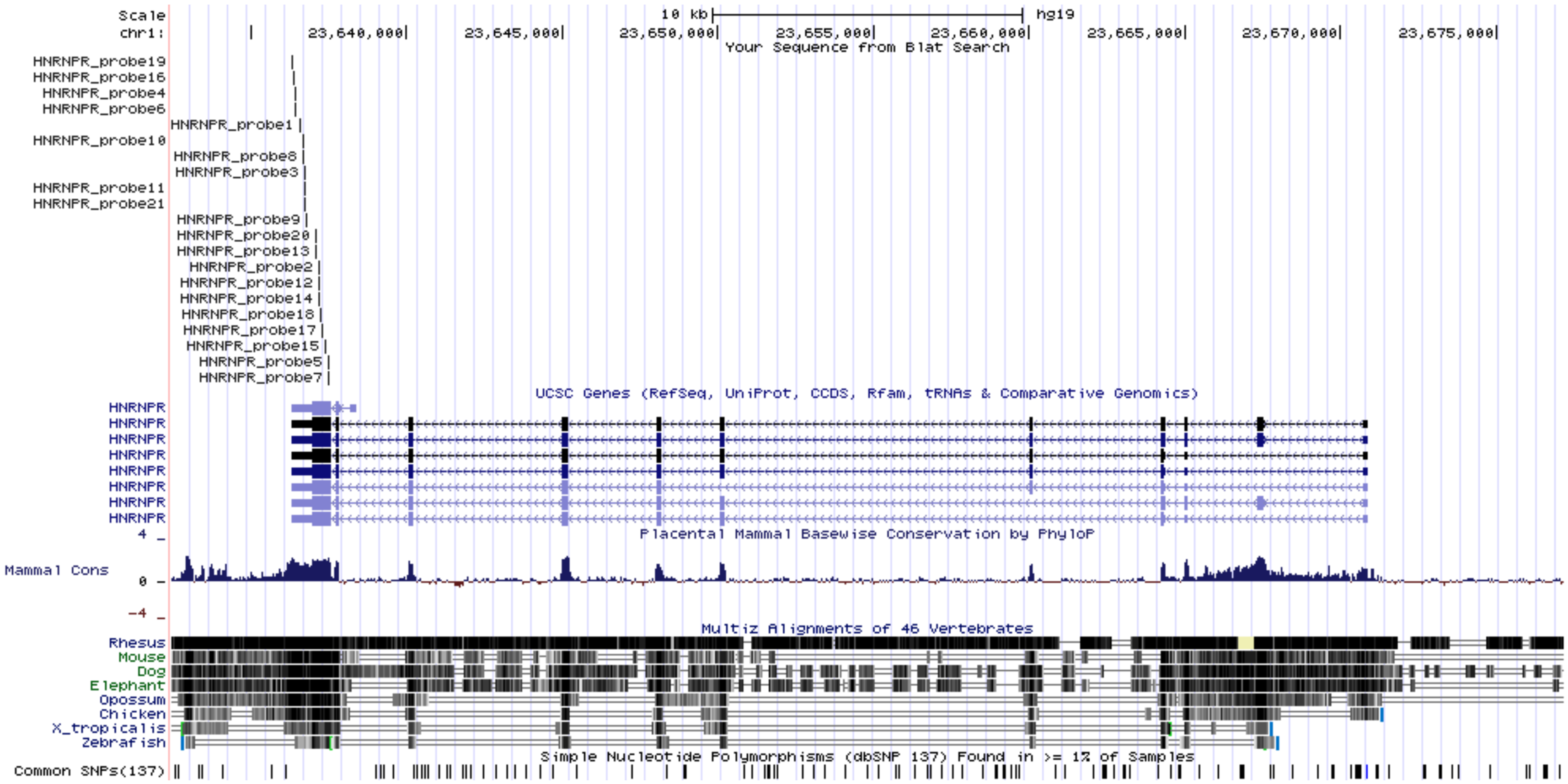

MTCH1(221619_s_at)

- See review of custom CDF probe set for MTCH1 above
- It is recommended to use that probe set

CYFIP1(208923_at)

- See review of custom CDF probe set for CYFIP1 above
- It is recommended to use that probe set
