# Supplementary material for: A robust prognostic signature for hormone-positive node-negative breast cancer
Source: Genome Med. 2013 Oct 11;5(10):92. doi: 10.1186/gm496 (PMC3961800; doi:10.1186/gm496)
Supplement: Additional file 8 — Describes alignment of all reference probe sets to the reference genome. [file gm496-S8.zip › Add 9 and 10 (7)/1217714361945551_add9.pdf]

Appendix 5A

**Reference gene set #1**			
**Gene(probe set)**	**CDF**	**Predictor status**	**Mapping comments**
NACAP1(211445_x_at)	standard	reference gene	CHR8: Probes 2,3,4,5, and 6 map perfectly to 5' UTR of NACAP1 locus on chr8. Probes 7, 8 map perfectly to exon nearest to the 3' UTR of FKSG17 gene on chr8, and probes 9,10, and 11 map perfectly to intronic region of FKSG17 locus on chr8. CHR12: Probes6 and 7 map perfectly to 3' UTR of NACA locus on chr12, and probes 2 an 3 map with little or no mismatch to exon nearest to the 3' end of NACA locus as well. Probes 4 and 7 also map perfectly to intergenic region on chr12. CHR17: Probes 4 and 6 map with little mismatch to 5' UTR of NACA2 locus on chr17. CHR4: Probes 4,6, and 7 map with little or no mismatch to intergenic region on chr4. CHR3 & CHR1: Probe 11 maps perfectly to intronic regions on chr3 and chr1. Probe1: Probe 1 also maps with little or no mismatch to intergenic regions on chr10, chr15, and chrX, and to intronic regions on chr16, chr2, chr14, and chr5.
PTMA(216515_x_at)	standard	reference gene	CHR2: Probes 3,4,10 map with little or no mismatch to 3' UTR of PTMA locus on chr2. CHR20: all probes map with little or no mismatch to intragenic region. CHR3: Probe 1 maps perfectly to intronic region. CHR12: Probes 2,3,10 map with little or no mismatch to intragenic region. CHR5: Probes 3,4,5,10 map with little or no mismatch to intronic region. CHR7: Probes 3, 6, 10 map with little or no mismatch to intragenic region. CHR13: Probes 3,4,5,9, 10 map with little or no mismatch to intragenic region. CHR17: Probes 9 and 10 map with little mismatch to intragenic region. CHR8: Probes 9 and to map with little or no mismatch to intragenic region. CHR6: Probe 10 maps with little mismatch to intragenic region. CHR14: Probes 3, 4, 5 map with little mismatch to intronic region. CHR6_qbl_hap6: Probe 3 maps with little mismatch to intronic region. CHRX: Probe 4 maps with some mismatch to intronic region. CHR6_ssto_hap7: Probes 4 and 5 map with little mismatch

			to intronic region.
RPL7(217092_x_at)	standard	reference gene	CHR8: Probe 2 maps perfectly to 4th exon from 3' end, Probe 4 maps perfectly to 3rd exon from 3' end, probe 8 maps with little mismatch to 2nd exon from 3' end, and probe 10 maps with little mismatch to exon nearest 3' end of the RPL7 locus on chr8. CHR22: All probes map perfectly to intronic region on chr22. CHR1: Probes 2,9,10 map with little or no mismatch to intragenic region. CHR2: Probes 2,4,10 map with little mismatch to intragenic region. CHR3: Probes 2,8,10 map with little or no mismatch to intronic region. CHR5: Probes 2,4,8,10 map with little or no mismatch to intronic region. CHR6: Probes 2,4,9,10 map with little or no mismatch to intronic region. CHR7: Probes 8 and 10 map with little or no mismatch to intragenic region. CHR9: Probe 10 maps perfectly to intronic region. CHR10: Probes 4,7 map with little mismatch to intragenic region. CHR12: Probes 2,4,7,8 map with little mismatch to intragenic region. CHR13: Probes 2,3,4,9,10 map with little mismatch to intragenic region. CHR16: Probes 2,8,10 map with little mismatch to intragenic region. CHR17: Probes 2,4,7,8,10 map with little or no mismatch to intragenic region. CHR20: Probes 7,10 map with little mismatch to intragenic region. CHRX: Probes 7,10 map with little mismatch to intragenic region. CHR6_mcf_hap[1-5]: Probes 2,4,10 map with little or no mismatch to intragenic regions.
MUL12B(103910_at)	custom	reference gene	Probes 1, 2, and 4 map with little or no mismatch to 3' UTR of MYL12B locus on chr18. Probe 3 failed to map. Probes 1,2, and 4 also map with little mismatch to intergenic region on chr4.
SFRS3(208672_s_at)	standard	reference gene	All probes map perfectly to 3' UTR of SFRS3 locus on chr6. Probes 3,4,5,6,7,8, and 9 also map with some mismatch to intergenic region on chr11. Probe 5 also maps with some mismatch to intergenic region on chr8, and probe 8 maps with some mismatch to two intergenic regions on chr2, as well as to an intergenic region on chr7.
CLTA(200960_x_at)	standard	reference	All probes map perfectly or with little mismatch to 3' UTR and exons 1-3

		gene	(from 3' end) of CLTA locus on chr9. Probes 9 and 11 also map with little or no mismatch to intergenic region on chr12, and probe 2 also maps with little mismatch to intronic region on chr15.
TRA2B(200893_at)	standard	reference gene	All probes map perfectly to 3' UTR of TRA2B (aka SFRS10) locus on chr3. Probe 1 also maps with some mismatch to intergenic region on chr4.
RPS2(217466_x_at)	standard	reference gene	All probes map perfectly to 3rd exon from 3' end of RPS2 locus on chr16, but 5 probes also map to coding region of SNORA64 locus and OK/KNS-c1.6 locus on the same chromosome. Probes 8, 9, 10, and 11 also map with little mismatch to intergenic region on chrX, and to intronic regions on chr1, chr4, chr6, and chr17. Probes 9,10, and 11 also map with little or no mismatch to an intergenic region on chr20. Probes 8,10, and 11 also map with little mismatch to another intronic region on chr17. Probes 6,7,8,9,10 and 11 also map with little or no mismatch to noncoding AB055772 locus on chr17 and to intronic region on chr12. Probes 8, 9, and 10 map with little or no mismatch to intronic regions on chr1 and chr19 and to intergenic region on chr18. Probes 10 and 11 map to intergenic region on chr8 and chr11. Probes 9 and 10 map with some mismatch to intronic region on chr5.
MTCH1(23787_at)	custom	reference gene	All probes map perfectly to 3' UTR of MTCH1 locus on chr6. Probes 1 maps with little mismatch to intergenic region on chr11, probe 4 maps with little mismatch to intergenic region on chrX, and probes 2,3, and 9 map with little mismatch to intergenic region on chr6.
HDLBP(221767_x_at)	standard	reference gene	Probes 4 and 5 map perfectly or with little mismatch to exon second nearest 3' end, and probes 6,7,8,9,10, and 11 map perfectly to 3' UTR of HDLBP locus on chr2. Probes 1,2,3 failed to map. Probe 10 also maps with little mismatch to intergenic region on chr1, probe 4 maps with some mismatch to intronic region on chr6, and probe 8 maps with some mismatch to intronic region on chr 5.
CYFIP1(23191_at)	custom	reference gene	All probes map perfectly to 5' UTR of CYFIP1 locus on chr15.

SUMO1(211069_s_at)	standard	reference gene	All probes map perfectly to 3' UTR of SUMO1 locus on chr2. All probes but probe 8 also map with little mismatch to noncoding SUMO1P3 locus on chr1. Probes 1,3,9, and 11 map with some mismatch to noncoding SUMO1P1 locus on chr20. Probes 3,4,7,8,9,10,11 map with some mismatch to intergenic region on chr5, and probes 1,3,4,7,8,9,11 map with little mismatch to intergenic region on chr19.
DHX15(201385_at)	standard	reference gene	All probes but probe 10 map perfectly to 3' UTR of DHX15 locus on chr4. Probe 10 maps with little mismatch to intergenic region on chr3.
HNRNPC(200014_s_at)	standard	reference gene	All probes map perfectly to 3' UTR of HNRNPC, HNRPC, and HNRPCL1 locus on chr14. All probes also map with little or no mismatch to 3' UTR of coding CR603438 locus on chr2.Probes 1,3,4,8,9, and 10 map with little mismatch to intergenic region on chr15 as well, and probes 2,5, and 6 also map with little mismatch to intergenic region on chr16. Probes 1,2,3,5,8, and 11 also map with little mismatch to intergenic region on chr11, and probe 10 maps with little mismatch to intergenic region on chr13.
UBE2D3(200667_at)	standard	reference gene	All probes map perfectly to 3' UTR of UBE2D3 locus on chr4. Probes 1,2,3,5,6,8,9, and 10 map with little or no mismatch to intergenic region on chr20. Probe 9 also maps with some mismatch to intergenic region on chr3, chr5, and chrX and to intronic region on chr14.
DAZAP2(9802_at)	custom	reference gene	All probes map perfectly to 3' UTR or exon nearest 3' UTR of DAZAP2 locus on chr12. Probe 15 maps with some mismatch to intergenic region on chr15, probes 3,4, and 8 map with some mismatch to intergenic region on chr2, probe 8 maps with some mismatch to intergenic region on chr1.
SNRNP200(200058_s_at)	standard	reference gene	All probes map perfectly to either 3' UTR or exons 1-3 nearest 3' end of SNRNP200 locus on chr2. However, there is an overlapping nearcoding gene on chr2 as well (KIAA0788), so all probes map to this locus as well. Probes 1,2,4,5, and 7 also map with some mismatch to intronic region on chr17.
YTHDC1(91746_at)	custom	reference gene	All probes map perfectly to 3' UTR of YTHDC1 locus on chr4. All probes also map perfectly to 3' UTR of YTHDC1 locus on 4_ctg9_hap1 haplotype.

COPB1(1315_at)	custom	reference gene	All probes map perfectly to 3' UTR or exon 2nd nearest to 3' end of COPB1 locus on chr11. Probe 17 also maps with little mistmatch to intronic region on chr15. Probe 5 also maps with little mismatch to intergenic region on chr2.
NDUFB8(4714_at)	custom	reference gene	All probes map perfectly to exons 1-4 nearest to 5' end of NDUFB8 locus on chr10. Probes 1, 8, and 14 also map with little mismatch to intronic regions on chr17,and probes 7 and 13 map with little mismatch to intergenic region on chr14.
SET(40189_at)	standard	reference gene	All probes map perfectly to 3' UTR of SET locus on chr9. Probes 1,2,3, 10,11,12,13, and 16 also map with some mismatch to intergenic region on chrX; probes 4,10, 11,12, 14, 15, and 16 map with some mismatch to intergenic region on chr5; probes 2 and 8 map with some mismatch to intergenic region on chr14; probes 1,2,3,4,6,8,11,12,13,14, and 16 map wiht some mismatch to intergenic region on chr12; probes 8,9, 10, 11, and 16 map with some mismatch to intergenic region on chr16. Probes 6, 8, 12,13, and 14 map with some mismatch to intronic region on chr6, and probe 12 maps with some mismatch to intronic region on chr13.
CELF1(221743_at)	standard	reference gene	No probes map to CELF1 locus. All probes map perfectly to intergenic region on chr11. Probes 2,4,5,6,7 and 11 map with some mismatch to intergenic region on chr15; probes 10 and 11 map with little mismatch to intronic region on chr5; probe 10 maps with some mismatch to intronic region on chr2; probe 8 maps with some mismatch to intronic region on chr10.
XPO1(208775_at)	standard	reference gene	All probes map perfectly to 3' UTR of XPO1 locus on chr2.
PTBP1(211270_x_at)	standard	reference gene	All probes map perfectly to 3' UTR of PTBP1 locus on chr19. Probe 10 also maps with little mismatch to intergenic region on chr19.
SF3B1(211185_s_at)	standard	reference gene	All probes but probe 1 map perfectly to 3' UTR or exon nearest 3' end of SF3B1 locus on chr2. Probe 1 failed to map.
ARPC2(10109_at)	custom	reference	All probes map perfectly to either 3' UTR or exons 1,4,5, or 6 from 3' end of

		gene	ARPC2 locus on chr2. Probe 10 also maps with some mismatch to intergenic region on chr10; Probes 12 and 15 map with some mismatch to an intergenic region on chr13, and probes 15 and 18 also map to a different intergenic region on chr13. Probe 3 also maps with some mismatch to intronic region on chr3.
C2orf28(51374_at)	custom	reference gene	All probes map perfectly to 3' UTR of exons 1-3 from 3' end of C2orf28 locus on chr2.
VAMP3(201336_at)	standard	reference gene	All probes map perfectly to 5' UTR of VAMP3 locus on chr1.
STARD7(200028_s_at)	standard	reference gene	All probes map perfectly to 3' UTR of STARD7 locus on chr2. Probe 4 also maps with some mismatch to intronic regions on chr4 and chr1.
SEC31A(22872_at)	custom	reference gene	All probes map perfectly either to 3' UTR or exons 1,3, or 4 from 3' end of SEC31A locus on chr4. Probe 2 also maps with some mismatch to intergenic region on chr1.
**Reference gene set #2**			
**Gene(probe set)**	**CDF**	**Predictor status**	**Mapping comments**
MFN2(9927_at)	custom	reference gene	All probes map perfectly either to 3' UTR or exons 1, 2, 3, or 4 from 3' end of MFN2 locus on chr1. Three probes also map with some mismatches to other locations.
WIPI2(26100_at)	custom	reference gene	All probes map perfectly (or near perfectly) either to 3' UTR or exons 1, 2, 3, or 4 from 3' end of WIPI2 locus on chr7. One probe also maps with some mismatches to other locations.
PFDN1(201507_at)	standard	reference gene	All probes but probe 4 map perfectly (or near perfectly) to 3’ UTR of PFDN1 locus on chr5. Three probes also map to intron of PPARG on chr3. Probe 8 had multiple additional alignments. Probe 4 failed to map.
UBE3A(7337_at)	custom	reference gene	All probes map perfectly to 3’ UTR or 1st or last exon from 3’ end of UBE3A locus on chr15. Five probes also map to intergenic region of chr21.
GTF3C2(2976_at)	custom	reference	All probes map perfectly to 3’ UTR or most exons of GTF3C2 locus on chr2.

		gene	
KHDRBS1(10657_at)	custom	reference gene	All probes map perfectly to 3' UTR or exon 1 from 3' end at KHDRBS1 locus on chr1. Three probes have additional alignments with some mismatches.
RARS(201330_at)	standard	reference gene	Six probes match perfectly (or near perfectly) to exons 2 and 3 from 3' end of RARS locus on chr5. Five probes failed to map.
MYL12A(201319_at)	standard	reference gene	Five probes match perfectly (or near perfectly) to 3' UTR of MYL12A locus on chr18. Probe7 also maps to intergenic region of chr8.
HNRNPD(3184_at)	custom	reference gene	All probes match perfectly (or near perfectly) to 3' UTR or exons 2 and 3 from 3' end of HNRNPD on chr4. Six probes map to intergenic region on chrX or elsewhere.
TARDBP(200020_at)	standard	reference gene	All probes match perfectly to 3' UTR of TARDBP locus on chr1. Multiple probes also align (with mismatches) to intergenic regions on chr6, chr8, chr13, chr20 and 3'UTR of LOC643387 on chr2.
HNRNPR(10236_at)	custom	reference gene	All probes match perfectly (or near perfectly) to 3' UTR or exon 1 from 3' end of HNRNPR on chr1. Six probes also align to intron of MLLT10 on chr10.
TRA2B(200893_at)	standard	reference gene	See above.
RPS2(217466_x_at)	standard	reference gene	See above.
MTCH1(221619_s_at)	standard	reference gene	See above. Use custom CDF probe set.
CYFIP1(208923_at)	standard	reference gene	See above. Use custom CDF probe set.
DHX15(201385_at)	standard	reference gene	See above.
HNRNPC(200014_s_at)	standard	reference gene	See above.
NDUFB8(4714_at)	custom	reference	See above.

		gene	
