# Supplementary material for: A robust prognostic signature for hormone-positive node-negative breast cancer
Source: Genome Med. 2013 Oct 11;5(10):92. doi: 10.1186/gm496 (PMC3961800; doi:10.1186/gm496)
Supplement: Additional file 9 — Contains actual probe sequences for all reference probe sets. [file gm496-S9.docx]

**Appendix 6A. Probe sequences for top 25 reference probe sets (Set #1)**

**NOTE: Also includes probe sets which failed manual review.**

>NACAP1_probe1

AGTGAGCTGAGATTGCACCACGGCA

>NACAP1_probe2

TGGGGAGCAAAGGCAGTCCGAGCCC

>NACAP1_probe3

GCAGTCCGAGCCCTGAAGAACAGTA

>NACAP1_probe4

ATGTAACCATCTGAAAGCAACTTTT

>NACAP1_probe5

CAACTTTTTTGGTGTCTCAGATGAG

>NACAP1_probe6

GATGAGTAACTGCAGCTTGGTTTGA

>NACAP1_probe7

AAAGTTATGGCTTCTTGTTGGATGA

>NACAP1_probe8

GTGGAAGATGGCTCTGATTTCAGGG

>NACAP1_probe9

AAAATTTGCTGAATGCTTGCTACGA

>NACAP1_probe10

GAATGCTTGCTACGAGCGGTGGCTC

>NACAP1_probe11

CTCATGCCTGAATCTCAGCACTTTG

>NACAP1_target

tggggagcaaaggcagtccgagccctgaagaacagtaatgatattgtaaatgctattatggaattaacaatgtaaccatctgaaagcaacttttttggtgtctcagatgagtaactgcagcttggtttgaaatttgtactgtttctatcataaataaagttatggcttcttgttggatgaaaaaaaaaagacagtattttgccaaattaaacagaaaagagagttcagtggaagatggctctgatttcagggtaataataatggtaggtaacaattaaatggcatttaaaaatttgctgaatgcttgctacgagcggtggctcatgcctgaatctcagcactttgggaggccaaggcaggcagatcatctgaggtcatgagttcgagaccagcctggccaacatgatgaaaccctgtctctactaaaaatacaaaaattagctgggcatggtggtgcacacctgtaatcccagttactcaggaggctgaggcaggagaatcacttgaatccagaaggcggaggttgcagtgagctgagattgcaccacggca

>PTMA_probe1

TTTTTTTCCTTTTCTGTCTATGAAG

>PTMA_probe2

ATGCCACCCGCAGATGACATGCGCT

>PTMA_probe3

CCACCCAACCCAAACCATGAGAATT

>PTMA_probe4

GGGTCAGCCATTTTTAATGATCTCA

>PTMA_probe5

TGATCTCAGATGACCAAACCAGCCT

>PTMA_probe6

ACCAGCCTTCAGAGCGTTCTCTGTC

>PTMA_probe7

TCCTGCTTCTAACGTCACTTGTGAT

>PTMA_probe8

ACTTGTGATGTGACCATGTTCGTTA

>PTMA_probe9

AAACAATCTTATTCTGAGCATTCCA

>PTMA_probe10

GAGCATTCCAGTAACTTTTTTGTGT

>PTMA_probe11

TTGTGTGTGCGTACTTAGCTGTACT

>PTMA_target

actggctgctctgaaaagccatctttgcattgttccttgtccggctccttgctcgccgcagccgcctttaccgctgcggactccggacacttcatcaccacagtccctgaactctcgctttctttttaatcccctgcatcggatcactggtgtgccggaccatgtcagacgcagccgtagacaccagctccgaaatcaccaccaaggacttaaagaagaaggaagctgtggaggaagcggaaaatggaagagacacccctgctaatgggaaggctaatgaggaaaatggggagcaggaagctgacaatgaagtagatgaagaagaggaagaaggtggggaggaagacgaggaggaagaagaaggcgatggtgaggaagaggatggtgatgaagacgaggaagctgagtccgctaggtcaagcgggcagctgaagatgatgagaatgatgatgcctataccaagaagcagaagaccaacaaggatgactagacagcaaaaaaggaaatgttaggagggtgacctattcaccctccacttcctgtctcagaatctacatgtggtcacctttgagtattgaggcccgccgcccaccgagggcaatgccacccgcagatgacatgcgctctccaccacccaacccaaaccatgagaatttgcaacaggggaggaaaaaagaaccaaaacttcccaaggccctgcttttttccttaaaaatactttaaaaaggaagtttgtttgcattttttaatttacattttatatttttggacatattgttagggtcagccatttttaatgatctcagatgaccaaaccagccttcagagcgttctctgtcctgcttctaacgtcacttgtgatgtgaccatgttcgttataatctcaaaggagaaaaaaaccttgtaagacaagcaaaaacgacaacagaaaaacaatcttattctgagcattccagtaacttttttgtgtgtgcgtacttagctgtactataagtagttggtttgtatgagatggttaaaagggccaaagataaaaggtttcttttttttccttttctgtctatgaagttgctgtttatttatttatttattttttgcctatttgaggtatgtgtgaaacaatgttgtccaacaataaacaggaattttattttcctgagttgttcta

>RPL7_probe1

AGCGAAGCACTGTCACAAGGAACAT

>RPL7_probe2

GCTGGCAACTTCTATGTACCTGCAG

>RPL7_probe3

GGCATTTGCCATCAGGATCAGAGGT

>RPL7_probe4

CGAAAGGTGTTGCAGCTTCTTCGCC

>RPL7_probe5

CTTCGCCTTCCTGAAATCTTCAATG

>RPL7_probe6

GAGTCATATATTGCTTGGGGGTACC

>RPL7_probe7

GACAGATAACACTTTGATTGCTCGA

>RPL7_probe8

GATTGCTCGATCTCTTGGTAAATAT

>RPL7_probe9

GTAAATATGGCATCATCTGCTGGAG

>RPL7_probe10

AAGCAAATAACTTCCTGTGGCCCTT

>RPL7_probe11

TGTGGCCCTTAAAATTATCTTCTCC

>RPL7_target

agcgaagcactgtcacaaggaacataggcagatggacagaactgaaattcgaatggcgagggtggcaagaaaagctggcaacttctatgtacctgcagaacccaaattggcatttgccatcaggatcagaggtatcaatggggtaagcccaaaggtccgaaaggtgttgcagcttcttcgccttcctgaaatcttcaatggaacg/Users/oanatttgtgaagctcaacagggcttcaattaacatgctgaggattgtagagtcatatattgcttgggggtacccaaatctaaagtcactaaatgaacttatctacaagcgtggttatggagaattcagtaagaagcgaattgctttgacagataacactttgattgctcgatctcttggtaaatatggcatcatctgctggaggatctgattcatgagatctatactgttggaaaatgcttcaaagaagcaaataacttcctgtggcccttaaaattatcttctcc

>MYL12B_probe1

GTTACATTGTCTTACTCTCTTTTAC

>MYL12B_probe2

GTTACATTGTCTTACTCTCTTTTAC

>MYL12B_probe3

GAGGCCCCAGGGCCAATCAATTTCA

>MYL12B_probe4

GTACCATTCAGGAAGATTACCTAAG

>SFRS3_probe1

GAAACACAGGCCATCAGGGAAAACG

>SFRS3_probe2

GAAAAATCCAACTCTCATCCTGGGC

>SFRS3_probe3

CATCCTGGGCAGAGGTTGCCTAGTT

>SFRS3_probe4

GATACATGGCTGTTCGTGACATTCT

>SFRS3_probe5

AATGTCCTGCCAGTTTAAGGGTACA

>SFRS3_probe6

GGGTACATTGTAGAGCCGAACTTTG

>SFRS3_probe7

GAGCCGAACTTTGAGTTACTGTGCA

>SFRS3_probe8

TACTTTACAATGTTCCCTTAAGCAA

>SFRS3_probe9

GATAATAAACCTCTAAACCTGCCCA

>SFRS3_probe10

AACCTGCCCAGCGGAAGTGTGTTTT

>SFRS3_probe11

TACTTTTTTTTCCATAGCTGGGATA

>SFRS3_target

tactttacaatgttcccttaagcaaaattgaatttgctttgaacttttagttatgcacagactgataataaacctctaaacctgcccagcggaagtgtgtttttttttaaatttaaatacagaaacaactggcaaaaattgaactaagatttacttttttttccatagctgggatataggctgcagctatagttgaacaagcagtctttaaaaactgctgtgaaacacaggccatcagggaaaacgaaatgctgcactattaaattagaggtttttgaaaaatccaactctcatcctgggcagaggttgcctagttggtatagaatgttaagtttcaagaaagtttacctttgctttaggtcataagttccttatttgattgctgtatatggatacatggctgttcgtgacattctttatgtgcaaatttgtgatttcaaaaatgtcctgccagtttaagggtacattgtagagccgaactttgagttactgtgca

>CLTA_probe1

CAAGAGTAGCCTCAACCTGTGCTTC

>CLTA_probe2

CAGGGTGGCAGATGAAGCTTTCTAC

>CLTA_probe3

ACAACCCTTCGCTGACGTGATTGGT

>CLTA_probe4

ACCATCCTTGCTACAGCCTAGAACA

>CLTA_probe5

TGACATTGACGAGTCGTCCCCAGGC

>CLTA_probe6

TAACCCCAAGTCTAGCAAGCAGGCC

>CLTA_probe7

GCAAGCAGGCCAAAGATGTCTCCCG

>CLTA_probe8

GCCACCCTGTGGAAACACTACATCT

>CLTA_probe9

ATCTGCAATATCTTAATCCTACTCA

>CLTA_probe10

GAAGCTCTTCACAGTCATTGGATTA

>CLTA_probe11

TGTTTGTGATTGCATGTTTCCTTCC

>CLTA_target

cagggtggcagatgaagctttctacaaacaacccttcgctgacgtgattggttatgtcacaaacataaaccatccttgctacagcctagaacaggcagcagaagaagcctttgtaaatgacattgacgagtcgtccccaggcactgagtgggaacgggtggcccggctgtgtgactttaaccccaagtctagcaagcaggccaaagatgtctcccgcatgcgctcagtcctcatctccctcaagcaggccccgctggtgcactgaagagccaccctgtggaaacactacatctgcaatatcttaatcctactcagtgaagctcttcacagtcattggattaattatgttgagttcttttggaccaaacctttttgtctttagagttgttcattgtttgtgattgcatgtttccttccttcaactgtgttctccctggcattcagagaggagggagaggaggaagaggaaggggagggaagcttcccaagagtagcctcaacctgtgcttc

>TRA2B_probe1

TACTTTTCTTTCTAACATATCAATG

>TRA2B_probe2

ATACCATACTTATATACCTGCAACT

>TRA2B_probe3

ATGCTCTGTAACTCTGTACTGCTAG

>TRA2B_probe4

AATACAGCCAGTGCTTAATGCTTAT

>TRA2B_probe5

AATGTGGATTTGTCGGCTTTTATGT

>TRA2B_probe6

GCAAGTGACAATACATTCCACCACA

>TRA2B_probe7

AATACACTCTTGTTCTTCTAGCTTT

>TRA2B_probe8

AAACCGGGTGCTTCAAAGTACATGA

>TRA2B_probe9

GGAACACTATACCTGTCATGGATGA

>TRA2B_probe10

GGATGAACTGAAGACTTTGCCTGTT

>TRA2B_probe11

GGAGGCCCAATTTCACTCAAATGTT

>TRA2B_target

tacttttctttctaacatatcaatgcttagcagaactattcagattgtcagtagtaaatttaaagacaaatgcccgttttcctccagtccatgaaacataccatacttatatacctgcaactaagtgtttaaaattatgctctgtaactctgtactgctagtattagaactaaaaatcttaaaatacagccagtgcttaatgcttatatcaatgtggatttgtcggcttttatgtaatctgtaatatgtatagcaggaaatacgaagagttacacagtgtatgccttaaaaggctgtttcttaaaggtgttacaaggggataatggtatttcaactagttatcagcaagtgacaatacattccaccacaaatacactcttgttcttctagcttttagactatatgaaaaaaccgggtgcttcaaagtacatgataagggaacactatacctgtcatggatgaactgaagactttgcctgttcattttttaaatattattttcaggtcctttgcttaccaaaggaggcccaatttcactcaaatgtt

>RPS2_probe1

TCTCGGCTCTGCATAGTTGCACTTG

>RPS2_probe2

TCGGCTCTGCATAGTTGCACTTGGC

>RPS2_probe3

GGCTCTGCATAGTTGCACTTGGCTT

>RPS2_probe4

GCTCTGCATAGTTGCACTTGGCTTC

>RPS2_probe5

TCTGCATAGTTGCACTTGGCTTCAC

>RPS2_probe6

AGAGATCATTGATTTCTTCCTGGGG

>RPS2_probe7

AGATCATTGATTTCTTCCTGGGGGC

>RPS2_probe8

CTGGGGGCCTCTCTCAAGGATGAGG

>RPS2_probe9

GGGCCTCTCTCAAGGATGAGGTTTT

>RPS2_probe10

GAGGTTTTGAAGATTATGCCAGTGC

>RPS2_probe11

TGCCAGTGCAGAAGCAGACCCGTGC

>RPS2_target

agagatcattgatttcttcctgggggcctctctcaaggatgaggttttgaagattatgccagtgcagaagcagacccgtgccggccagcgcaccaggttcaaggtacccggctgtcctgggagggggctgcgctgggcttgccgggactctctcggctctgcatagttgcacttggcttcac

>MTCH1_probe1

GTTTTTCTCAACACTACTTTTCTGA

>MTCH1_probe2

GCTCAGCTGGGAGCATCATTCTCCT

>MTCH1_probe3

GCTCAGCTGGGAGCATCATTCTCCT

>MTCH1_probe4

GAGAATGGCTTATGGGGGCCCAGGT

>MTCH1_probe5

GTTTAATGGTGATGCCTCGCGTACA

>MTCH1_probe6

TCTCTAGTCCTACCCAGTTTTAAAG

>MTCH1_probe7

GCCTCGCGTACAGGATCTGGTTACC

>MTCH1_probe8

GTTGGGCAGATCAGTGTCTCTAGTC

>MTCH1_probe9

CACCATCATGTCTAGGCCTATGCTA

>MTCH1_probe10

GACCTCATCTCCCGCAAATAAATGT

>HDLBP_probe1

AACGCCCGCAGCACAACGAAGAGGC

>HDLBP_probe2

CCGCAGCACAACGAAGAGGCCAATG

>HDLBP_probe3

AAGAGGCCAATGGGCACTCTTCCAG

>HDLBP_probe4

CACTCTTCCAGAGGCTTTGTGGTGC

>HDLBP_probe5

TCCAGAGGCTTTGTGGTGCGGGACC

>HDLBP_probe6

ACCTGCTCCACTGTTTAACACTAAA

>HDLBP_probe7

AACCAAGGTCATGAGCATTCGTGCT

>HDLBP_probe8

TAAGATAACAGACTCCAGCTCCTGG

>HDLBP_probe9

TAGGATTCCACTTCCTGTGTCATGA

>HDLBP_probe10

CCACTTCCTGTGTCATGACCTCAGG

>HDLBP_probe11

GACCTCAGGAAATAAACGTCCTTGA

>HDLBP_target

aacgcccgcagcacaacgaagaggccaatgggcactcttccagaggctttgtggtgcgggaccgcantccnngnancgcnnnnannnngtgagaanggctcnctgancatgagcangctcnngaggaatttcccagcnnttgngggctcaggtggctcncncaaganccctcnccttggggccccananacgatnangatcananaaganacagaacccntctccagcctgntganccgaaccnnaancannacaantggnttgtctcaatctngacccnnnngnntggaccctccgtaaattnttgacgctcttcccnccttcccgaggtcccgcnagggagcnctagncgncctggctgtngtngtgcnggncgctcctnncaggcctggccgtgcccgctcaggacctgctccactgtttaacactaaaccaaggtcatgagcattcgtgctaagataacagactccagctcctggtccacccggcatgtcagtcagcactctggccttcatcacgaganntccgcagccgtggctaggattccacttcctgtgtcatgacctcaggaaataaacgtccttga

>CYFIP1_probe1

TGGGATGTTCTGGCAGCTGTGTCAT

>CYFIP1_probe2

TTGTTGCCATCACGTTCCTACAAAA

>CYFIP1_probe3

GCCTTTCTCTCCGTAAACTATTTAG

>CYFIP1_probe4

AATAGTGAACTTGATTCCCCTGCTT

>CYFIP1_probe5

ATGCTGCTGGGTTCATTCATTCATT

>CYFIP1_probe6

CTGCTTCCACTAAATCCAGTTGTGA

>CYFIP1_probe7

GCACTCCGTAACTCAACATGGCATG

>CYFIP1_probe8

GAGAATATTGGCTGCTGATTGTTGC

>CYFIP1_probe9

GTTTAGGGATCTTTCTGATGGTCTT

>CYFIP1_probe10

TTTTCAGTATCTCTGTACCTGTTAA

>CYFIP1_probe11

CTTAGTTCTAAGTCATTGTTCCCAT

>SUMO1_probe1

AAATCTTGTCAGAAGATCCCAGAAA

>SUMO1_probe2

AAAGTTCTAATTTTCATTAGCAATT

>SUMO1_probe3

ATTTGTACTTTTTGGCCTGGGATAT

>SUMO1_probe4

GCCTGGGATATGGGTTTTAAATGGA

>SUMO1_probe5

AATGGACATTGTCTGTACCAGCTTC

>SUMO1_probe6

CATTGTCTGTACCAGCTTCATTAAA

>SUMO1_probe7

AATGACCTTTCCTTAACTTGAAGCT

>SUMO1_probe8

GACCTTTCCTTAACTTGAAGCTACT

>SUMO1_probe9

GAGGGTCTGGACCAAAAGAAGAGGA

>SUMO1_probe10

AGGTGAGAGTAATGACTAACTCCAA

>SUMO1_probe11

CTAACTCCAAAGATGGCTTCACTGA

>SUMO1_target

aatgacctttccttaacttgaagctacttttaaaatttgagggtctggaccaaaagaagaggaatatcaggttgaagtcaagatgacagataaggtgagagtaatgactaactccaaagatggcttcactgaagaaaaggcattttaagattttttaaaaatcttgtcagaagatcccagaaaagttctaattttcattagcaattaataaagctatacatgcagaaatgaatacaacagaacactgctctttttgattttatttgtactttttggcctgggatatgggttttaaatggacattgtctgtaccagcttcattaaa

>DHX15_probe1

AAGTTCGAGTTGTGCTCTTCACGTT

>DHX15_probe2

TGTGCTCTTCACGTTGGTTCGATAA

>DHX15_probe3

CACGTTGGTTCGATAATGGCCTTTA

>DHX15_probe4

GTAAATATTCCATTCTGATTTCATA

>DHX15_probe5

ATTAAACATTTATGCCTCCCTTTTG

>DHX15_probe6

CCTCCCTTTTGTGTTGACACTGTAG

>DHX15_probe7

GTTGACACTGTAGCTCATACTGGAA

>DHX15_probe8

GTGATTATCGACCATGGTATGCATG

>DHX15_probe9

GGTATGCATGATCGTTGTAATTGTT

>DHX15_probe10

TTTTTTGTTTCAGTACCAGAGGCAC

>DHX15_probe11

GTACCAGAGGCACTGACTTCAATAA

>DHX15_target

aagttcgagttgtgctcttcacgttggttcgataatggcctttatttgaaagctttttaatttttctttacagtaaatattccattctgatttcataaattaaacatttatgcctcccttttgtgttgacactgtagctcatactggaaaagtcgatcaatgttttgcagtttattgaaagtagttctatatataacaatgttataagcatttctttagaaatggttgaaaatgcttctaaaatgtgattatcgaccatggtatgcatgatcgttgtaattgttgacattccttttagaagttgtgaaatgttacaacttgtgcttatgtagacacaattttttgtttcagtaccagaggcactgacttcaataa

>HNRNPC_probe1

AATAATCTCTTGTTATGCAGGGAGT

>HNRNPC_probe2

TATGCAGGGAGTACAGTTCTTTTCA

>HNRNPC_probe3

TCTTTTCATTCATACATAAGTTCAG

>HNRNPC_probe4

TAAGTTCAGTAGTTGCTTCCCTAAC

>HNRNPC_probe5

GTTGCTTCCCTAACTGCAAAGGCAA

>HNRNPC_probe6

ACTGCAAAGGCAATCTCATTTAGTT

>HNRNPC_probe7

GAGTAGCTCTTGAAAGCAGCTTTGA

>HNRNPC_probe8

AGAAGTATGTGTGTTACACCCTCAC

>HNRNPC_probe9

TGCTGTGTGGGGCAGTTCAACACAA

>HNRNPC_probe10

GTTGGCATGTCAAATGCATCCTCTA

>HNRNPC_probe11

ACAGCCTGATGTTTGGGACCTTTTT

>HNRNPC_target

aataatctcttgttatgcagggagtacagttcttttcattcatacataagttcagtagttgcttccctaactgcaaaggcaatctcatttagttgagtagctcttgaaagcagctttgagttagaagtatgtgtgttacaccctcacattagtgtgctgtgtggggcagttcaacacaaatgtaacaattatttttgtgaatgagagttggcatgtcaaatgcatcctctagaaaaataattagtgttatagtcttaagatttgttttctaaagttgatactgtgggatttttgtgaacagcctgatgtttgggaccttttt

>UBE2D3_probe1

GTTGGGATTTGCTTCATTGTTTGAC

>UBE2D3_probe2

TGCACAGTCTGTTACAGGTTGACAC

>UBE2D3_probe3

TTGACACATTGCTTGACCTGATTTA

>UBE2D3_probe4

TAGTGTAGCTTTAATGTGCTGCACA

>UBE2D3_probe5

GTGCTGCACATGATACTGGCAGCCC

>UBE2D3_probe6

ACTGGCAGCCCTAGAGTTCATAGAT

>UBE2D3_probe7

GTTCATAGATGGACTTTTGGGACCC

>UBE2D3_probe8

GGGACCCAGCAGTTTTGAAATGTGT

>UBE2D3_probe9

GCAGCCCCTGTCTAACTGAAATTTC

>UBE2D3_probe10

CTAACTGAAATTTCTCTTCACCTTG

>UBE2D3_probe11

CTTCACCTTGTACACTTGACAGCTG

>UBE2D3_target

gttgggatttgcttcattgtttgacatcaaatgatgatgtaaagttcgaaagagtgaatattttgccatgttcagttaaagtgcacagtctgttacaggttgacacattgcttgacctgatttatgcagaattaataagctatttggatagtgtagctttaatgtgctgcacatgatactggcagccctagagttcatagatggacttttgggacccagcagttttgaaatgtgtttatggagtttaagaaatttattttccaggtgcagcccctgtctaactgaaatttctcttcaccttgtacacttgacagctg

>DAZAP2_probe1

AGAGTGTCTGATGCGGCCACTCATT

>DAZAP2_probe2

TGAAGCCGCCCTAAGGATTTTCCTT

>DAZAP2_probe3

GGGGAACTTCTTCATGGGTGGTTCA

>DAZAP2_probe4

TTGTGTGTTCTGTACATGTGATGTT

>DAZAP2_probe5

CTCCCAATGCTGCTCAGCTTGCAGT

>DAZAP2_probe6

GAGGAGGATGCATTTCAAAAGCTTG

>DAZAP2_probe7

GATGTCGTGCAAACTGTACTGTGAA

>DAZAP2_probe8

ATAGGTTGTCTCTGCATACACGAAC

>DAZAP2_probe9

GATTCTTTACTTAGCTTGTTTTTAG

>DAZAP2_probe10

ATTTATATCCCATCTAGAATTCAGC

>DAZAP2_probe11

TGCAGTCATGCAGGGAGCCAACGTC

>DAZAP2_probe12

GTGGTGCACTTAACTTGTGGAATTT

>DAZAP2_probe13

GTTTGACTGTACCATTGACTGTTAT

>DAZAP2_probe14

GATGAAGTTGCATTACACCTCACTG

>DAZAP2_probe15

AAGTTCAGCGTTGTATGTCTCTCTC

>DAZAP2_probe16

AATACTGTACCATACTGGTCTTTGC

>DAZAP2_probe17

TCTTTCTGGTGCCCAAACTTTCAGG

>DAZAP2_probe18

TACACGAACCTAACCCAAATTTGCT

>DAZAP2_probe19

GAATTCAGCTAGGTGCTGCTGCTGC

>DAZAP2_probe20

ACGTCCTCGTAACTCAGCGGAAGGG

>DAZAP2_probe21

CTCTCTCTACACTGTGGTGCACTTA

>DAZAP2_probe22

AATGACTTGAGTCCAGTGAAATCTC

>DAZAP2_probe23

TAGCAGTACCTCCCTAAAGCATTTT

>DAZAP2_probe24

ACACCTCACTGCAAGGATTCTTTAC

>DAZAP2_probe25

GGCTCCCCAGAATTCCTAGACTGGG

>DAZAP2_probe26

CTCTGTTTCCTTTGATGACGCTTTG

>SNRNP200_probe1

GAAGTCACAGGCCCTGTCATTGCGC

>SNRNP200_probe2

GATGCCAAGTCCAATAGCCTCATCT

>SNRNP200_probe3

CCCACAACTACACTCTGTACTTCAT

>SNRNP200_probe4

GGAGTACAAATTCAGCGTGGATGTG

>SNRNP200_probe5

GATTCAGATTGAGTCCTGAGGCATT

>SNRNP200_probe6

GTAGGAATCCTGGTTGTGGGGACCA

>SNRNP200_probe7

ACTCTGGATCCAGTGACAGCAGGTG

>SNRNP200_probe8

ACAGCAGGTGTCATGGGTCAAGCAT

>SNRNP200_probe9

AATCATATATAGCATTTTCAGGCAT

>SNRNP200_probe10

GGCATGTTCCTGGTAGTTCTTTTGA

>SNRNP200_probe11

CTGGTAGTTCTTTTGAGTCTGACAT

>SNRNP200_target

gaagtcacaggccctgtcattgcgcctctcttcccgcagaaacgtgaagagggctggtgggtggtgattggagatgccaagtccaatagcctcatctccatcaagaggctgaccttgcagcagaaggccaaggtgaagttggactttgtggccccagccactggtgcccacaactacactctgtacttcatgagtgacgcttacatgggatgtgaccaggagtacaaattcagcgtggatgtgaaagaagctgagacagacagtgattcagattgagtcctgaggcatttacttttgggtaaaggagagttgagcctgaattaggaatgtgtacattgtaggaatcctggttgtggggaccaggtctgtgggcctcaggtctggccagccagggctggtgctgtccccgcctacctccacttcctttcccttgctcactctggatccagtgacagcaggtgtcatgggtcaagcataaatcatatatagcattttcaggcatgttcctggtagttcttttgagtctgacat

>YTHDC1_probe1

GTATGATGGTTTGACTGTATGGCAG

>YTHDC1_probe2

GGATCTTGATTGATAACTGCCATGA

>YTHDC1_probe3

GTGTGTTCATCCTAGAGTTATTTTT

>YTHDC1_probe4

CCTTCCCCTCCAAATTGTATACATT

>YTHDC1_probe5

TGTTGTAGCAGCCTCTTGTTTTTTT

>YTHDC1_probe6

GCGTGGCAGCGGAAGACGATTCCCA

>YTHDC1_probe7

AATTCCTATGTTCAGTAGCGTGGTT

>YTHDC1_probe8

AACTGCCATGATATTTTGCTTTGAT

>YTHDC1_probe9

ACATGTAGTTGCACACGGTTCAGTA

>YTHDC1_probe10

TTTCCTCAGTCTTCAATGACGAGAG

>YTHDC1_probe11

ATGTTCACAACTTGCGTGCGTGGCA

>COPB1_probe1

AGTCCTTGAAGCTTTACAGTTAATT

>COPB1_probe2

ACCTTTATGCTCGTTCCATATTTGG

>COPB1_probe3

TATGGCAGCCAACCTTTATGCTCGT

>COPB1_probe4

GTTTCATGTACCAAGACCCTTTTCA

>COPB1_probe5

GTTTGTCTTTTGTCTTAACAGTTCT

>COPB1_probe6

GAATGCTGTCCTCAAAGTATATAAT

>COPB1_probe7

TGCTGTCCTCAAAGTATATAATGTT

>COPB1_probe8

GATGCACTTGCAAATGTCAGCATTG

>COPB1_probe9

ACCAAGACCCTTTTCACAGTACAAT

>COPB1_probe10

GAATACTTTTCAGCCAATAATTTAT

>COPB1_probe11

GACCCTTTTCACAGTACAATAAACA

>COPB1_probe12

TAATGTTTCATGTACCAAGACCCTT

>COPB1_probe13

CTGCTGTTACCGGCCATATAAGAAT

>COPB1_probe14

CATGTACCAAGACCCTTTTCACAGT

>COPB1_probe15

AAATGACTACTTACAGCACATATTA

>COPB1_probe16

GGTATGGGCTTACTGGACTCCAACA

>COPB1_probe17

TAACAGTTCTGAATGCTGTCCTCAA

>COPB1_probe18

GTCTTTTGTCTTAACAGTTCTGAAT

>COPB1_probe19

GAAGCCAATTCACCAGGGACCAGAT

>COPB1_probe20

ACTCCAACATCTTTTGTACTCTTTC

>COPB1_probe21

AGTTCTGAATGCTGTCCTCAAAGTA

>COPB1_probe22

GCCCTTTCTGGTTACTGTGGCTTTA

>NDUFB8_probe1

GAGATCTGAGGAGGCTTCGTGGGCT

>NDUFB8_probe2

GCACTGGCACCTAGACATGTACAAC

>NDUFB8_probe3

CGGGTGGTTCACTATGAGATCTGAG

>NDUFB8_probe4

TGGTATAGCTGGGACCAGCCGGGCC

>NDUFB8_probe5

TGGGTCCTCTAACTAGGACTCCCTC

>NDUFB8_probe6

CCCTGACCGCTCACAGCATGAGAGA

>NDUFB8_probe7

GCCGGGCCTGAGGTTGAACTGGGGT

>NDUFB8_probe8

CGGTGATCCCTCCAAAGAACCAGAG

>NDUFB8_probe9

TACGAACCTTACCCGGATGATGGCA

>NDUFB8_probe10

ACACCTGTTTCTTGGCATGTCATGT

>NDUFB8_probe11

AACCGTGTGGATACATCCCCCACAC

>NDUFB8_probe12

TCATGTGCTGGGTGGGGGACGTGTA

>NDUFB8_probe13

TCTAACTAGGACTCCCTCATTCCTA

>NDUFB8_probe14

GGACTCCCTCATTCCTAGAAATTTA

>NDUFB8_probe15

CATGACCAAGGACATGTTCCCGGGG

>NDUFB8_probe16

GATCCCTCCAAAGAACCAGAGCGGG

>NDUFB8_probe17

CCAGAGCGGGTGGTTCACTATGAGA

>SET_probe1

ATTGGCCTTTTACCTGGATATAAAT

>SET_probe2

ACCATCCAACAGACCTGGTGCTCTA

>SET_probe3

CCATCCAACAGACCTGGTGCTCTAA

>SET_probe4

TGCTCTAATGCCAAGTTATACACGG

>SET_probe5

ATAGGCTCTCAGTAAGAAGTCTGAT

>SET_probe6

GGTATAAAGCTCTCAAATGTGACCA

>SET_probe7

AAGCTCTCAAATGTGACCATGTGAA

>SET_probe8

TAATGGACTCAGCTCTGTCTGCTCA

>SET_probe9

AATGCCATTGTGCAGAGAAGCACCC

>SET_probe10

GAAGCACCCTAATGCATAAGCTTTT

>SET_probe11

CTAATGCATAAGCTTTTTAATGCTG

>SET_probe12

AATTAAATGCCACTTTTTCAGAGGT

>SET_probe13

CCACTTTTTCAGAGGTGAATTAATG

>SET_probe14

TAAATGGAACTATTCCATCAATAGG

>SET_probe15

CACTGTATACCGATCAGGAATCTTG

>SET_probe16

ATACCGATCAGGAATCTTGCTCCAA

>SET_consensus

gcgagcgagcgagagagggggagggagagcgagcgagcgccgggaggaggcggccggnccgagcgggcgcccgcgcgtgtggcgtgagggnaagccgcttnnccnnncnnnnnnnnnncnnnnnncnnnncctccccgctccccccccgaccgcggannagcaccatgtcggcgccggcggcnaaagtcagtaaaaaggagctcaactccaaccacgacggggccgacgagacctcagaaaaagaacagcaagaagcgattgaacacattgatgaagtacaaaatgaaatagacagacttaatgaacaagccagtgaggagattttgaaagtagaacagaaatataacaaactccgccaaccattttttcagaagaggtcagaattgatcgccaaaatcccaaatttttgggtaacaacatttgtcaaccatccacaagtgtctgcactgcttggggaggaagatgaagaggcactgcannnnnnnnnnnnnnnnnnnnnnnnnnnnnnnnnnnnnnnnnnnnnnnnnnnnnnnnnanngatttttattttgatgaaaatccttactttgaaaataaagttctctccaaagaatttcatctgaatgagagtggtgatccatcttcgaagtccaccgaaatcaaatggaaatctggaaaggatttgacgaaacgttcgagtcaaacgcagaataaagccagcaggaagaggcagcatgaggaaccagagagcttctttacctggtttactgaccattcnnnnnnnnnnnnnnnnnagttaggagaggtcatcaaagatgatatttggccaaacccattacagtactacttggttcccgatatggatgatgaagaaggagaaggagaagaagatgatgatgatgatgaagaggaggaaggattagaagatattgacgaagaaggggatgaggatgaaggtgaagaagatgaagatgatgatgaaggggaggaaggagaggaggatgaaggagaagatgactaaatagaacactgatggattccaaccttcctttttttaaattttctccagtccctgggagcaagnngnnnnnnnnntttttttttttttttttttnnnnccctcttgtgctcagtcgccctgttcttgaggtctcttttctctactccatggttctcaatttatttggggggaaataccttgagcagaatacaatgggaaaagagtctctacccctttctgttcgaagttcatttttatcccttcctgtctgaacaaaaactgnnnnnnnnnnnnnnnnnnnnnnnctgtgggaaaaaagaaaaacctgctcccttnnctctgctggaagctggagggtgctaggcccctgtgtagtagtgcatagaattctagcttttttcctcctttctctgtatattgggctcagagagtacactgtgtctctatgtgaatatggacagttagcatttaccaacatgtatctgtctactttctcttgtttaaaaaaagaaaaaaaaancttaaaaaaatggggttatagaaggtcagcaaagggtgggtttgagatgtttgggtgggttaagtgggcattttgacaacatggcttctcctttggcatgtttaattgtgatatttgacagacatccttgcagtttaagatgacacttttaaaataaattctctcctaatgatgacttgagccctgccactcaatgggagaatcagcagaacctgtaggatcttatttggaattgacattctctattgtaattttgttcctgtttatttttaaattttctttttgtttcactggaaaggaaagatgatgctcagttttaaacgttaaaagtgtacaagttgctttgnnttacaataaaactaaatgtgnnnnnnnnaannnnnnnnnnnnnnnttctctcagncataggntatgcttactatgaccttccaagtttgacttgtataacatcactgtcnaaactttgtcaccctaacttcgtattttttgnatacgcactttngcaggatgacctcagggctatgtggattgagtaatgggnatttgaatcaatgtattaatatctccatagctgggaaacgtgggttcaatttgccnattggtttctgaaagtattcacatcatttgggataccagatagctcaatactctctgagtacattgtgcccttgatttttatctccaagtggcagtttttaaaattggccttttacctggatataaattaattgtgcctgccaccaccatccaacagacctggtgctctaatgccaagttatacacgggacnagttgctggcatgtcttcattggctatataaaatgtggccaagaagataggctctcagtaagaagtctgatnnnnnnnnnnnnnnntccctgctttctggtataaagctctcaaatgtgaccatgtgaatctgggtgggataatggactcagctctgtctgctcaatgccattgtgcagagaagcaccctaatgcataagctttttaatgctgtaaaatatagtcgctgaaattaaatgccactttttcagaggtgaattaatggannnnnnnnnnnnnnnnnnnnnnnnnnnnnngtataaaacttgataaatggaactattccatcaataggcaaaagtgtaacaacctatctagatggatagtatgtaatttctgcacaggtctctgtttagtaaatacatcactgtataccgatcaggaatcttgctccaataaaggaacataaagatttnnaaanaaaaaaaaaaaaaaaaaaaaaaaaaaaaaaaaaaaaa

>CELF1_probe1

TTGCCACTATGACCAAACGCACAGT

>CELF1_probe2

AAACGCACAGTCTGTTCTGCAGCAA

>CELF1_probe3

CTGCAGCAACAACGGGATTCAATCA

>CELF1_probe4

TCAACTCAGTCGTGATTCAGCCGTA

>CELF1_probe5

TCAGCCGTAGAAATGCTTTTCCTTT

>CELF1_probe6

TTATCTTGTTTGAGCTTTTCCTTTC

>CELF1_probe7

GAACTTGTGTTGTACTCTGTAGAAA

>CELF1_probe8

GTCCCAATGGGGAACCTAAATCTGT

>CELF1_probe9

GTTTTAATTGCACAGACACATGGAC

>CELF1_probe10

AAAGTCATTTTGTATCTGCCAAGTG

>CELF1_probe11

ATCTGCCAAGTGTGGTACCTTCCTT

>CELF_consensus

cgctaggtagaggcggtggcggattaaagggttcaagagcgtttaatgtgtagggtgattatttttttcaagatgtatgtttaattttgaagtggcaactttctcctctatngcccttagagcgttngcctgtgcacttagactgtcacttcgtgtggcctccaggtcttaccggggcttccgggaggctggctgctttgctcagagagggtgggaagggggcctggagagacncgagaagcagaggtagagcctagaaggtggcagcaggtgggtaagaggcttatttagcacattaggggcagtgagcacctggaggaaggagggcgctcccaatcacccgtaggaggccatctgcacaccaagcggcaattcacctgctggcgcttttcctaggtgacaagcacaatactacagtcttcacactgtttacagccctgggcaccagccacccggcactggctcttcatcacagctctgctcttgcttagctagtggggtgggggaaagggcagggatttgtttttttaattgggtggagagccaaacagctactgtccctgggtgccaagcaagccagttttttggttccctgagggaaactgaccctcctctcttgtggcaccatccagcctcagggtcttggagacttgagtaagaatgtgagtggagggggagaggtgaggagaggagcacagggtggatctgtggagggaagaggttacagggggaggagctgatgatagatcccacccagacttaagctgctggtgggtgggtgagctgggaagtaggactgtccaggnaagggtggagagatgtagctaggggctggggagggggaggtggaagcgctattgagcatcctccacaccaaggttgatgaaggaagggatcccagcagggtttctgctctggggctggcaggttgcctggtattatgcccaaggccgctctgcctgggggaaagggcagccaggcagaggcccagtgtctggtaggctgctgaatttcctggaaggggtgattggatggaaagaggccagaaaccccagcctgagagactgctgtgcaccccacagtctgactgcacagagccgcctctgttggcaggaggcactgaggctccccttcctgtgtattgagaagcagtgtttgccaatatattttgctttcaattccaagaggagctctgggaaaacctgtggataaaaccaaatgccaaatgttggacgttgtttccttttccttttctctctctgattgttttaattgttctgtggtggttttaatggatttgagaccctggagcggcagctgcctttctgatttccagctgctttttgtgaataatttaaaaanaaaaaaaaaaagaaannnnacattttggnagacaaacctgtgtgagttttttattggntacaaacgttgtatttaacactaggggtttngntacagttttttgnccttttctactagaaaacaatgtaaagtgatttcacaatgtgaagagaaaaaaaaattgccactatgaccaaacgcacagtctgttctgcagcaacaacgggattcaatcaactcagtcgtgattcagccgtagaaatgcttttcctttatcttgtttgagcttttcctttctttcctgttttgatttgcaaaagaaaatgtcttttttgtgtgaacttgtgttgtactctgtagaaaattatggattttactttaatggtttaaaaaaaggcaaggagagcccttgtcgcttttcttacctaatcacagagtttgtgtagtgaatttaaaaagaaaaaaaanttgttataagtttggagcaagggagtatgtgtttcaaaggaatctccttcctttttttgtgtgtttttccttttgtcccaatggggaacctaaatctgttttaattgcacagacacatggacaaaaagtcattttgtatctgccaagtgtggtaccttcctttgtttatttgctattaaactgtttgagaagaaaaaaaaaaaaaaaaaaaaananaaanaannnnaannaannnnnnnngntaaagatagaacctagcaagaaaaaattaatggaactttacgaaatgaataactgaataagcggatgaggctaataaaagactttggatcaaaggtatggtaagagagtgattcagaaggcactgaaagagatggaacagtacggagtggctagagaagaactcaaaggagcctgccatgttgtggaactcacatagaga

>XPO1_probe1

TAGGGAGCATTTTCCTTCTAGTCTA

>XPO1_probe2

GCATTGTCTGAAGTTAGCACCTCTT

>XPO1_probe3

GCACCTCTTGGACTGAATCGTTTGT

>XPO1_probe4

GAATCGTTTGTCTAGACTACATGTA

>XPO1_probe5

GATCATGTGCATATCATCCCATTGT

>XPO1_probe6

ATCATCCCATTGTAAAGCGACTTCA

>XPO1_probe7

GTGTGTGCTGTCGCTTGTCGACAAC

>XPO1_probe8

GTCGCTTGTCGACAACAGCTTTTTG

>XPO1_probe9

ATTTGTGAGCCTTCATTAACTCGAA

>XPO1_probe10

GTTAGAATAGGCTGCATCTTTTTAA

>XPO1_probe11

ACAACTCTGGCTTTTGAGATGACTT

>PTBP1_probe1

TTCACCTGCAGTCGCCTAGAAAACT

>PTBP1_probe2

AAACTTGCTCTCAAACTTCAGGGTT

>PTBP1_probe3

AAGTCTCATTTCTGTGTTTTGCCTG

>PTBP1_probe4

CCTCTGATGCTGGGACCCGGAAGGC

>PTBP1_probe5

ATACCTGTTGTGAGACCCGAGGGGC

>PTBP1_probe6

CGGCGCGGTTTTTTATGGTGACACA

>PTBP1_probe7

TCCAGGCTCAGTATTGTGACCGCGG

>PTBP1_probe8

TGCCTTACCCGATGGCTTGTGACGC

>PTBP1_probe9

TGTTCGCTGTGGACGCTGTAGAGGC

>PTBP1_probe10

GTTGGCCAGTCTGTACCTGGACTTC

>PTBP1_probe11

GAATAAATCTTCTGTATCCTCAAAA

>SF3B1_probe1

GTTTACAGGGTCTGTTTCACCCAGC

>SF3B1_probe2

TTCACCCAGCCCGGAAAGTCAGAGA

>SF3B1_probe3

CAACTCCATCTACATTGGTTCCCAG

>SF3B1_probe4

CTCATAGCACATTACCCAAGAATCT

>SF3B1_probe5

GAACACCTATATTCGTTATGAACTT

>SF3B1_probe6

TTAATGCACAGCTACTTCACACCTT

>SF3B1_probe7

CACACCTTAAACTTGCTTTGATTTG

>SF3B1_probe8

AATAACCTGTCTTTGTTTTTGATGT

>SF3B1_probe9

GTAAATGCCAGTAGTGACCAAGAAC

>SF3B1_probe10

TACACTATACTGGAGGGATTTCATT

>SF3B1_probe11

GATTTAGAACTCATTCCTTGTGTTT

>ARPC2_probe1

ACTGGATAATCGTAGCTTTTAATGT

>ARPC2_probe2

GTAGCTTTTAATGTTGCGCCTCTTC

>ARPC2_probe3

GTGACAACATTGGCTACATTACCTT

>ARPC2_probe4

TGCGCCTCTTCAGGTTCTTAAGGGA

>ARPC2_probe5

GCTGTGCTTGCAAAGACTTCATAGT

>ARPC2_probe6

ATCTTCCGGCATCCAAGGATTCCAT

>ARPC2_probe7

GAGCTGAAAGACACAGACGCCGCTG

>ARPC2_probe8

AAAGAAGGACGCAGAGCCAGCCACA

>ARPC2_probe9

ATCTGCAGAAACGAGCTGTGCTTGC

>ARPC2_probe10

GTCTCTTTGCTATATGACCTTGAAA

>ARPC2_probe11

GAGGAAGCGGCTGGCAACTGAAGGC

>ARPC2_probe12

CTCTTTTCCAAGCTGTTTCGCTTTG

>ARPC2_probe13

CGTTTTCATCCCGCTAATCTTGGGA

>ARPC2_probe14

GTTTCGCTTTGCAATATATTACTGG

>ARPC2_probe15

GGAACACTTGCTACTGGATAATCGT

>ARPC2_probe16

GAAGCGAAATTGTTTTGCCTCTGTC

>ARPC2_probe17

GAGTCACAGTAGTCTTCAGCACAGT

>ARPC2_probe18

GGAAGCGGCTGGCAACTGAAGGCTG

>ARPC2_probe19

TGCAGTCATAACTTGTTTTCTCCTA

>ARPC2_probe20

TCCTCTTTAGCCACAGGGAACCTCC

>ARPC2_probe21

GACCTTGAAAATCTTCCGGCATCCA

>ARPC2_probe22

GGATTCCATTGTGCATCAAGCTGGC

>ARPC2_probe23

TTCATCCCGCTAATCTTGGGAATAA

>C2orf28_probe1

CAACATGTCAACTGTCCTGGAGGAA

>C2orf28_probe2

TGTTCTTCGGGATTCTGGGAGCCAC

>C2orf28_probe3

CCAGTGGATTCGCCTCAAGGTTGAG

>C2orf28_probe4

TGAACTATCTTAGCCCAGTCAGGGA

>C2orf28_probe5

GGATCTTGTGTACCTGATGGTCCAG

>C2orf28_probe6

AGTTCCCATTGGTGTTGTTGCCTAT

>C2orf28_probe7

GAACCTTTGCAATAACACTGGGGAC

>C2orf28_probe8

TCCAGGTCTTTTGCAGTGTGTTTGT

>C2orf28_probe9

TTCTCACTGCTTATGTTCTTCGGGA

>VAMP3_probe1

GAGACTCAACATCAGGATCCACAGC

>VAMP3_probe2

ACAGACTTTATCGCTCTGTGGCTCA

>VAMP3_probe3

AAGCAGCAACAGCTGAGGCGCACCA

>VAMP3_probe4

GCTTCCATTTCTTTAACGTCTGTTC

>VAMP3_probe5

TCTGTTCCCTTAACATCGCTGAAAT

>VAMP3_probe6

GAAGAGATGCCTTGCGGTGTGGCCA

>VAMP3_probe7

GACTCAGAAACCTTGGTACTCGCCC

>VAMP3_probe8

ACTGGCTCCTGCATTAACCCAGAAA

>VAMP3_probe9

TAACCCAGAAATACCTCGCTTCTAT

>VAMP3_probe10

CTCGCTTCTATCTGTGCACTTAGCT

>VAMP3_probe11

GGGAACTTACCCACTGTAATCACCT

>STARD7_probe1

TTGTGCCAAGGAAGTAGCTGCCCCA

>STARD7_probe2

CCTTCTCCGCGTCATTGTTGGAAGA

>STARD7_probe3

AGGAGAGATGCATCGAGCAGTCCCA

>STARD7_probe4

GCTGCTTTTCATTTATTACTTCTTC

>STARD7_probe5

CTTCTTCTTTCCAGGACCTGACAGA

>STARD7_probe6

TTATGTCCAAACTTAGCACCTGCAA

>STARD7_probe7

TGTGCGTCTGCGAGCGCACACACAT

>STARD7_probe8

AGGAGTTGCGGTTGCTCCATGTTCT

>STARD7_probe9

GCTCCATGTTCTGACTTAGGGCAAT

>STARD7_probe10

CTGCACTTGGGGTCTGTCTGTACAG

>STARD7_probe11

GTCTGTACAGTTACTCATGTCATTG

>SEC31A_probe1

TTCAGTGAGACCTCTGCTTTCATGC

>SEC31A_probe2

AGCATGTTTGCATAGCAACCAGTCA

>SEC31A_probe3

GTTGCCAGTGATGATTTTCCTATTC

>SEC31A_probe4

ATTTCTGCTGATATACTCACCTTAG

>SEC31A_probe5

GCTGCCTTTCTTCAGCAACAGACCC

>SEC31A_probe6

AGCAACCAGTCAAGAGCATTTACAC

>SEC31A_probe7

TGAAGGTGCCCCAGGGGCTCCTATT

>SEC31A_probe8

AGTATGGTTTCCTGAAGTATTCTGA

>SEC31A_probe9

TAAAACTAAATTTCTTTCATGTCCT

>SEC31A_probe10

TGCTCAGAACCCTGGTGCTTTATTT

>SEC31A_probe11

GCGTACAGCAACCTCTTGGTCAAAC

>SEC31A_probe12

TTCTCTTCCACTCAATATTGCCATT

>SEC31A_probe13

GGACTAGTCCTCATTAGCATGTTTG

>SEC31A_probe14

CATACCCACATAGTTAGCACCAGCA

>SEC31A_probe15

GAGCATTTACACTATTTCTGCTGAT

>SEC31A_probe16

AGAAGGATTGACCATGCATACCCAC

>SEC31A_probe17

GCACCAGCAACTTCAGTGAGACCTC

>SEC31A_probe18

TTGAGGATCTTATTCAGCGCTGCCT

>SEC31A_probe19

GCCAGTTCTCAAAGTTGTTCTCACC

>SEC31A_probe20

TACTCACCTTAGAACTGCTCAGAAC

>SEC31A_probe21

GTATTTCCTGGATTACACATAGTAT

**Appendix 6B. Probe sequences for top 15 reference probe sets (Set #2)**

**NOTE: Also includes probe sets which failed manual review.**

>MFN2_probe1

GTCTATGAGCGTCTGACCTGGACCA

>MFN2_probe2

GTTACTCCTGTATCATTGCTCATAA

>MFN2_probe3

AGCCTCTGTGCACTGTTTGGTGGCC

>MFN2_probe4

TGTATTTAAAGCCCTCAGTCTGTCC

>MFN2_probe5

GCCTGAATGGACAGGGGCCACTTCA

>MFN2_probe6

ATCACTGTCACACAATTCCAATGGA

>MFN2_probe7

GACCTTTGCTCATCTGTGTCAGCAA

>MFN2_probe8

CCCGGCGTGTGCCGGGCCTGAATGG

>MFN2_probe9

GCTGGAGCGCAAGACGTGCTGACAC

>MFN2_probe10

AGGTGATGTCCTGTTCACATACCTG

>MFN2_probe11

GCCTTCAAGCGCCAGTTTGTGGAGC

>MFN2_probe12

CCTCCATGGGCATTCTTGTTGTTGG

>MFN2_probe13

TCATGGTTTCCATGGTTACCGGCCT

>MFN2_probe14

CCCAGCCATCACTCATCTTTGAGGA

>MFN2_probe15

GCGAAGTGATGGACTCTGCCAGGTG

>MFN2_probe16

GCCACTTCACAGCATGTCAGGGAAA

>MFN2_probe17

GTCCTGTTGTGTGGGGCGAAGTGAT

>MFN2_probe18

GTGCTGACACAGTGAGTTTTCTCTG

>MFN2_probe19

GCAGCTTGTCATCAGCTACACTGGC

>MFN2_probe20

GTGTCAGCAAGTTGACGTCACCCGG

>MFN2_probe21

CTGCCAGGTGGACATGCTGTGGGTG

>WIPI2_probe1

CAATGAGATCTTGGACTCTGCCTCT

>WIPI2_probe2

AGATCCCGCGGTTGTTGGTGGGTGC

>WIPI2_probe3

TCTACTTCACTCTTCCTGTTGAAAA

>WIPI2_probe4

CAGACTCTGCATTCCAAACCAAGGC

>WIPI2_probe5

GACTGACTGAACTTGACCTGTGACC

>WIPI2_probe6

AGCAACAGAGAGTAGGCGGCTGGGC

>WIPI2_probe7

TGCCTGGACTCGCTGGAGCAAAGGA

>WIPI2_probe8

CCGCCCATGATTCTTCGGACTGACT

>WIPI2_probe9

GCCCCTTAGTCACTCAGACATACGG

>WIPI2_probe10

CGCCGACGGGTACCTGTACATGTAC

>WIPI2_probe11

GTCATGTGCCTTTCTATTTTCATCT

>WIPI2_probe12

TAGGGGAGCTAGAAGCCACTTTCCA

>WIPI2_probe13

GGGCTTCCTACCTGTGTGAGAGGTC

>WIPI2_probe14

TCGCTTCCCTTTTCATATTTACAGA

>WIPI2_probe15

ACTTGAAAGGTTGCCTGGACTCGCT

>WIPI2_probe16

GCATGAACGTGCCAAGCCAGCATAG

>WIPI2_probe17

CCCTGCGCCTGGATGAGGACAGCGA

>WIPI2_probe18

CAGAACTCAAGTGTGGTGGCCGTCT

>WIPI2_probe19

AATTGGATCGCTCTGGGATTTCTTC

>WIPI2_probe20

GGACAGCGAGGTTCTTTCTGATACT

>WIPI2_probe21

CCCACCAGGTGTGCTGGGCAGACTT

>WIPI2_probe22

AAATGATCTGTTCTTCTACTTCACT

>WIPI2_probe23

AAACAACCTCAAGTACCTCAGACTC

>WIPI2_probe24

GGGCAGACTTCAGCTGGGACAGAAG

>WIPI2_probe25

TTCGGGAAAGTGCTCATGGCCTCCA

>WIPI2_probe26

CAAGCTTCAGTATTTGCCTCGCTTC

>WIPI2_probe27

GCGTAAGGAAACCGTGGCGTCGCGC

>WIPI2_probe28

CCACAAAAACATCTGCTCGCTAGCC

>WIPI2_probe29

TCTGCTTGTCAAGGCCAGTTCTGCA

>WIPI2_probe30

GCGAGTGTGCCCTGATGAAGCAGCA

>WIPI2_probe31

GAGGTCGTAGCGGGAGACAGCAACA

>WIPI2_probe32

TGGGACAGAAGTCCGATCTCCCTAG

>PFDN1_probe1

GGCAGTCTGCCTAAAGATTCCTTTC

>PFDN1_probe2

GCCTTCTCCCATACATTCCAAAAGG

>PFDN1_probe3

GTTCAACAGTAAGCAGCACCTCCAA

>PFDN1_probe4

TCTCCTTTCGGCCAGTATCATAAGA

>PFDN1_probe5

TGGACGCCATAATCCTGAGGCTCCT

>PFDN1_probe6

GGCTCCTAGAGGCTGAGGGGGCAAC

>PFDN1_probe7

TGAGGGGGCAACGGTGTGATCCAGC

>PFDN1_probe8

GCAAGCCAGTTGTCAAACACAGCCA

>PFDN1_probe9

GTGAGAGAGGCAGTGGCCGTCCTCC

>PFDN1_probe10

TTCCTGTACCTTTGACTAACGCTCA

>PFDN1_probe11

CTTCCGGGCCTGCATGCAGTAGACA

>UBE3A_probe1

ATCAGCCATTTTATCGAGGCACGTG

>UBE3A_probe2

TAGCTAATGTGCTGAGCTTGTGCCT

>UBE3A_probe3

TAGACCACGTAACCTTCAAGTATGT

>UBE3A_probe4

GAACTACTCTCCCAAGGAAAATATT

>UBE3A_probe5

TAAGGAAGCGCGGGTCCCGCATGAG

>UBE3A_probe6

GCCATCATCTTGTTGAATCAGCCAT

>UBE3A_probe7

TACAACGGGCACAGACAGAGCACCT

>UBE3A_probe8

TTTACTTCCGGAATACTCAAGCAAA

>UBE3A_probe9

TATGGTGACCAATGAATCTCCCTTA

>UBE3A_probe10

GATTGTTTTAACTGATTACTGTAGA

>UBE3A_probe11

TTCCTAGTCTTCTGTGTATGTGATG

>UBE3A_probe12

AGGATGTCTTTCAGGATTATTTTAA

>UBE3A_probe13

TAATTACTTACTTATTACCTAGATT

>UBE3A_probe14

GACTACAGGAGACGACGGGGCCTTT

>UBE3A_probe15

GACAGAACTGTTTGTTATGTACCAT

>UBE3A_probe16

ACTGTGCCTTGTGTTACTTAATCAT

>UBE3A_probe17

GCGACGAACGCCGGGATTTCGGCGG

>UBE3A_probe18

GTATAGCCCCACAGATTAAATTTAA

>UBE3A_probe19

TTGCCACCATTTGTAGACCACGTAA

>UBE3A_probe20

GAAGACAATGCTTTCCATATTGTGA

>UBE3A_probe21

GCTTTAATGTGCTTTTACTTCCGGA

>UBE3A_probe22

ATTTTTTTGCGTGAAAGTGTTACAT

>UBE3A_probe23

CGGATAAGGAAGCGCGGGTCCCGCA

>UBE3A_probe24

CTGGGCTCGGGGTGACTACAGGAGA

>UBE3A_probe25

AAAGATGGCTACTGTGCCTTGTGTT

>UBE3A_probe26

AAGGCCATCACGTATGCCAAAGGAT

>UBE3A_probe27

GACTCTTCTTGCAGTTTACAACGGG

>UBE3A_probe28

GAGACATTGATATATCCTTTTGCTA

>UBE3A_probe29

GATTACTGTAGATCAACCTGATGAT

>UBE3A_probe30

GGCTCGGGGTGACTACAGGAGACGA

>UBE3A_probe31

GCCTCGTTTTCCGGATAAGGAAGCG

>UBE3A_probe32

CCATTTGTAGACCACGTAACCTTCA

>UBE3A_probe33

TTCGTGTTGCCATCATCTTGTTGAA

>UBE3A_probe34

TTACCTACATCTCATACTTGCTTTA

>UBE3A_probe35

GAGCTTGTGCCTTGGTGATTGATTG

>UBE3A_probe36

CAAGGCTTTTCGGAGAGGTTTTCAT

>UBE3A_probe37

GGGTGACTACAGGAGACGACGGGGC

>UBE3A_probe38

TGTTACATATTCTTTCACTTGTATG

>UBE3A_probe39

GATAAGGTAACATGGGGTTTTTCTG

>UBE3A_probe40

GAATTACATTGTATAGCCCCACAGA

>UBE3A_probe41

GATATATCCTTTTGCTACAAGCTAT

>UBE3A_probe42

TGACGGTGGCTATACCAGGGACTCT

>UBE3A_probe43

GAAACTATTACTCCTAAGAATTACA

>UBE3A_probe44

GCTGGCGACGAACGCCGGGATTTCG

>UBE3A_probe45

ATGCAGCTTTCAAATCATTGGGGGG

>UBE3A_probe46

GAGGCACGTGATCAGTGTTGCAACA

>GTF3C2_probe1

GGGCAGGAGCCTCGCAATATGTGGC

>GTF3C2_probe2

GGCTCCTCAGCCTAAGACTATGGCT

>GTF3C2_probe3

AGAAACACTCAGGCCTGACCTAGGC

>GTF3C2_probe4

TAACCATCATGTATGCCCACGAGGG

>GTF3C2_probe5

TAACCATCATGTATGCCCACGAGGG

>GTF3C2_probe6

GACCCCTCTGAGTGTGGTCAGTGCC

>GTF3C2_probe7

TCCCTGTGATTGCCCTGTTAAGTAT

>GTF3C2_probe8

TCCCTGTGATTGCCCTGTTAAGTAT

>GTF3C2_probe9

TGCTCCTGCTTACGAAGTATTCCCA

>GTF3C2_probe10

TGCTCCTGCTTACGAAGTATTCCCA

>GTF3C2_probe11

GATTGCTTGTGACAACGGCTGCATC

>GTF3C2_probe12

GGCTCCTGTCTGACTATTCCAGGAT

>GTF3C2_probe13

CCCTACCGATAGAACAGTGGCTCAG

>GTF3C2_probe14

GCATGAAGGCTCCTGTCTGACTATT

>GTF3C2_probe15

TGCATCTGGGACCTCAAGTTCTGCC

>GTF3C2_probe16

CCACCAACACCTAGCTGCTGGATAT

>GTF3C2_probe17

CACAGACACCCTACCGATAGAACAG

>GTF3C2_probe18

TGGCCTGCTCAGACGGGAAAGTACT

>GTF3C2_probe19

GTATCTGCATGAAGGCTCCTGTCTG

>GTF3C2_probe20

CAAGGAATACCACAGACACCCTACC

>GTF3C2_probe21

AACCATAGCTATCATGTGTTTCCCA

>GTF3C2_probe22

ATAGCTATCATGTGTTTCCCAAATC

>GTF3C2_probe23

ACAGGGCCCACTTTGTCTATGGGAT

>GTF3C2_probe24

TTCCCAATCACTGGTCATCTGACCC

>GTF3C2_probe25

TTCCCAATCACTGGTCATCTGACCC

>GTF3C2_probe26

GGAAATCTAGTCATCTTCCCTGTGA

>GTF3C2_probe27

GGAAATCTAGTCATCTTCCCTGTGA

>GTF3C2_probe28

GACATGAATGAGACACACCCACTGA

>GTF3C2_probe29

GCAACTCTGCAGGTGGGGTCTATGC

>GTF3C2_probe30

AAGTACTGCTATTCAGTCTACCCCA

>GTF3C2_probe31

TATTACTGCCTTCTGAAACTTCCTC

>GTF3C2_probe32

TATTACTGCCTTCTGAAACTTCCTC

>KHDRBS1_probe1

GTTACTGATTTCTTGTATCTCCCAG

>KHDRBS1_probe2

GCTACATGTGTAAGTCTGCCTAAAT

>KHDRBS1_probe3

AATCTAGCCCCAGACATACTGTGTT

>KHDRBS1_probe4

CCTCCCATTTTGTTCTCGGAAGATT

>KHDRBS1_probe5

GTCCATTTGAGATTCTGCACTCCAT

>KHDRBS1_probe6

CCCCTCCTGCTAGGCCAGTGAAGGG

>KHDRBS1_probe7

TAATTGGATTTGTACCGTCCTCCCA

>KHDRBS1_probe8

GTCAAGTATGTCTCAACACTAGCAT

>KHDRBS1_probe9

GAAAAGTTCACTTGGACGCTGGGGC

>KHDRBS1_probe10

TTGTCAATATATCGAACTGTTCCCA

>KHDRBS1_probe11

TAACTCTGCATTCTGGCTTCTGTAT

>KHDRBS1_probe12

TGTCTAAGTGTTTTTCTTCGTGGTC

>KHDRBS1_probe13

AGGCCTCCTGAATTGAGTTTGATGC

>KHDRBS1_probe14

GACTGGAATGGGACCAGGCCGTCGC

>KHDRBS1_probe15

GATGCAGAGCTTTTTAGCCATGAAG

>KHDRBS1_probe16

ATACAGAGAGCACCCATATGGACGT

>KHDRBS1_probe17

GTAGATGCTTTTTTCTTTGTTGTTT

>KHDRBS1_probe18

TGACTTTTTCATTACGTGGGTTTTG

>KHDRBS1_probe19

GTATCTCCCAGGATTCCTGTTGCTT

>KHDRBS1_probe20

TTGCTTTACCCACAACAGACAAGTA

>KHDRBS1_probe21

CCTTATTCCATTCTTAACTCTGCAT

>RARS_probe1

GTTGAATGACTACATCTTCTCCTTT

>RARS_probe2

AGCTGCTTACTTGTTGTATGCCTTC

>RARS_probe3

GTATGCCTTCACTAGAATCAGGTCT

>RARS_probe4

AATCAGGTCTATTGCACGTCTGGCC

>RARS_probe5

GGAAACTAGGCCGGTGCATTTTACG

>RARS_probe6

GAGCTGGCAACTGCTTTCACAGAGT

>RARS_probe7

GAACATGTGGCGTATCTTGTGTGAA

>RARS_probe8

CTGGCCCAAGGGTGTAATCCCTCAC

>RARS_probe9

AATCCCTCACAGGTTTGAACCCTGT

>RARS_probe10

TTTTCCCAAGTGGCCATTGGCCCTG

>RARS_probe11

GCTTTTTTTCAATCTTGTGGGCACA

>MYL12A_probe1

GCAACTGGCACCATACAGGAAGATT

>MYL12A_probe2

CAAATTCCAGCCAACGTCCTTGTTG

>MYL12A_probe3

AACGTCCTTGTTGCACTTTGGGTAT

>MYL12A_probe4

GCACTTTGGGTATTCTGAGATTTTC

>MYL12A_probe5

TCTTGCCATTCCCTTAGGCTTTAGC

>MYL12A_probe6

GGCTTTAGCAGCTTTGCATTTCCTG

>MYL12A_probe7

TTGCATTTCCTGTTGTATTTATTCT

>MYL12A_probe8

TATTCTCAGCCATTTTGGGCATATG

>MYL12A_probe9

CAGACTGGAAACGGGACTTTCTATT

>MYL12A_probe10

CTTCTCCCCCAATAACTGTGGGTCT

>MYL12A_probe11

TCAGAGAAAGTTAGTTCGGCTCGAT

>HNRNPD_probe1

GATAGTTAATGTTTTATGCTTCCAT

>HNRNPD_probe2

TATTCCATTTGCAACTTATCCCCAA

>HNRNPD_probe3

GCAAAAGTACCCCTTTGCACAGATA

>HNRNPD_probe4

GAAATGCGGCTAGTTCAGAGAGATT

>HNRNPD_probe5

AATTTTTTGTATCAAGTCCCTGAAT

>HNRNPD_probe6

GACAGGCTTGCCGAAATTGAGGACA

>HNRNPD_probe7

AACAGCCAAGGTTACGGTGGTTATG

>HNRNPD_probe8

GTGTCCTCCCTGTCCAAATTGGGAA

>HNRNPD_probe9

GATTCATTTGAAGGTGGCTCCTGCC

>HNRNPD_probe10

ATAATACTTCCTTATGTAGCCATTA

>HNRNPD_probe11

AATGTCAATTTGTTTGTTGGTTGTT

>HNRNPD_probe12

GAGTGGTTATGGGAAGGTATCCAGG

>HNRNPD_probe13

GACTACACTGGTTACAACAACTACT

>HNRNPD_probe14

GGTGGTCATCAAAATAGCTACAAAC

>HNRNPD_probe15

AAGTTTGGAAGACAGGCTTGCCGAA

>HNRNPD_probe16

GGAACCAGGGATATAGTAACTATTG

>HNRNPD_probe17

GAGCTGTGGTGGACTTCATAGATGA

>HNRNPD_probe18

AGTCCCTGAATGGAAGTATGACGTT

>HNRNPD_probe19

AAAAGCCCAGTGTGACAGTGTCATG

>HNRNPD_probe20

GAAGTTTAATTCTGAGTTCTCATTA

>HNRNPD_probe21

GGAGGATATGACTACACTGGTTACA

>HNRNPD_probe22

GAGAGATTTTTAGAGCTGTGGTGGA

>HNRNPD_probe23

TTATTCCATTTGCAACTTATCCCCA

>HNRNPD_probe24

AGGTTACGGTGGTTATGGAGGATAT

>HNRNPD_probe25

ATTTGCTTTCATTGTTTTATTTCTT

>HNRNPD_probe26

AGAAATTTGCTTTCATTGTTTTATT

>HNRNPD_probe27

CCTTTCCCCCAGTATTGTAGAGCAA

>HNRNPD_probe28

GGTATCCAGGCGAGGTGGTCATCAA

>HNRNPD_probe29

GTATGACGTTGGGTCCCTCTGAAGT

>HNRNPD_probe30

GTATGACGTTGGGTCCCTCTGAAGT

>HNRNPD_probe31

AACAACTACTATGGATATGGTGATT

>HNRNPD_probe32

TGTGCTTTTTAGAACAAATCTGGAT

>TARDBP_probe1

GAGAGCGCGTGCAGAGACTTGGTGG

>TARDBP_probe2

TGGCGAGATGTGTCTCTCAATCCTG

>TARDBP_probe3

TCTCTCAATCCTGTGGCTTTGGTGA

>TARDBP_probe4

GTTTTTGTTCTTAGATAACCCACAT

>TARDBP_probe5

TGAAATGATACTTGTACTCCCCCTA

>TARDBP_probe6

CTTTGTCAACTGCTGTGAATGCTGT

>TARDBP_probe7

GAATGCTGTATGGTGTGTGTTCTCT

>TARDBP_probe8

GGACTGAGCTTGTGGTGTGCTTTGC

>TARDBP_probe9

GCAGAGTTCACCAGTGAGCTCAGGT

>TARDBP_probe10

GTTCTAATGTCTGTTAGCTACCCAT

>TARDBP_probe11

AAGAATGCTGTTTGCTGCAGTTCTG

>HNRNPR_probe1

AAGCTAGTGCTTTGTCTTAGTAGTT

>HNRNPR_probe2

GGGGCAATCGTGGGGGCAATGTAGG

>HNRNPR_probe3

TCGTTTCAGGCTTCATTTTAGCTTC

>HNRNPR_probe4

TCACACCTTTTTGAAATCTGCCCTA

>HNRNPR_probe5

ACCCTCCAGATTACTACGGCTATGA

>HNRNPR_probe6

ATTGTTATAACTTCACACCTTTTTG

>HNRNPR_probe7

TGGATATGGCTACCCTCCAGATTAC

>HNRNPR_probe8

AAACAAGCTGGGCACACTGTTAAAT

>HNRNPR_probe9

GCTCTTGGACATTATTGGGCTTGCA

>HNRNPR_probe10

CATGATTTTGCAGAACCTTTGGTTT

>HNRNPR_probe11

CAATGCTTTTATCGTTTCAGGCTTC

>HNRNPR_probe12

GTTCCCGTGGATCTCGGGGCAATCG

>HNRNPR_probe13

GATTCCAAGCGTCGTCAGACCAACA

>HNRNPR_probe14

TCAACAGCAGAGAGGCCGTGGTTCC

>HNRNPR_probe15

GGCTATGAAGATCCCTACTACGGCT

>HNRNPR_probe16

AAAGCCGTGACAATTTGTTCTTTGA

>HNRNPR_probe17

TCACAGAGGGGGGCACCTTTGGGAC

>HNRNPR_probe18

ACCTTTGGGACCACCAAGAGGCTCT

>HNRNPR_probe19

GTATTTCCAATTTCTTGTTCATGTA

>HNRNPR_probe20

GTCGTCAGACCAACAACCAACAGAA

>HNRNPR_probe21

TGGGCTTGCAGAGTTCCCTTATTCT
