# Supplementary figures and images for: A robust prognostic signature for hormone-positive node-negative breast cancer
Source: Genome Med. 2013 Oct 11;5(10):92. doi: 10.1186/gm496 (PMC3961800; doi:10.1186/gm496)

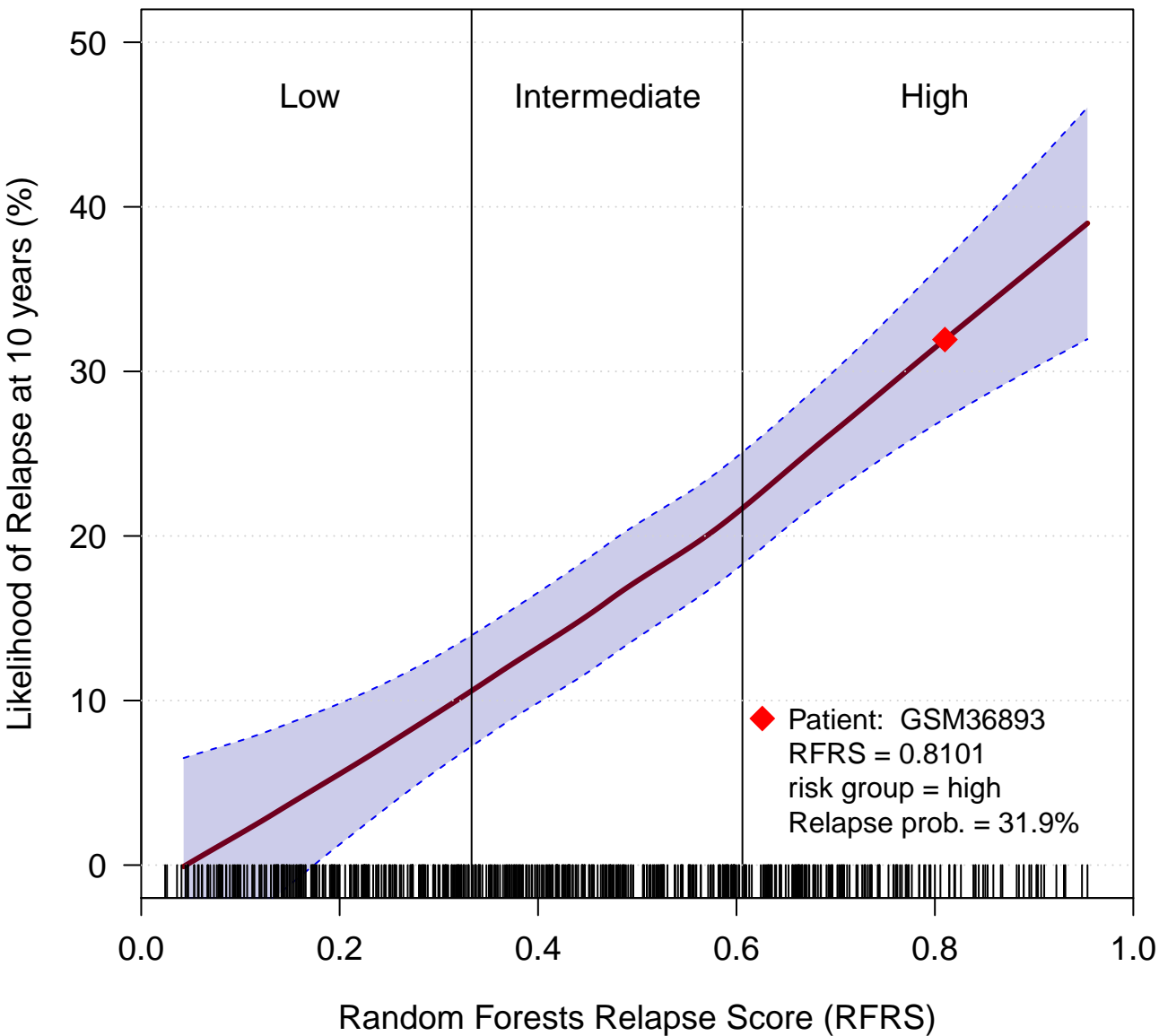

Supplement: Additional file 13 — Shows a sample patient report produced by the algorithm. [file gm496-S13.pdf]
